# Asymmetric synthesis of chiral β-alkynyl carbonyl and sulfonyl derivatives *via* sequential palladium and copper catalysis†
†Electronic supplementary information (ESI) available. See DOI: 10.1039/c6sc01724j


**DOI:** 10.1039/c6sc01724j

**Published:** 2016-06-10

**Authors:** Barry M. Trost, James T. Masters, Benjamin R. Taft, Jean-Philip Lumb

**Affiliations:** a Department of Chemistry , Stanford University , Stanford , CA 94305-5080 , USA . Email: bmtrost@stanford.edu

## Abstract

We present a full account detailing the development of a sequential catalysis strategy for the synthesis of chiral β-alkynyl carbonyl and sulfonyl derivatives. A palladium-catalyzed cross coupling of terminal alkyne donors with acetylenic ester, ketone, and sulfone acceptors generates stereodefined enynes in high yield. These compounds are engaged in an unprecedented, regio- and enantioselective copper-catalyzed conjugate reduction. The process exhibits a high functional group tolerance, and this enables the synthesis of a broad range of chiral products from simple, readily available alkyne precursors. The utility of the method is demonstrated through the elaboration of the chiral β-alkynyl products into a variety of different molecular scaffolds. Its value in complex molecule synthesis is further validated through a concise, enantioselective synthesis of AMG 837, a potent GPR40 receptor agonist.

## Abstract 2

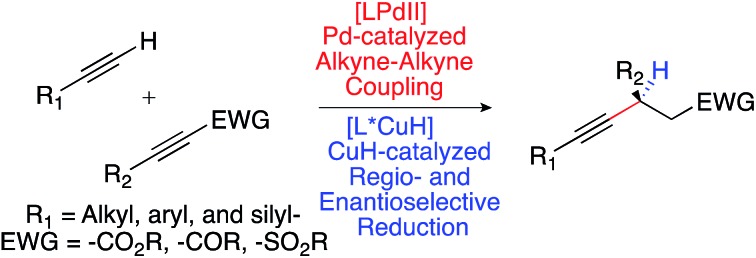
A sequential catalysis strategy for the synthesis of chiral β-alkynyl carbonyl and sulfonyl derivatives.

## Introduction

Conjugate addition reactions are fundamental bond forming processes that are widely employed in the synthesis of functionalized molecules.^1^ As a result, the development of efficient methods for asymmetric conjugate addition remains of paramount importance in modern organic synthesis. Transition metal catalysis is a well-established means of effecting the conjugate addition of a carbon nucleophile,^2^ and such catalytic processes often enable the rapid assembly of molecular complexity in a highly atom economic^3^ fashion.

**Scheme 1:**
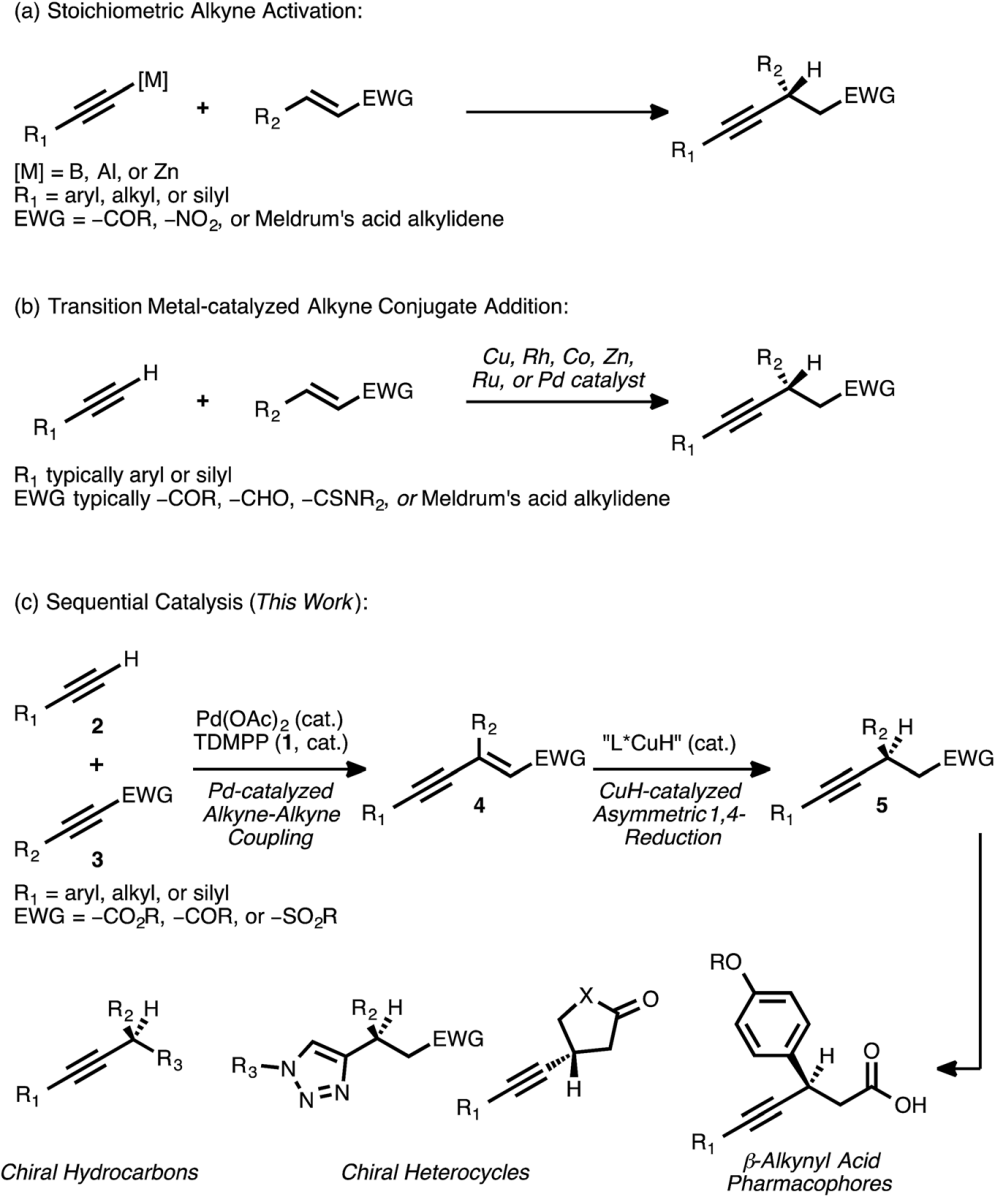
Strategies for the asymmetric conjugate addition of alkynes to activated olefins.

The asymmetric addition of a terminal alkyne to an activated olefin—for example, to an α,β-unsaturated ester, ketone, sulfone, aldehyde, or a synthetic equivalent thereof—is a conjugate addition reaction that has attracted significant attention. Novel methods involving the activation of a terminal alkyne as a more nucleophilic boron,^4^ aluminum,^5^ or zinc^6^ acetylide have been developed toward this end (Scheme 1A). However, strategies based on transition metal catalysis have the potential to improve functional group tolerance while avoiding the generation of stoichiometric metal waste.

Over the past several years, numerous methods for the transition metal-catalyzed asymmetric conjugate addition of a terminal alkyne to an activated olefin have been described (Scheme 1B). Carreira and co-workers reported the Cu-catalyzed addition of aryl acetylenes to Meldrum's acid alkylidenes with high yields and enantioselectivities,^7^ with an example of an alkyl acetylene addition that proceeded with more modest yield (29%) and enantioselectivity (68% ee).7*a* Shibasaki and co-workers disclosed the Cu-catalyzed addition of various terminal alkynes to α,β-unsaturated thioamides.^8^ Hayashi and Fillion have reported the asymmetric conjugate addition of silyl acetylenes to enones, enals, and Meldrum's acid alkylidenes under Rh^9^ and Co^10^ catalysis. Very recently, the asymmetric addition of triisopropylsilylacetylene to select α,β-unsaturated lactone and lactam substrates was accomplished using Rh catalysis, with diphenyl[(triisopropylsilyl)ethynyl]methanol serving as the alkynylating reagent.9*e* Blay and Pedro have reported asymmetric additions of primarily aryl acetylenes to arylidenediketones,^11^ coumarins,^12^ β-trifluoromethyl-α,β-unsaturated enones,^13^ and other electron-deficient enones^14^ under Zn or Cu catalysis. Mascareñas and co-workers recently described the Pd-catalyzed addition of various terminal alkynes to enones, enals, and acrylates in a racemic fashion, but investigations toward an asymmetric variant of the process were met with modest success (38–39% ee).^15^ In a similar vein, Ito and Nishiyama have reported racemic additions of terminal alkynes to different activated olefins using Ru pincer catalysts.^16^ These conditions were extended to one example of an asymmetric addition, the reaction of phenylacetylene with 3-penten-2-one (49% yield, 82% ee).

These reports illustrate how transition metal catalysis can be effective for the asymmetric conjugate addition of select types of alkynes to certain activated olefins. However, they also demonstrate how such direct approaches can be limited in terms of substrate scope. As these examples illustrate, the highest levels of reactivity are often observed when aryl or silyl acetylenes are employed as donors. Major restrictions regarding the scope of the acceptor olefin are also apparent. Significantly, no general strategy currently exists for the catalytic asymmetric addition of a terminal alkyne to an acyclic α,β-unsaturated ester. The catalytic asymmetric conjugate alkynylation of an α,β-unsaturated sulfone is, similarly, unknown.

A procedure to effect the formal asymmetric conjugate addition of a broad set of terminal alkynes to various acceptor olefins would complement these strategies. Importantly, the products obtained—chiral β-alkynyl carbonyl or sulfonyl derivatives—are valuable both on their own and as precursors to more functionalized molecules. For example, enantioenriched β-aryl, β-alkynyl carboxylic acids have been identified as potent pharmacophores and have been investigated for the treatment of type II diabetes.^17^ In addition, chemoselective^18^ functional group manipulations that engage either the alkyne group or the carbonyl group are possible. In an early illustration of this concept, Hayashi and co-workers reported the conversion of a β-alkynyl ketone to a 1,2,3-triazole through a Cu-catalyzed azide-alkyne cycloaddition; this transformation proceeded in high yield and without interference by the carbonyl moiety.9*b* The prospect of converting β-alkynyl carbonyl derivatives into different heterocyclic scaffolds *via* such catalytic processes is attractive, and it further motivates interest in a general method for the synthesis of these compounds.

Recognizing the need for a broadly applicable method for the synthesis of chiral β-alkynyl carbonyl and sulfonyl derivatives—and cognizant of the rich synthetic utility associated with these products—we considered a new approach. We envisioned that the subject compounds might be accessible through a sequential catalysis process involving: (a) a Pd-catalyzed cross coupling of alkynes to generate stereodefined enynes, and (b) a regio- and enantioselective CuH-catalyzed asymmetric conjugate reduction of these intermediates (Scheme 1C).

Our research group has established that combinations of palladium acetate and tris(2,6-dimethoxyphenylphosphine) (TDMPP, **1**) can efficiently promote the cross coupling of a diverse set of terminal alkynes **2** with various acceptor alkynes **3** (EWG = –CO_2_R, –COR, –SO_2_Ph).^19^ In this completely atom-economic addition reaction, the enyne products **4** are typically formed in very high yields. Except in certain rare cases,^20^ this reaction proceeds with complete regio- and stereoselectivity, yielding solely the enyne isomer shown. The ability to generate stereodefined olefins in this manner is crucial to the proposed second stage of transition metal-catalyzed conjugate reduction, as a reduction reaction performed using a mixture of olefin isomers would be expected to result in poor enantioselectivity.^21^

Although CuH-catalyzed asymmetric conjugate reductions of α,β-unsaturated esters, ketones, and sulfones are known,^22^ the extended π-system borne by an activated enyne of the type **4** presents a critical issue of regioselectivity. Such a substrate has the potential to undergo an undesired 1,6-reduction of the alkyne group in competition with the desired 1,4-reduction event. Indeed, previous research has revealed that Cu-mediated conjugate addition and reduction reactions of activated enynes typically proceed with high selectivity for the 1,6-pathway.^23^ A 1,4-selective asymmetric conjugate reduction of an activated enyne would thus constitute a reversal of typical reaction trends, and it would provide direct access to the chiral β-alkynyl product of interest. Combining such a selective reduction event with the Pd-catalyzed coupling of alkynes would thus provide efficient access to a broad range of chiral β-alkynyl carbonyl and sulfonyl derivatives starting from simple alkyne precursors.

We previously reported preliminary results toward this end, highlighting the utility of dual Pd and CuH catalysis in the synthesis of selected examples of chiral β-alkynyl esters.^24^ Herein, we provide a comprehensive account describing not only the development of that process but also further progress in this area. These subsequent studies have enabled significant expansions in both the scope and the synthetic utility of this sequential catalysis strategy. Specifically, the detailed reaction optimization efforts that revealed the critical influence of ligand selection on the regioselectivity of the reduction event are presented. The application of the optimized reaction conditions to the synthesis of many new chiral β-alkynyl esters bearing valuable functionality to truly highlight the selectivity now demonstrates the very broad breadth of the methodology. We show, for the first time, the introduction of further structural diversity at the enyne stage *via* Pd^0^-catalyzed allylic alkylation followed by the asymmetric reduction. Moreover, efficient syntheses of chiral β-alkynyl ketones and sulfones *via* the sequential catalysis approach are reported here for the first time. The chemoselective elaboration of these latter products provides access to new chemical scaffolds that are not readily accessible from the corresponding β-alkynyl esters. In addition, the value of the method in a more complex molecular setting is demonstrated through a concise, asymmetric synthesis of a β-alkynyl, β-aryl carboxylic acid derivative of pharmaceutical relevance.

## Results and discussion

### Reaction optimization

**Scheme 2:**
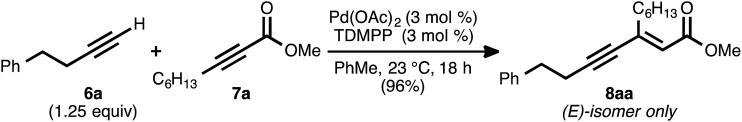
Pd-catalyzed alkyne–alkyne coupling to generate a model substrate.

Our reaction discovery efforts began with the preparation of a model enyne *via* the Pd-catalyzed alkyne–alkyne coupling reaction. Given the lack of known methods for the direct asymmetric addition of alkynes to acyclic α,β-unsaturated esters, we were attracted to propiolates as acceptor alkynes. The Pd-catalyzed alkyne–alkyne coupling was thus performed using 4-phenyl-1-butyne (**6a**) as the donor alkyne and methyl 2-nonynoate (**7a**) as the acceptor alkyne (Scheme 2).19*b* Ynenoate **8aa** was obtained as a single regio- and stereoisomer in excellent yield (96%).

**Fig. 1:**
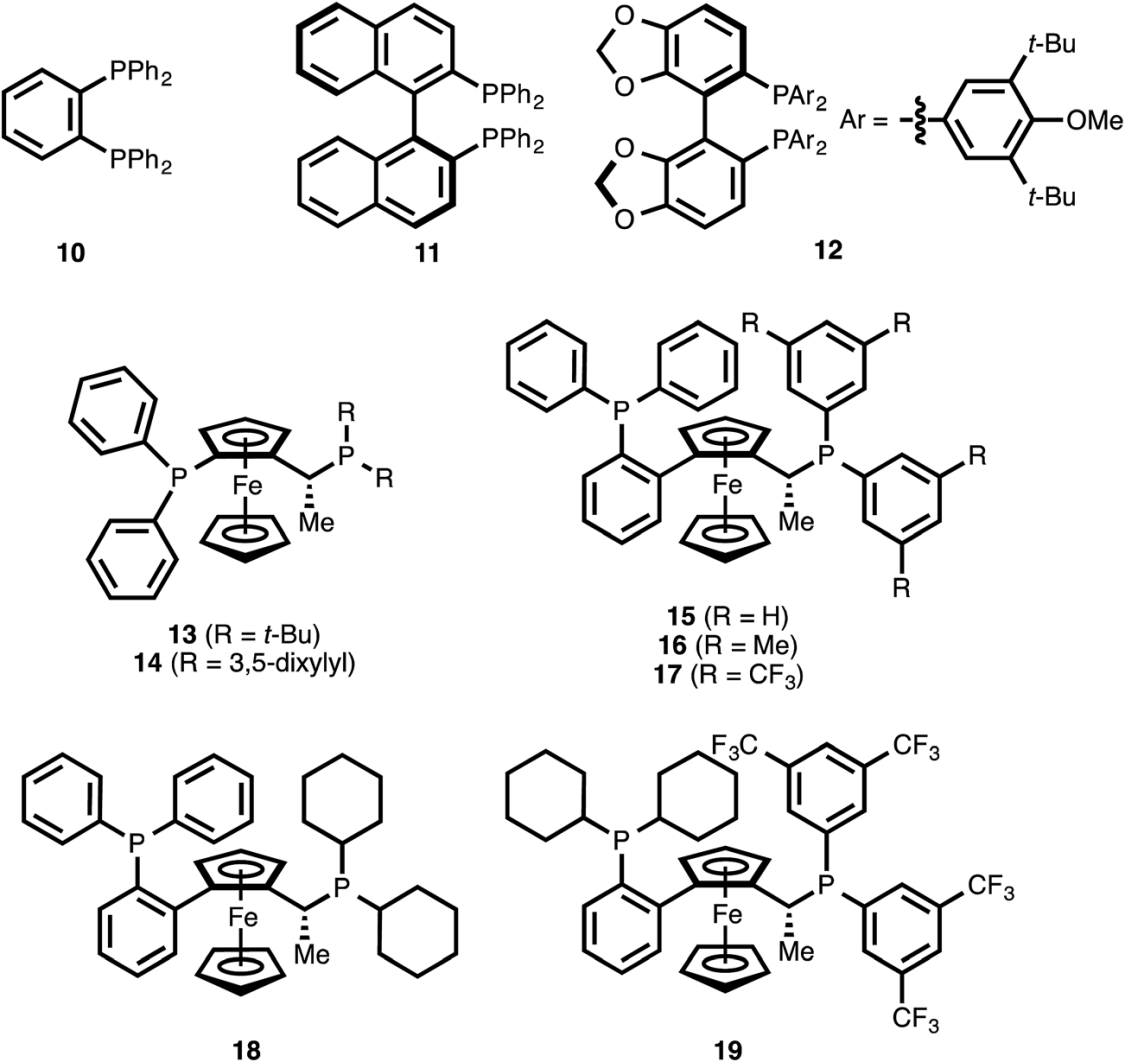
Ligands examined in the CuH-catalyzed conjugate reduction of ynenoate **8aa**.

The asymmetric conjugate reduction stage was then explored, using **8aa** as the substrate of interest. Drawing on prior reports of CuH-catalyzed reductions, Cu(OAc)_2_·H_2_O was selected as the Cu pre-catalyst, diethoxymethylsilane was used as the stoichiometric reductant, and the reactions were conducted in toluene at 4 °C.^22^ In addition, *tert*-butanol was included in the reaction mixture, as it had been reported that this additive could improve the rate of conjugate reduction reactions.22c,d We anticipated that the steric and electronic properties of the bisphosphine ligand might significantly impact the regioselectivity of the process. To this end, the nature of the ligand was systematically varied in these optimization experiments, which were assayed in terms of reaction conversion and regioselectivity (Table 1 and Fig. 1).

**Table 1:** Optimization of the asymmetric CuH-catalyzed conjugate reduction of ynenoate **8aa** to β-alkynyl ester **9aa**

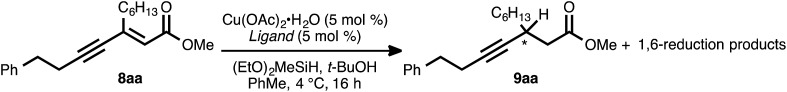			
Entry	Ligand	Conversion^*a*^ (%)	1,4 : Σ1,6 selectivity^*b*^
1	**10**	91	1.3 : 1
2	**11**	58	1 : 8.6
3	**12**	71	1 : 5.1
4	**13**	92	1 : 1.3
5	**14**	93	1.1 : 1
6	**15**	60	1 : 3.7
7	**16**	80	1 : 4.6
**8**	**17**	**>95**	**>19 : 1 (99% ee)**
9	**18**	10	N. D.
10	**19**	11	N. D.

^*a*^Conversion was determined by ^1^H NMR analysis of the crude reaction mixture relative to mesitylene as an internal standard.

^*b*^Determined by ^1^H NMR analysis of the crude reaction mixture. N. D. = not determined.

The use of the achiral ligand 1,2-bis(diphenylphosphino)benzene (**10**)^25^ led to high conversion of ynenoate **8aa**, but the 1,4-reduction product—β-alkynyl ester **9aa**—was obtained with only moderate selectivity (entry 1). The mass balance comprised an intractable mixture of compounds, the ^1^H NMR spectra of which were consistent with 1,6-reduction byproducts. When axially chiral, *C*_2_-symmetric ligands such as BINAP22*b* (**11**) or DTBM-SEGPHOS (**12**)22*d* were employed, significant decreases in both conversion and 1,4-selectivity were observed (entries 2 and 3). The complexity of these product mixtures, together with the fact that the various products were inseparable *via* chromatography, precluded a determination of the enantioselectivity of the 1,4-reduction event. The use of ferrocene-derived JOSIPHOS ligands^26^ led to more promising results. Specifically, both *tert*-butyl- and xylyl-derived ligands **13** and **14** delivered high conversion, and they provided the highest levels of regioselectivity observed among the chiral ligands examined up this point (entries 4 and 5).

The fact that xylyl-substituted ligand **14** delivered a greater level of 1,4-selectivity than did *tert*-butyl-substituted ligand **13** suggested that aromatic substitution on the eastern portion of the bisphosphine might be beneficial for 1,4-selectivity. It was therefore postulated that modulation of this specific aromatic subunit might yield improved 1,4-selectivity. To this end, various commercially available ligands of the related WALPHOS ligand family (**15–19**) were examined. Relative to the JOSIPHOS ligands, the WALPHOS series offers greater variation in terms of aromatic substituents on the easternmost phosphine. However, these ligands differ from those in the JOSIPHOS family in that the western portion of the ferrocene unit is not bonded directly to the phosphorous atom but is, instead, annealed onto the 2-position of a triphenylphosphino unit. This gross structural change on its own was sufficient to direct the reduction event toward the 1,6-pathway (entry 6 *vs.* entry 5). That effect was magnified upon replacement of this eastern phenyl substituent with a xylyl unit (entry 7 *vs.* entries 5 and 6). However, a significant electronic influence was observed within the WALPHOS series: the use of the 3,5-bis(trifluoromethyl) WALPHOS ligand **17** delivered quantitative reaction conversion, essentially complete 1,4-selectivity, and excellent enantioselectivity (99% ee, entry 8).

One possible explanation for this result is that the electron-withdrawing nature of ligand **17** generates a more Lewis acidic Cu catalyst, one that is better able to coordinate to the carbonyl oxygen atom of ynenoate **8aa**. Such secondary binding could help to promote the 1,4-reduction event by increasing the electrophilicity of the β-carbon or by directing hydride delivery to this position through a template effect.

It is noteworthy that both the western triphenylphosphino substituent and the eastern bis(3,5-bis(trifluoromethyl)phenyl) groups of ligand **17** were necessary for reactivity: the use of the related bis(dicyclohexyl)phosphino ligands **18** and **19** resulted in low levels of reaction conversion (entries 9 and 10).

### Synthesis of chiral β-alkynyl esters

With the optimized conditions for the asymmetric conjugate reduction stage established, the scope of this new synthesis of chiral β-alkynyl esters was investigated. A range of donor alkynes **6** was examined, using methyl 2-nonynoate (**7a**) as the acceptor alkyne (Table 2). In addition to alkyl alkyne **6a**, various aryl alkyne donors reacted successfully, including electron-neutral, electron-rich, and electron-deficient examples. Of particular note is the fact that *p*-bromophenylacetylene (**6d**) underwent both the Pd^II^-catalyzed alkyne–alkyne coupling and the Cu^I^-catalyzed reduction without competitive oxidative addition. Silyl substituted donor alkynes were also compatible, with both benzyldimethylsilylacetylene (**6e**) and trimethylsilylacetylene (**6f**) reacting smoothly. In the latter case, the corresponding trimethylsilylacetylene-derived β-alkynyl ester (**9fa**) was identified after the reduction stage (^1^H NMR analysis of an aliquot), but the crude product was treated with TBAF to liberate the terminal alkyne. The latter was isolated in very good yield after column chromatography. The more sterically encumbered donor alkyne **6g** was also employed without complication.

**Table 2:** Substrate scope in the synthesis of β-alkynyl esters (donor alkynes)^*a*^

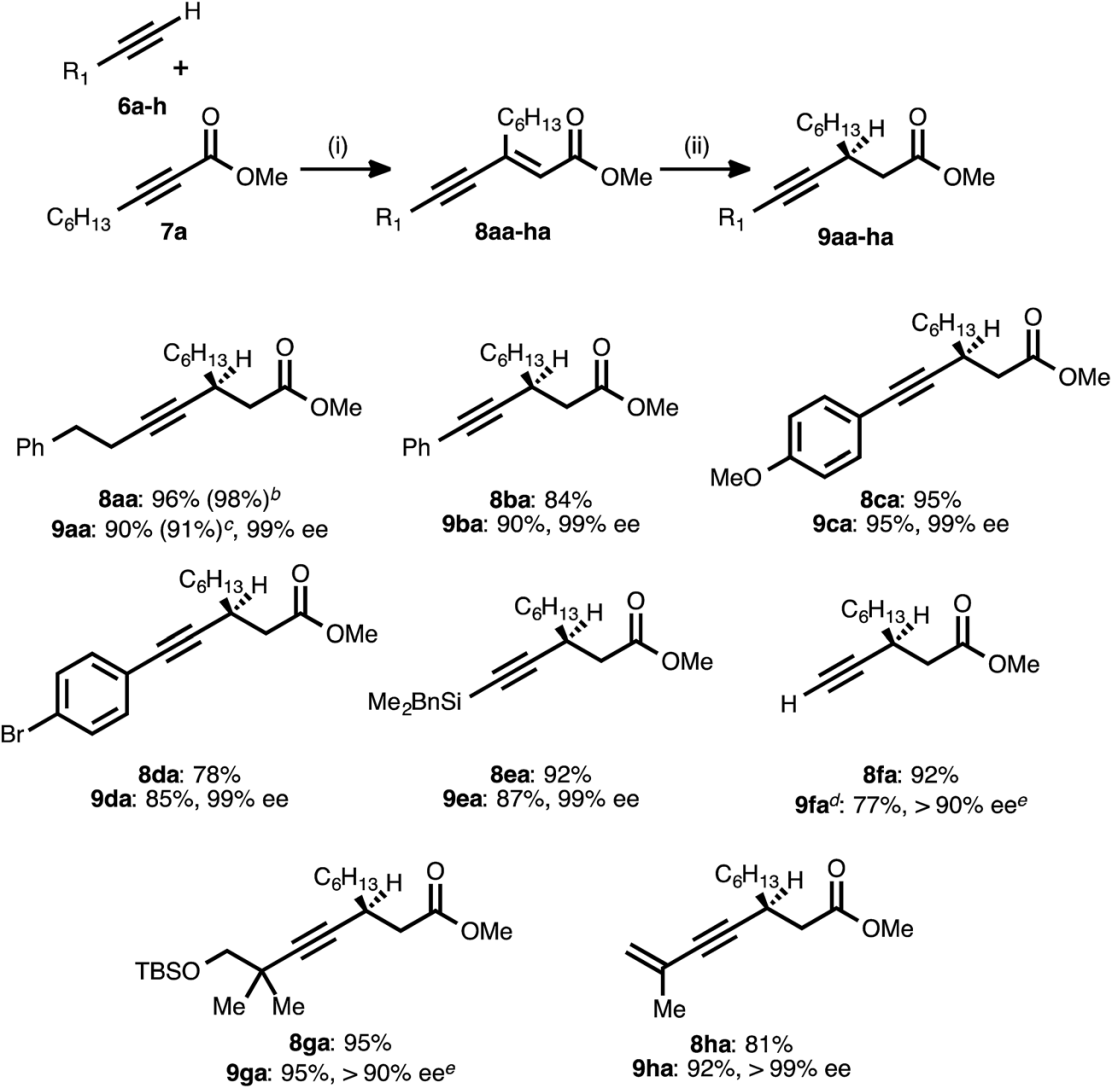

^*a*^Reaction conditions: (i) **6a–h** : **7a** ratio = 1.25–1.75 : 1.0, 3 mol% Pd(OAc)_2_/TDMPP, 1.0 M in PhMe at 23 °C for 1–18 h. (ii) 5 mol% Cu(OAc)_2_·H_2_O/**17**, (EtO)_2_MeSiH (2 equiv.), *t*-BuOH (2 equiv.), 0.20 M in PhMe at 4 °C for 14–20 h. Yields are of isolated products after chromatography. Enatiomeric excesses were determined by chiral HPLC analysis.

^*b*^Result obtained from a 15 mmol-scale reaction using 1.5 mol% Pd(OAc)_2_/TDMPP.

^*c*^Result obtained from a 10 mmol-scale reaction using 2.5 mol% Cu(OAc)_2_ mol% Cu(OAc)_2_·H_2_O/**17**.

^*d*^The crude reduction product was treated with TBAF (1 M in THF), and the corresponding terminal alkyne (R_1_ = H) was isolated after chromatography.

^*e*^The ee was determined after conversion of the product into a diastereomeric mixture of amides using (*S*)-α-methylbenzylamine (>95 : 5 dr observed).

To further probe the regioselectivity of the reduction stage, an ynenoate bearing an extended (1,7-) π-system was prepared using enynyl donor alkyne **6h**. The CuH-catalyzed reduction of this substrate maintained excellent regioselectivity, delivering 1,4-reduction product **9ha** in high yield and with excellent enantioselectivity.

To further assess reaction robustness and scalability, each stage was examined on larger scale using a reduced catalyst loading. A 15 mmol-scale coupling between donor **6a** and acceptor **7a** was performed using 1.5 mol% Pd(OAc)_2_/TDMPP, and this delivered ynenoate **8aa** in 98% yield. A 10 mmol-scale conjugate reduction of the latter compound was performed using 2.5 mol% Cu(OAc)_2_·H_2_O/**17**, and this furnished ester **9aa** in 91% yield and 99% ee. Importantly, these results meet or exceed those obtained on smaller scale (1.0 mmol and 0.30 mmol, respectively) and with higher catalyst loadings.

Attention next turned to the scope of the process with regard to other propiolate acceptors, using 4-phenyl-1-butyne (**6a**) as the donor alkyne (Table 3). The steric bulk around the ester unit could be increased without ill effect: in addition to the methyl ester examples shown previously, ethyl and benzyl esters reacted smoothly in both stages and delivered excellent enantioselectivity. The sterically demanding, cyclopropyl-derived acceptor **7d** also underwent the alkyne–alkyne coupling in high yield. When the resulting ynenoate (**8ad**) was subjected to the conjugate reduction stage, the reaction reached 80% conversion and delivered a 4 : 1 mixture of 1,4- to 1,6-reduction products from which pure **9ad** was isolated in 54% yield. Importantly, the enantioselectivity of the event remained high (99% ee). In addition to these hydrocarbon-substituted acceptor alkynes, a propiolate bearing a primary alkyl chloride was prepared and was found to react smoothly in both stages.

**Table 3:** Substrate scope in the synthesis of β-alkynyl esters (acceptor alkynes)^*a*^

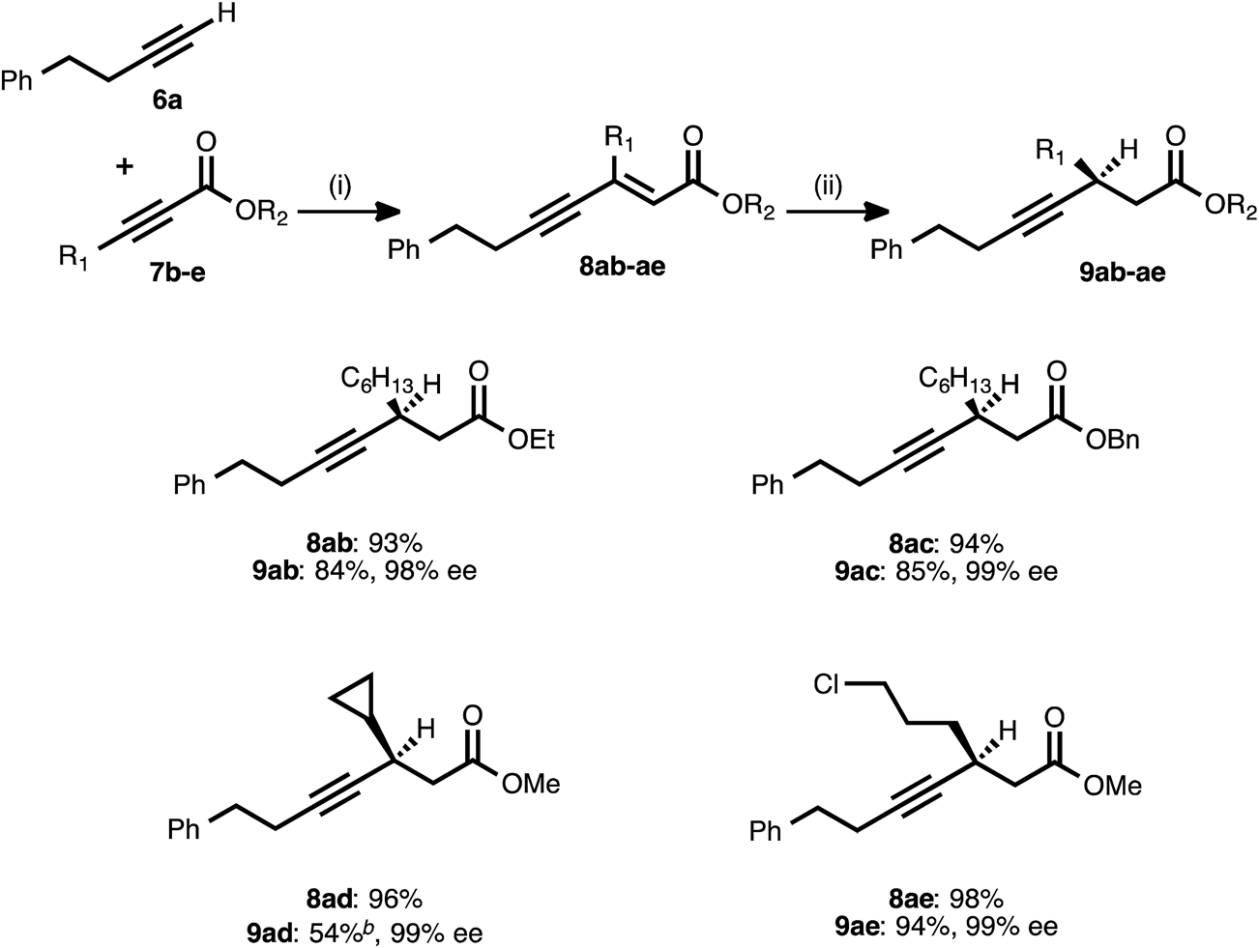

^*a*^Reaction conditions: (i) **6a** : **7b–e** ratio = 1.25 : 1.0, 3 mol% Pd(OAc)_2_/TDMPP, 1.0 M in PhMe at 23 °C for 1–18 h. (ii) 5 mol% Cu(OAc)_2_·H_2_O/**17**, (EtO)_2_MeSiH (2 equiv.), *t*-BuOH (2 equiv.), 0.20 M in PhMe at 4 °C for 14–20 h. Yields are of isolated products after chromatography. Enantiomeric excesses were determined by chiral HPLC analysis.

^*b*^The reaction reached 80% conversion (^1^H NMR) and produced a mixture of 1,4- and 1,6-reduction products (4 : 1 1,4 : Σ1,6, ^1^H NMR analysis of the crude reaction mixture), from which pure **9ad** was isolated in 54% isolated yield.

The fact that the conjugate reduction of β-cyclopropyl-substituted ynenoate **8ad** proceeded with slight decreases in efficiency and regioselectivity can be understood in terms of the increased steric clash associated with the coordination of the Cu catalyst to this more sterically congested olefin. These interactions could be expected to retard the rate of hydride addition, and they also could cause hydride addition across the less sterically demanding alkyne unit to become more favorable.^27^ A more surprising change in regiochemical outcome came during the examination of ynenoates bearing alkyl groups of different chain lengths at their β-positions (Table 4). When methyl substituted propiolate **7f** was coupled with phenylacetylene (**6b**) and the resulting ynenoate was subjected to the standard reduction conditions, 2.3 : 1 1,4 : Σ1,6 regioselectivity was observed. The conjugate reduction of the β-ethyl congener (**8bg**) led to a similar outcome (2.7 : 1 1,4 : Σ1,6). However, a marked increase in regioselectivity (12.5 : 1 1,4 : Σ1,6) was observed in the reaction of the β-*n*-propyl analogue (**8bh**). As previously described, excellent regioselectivity (>19 : 1) was observed upon moving to longer chain lengths (*e.g.*, the reactions of β-chloropropyl substrate **8ae** or β-hexyl compound **8aa**). While a detailed explanation for this phenomenon remains elusive, a trend of increasing 1,4-selectivity with increasing chain length clearly emerges from these data.

**Table 4:** Substrate scope in the synthesis of β-alkynyl esters (acceptor alkynes of different chain lengths at the β-position)^*a*^

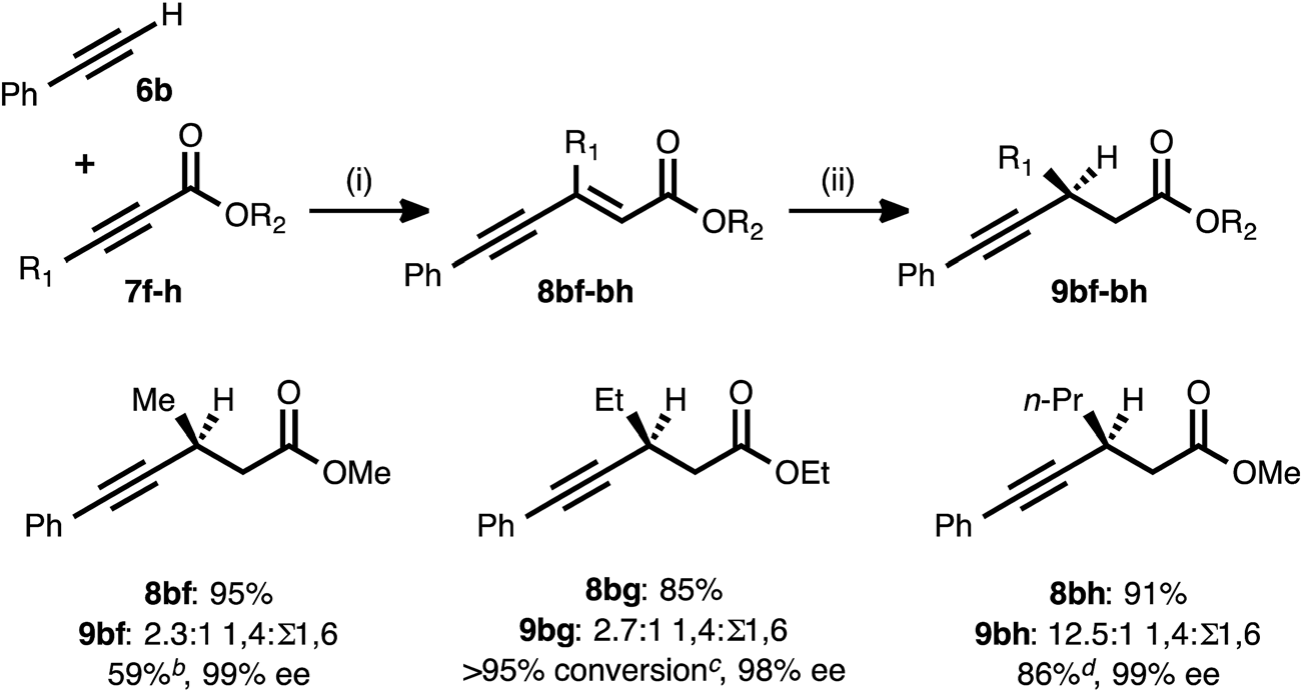

^*a*^Reaction conditions: (i) **6b** : **7f–h** ratio = 1.25 : 1.0, 3 mol% Pd(OAc)_2_/TDMPP, 1.0 M in PhMe at 23 °C for 1–6 h. (ii) 5 mol% Cu(OAc)_2_·H_2_O/**17**, (EtO)_2_MeSiH (2 equiv.), *t*-BuOH (2 equiv.), 0.20 M in PhMe at 4 °C for 14–16 h. Yields are of isolated products after chromatography. Ratios of 1,4 : 1,6 selectivity were determined by ^1^H NMR analysis of the crude reaction mixture. Enantiomeric excesses were determined by chiral HPLC analysis.

^*b*^Isolated yield of analytically pure 1,4-reduction product **9bf** obtained from the crude 2.3 : 1 mixture of regioisomeric products.

^*c*^Quantitative reaction conversion and yield were obtained, but 1,4-reduction product **9bg** could not be separated from the various 1,6-reduction products.

^*d*^Isolated yield of **9bh** (>90% purity) from the crude 12.5 : 1 mixture of regioisomeric products.

**Scheme 3:**
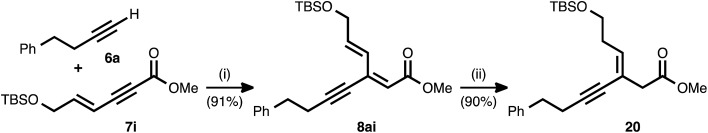
Regio- and stereoselective reduction of a substrate featuring an extended π-system. Conditions: (i) 3 mol% Pd(OAc)_2_/TDMPP, 1.0 M in PhMe at 23 °C for 16 h. (ii) 5 mol% Cu(OAc)_2_·H_2_O/**17**, (EtO)_2_MeSiH (2 equiv.), *t*-BuOH (2 equiv.), 0.20 M in PhMe at 4 °C for 16 h.

As shown in Table 2, the conjugate reduction of an ynenoate derived from an enynyl alkyne donor maintained excellent 1,4-selectivity. To further study the behavior of extended π-systems in the method, enynyl alkyne acceptor **7i** was prepared (Scheme 3). This compound reacted smoothly with 4-phenyl-1-butyne (**6a**), delivering ynenoate **8ai**. To our surprise, the reduction of the latter substrate using ligand **17** generated, exclusively and in high yield, (*Z*)-enyne **20**. The same product was obtained when the reaction was performed using 1,2-bis(diphenylphosphino)benzene (**10**) as the ligand. The outstanding regio- and stereoselectivity observed in this case is remarkable, and it may be the result of a multi-dentate coordination event involving the Cu catalyst and both the alkyne and olefin subunits of this unique, cross-conjugated substrate.

Propiolate acceptors bearing heteroatom substitution were next examined. Initial efforts focused on the reaction of carbonate-bearing propiolate **7j** with different donor alkynes (Table 5). Cross couplings between this acceptor and 4-phenyl-1-butyne (**6a**), phenylacetylene (**6b**), and benzyldimethylsilylacetylene (**6e**) all proceeded in good to very good yields. The ynenoates so obtained were smoothly reduced to the corresponding β-alkynyl esters in high yield, with >19 : 1 regioselectivity, and in 99% ee. Notably, there was no indication that competitive ionization or reduction of the propargylic or allylic carbonates occurred during either of the two transition metal-catalyzed reaction stages.

**Table 5:** Synthesis of β-alkynyl esters bearing carbonate functionality^*a*^

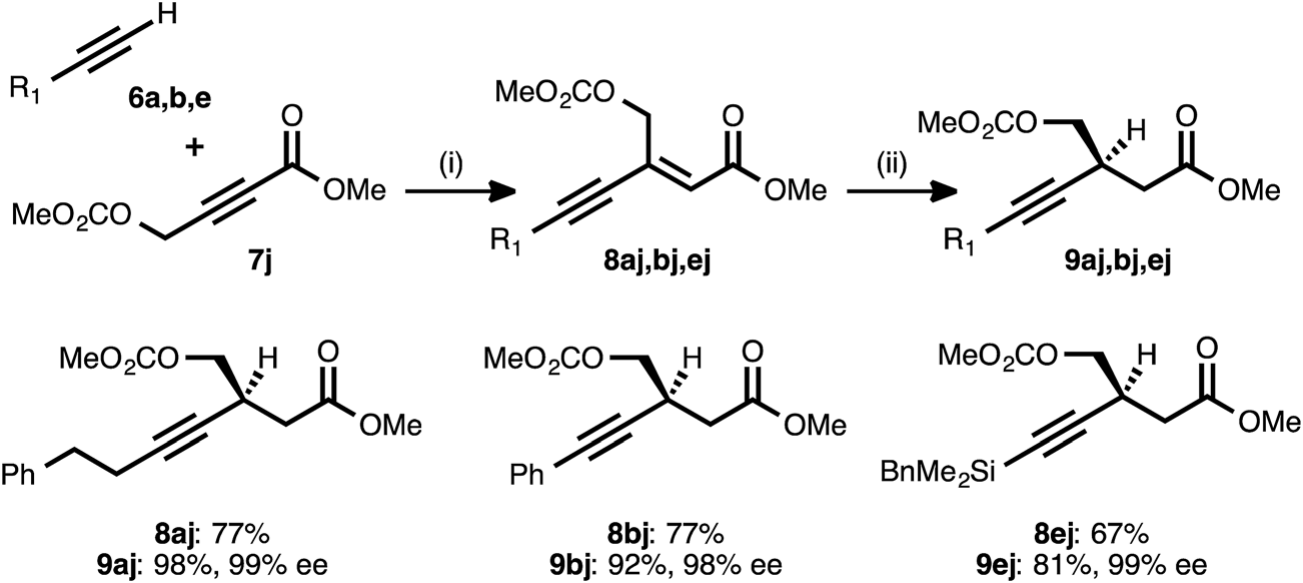

^*a*^Reaction conditions: (i) **6** : **7j** ratio = 1.25–1.75 : 1.0, 3 mol% Pd(OAc)_2_/TDMPP, 1.0 M in PhMe at 23 °C for 5–18 h. (ii) 5 mol% Cu(OAc)_2_·H_2_O/**17**, (EtO)_2_MeSiH (2 equiv.), *t*-BuOH (2 equiv.), 0.20 M in PhMe at 4 °C for 16–20 h. Yields are of isolated products after chromatography. Enantiomeric excesses were determined by chiral HPLC analysis.

To explore the use of nitrogen-bearing acceptors in the method, propargylamine derivative **7k** was prepared. Coupling reactions between this acceptor and donor alkynes **6a**, **6b**, and **6e** all proceeded smoothly, even when performed with a lower catalyst loading (1.5 mol%) and under shorter reaction times (1–3 h, Table 6).^28^ However, the conjugate reduction of the resulting ynenoates using the standard conditions (with ligand **17**) furnished β-alkynyl esters with more modest regio- and enantioselectivity. As a representative example, the reduction of ynenoate **8ak** yielded ester **9ak** with 9 : 1 1,4 : Σ1,6 selectivity and 70% ee. A survey of different ligands led to the discovery that JOSIPHOS ligand **13** was particularly well matched for the reduction of these ynenoates: when it was used in place of ligand **17**, excellent regioselectivity (>19 : 1) and enantioselectivity (≥92% ee) were obtained for the range of substrates. The decreased selectivity associated with the electron-deficient WALPHOS ligand **17** may have resulted from secondary interactions between the copper catalyst and the Lewis basic carbamate group, interactions that may have been disfavored when the more electron-rich and sterically encumbered JOSIPHOS ligand **13** was used.

**Table 6:** Synthesis of nitrogen substituted β-alkynyl esters^*a*^

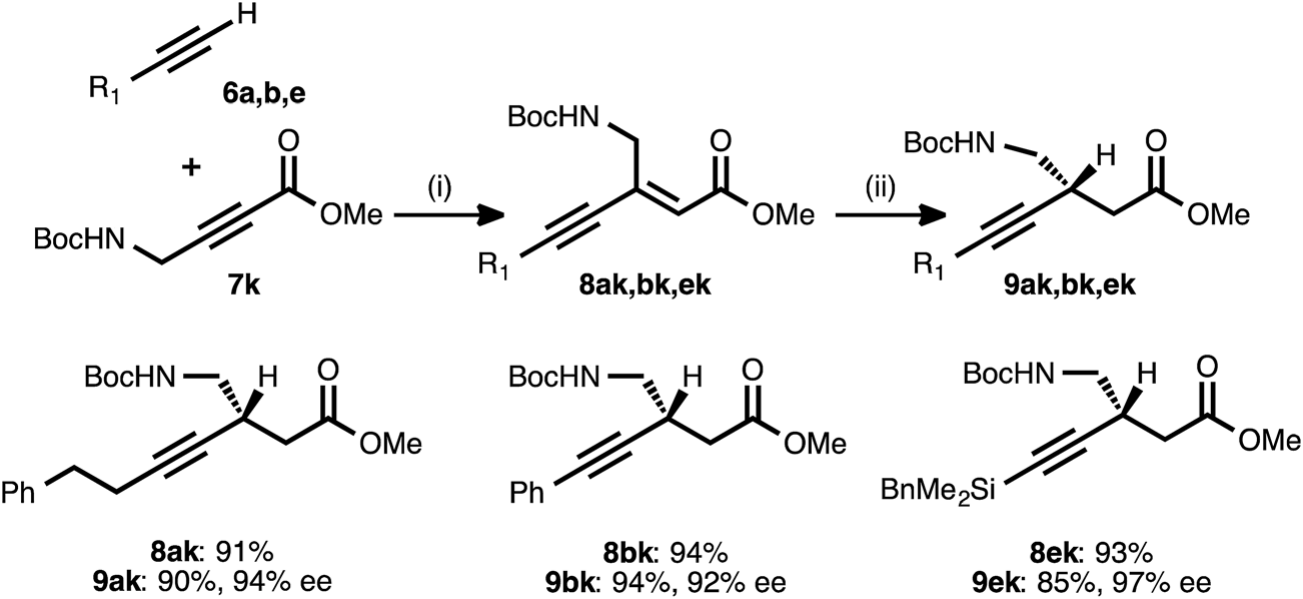

^*a*^Reaction conditions: (i) **6** : **7k** ratio = 1.25–1.75 : 1.0, 1.5 mol% Pd(OAc)_2_/TDMPP, 1.0 M in PhMe at 23 °C for 1–3 h. (ii) 2 mol% Cu(OAc)_2_·H_2_O/**13**, (EtO)_2_MeSiH (1.5 equiv.), *t*-BuOH (1.5 equiv.), 0.20 M in PhMe at 4 °C for 4 h. Yields are of isolated products after chromatography. Enantiomeric excesses were determined by chiral HPLC analysis.

### Synthesis of chiral β-alkynyl esters *via* the Pd-catalyzed allylic alkylation of an ynenoate

As noted, carbonate-substituted ynenoate **8aj** underwent both the Pd^II^- and Cu^I^-catalyzed reaction stages without competitive ionization. However, we questioned whether this ionization event could be intentionally induced under Pd^0^ catalysis, with the concomitant formation of a π-allylpalladium complex. Subsequent trapping of this complex with a heteroatom nucleophilic would generate a new ynenoate, itself a candidate for asymmetric reduction. This catalytic allylic alkylation process would thus provide a convenient means for the convergent synthesis of additional, valuable ynenoates and chiral β-alkynyl esters.^29^

**Scheme 4:**
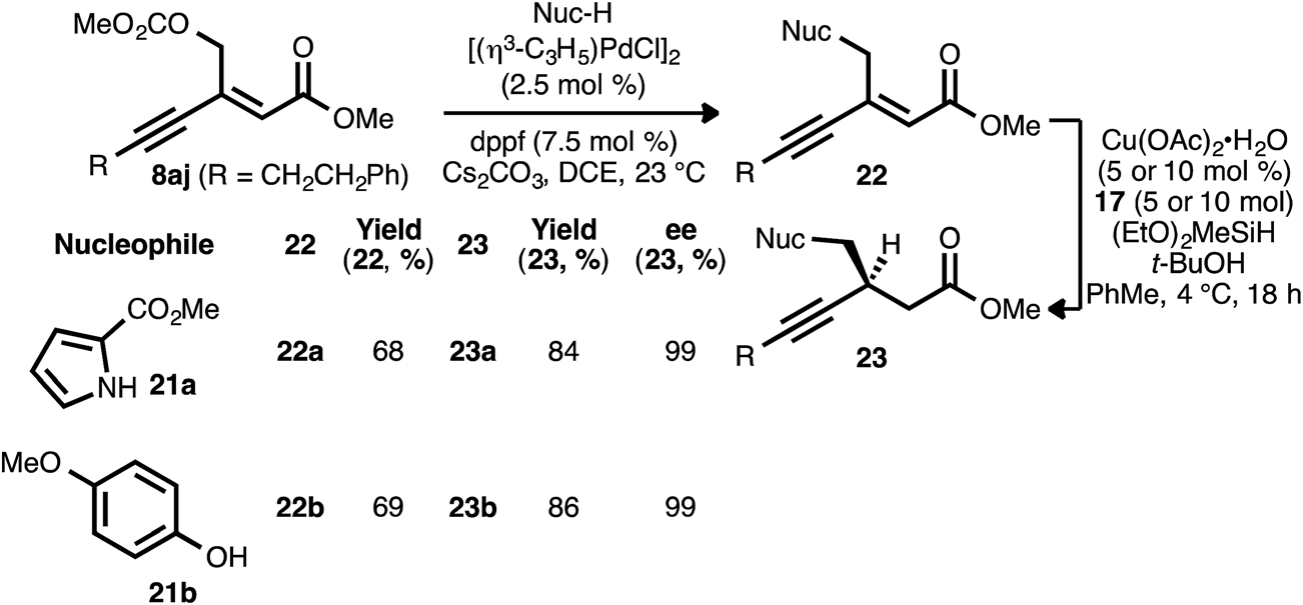
A Pd-AA approach to heteroatom-substituted β-alkynyl esters.

In pursuit of this, compound **8aj** was reacted with pyrrole nucleophile **21a** in the presence of Cs_2_CO_3_ and catalytic amounts of [(η^3^-C_3_H_5_)PdCl]_2_ and diphenylphosphinoferrocene (dppf) in 1,2-dichloroethane (Scheme 4).^30^ To our delight, desired allylic alkylation product **22a** was successfully formed as a mixture of olefin isomers (9 : 1 *Z* : *E*), and pure (*Z*)-**22a** was isolated in very good yield after chromatography. Following conjugate reduction using ligand **17**, β-alkynyl ester **23a** was obtained in high yield and enantioselectivity. Allylic substitution with phenol nucleophile **21b** likewise yielded oxygen-substituted ynenoate **22b**, in this case as a single olefin isomer (>19 : 1 *Z* : *E*). The asymmetric conjugate reduction of **22b** was similarly effective, giving rise to oxygen-substituted β-alkynyl ester **23b**.

### Synthesis of chiral β-aryl, β-alkynyl esters: total synthesis of AMG 837

**Scheme 5:**
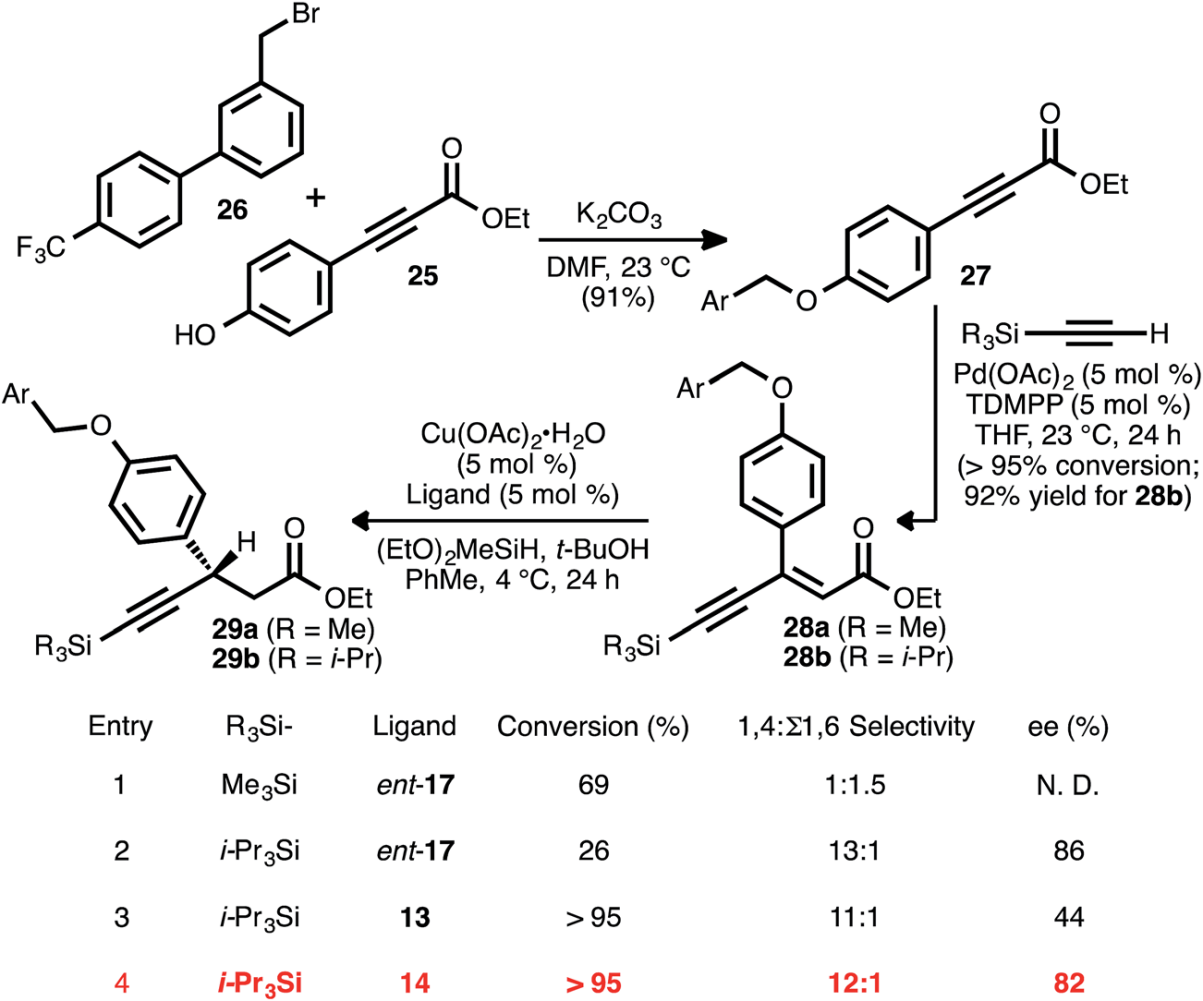
Synthesis of β-alkynyl esters **29a** and **29b***en route* to AMG 837 (**24**).

Recognizing the biological relevance of, specifically, β-aryl, β-alkynyl carboxylic acid derivatives, we were motivated to apply the present method to the synthesis of a representative compound within this subclass. As a target of interest, we selected AMG 837 (**24**), a GPR40 receptor agonist developed by Amgen, Inc.17c,^31^ The requisite acceptor alkyne was expediently prepared *via* the alkylation of known phenol **25** (ref. 32) with known bromide **26** (ref. 31*a*) (Scheme 5). Silyl-substituted acetylenes were chosen as alkyne coupling partners, as it was anticipated that the ethynylsilane moiety could be converted to the propynyl group of the target *via* desilylation and alkylation. To this end, the alkyne–alkyne coupling between trimethylsilylacetylene and propiolate **27** was performed, and this furnished ynenoate **28a** with >95% conversion. However, the conjugate reduction of this intermediate using ligand *ent*-**17** delivered predominantly 1,6-reduction products (1 : 1.5 1,4 : Σ1,6 selectivity, entry 1). We suspected that this might have been a consequence of the increased steric hindrance around the β-carbon of this substrate, analogous to that observed in the reaction of β-cyclopropyl substrate **8ad**. We questioned whether a corresponding increase in steric demand around the alkyne unit might serve to disfavor 1,6-reduction and redirect reactivity back toward the 1,4-pathway. Thus, the alkyne coupling reaction was performed using triisopropylsilylacetylene, delivering ynenoate **28b** in 92% isolated yield. Gratifyingly, the reduction of this compound using ligand *ent*-**17** proceeded with 13 : 1 regioselectivity in favor of the 1,4-pathway and with 86% ee, albeit with only 26% conversion (entry 2). Further ligand evaluation led us to discover that reactions promoted by JOSIPHOS ligands **13** and **14** proceeded with >95% conversion, maintained high regioselectivity, and, in the latter case, provided satisfying enantioselectivity (82% ee, entries 3 and 4).

**Scheme 6:**
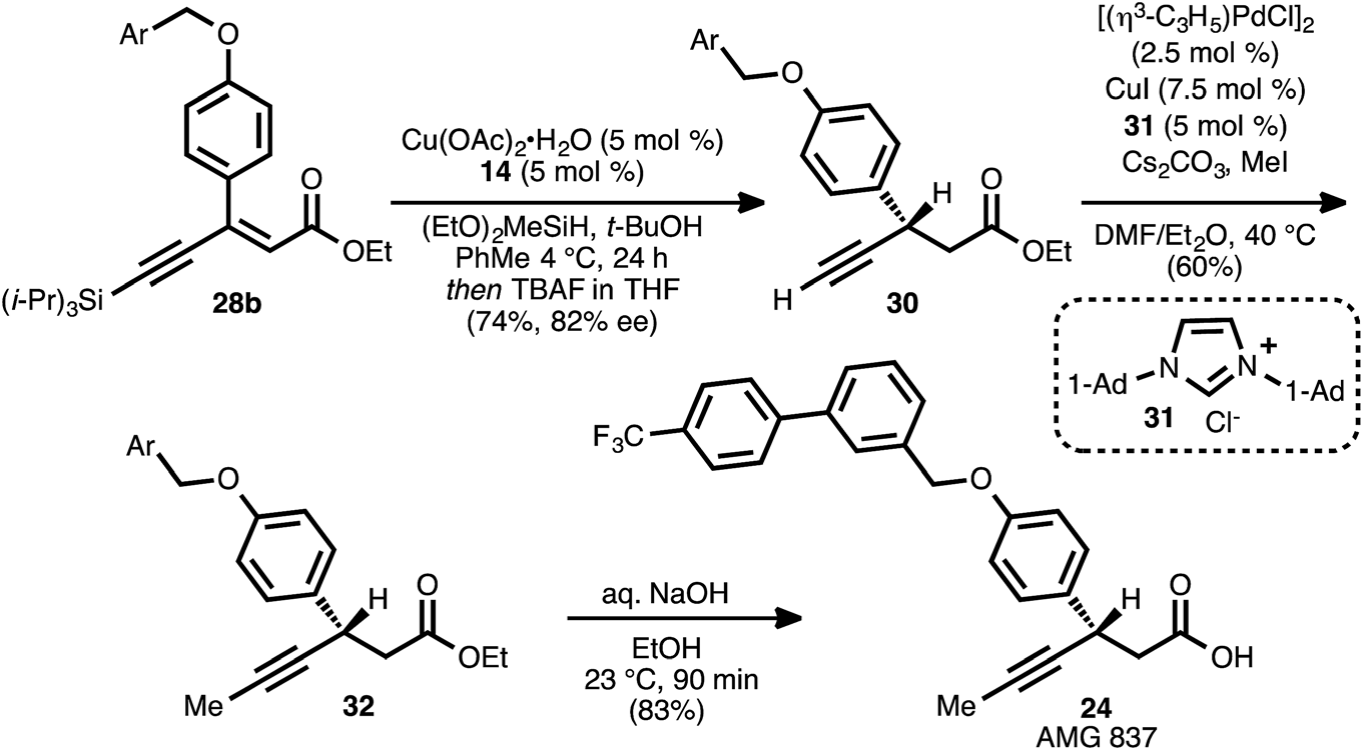
Completion of the synthesis of AMG 837 (**24**).

The reaction conditions of Scheme 5, entry 4 were incorporated into a one-pot conjugate reduction-alkyne desilylation process (Scheme 6). The conjugate reduction was carried out to completion, and then the reaction mixture was directly treated with tetrabutylammonium fluoride to effect alkyne desilylation. This sequence delivered terminal alkyne **30** in 74% yield from **28b** (82% ee). Alkyne methylation using Pd and Cu co-catalysis in the presence of NHC ligand **31** (ref. 8*b* and 33) furnished β-alkynyl ester **32**. Hydrolysis of the ethyl ester moiety of **32** proceeded smoothly, and AMG 837 (**24**) was obtained in 83% yield.

The brevity and efficiency of this synthesis, which was completed in five steps and 31% yield from compound **25**, stemmed from the use of a propiolate as a coupling partner. By directly employing an ester-derived acceptor, the need to unmask an ester equivalent (*e.g.*, a Meldrum's acid alkylidene) during the synthetic sequence was avoided. Moreover, the successful synthesis of this target highlights the applicability of the present method toward the specific subclass of β-aryl, β-alkynyl esters.

### Synthesis of chiral β-alkynyl ketones

The success achieved in the synthesis of chiral β-alkynyl esters from terminal alkynes and propiolates prompted us to examine other classes of activated alkynes as acceptors. Ketone-derived acceptors (ynones) were readily engaged as partners in the alkyne–alkyne coupling, giving rise to a range of ynenone products (Table 7). These compounds were then exposed to the reduction conditions developed for ynenoates, with one modification: *tert*-butanol was omitted as an additive, as it has been shown to promote 1,2-reduction in reactions of enones.22*e* Under these modified conditions, asymmetric hydrosilylation of the ynenone substrates occurred, giving rise to silyl enol ether intermediates. The addition of either tetrabutylammonium fluoride or methanolic HCl rapidly effected desilylation, and the corresponding β-alkynyl ketones were isolated in good yields and with excellent levels of enantioselectivity. Alkyl, aryl, and silyl donor alkynes were all compatible with this process, as were ynone acceptors bearing methyl, ethyl, and cyclohexyl substituents.

**Table 7:** Synthesis of β-alkynyl ketones *via* sequential catalysis^*a*^

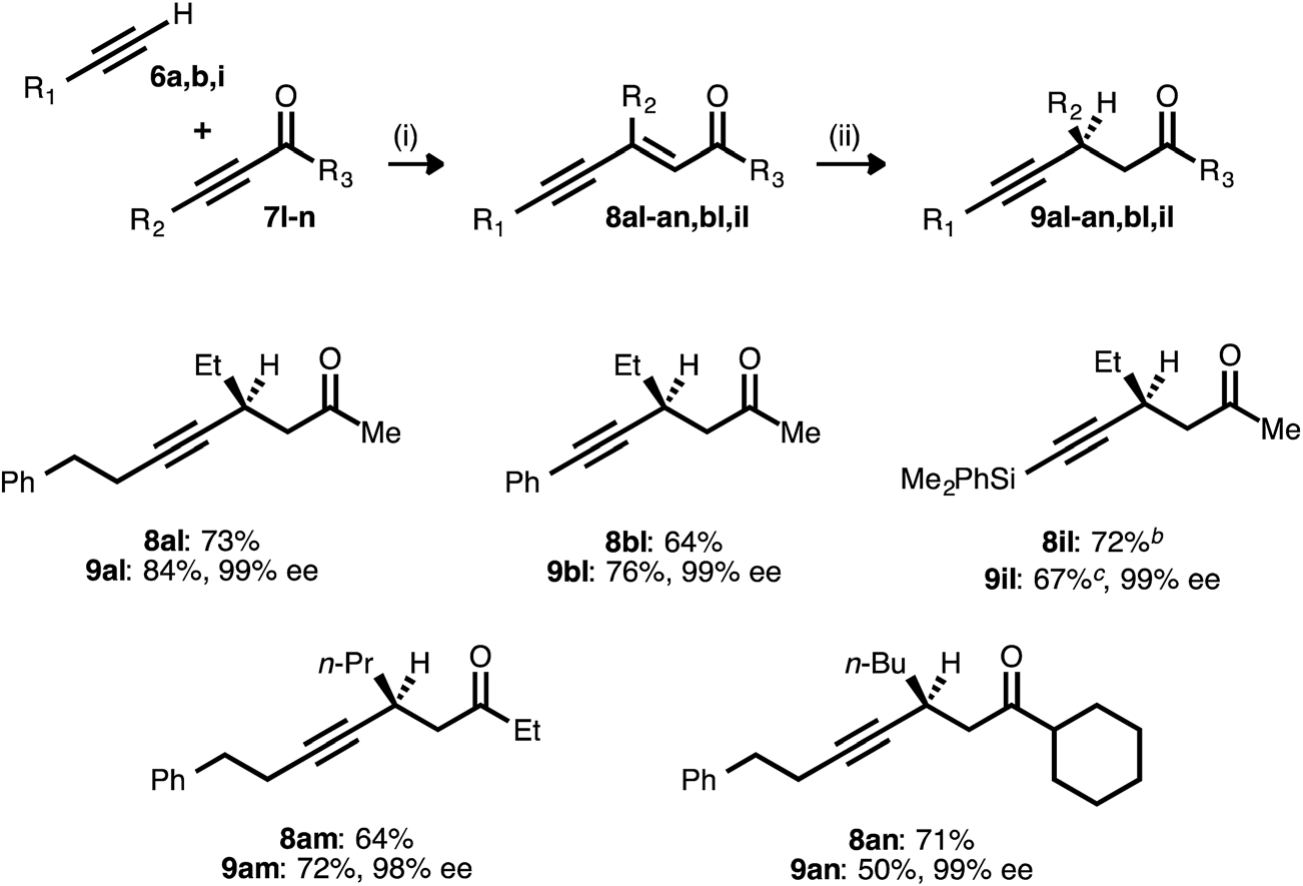

^*a*^Reaction conditions: (i) **7** : **6** ratio = 1.5–2.0 : 1.0, 5 mol% Pd(OAc)_2_/TDMPP, 1.0 M in PhMe at 23 °C for 14 or 24 h. (ii) 5 mol% Cu(OAc)_2_·H_2_O/**17**, (EtO)_2_MeSiH (2 equiv.), 0.20 M in PhMe at 23 °C for 0.25–2 h, followed by quenching with TBAF (1.0 M in THF, 3.5 equiv.). Yields are of isolated products after chromatography. Enantiomeric excesses were determined by chiral HPLC analysis.

^*b*^The coupling was performed using a 2.0 : 1.0 ratio of **6i** : **7l**.

^*c*^The reduction was conducted at 0 °C, and the silyl enol ether was quenched using 1% HCl in MeOH instead of TBAF.

### Synthesis of chiral β-alkynyl sulfones

Alkyne–alkyne couplings employing acetylenic sulfones as acceptors were particularly successful. Reactions between terminal alkynes **6a**, **6b**, and **6i** and sulfone **7o** proceeded in high yields, setting the stage for the conversion of the resulting enynyl sulfones to synthetically valuable β-alkynyl sulfones *via* conjugate reduction (Table 8). This process was initially investigated using the conditions developed for ynenoate reduction: namely, the use of catalytic Cu(OAc)_2_·H_2_O, ligand **17**, and diethoxymethylsilane in toluene with added *tert*-butanol. Although the desired β-alkynyl sulfones were obtained with complete regio- and stereoselectivity, reaction conversion was limited (<20% conversion after 24 h at room temperature). A report from Charette and Desrosiers22*g* describing the asymmetric conjugate reduction of α,β-unsaturated sulfones in mixed organic/aqueous media encouraged us to examine water as a reaction additive. We soon discovered that reactions performed using water in place of the *tert*-butanol additive proceeded to high conversion. When conducted at 50 °C, these transformations consistently proceeded to completion within 6 h. The corresponding alkyl-, aryl-, and silyl-substituted β-alkynyl sulfones were isolated in high yields. Excellent enantioselectivity (98–99% ee) was still uniformly observed, despite this introduction of water and this increase in reaction temperature.

**Table 8:** Synthesis of β-alkynyl sulfones *via* sequential catalysis^*a*^

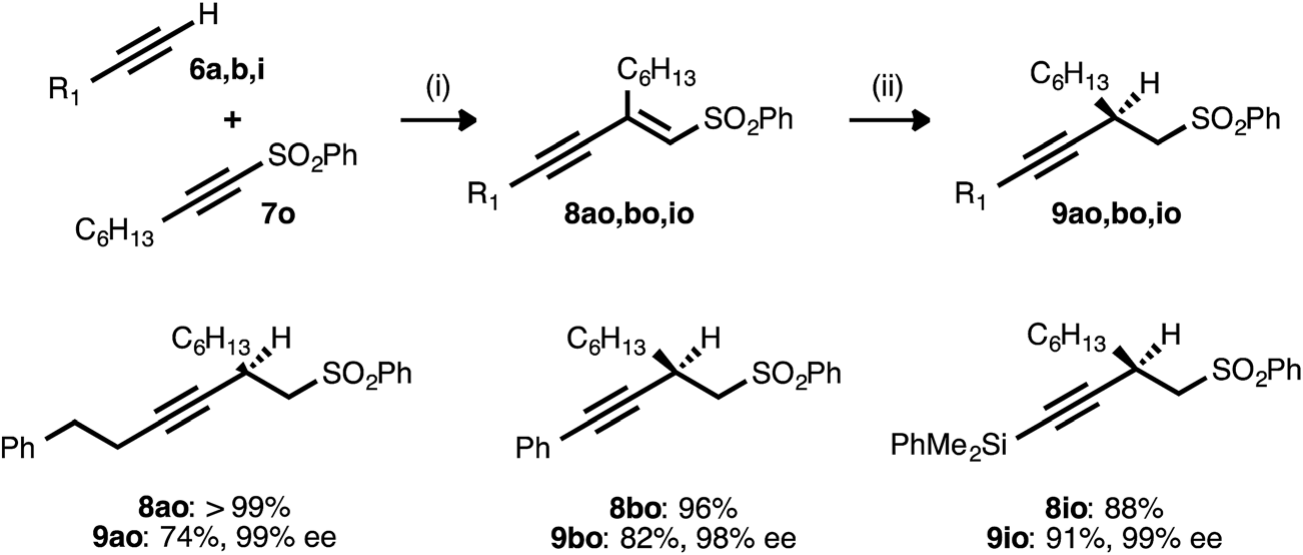

^*a*^Reaction conditions: (i) **6** : **7o** ratio = 1.25 : 1.0, 3 mol% Pd(OAc)_2_/TDMPP, 1.0 M in PhMe at 23 °C for 17–22 h. (ii) 5 mol% Cu(OAc)_2_·H_2_O/**17**, (EtO)_2_MeSiH (2.0 equiv.), H_2_O (5.0 equiv.), 0.20 M in PhMe at 50 °C for 2.5–6 h. Yields are of isolated products after chromatography. Enantiomeric excesses were determined by chiral HPLC analysis.

### One-pot β-alkynyl ester synthesis

**Scheme 7:**
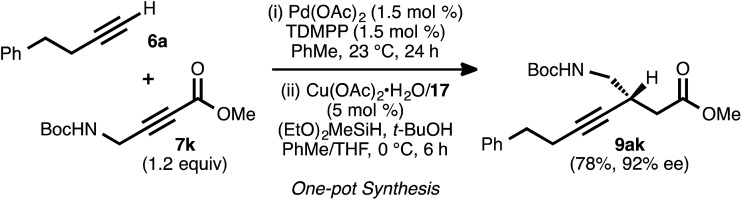
One-pot sequential alkyne–alkyne coupling/conjugate reduction.

To further assess the robustness and operationally simplicity of the method, a one-pot, sequential alkyne–alkyne coupling/conjugate reduction process was investigated (Scheme 7). In this event, the coupling was carried out using donor **6a** and a slight excess of acceptor **7k**. Following complete consumption of the donor alkyne, the reaction vessel was cooled to 0 °C. A pre-formed solution of the copper catalyst was introduced, and then the silane and *tert*-butanol were added. Under these conditions, asymmetric conjugate reduction occurred smoothly, and ester **9ak** was obtained in 78% isolated yield and 92% ee.

### Synthetic utility

**Scheme 8:**
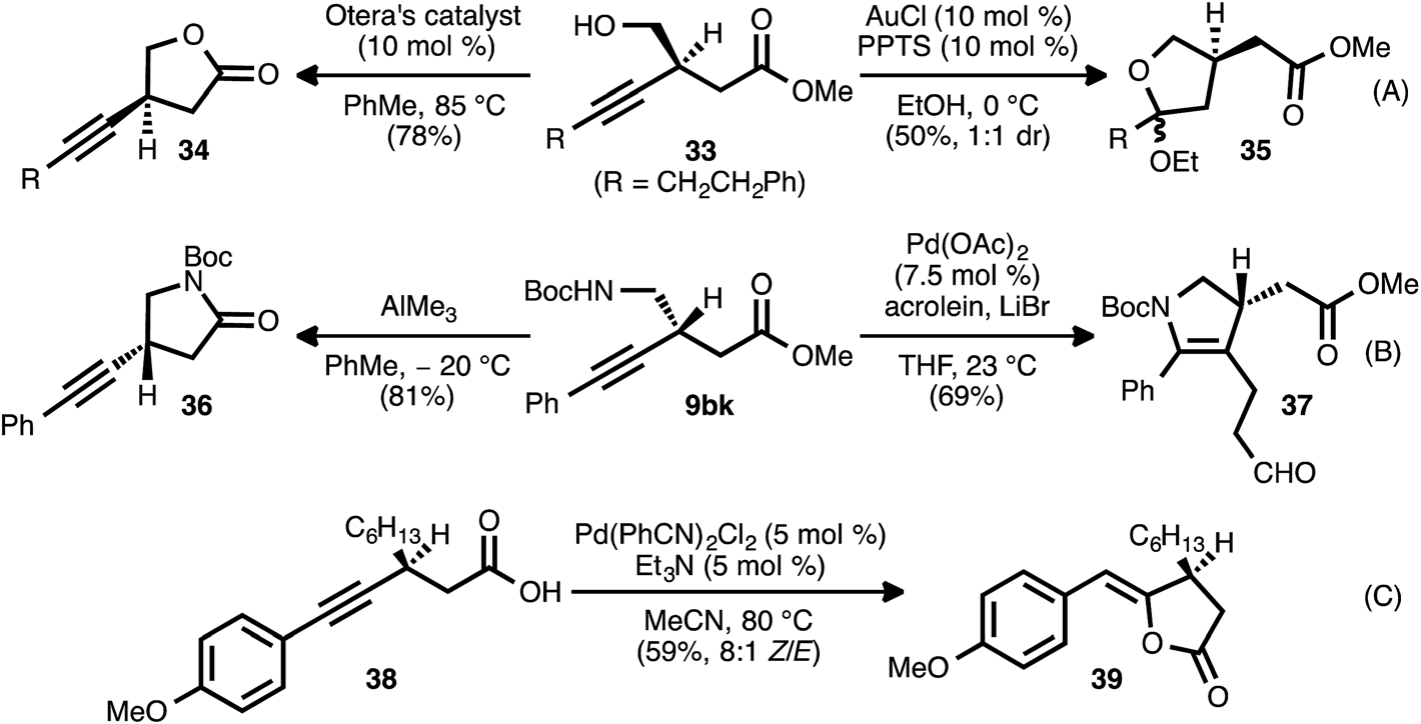
Chemoselective elaboration of β-alkynyl esters.

The β-alkynyl compounds so produced were readily converted into a range of other chiral small molecules through chemoselective transformations. Carbonate-bearing compound **9aj** was hydrolyzed to furnish alcohol **33** (Scheme 8, eqn (A)). Treatment of the latter compound with Otera's esterification catalyst^34^ effected cyclization of the oxygen atom onto the ester group, furnishing chiral lactone **34** in 78% yield. Alternatively, cyclization onto the alkyne group was possible using gold and Brønsted acid co-catalysis, leading to chiral tetrahydrofuran **35**(ref. 35). Nitrogen-bearing substrate **9bk** was similarly converted to pyrrolidinone **36***via* trimethylaluminum-mediated lactamization (eqn (B)). Upon exposure to Pd(OAc)_2_, LiBr, and acrolein, compound **9bk** underwent a tandem 5-*endo*-dig cyclization/reductive Heck-type addition, generating chiral dihydropyrrole **37**.^36^ It was also possible to engage both the carboxylate group and the alkyne moiety of a substrate in a single bond-forming event: when β-alkynyl ester **9ca** was hydrolyzed to acid **38** and the latter compound was treated with catalytic Pd(PhCN)_2_Cl_2_ and Et_3_N in refluxing MeCN, cycloisomerization occurred to generate chiral lactone **39** (eqn (C)).^37^

**Scheme 9:**
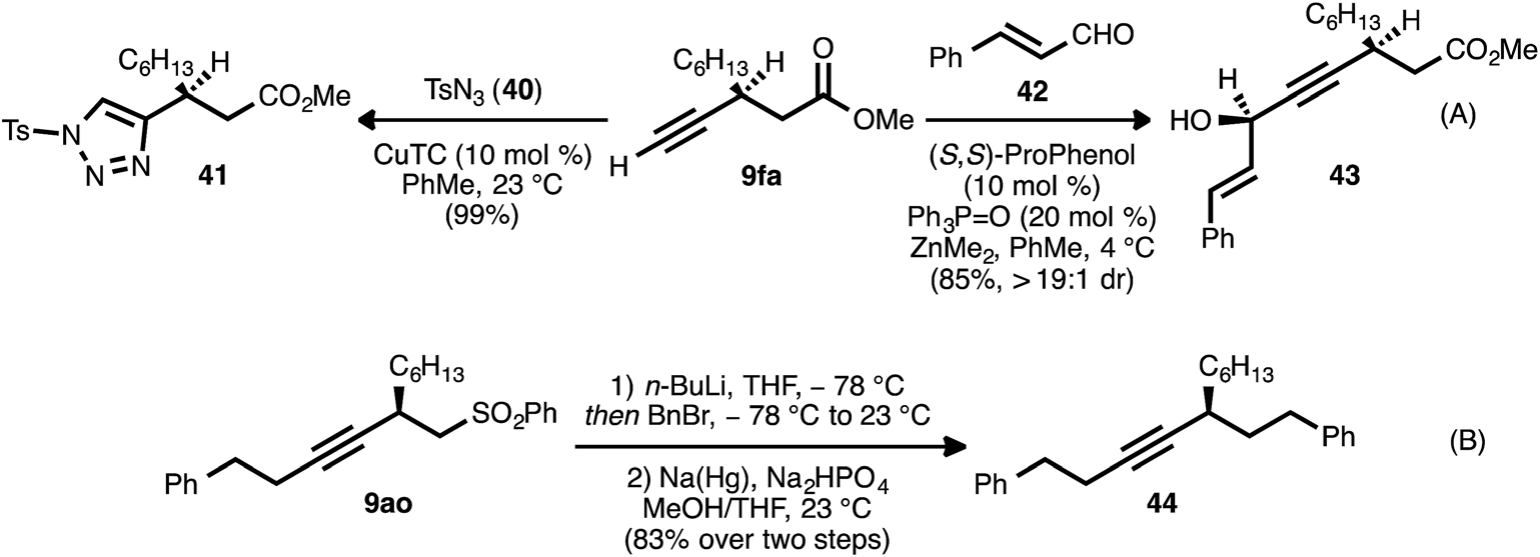
Addition reactions of β-alkynyl carbonyl and sulfonyl derivatives.

In addition to these cycloisomerization processes, addition reactions involving β-alkynyl carbonyl and sulfonyl derivatives were also possible (Scheme 9). In the presence of a catalytic amount of copper thiophene-2-carboxylate (CuTC), the terminal alkyne group of ester **9fa** underwent cycloaddition with tosyl azide (**40**) to deliver 1,2,3-triazole **41** in quantitative yield (eqn (A)).^38^ Fokin and co-workers have described such tosyl-substituted triazoles as efficient precursors to azavinyl carbenes,^39^ and this high-yielding, catalytic asymmetric synthesis of **41** provides an efficient entry into a new series of these compounds. The asymmetric addition of the terminal alkyne group of **9fa** to *trans*-cinnamaldehyde (**42**) under Zn-ProPhenol catalysis^40^ likewise proceeded smoothly and with complete diastereocontrol, delivering tertiary alcohol **43** in high yield (85%).

In a further display of chemoselectivity, β-alkynyl sulfone **9ao** was engaged in an alkylation/reductive desulfonylation sequence (eqn (B)). Following deprotonation of this substrate with *n*-butyllithium, alkylation with benzyl bromide occurred cleanly. Treatment of the crude product with sodium–mercury amalgam in buffered MeOH/THF22*g* delivered chiral hydrocarbon **44** in high yield (83% for two steps). Notably, competitive reduction of the alkyne moiety was not observed during this process.

### Determination of the absolute stereochemistry

**Scheme 10:**
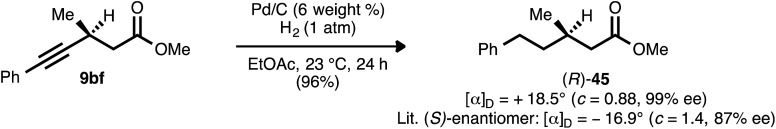
Catalytic hydrogenation of β-alkynyl ester **9bf**.

β-Alkynyl ester **9bf**, which was prepared from ynenoate **8bf***via* conjugate reduction using WALPHOS ligand **17**, was hydrogenated to aliphatic compound **45** (Scheme 10). The optical rotation exhibited by this product was of comparable magnitude but opposite sign to that reported by Feringa and co-workers for the known (*S*)-enantiomer.^41^ The product is therefore assigned as the (*R*)-enantiomer, and the stereochemical assignments of the remaining β-alkynyl esters, ketones, and sulfones prepared using ligand **17** are assigned by analogy.

Asymmetric conjugate reductions performed using JOSIPHOS ligand **13** have been reported to proceed with the opposite sense of absolute stereoinduction relative to those performed using WALPHOS ligand **17**.22f,j As a result, the β-alkynyl esters prepared using ligand **13** (*e.g.*, compounds **9ak**, **9bk**, and **9ek**) are assigned as depicted: enantiomeric to the set of products obtained using ligand **17**. Entirely consistent with these reports, the conjugate reduction of ynenoate **28b** using WALPHOS ligand *ent*-**17** furnished the same major enantiomer of product as did the reduction of **28b** using either JOSIPHOS ligand **13** or JOSIPHOS ligand **14** (*Cf.*Scheme 5). The preceding data suggest that the product, β-alkynyl ester **29b**, would bear (*S*)-stereochemistry. This assignment was verified upon completion of the synthesis of AMG 837 (**24**) from **29b**: the optical rotation data obtained for the latter were in agreement with prior reports (observed [*α*]25D = +4.4° (*c* = 0.9, CHCl_3_); lit. [(*S*)-enantiomer]: +7.2° (*c* = 0.5, CHCl_3_)31*b*).

## Conclusions

We present a full account describing a sequential catalysis strategy for the asymmetric synthesis of chiral β-alkynyl carbonyl and sulfonyl derivatives from simple alkyne precursors. A Pd-catalyzed alkyne–alkyne cross coupling generates, with complete regio- and stereoselectivity, enyne intermediates that are reduced under CuH catalysis. A detailed assessment of ligand effects in the latter stage led to the identification of WALPHOS ligand **17** and JOSIPHOS ligands **13** and **14** as highly effective for the regio- and enantioselective reduction of a broad range of activated enynes.

Relative to our initial report in this area, the substrate scope has been significantly expanded. Fourteen new, sterically and electronically diverse chiral β-alkynyl esters have been prepared. Additionally, the scope of the sequential catalysis approach has been expanded to encompass ynones and acetylenic sulfones as acceptor alkynes. To this end, new and previously unreported syntheses of chiral β-alkynyl ketones and sulfones are described.

The transformations described herein demonstrated high levels of robustness. Excellent results (>90% yield and 99% ee) were obtained when the reactions were executed on gram scale. A one-pot β-alkynyl ester synthesis was also developed, one that furnished the target in high yield without sacrificing selectivity.

In contrast to other methods for the synthesis of these targets, such as the direct addition of an alkyne to an activated olefin, the process described herein displays a significant breadth of scope. Alkyl, silyl, and aryl-substituted alkynes were all engaged as donors, and ester, ketone, and sulfone-derived acceptors all proved compatible. This ability to directly employ ester-based acceptors is especially noteworthy, given the paucity of methods for the catalytic asymmetric alkynylation of acrylates.

The method tolerates oxygen and nitrogen substitution, and this enabled the preparation of six different heterocyclic scaffolds from the corresponding β-alkynyl compounds. In addition, a new Pd-catalyzed allylic alkylation reaction was developed to enable the convergent introduction of heteroatom functionality to the ynenoate intermediate. This expanded the scope of the method even further, as the ynenoates so prepared responded well to the conjugate reduction conditions. Upon further reaction optimization, the substrate scope was expanded to include β-aryl ynenoates. This facilitated a concise synthesis of AMG 837, a chiral β-alkynyl carboxylic acid of pharmaceutical interest.

In a further demonstration of the synthetic utility of the process, the β-alkynyl products were engaged in efficient addition reactions. These included a catalytic, stereocontrolled addition of a terminal alkyne to an aldehyde. The versatility of the sulfone substituent in the product *via* an alkylation–desulfonylation sequence involving the β-alkynyl sulfone provides tertiary propargylic all carbon stereocenters. Further, given the flexibility of the alkyne beyond semi-hydrogenation both to *Z*- and *E*-olefins as well as full saturation truly becomes enabling in creating structural diversity. The ability to access such diverse products from simple alkyne precursors highlights the utility of the method in complex molecule synthesis.

## Supplementary Material

Supplementary information
