# Supplementary material for: Asymmetric synthesis of chiral β-alkynyl carbonyl and sulfonyl derivatives via sequential palladium and copper catalysis
Source: Chem Sci. 2016 Jun 10;7(9):6217–31. doi: 10.1039/c6sc01724j (PMC5044515; doi:10.1039/c6sc01724j)

Electronic Supplementary Material (ESI) for Chemical Science.
This journal is © The Royal Society of Chemistry 2016

Asymmetric Synthesis of Chiral β-Alkynyl Carbonyl and Sulfonyl Derivatives *via* Sequential Palladium and Copper Catalysis

Barry M. Trost*, James T. Masters, Benjamin R. Taft, and Jean-Philip Lumb
Department of Chemistry, Stanford University, Stanford, CA 94305-4401

Supporting Information

Table of Contents

General Considerations……………………………………………………………….S2

Experimental Procedures……………………………………………………………..S3

^{1}H and ^{13}C NMR Spectra…………………………………...……………………..S75

General Considerations: Unless otherwise noted, all alkyne-alkyne coupling reactions were performed in 2-dram vials equipped with Teflon coated stir bars. Importantly, care was not taken to exclude moisture or oxygen from the reaction mixture. Following the addition of solids and liquids to the reaction vessel, the vial was simply capped, and stirring was continued for the specified amount of time. Commercially available terminal alkynes were distilled prior to use in these reactions. Unless otherwise noted, all conjugate reduction reactions were performed in 5 mL conical microwave vials equipped with Teflon-coated stir bars under an atmosphere of argon (balloon). PhMe was freshly distilled over CaH_2, and THF was freshly distilled over Na/benzophenone. CH_2Cl_2 was obtained from a Seca solvent purification system by Glass Contour. Solvents and reagents were transferred *via* syringes using stainless steel needles that were dried in an oven and cooled in a desiccator.

Analytical thin-layer chromatography was performed on pre-coated 250 μm layer thickness silica gel 60 F_{254} plates (EMD Chemicals Inc.). Visualization was performed by ultraviolet light and/or by staining with aqueous potassium permanganate or acidic, ethanolic *para*-anisaldehyde. Unless otherwise indicated, flash column chromatography was performed using 40-63 μm silica gel (Silicycle silica gel) using compressed air. The eluent employed for flash chromatography is reported using volume/volume ratios. Proton nuclear magnetic resonance (^{1}H NMR) spectra were acquired using a Varian Inova 500 MHz, Varian Inova 300 MHz, or Varian Mercury 400 MHz spectrometer. Chemical shifts (δ) are reported in parts per million (ppm) and are calibrated to the residual solvent peak: proton ($CDCl_3$ 7.26 ppm, C_6D_6 7.16 ppm). Coupling constants (*J*) are reported in Hz. Multiplicities are reported using the following abbreviations: s = singlet; d = doublet; t = triplet; q = quartet; sex = sextet; m = multiplet (range of multiplet is given). Carbon nuclear magnetic resonance (^{13}C NMR) spectra were recorded using a Varian Inova spectrometer at 125 MHz, Varian Inova spectrometer at 75 MHz, or a Varian Mercury spectrometer at 100 MHz. Chemical shifts (δ) are reported in parts per million (ppm) and are calibrated to solvent peaks: carbon ($CDCl_3$ 77.16 ppm, C_6D_6 128.06 ppm). Infrared spectroscopic data was recorded on a Thermo Scientific Nicolet IR100 FT-IR spectrometer, using thin films of the sample on NaCl plates. The absorbance frequencies are recorded in wavenumbers (cm^{-1}).

Chiral HPLC analysis was performed using an Agilent Technologies 1200 Series HPLC equipped with a Daicel Chemical Chiralpak® chiral stationary phase column (either IA [amylose tris(3,5-dimethylphenylcarbamate) immobilized on silica support], IB [cellulose tris(3,5-dimethylphenylcarbamate) immobilized on silica support], or IC [cellulose tris(3,5-dichlorophenylcarbamate) immobilized on silica support]). Optical rotations were measured using a JASCO P2000 polarimeter with 5 cm glass cells and a sodium 589 nm filter, and they are reported as $[\alpha]_D^T$, concentration (g/100 mL), and solvent. Melting points were determined on a Thomas Hoover Capillary Melting Point Apparatus and are uncorrected. High-resolution mass spectra were acquired by the Vincent Coates Foundation Mass Spectrometry Laboratory, Stanford University Mass Spectrometry (http://massspec.stanford.edu).

Experimental Procedures

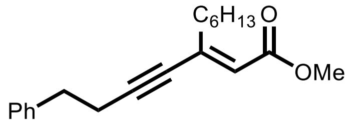

Pd-catalyzed Alkyne-Alkyne Coupling with Ester Acceptors: Representative Procedure A, Synthesis of methyl *(E)*-3-(4-phenylbut-1-yn-1-yl)non-2-enoate (**8aa**):

A 2-dram screw-cap vial was charged with $Pd(OAc)_2$ (6.7 mg, 0.03 mmol, 3 mol %) and tris(2,6-dimethoxyphenyl)phosphine (13.3 mg, 0.03 mmol, 3 mol %). PhMe (1.0 mL) was added. The mixture was allowed to stir for 15 minutes, generating a homogeneous, orange-red solution. Methyl 2-nonynoate (184 µL, 1.0 mmol, 1.0 equivalent) was added followed immediately by 4-phenyl-1-butyne (175 µL, 1.25 mmol, 1.25 equivalents). Upon addition of the donor alkyne, the reaction quickly changed in appearance to homogeneous and black. The reaction mixture was stirred for 18 h, at which point it was filtered through a pipette plug of Florisil® (3 cm), which was rinsed with Et_2O (10 mL). The solution was concentrated, and the residue was purified *via* column chromatography (20:1 hexanes:Et_2O) to afford the title compound as a brown oil (287.4 mg, 96%). $\mathbf{R_f}$ = 0.38 (10:1 hexanes:EtOAc) **^{1}H NMR** (400 MHz, $CDCl_3$) δ 7.33-7.29 (m, 2H), 7.25-7.22 (m, 3H), 5.96 (s, 1H), 3.70 (s, 3H), 2.87 (t, *J* =

7.4 Hz, 2H), 2.72-2.64 (m, 4H), 1.54-1.49 (m, 2H), 1.33-1.28 (m, 6H,) 0.90 (t, *J* = 6.9 Hz, 3H) **^{13}C NMR** (100 MHz, $CDCl_3$) δ 166.6, 144.5, 140.5, 128.7, 128.6, 126.6, 122.8, 95.5, 82.9, 51.3, 35.0, 32.8, 31.9, 29.2, 28.6, 22.9, 22.0, 14.4 **IR** (film): 3063, 3028, 2929, 2858, 2218, 1717, 1611, 1496, 1452, 1433, 1347, 1270, 1243, 1191, 1158, 1077, 1032, 871, 748, 698 cm^{-1}. **HRMS**: Calculated for $C_{20}H_{27}O_2$ $(M+H)^+$: 299.2006; Found 299.2004.

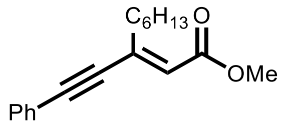

methyl *(E)*-3-(phenylethynyl)non-2-enoate (**8ba**)

Prepared using Representative Procedure A using $Pd(OAc)_2$ (6.7 mg, 0.03 mmol, 3 mol %), tris(2,6-dimethoxyphenyl)phosphine (13.3 mg, 0.03 mmol, 3 mol %), methyl 2-nonynoate (184 μL, 1.0 mmol, 1.0 equivalent), and phenylacetylene (137 μL, 1.25 mmol) in PhMe (1.0 mL). After 3 h 30 min, additional phenylacetylene (55 μL, 0.50 mmol) was added, and stirring was continued for an additional 2 h, at which point the reaction was worked up as described in the representative procedure. Purification *via* column chromatography (25:1 hexanes:EtOAc) afforded the title compound as a brown oil (228 mg, 84% yield). $\mathbf{R}_f$ = 0.17 (25:1 hexanes:Et_2O). **^{1}H NMR** (300 MHz, $CDCl_3$) δ 7.49-7.46 (m, 2H), 7.36-7.34 (m, 3H), 6.15 (t, *J* = 0.9 Hz, 1H), 3.73 (s, 3H), 2.88 (td, *J* = 7.7 Hz, 0.9 Hz, 2H), 1.69-1.63 (m, 2H), 1.45-1.29 (m, 6H), 0.89 (m, 3H). **^{13}C NMR** (75 MHz, $CDCl_3$) δ 166.4, 143.7, 132.0, 129.1, 128.5, 123.4, 122.5, 94.5, 90.4, 51.4, 32.4, 31.8, 29.1, 28.6, 22.7, 14.2. **IR** (film): 2953, 2929, 2858, 2202, 1718, 1609, 1490, 1433, 1353, 1314, 1272, 1199, 1140, 1027, 922, 870, 756, 690 cm^{-1} **HRMS:** Calculated for $C_{18}H_{22}O_2Na$ $(M+Na)^+$: 293.1517; Found 293.1513.

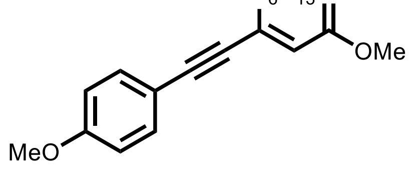

methyl *(E)*-3-((4-methoxyphenyl)ethynyl)non-2-enoate (**8ca**)

Prepared according to Representative Procedure A using $Pd(OAc)_2$ (3.3 mg, 0.015 mmol, 3 mol %) and tris(2,6-dimethoxyphenyl)phosphine (6.7 mg, 0.015 mmol, 3 mol %), methyl 2-nonynoate (92 µL, 0.50 mmol), and 4'-methoxyphenylacetylene (81 µL, 0.625 mmol) in PhMe (0.50 mL). The reaction duration was 1 h. Purification *via* column chromatography (10:1 hexanes:EtOAc) afforded the title compound (143.1 mg, 95%) as a clear, faintly yellow oil. Note: TLC analysis (6:1 hexanes:EtOAc) could be used to monitor for the consumption of acceptor alkyne; however, in this solvent system, the donor alkyne and enynoate product are co-polar. TLC analysis using 6:1 hexanes:Et_2O differentiates both the donor alkyne (R_f = 0.56) and the acceptor (R_f = 0.50) from the product ynenoate (R_f = 0.42). **R_f** = 0.38 (10:1 hexanes:EtOAc). **^{1}H NMR** (400 MHz; $CDCl_3$): δ 7.43-7.39 (m, 2H), 6.88-6.85 (m, 2H), 6.10 (s, 1H), 3.82 (s, 3H), 3.72 (s, 3H), 2.83 (t, *J* = 7.7 Hz, 2H), 1.69-1.61 (m, 2H), 1.43-1.29 (m, 6H), 0.89 (t, *J* = 7.1 Hz, 3H). **^{13}C NMR** (100 MHz; $CDCl_3$): δ 166.5, 160.3, 144.1, 133.6, 122.6, 114.6, 114.2, 95.0, 89.5, 55.5, 51.3, 32.5, 31.8, 29.1, 28.7, 22.7, 14.3. **IR** (film): 2889, 2818, 2167, 1692, 1576, 1489, 1414, 1335, 1273, 1233, 1156, 1123, 1061, 1018, 911, 857, 821, 716 cm-1 **HRMS** (ESI+): Calculated for $C_{19}H_{25}O_3$ $(M+H)^+$: 301.1798; Found 301.1792.

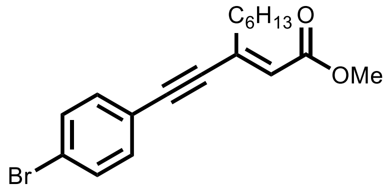

methyl *(E)*-3-((4-bromophenyl)ethynyl)non-2-enoate (**8da**)

Prepared according to Representative Procedure A using $Pd(OAc)_2$ (3.3 mg, 0.015 mmol, 3 mol %), tris(2,6-dimethoxyphenyl)phosphine (6.7 mg, 0.015 mmol, 3 mol %), methyl 2-nonynoate (92 µL, 0.50 mmol), and 4-bromophenylacetylene[1] (113.2 mg, 0.625 mmol) in PhMe (0.50 mL). After 5 h, additional 4-bromophenylacetylene (45.3 mg, 0.25 mmol) was added, and the reaction mixture was stirred for an additional 12 h. Purification *via*

column chromatography (20:1 hexanes:Et_2O) afforded the title compound (137.0 mg, 78%) as a clear, red-brown oil. **R*f*** = 0.55 (10:1 hexanes:EtOAc). **^{1}H NMR** (300 MHz; $CDCl_3$): δ 7.50-7.46 (m, 2H), 7.34-7.30 (m, 2H), 6.14 (t, *J* = 0.9 Hz, 1H), 3.73 (s, 3H), 2.85-2.80 (m, 2H), 1.68-1.58 (m, 2H), 1.43-1.27 (m, 6H), 0.89 (d, *J* = 14.1 Hz, 3H). **^{13}C NMR** (100 MHz; $CDCl_3$): 166.3, 143.3, 133.4, 131.9, 123.9, 123.5, 121.5, 93.2, 91.4, 51.4, 32.3, 31.8, 29.1, 28.6, 22.7, 14.2 **IR** (film): 2887, 2818, 1694, 1586, 1561, 1465, 1413, 1334, 1155, 1124, 1054, 997, 859, 812 cm-1 **HRMS** (ESI+): Calculated for $C_{18}H_{22}O_2Br$ $(M+H)^+$: 349.0798; Found 349.0789.

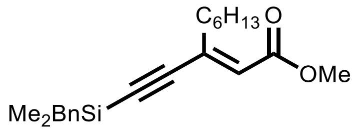

methyl *(E)*-3-((benzyldimethylsilyl)ethynyl)non-2-enoate (**8ea**)

Prepared according to Representative Procedure A using $Pd(OAc)_2$ (6.7 mg, 0.03 mmol, 3 mol %), tris(2,6-dimethoxyphenyl)phosphine (13.3 mg, 0.03 mmol, 3 mol %), methyl 2-nonynoate (184 μL, 1.0 mmol, 1.0 equivalent), and benzyldimethylsilylacetylene (218 mg, 1.25 mmol) in PhMe (1.0 mL). The reaction duration was 6 h 30 min. Purification *via* column chromatography (50:1 hexanes:Et_2O) afforded the title compound (313 mg, 92%) as a brown oil. **R*f*** = 0.45 (10:1 hexanes:EtOAc). **^{1}H NMR** (400 MHz, $CDCl_3$) δ 7.26-7.21 (m, 2H), 7.12-7.06 (m, 3H), 6.06 (t, *J* = 1.0 Hz, 1H), 3.71 (s, 3H), 2.71 (td, *J* = 7.7 Hz, 0.8 Hz, 2H), 2.23 (s, 2H), 1.58-1.54 (m, 2H), 1.31-1.29 (m, 6H), 0.89 (t, *J* = 7.0 Hz, 3H), 0.17, s, 6H. **^{13}C NMR** (125 MHz, $CDCl_3$) δ 166.3, 143.1, 138.7, 128.5, 128.3, 124.6, 124.5, 106.9, 98.6, 51.4, 32.1, 31.8, 29.0, 28.5, 26.1, 22.7, 14.2, -2.1. **IR** (film): 3025, 2956, 2929, 2859, 2144, 1720, 1610, 1493, 1453, 1434, 1352, 1251, 1149, 1057, 1026, 926, 841, 762, 698. **HRMS**: Calculated for $C_{21}H_{31}O_2Si$ $(M+H)^+$: 343.2088; Found 343.2095.

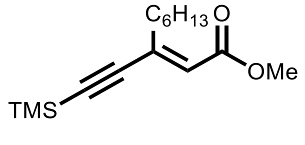

methyl *(E)*-3-((trimethylsilyl)ethynyl)non-2-enoate (**8fa**)

Prepared according to Representative Procedure A using Pd(OAc)$_2$ (20.2 mg, 0.09 mmol, 3 mol %), tris(2,6-dimethoxyphenyl)phosphine (39.8 mg, 0.09 mmol, 3 mol %), methyl 2-nonynoate (550 μL, 3.0 mmol) and trimethylsilylacetylene (530 μL, 3.75 mmol, 1.25 equivalent) in PhMe (3 mL). The reaction duration was 2 h. Purification *via* column chromatography (30:1 to 20:1 hexanes:EtOAc) afforded the title compound (731.9 mg, 92%) as a clear, brown oil. **R$_f$** = 0.57 (10:1 hexanes:EtOAc). **^{1}H NMR** (400 MHz; CDCl$_3$): δ 6.06 (s, 1H), 3.69 (s, 3H), 2.71 (t, J = 7.6 Hz, 2H), 1.60-1.52 (m, 2H), 1.36-1.27 (m, 6H), 0.88 (t, J = 6.8 Hz, 3H), 0.20 (s, 9H). **^{13}C NMR** (100 MHz; CDCl$_3$): δ 166.4, 143.4, 124.3, 105.6, 100.3, 51.4, 32.1, 31.7, 29.0, 28.4, 22.7, 14.2, -0.1. **IR** (film): 2916, 2819, 2115, 1698, 1588, 1414, 1334, 1234, 1132, 1075, 1011, 914, 833, 750, 691, 693 cm-1. **HRMS** (ESI+): Calculated for $C_{15}H_{26}NaO_2Si$ $(M+Na)^+$: 289.1594; Found 289.1599.

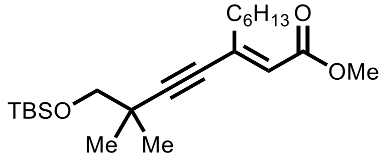

methyl *(E)*-3-(4-((*tert*-butyldimethylsilyl)oxy)-3,3-dimethylbut-1-yn-1-yl)non-2-enoate (**8ga**)

Prepared according to Representative Procedure A using Pd(OAc)$_2$ (3.4 mg, 0.015 mmol, 3 mol %), tris-2,6-dimethoxyphenylphosphine (TDMPP, 6.3 mg, 0.015 mmol, 3 mol %), methyl 2-nonynoate, (92 μL, 0.50 mmol, 1.0 equivalent) and known donor alkyne *tert*-butyl((2,2-dimethylbut-3-yn-1-yl)oxy)dimethylsilane[2] (133 mg, 0.625 mmol) in PhMe (0.50 mL). The reaction duration was 24 h. Purification *via* column chromatography (20:1 hexanes:EtOAc) afforded the title compound (180.4 mg, 95%) as a viscous, orange oil. **R$_f$** = 0.58 (10:1 hexanes:EtOAc). **^{1}H NMR** (500 MHz; CDCl$_3$): δ 5.96 (t, J = 1.0 Hz, 1H), 3.69 (s, 3H), 3.45 (s, 2H), 2.72-2.69 (m, 2H), 1.57-1.53 (m, 2H), 1.34-1.28 (m, 6H), 1.20 (s, 6H), 0.90-0.88 (m, 12H), 0.05 (s, 6H). **^{13}C NMR** (100 MHz; CDCl$_3$): δ 166.7, 144.6, 122.5, 102.3, 81.9, 71.1, 51.2, 34.5, 32.6, 31.8, 29.0, 28.4, 26.0, 25.4, 22.7, 18.4, 14.2, -5.3. **IR** (film):

2889, 2818, 1696, 1589, 1449, 1341, 1237, 1132, 1093, 827, 766 cm^{-1}. **HRMS** (ESI+): Calculated for $C_{22}H_{41}O_3Si$ $(M+H)^+$: 381.2819; Found 381.2816.

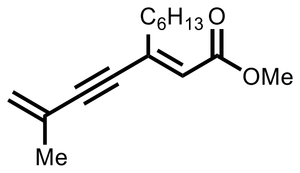

methyl *(E)*-3-(3-methylbut-3-en-1-yn-1-yl)non-2-enoate (**8ha**)

Prepared according to Representative Procedure A using $Pd(OAc)_2$ (3.4 mg, 0.015 mmol, 3 mol %), tris-2,6-dimethoxyphenylphosphine (TDMPP, 6.3 mg, 0.015 mmol, 3 mol %), methyl 2-nonynoate, (92 µL, 0.50 mmol, 1.0 equivalent) and 2-methyl-1-buten-3-yne (60 µL, 0.625 mmol) in PhMe (0.50 mL). In light of the volatility of this donor alkyne (b. p. = 32 °C), the vial threads were wrapped with PTFE tape, and the capped vial was sealed with Parafilm®. The reaction duration was 18 h. Purification *via* column chromatography (20:1 hexanes:EtOAc) afforded the title compound (94.4 mg, 81%) as a clear, brown oil. $\mathbf{R}_f$ = 0.53 (10:1 hexanes:EtOAc). **^{1}H NMR** (500 MHz; $CDCl_3$): δ 6.04 (s, 1H), 5.37 (s, 1H), 5.32 (s, 1H), 3.70 (s, 3H), 2.75 (t, J = 7.6 Hz, 2H), 1.92 (s, 3H), 1.60-1.54 (m, 2H), 1.36-1.30 (m, 6H), 0.88 (t, J = 6.1 Hz, 3H). **^{13}C NMR** (100 MHz; $CDCl_3$): δ 166.4, 143.7, 126.5, 123.7, 123.4, 95.7, 89.4, 51.3, 32.4, 31.8, 29.1, 28.6, 23.3, 22.7, 14.2. **IR** (film): 2888, 2819, 2166, 1696, 1591, 1414, 1333, 1300, 1134, 1015, 887, 859, 715 cm^{-1}. **HRMS** (ESI+): Calculated for $C_{15}H_{23}O_2$ $(M+H)^+$: 235.1693; Found 235.1687.

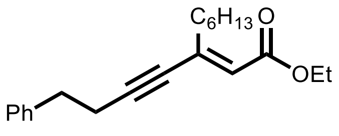

ethyl *(E)*-3-(4-phenylbut-1-yn-1-yl)non-2-enoate (**8ab**)

Prepared according to Representative Procedure A using $Pd(OAc)_2$ (3.4 mg, 0.015 mmol, 3 mol %), tris-2,6-dimethoxyphenylphosphine (TDMPP, 6.3 mg, 0.015 mmol, 3 mol %), 4-phenyl-1-butyne (88 μL, 0.625 mmol) and known acceptor ethyl 2-hexynoate[3] (91.1 mg, 0.50 mmol) in PhMe (0.50 mL). The reaction duration was 4 h. Purification *via* column chromatography (20:1 hexanes:Et_2O) afforded the title compound (145.9 mg, 93%) as an oil. **R_f** = 0.53 (10:1 hexanes:EtOAc). **^{1}H NMR** (400 MHz; $CDCl_3$): δ 7.33-7.28 (m, 2H), 7.24-7.22 (m, 3H), 5.95 (s, 1H), 4.15 (q, *J* = 7.1 Hz, 2H), 2.86 (t, *J* = 7.4 Hz, 2H), 2.70-2.63 (m, 4H), 1.52-1.48 (m, 2H), 1.33-1.25 (m, 9H), 0.89 (t, *J* = 6.9 Hz, 3H). **^{13}C NMR** (100 MHz; $CDCl_3$): δ 166.2, 144.0, 140.5, 128.6, 128.5, 126.5, 123.2, 95.2, 82.8, 60.0, 35.0, 32.7, 31.8, 29.1, 28.6, 22.8, 21.9, 14.4, 14.3. **IR** (film): 2914, 2888, 2818, 2188, 1690, 1588, 1475, 1434, 1349, 1324, 1282, 1251, 1226, 1138, 1080, 1027, 860, 737, 688 cm^{-1}. **HRMS** (ESI+): Calculated for $C_{21}H_{29}O_2$ $(M+H)^+$: 313.2162; Found 313.2158.

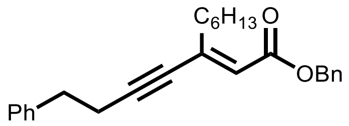

benzyl *(E)*-3-(4-phenylbut-1-yn-1-yl)non-2-enoate (**8ac**)

Prepared according to Representative Procedure A using $Pd(OAc)_2$ (3.4 mg, 0.015 mmol, 3 mol %), tris-2,6-dimethoxyphenylphosphine (TDMPP, 6.3 mg, 0.015 mmol, 3 mol %), 4-phenyl-1-butyne (88 μL, 0.625 mmol) and known acceptor benzyl 2-nonynoate[4] (122.2 mg, 0.50 mmol) in PhMe (0.50 mL). The reaction duration was 3 h. Purification *via* column chromatography (30:1 hexanes:Et_2O) afforded the title compound (176.8 mg, 94%) as a clear, light orange oil. **R_f** = 0.39 (20:1 hexanes:Et_2O). **^{1}H NMR** (400 MHz; $CDCl_3$): δ 7.37-7.29 (m, 7H), 7.25-7.22 (m, 3H), 6.02 (s, 1H), 5.15 (s, 2H), 2.86 (t, *J* = 7.4 Hz, 2H), 2.70 (t, *J* = 7.7 Hz, 2H), 2.66 (t, *J* = 7.4 Hz, 2H), 1.56-1.47 (m, 2H), 1.33-1.22 (m, 6H), 0.89 (t, *J* = 6.8 Hz, 3H). **^{13}C NMR** (100 MHz; $CDCl_3$): 165.9, 144.8, 140.4, 136.3, 128.7, 128.6, 128.5, 128.2 (2C), 126.5, 122.8, 95.7, 82.8, 65.9, 34.9, 32.8, 31.8, 29.1, 28.6, 22.8, 21.9, 14.3. **IR** (film): 2887, 2818, 2186, 1690, 1586, 1476, 1357, 1249, 1130, 1013, 859, 735, 687 cm-1. **HRMS** (ESI+): Calculated for $C_{26}H_{31}O_2$ $(M+H)^+$: 375.2319; Found 375.2313.

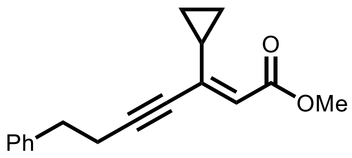

methyl *(E)*-3-cyclopropyl-7-phenylhept-2-en-4-ynoate (**8ad**)

Prepared according to Representative Procedure A using $Pd(OAc)_2$ (3.4 mg, 0.015 mmol, 3 mol %), tris-2,6-dimethoxyphenylphosphine (TDMPP, 6.3 mg, 0.015 mmol, 3 mol %), 4-phenyl-1-butyne (88 μL, 0.625 mmol) and known acceptor methyl 3-cyclopropylpropiolate[5] (62.0 mg, 0.50 mmol) in PhMe (0.50 mL). The reaction duration was 14 h. Purification *via* column chromatography (10:1 hexanes:EtOAc) afforded the title compound (122.5 mg, 96%) as a clear, light brown oil. $\mathbf{R_f}$ = 0.41 (10:1 hexanes:EtOAc). **^{1}H NMR** (400 MHz; $CDCl_3$): δ 7.33-7.29 (m, 2H), 7.25-7.19 (m, 3H), 5.99 (s, 1H), 3.71 (s, 3H), 3.17-3.10 (m, 1H), 2.83 (t, *J* = 7.3 Hz, 2H), 2.61 (t, *J* = 7.4 Hz, 2H), 0.85-0.79 (m, 4H). **^{13}C NMR** (100 MHz; $CDCl_3$): 167.3, 147.5, 140.2, 128.6, 128.5, 126.6, 121.5, 95.0, 78.1, 51.2, 34.8, 21.6, 12.7, 8.6. **IR** (film): 3043, 2985, 2907, 2192, 1688, 1574, 1475, 1434, 1413, 1369, 1250, 1175, 1144, 1016, 949, 909, 845 cm^{-1}. **HRMS** (ESI+): Calculated for $C_{17}H_{19}O_2$ $(M+H)^+$: 255.1380; Found 255.1372.

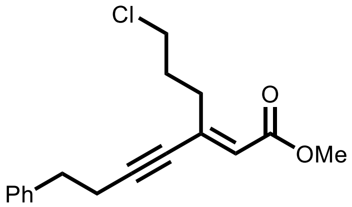

methyl *(E)*-3-(3-chloropropyl)-7-phenylhept-2-en-4-ynoate (**8ae**)

Prepared according to Representative Procedure A using $Pd(OAc)_2$ (3.4 mg, 0.015 mmol, 3 mol %), tris-2,6-dimethoxyphenylphosphine (TDMPP, 6.3 mg, 0.015 mmol, 3 mol %), 4-phenyl-1-butyne (88 μL, 0.625 mmol) and known acceptor[6] methyl 6-chlorohex-2-ynoate (80.3 mg, 0.50 mmol) in PhMe (0.50 mL). The reaction duration was 1 h. Purification *via* column chromatography (20:1 to 10:1 hexanes:EtOAc) afforded the title compound (143.2

mg, 98%) as a clear, light yellow oil. **R*f*** = 0.43 (10:1 hexanes:EtOAc). **^{1}H NMR** (400 MHz; $CDCl_3$): δ 7.34-7.29 (m, 2H), 7.25-7.22 (m, 3H), 6.00 (s, 1H), 3.70 (s, 3H), 3.52 (t, *J* = 6.9 Hz, 2H), 2.89-2.81 (m, 4H), 2.69-2.65 (m, 2H), 2.01-1.94 (m, 2H). **^{13}C NMR** (100 MHz; $CDCl_3$): 166.3, 142.0, 142.3, 128.59, 128.57, 126.6, 123.7, 96.3, 82.2, 51.4, 44.4, 34.8, 31.4, 30.1, 21.8. **IR** (film): 2985, 2908, 2186, 1691, 1590, 1475, 1413, 1325, 1233, 1175, 1146, 1062, 1018, 860 cm^{-1}. **HRMS** (ESI+): Calculated for $C_{17}H_{20}ClO_2$ $(M+H)^+$: 291.1146; Found 291.1143.

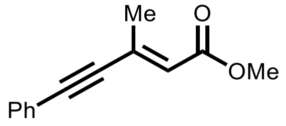

methyl *(E)*-3-methyl-5-phenylpent-2-en-4-ynoate (**8bf**)

Prepared according to Representative Procedure A using $Pd(OAc)_2$ (6.7 mg, 0.03 mmol, 3 mol %), tris(2,6-dimethoxyphenyl)phosphine (13.3 mg, 0.03 mmol, 3 mol %), methyl but-2-ynoate (98 mg, 1.0 mmol), and phenylacetylene (137 µL, 1.25 mmol) in PhMe (1.0 mL). After 3 h 30 min additional phenylacetylene (55 µL, 0.5 mmol) was added. The reaction mixture was stirred for an additional 2 h, at which point it was worked up as described. Purification *via* column chromatography (20:1 hexanes:EtOAc) afforded the title compound as a light brown oil (190 mg, 0.95 mmol, 95% yield). **R*f*** = 0.25 (20:1 hexanes:EtOAc). **^{1}H NMR** (300 MHz, $CDCl_3$) δ 7.49-7.44 (m, 2H), 7.37-7.30 (m, 3H), 6.17 (q, *J* = 1.5 Hz, 1H), 3.74 (s, 3H), 2.40 (d, *J* = 1.5 Hz, 3H). **^{13}C NMR** (75 MHz, $CDCl_3$) δ 166.6, 138.3, 132.0, 129.2, 128.5, 123.7, 122.4, 93.9, 91.2, 51.3, 20.0. **IR** (film): 2949, 2204, 1716, 1614, 1489, 1434, 1375, 1344, 1304, 1273, 1198, 1137, 1070, 1030, 922, 869, 756, 689 cm^{-1}. *These ^{1}H and ^{13}C NMR data matched that previously reported for this compound.*[7]

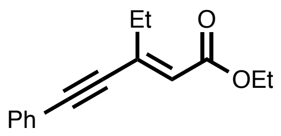

ethyl *(E)*-3-ethyl-5-phenylpent-2-en-4-ynoate (**8bg**)

Prepared according to Representative Procedure A using $Pd(OAc)_2$ (3.4 mg, 0.015 mmol, 3 mol %), tris-2,6-dimethoxyphenylphosphine (TDMPP, 6.3 mg, 0.015 mmol, 3 mol %), ethyl pent-2-ynoate (66 μL, 0.50 mmol), and phenylacetylene (69 μL, 0.625 mmol) in PhMe (0.50 mL). The reaction duration was 3 h. Purification *via* column chromatography (30:1 to 20:1 hexanes:EtOAc) afforded the title compound (96.8 mg, 85%) as a clear, brown oil. $\mathbf{R}_f$ = 0.41 (10:1 hexanes:EtOAc). **^{1}H NMR** (400 MHz; $CDCl_3$): δ 7.49-7.46 (m, 2H), 7.36-7.33 (m, 3H), 6.13 (t, J = 1.0 Hz, 1H), 4.19 (q, J = 7.1 Hz, 2H), 2.85 (qd, J = 7.5, 1.0 Hz, 2H), 1.30 (t, J = 7.1 Hz, 3H), 1.23 (t, J = 7.5 Hz, 3H). **^{13}C NMR** (100 MHz; $CDCl_3$): δ 166.0, 144.4, 132.0, 129.1, 128.6, 123.5, 122.6, 94.6, 90.2, 60.2, 25.9, 14.4, 13.1. **IR** (film): 2935, 2895, 2170, 1690, 1587, 1423, 1349, 1327, 1288, 1250, 1171, 1121, 1019, 860, 746, 680 cm^{-1}. **HRMS** (ESI+): Calculated for $C_{15}H_{16}NaO_2$ $(M+Na)^+$: 251.1043; Found 251.1053.

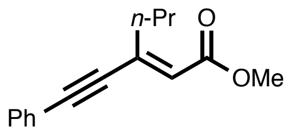

methyl *(E)*-3-(phenylethynyl)hex-2-enoate (**8bh**)

Prepared according to Representative Procedure A using $Pd(OAc)_2$ (3.4 mg, 0.015 mmol, 3 mol %), tris-2,6-dimethoxyphenylphosphine (TDMPP, 6.3 mg, 0.015 mmol, 3 mol %), methyl hex-2-ynoate (67 μL, 0.50 mmol), and phenylacetylene (69 μL, 0.625 mmol) in PhMe (0.50 mL). The reaction duration was 1 h. Purification *via* column chromatography (20:1 to 10:1 hexanes:EtOAc) afforded the title compound (103.9 mg, 91%) as a clear, brown oil. $\mathbf{R}_f$ = 0.26 (10:1 hexanes:EtOAc). **^{1}H NMR** (400 MHz; $CDCl_3$): δ 7.49-7.46 (m, 2H), 7.36-7.33 (m, 3H), 6.17 (s, 1H), 3.73 (s, 3H), 2.84 (t, J = 7.6 Hz, 2H), 1.75-1.66 (m, 2H), 1.01 (t, J = 7.4 Hz, 3H). **^{13}C NMR** (100 MHz; $CDCl_3$): δ 166.4, 143.4, 132.0, 129.1, 128.5, 123.6, 122.5, 94.5, 90.3, 51.4, 34.2, 22.0, 13.9. **IR** (film): 2985, 2908, 2186, 1691, 1590, 1475, 1413, 1325, 1233, 1175, 1146, 1062, 1018, 860 cm^{-1}. **HRMS** (ESI^+): Calculated for $C_{17}H_{20}ClO_2$ (M+H)+: 291.1146; Found 291.1143.

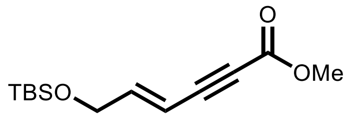

methyl *(E)*-6-((*tert*-butyldimethylsilyl)oxy)hex-4-en-2-ynoate (**7i**)

To a – 78 °C solution of known alkyne *(E)-tert*-butyldimethyl(pent-2-en-4-yn-1-yloxy)silane[8] (589.1 mg, 3.0 mmol, 1.0 equivalent) in THF (12 mL) was added *n*-BuLi (1.6 M in hexanes, 2.06 mL, 3.3 mmol, 1.1 equivalents), yielding a homogeneous, deep pink mixture that soon progressed to homogeneous and red-brown. The reaction mixture was stirred for 30 min at – 78 °C, and then methyl chloroformate (0.29 mL, 3.75 mmol, 1.25 equivalents) was added. This resulted in a change in appearance to homogeneous and golden yellow. The reaction mixture was stirred for 5 min, and then the cooling bath was removed and the vessel allowed to warm to room temperature. After 30 min, the reaction mixture was quenched with saturated aqueous NH_4Cl (20 mL) and poured into Et_2O (20 mL). The phases were separated, and the aqueous phase was extracted with Et_2O (20 mL). The pooled organic phases were dried over $MgSO_4$, filtered, and concentrated. Purification via column chromatography (20:1 to 10:1 hexanes:EtOAc) afforded the title compound (608.3 mg, 80 %) as a clear, yellow oil. $\mathbf{R}_f$ = 0.34 (10:1 hexanes:EtOAc). **^{1}H NMR** (400 MHz; $CDCl_3$): δ 6.56 (dt, *J* = 15.8, 3.6 Hz, 1H), 5.90 (dt, *J* = 15.7, 2.5 Hz, 1H), 4.28 (dd, *J* = 3.6, 2.4 Hz, 2H), 3.79 (s, 3H), 0.91 (s, 9H), 0.07 (s, 6H). **^{13}C NMR** (100 MHz; $CDCl_3$): δ 154.6, 149.8, 105.7, 85.6, 80.7, 62.7, 52.8, 25.9, 18.4, -5.3. **IR** (film): 2914, 2818, 2188, 1693, 1415, 1358, 1224, 1118, 1086, 995, 939, 826, 768, 739, 668 cm^{-1}. **HRMS** (ESI+): Calculated for $C_{13}H_{23}O_3Si$ $(M+H)^+$: 255.1411; Found 255.1406.

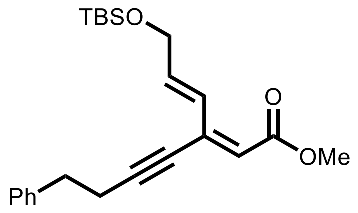

methyl *(E)*-3-(*(E)*-3-((*tert*-butyldimethylsilyl)oxy)prop-1-en-1-yl)-7-phenylhept-2-en-4-ynoate (**8ai**)

Prepared according to Representative Procedure A using $Pd(OAc)_2$ (3.4 mg, 0.015 mmol, 3 mol %), tris-2,6-dimethoxyphenylphosphine (TDMPP, 6.3 mg, 0.015 mmol, 3 mol %), acceptor methyl *(E)*-6-((*tert*-butyldimethylsilyl)oxy)hex-4-en-2-ynoate (127.2 mg, 0.50 mmol) and donor 4-phenyl-1-butyne (88 µL, 0.625 mmol) in PhMe (0.50 mL). The reaction duration was 16 h. Purification *via* column chromatography (20:1 to 10:1 hexanes:EtOAc) afforded the title compound (175.9 mg, 91%) as a clear, red-orange oil. $\mathbf{R_f}$ = 0.38 (10:1 hexanes:EtOAc). **^{1}H NMR** (400 MHz; $CDCl_3$): δ 7.62 (dtd, J = 15.4, 1.9, 0.8 Hz, 1H), 7.33-7.29 (m, 2H), 7.25-7.21 (m, 3H), 6.41 (dtd, J = 15.4, 4.6, 0.7 Hz, 1H), 5.92 (s, 1H), 4.31 (ddd, J = 4.6, 1.9, 0.8 Hz, 2H), 3.72 (s, 3H), 2.90 (t, J = 7.4 Hz, 2H), 2.70 (t, J = 7.4 Hz, 2H), 0.94 (s, 9H), 0.10 (s, 6H). **^{13}C NMR** (100 MHz; $CDCl_3$): δ 166.3, 141.5, 140.4, 136.9, 128.63, 128.58, 126.6, 124.3, 121.1, 95.6, 79.3, 63.4, 51.4, 34.9, 26.1, 21.7, 18.6, -5.1. **IR** (film): 2910, 2816, 2195, 1690, 1616, 1558, 1413, 1339, 1228, 1216, 1143, 1061, 995, 958, 825, 767, 736, 688 cm^{-1}. **HRMS** (ESI+): Calculated for $C_{23}H_{33}O_3Si$ $(M+H)^+$: 385.2193; Found 385.2187.

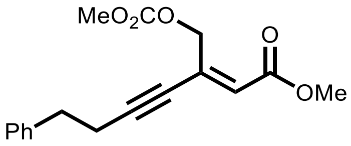

methyl *(Z)*-3-(((methoxycarbonyl)oxy)methyl)-7-phenylhept-2-en-4-ynoate (**8aj**)

Prepared according to Representative Procedure A using $Pd(OAc)_2$ (6.7 mg, 0.03 mmol, 3 mol %), tris(2,6-dimethoxyphenyl)phosphine (13.3 mg, 0.03 mmol, 3 mol %), known acceptor methyl 4-((methoxycarbonyl)oxy)but-2-ynoate[9] (172 mg, 1.0 mmol), and 4-phenyl-1-butyne (176 µL, 1.25 mmol) in PhMe (1.0 mL). The reaction duration was 18 h. Purification *via* column chromatography (6:1 hexanes:EtOAc) afforded the title compound as a dark yellow oil (233 mg, 0.77 mmol, 77% yield). $\mathbf{R_f}$ = 0.40 (6:1 hexanes:EtOAc). **^{1}H NMR** (300 MHz, $CDCl_3$) δ 7.34-7.28 (m, 2H), 7.25-7.20 (m, 3H), 6.08 (t, J = 1.8 Hz, 1H), 5.22 (d, J

= 1.8 Hz, 2H), 3.80 (s, 3H), 3.72 (s, 3H), 2.86 (t, *J* = 7.5 Hz, 2H), 2.65 (t, *J* = 7.6 Hz, 2H). **^{13}C NMR** (75 MHz, $CDCl_3$) δ 165.8, 155.6, 140.2, 137.9, 128.53, 128.46, 126.5, 124.2, 98.9, 78.5, 65.1, 55.1, 51.7, 34.6, 22.0. **IR** (film): 3063, 3028, 2954, 2220, 1755, 1714, 1608, 1496, 1443, 1373, 1338, 1262, 1192, 1166, 1124, 1030, 986, 907, 872, 789, 750, 700 cm^{-1}. **HRMS:** Calculated for $C_{17}H_{19}O_5$ $(M+H)^+$: 303.1227; Found 303.1229.

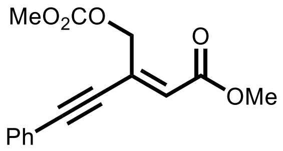

methyl *(Z)*-3-(((methoxycarbonyl)oxy)methyl)-5-phenylpent-2-en-4-ynoate (**8bj**)

Prepared according to Representative Procedure A using $Pd(OAc)_2$ (6.7 mg, 0.03 mmol, 3 mol %), tris(2,6-dimethoxyphenyl)phosphine (13.3 mg, 0.03 mmol, 3 mol %), methyl 4-((methoxycarbonyl)oxy)but-2-ynoate (172 mg, 1.0 mmol), and phenylacetylene (137 μL, 1.25 mmol) in PhMe (1.0 mL). The reaction was stirred for 2 h at room temperature, at which point additional phenylacetylene (55 μL, 0.5 mmol) was added. The reaction mixture was stirred for an additional 2 h 30 min and then worked up as described. Purification *via* column chromatography (8:1 hexanes:EtOAc) afforded the title compound as a brown solid (210 mg, 0.77 mmol, 77% yield). **R_f** = 0.20 (8:1 hexanes:EtOAc). **MP** = 68 – 69 °C. **^{1}H NMR** (300 MHz, $CDCl_3$) δ 7.50-7.45 (m, 2H), 7.39-7.31 (m, 3H), 6.26 (t, *J* = 2.0 Hz, 1H), 5.34 (d, *J* = 2.0 Hz, 2H), 3.82 (s, 3H), 3.76 (s, 3H). **^{13}C NMR** (125 MHz, $CDCl_3$) δ 165.8, 155.8, 137.9, 132.2, 129.6, 128.6, 124.3, 122.0, 97.9, 86.2, 65.2, 55.2, 51.9. **IR** (film): 3021, 2949, 2207, 1760, 1705, 1610, 1593, 1488, 1448, 1435, 1348, 1313, 1260, 1208, 1120, 1071, 1026, 1012, 979, 926, 904, 872, 845, 786, 760, 693 cm$^{-1.}$ **HRMS**: Calculated for $C_{15}H_{15}O_5$ $(M+H)^+$: 275.0914; Found 275.0912.

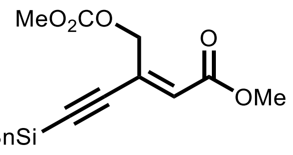

Methyl *(Z)*-5-(benzyldimethylsilyl)-3-(((methoxycarbonyl)oxy)methyl)pent-2-en-4-ynoate (**8ej**)

Prepared according to Representative Procedure A using $Pd(OAc)_2$ (6.7 mg, 0.03 mmol, 3 mol %), tris(2,6-dimethoxyphenyl)phosphine (13.3 mg, 0.03 mmol, 3 mol %), methyl 4-((methoxycarbonyl)oxy)but-2-ynoate (172 mg, 1.0 mmol), and benzyldimethylsilylacetylene (218 mg, 1.25 mmol) in PhMe (1.0 mL). The reaction duration was 9 h. Purification *via* column chromatography (8:1 hexanes:EtOAc) afforded the title compound as a light brown oil (233 mg, 0.67 mmol, 67% yield). $\mathbf{R_f}$ = 0.32 (8:1 hexanes:EtOAc). ^{1}**H NMR** (300 MHz, $CDCl_3$) δ 7.24-7.21 (m, 2H), 7.13-7.06 (m, 3H), 6.18 (t, *J* = 2.0 Hz, 1H), 5.22 (d, *J* = 2.0 Hz, 2H), 3.79 (s, 3H), 3.74 (s, 3H), 2.23 (s, 2H), 0.16 (s, 6H). ^{13}**C NMR** (75 MHz, $CDCl_3$) δ 165.8, 155.8, 138.6, 137.4, 128.6, 128.5, 125.9, 124.8, 102.8, 102.1, 64.9, 55.3, 52.1, 26.0, -2.3. **IR** (film): 3025, 2957, 1757, 1716, 1607, 1494, 1443, 1337, 1239, 1151, 1057, 988, 906, 842, 790, 764, 700 cm^{-1}. **HRMS**: Calculated for $C_{18}H_{23}O_5Si$ $(M+H)^+$: 347.1309; Found 347.1312.

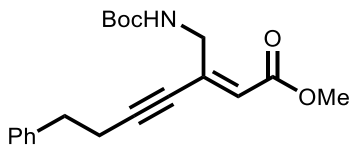

methyl *(Z)*-3-(((*tert*-butoxycarbonyl)amino)methyl)-7-phenylhept-2-en-4-ynoate (**8ak**)

Prepared according to Representative Procedure A using $Pd(OAc)_2$ (3.4 mg, 0.015 mmol, 1.5 mol %), tris-2,6-dimethoxyphenylphosphine (TDMPP, 6.3 mg, 0.015 mmol, 1.5 mol %), 4-phenyl-1-butyne (176 μL, 1.25 mmol), and known acceptor methyl 4-((*tert*-butoxycarbonyl)amino)but-2-ynoate[10] (213 mg, 1.0 mmol) in PhMe (1.0 mL). The reaction duration was 3 h. The crude product was purified by column chromatography (6:1 to 4:1 hexanes:EtOAc) to afford the title compound as a light yellow solid (311 mg, 0.91 mmol, 91% yield). $\mathbf{R_f}$ = 0.30 (4:1 hexanes:EtOAc). **MP** = 84 – 85 °C. ^{1}**H NMR** (400 MHz, $CDCl_3$) δ 7.34-7.30 (m, 2H), 7.25-7.22 (m, 3H), 6.03 (s, 1H), 4.96 (br s, 1H), 4.29 (d, *J* = 5.8 Hz, 2H), 3.71 (s, 3H), 2.89-2.86 (t, *J* = 7.4 Hz, 2H), 2.68-2.65 (t, *J* = 7.5 Hz, 2H), 1.45 (s, 9H). ^{13}**C**

NMR (125 MHz, $CDCl_3$) δ 166.2, 155.8, 141.0, 140.3, 128.64, 128.56, 126.6, 123.7, 98.2, 80.6, 79.5, 51.6, 41.3, 34.7, 28.5, 22.1. **IR** (film): 3419, 2978, 1717, 1605, 1498, 1455, 1368, 1253, 1160, 750, 700 cm^{-1}. **HRMS**: Calculated for $C_{20}H_{25}NNaO_4$ $(M+Na)^+$: 366.1676; Found 366.1682.

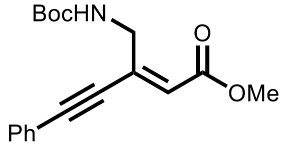

methyl *(Z)*-3-(((*tert*-butoxycarbonyl)amino)methyl)-5-phenylpent-2-en-4-ynoate (**8bk**)

Prepared according to Representative Procedure A using $Pd(OAc)_2$ (3.4 mg, 0.015 mmol, 1.5 mol %), tris-2,6-dimethoxyphenylphosphine (TDMPP, 6.3 mg, 0.015 mmol, 1.5 mol %), phenylacetylene (137 µL, 1.25 mmol), and methyl 4-((*tert*-butoxycarbonyl)amino)but-2-ynoate (213 mg, 1.0 mmol) in PhMe (1.0 mL). The reaction duration was 1 hour. The crude product was purified by column chromatography (5:1 hexanes:EtOAc) to afford the title compound as a white solid (295 mg, 0.94 mmol, 94% yield). **^{1}H NMR** (400 MHz, $CDCl_3$) δ 7.51-7.45 (m, 2H), 7.39-7.30 (m, 3H), 6.21 (t, J = 1.6 Hz, 1H) 5.20-4.85 (N-H, br, 1H), 4.44-4.35 (br, d, J = 6 Hz, 2H), 3.74 (s, 3H), 1.43 (s, 9H). *These ^{1}H NMR data matched that previously reported for this compound.*[10]

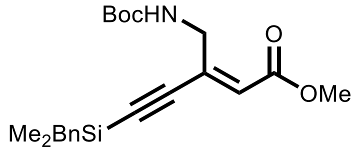

methyl *(Z)*-5-(benzyldimethylsilyl)-3-(((*tert*-butoxycarbonyl)amino)methyl)pent-2-en-4-ynoate (**8ek**)

Prepared according to Representative Procedure A using $Pd(OAc)_2$ (3.4 mg, 0.015 mmol, 1.5 mol %), tris-2,6-dimethoxyphenylphosphine (TDMPP, 6.3 mg, 0.015 mmol, 1.5 mol %), benzyldimethylsilylacetylene (218 mg, 1.25 mmol), and known acceptor methyl 4-

((*tert*-butoxycarbonyl)amino)but-2-ynoate (213 mg, 1.0 mmol) in PhMe (1.0 mL). The reaction duration was 3 h. The crude product was purified by column chromatography (3:1 hexanes:EtOAc) to afford the title compound as a light yellow solid (360 mg, 0.93 mmol, 93% yield). **^{1}H NMR** (500 MHz, $CDCl_3$) δ 7.44 (m, 2H), 7.10 (m, 3H), 6.13 (t, J = 1.75 Hz, 1H), 4.96 (br, N-H, 1H), 4.30 (br, d, J = 5.5 Hz, 2H), 3.72 (s, 3H), 2.24 (s, 2H), 1.45 (s, 9H), 0.18 (s, 6H).

These ^{1}H data matched that previously reported for this compound.[10]

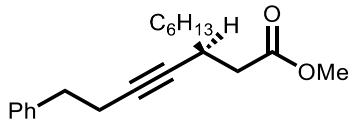

Cu-catalyzed Asymmetric Conjugate Reduction of Ynenoates: Representative Procedure B, Synthesis of methyl *(S)*-3-(4-phenylbut-1-yn-1-yl)nonanoate (**9aa**):

An oven-dried round-bottom Biotage® microwave vial (0.50 – 2.0 mL size) was charged with $Cu(OAc)_2 \bullet H_2O$ (3.0 mg, 0.015 mmol, 5 mol %) and WALPHOS ligand W001-1 (**17**, (*R*)-1-[(*R*)-2-(2'-diphenylphosphinophenyl)-ferrocenyl]-ethyl-di-bis(3,5-trifluoromethyl-phenyl)phosphine, 14.0 mg, 0.015 mmol, 5 mol %). The vial was sealed with a septum, purged with Ar, and kept under an Ar atmosphere (balloon). Distilled, degassed (N_2 sparged) PhMe (1.5 mL) was added, and the mixture was stirred for 30 min. A homogeneous blue-green solution resulted. Diethoxymethylsilane (96 µL, 0.60 mmol, 2 equivalents) was added, and over 30 min, the appearance changed to homogeneous and yellow-brown. The vial was cooled to 0 °C, and methyl *(E)*-3-(4-phenylbut-1-yn-1-yl)non-2-enoate (90.0 mg, 0.30 mmol, 1 equivalent) was added neat *via* microliter syringe, followed by *tert*-butanol (57 µL, 0.60 mmol, 2 equivalents). The vessel was moved to a cold room (4 °C) where it was stirred for 14 h. After this time, the reaction mixture was filtered through a pipette plug of Florisil® (3 cm), which was rinsed with 2:1 hexanes:EtOAc (3 x 3 mL). The solution was concentrated, and the residue was purified *via* column chromatography (20:1 hexanes:EtOAc) to afford the title compound (81.9 mg, 90%, 99% ee) as a light yellow oil. **R_f** = 0.20 (20:1 hexanes:EtOAc). **^{1}H NMR** (400 MHz, $CDCl_3$) δ 7.35-7.30 (m, 2H), 7.24-7.22 (m, 3H), 3.72 (s, 3H), 2.84-2.80 (m, 3H), 2.53-2.40 (m, 4H), 1.49-1.25 (m, 10H), 0.93 (t, J = 7.0 Hz, 3H). **^{13}C NMR** (100 MHz,

$CDCl_3$) δ 172.5, 141.0, 128.6, 128.4, 126.2, 82.6, 81.2, 51.7, 40.5, 35.6, 35.0, 31.9, 29.1, 28.4, 27.2, 22.8, 21.1, 14.2. **IR** (film): 2953, 2928, 2857, 1742, 1455, 1436, 1163, 699 cm^{-1}. **HRMS**: Calculated for $C_{20}H_{29}O_2$ $(M+H)^+$: 301.2162, Found 301.2163. **Chiral HPLC:** Chiralpak® IB column; heptane/isopropanol 999:1; flow rate = 0.4 mL/min; detection at 214 nm; t_1= 31.0 min (major); t_2 = 33.7 min (minor). **$[\alpha]_D^{25}$** = + 4.95° (*c* = 0.76, CH_2Cl_2, 99% ee)

Note 1: Filtration of the reaction mixture through Florisil® removes the catalyst and ligand, providing a solution containing the desired product and silane-containing byproducts. The pure product was typically isolated free of these byproducts after chromatography; however, on some occasions, the byproducts co-eluted. In these cases, the impure material was dissolved in THF and treated (at 23 °C) with TBAF (1 M in THF), which rapidly destroyed the silane-derived impurities. Chromatography on silica gel then provided analytically pure products.

Note 2: Very viscous substrates could alternatively be added to the catalyst solution as a solution in PhMe.

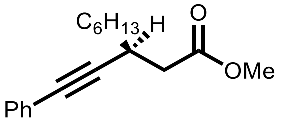

methyl *(S)*-3-(phenylethynyl)nonanoate (**9ba**):

Prepared according to Representative Procedure B using $Cu(OAc)_2•H_2O$ (3.0 mg, 0.015 mmol, 5 mol %), WALPHOS ligand **17** (14.0 mg, 0.015 mmol, 5 mol %), methyl *(E)*-3-(phenylethynyl)non-2-enoate (81.0 mg, 0.30 mmol), diethoxymethylsilane (96 µL, 0.60 mmol, 2 equivalents), and *tert*-butanol (57 µL, 0.60 mmol, 2 equivalents) in PhMe (1.5 mL). The reaction duration was 14 h. Purification *via* column chromatography (20:1 hexanes:EtOAc) afforded the title compound (73.7 mg, 90%, 99% ee) as a light yellow oil. **R_f** = 0.20 (20:1 hexanes:EtOAc).**^1H NMR** (500 MHz, $CDCl_3$) δ 7.39-7.37 (m, 2H), 7.29-7.26 (m, 3H), 3.72 (s, 3H), 3.10-3.04 (m, 1H), 2.64-2.60 (dd, *J* = 15.3 Hz, 7.6 Hz, 1H), 2.55-2.50

(dd, J = 15.3 Hz, 7.0 Hz, 1H), 1.58-1.43 (m, 4H), 1.31-1.29 (m, 6H), 0.89 (t, J = 6.7 Hz, 3H). **^{13}C NMR** (100 MHz, $CDCl_3$) δ 172.3, 131.8, 128.3, 127.8, 123.7, 91.6, 82.2, 51.9, 40.2, 34.8, 31.9, 29.14, 29.07, 27.3, 22.8, 14.2. **IR** (film): 2954, 2929, 2857, 1742, 1438, 1164, 756 cm^{-1}. **HRMS**: Calculated for $C_{18}H_{25}O_2$ $(M+H)^+$: 273.1849; Found 273.1847. **Chiral HPLC:** Chiralpak® IB column; heptane/isopropanol 999:1; flow rate = 0.5 mL/min; detection at 214 nm; t_1 = 18.4 min (major); t_2 = 35.2 min (minor). **$[\alpha]_D^{25}$** = + 1.42° (c = 0.73, CH_2Cl_2, 99% ee).

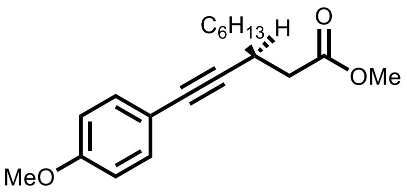

methyl *(S)*-3-((4-methoxyphenyl)ethynyl)nonanoate (**9ca**):

Prepared according to Representative Procedure B using $Cu(OAc)_2$•H_2O (2.0 mg, 0.01 mmol, 5 mol %), WALPHOS ligand **17** (9.3 mg, 0.01 mmol, 5 mol %), diethoxymethylsilane (64 µL, 0.40 mmol), *tert*-butanol (38 µL, 0.40 mmol), and methyl *(E)*-3-((4-methoxyphenyl)ethynyl)non-2-enoate (60.0 mg, 0.20 mmol) in PhMe (1.0 mL). The reaction duration was 16 h. Purification *via* column chromatography (10:1 to 8:1 hexanes:EtOAc) afforded the title compound (57.2 mg, 95%, *ca*. 90% purity as judged by ^{1}H NMR, 99% ee) as a clear, faintly yellow oil. **R_f** = 0.29 (8:1 hexanes:EtOAc). **^{1}H NMR** (400 MHz; $CDCl_3$): δ 7.33-7.29 (m, 2H), 6.81-6.79 (m, 2H), 3.79 (s, 3H), 3.71 (s, 3H), 3.08-3.01 (m, 1H), 2.61 (dd, J = 15.2, 7.6 Hz, 1H), 2.51 (dd, J = 15.3, 7.1 Hz, 1H), 1.58-1.27 (m, 10H), 0.89 (t, J = 6.9 Hz, 3H). **^{13}C NMR** (100 MHz; $CDCl_3$): 172.4, 159.3, 133.1, 115.9, 113.9, 90.0, 81.9, 55.4, 51.8, 40.3, 34.9, 31.9, 29.1, 29.0, 27.3, 22.8, 14.2. **IR** (film): 2888, 2817, 1716, 1585, 1489, 1418, 1271, 1230, 1154, 1091, 1019, 821 cm^{-1}. **HRMS** (ESI+): Calculated for $C_{19}H_{27}O_3$ $(M+H)^+$: 303.1955; Found 303.1946. **Chiral HPLC**: CHIRALPAK® AD-H, 99:1 heptane:isopropanol, 0.5 mL/min, 254 nm, 21.51 min (major), 24.19 min (minor).**$[\alpha]_D^{25}$** = − 2.7° (c = 0.50, $CHCl_3$).

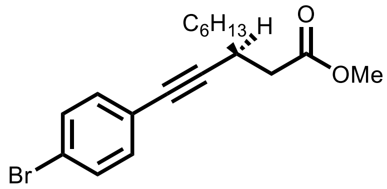

methyl *(S)*-3-((4-bromophenyl)ethynyl)nonanoate (**9da**):

Prepared according to Representative Procedure B using $Cu(OAc)_2$•H_2O (2.0 mg, 0.01 mmol, 5 mol %), WALPHOS ligand **17** (9.3 mg, 0.01 mmol, 5 mol %), diethoxymethylsilane (64 µL, 0.40 mmol), *tert*-butanol (38 µL, 0.40 mmol), and methyl *(E)*-3-((4-bromophenyl)ethynyl)non-2-enoate (69.9 mg, 0.20 mmol) in PhMe (1.0 mL). The reaction duration was 18 h. After this time, the reaction mixture was passed through a silica gel column (eluting with 20:1 hexanes:EtOAc) to afford the product contaminated with small amounts of silane and/or byproducts thereof. This material was treated with TBAF (1 M in THF, 0.30 mL, 0.30 mmol), and the mixture was stirred for 10 min. The mixture was then directly chromatographed on silica gel (20:1 hexanes:EtOAc) to afford the title compound (59.6 mg, 85%, 99% ee) as a viscous, clear, light yellow oil, free of the silicon byproducts. **R*f*** = 0.39 (8:1 hexanes:EtOAc). **^{1}H NMR** (400 MHz; $CDCl_3$): δ 7.42-7.39 (m, 2H), 7.25-7.22 (m, 2H), 3.71 (s, 3H), 3.08-3.02 (m, 1H), 2.60 (dd, *J* = 15.3, 7.7 Hz, 1H), 2.51 (dd, *J* = 15.4, 7.0 Hz, 1H), 1.57-1.42 (m, 4H), 1.34-1.25 (m, 6H), 0.89 (t, *J* = 6.8 Hz, 3H). **^{13}C NMR** (100 MHz; $CDCl_3$): 172.2, 133.2, 131.5, 122.7, 122.0, 92.9, 81.2, 51.9, 40.0, 34.7, 31.9, 29.11, 29.10, 27.3, 22.8, 14.2. **IR** (film): 2887, 2816, 1717, 1465, 1416, 1342, 1148, 1056, 997, 812 cm^{-1}. **HRMS** (ESI+): Calculated for $C_{18}H_{24}O_2Br$ $(M+H)^+$: 351.0954; Found 351.0948. **Chiral HPLC**: Agilent IB, 400:1 heptane:isopropanol, 0.8 mL/min, 254 nm, 8.13 min (major), 8.96 min (minor). $[\alpha]_D^{25}$ = − 2.2° (*c* = 0.50, $CHCl_3$)

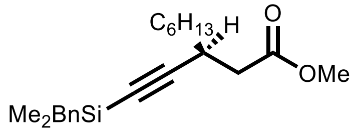

methyl *(S)*-3-((benzyldimethylsilyl)ethynyl)nonanoate (**9ea**):

Prepared according to Representative Procedure B using $Cu(OAc)_2 \cdot H_2O$ (3.0 mg, 0.015 mmol, 5 mol %), WALPHOS ligand **17** (14.0 mg, 0.015 mmol, 5 mol %), methyl *(E)*-3-((benzyldimethylsilyl)ethynyl)non-2-enoate (103.0 mg, 0.30 mmol), diethoxymethylsilane (96 µL, 0.60 mmol, 2 equivalents), and *tert*-butanol (57 µL, 0.60 mmol, 2 equivalents) in PhMe (1.5 mL). The reaction duration was 14 h. The crude product was purified by column chromatography (20:1 hexanes:EtOAc) to afford the title compound as a light yellow oil (90.2 mg, 0.26 mmol, 87% yield, 99% ee). $\mathbf{R_f}$ = 0.23 (20:1 hexanes:EtOAc). **^{1}H NMR** (500 MHz, $CDCl_3$) δ 7.23-7.20 (m, 2H), 7.10-7.06 (m, 3H), 3.69 (s, 3H), 2.89-2.83 (m, 1H) 2.54-2.40 (dd, J = 15.3 Hz, 7.4 Hz, 1H), 2.44-2.40 (dd, J = 15.4 Hz, 7.3 Hz, 1H), 2.16 (s, 2H), 1.48-1.26 (m, 10H), 0.89 (t, J = 6.9 Hz, 3H), 0.10 (s, 6H). **^{13}C NMR** (125 MHz, $CDCl_3$) δ 172.1, 139.3, 128.5, 128.2, 124.4, 110.1, 84.4, 51.8, 40.0, 34.5, 31.8, 29.4, 29.1, 27.1, 26.6, 22.7, 14.2, -1.8. **IR** (film): 3025, 2956, 2929, 2858, 2710, 1250, 1208, 1160, 839, 698 cm^{-1}. **HRMS**: Calculated for $C_{21}H_{33}O_2Si$ $(M+H)^+$: 345.2244; Found 345.2248. $[\alpha]_D^{25}$ = + 1.03° (c = 1.18, CH_2Cl_2, 99% ee). **Chiral HPLC:** Chiralpak® IB column; heptane/isopropanol 999:1; flow rate = 0.5 mL/min; detection at 214 nm; t_1= 14.2 min (major); t_2 = 16.2 min (minor).

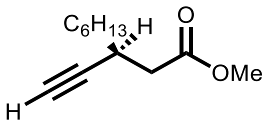

methyl *(S)*-3-ethynylnonanoate (**9fa**):

Prepared using a modification of Representative Procedure B. A flame-dried 50 mL flask equipped with a stir bar was charged with $Cu(OAc)_2 \cdot H_2O$ (12.5 mg, 0.0625 mmol, 5 mol %) and WALPHOS ligand **17** (58.2 mg, 0.0625 mmol, 5 mol %). The flask was sealed with a septum and flushed with Ar (balloon). Freshly distilled, degassed (Ar sparge) PhMe (10.0 mL) was added, and the mixture was stirred for 20 min to afford a bright green, homogeneous solution. Diethoxymethylsilane (0.80 mL, 5 mmol, 2 equivalents) was added, and the mixture was stirred until a change in appearance to homogeneous and brown was observed (< 30 min). The flask was cooled to 0 °C, and a solution of methyl *(E)*-3-((trimethylsilyl)ethynyl)non-2-enoate (666.1 mg, 2.5 mmol, 1.0 equivalent) in PhMe (2 mL)

was added *via* cannula syringe, using additional PhMe (0.50 mL) to rinse the vial containing the substrate and the cannula. To the mixture was then added *tert*-butanol (0.48 mL, 5 mmol, 2 equivalents). The vessel was kept under an Ar balloon and transferred to a cold room (4 °C) where it was stirred for 18 h.

The reaction mixture was then warmed to room temperature and loaded directly onto a silica gel column that was eluted with 40:1 to 30:1 hexanes:EtOAc. The fractions containing the TMS-protected, 1,4-reduction product (R_f = 0.53, 10:1 hexanes:EtOAc) were concentrated, and this crude material was redissolved in THF (2.5 mL) and treated with TBAF (1 M in THF, 6.0 mL, 6.0 mmol, 2.4 equivalents). The resulting black solution was stirred for 30 minutes, at which point it was poured into a mixture of saturated NH_4Cl (10 mL) and Et_2O (25 mL). The phases were separated, and the aqueous phase was extracted with Et_2O (25 mL). The pooled organic phases were dried over $MgSO_4$, filtered, and concentrated. The crude material was purified by column chromatography (30:1 hexanes:EtOAc) to deliver the title compound (378.9 mg, 77%) as a light yellow, clear oil. **R_f** = 0.47 (10:1 hexanes:EtOAc). **^{1}H NMR** (400 MHz; $CDCl_3$): δ 3.69 (s, 3H), 2.84-2.81 (m, 1H), 2.53 (dd, J = 15.5, 7.8 Hz, 1H), 2.43 (dd, J = 15.5, 6.9 Hz, 1H), 2.06 (d, J = 2.4 Hz, 1H), 1.51-1.27 (m, 10H), 0.87 (t, J = 6.3 Hz, 3H). **^{13}C NMR** (100 MHz; $CDCl_3$): δ 172.1, 86.1, 69.8, 51.8, 39.9, 34.5, 31.8, 29.1, 28.1, 27.1, 22.7, 14.2. **IR** (film): 3263, 2889, 2818, 1718, 1418, 1342, 1233, 1149, 1094, 1006, 871, 626 cm^{-1}. **HRMS** (ESI+): Calculated for $C_{12}H_{20}NaO_2$ $(M+H)^+$: 219.1356; Found 219.1359. **$[\alpha]_D^{25}$** = – 6.6 ° (c = 0.5, $CHCl_3$). Determination of the ee: a sample of the ester obtained was converted to a mixture of diastereomeric amides *via* hydrolysis (3 equiv KOH, 3:1:1 MeOH:THF:H_2O, 50 °C for 1 h) and subsequent amidation with *(S)*-α-methylbenzylamine (EDCI-HCl, 5 equiv of amine, 1.5 equiv DMAP, CH_2Cl_2, overnight). After extractive workup, drying of the organic phase, and concentration, the crude material was analyzed by ^{1}H NMR, which indicated a single diastereomer (> 19:1 dr or > 90% ee). This was verified upon the independent synthesis of the corresponding 1:1 mixture of diastereomeric amides by the analogous amidation of racemic β-alkynyl ester. Well-resolved diagnostic peaks include those for the terminal alkyne C-H (δ 2.13, d, J = 2.4 Hz, minor and δ 2.07, d, J = 2.4 Hz, major).

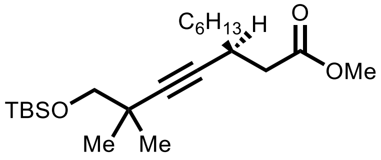

methyl *(S)*-3-(4-((*tert*-butyldimethylsilyl)oxy)-3,3-dimethylbut-1-yn-1-yl)nonanoate (**9ga**):

Prepared according to Representative Procedure B using $Cu(OAc)_2$•H_2O (2.0 mg, 0.01 mmol, 5 mol %) WALPHOS ligand **17** (9.3 mg, 0.01 mmol, 5 mol %), diethoxymethylsilane (64 μL, 0.40 mmol), *tert*-butanol (38 μL, 0.40 mmol), and methyl *(E)*-3-(4-((*tert*-butyldimethylsilyl)oxy)-3,3-dimethylbut-1-yn-1-yl)non-2-enoate (76.1 mg, 0.20 mmol) in PhMe (1.0 mL). The reaction duration was 18 h. Purification *via* column chromatography (20:1 hexanes:EtOAc) afforded the title compound (72.8 mg, 95%) as a viscous, light yellow oil. $\mathbf{R}_f$ = 0.48 (8:1 hexanes:EtOAc). **^{1}H NMR** (500 MHz; $CDCl_3$): δ 3.68 (s, 3H), 3.37 (s, 2H), 2.81-2.76 (m, 1H), 2.45 (dd, *J* = 14.9, 7.5 Hz, 1H), 2.38 (dd, *J* = 14.9, 7.3 Hz, 1H), 1.43-1.25 (m, 10H), 1.11 (s, 6H), 0.90-0.87 (m, 12H), 0.04 (s, 6H). **^{13}C NMR** (100 MHz; $CDCl_3$): 172.6, 88.2, 81.8, 71.6, 51.7, 40.8, 35.0, 33.5, 31.9, 29.1, 28.6, 27.2, 26.1, 25.89, 25.88, 22.8, 18.5, 14.3, -5.2. **IR** (film): 2915, 2889, 2818, 1721, 1449, 1417, 1341, 1235, 1146, 1090, 826, 765 cm^{-1}. **HRMS** (ESI+): Calculated for $C_{22}H_{43}O_3Si(M+H)^+$: 383.2976; Found 383.2975. $[\alpha]_D^{25}$ = − 1.7° (*c* = 0.50, $CHCl_3$). Determination of the ee: analogously to the previous example, a sample of the material was hydrolyzed and amidated with *(S)*-α-methylbenzylamine. > 19:1 dr (corresponding to > 90% ee) was observed in the ^{1}H NMR spectrum of the mixture of amides, which was verified by the independent synthesis of a 1:1 mixture of diastereomers from racemic material. Well-resolved diagnostic peaks include those for the methylene α to oxygen (6.28, d, *J* = 7.8 Hz, major), 6.25 (d, *J* = 8.2 Hz, minor).

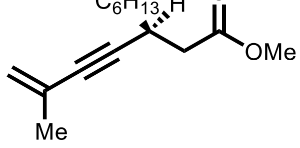

methyl *(S)*-3-(3-methylbut-3-en-1-yn-1-yl)nonanoate (**9ha**):

Prepared according to Representative Procedure B using $Cu(OAc)_2$•H_2O (1.0 mg, 0.005 mmol, 5 mol %), WALPHOS ligand **17** (4.7 mg, 0.005 mmol, 5 mol %), diethoxymethylsilane (32 µL, 0.20 mmol), *tert*-butanol (19 µL, 0.20 mmol), and methyl *(E)*-3-(3-methylbut-3-en-1-yn-1-yl)non-2-enoate (23.4 mg, 0.10 mmol) in PhMe (0.5 mL). The reaction duration was 15 h. After this time, the reaction mixture was treated, at 4 °C, with TBAF (1 M in THF, 0.35 mL, 0.35 mmol) to consume silane byproducts previously found to be inseparable from the product *via* chromatography. Vigorous gas evolution occurred, and the dark red/brown solution was stirred for 10 min. The reaction mixture was then directly chromatographed on silica gel (20:1 hexanes:EtOAc) to afford the title compound (21.6 mg, 92%, 99% ee) as a viscous, clear, light yellow oil. $\mathbf{R_f}$ = 0.42 (8:1 hexanes:EtOAc). ^{1}**H NMR** (400 MHz; $CDCl_3$): δ 5.20-5.18 (m, 1H), 5.15-5.13 (m, 1H), 3.69 (s, 3H), 2.98-2.91 (m, 1H), 2.53 (dd, J = 15.2, 7.5 Hz, 1H), 2.45 (dd, J = 15.2, 7.2 Hz, 1H), 1.85 (dd, J = 1.5, 1.0 Hz, 3H), 1.51-1.41 (m, 4H), 1.31-1.25 (m, 6H), 0.87 (d, J = 7.0 Hz, 3H). ^{13}**C NMR** (100 MHz; $CDCl_3$): 172.3, 127.2, 120.9, 90.6, 83.4, 51.8, 40.2, 34.8, 31.9, 29.1, 28.9, 27.2, 23.9, 22.7, 14.2. **IR** (film): 2913, 2888, 2818, 1719, 1593, 1417, 1354, 1231, 1147, 997, 880 cm^{-1}. **HRMS** (ESI+): Calculated for $C_{15}H_{25}O_2$ $(M+H)^+$: 237.1849; Found 237.1843. **Chiral HPLC**: Agilent IB, 2000:1 heptane:isopropanol, 0.4 mL/min, 220 nm, 17.92 min (major), 19.28 min (minor). $[\alpha]_D^{24}$ = − 5.0° (c = 0.50, $CHCl_3$).

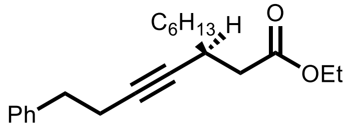

ethyl *(S)*-3-(4-phenylbut-1-yn-1-yl)nonanoate (**9ab**):

Prepared according to Representative Procedure B using $Cu(OAc)_2$•H_2O (2.0 mg, 0.01 mmol, 5 mol %), WALPHOS ligand **17** (9.3 mg, 0.01 mmol, 5 mol %), diethoxymethylsilane (64 µL, 0.40 mmol), *tert*-butanol (38 µL, 0.40 mmol), and ethyl *(E)*-3-(4-phenylbut-1-yn-1-yl)non-2-enoate (62.5 mg, 0.20 mmol) in PhMe (1.0 mL). The reaction duration was 16 h. Purification *via* column chromatography (20:1 hexanes:EtOAc) afforded the title compound

(53.0 mg, 84%, 98% ee) as a clear, faintly yellow oil. **R$_f$** = 0.43 (8:1 hexanes:EtOAc). **^{1}H NMR** (400 MHz; $CDCl_3$): δ 7.30-7.26 (m, 2H), 7.22-7.18 (m, 3H), 4.14 (q, *J* = 7.1 Hz, 2H), 2.78 (t, *J* = 7.5 Hz, 3H), 2.47-2.36 (m, 4H), 1.45-1.22 (m, 13H), 0.89 (t, *J* = 6.9 Hz, 3H). **^{13}C NMR** (100 MHz; $CDCl_3$): 172.0, 141.1, 128.6, 128.4, 126.3, 82.7, 81.1, 60.5, 40.8, 35.6, 35.0, 31.9, 29.2, 28.5, 27.2, 22.8, 21.1, 14.4, 14.3. **IR** (film): 3020, 2986, 2887, 2817, 1713, 1582, 1475, 1434, 1352, 1328, 1229, 1145, 1091, 1062, 1019 cm^{-1}. **HRMS** (ESI+): Calculated for $C_{21}H_{31}O_2$ $(M+H)^+$: 315.2319; Found 315.2313. **Chiral HPLC**: Agilent IB, 1000:1 heptane:isopropanol, 0.8 mL/min, 214 nm, 14.52 min (major), 16.73 min (minor). **$[\alpha]_D^{25}$** = − 4.2° (*c* = 0.50, $CHCl_3$).

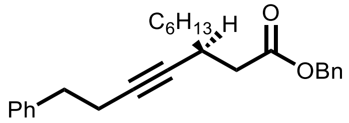

benzyl *(S)*-3-(4-phenylbut-1-yn-1-yl)nonanoate (**9ac**):

Prepared according to Representative Procedure B using $Cu(OAc)_2 \cdot H_2O$ (2.0 mg, 0.01 mmol, 5 mol %), WALPHOS ligand **17** (9.3 mg, 0.01 mmol, 5 mol %), diethoxymethylsilane (64 μL, 0.40 mmol), *tert*-butanol (38 μL, 0.40 mmol), and benzyl *(E)*-3-(4-phenylbut-1-yn-1-yl)non-2-enoate (74.9 mg, 0.20 mmol) in PhMe (1.0 mL). The reaction duration was 18 h. Purification *via* column chromatography (20:1 hexanes:EtOAc) afforded the title compound (64.0 mg, 85%, 99% ee) as a clear, light yellow oil. **R$_f$** = 0.45 (10:1 hexanes:EtOAc). **^{1}H NMR** (400 MHz; $CDCl_3$): δ 7.37-7.27 (m, 7H), 7.22-7.18 (m, 3H), 5.14 (d, *J* = 1.2 Hz, 2H), 2.85-2.80 (m, 1H), 2.76 (t, *J* = 7.5 Hz, 2H), 2.54-2.39 (m, 4H), 1.44-1.24 (m, 10H), 0.89 (t, *J* = 7.0 Hz, 3H). **^{13}C NMR** (100 MHz; $CDCl_3$): 171.8, 141.0, 136.1, 128.7, 128.6, 128.4, 128.32, 128.31, 126.3, 82.6, 81.3, 66.4, 40.7, 35.6, 35.0, 31.9, 29.1, 28.6, 27.2, 22.8, 21.1, 14.2 **IR** (film): 2987, 2887, 1716, 1582, 1476, 1434, 1360, 1331, 1141, 1091, 986, 737, 688 cm^{-1}. **HRMS** (ESI+): Calculated for $C_{26}H_{33}O_2$ $(M+H)^+$: 377.2475; Found 377.2470. **Chiral HPLC**: Agilent IB, 99:1 heptane:isopropanol, 0.8 mL/min, 220 nm, 8.89 min (minor), 10.52 min (major). **$[\alpha]_D^{25}$** = − 3.8° (*c* = 0.50, $CHCl_3$).

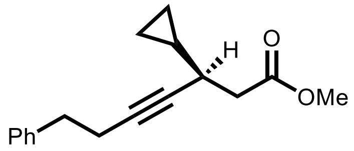

methyl *(R)*-3-cyclopropyl-7-phenylhept-4-ynoate (**9ad**):

Prepared according to Representative Procedure B using $Cu(OAc)_2•H_2O$ (1.0 mg, 0.005 mmol, 5 mol %), WALPHOS ligand **17** (4.7 mg, 0.005 mmol, 5 mol %), diethoxymethylsilane (32 μL, 0.20 mmol), *tert*-butanol (19 μL, 0.20 mmol), and methyl *(E)*-3-cyclopropyl-7-phenylhept-2-en-4-ynoate (23.4 mg, 0.10 mmol) in PhMe (0.5 mL). The reaction duration was 24 h. After this time, the reaction mixture was warmed to room temperature, diluted with Et_2O (3 mL), and filtered through a short pipette plug of Florisil®. The solution was concentrated, and ^1H NMR analysis of the crude material indicated 68% conversion and *ca*. 4:1 1,4:Σ1,6 regioselectivity. The residue was purified by preparative thin-layer chromatography (20:1 hexanes:EtOAc) to afford the title compound (13.9 mg, 54% yield, 99% ee) as a clear, light yellow oil. $\mathbf{R_f}$ = 0.29 (10:1 hexanes:EtOAc). **^{1}H NMR** (600 MHz; $CDCl_3$): δ 7.30-7.27 (m, 2H), 7.21-7.16 (m, 3H), 3.68 (s, 3H), 2.79-2.76 (m, 2H), 2.62-2.58 (m, 1H), 2.55-2.52 (m, 2H), 2.44-2.41 (m, 2H), 0.88-0.82 (m, 1H), 0.44-0.38 (m, 2H), 0.30-0.25 (m, 2H). **^{13}C NMR** (100 MHz; $CDCl_3$): 172.4, 141.0, 128.6, 128.4, 126.3, 81.6, 80.4, 51.8, 40.9, 35.5, 32.2, 21.0, 14.8, 3.8, 2.5. **IR** (film): 3038, 2961, 2908, 1716, 1581, 1475, 1434, 1416, 1334, 1250, 1149, 1006, 814, 737, 689 cm^{-1}. **HRMS** (ESI+): Calculated for $C_{17}H_{21}O_2$ $(M+H)^+$: 257.1536; Found 257.1529. **Chiral HPLC**: Agilent IB, 400:1 heptane:isopropanol, 0.8 mL/min, 214 nm, 14.66 min (major), 17.34 min (minor). $[\alpha]_D^{25}$ = −34.7° (*c* = 0.50, $CHCl_3$).

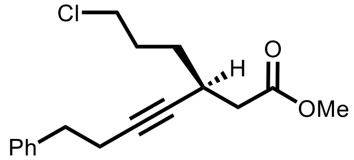

methyl *(S)*-3-(3-chloropropyl)-7-phenylhept-4-ynoate (**9ae**):

Prepared according to Representative Procedure B using $Cu(OAc)_2 \cdot H_2O$ (2.0 mg, 0.01 mmol, 5 mol %), WALPHOS ligand **17** (9.3 mg, 0.01 mmol, 5 mol %), diethoxymethylsilane (64 µL, 0.40 mmol), *tert*-butanol (38 µL, 0.40 mmol), and methyl *(E)*-3-(3-chloropropyl)-7-phenylhept-2-en-4-ynoate (58.2 mg, 0.20 mmol) in PhMe (1.0 mL). The reaction duration was 16 h. Purification *via* column chromatography (8:1 hexanes:EtOAc) afforded the title compound (55.0 mg, 94%, 99% ee) as a clear, light yellow oil. $\mathbf{R_f}$ = 0.27 (8:1 hexanes:EtOAc). **^{1}H NMR** (400 MHz; $CDCl_3$): δ 7.31-7.27 (m, 2H), 7.23-7.19 (m, 3H), 3.69 (s, 3H), 3.52 (t, J = 6.6 Hz, 2H), 2.86-2.81 (m, 1H), 2.78 (t, J = 7.5 Hz, 2H), 2.52-2.36 (m, 4H), 1.95-1.74 (m, 2H), 1.67-1.57 (m, 1H), 1.50-1.40 (m, 1H). **^{13}C NMR** (100 MHz; $CDCl_3$): 172.1, 140.9, 128.6, 128.4, 126.3, 82.0, 81.8, 51.8, 44.9, 40.4, 35.4, 32.1, 30.3, 27.9, 20.9. **IR** (film): 2909, 2887, 1715, 1432, 1416, 1344, 1247, 1154, 1061, 737, 690 cm^{-1}. **HRMS** (ESI+): Calculated for $C_{17}H_{22}ClO_2$ $(M+H)^+$: 293.1303; Found 293.1307. **Chiral HPLC**: Agilent IB, 500:1 heptane:isopropanol, 0.5 mL/min, 220 nm, 43.90 min (major), 46.61 min (minor). $[\alpha]_D^{25}$ = − 3.2° (c = 0.50, $CHCl_3$)

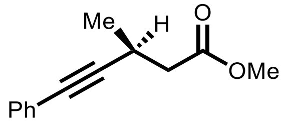

methyl *(S)*-3-methyl-5-phenylpent-4-ynoate (**9bf**):

Prepared according to Representative Procedure B using $Cu(OAc)_2 \cdot H_2O$ (1.0 mg, 0.005 mmol, 5 mol %), WALPHOS ligand **17** (4.7 mg, 0.005 mmol, 5 mol %), diethoxymethylsilane (32 µL, 0.20 mmol), *tert*-butanol (19 µL, 0.20 mmol), and methyl *(E)*-3-methyl-5-phenylpent-2-en-4-ynoate (20.0 mg, 0.10 mmol) in PhMe (0.5 mL). The reaction duration was 14 h. After this time, the reaction mixture was warmed to room temperature, diluted with Et_2O (3 mL), and filtered through a short pipette plug of Florisil®. The solution was concentrated, and ^{1}H NMR analysis of the crude material indicated *ca*. 2.3:1 1,4:Σ1,6 regioselectivity (determined by integration of the methyl ester signals). The crude product was purified by column chromatography (30:1 hexanes:EtOAc) to afford the analytically pure title compound as a clear oil (11.9 mg, 0.059 mmol, 59% yield, 99% ee). $\mathbf{R_f}$ = 0.40 (10:1

hexanes:EtOAc). **^{1}H NMR** (300 MHz, $CDCl_3$) δ 7.40-7.37 (m, 2H), 7.29-7.27 (m, 3H), 3.72 (s, 3H), 3.24-3.12 (sex, *J* = 7.1 Hz, 1H), 2.70-2.62 (dd, *J* = 15.4 Hz, 7.1 Hz, 1H), 2.53-2.45 (dd, *J* = 15.4 Hz, 7.6 Hz, 1H), 1.33 (d, *J* = 6.9 Hz, 3H). **^{13}C NMR** (75 MHz, $CDCl_3$) δ 172.1, 131.7, 128.3, 127.9, 123.6, 92.6, 81.2, 51.8, 41.5, 23.5, 20.9. **IR** (film): 2975, 2952, 1740, 1490, 1438, 1358, 1281, 1253, 1171, 757, 692 cm^{-1}. **HRMS:** Calculated for $C_{13}H_{14}O_2Na$ $(M+Na)^+$: 225.0891; Found 225.0896. **Chiral HPLC:** Chiralpak® IB column; heptane/isopropanol 500:1; flow rate = 0.8 mL/min; detection at 214 nm; t_1= 11.9 min (major); t_2 = 15.7 min (minor)**.** $[\alpha]_D^{25}$ = + 34.56° (*c* = 0.71, CH_2Cl_2, 99% ee).

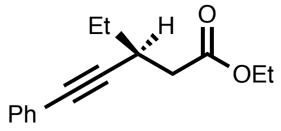

ethyl *(S)*-3-ethyl-5-phenylpent-4-ynoate (**9bg**):

Prepared according to Representative Procedure B using $Cu(OAc)_2$•H_2O (2.0 mg, 0.01 mmol, 5 mol %), WALPHOS ligand **17** (9.3 mg, 0.01 mmol, 5 mol %), diethoxymethylsilane (64 μL, 0.40 mmol), *tert*-butanol (38 μL, 0.40 mmol), and ethyl *(E)*-3-ethyl-5-phenylpent-2-en-4-ynoate (45.6 mg, 0.20 mmol) in PhMe (1.0 mL). The reaction duration was 16 h. Purification *via* column chromatography (20:1 to 10:1 hexanes:EtOAc) afforded the title compound (50.0 mg, > 100%, 99% ee) as a clear, yellow oil and as an inseparable mixture of regioisomers (2.7:1 1,4:Σ1,6, determined by integration of the signals corresponding to the ethyl ester methylene groups). **R_f** = 0.31 (10:1 hexanes:EtOAc). **^{1}H NMR** (600 MHz, $CDCl_3$, major isomer) δ 7.39-7.38 (m, 2H), 7.28-7.26 (m, 3H), 4.18 (q, *J* = 7.1 Hz, 2H), 3.05-3.00 (m, 1H), 2.60 (dd, *J* = 15.2, 7.7 Hz, 1H), 2.51 (dd, *J* = 15.2, 7.0 Hz, 1H), 1.68-1.63 (m, 1H), 1.58-1.53 (m, 1H), 1.27 (t, *J* = 7.1 Hz, 3H), 1.09 (t, *J* = 7.4 Hz, 3H). **^{13}C NMR** (100 MHz, $CDCl_3$, major isomer) δ 171.8, 131.8, 128.3, 127.8, 123.8, 91.5, 82.3, 68.3, 60.7, 40.1, 30.7, 27.9, 14.4, 11.8 . **IR** (film): 2924, 2890, 1712, 1576, 1469, 1441, 1352, 1256, 1157, 1019, 746, 683 cm^{-1}. **HRMS:** Calculated for $C_{15}H_{18}NaO_2$ $(M+Na)^+$: 253.1199; Found 253.1201. **Chiral HPLC:**

Chiralpak® IA column; heptane/isopropanol 500:1; flow rate = 0.8 mL/min; detection at 220 nm; t_1= 10.0 min (major); t_2 = 14.3 min (minor). **[α]$_D^{25}$** = + 13.5° (*c* = 0.50, $CHCl_3$).

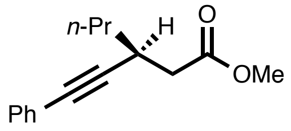

methyl *(S)*-3-(phenylethynyl)hexanoate (**9bh**):

Prepared according to Representative Procedure B using $Cu(OAc)_2$•H_2O (2.0 mg, 0.01 mmol, 5 mol %), WALPHOS ligand **17** (9.3 mg, 0.01 mmol, 5 mol %), diethoxymethylsilane (64 μL, 0.40 mmol), *tert*-butanol (38 μL, 0.40 mmol), and methyl *(E)*-3-(phenylethynyl)hex-2-enoate (45.6 mg, 0.20 mmol) in PhMe (1.0 mL). The reaction duration was 16 h. The reaction mixture was filtered through Florisil®, and the solution was concentrated. ^{1}H NMR analysis of the crude material indicated *ca*. 12.5:1 1,4:Σ1,6 regioselectivity (determined by integration of the methyl ester signals). Purification *via* column chromatography (20:1 to 10:1 hexanes:EtOAc) afforded the title compound (42.3 mg, 86%, 99% ee) as a clear, yellow oil in *ca.* 92% purity with traces of regioisomeric byproducts. **R$_f$** = 0.31 (10:1 hexanes:EtOAc). **^{1}H NMR** (400 MHz, $CDCl_3$) δ 7.40-7.36 (m, 2H), 7.29-7.26 (m, 3H), 3.72 (s, 3H), 3.12-3.05 (m, 1H), 2.63 (dd, *J* = 15.3, 7.6 Hz, 1H), 2.52 (dd, *J* = 15.3, 7.1 Hz, 1H), 1.65-1.47 (m, 4H), 0.96 (t, *J* = 7.1 Hz, 3H). **^{13}C NMR** (100 MHz, $CDCl_3$):δ 172.3, 131.8, 128.3, 127.8, 123.7, 91.5, 82.2, 51.9, 40.2, 36.9, 28.8, 20.6, 14.0. **IR** (film): 2916, 2832, 1717, 1576, 1470, 1418, 1343, 1192, 1151, 1084, 1055, 1005, 902, 747 cm^{-1}. **HRMS:** Calculated for $C_{15}H_{18}NaO_2$ $(M+Na)^+$: 253.1199; Found 253.1203. **Chiral HPLC:** Chiralpak® IB column; heptane/isopropanol 500:1; flow rate = 0.8 mL/min; detection at 220 nm; t_1= 9.6 min (major); t_2 = 13.6 min (minor). **[α]$_D^{25}$** = + 14.9° (*c* = 0.50, $CHCl_3$).

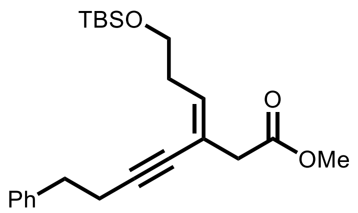

methyl *(Z)*-3-(3-((*tert*-butyldimethylsilyl)oxy)propylidene)-7-phenylhept-4-ynoate (**20**)

Prepared according to Representative Procedure B using $Cu(OAc)_2$•H_2O (2.0 mg, 0.01 mmol, 5 mol %), WALPHOS ligand **17** (9.3 mg, 0.01 mmol, 5 mol %), diethoxymethylsilane (64 µL, 0.40 mmol), *tert*-butanol (38 µL, 0.40 mmol), and methyl *(E)*-3-(*(E)*-3-((*tert*-butyldimethylsilyl)oxy)prop-1-en-1-yl)-7-phenylhept-2-en-4-ynoate (76.9 mg, 0.20 mmol) in PhMe (1.0 mL). The reaction duration was 16 h. Purification *via* column chromatography (20:1 to 10:1 hexanes:EtOAc) afforded the title compound (69.3 mg, 90%, exclusively as its (*Z*) olefin isomer) as a clear oil. **R_f** = 0.28 (10:1 hexanes:EtOAc). **^{1}H NMR** (400 MHz; $CDCl_3$): δ 7.31-7.27 (m, 2H), 7.24-7.19 (m, 3H), 5.79 (t, *J* = 7.3 Hz, 1H), 3.69 (s, 3H), 3.63 (t, *J* = 6.7 Hz, 2H), 3.10 (d, *J* = 0.9 Hz, 2H), 2.85 (t, *J* = 7.5 Hz, 2H), 2.63 (t, *J* = 7.5 Hz, 2H), 2.45 (q, *J* = 7.0 Hz, 2H), 0.90 (s, 9H), 0.06 (s, 6H). **^{13}C NMR** (100 MHz; $CDCl_3$): 171.5, 140.8, 136.9, 128.6, 128.5, 126.4, 117.4, 94.3, 78.8, 62.3, 52.1, 42.7, 35.3, 34.4, 26.1, 21.9, 18.5, -5.1. **IR** (film): 2986, 2911, 1721, 1415, 1238, 1143, 1084, 993, 924, 825, 766, 690 cm^{-1}. **HRMS** (ESI+): Calculated for $C_{23}H_{35}O_3Si$ $(M+H)^+$: 387.2350; Found 387.2346.
Determination of olefin geometry:

The product is assigned as its (*Z*)-isomer on the basis of an observed nOe between the α-methylene (δ 3.10) and the vinyl hydrogen (δ 5.79): irradiation of the methylene resulted in a + 5.3% enhancement at the vinylic hydrogen.

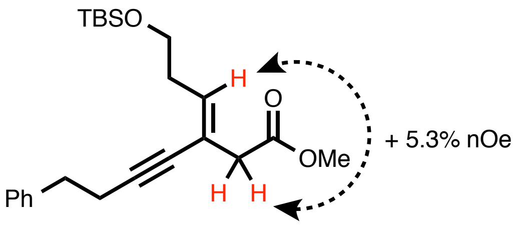

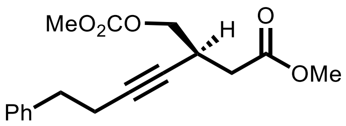

methyl *(S)*-3-(((methoxycarbonyl)oxy)methyl)-7-phenylhept-4-ynoate (**9aj**):

Prepared according to Representative Procedure B using $Cu(OAc)_2•H_2O$ (5.0 mg, 0.025 mmol, 5 mol %), WALPHOS ligand **17** (23.1 mg, 0.025 mmol, 5 mol %), diethoxymethylsilane (159 µL, 1.0 mmol), *tert*-butanol (95 µL, 1.0 mmol), and methyl *(Z)*-3-(((methoxycarbonyl)oxy)methyl)-7-phenylhept-2-en-4-ynoate (150.0 mg, 0.50 mmol) in PhMe (2.5 mL). The reaction duration was 20 h. The crude product was purified by column chromatography (6:1 hexanes:EtOAc) to afford the title compound as a light yellow oil (148 mg, 0.49 mmol, 98% yield, 99% ee). $\mathbf{R_f}$ = 0.33 (6:1 hexanes:EtOAc). **^{1}H NMR** (400 MHz, $CDCl_3$) δ 7.31-7.27 (m, 2H), 7.22-7.18 (m, 3H), 4.24-4.20 (dd, *J* = 10.5 Hz, 5.5 Hz, 1H,), 4.09-4.05 (dd, *J* = 10.5 Hz, 7.5 Hz, 1H), 3.79 (s, 3H), 3.69 (s, 3H), 3.23-3.17 (m, 1H), 2.77 (t, *J* = 7.5 Hz, 2H), 2.61-2.55 (dd, *J* = 15.8 Hz, 6.5 Hz, 1H), 2.52-2.46 (dd, *J* = 7.9 Hz, 1H), 2.45-2.40 (td, *J* = 7.5 Hz, 2.2 Hz, 2H). **^{13}C NMR** (125 MHz, $CDCl_3$) δ 171.6, 155.5, 140.7, 128.6, 128.4, 126.3, 82.9, 78.3, 69.1, 55.0, 52.0, 36.8, 35.2, 28.3, 21.0. **IR** (film): 3028, 2955, 2856, 1750, 1441, 1385, 1365, 1271, 1171, 971, 791, 700 cm^{-1}. **HRMS**: Calculated for $C_{17}H_{20}O_5Na$ $(M+Na)^+$: 327.1208; Found 327.1213. **Chiral HPLC:** Chiralpak® IB column; heptane/isopropanol 99:1; flow rate = 0.8 mL/min; detection at 214 nm; t_1= 16.8 min (major); t_2 = 19.3 min (minor). $[\alpha]_D^{25}$ = + 9.25° (*c* = 1.3, CH_2Cl_2, 99% ee).

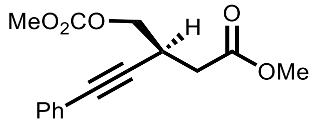

methyl *(S)*-3-(((methoxycarbonyl)oxy)methyl)-5-phenylpent-4-ynoate (**9bj**):

Prepared according to Representative Procedure B using $Cu(OAc)_2•H_2O$ (1.4 mg, 0.007 mmol, 5 mol %), WALPHOS ligand **17** (6.6 mg, 0.007 mmol, 5 mol %), diethoxymethylsilane (45 µL, 0.28 mmol), *tert*-butanol (27 µL, 0.28 mmol), and methyl *(Z)*-3-(((methoxycarbonyl)oxy)methyl)-5-phenylpent-2-en-4-ynoate (39.0 mg, 0.14 mmol) in PhMe (0.70 mL). The reaction duration was 16 h. The crude product was purified by column chromatography (4:1 hexanes:EtOAc) to afford the title compound as a light yellow oil (36.3 mg, 0.13 mmol, 92% yield, 99% ee). $\mathbf{R_f}$ = 0.40 (4:1 hexanes:EtOAc). **^{1}H NMR** (500 MHz, $CDCl_3$) δ 7.39-7.38 (m, 2H), 7.31-7.27 (m, 3H), 4.39-4.35 (dd, *J* = 10.6 Hz, 5.5 Hz, 1H),

4.25-4.22 (dd, J = 10.6 Hz, 7.4 Hz, 1H), 3.80 (s, 3H), 3.72 (s, 3H), 3.49-3.45 (m, 1H), 2.74-2.69 (dd, J = 15.9 Hz, 6.7 Hz, 1H), 2.69-2.64 (dd, J = 15.9 Hz, 7.5 Hz, 1H). **^{13}C NMR** (125 MHz, $CDCl_3$) δ 171.5, 155.7, 132.0, 128.5, 123.0, 87.0, 83.6, 68.6, 55.2, 52.3, 52.0, 36.7, 29.2. **IR** (film): 2957, 1752, 1491, 1442, 1386, 1271, 1171, 969, 895, 790, 759, 693 cm^{-1}. **HRMS:** Calculated for $C_{15}H_{16}O_5Na$ $(M+Na)^+$: 299.0895; Found 299.0902. **Chiral HPLC:** Chiralpak® IB column; heptane/isopropanol 99:1; flow rate = 0.8 mL/min; detection at 214 nm; t_1= 13.9 min (major); t_2 = 16.2 min (minor). **$[\alpha]_D^{25}$** = + 17.16° (c = 0.91, CH_2Cl_2, 99% ee).

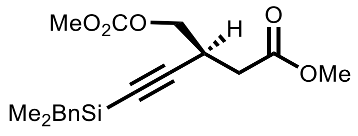

methyl *(S)*-5-(benzyldimethylsilyl)-3-(((methoxycarbonyl)oxy)methyl)pent-4-ynoate (**9ej**):

Prepared according to Representative Procedure B using $Cu(OAc)_2$•H_2O (1.4 mg, 0.007 mmol, 5 mol %), WALPHOS ligand **17** (6.6 mg, 0.007 mmol, 5 mol %), diethoxymethylsilane (45 μL, 0.28 mmol), *tert*-butanol (27 μL, 0.28 mmol), and methyl *(Z)*-5-(benzyldimethylsilyl)-3-(((methoxycarbonyl)oxy)methyl)pent-2-en-4-ynoate (48.0 mg, 0.14 mmol) in PhMe (0.70 mL). The reaction duration was 16 h. The crude product was purified by column chromatography (6:1 hexanes:EtOAc) to afford the title compound as a brown oil (39.3 mg, 0.11 mmol, 81% yield, 99% ee). **R_f** = 0.33 (4:1 hexanes:EtOAc). **^{1}H NMR** (500 MHz, $CDCl_3$) δ 7.23-7.20 (m, 2H), 7.10-7.04 (m, 3H), 4.28-4.25 (dd, J = 10.6 Hz, 5.4 Hz, 1H), 4.14-4.10 (dd, J = 10.6 Hz, 7.5 Hz, 1H), 3.80 (s, 3H), 3.70 (s, 3H), 3.29-3.24 (m, 1H), 2.64-2.59 (dd, J = 15.9 Hz, 6.8 Hz, 1H), 2.57-2.53 (dd, J = 15.9 Hz, 7.3 Hz, 1H), 2.16 (s, 2H), 0.10 (s, 6H). **^{13}C NMR** (125 MHz, $CDCl_3$) δ 171.3, 155.5, 139.0, 128.4, 128.2, 124.4, 104.9, 86.5, 68.5, 55.1, 52.0, 36.4, 29.2, 26.3, -2.0. **IR** (film): 3025, 2958, 2174, 1752, 1600, 1494, 1441, 1385, 1364, 1271, 1208, 1171, 1057, 972, 840, 794, 764, 700 cm^{-1}. **HRMS**: Calculated for $C_{18}H_{24}O_5NaSi$ $(M+Na)^+$: 371.1285; Found 371.1291. **Chiral HPLC:** Chiralpak® IB column; heptane/isopropanol 99:1; flow rate = 0.8 mL/min; detection at 214 nm; t_1= 9.7 min (major); t_2 = 11.7 min (minor). **$[\alpha]_D^{25}$** = +11.86° (c = 1.17, CH_2Cl_2, 99% ee).

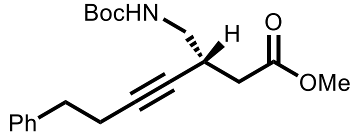

Cu-catalyzed Asymmetric Conjugate Reduction of Carbamates: Representative Procedure C, Synthesis of methyl *(R)*-3-(((*tert*-butoxycarbonyl)amino)methyl)-7-phenylhept-4-ynoate (**9ak**):

An oven-dried Biotage® microwave vial (0.50 – 2.0 mL size) equipped with a stir bar was charged with $Cu(OAc)_2$•H_2O (0.9 mg, 0.0045 mmol, 2 mol %) and JOSIPHOS ligand J002-1 (**13**, (*R*)-1-[(*S*)-2-(diphenylphosphino)ferrocenyl]-ethyl-di-*tert*-butylphosphine, 2.5 mg, 0.0046 mmol, 2 mol %), sealed with a septum, purged with argon, then kept under an argon atmosphere (balloon). Distilled, degassed (N_2 sparged) PhMe (0.92 mL) was added *via* syringe, and the mixture was stirred for 30 min. Diethoxymethylsilane (56 µL, 0.35 mmol, 1.5 equivalents) was added *via* syringe, and the mixture was stirred for 30 min. The vial was cooled to 0 °C, and then a solution of methyl *(Z)*-3-(((*tert*-butoxycarbonyl)amino)methyl)-7-phenylhept-2-en-4-ynoate (79.0 mg, 0.23 mmol, 1.0 equivalent) in THF (0.23 mL) was cannulated into the reaction mixture. *tert*-Butanol (33 µL, 0.35 mmol, 1.5 equivalents) was added, and the vial was placed in a cold room (4 °C) and stirred for 4 h. The crude reaction mixture was then warmed to room temperature, filtered through Florisil (3.5 cm, eluting with 2:1 hexanes:EtOAc, 3 x 3 mL), and the resulting solution was concentrated *in vacuo*. The crude product was purified by column chromatography (6:1 Hexanes:EtOAc) to afford the title compound as a viscous oil (71.5 mg, 90% yield, 94% ee). **R*f*** = 0.17 (6:1 hexanes:EtOAc). **^{1}H NMR** (500 MHz, $CDCl_3$) δ 7.32-7.29 (m, 2H), 7.23-7.20 (m, 3H), 4.67-4.45 (br s, 1H), 3.68 (s, 3H), 3.25-3.20 (m, 1H), 3.16-3.11 (m, 1H), 2.99-2.96 (m, 1H), 2.78 (t, *J* = 7.3 Hz, 2H), 2.47-2.43 (m, 4H), 1.44 (s, 9H). **^{13}C NMR** (125 MHz, $CDCl_3$) δ 171.9, 156.0, 140.8, 128.6, 128.5, 126.4, 82.5, 80.4, 79.5, 51.9, 44.2, 37.4, 35.2, 29.5, 28.5, 21.0. **IR** (film): 3374, 3027, 2977, 2931, 1740, 1716, 1512, 1454, 1437, 1392, 1366, 1252, 1168, 700 cm^{-1}. **HRMS:** Calculated for $C_{20}H_{27}NO_4Na$ $(M+Na)^+$: 368.1838; Found 368.1844. **Chiral HPLC:** Chiralpak® IB column; heptane/isopropanol 99:1; flow rate = 0.8 mL/min; detection

at 214 nm; t_1 = 24.7 min (minor); t_2= 31.0 min (major). **$[\alpha]_D^{25}$** = – 2.29° (*c* = 0.83, CH_2Cl_2, 94% ee).

One-pot synthesis:

An oven-dried Biotage® microwave vial (2.0 – 5.0 mL size) equipped with a stir bar was charged with $Pd(OAc)_2$ (1.7 mg, 0.0076 mmol, 1.5 mol %), tris(2,6-dimethoxyphenyl)phosphine (TDMPP) (3.3 mg, 0.0075 mmol, 1.5 mol %), and methyl *(Z)*-3-(((*tert*-butoxycarbonyl)amino)methyl)-7-phenylhept-2-en-4-ynoate (128.0 mg, 0.60 mmol, 1.2 equivalents). The vial was capped with a rubber septum. PhMe (0.5 mL) was added *via* syringe, and the mixture was stirred at room temperature for 20 min. 4-Phenyl-1-butyne (65.0 mg, 0.50 mmol, 1.0 equivalent) was then added *via* syringe, and the mixture was stirred at room temperature for 24 h. The reaction tube was then purged with N_2 and cooled to 0 ˚C. A pre-formed solution of $Cu(OAc)_2{\cdot}H_2O$/JOSIPHOS J002-1 ligand (**13**), prepared by mixing 5.0 mg of $Cu(OAc)_2{\cdot}H_2O$ (0.025 mmol, 5.0 mol %) and 13.6 mg of ligand (0.025 mmol, 5.0 mol %) in PhMe:THF (4:1, 1.0 mL) for 30 min, was then added *via* cannula. Diethoxymethylsilane (0.24 mL, 1.50 mmol, 3 equivalents) was added, followed by *tert*-butanol (95 μL, 1.0 mmol, 2 equivalents). The mixture was stirred under N_2 at 0 ˚C until the reaction was complete by TLC (3-6 hrs). The crude reaction mixture was then warmed to room temperature, loaded directly onto a silica gel column, and purified by column chromatography (19:1 to 7:3 hexanes:EtOAc) to afford methyl *(R)*-3-(((*tert*-butoxycarbonyl)amino)methyl)-7-phenylhept-4-ynoate as a viscous oil (134 mg, 0.39 mmol, 78% yield, 92% ee). Spectral data matched that of the previously reported product (*vide supra*).

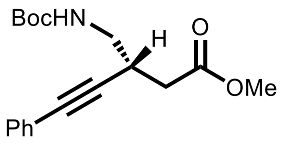

methyl *(R)*-3-(((*tert*-butoxycarbonyl)amino)methyl)-5-phenylpent-4-ynoate (**9bk**):

Prepared according to Representative Procedure C using $Cu(OAc)_2•H_2O$ (2.0 mg, 0.010 mmol, 2 mol %), JOSIPHOS ligand **13** (5.6 mg, 0.010 mmol, 2 mol %), diethoxymethylsilane (121 µL, 0.75 mmol), *tert*-butanol (72 µL, 0.75 mmol), methyl *(Z)*-3-(((*tert*-butoxycarbonyl)amino)methyl)-5-phenylpent-2-en-4-ynoate (158 mg, 0.50 mmol), PhMe (2.0 mL), and THF (0.5 mL). The reaction duration was 4 h. The crude product was purified by column chromatography (19:1 hexanes:EtOAc to 7:3 hexanes:EtOAc) to afford the title compound as a white solid (149 mg, 0.47 mmol, 94% yield, 92% ee). $\mathbf{R}_f$ = 0.22 (4:1 hexanes:EtOAc). **M.P.** = 69 – 70 °C. **^{1}H NMR** (500 MHz, $CDCl_3$) δ 7.42-7.38 (m, 2H), 7.32-7.28 (m, 3H), 4.95-4.55 (N-H, br, 1H), 3.73 (s, 3H), 3.37 (m, 2H), 3.29 (m, 1H), 2.61, (d, J = 7.1 Hz, 2H), 1.46 (s, 9H). **^{13}C NMR** (125 MHz, $CDCl_3$) δ 171.8, 156.0, 131.9, 128.4, 128.3, 123.1, 89.0, 83.3, 79.8, 52.1, 44.1, 37.3, 30.2, 28.6. **IR** (film) = 3359, 2974, 2930, 1727, 1693, 1519, 1438, 1365, 1268, 1250, 1166 cm^{-1}. **HRMS**: Calculated for $C_{18}H_{23}NO_4Na$ $(M+Na)^+$: 340.1525; Found 340.1523. **Chiral HPLC:** Chiralpak® IB column; heptane/isopropanol 99:1; flow rate = 0.8 mL/min; detection at 254 nm; t_1 = 18.5 min (minor); t_2= 21.9 min (major). $[\alpha]_D^{24}$ = -3.95° (c = 0.36, CH_2Cl_2, 92% ee).

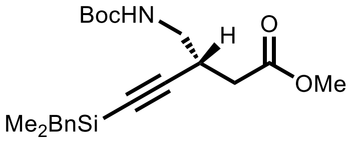

methyl *(R)*-5-(benzyldimethylsilyl)-3-(((*tert*-butoxycarbonyl)amino)methyl)pent-4-ynoate (**9ek**):

Prepared according to Representative Procedure C using $Cu(OAc)_2•H_2O$ (0.8 mg, 0.004 mmol, 2 mol %), JOSIPHOS ligand **13** (2.2 mg, 0.004 mmol, 2 mol %), diethoxymethylsilane (48 µL, 0.30 mmol), *tert*-butanol (29 µL, 0.30 mmol), and methyl *(Z)*-5-(benzyldimethylsilyl)-3-(((*tert*-butoxycarbonyl)amino)methyl)pent-2-en-4-ynoate (77.0 mg, 0.20 mmol), PhMe (0.8 mL), and THF (0.2 mL). The reaction duration was 4 h. The crude product was purified by column chromatography (4:1 hexanes:EtOAc) to afford the title compound as a light yellow oil (66.1 mg, 0.17 mmol, 85% yield, 97% ee). $\mathbf{R}_f$ = 0.20 (6:1 hexanes:EtOAc). **^{1}H NMR** (500 MHz, $CDCl_3$) δ 7.25-7.21 (m, 2H), 7.09-7.05 (m, 3H), 4.76-

4.54 (br s, 1H), 3.69 (s, 3H), 3.29-3.18 (m, 2H), 3.08-3.02 (m, 1H), 2.49 (d, J = 7.2 Hz, 2H), 2.16 (s, 2H), 1.46 (s, 9H), 0.11 (s, 6H). **^{13}C NMR** (125 MHz, $CDCl_3$) δ 171.5, 155.9, 139.1, 128.4, 128.3, 124.4, 107.4, 85.9, 79.6, 51.9, 43.7, 37.0, 30.3, 28.5, 26.4, -1.9. **IR** (film): 3382, 3025, 2976, 2173, 1741, 1718, 1600, 1510, 1453, 1438, 1392, 1366, 1251, 1208, 1168, 1057, 1009, 971, 840, 797, 763, 700 cm^{-1}. **HRMS:** Calculated for $C_{21}H_{31}NO_4NaSi$ $(M+Na)^+$: 412.1920; Found 412.1917 **Chiral HPLC:** Chiralpak® IB column; heptane/isopropanol 99:1; flow rate = 0.8 mL/min; detection at 214 nm; t_1 = 12.4 min (minor); t_2= 13.2 min (major). $[\alpha]_D^{24}$ = – 1.28° (c = 1.41, CH_2Cl_2, 97% ee)

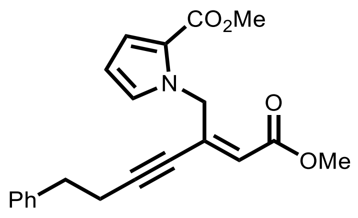

methyl *(Z)*-1-(2-(2-methoxy-2-oxoethylidene)-6-phenylhex-3-yn-1-yl)-1H-pyrrole-2-carboxylate (**22a**):

An oven-dried Biotage® microwave vial (2.0 – 5.0 mL size) equipped with a stir bar was charged with $[(\eta^3\text{-}C_3H_5)PdCl]_2$ (1.8 mg, 0.005 mmol, 2.5 mol %), dppf (8.3 mg, 0.015 mmol, 7.5 mol %), methyl 2-pyrrolecarboxylate (75.1 mg, 0.60 mmol, 3.0 equivalents), and Cs_2CO_3 (71.7 mg, 0.22 mmol, 1.1 equivalents). The vial was sealed with a septum cap and flushed with N_2. Freshly distilled 1,2-dichloroethane (1.0 mL) was added, and the mixture was stirred for 15 minutes. To this heterogeneous, orange mixture was added methyl *(Z)*-3-(((methoxycarbonyl)oxy)methyl)-7-phenylhept-2-en-4-ynoate (60.5 mg, 0.20 mmol, 1.0 equivalent) as a solution in 1,2-dichloroethane (200 µL + 50 µL rinse), which was prepared in a flame-dried ½-dram vial. The reaction mixture was stirred *vigorously* for 6 h, during which time its appearance changed from heterogeneous and orange to heterogeneous and brown. After this time, the reaction mixture was poured into a mixture of saturated aqueous $NaHCO_3$ (10 mL) and Et_2O (10 mL). The phases were separated, and the aqueous phase was extracted with Et_2O (2 x 10 mL). The pooled organic phases were dried over $MgSO_4$, filtered, and concentrated. ^{1}H NMR analysis of the crude mixture indicated a 9:1 mixture of olefin

isomers. The major isomer was isolated by column chromatography (8:1 hexanes:EtOAc), which provided the title compound (47.6 mg, 68%) as a tan solid. **R*f*** = 0.38 (4:1 hexanes:EtOAc). **M. P.** = 65–67 °C. **^{1}H NMR** (400 MHz; $CDCl_3$): δ 7.30-7.27 (m, 2H), 7.23-7.21 (m, 1H), 7.11-7.09 (m, 2H), 6.97 (dd, *J* = 3.9, 1.8 Hz, 1H), 6.81 (t, *J* = 2.2 Hz, 1H), 6.15 (dd, *J* = 3.9, 2.6 Hz, 1H), 6.14 (dd, *J* = 3.9, 2.6 Hz, 1H), 6.06 (t, *J* = 1.9 Hz, 1H), 5.61 (d, *J* = 2.0 Hz, 2H), 3.79 (s, 3H), 3.77 (s, 3H), 2.62 (t, *J* = 7.6 Hz, 2H), 2.39 (t, *J* = 7.6 Hz, 2H). **^{13}C NMR** (100 MHz; $CDCl_3$): 166.3, 161.7, 140.4, 140.3, 129.7, 128.5, 128.4, 126.5, 122.8, 122.5, 118.1, 108.3, 98.4, 78.6, 51.7, 51.2, 49.7, 34.4, 21.7. **IR** (film): 2908, 2188, 1685, 1586, 1512, 1417, 1392, 1310, 1231, 1148, 1093, 1064, 1016, 858, 732, 690 cm^{-1}. **HRMS** (ESI+): Calculated for $C_{21}H_{21}NNaO_4$ (M+Na)+: 374.1363; Found 374.1363.

Determination of the olefin geometry:

Irradiation of the vinylic hydrogen (δ 6.06) of the major product isomer led to a very weak nOe enhancement (+ 0.4 %) at the indicated methylene (δ 5.61):

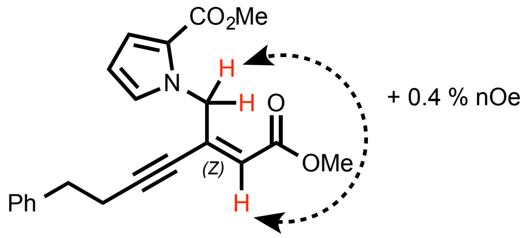

In contrast, irradiation of the vinylic hydrogen (δ 5.04) of the minor product isomer led to a stronger nOe enhancement (+ 3.6%) at the methylene group (δ 5.47). These results suggest (*E*)-geometry for the minor product and (*Z*)-geometry for the major product.

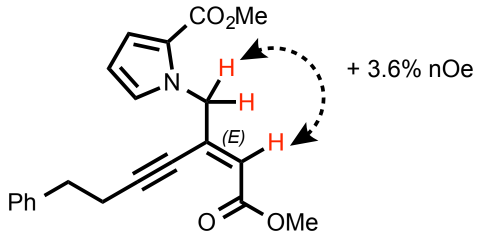

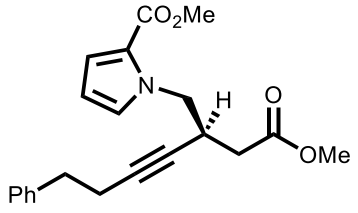

methyl *(S)*-1-(2-(2-methoxy-2-oxoethyl)-6-phenylhex-3-yn-1-yl)-1H-pyrrole-2-carboxylate (**23a**):

An oven-dried Biotage® microwave vial (0.50 – 2.0 mL size) equipped with a stir bar was charged with $Cu(OAc)_2$•H_2O (2.0 mg, 0.010 mmol, 10 mol %) and WALPHOS ligand **17** (9.3 mg, 0.010 mmol, 10 mol %). The vial was sealed with a septum and flushed with Ar (balloon). Freshly distilled, degassed (Ar sparged) PhMe (300 µL) was added, and the mixture was stirred for 20 min to afford a bright green, homogeneous solution. Diethoxymethylsilane (32 µL, 0.20 mmol, 2.0 equivalents) was added, and the mixture was stirred until a change in appearance to homogeneous and brown was observed (*ca.* 15-30 min). The vial was cooled to 0 °C, and methyl *(Z)*-1-(2-(2-methoxy-2-oxoethylidene)-6-phenylhex-3-yn-1-yl)-1H-pyrrole-2-carboxylate (35.1 mg, 0.10 mmol, 1.0 equivalent) was added as a solution in PhMe (200 µL), which was prepared in a flame-dried ½-dram vial. To the mixture was then added *tert*-butanol (19 µL, 0.20 mmol, 2.0 equivalents). The vessel was kept under an Ar balloon and transferred to a cold room (4 °C) where it was stirred for 18 h. After this time, TBAF (1 M in THF, 300 µL, 0.30 mmol, 3.0 equivalents) was added, and the mixture was stirred for 10 minutes. The mixture was then applied to a silica gel column that was eluted with 6:1 to 4:1 hexanes:EtOAc to afford the title compound (29.7 mg, 84%, 99% ee) as a viscous light yellow oil. $\mathbf{R}_f$ = 0.30 (4:1 hexanes:EtOAc). **^{1}H NMR** (600 MHz; $CDCl_3$): δ 7.30-7.27 (m, 2H), 7.22-7.17 (m, 3H), 6.95 (dd, *J* = 3.9, 1.8 Hz, 1H), 6.79 (t, *J* = 2.1 Hz, 1H), 6.08 (dd, *J* = 3.9, 2.6 Hz, 1H), 4.45 (dd, *J* = 13.3, 6.7 Hz, 1H), 4.35 (dd, *J* = 13.3, 7.5 Hz, 1H), 3.80 (s, 3H), 3.66 (s, 3H), 3.32-3.30 (m, 1H), 2.78-2.73 (m, 2H), 2.45-2.37 (m, 4H). **^{13}C NMR** (100 MHz; $CDCl_3$): 171.6, 161.7, 140.8, 130.0, 128.6, 128.4, 126.4, 121.6, 118.7, 108.1, 83.3, 79.6, 52.2, 51.9, 51.2, 37.2, 35.2, 30.7, 21.0. **IR** (film): 2909, 1716, 1861, 1418, 1391, 1310, 1233, 1093, 734, 690 cm^{-1}. **HRMS** (ESI+): Calculated for $C_{21}H_{23}NNaO_4$ $(M+Na)^+$: 376.1519; Found

376.1515. **Chiral HPLC**: Agilent IB, 98:2 heptane:isopropanol, 0.8 mL/min, 254 nm, 13.40 min (major), 14.74 min (minor). $[\alpha]_D^{25}$ = – 19.3 ° (c = 0.5, $CHCl_3$).

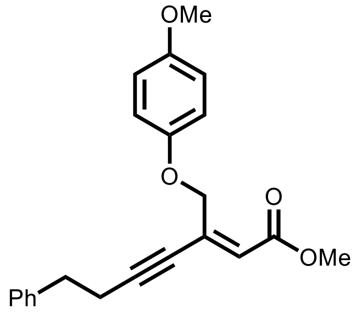

methyl *(Z)*-3-((4-methoxyphenoxy)methyl)-7-phenylhept-2-en-4-ynoate (**22b**):

An oven-dried Biotage® microwave vial (2.0 – 5.0 mL size) equipped with a stir bar was charged with $[(\eta^3\text{-}C_3H_5)PdCl]_2$ (1.8 mg, 0.005 mmol, 2.5 mol %), dppf (8.3 mg, 0.015 mmol, 7.5 mol %), 4-methoxyphenol (74.5 mg, 0.60 mmol, 3.0 equivalents), and Cs_2CO_3 (71.7 mg, 0.22 mmol, 1.1 equivalents). The vial was sealed with a septum cap and flushed with N_2. Freshly distilled 1,2-dichloroethane (1.0 mL) was added, and the mixture was stirred for 15 minutes. To this heterogeneous, orange mixture was added methyl *(Z)*-3-(((methoxycarbonyl)oxy)methyl)-7-phenylhept-2-en-4-ynoate (60.5 mg, 0.20 mmol, 1.0 equivalent) as a solution in 1,2-dichloroethane (200 µL + 50 µL rinse), which was prepared in a flame-dried ½-dram vial. The reaction mixture was stirred *vigorously* for 6 h. After this time, the reaction mixture was poured into a mixture of saturated aqueous $NaHCO_3$ (10 mL) and Et_2O (10 mL). The phases were separated, and the aqueous phase was extracted with Et_2O (2 x 10 mL). The pooled organic phases were dried over $MgSO_4$, filtered, and concentrated. ^{1}H NMR analysis of the crude mixture indicated a single olefin isomer. Column chromatography (8:1 hexanes:EtOAc) delivered the title compound (48.2 mg, 69%) as a viscous, light yellow oil. The product was assigned as the (*Z*)-isomer by analogy to the preceding Pd-AA reaction; in support of this, no detectable nOe enhancement (< 0.1 %) was observed between the vinylic hydrogen (δ 6.12) and the allylic hydrogens (δ 5.11). $\mathbf{R_f}$ = 0.38 (6:1 hexanes:EtOAc). **^{1}H NMR** (400 MHz; $CDCl_3$): δ 7.30-7.18 (m, 5H), 6.90-6.80 (m, 4H), 6.12 (t, J = 1.7 Hz, 1H), 5.11 (d, J = 1.8 Hz, 2H), 3.77 (s, 3H), 3.74 (s, 3H), 2.80 (t, J = 7.4

Hz, 2H), 2.63 (t, *J* = 7.4 Hz, 2H). **^{13}C NMR** (100 MHz; $CDCl_3$): 166.2, 154.1, 152.7, 140.5, 140.4, 128.6, 128.5, 126.5, 124.2, 116.1, 114.6, 98.8, 79.9, 66.5, 55.8, 51.7, 34.5, 22.1. **IR** (film): 2985, 2908, 2794, 2189, 1688, 1584, 1486, 1415, 1344, 1318, 1215, 1148, 1092, 1023, 861, 814 cm^{-1}. **HRMS** (ESI+): Calculated for $C_{22}H_{22}NaO_4$ $(M+Na)^+$: 373.1410; Found 373.1409.

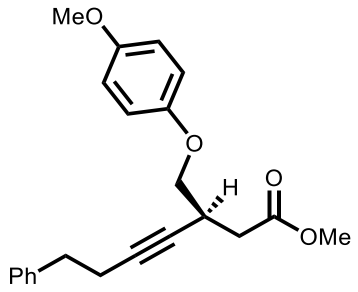

methyl *(S)*-3-((4-methoxyphenoxy)methyl)-7-phenylhept-4-ynoate (**23b**):

An oven-dried Biotage® microwave vial (0.50 – 2.0 mL size) equipped with a stir bar was charged with $Cu(OAc)_2 \bullet H_2O$ (1.0 mg, 0.005 mmol, 5 mol %) and WALPHOS ligand **17** (4.6 mg, 0.005 mmol, 5 mol %). The vial was sealed with a septum and flushed with Ar (balloon). Freshly distilled, degassed (Ar sparged) PhMe (300 µL) was added, and the mixture was stirred for 20 min to afford a bright green, homogeneous solution. Diethoxymethylsilane (32 µL, 0.20 mmol, 2.0 equivalents) was added, and the mixture was stirred until a change in appearance to homogeneous and brown was observed (*ca.* 15-30 min). The vial was cooled to 0 °C, and methyl *(Z)*-3-((4-methoxyphenoxy)methyl)-7-phenylhept-2-en-4-ynoate (35.0 mg, 0.10 mmol, 1.0 equivalent) was added as a solution in PhMe (200 µL), which was prepared in a flame-dried ½-dram vial. To the mixture was then added *tert*-butanol (19 µL, 0.20 mmol, 2.0 equivalents). The vessel was kept under an Ar balloon and transferred to a cold room (4 °C) where it was stirred for 24 h. After this time, TBAF (1 M in THF, 300 µL, 0.30 mmol, 3.0 equivalents) was added, and the mixture was stirred for 10 minutes. The mixture was then applied to a silica gel column which was eluted with 10:1 to 8:1 hexanes:EtOAc to afford the title compound (30.3 mg, 86%, 99% ee) as a viscous yellow oil. $\mathbf{R_f}$ = 0.30 (6:1 hexanes:EtOAc). **^{1}H NMR** (400 MHz; $CDCl_3$): δ 7.30-7.26 (m, 2H), 7.22-7.20 (m, 3H), 6.83

(s, 4H), 4.01 (dd, J = 9.2, 5.0 Hz, 1H), 3.84 (dd, J = 9.1, 8.2 Hz, 1H), 3.77 (s, 3H), 3.70 (s, 3H), 3.32-3.24 (m, 1H), 2.81-2.72 (m, 3H), 2.53 (dd, J = 15.7, 8.0 Hz, 1H), 2.45 (td, J = 7.5, 2.2 Hz, 2H). **^{13}C NMR** (100 MHz; $CDCl_3$): δ 172.1, 154.2, 152.7, 140.8, 128.6, 128.4, 126.3, 115.8, 114.7, 82.5, 79.4, 70.5, 55.9, 51.9, 37.1, 35.3, 28.9, 21.1. **IR** (film): 2985, 2908, 1715, 1487, 1418, 1344, 1214, 1153, 1092, 1028, 814, 739, 690 cm^{-1}. **HRMS** (ESI+): Calculated for $C_{22}H_{25}O_4$ $(M+H)^+$: 353.1747; Found 353.1743. **Chiral HPLC**: Agilent IB, 95:5 heptane:isopropanol, 0.8 mL/min, 220 nm, 16.22 min (major), 22.24 min (minor). **$[\alpha]_D^{25}$** = + 6.9° (c = 0.5, $CHCl_3$).

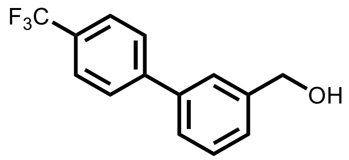

(4'-(trifluoromethyl)-[1,1'-biphenyl]-3-yl)methanol

A flame-dried 100 mL round-bottomed flask equipped with a stir bar was charged with Pd(dppf)Cl_2•CH_2Cl_2 (408.3 mg, 0.50 mmol, 5 mol %) and 3-(hydroxymethyl)phenylboronic acid (1.90 g, 12.5 mmol, 1.25 equivalents). The flask was sealed with a septum and flushed with Ar, and then degassed (Ar sparged) DMF (10 mL) and THF (freshly distilled, 10 mL) were added. Cs_2CO_3 (6.52 g, 20.0 mmol, 2.0 equivalents) was added under Ar flow, and then 4-bromobenzotrifluoride (1.40 mL, 10.0 mmol, 1.0 equivalent) was added *via* syringe. The vessel was heated to 90 °C, and then it was sealed with a yellow plastic cap. The reaction mixture was stirred for 18 h. The vessel was cooled to room temperature, and the reaction mixture was poured into Et_2O (200 mL). The organic phase was washed with H_2O (2 x 100 mL), 10% aqueous LiCl (50 mL), and brine (50 mL). The organic phase was dried over $MgSO_4$, filtered, and concentrated. Purification of the residue by column chromatography (2:1 to 1:1 hexanes:EtOAc) afforded the title compound (2.32 g, 92%) as a viscous, brown oil that solidified on standing, yielding an off-white solid. **R_f** = 0.30 (2:1 hexanes:EtOAc). **^{1}H NMR** (600 MHz; $CDCl_3$): δ 7.72-7.69 (m, 4H), 7.62 (s, 1H), 7.54-7.52 (m, 1H), 7.47 (t, J = 7.6 Hz, 1H), 7.42-7.40 (m, 1H), 4.79 (d, J = 4.9 Hz, 2H), 1.74 (s, 1H). *These ^{1}H NMR data matched literature data*.[11]

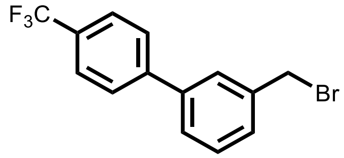

3-(bromomethyl)-4'-(trifluoromethyl)-1,1'-biphenyl (**26**):

A flame-dried 50 mL round-bottomed flask equipped with a stir bar was charged with alcohol (4'-(trifluoromethyl)-[1,1'-biphenyl]-3-yl)methanol (1.73 g, 6.87 mmol, 1.0 equivalent). The flask was sealed with a septum and flushed with N_2, and then PhMe (13.7 mL) was added followed by Et_3N (96 µL, 0.69 mmol, 10 mol %). The vessel was heated to 35 °C. Neat $SOBr_2$ (0.60 mL, 7.72 mmol, 1.125 equivalents) was added *via* syringe pump over 2 h. The reaction mixture was stirred for an additional 0.5 h, and then it was cooled to room temperature. H_2O (15 mL) was added *cautiously* in portions, and then the mixture was poured into Et_2O (50 mL). The phases were separated, and the organic phase was washed with saturated aqueous $NaHCO_3$ (2 x 50 mL). The organic phase was dried over $MgSO_4$, filtered, and concentrated. The residue was purified by column chromatography (10:1 hexanes:EtOAc) to afford the title compound (1.98 g, 92%) as a clear, colorless oil that solidified upon standing in a refrigerator, yielding a white solid. $\mathbf{R}_f$ = 0.53 (10:1 hexanes:EtOAc). M. P. = 50–52 °C (lit. M. P. = 60–62 °C)[12] **^{1}H NMR** (600 MHz; $CDCl_3$): δ 7.72-7.68 (m, 4H), 7.62 (d, *J* = 1.6 Hz, 1H), 7.53 (dt, *J* = 7.1, 1.8 Hz, 1H), 7.47-7.43 (m, 2H), 4.57 (s, 2H).
These ^{1}H NMR data matched literature data.[12]

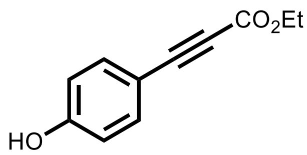

ethyl 3-(4-hydroxyphenyl)propiolate (**25**):

A flame-dried 100 mL round-bottomed flask equipped with a large stir bar was charged with 4-iodophenol (2.20 g, 10.0 mmol, 1.0 equivalent) and Cu_2O (2.146 g, 15.0 mmol, 1.5 equivalents). The flask was sealed with a septum and flushed with N_2. Degassed

(N$_2$ sparged) DMF (20 mL) was added, followed by ethyl propiolate (2.03 mL, 20.0 mmol, 2.0 equivalents). The vessel was heated to 110 °C, and then it was sealed with a yellow plastic cap. The reaction mixture was stirred for 18 h. The vessel was then cooled to room temperature, and the reaction mixture was vacuum filtered through a Celite® pad, which was rinsed with Et_2O (150 mL). The filtrate was washed with H_2O (2 x 50 mL) then 10% aqueous LiCl (50 mL), and then these aqueous phases were pooled and back-extracted with Et_2O (50 mL). The pooled organic phases were washed with brine (50 mL), dried over $MgSO_4$, filtered, and concentrated. The residue was purified by column chromatography (6:1 to 2:1 hexanes:EtOAc) to afford the title compound (1.402 g, 74%) as an orange solid. **R*f*** = 0.33 (2:1 hexanes:EtOAc). M. P. = 83–84 °C. **^{1}H NMR** (400 MHz; $CDCl_3$): δ 7.48-7.45 (m, 2H), 6.85-6.82 (m, 2H), 5.87 (br s, 1H), 4.30 (q, J = 7.1 Hz, 2H), 1.35 (t, J = 7.1 Hz, 3H). **13C NMR** (100 MHz; $CDCl_3$): 158.2, 154.9, 135.3, 116.0, 111.5, 87.5, 80.1, 62.3, 14.2. **IR** (film): 3219 (br), 2178, 1647, 1582, 1492, 1421, 1355, 1298, 1266, 1192, 1149, 1007, 825, 740 cm^{-1}. *These ^{1}H and ^{13}C NMR data matched literature data.*[13]

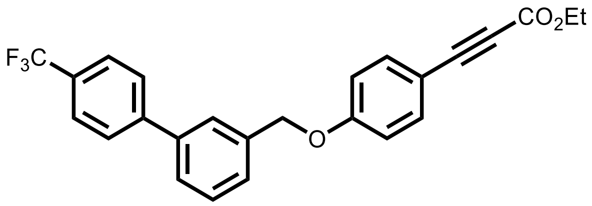

ethyl 3-(4-((4'-(trifluoromethyl)-[1,1'-biphenyl]-3-yl)methoxy)phenyl)propiolate (**27**):

A flame-dried 2-dram vial equipped with a large stir bar was charged with propiolate ethyl 3-(4-hydroxyphenyl)propiolate (285.0 mg, 1.50 mmol, 1.0 equivalent) and DMF (3.0 mL). 3-(Bromomethyl)-4'-(trifluoromethyl)-1,1'-biphenyl (543.0 mg, 1.72 mmol, 1.15 equivalents) was added, followed by K_2CO_3 (414.2 mg, 3.00 mmol, 2.0 equivalents). The heterogeneous reaction mixture was stirred vigorously for 1 h, at which point the reaction mixture was applied directly to a silica gel column that was eluted with 6:1 to 4:1 hexanes:EtOAc to afford the title compound (578.6 mg, 91%) as a white solid. **R*f*** = 0.46 (4:1 hexanes:EtOAc). **M. P.** = 98–100 °C. **^{1}H NMR** (400 MHz; $CDCl_3$): δ 7.70 (s, 4H), 7.66-7.64 (m, 1H), 7.59-7.44 (m, 5H), 7.00-6.96 (m, 2H), 5.16 (s, 2H), 4.29 (q, J = 7.1 Hz, 2H), 1.35 (t,

J = 7.1 Hz, 3H). **^{13}C NMR** (100 MHz; $CDCl_3$): 160.6, 154.4, 144.3 (q, J = 1.2 Hz), 140.4, 137.1, 135.1, 129.7 (q, J = 32.4 Hz), 129.5, 127.6, 127.4, 127.3, 126.5, 125.9 (q, J = 3.6 Hz), 124.3 (q, J = 270.5 Hz), 115.2, 112.0, 86.8, 80.4, 70.0, 62.1, 14.3. **^{19}F NMR** (376 MHz; $CDCl_3$): – 62.7. **IR** (film): 2173, 1678, 1584, 1491, 1316, 1239, 1151, 1105, 1058, 833 cm^{-1}. **HRMS** (ESI+): Calculated for $C_{25}H_{19}F_3NaO_3$ $(M+Na)^+$: 447.1179; Found 447.1172.

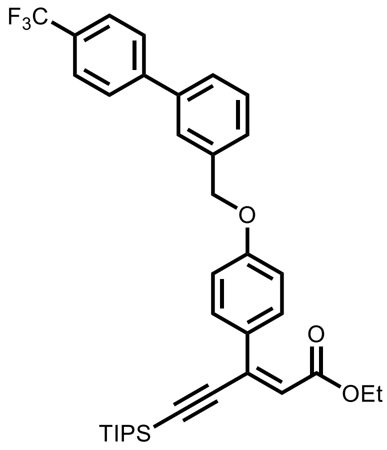

ethyl *(E)*-3-(4-((4'-(trifluoromethyl)-[1,1'-biphenyl]-3-yl)methoxy)phenyl)-5-(triisopropylsilyl)pent-2-en-4-ynoate (**28b**):

A 2-dram vial equipped with a stir bar was charged with $Pd(OAc)_2$ (15.0 mg, 0.067 mmol, 5 mol %) and tris(2,6-dimethoxyphenyl)phosphine (29.6 mg, 0.067 mmol, 5 mol %). THF (1.0 mL) was added, and the mixture was stirred for 15 minutes, generating a homogeneous, orange-red solution. To a separate 2-dram vial containing acceptor ethyl 3-(4-((4'-(trifluoromethyl)-[1,1'-biphenyl]-3-yl)methoxy)phenyl)propiolate (568.4 mg, 1.34 mmol, 1.0 equivalent) was added a stir bar and THF (1.0 mL). The former solution was transferred to the latter by pipette, and then triisopropylsilylacetylene (900 µL, 4.02 mmol, 3.0 equivalents) was added. The homogeneous, black mixture was stirred for 24 h, at which point it was loaded directly onto a silica gel column that was eluted with 20:1 to 10:1 hexanes:EtOAc to afford the title compound (747.8 mg, 92%) as a viscous, red-orange oil. **R_f** = 0.53 (4:1 hexanes:EtOAc). **^{1}H NMR** (600 MHz; $CDCl_3$): δ 7.70 (s, 4H), 7.67-7.66 (m, 1H), 7.57 (dt, J

= 7.4, 1.7 Hz, 1H), 7.52-7.46 (m, 4H), 6.98-6.96 (m, 2H), 6.29 (s, 1H), 5.16 (s, 2H), 4.11 (q, *J* = 7.1 Hz, 2H), 1.19 (t, *J* = 7.1 Hz, 3H), 1.12-1.05 (m, 21H). **^{13}C NMR** (125 MHz; $CDCl_3$): 165.6, 159.5, 144.4 (q, *J* = 1.1 Hz), 140.2, 137.74, 137.70, 130.8, 129.6 (q, *J* = 32.4 Hz), 129.4, 128.7, 127.6, 127.4, 127.1, 126.5, 125.8 (q, *J* = 3.6 Hz), 124.4 (q, *J* = 270.5 Hz), 123.8, 114.0, 107.6, 97.4, 69.9, 60.5, 18.7, 14.2, 11.3. **^{19}F NMR** (376 MHz; $CDCl_3$): – 62.8. **IR** (film): 2902, 2826, 1694, 1583, 1488, 1443, 1308, 1208, 1150, 1058, 1003, 824, 780, 662 cm^{-1}. **HRMS** (ESI+): Calculated for $C_{36}H_{42}F_3O_3Si$ $(M+H)^+$: 607.2850; Found 607.2841.

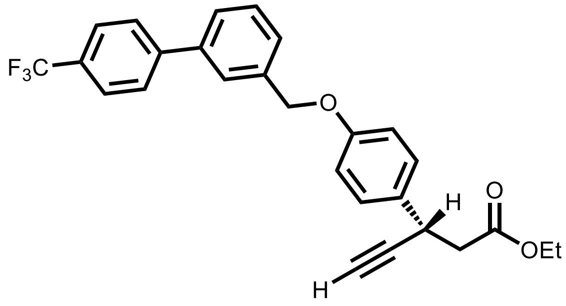

ethyl *(R)*-3-(4-((4'-(trifluoromethyl)-[1,1'-biphenyl]-3-yl)methoxy)phenyl)pent-4-ynoate (**30**):

An oven-dried round-bottom Biotage® microwave vial (2.0 – 5.0 mL size) equipped with a stir bar was charged with $Cu(OAc)_2$•H_2O (7.5 mg, 0.0375 mmol, 5 mol %) and JOSIPHOS ligand **14** ((*R*)-1-[(*S*)-2-(diphenylphosphino)ferrocenyl]-ethyl-di-3,5-xylylphosphine, 24.0 mg, 0.0375 mmol, 5 mol %). The vial was sealed with a septum, flushed with Ar (balloon), and kept under an Ar atmosphere. Distilled, degassed (Ar sparged) PhMe (2.0 mL) was added, and the mixture was stirred for 30 min. Diethoxymethylsilane (0.60 mL, 3.75 mmol, 5 equivalents) was added. The mixture was stirred for 30 min, during which time the original blue-green, homogeneous appearance of the catalyst solution turned to golden yellow and homogeneous. The vessel was cooled to 0 °C. A separate oven-dried round-bottomed Biotage® microwave vial (2.0 – 5.0 mL size) was charged with ethyl *(E)*-3-(4-((4'-(trifluoromethyl)-[1,1'-biphenyl]-3-yl)methoxy)phenyl)-5-(triisopropylsilyl)pent-2-en-4-ynoate (455.1 mg, 0.75 mmol, 1 equivalent), and the vessel was flushed with Ar (balloon). Distilled, degassed PhMe (1.25 mL) was added to dissolve the substrate, and the solution was cooled to 0 °C. The substrate solution was transferred to the catalyst/silane mixture *via*

cannula, using additional PhMe (0.50 mL) to rinse the vial and cannula. *tert*-Butanol (215 μL, 2.25 mmol, 3 equivalents) was added, and the vessel was transferred to a cold room (4 °C) where it was stirred for 24 h.

After this time, a 50 μL aliquot was removed. This sample was diluted with Et_2O and filtered through a small pipette plug of Florisil®. The solution was concentrated, and ^1H NMR analysis of this sample indicated the formation of the corresponding β-alkynyl ester with 96% reaction conversion and with 8:1 1,4:Σ1,6 regioselectivity.

The remainder of the reaction mixture was transferred to a pre-cooled (0 °C), flame-dried 50 mL round-bottom flask *via* cannula, using THF (3.75 mL) to rinse the vial and cannula. To this mixture was added TBAF (1 M in THF, 7.5 mL, 7.5 mmol, 10 equivalents). Gas evolution occurred. The reaction mixture was stirred at 0 °C for 4 h, at which point it was warmed to room temperature and filtered through a column of Florisil® (10 cm by 5 cm), eluting with Et_2O (150 mL). The solution was concentrated, and the residue was purified by column chromatography (10:1 to 6:1 hexanes:EtOAc) to yield the title compound as a single regioisomer and as a white gum (251.3 mg, 74%, 82% ee). $\mathbf{R_f}$ = 0.50 (4:1 hexanes:EtOAc). **^{1}H NMR** (600 MHz; $CDCl_3$): δ 7.70 (s, 4H), 7.67 (s, 1H), 7.57-7.55 (m, 1H), 7.51-7.46 (m, 2H), 7.35-7.32 (m, 2H), 6.98-6.96 (m, 2H), 5.13 (s, 2H), 4.18-4.12 (m, 3H), 2.82 (dd, *J* = 15.4, 8.3 Hz, 1H), 2.71 (dd, *J* = 15.4, 7.0 Hz, 1H), 2.29 (d, *J* = 2.5 Hz, 1H), 1.24-1.22 (m, 3H). **^{13}C NMR** (125 MHz; $CDCl_3$): 170.8, 158.0, 144.4 (q, *J* = 1.4 Hz), 140.2, 137.9, 132.5, 129.5 (q, *J* = 32.4 Hz), 129.3, 128.6, 127.5, 127.3, 127.0, 126.4, 125.8 (q, *J* = 3.9 Hz), 124.4 (q, *J* = 270.5 Hz), 115.0, 84.5, 71.4, 69.9, 60.8, 43.2, 33.2, 14.2. **^{19}F NMR** (376 MHz; $CDCl_3$): – 62.8. **IR** (film): 3251, 2993, 2941, 2895, 1710, 1591, 1563, 1489, 1443, 1383, 1352, 1309, 1228, 1149, 1109, 1057, 1004, 832, 781, 731, 712, 690 cm^{-1}. **HRMS** (ESI+): Calculated for $C_{27}H_{24}F_3O_3$ $(M+H)^+$: 453.1672; Found 453.1662. $\mathbf{[\alpha]_D^{23}}$ = + 3.8 ° (*c* = 0.65, $CHCl_3$)

The ee was determined by converting a sample of the title compound to a mixture of diastereomeric amides *via* amidation with *(S)*-α-methylbenzylamine. To a solution of the title compound (22.6 mg, 0.05 mmol) in EtOH/THF (5:1, 0.60 mL) at 23 °C was added a solution of NaOH (10 mg, 0.25 mmol, 5 equiv) in H_2O (0.10 mL). The mixture was heated to 50 °C for 1 h, at which point it was cooled to room temperature, acidified with 1 N HCl, and

extracted with Et_2O. The extracts were dried over $MgSO_4$, filtered, and concentrated. Flash column chromatography (1:1 hexanes:EtOAc) delivered 9.2 mg of the corresponding acid, which was dissolved in 1,2-dichloroethane (0.25 mL). This solution was treated with triethylamine (15 μL, 0.10 mmol, 5 equivalents), EDCI-HCl (12.5 mg, 0.065 mmol, 3 equivalents), *(S)*-α-methylbenzylamine (28 μL, 0.22 mmol, 10 equivalents), and a single small crystal of DMAP. The reaction mixture was stirred for 5 h at 23 °C, at which point it was diluted with Et_2O and quenched with 1 N HCl. The phases were separated, the aqueous phase was extracted with Et_2O, and the pooled organic phases were dried over $MgSO_4$, filtered, and concentrated. The crude mixture was analyzed by 600 MHz ^{1}H NMR spectroscopy. The dr was determined to be 91:9, corresponding to an ee of 82%. Salient, well-resolved, diastereotopic NMR signals include the terminal alkyne C–H (2.33 ppm, d, J = 2.4 Hz, major; 2.25 ppm, d, J = 2.4, minor) and the methyl group of the amide (1.47 ppm, d, J = 7.2 Hz, major; 1.35 ppm, d, J = 6.6 Hz, minor).

The assignment of these signals was confirmed upon the independent synthesis of a 1:1 mixture of amide diastereomers from a sample of racemic β-aryl, β-alkynyl ester. The latter compound was prepared *via* a conjugate reduction performed using an equimolar mixture of enantiomeric JOSIPHOS ligands.

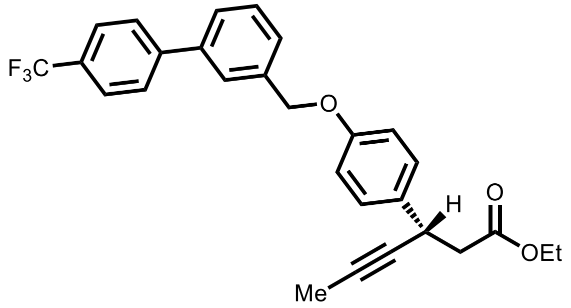

ethyl *(S)*-3-(4-((4'-(trifluoromethyl)-[1,1'-biphenyl]-3-yl)methoxy)phenyl)hex-4-ynoate (**32**):

An oven-dried round bottom Biotage® microwave vial (0.50 – 2.0 mL size) equipped with a stir bar was charged with $[(\eta^3\text{-}C_3H_5)PdCl]_2$ (1.4 mg, 0.0038 mmol, 5 mol % Pd), CuI (2.1 mg, 0.0113 mmol, 7.5 mol %), 1,3-bis-(1-adamantyl)imidazolium chloride (2.8 mg, 0.0075 mmol, 5 mol %), and Cs_2CO_3 (68.5 mg, 0.21 mmol, 1.4 equivalents). The vial was

sealed with a septum and flushed with Ar (balloon). A separate oven-dried round bottom Biotage® microwave vial (0.50 – 2.0 mL size) was charged with ethyl *(R)*-3-(4-((4'-(trifluoromethyl)-[1,1'-biphenyl]-3-yl)methoxy)phenyl)pent-4-ynoate (67.9 mg, 0.15 mmol, 1 equivalent). This vial was sealed with a septum and flushed with Ar (balloon). The substrate was dissolved in degassed (N_2 sparged) Et_2O (0.50 mL), and the solution was transferred to the remaining vial *via* cannula. Degassed (N_2 sparged) DMF (0.25 mL) was added to vial previously containing the substrate and was transferred similarly. Iodomethane (47 μL, 0.75 mmol, 5 equivalents) was added to the reaction mixture. The vial was sealed with a septum-lined metal cap and then heated to 40 °C. The reaction mixture was stirred vigorously at this temperature for 24 h. The appearance was initially brown/black, but this yielded to a yellow color after a few hours. After the 24 h, the vessel was cooled to 23 °C, and the reaction mixture was diluted with Et_2O (3 mL). The mixture was filtered through a pipette plug of Florisil®, which was rinsed with additional Et_2O (10 mL). The clear yellow solution was concentrated, and purification of the residue by column chromatography (4:1 hexanes:EtOAc) delivered the title compound as a viscous yellow oil (41.9 mg, 60%). $\mathbf{R_f}$ = 0.50 (4:1 hexanes:EtOAc). **^{1}H NMR** (400 MHz; $CDCl_3$): δ 7.70 (s, 4H), 7.66 (s, 1H), 7.57-7.55 (m, 1H), 7.51-7.45 (m, 2H), 7.33-7.29 (m, 2H), 6.97-6.93 (m, 2H), 5.12 (s, 2H), 4.16-4.04 (m, 3H), 2.74 (dd, *J* = 15.1, 8.4 Hz, 1H), 2.64 (dd, *J* = 15.1, 7.0 Hz, 1H), 1.83 (d, *J* = 2.4 Hz, 3H), 1.22 (t, *J* = 7.1 Hz, 3H). **^{13}C NMR** (125 MHz; $CDCl_3$): 171.2, 157.8, 144.5 (q, *J* = 1.4 Hz), 140.2, 138.0, 133.8, 129.6 (q, *J* = 32.4 Hz), 129.4, 128.6, 127.6, 127.4, 127.0, 126.4, 125.8 (q, *J* = 3.6 Hz), 124.4 (q, *J* = 270.4 Hz), 115.0, 79.7, 78.9, 70.0, 60.7, 43.7, 33.6, 14.3, 3.8. **^{19}F NMR** (376 MHz; $CDCl_3$): – 62.8. **IR** (film): 2880, 1711, 1590, 1489, 1382, 1352, 1308, 1226, 1148, 1108, 1056, 1003, 832, 780, 690 cm^{-1}. **HRMS** (ESI+): Calculated for $C_{28}H_{26}F_3O_3$ $(M+H)^+$: 467.1829; Found 467.1827. $\mathbf{[\alpha]_D^{23}}$ = + 4.9 ° (*c* = 1.0, $CHCl_3$)

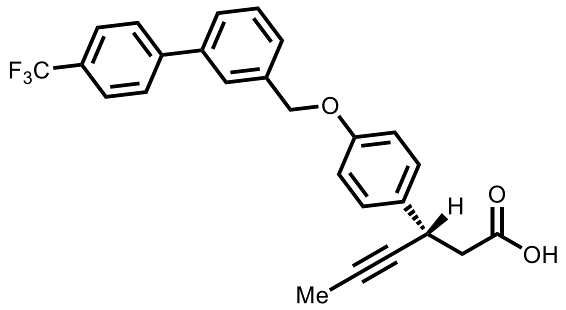

(S)-3-(4-((4'-(trifluoromethyl)-[1,1'-biphenyl]-3-yl)methoxy)phenyl)hex-4-ynoic acid (AMG 837, **24**):

To a solution of ethyl *(S)*-3-(4-((4'-(trifluoromethyl)-[1,1'-biphenyl]-3-yl)methoxy)phenyl)hex-4-ynoate (11.6 mg, 0.025 mmol, 1 equivalent) in EtOH (0.40 mL) in a 2-dram vial and at 23 °C was added a solution of NaOH (10 mg, 0.25 mmol, 10 equivalents) in H_2O (0.10 mL). The homogeneous, light yellow substrate solution immediately turned milky-white upon addition of the NaOH solution, but within 5 min the appearance returned to homogeneous and light yellow. After 90 min, the reaction mixture was poured into a separatory funnel containing EtOAc (5 mL) and 1 N H_2SO_4 (3 mL). The mixture was shaken, and the phases were separated. The aqueous phase was extracted with EtOAc (5 mL). The pooled organic phases were washed with H_2O (5 mL) and then with brine (5 mL), dried over Na_2SO_4, filtered, and concentrated. The residue was purified by preparative thin-layer chromatography (19:1 CH_2Cl_2:MeOH), which afforded the title compound as a clear film (9.0 mg, 83%). **R_f** = 0.20 (1:1 hexanes:EtOAc). **^{1}H NMR** (600 MHz; $CDCl_3$): δ 7.70 (s, 4H), 7.66-7.64 (m, 1H), 7.57-7.55 (m, 1H), 7.50-7.45 (m, 2H), 7.33-7.31 (m, 2H), 6.98-6.94 (m, 2H), 5.12 (s, 2H), 4.08-4.04 (m, 1H), 2.81 (dd, *J* = 15.7, 8.4 Hz, 1H), 2.72 (dd, *J* = 15.7, 6.7 Hz, 1H), 1.83 (d, *J* = 2.4 Hz, 3H). **^{13}C NMR** (125 MHz; $CDCl_3$): 176.7, 157.9, 144.5 (q, *J* = 1.0 Hz), 140.3, 138.0, 133.5, 129.6 (q, *J* = 32.4 Hz), 129.5, 128.6, 127.6, 127.4, 127.0, 126.5, 125.9 (q, *J* = 3.8 Hz), 124.4 (q, *J* = 270.5 Hz), 115.1, 79.5, 79.3, 70.0, 43.3, 33.3, 3.8. **^{19}F NMR** (376 MHz; $CDCl_3$): – 62.8. **IR** (film): 2881, 1687, 1590, 1489, 1384, 1308, 1225, 1150, 1109, 1056, 1003, 944, 831, 780, 748, 710, 690 cm^{-1}. **HRMS** (ESI+): Calculated for $C_{26}H_{21}F_3O_3$ $(M+H)^+$: 439.1516; Found 439.1510. **$[\alpha]_D^{25}$** = + 4.4 ° (*c* = 0.9, $CHCl_3$); lit.: + 7.2 ° (*c* = 0.5, $CHCl_3$);[14] lit.: + 10.9 ° (*c* = 0.44, $CHCl_3$).[15]

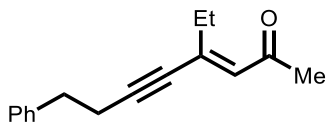

Pd-catalyzed Alkyne-Alkyne Coupling with Ketone Acceptors: Representative Procedure D, Synthesis of *(E)*-4-ethyl-8-phenyloct-3-en-5-yn-2-one (**8al**):

A 2-dram, screw-cap vial equipped with a stir bar was charged with $Pd(OAc)_2$ (11.2 mg, 0.05 mmol, 5 mol %) and tris(2,6-dimethoxyphenyl)phosphine (TDMPP, 22.1 mg, 0.05 mmol, 5 mol %). PhMe (1.0 mL) was added. The mixture was stirred for 15 minutes, generating a homogeneous, orange-red solution. To the solution was added 3-hexyn-2-one (GFS Chemicals, 164 μL, 1.5 mmol, 1.5 equivalents) followed by 4-phenyl-1-butyne (141 μL, 1.0 mmol, 1.0 equivalent). Upon addition of the donor alkyne, the reaction quickly changes in appearance to homogeneous and black. The reaction mixture was stirred for 14 h, at which point it was filtered through a pipette plug of Florisil® (3 cm), which was rinsed with Et_2O (10 mL). The solution was concentrated, and the residue was purified *via* column chromatography (10:1 hexanes:EtOAc) to afford the title compound (165.0 mg, 73%) as a clear, orange oil. $\mathbf{R_f}$ = 0.35 (10:1 hexanes:EtOAc). **^{1}H NMR** (500 MHz; $CDCl_3$): δ 7.33-7.29 (m, 2H), 7.24-7.22 (m, 3H), 6.31 (s, 1H), 2.88 (t, *J* = 7.4 Hz, 2H), 2.68-2.63 (m, 4H), 2.18 (s, 3H), 1.06 (t, *J* = 7.5 Hz, 3H). **^{13}C NMR** (125 MHz; $CDCl_3$): 197.9, 143.8, 140.4, 129.6, 128.6, 128.5, 126.5, 95.9, 83.0, 34.9, 32.0, 26.1, 21.9, 12.9. **IR** (film): 2986, 2928, 2893, 2182, 1656, 1562, 1434, 1337, 1154, 689 cm^{-1}. **HRMS** (ESI+): Calculated for $C_{16}H_{19}O$ $(M+H)^+$: 227.1431; Found 227.1430.

Note: This acceptor alkyne is co-polar on silica gel with some of the ynenone products; however, the excess acceptor can be removed *in vacuo* (rotary evaporator or high vacuum, *ca.* 1 torr).

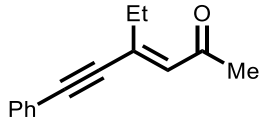

(E)-4-ethyl-6-phenylhex-3-en-5-yn-2-one (**8bl**):

Prepared according to Representative Procedure D using $Pd(OAc)_2$ (11.2 mg, 0.05 mmol, 5 mol %), tris(2,6-dimethoxyphenyl)phosphine (TDMPP, 22.1 mg, 0.05 mmol, 5 mol %), 3-hexyn-2-one (164 µL, 1.5 mmol), and freshly distilled phenylacetylene (110 µL, 1.0 mmol) in PhMe (1.0 mL). The reaction duration was 14 h. Purification *via* column chromatography (20:1 hexanes:EtOAc) afforded the title compound (126.0 mg, 64%) as a clear, orange oil. **R_f** = 0.35 (10:1 hexanes:EtOAc). **^{1}H NMR** (500 MHz; $CDCl_3$): δ 7.49-7.47 (m, 2H), 7.37-7.33 (m, 3H), 6.50 (s, 1H), 2.81 (qd, *J* = 7.5, 1.1 Hz, 2H), 2.24 (s, 3H), 1.21 (t, *J* = 7.5 Hz, 3H). **^{13}C NMR** (125 MHz; $CDCl_3$): 197.8, 143.0, 132.1, 130.2, 129.2, 128.6, 122.5, 94.9, 90.7, 32.1, 26.0, 13.0. **IR** (film): 2929, 2162, 1658, 1556, 1469, 1422, 1336, 1169, 1128, 1055, 942, 840, 746 cm^{-1}. **HRMS** (ESI+): Calculated for $C_{14}H_{15}O$ $(M+H)^+$: 199.1117; Found 199.1122.

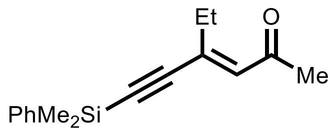

(E)-6-(dimethyl(phenyl)silyl)-4-ethylhex-3-en-5-yn-2-one (**8il**):

Prepared according to Representative Procedure D using $Pd(OAc)_2$ (11.2 mg, 0.05 mmol, 5 mol %), tris(2,6-dimethoxyphenyl)phosphine (TDMPP, 22.1 mg, 0.05 mmol, 5 mol %), 3-hexyn-2-one (110 µL, 1.0 mmol, 1.0 equivalent), and (dimethylphenylsilyl)acetylene (353 µL, 2.0 mmol, 2.0 equivalents) in PhMe (1.0 mL). The reaction duration was 24 h. Purification *via* column chromatography (20:1 hexanes:EtOAc) afforded the title compound (183.4 mg, 72%) as a clear, red-brown oil. **R_f** = 0.38 (10:1 hexanes:EtOAc). **^{1}H NMR** (400 MHz; $CDCl_3$): δ 7.65-7.62 (m, 2H), 7.42-7.38 (m, 3H), 6.48 (s, 1H), 2.72 (qd, *J* = 7.5, 1.1 Hz, 2H), 2.21 (s, 3H), 1.16 (t, *J* = 7.5 Hz, 3H), 0.47 (s, 6H). **^{13}C NMR** (125 MHz; $CDCl_3$): 197.9, 142.3, 136.4, 133.8, 131.2, 129.7, 128.1, 107.4, 98.4, 32.0, 25.7, 12.9, -0.9. **IR** (film): 3027, 2925, 2113, 1661, 1562, 1440, 1408, 1338, 1233, 1195, 1143, 1100, 1036, 1003, 943, 903,

827, 807, 771 cm^{-1}. **HRMS** (ESI+): Calculated for $C_{16}H_{21}OSi$ $(M+H)^+$: 257.1356; Found 257.1348.

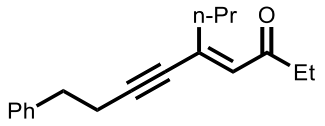

(E)-9-phenyl-5-propylnon-4-en-6-yn-3-one (**8am**):

Prepared according to Representative Procedure D using known acceptor 4-octyn-3-one[16] (123.7 mg, 1.0 mmol, 2.0 equivalents), 4-phenyl-1-butyne (70 μL, 0.50 mmol, 1.0 equivalents), $Pd(OAc)_2$ (5.6 mg, 0.025 mmol, 5 mol %), and TDMPP (11.0 mg, 0.025 mmol, 5 mol %) in PhMe (0.50 mL). The reaction duration was 24 h. Purification *via* column chromatography (20:1 hexanes:EtOAc) afforded the title compound (81.2 mg, 64%) as a clear, red-orange oil. $\mathbf{R}_f$ = 0.42 (10:1 hexanes:EtOAc). **^{1}H NMR** (600 MHz; $CDCl_3$): δ 7.32-7.29 (m, 2H), 7.24-7.21 (m, 3H), 6.33 (s, 1H), 2.87 (t, J = 7.4 Hz, 2H), 2.67-2.62 (m, 4H), 2.45 (q, J = 7.3 Hz, 2H), 1.55-1.50 (m, 2H), 1.07 (t, J = 7.3 Hz, 3H), 0.91 (t, J = 7.4 Hz, 3H). **^{13}C NMR** (100 MHz; $CDCl_3$): 200.9, 142.0, 140.4, 129.9, 128.6, 128.5, 126.5, 91.2, 83.4, 37.7, 35.0, 34.7, 21.92, 21.90, 13.9, 8.1. **IR** (film): 2986, 2920, 2183, 1660, 1563, 1475, 1434, 1342, 1104, 1019, 852, 738, 689 cm^{-1}. **HRMS** (ESI+): Calculated for $C_{18}H_{23}O$ $(M+H)^+$: 255.1743; Found 255.1737.

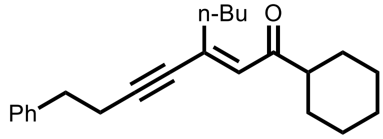

(E)-3-butyl-1-cyclohexyl-7-phenylhept-2-en-4-yn-1-one (**8an**):

Prepared according to Representative Procedure D using known acceptor 1-cyclohexylhept-2-yn-1-one[17] (96.2 mg, 0.50 mmol, 1.0 equivalents), 4-phenyl-1-butyne (141 μL, 1.0 mmol, 2.0 equivalents), $Pd(OAc)_2$ (5.6 mg, 0.025 mmol, 5 mol %), and TDMPP (11.1 mg, 0.025 mmol, 5 mol %) in PhMe (0.50 mL). The reaction duration was 24 h. Purification

via column chromatography (30:1 hexanes:Et_2O) afforded the title compound (114.8 mg, 71%) as a clear, red-orange oil. **R*f*** = 0.49 (10:1 hexanes:EtOAc). **^{1}H NMR** (400 MHz; $CDCl_3$): δ 7.33-7.29 (m, 2H), 7.25-7.21 (m, 3H), 6.37 (s, 1H), 2.87 (t, *J* = 7.4 Hz, 2H), 2.68-2.61 (m, 4H), 2.33-2.27 (m, 1H), 1.84-1.77 (m, 4H), 1.68-1.65 (m, 1H), 1.48-1.43 (m, 2H), 1.36-1.19 (m, 7H), 0.90 (t, *J* = 7.3 Hz, 3H). **^{13}C NMR** (100 MHz; $CDCl_3$): 203.6, 142.6, 140.4, 129.4, 128.6, 128.5, 126.5, 95.0, 83.6, 51.9, 35.0, 32.6, 30.8, 28.6, 26.0, 25.9, 22.6, 21.9, 14.1. **IR** (film): 2986, 2888, 2816, 2184, 1653, 1560, 1475, 1431, 1354, 1232, 1128, 1052, 1016, 849, 737, 689 cm^{-1}. **HRMS** (ESI+): Calculated for $C_{23}H_{31}O$ $(M+H)^+$: 323.2369; Found 323.2363.

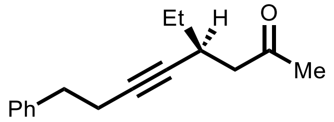

Cu-catalyzed Asymmetric Conjugate Reduction of Ynenones: Representative Procedure E, Synthesis of *(S)*-4-ethyl-8-phenyloct-5-yn-2-one (**9al**):

An oven-dried Biotage® microwave vial (0.50 – 2.0 mL size) equipped with a stir bar was charged with $Cu(OAc)_2$•H_2O (2.0 mg, 0.01 mmol, 5 mol %) and WALPHOS W001-1 ligand (**17**, 9.3 mg, 0.01 mmol, 5 mol %). The vial was sealed with a septum and flushed with Ar (balloon). Freshly distilled, degassed (Ar sparged) PhMe (0.75 mL) was added, and the mixture was stirred for 20 min to afford a bright green, homogeneous solution. Diethoxymethylsilane (32 µL, 0.20 mmol, 2.0 equivalents) was added, and the mixture was stirred until a change in appearance to homogeneous and brown was observed (*ca.* 15-30 min). To this solution was added *(E)*-4-ethyl-8-phenyloct-3-en-5-yn-2-one (45.3 mg, 0.20 mmol, 1 equivalent) as a solution in PhMe (200 µL + 50 µL rinse, prepared in a flame-dried ½-dram vial). The reaction was monitored by TLC (10:1 hexanes:EtOAc) for consumption of substrate and formation of its higher-R_f silyl enol ether. After 15 min, TBAF (1 M in THF, 0.70 mL, 0.70 mmol, 3.5 equivalents) was added, resulting in vigorous gas evolution. The deep red solution was stirred for 15 min, at which point TLC analysis (10:1 hexanes:EtOAc) indicated complete consumption of both the silyl enol ether and the starting material and the

formation of the conjugate reduction product. The reaction mixture was directly loaded onto a silica gel column that was eluted with 10:1 hexanes:EtOAc to afford the title compound (38.3 mg, 84%, 99% ee) as a clear, faintly yellow oil. **R*f*** = 0.19 (10:1 hexanes:EtOAc). **^{1}H NMR** (400 MHz; $CDCl_3$): δ 7.30-7.27 (m, 2H), 7.22-7.18 (m, 3H), 2.76-2.72 (m, 1H), 2.57 (dd, *J* = 16.0, 8.0 Hz, 1H), 2.47-2.42 (m, 3H), 2.13 (s, 3H), 1.53-1.30 (m, 2H), 0.95 (t, *J* = 7.4 Hz, 3H). **^{13}C NMR** (100 MHz; $CDCl_3$): 207.4, 141.0, 128.6, 128.4, 126.3, 82.9, 81.2, 49.1, 35.6, 30.6, 29.0, 28.2, 21.0, 11.7. **IR** (film): 2922, 2886, 2835, 1695, 1434, 1411, 1341, 1146, 1062, 739, 689 cm^{-1}. **HRMS** (ESI+): Calculated for $C_{16}H_{21}O$ $(M+H)^+$: 229.1587; Found 229.1591. **Chiral HPLC**: Agilent IA, 500:1 heptane:isopropanol, 0.5 mL/min, 220 nm, 22.93 min (major), 24.51 min (minor). **$[\alpha]_D^{25}$** = + 3.4° (*c* = 0.50, $CHCl_3$).

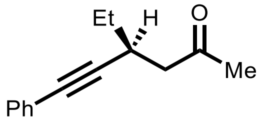

(S)-4-ethyl-6-phenylhex-5-yn-2-one (**9bl**):

Prepared according to Representative Procedure E using $Cu(OAc)_2$•H_2O (2.0 mg, 0.01 mmol, 5 mol %), WALPHOS ligand **17** (9.3 mg, 0.01 mmol, 5 mol %), *(E)*-4-ethyl-6-phenylhex-3-en-5-yn-2-one (39.6 mg, 0.20 mmol), and diethoxymethylsilane (32 µL, 0.20 mmol, 2.0 equivalents) in PhMe (0.75 mL for the catalyst solution and 0.25 mL total to transfer the substrate, including the rinse). The reaction duration was 15 min. Purification *via* column chromatography (10:1 hexanes:EtOAc) afforded the title compound (30.5 mg, 76%, 99% ee) as a viscous yellow oil. **R*f*** = 0.19 (10:1 hexanes:EtOAc). **^{1}H NMR** (400 MHz; $CDCl_3$): δ 7.37-7.35 (m, 2H), 7.27-7.24 (m, 3H), 3.04-2.97 (m, 1H), 2.74 (dd, *J* = 16.3, 7.6 Hz, 1H), 2.58 (dd, *J* = 16.3, 6.5 Hz, 1H), 2.20 (s, 3H), 1.64-1.41 (m, 2H), 1.06 (t, *J* = 7.4 Hz, 3H). **^{13}C NMR** (100 MHz; $CDCl_3$): 207.0, 131.7, 128.3, 127.8, 123.7, 91.9, 82.2, 48.7, 30.7, 29.5, 28.1, 11.8. **IR** (film): 2924, 2888, 2835, 1694, 1575, 1469, 1422, 1341, 1226, 1146, 1055, 747, 683 cm^{-1}. **HRMS** (ESI+): Calculated for $C_{14}H_{17}O$ $(M+H)^+$: 201.1274; Found 201.1276. **Chiral HPLC**: Agilent IA, 500:1 heptane:isopropanol, 0.8 mL/min, 214 nm, 12.58 min (major), 14.60 min (minor). **$[\alpha]_D^{25}$** = + 10.3° (*c* = 0.50, $CHCl_3$).

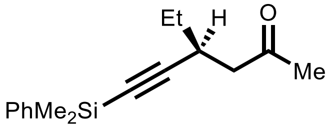

(S)-6-(dimethyl(phenyl)silyl)-4-ethylhex-5-yn-2-one (**9il**):

Prepared using a modification of Representative Procedure E. An oven-dried Biotage® microwave vial (0.50 – 2.0 mL size) equipped with a stir bar was charged with $Cu(OAc)_2$•H_2O (2.0 mg, 0.01 mmol, 5 mol %) and WALPHOS ligand **17** (9.3 mg, 0.01 mmol, 5 mol %). The vial was sealed with a septum and flushed with Ar (balloon). Freshly distilled, degassed (Ar sparged) PhMe (1.0 mL) was added, and the mixture was stirred for 20 min to afford a bright green, homogeneous solution. Diethoxymethylsilane (32 µL, 0.20 mmol, 2.0 equivalents) was added, and the mixture was stirred until a change in appearance to homogeneous and brown was observed (*ca.* 15-30 min). The vessel was then cooled to 0 °C, and substrate *(E)*-6-(dimethyl(phenyl)silyl)-4-ethylhex-3-en-5-yn-2-one (51.2 mg, 0.20 mmol) was added neat. The reaction mixture was stirred for 2 h, and then it was treated with 1% methanolic HCl (1.0 mL). After 10 min, the reaction mixture was loaded directly onto a silica gel column. Elution with 20:1 to 10:1 hexanes:EtOAc afforded the title compound (34.4 mg, 67%, 99% ee) as a viscous, clear, light yellow oil. $\mathbf{R}_f$ = 0.22 (10:1 hexanes:EtOAc). ^{1}**H NMR** (400 MHz; $CDCl_3$): δ 7.63-7.60 (m, 2H), 7.40-7.34 (m, 3H), 2.92-2.85 (m, 1H), 2.70 (dd, *J* = 16.2, 7.3 Hz, 1H), 2.54 (dd, *J* = 16.2, 6.7 Hz, 1H), 2.18 (s, 3H), 1.61-1.39 (m, 2H), 1.03 (t, *J* = 7.4 Hz, 3H), 0.38 (s, 6H). ^{13}**C NMR** (100 MHz; $CDCl_3$): 206.9, 137.6, 133.8, 129.4, 127.9, 111.0, 83.9, 48.5, 30.8, 29.9, 27.8, 11.7, -0.5. **IR** (film): 3026, 2923, 2890, 2836, 2138, 1969, 1440, 1408, 1341, 1232, 1145, 1100, 824, 769 cm^{-1}. **HRMS** (ESI+): Calculated for $C_{16}H_{23}O_3Si$ $(M+H)^+$: 259.1513; Found 259.1506. **Chiral HPLC**: Agilent IA, 1000:1 heptane:isopropanol, 0.8 mL/min, 220 nm, 12.04 min (major), 13.55 min (minor). $[\alpha]_D^{25}$ = + 4.1° (*c* = 0.50, $CHCl_3$).

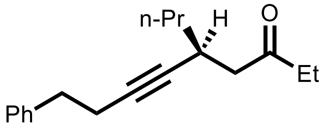

(S)-9-phenyl-5-propylnon-6-yn-3-one (**9am**):

Prepared according to Representative Procedure E using $Cu(OAc)_2•H_2O$ (2.0 mg, 0.01 mmol, 5 mol %), WALPHOS ligand **17** (9.3 mg, 0.01 mmol, 5 mol %), *(E)*-9-phenyl-5-propylnon-4-en-6-yn-3-one (50.8 mg, 0.20 mmol), and diethoxymethylsilane (32 µL, 0.20 mmol, 2.0 equivalents) in PhMe (0.75 mL for the catalyst solution and 0.25 mL total to transfer the substrate, including the rinse). The reaction duration was 2 h. Purification *via* column chromatography (20:1 hexanes:EtOAc) afforded the title compound (37.0 mg, 72%, 98% ee) as a clear, viscous, light yellow oil. $\mathbf{R_f}$ = 0.31 (10:1 hexanes:EtOAc). **^{1}H NMR** (400 MHz; $CDCl_3$): δ 7.30-7.26 (m, 2H), 7.22-7.19 (m, 3H), 2.86-2.80 (m, 1H), 2.77 (t, *J* = 7.5 Hz, 2H), 2.55 (dd, *J* = 15.8, 7.9 Hz, 1H), 2.45-2.38 (m, 5H), 1.49-1.29 (m, 4H), 1.04 (t, *J* = 7.3 Hz, 3H), 0.89 (t, *J* = 6.9 Hz, 3H). **^{13}C NMR** (100 MHz; $CDCl_3$): 210.0, 141.0, 128.6, 128.4, 126.3, 83.2, 80.9, 48.2, 37.4, 36.7, 35.6, 27.3, 21.0, 20.5, 14.0, 7.8. **IR** (film): 2985, 2916, 2890, 2832, 1692, 1475, 1434, 1391, 1342, 1101, 1061, 1016, 736 cm^{-1}. **HRMS** (ESI+): Calculated for $C_{18}H_{25}O$ $(M+H)^+$: 257.1900; Found 257.1894. **Chiral HPLC**: CHIRALPAK® AD-H, 200:1 heptane:isopropanol, 0.8 mL/min, 214 nm, 15.23 min (major), 18.15 min (minor). $[\alpha]_D^{25}$ = + 2.0° (*c* = 0.50, $CHCl_3$).

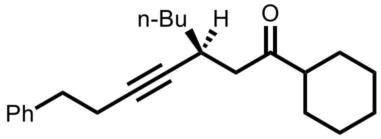

(S)-3-butyl-1-cyclohexyl-7-phenylhept-4-yn-1-one (**9an**):

Prepared according to Representative Procedure E using $Cu(OAc)_2•H_2O$ (2.0 mg, 0.01 mmol, 5 mol %), WALPHOS ligand **17** (9.3 mg, 0.01 mmol, 5 mol %), *(E)*-3-butyl-1-cyclohexyl-7-phenylhept-2-en-4-yn-1-one (64.5 mg, 0.20 mmol), and diethoxymethylsilane (32 µL, 0.20 mmol, 2.0 equivalents) in PhMe (0.75 mL for the catalyst solution and 0.25 mL total to transfer the substrate, including the rinse). The reaction duration was 1 h 30 min. Purification *via* column chromatography (10:1 to 6:1 hexanes:EtOAc) afforded impure product, which was repurified *via* column chromatography (20:1 hexanes:Et_2O) to deliver the

title compound (analytically pure, 32.7 mg, 50%, 99% ee) as a viscous yellow oil. **R$_f$** = 0.37 (10:1 hexanes:EtOAc). **^{1}H NMR** (300 MHz; $CDCl_3$): δ 7.31-7.27 (m, 2H), 7.22-7.17 (m, 3H), 2.87-2.75 (m, 3H), 2.61 (dd, *J* = 16.4, 7.3 Hz, 1H), 2.46-2.39 (m, 3H), 2.36-2.28 (m, 1H), 1.84-1.75 (m, 4H), 1.68-1.64 (m, 1H), 1.40-1.20 (m, 11H), 0.89 (t, *J* = 7.1 Hz, 3H). **^{13}C NMR** (75 MHz; $CDCl_3$): 212.4, 141.1, 128.6, 128.4, 126.3, 83.6, 80.6, 51.2, 46.6, 35.6, 34.9, 29.6, 28.4, 28.3, 27.1, 26.0, 25.81, 25.79, 22.6, 21.1, 14.2. **IR** (film): 3019, 2985, 2888, 2815, 1686, 1581, 1475, 1431, 1355, 1128, 1062, 1016, 880, 737, 689 cm^{-1}. **HRMS** (ESI+): Calculated for $C_{23}H_{33}O$ $(M+H)^+$: 325.2526; Found 325.2517. **Chiral HPLC**: Agilent IB, 400:1 heptane:isopropanol, 0.8 mL/min, 214 nm, 13.40 min (major), 15.85 min (minor). **$[\alpha]_D^{25}$** = − 6.0° (*c* = 0.50, $CHCl_3$).

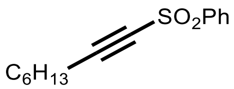

(oct-1-yn-1-ylsulfonyl)benzene (**7o**):

The procedure was adapted from the literature.[18] To a – 78 °C solution of 1-octyne (0.44 mL, 3 mmol, 1 equivalent) in THF (6 mL) was added *n*-BuLi (2.5 M in hexanes, 1.26 mL, 3.15 mmol, 1.05 equivalents). The reaction mixture was stirred for 30 min, and then a solution of diphenyl disulfide (688 mg, 3.15 mmol, 1.05 equivalents) in THF (6.3 mL) was added *via* cannula. The cooling bath was removed, and the mixture was stirred at room temperature for 1 h, after which time it was again cooled to – 78 °C and a solution of 2-nitrobenzyl bromide (713 mg, 3.3 mmol, 1.1 equivalents) in THF (6.6 mL) was added *via* cannula to consume phenylthiolate. After 5 min, the cooling bath was removed, and the mixture was stirred at room temperature for 1 h 30 min. The now bright red solution was quenched with saturated NH_4Cl (20 mL), and the mixture was poured into Et_2O (20 mL). The phases were separated, and the aqueous phase was extracted with Et_2O (3 x 20 mL). The pooled organic phases were dried over $MgSO_4$, filtered, and concentrated. Purification *via* column chromatography (20:1 hexanes:EtOAc) afforded the intermediate alkynyl sulfide (585 mg, 89%, *ca*. 90% purity judged by ^{1}H NMR) as a clear, colorless oil. **^{1}H NMR** (400 MHz;

$CDCl_3$): δ 7.42-7.40 (m, 2H), 7.34-7.29 (m, 2H), 7.21-7.17 (m, 1H), 2.45 (t, J = 7.1 Hz, 2H), 1.64-1.28 (m, 8H), 0.90 (t, J = 7.0 Hz, 3H).

To a 0 °C solution of this intermediate (575 mg, 2.6 mmol, 1 equivalent) in CH_2Cl_2 (6.5 mL) was added, dropwise, a freshly prepared solution of *m*-CPBA (77% purity, 1.0 g, 5.8 mmol, 2.2 equivalents) in CH_2Cl_2 (14.5 mL). During the addition, the homogeneous, clear solution became milky white. After 5 min, the cooling bath was removed, and the reaction mixture was stirred for 1 h at room temperature. The reaction mixture was then poured into Et_2O (50 mL) and washed with saturated aqueous $NaHCO_3$ (3 x 50 mL). The organic phase was dried over $MgSO_4$, filtered, and concentrated. Purification *via* column chromatography (8:1 hexanes:EtOAc) afforded the title compound (496 mg, 75%) as a clear, colorless oil. **R_f** = 0.50 (2:1 hexanes:EtOAc). **^{1}H NMR** (400 MHz; $CDCl_3$): δ 8.01-7.99 (m, 2H), 7.68-7.64 (m, 1H), 7.59-7.55 (m, 2H), 2.36 (t, J = 7.2 Hz, 2H), 1.58-1.51 (m, 2H), 1.36-1.20 (m, 6H), 0.86 (t, J = 6.9 Hz, 3H). **^{13}C NMR** (100 MHz; $CDCl_3$): 142.2, 134.0, 129.4, 127.3, 98.1, 78.3, 31.2, 28.5, 27.0, 22.5, 19.1, 14.1. **IR** (film): 2888, 2818, 2170, 1427, 1311, 1147, 1075, 747, 718, 677, 627, 567 cm^{-1}. **HRMS** (ESI+): Calculated for $C_{14}H_{19}O_2S$ $(M+H)^+$: 251.1100; Found 251.1096.

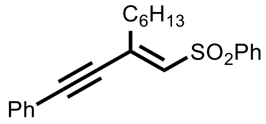

Pd-catalyzed Alkyne-Alkyne Coupling with Sulfone Acceptors: Representative Procedure F, Synthesis of *(E)*-((2-(phenylethynyl)oct-1-en-1-yl)sulfonyl)benzene (**8bo**):

A 2-dram, screw-cap vial equipped with a stir bar was charged with $Pd(OAc)_2$ (3.4 mg, 0.015 mmol, 3 mol %) and tris-2,6-dimethoxyphenylphosphine (6.3 mg, 0.015 mmol, 3 mol %). PhMe (0.50 mL) was added, and the mixture was stirred for 15 minutes, generating a homogeneous, orange-red solution. To the solution was added (oct-1-yn-1-ylsulfonyl)benzene (125.1 mg, 0.50 mmol, 1 equivalent) followed by freshly distilled phenylacetylene (69 μL, 0.625 mmol, 1.25 equivalents). The vial was capped, and the reaction mixture was stirred for

18 h. After this time, the reaction mixture was filtered through a pipette plug of Florisil® (3 cm), which was rinsed with Et_2O (10 mL). The solution was concentrated, and the residue was purified *via* column chromatography (8:1 to 4:1 hexanes:EtOAc) to afford the title compound (169.4 mg, 96%) as a viscous, brown oil. **R*f*** = 0.50 (2:1 hexanes:EtOAc). **^{1}H NMR** (400 MHz; $CDCl_3$): δ 7.96-7.94 (m, 2H), 7.66-7.62 (m, 1H), 7.58-7.55 (m, 2H), 7.44-7.42 (m, 2H), 7.37-7.32 (m, 3H), 6.62 (t, *J* = 0.9 Hz, 1H), 2.79-2.75 (m, 2H), 1.63-1.55 (m, 2H), 1.36-1.27 (m, 6H), 0.89 (t, *J* = 6.9 Hz, 3H). **^{13}C NMR** (100 MHz; $CDCl_3$): 142.0, 141.1, 133.6, 133.5, 132.1, 129.6, 129.4, 128.6, 127.5, 121.8, 96.7, 88.0, 31.8, 31.7, 29.0, 28.4, 22.7, 14.2. **IR** (film): 3364, 2914, 2887, 2817, 2164, 1575, 1550, 1468, 1437, 1425, 1300, 1134, 1070, 1055, 1011, 985, 803, 745 cm^{-1}. **HRMS** (ESI+): Calculated for $C_{22}H_{25}O_2S$ $(M+H)^+$: 353.1570; Found 353.1564.

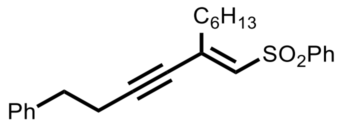

(E)-((2-(4-phenylbut-1-yn-1-yl)oct-1-en-1-yl)sulfonyl)benzene (**8ao**):

Prepared according to Representative Procedure F using $Pd(OAc)_2$ (3.4 mg, 0.015 mmol, 3 mol %), tris-2,6-dimethoxyphenylphosphine (6.3 mg, 0.015 mmol, 3 mol %), (oct-1-yn-1-ylsulfonyl)benzene (125.1 mg, 0.50 mmol), and 4-phenyl-1-butyne (88 μL, 0.625 mmol) in PhMe (0.50 mL). The reaction duration was 17 h. Purification *via* column chromatography (8:1 to 4:1 hexanes:EtOAc) afforded the title compound (190.4 mg, > 99% yield) as a viscous oil. **R*f*** = 0.50 (2:1 hexanes:EtOAc). **^{1}H NMR** (400 MHz; $CDCl_3$): δ 7.91-7.89 (m, 2H), 7.63-7.59 (m, 1H), 7.56-7.52 (m, 2H), 7.31-7.17 (m, 5H), 6.42 (s, 1H), 2.83 (t, *J* = 7.3 Hz, 2H), 2.65-2.58 (m, 4H), 1.43-1.38 (m, 2H), 1.30-1.22 (m, 6H), 0.89 (t, *J* = 6.9 Hz, 3H). **^{13}C NMR** (100 MHz; $CDCl_3$): 142.1, 141.9, 140.0, 133.4, 133.0, 129.3, 128.6, 128.5, 127.4, 126.6, 97.9, 80.6, 34.6, 32.0, 31.7, 29.0, 28.2, 22.7, 21.8, 14.2. **IR** (film): 2888, 2818, 2186, 1555, 1426, 1289, 1131, 1070, 806, 740 cm^{-1}. **HRMS** (ESI+): Calculated for $C_{24}H_{29}O_2S$ $(M+H)^+$: 381.1883; Found 381.1878.

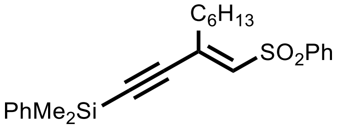

(E)-dimethyl(phenyl)(3-((phenylsulfonyl)methylene)non-1-yn-1-yl)silane (**8io**):

Prepared according to Representative Procedure F using $Pd(OAc)_2$ (3.4 mg, 0.015 mmol, 3 mol %), tris-2,6-dimethoxyphenylphosphine (6.3 mg, 0.015 mmol, 3 mol %), (oct-1-yn-1-ylsulfonyl)benzene (125.1 mg, 0.50 mmol), and (dimethylphenylsilyl)acetylene (111 µL, 0.625 mmol) in PhMe (0.50 mL). The reaction duration was 22 h. Purification *via* column chromatography (8:1 hexanes:EtOAc) afforded the title compound (180.2 mg, 88% yield) as a viscous, golden yellow oil. $\mathbf{R}_f$ = 0.53 (2:1 hexanes:EtOAc). **^{1}H NMR** (400 MHz; $CDCl_3$): δ 7.93-7.90 (m, 2H), 7.65-7.61 (m, 1H), 7.59-7.53 (m, 4H), 7.40-7.35 (m, 3H), 6.58 (t, *J* = 1.0 Hz, 1H), 2.72-2.68 (m, 2H), 1.58-1.50 (m, 2H), 1.32-1.24 (m, 6H), 0.88 (t, *J* = 6.9 Hz, 3H), 0.43 (s, 6H). **^{13}C NMR** (100 MHz; $CDCl_3$): 141.7, 140.4, 135.9, 134.9, 133.7, 133.6, 129.9, 129.4, 128.2, 127.6, 104.3, 101.0, 31.7, 21.5, 28.9, 28.2, 22.6, 14.2, -1.1. **IR** (film): 3026, 2916, 2888, 2818, 2116, 1555, 1426, 1408, 1359, 1289, 1233, 1134, 1101, 1070, 1010, 985, 873, 826, 803 cm^{-1}. **HRMS** (ESI+): Calculated for $C_{24}H_{31}O_2SSi$ $(M+H)^+$: 411.1809; Found 411.1802.

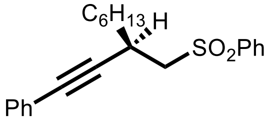

Cu-catalyzed Asymmetric Conjugate Reduction of Enynyl Sulfones: Representative Procedure G, Synthesis of *(R)*-((2-(phenylethynyl)octyl)sulfonyl)benzene (**9bo**):

An oven-dried Biotage® microwave vial (0.50 – 2.0 mL size) equipped with a stir bar was charged with $Cu(OAc)_2{\bullet}H_2O$ (1.0 mg, 0.005 mmol, 5 mol %) and WALPHOS W001-1 ligand (**17**, 4.6 mg, 0.005 mmol, 5 mol %). The vial was sealed with a septum and flushed with Ar (balloon). Freshly distilled, degassed (Ar sparged) PhMe (0.25 mL) was added, and the mixture was stirred for 20 min to afford a bright green, homogeneous solution.

Diethoxymethylsilane (32 μL, 0.20 mmol, 2.0 equivalents) was added, and the mixture was stirred until a change in appearance to homogeneous and brown was observed (*ca.* 15-30 min). Substrate *(E)*-((2-(phenylethynyl)oct-1-en-1-yl)sulfonyl)benzene (35.2 mg, 0.10 mmol, 1 equivalent) was added as a solution in PhMe (200 μL + 50 μL rinse, prepared in a ½-dram vial that was not dried). Water (9 μL, 0.50 mmol, 5 equivalents) was added, and the vessel was heated to 50 °C and stirred under an Ar balloon. After 4 h, the vessel was cooled to room temperature and the reaction mixture was directly loaded onto a silica gel column. Elution with 8:1 to 4:1 hexanes:EtOAc afforded the title compound (28.9 mg, 82%, 98% ee) as a viscous, light yellow oil. **R*f*** = 0.52 (2:1 hexanes:EtOAc). **^{1}H NMR** (500 MHz; $CDCl_3$): δ 7.98-7.96 (m, 2H), 7.59-7.50 (m, 3H), 7.25-7.16 (m, 5H), 3.45 (dd, *J* = 14.3, 8.0 Hz, 1H), 3.27 (dd, *J* = 14.3, 5.1 Hz, 1H), 3.21-3.15 (m, 1H), 1.74-1.69 (m, 1H), 1.64-1.56 (m, 1H), 1.53-1.42 (m, 2H), 1.34-1.23 (m, 6H), 0.88 (t, *J* = 7.0 Hz, 3H). **^{13}C NMR** (100 MHz; $CDCl_3$): 139.7, 133.8, 131.7, 129.3, 128.5, 128.2, 128.1, 123.0, 89.3, 83.6, 60.8, 35.1, 31.8, 28.9, 27.9, 26.9, 22.7, 14.2. **IR** (film): 3019, 2887, 2817, 1576, 1469, 1426, 1289, 1128, 1070, 1011, 846, 745 cm^{-1}. **HRMS** (ESI+): Calculated for $C_{22}H_{27}O_2S$ $(M+H)^+$: 355.1726; Found 355.1721. **Chiral HPLC**: Agilent IB, 98:2 heptane:isopropanol, 0.6 mL/min, 254 nm, 21.06 min (major), 23.37 min (minor). **$[\alpha]_D^{25}$** = + 27.4° (*c* = 0.50, $CHCl_3$)

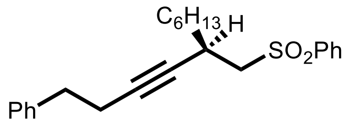

(R)-((2-(4-phenylbut-1-yn-1-yl)octyl)sulfonyl)benzene (**9ao**):

Prepared according to Representative Procedure G using $Cu(OAc)_2$•H_2O (1.0 mg, 0.005 mmol, 5 mol %), WALPHOS ligand **17** (4.6 mg, 0.005 mmol, 5 mol %), *(E)*-((2-(4-phenylbut-1-yn-1-yl)oct-1-en-1-yl)sulfonyl)benzene (37.9 mg, 0.10 mmol), diethoxymethylsilane (32 μL, 0.20 mmol, 2.0 equivalents), and water (9 μL, 0.50 mmol, 5 equivalents) in PhMe (0.25 mL for the catalyst solution and 0.25 mL total to transfer the substrate, including the rinse). The reaction duration was 2 h 30 min. Purification *via* column chromatography (6:1 to 4:1 hexanes:Et_2O) afforded the title compound (28.3 mg, 74%, 99%

ee) as a viscous, clear, light yellow oil. **R$_f$** = 0.55 (2:1 hexanes:EtOAc). **^{1}H NMR** (400 MHz; $CDCl_3$): δ 7.92-7.90 (m, 2H), 7.65-7.61 (m, 1H), 7.56-7.50 (m, 2H), 7.29-7.25 (m, 2H), 7.21-7.17 (m, 1H), 7.15-7.12 (m, 2H), 3.28 (dd, *J* = 14.3, 8.0 Hz, 1H), 3.13 (dd, *J* = 14.3, 5.1 Hz, 1H), 2.93-2.83 (m, 1H), 2.62 (t, *J* = 7.5 Hz, 2H), 2.22-2.17 (m, 2H), 1.60-1.53 (m, 1H), 1.41-1.19 (m, 9H), 0.88 (t, *J* = 7.0 Hz, 3H). **^{13}C NMR** (100 MHz; $CDCl_3$): 140.8, 139.8, 133.7, 129.2, 128.54, 128.49, 128.40, 126.4, 82.9, 80.6, 61.0, 35.2, 35.1, 31.8, 28.9, 27.2, 26.7, 22.7, 20.9, 14.2. **IR** (film): 3020, 2985, 2886, 2817, 1475, 1427, 1302, 1289, 1132, 1070, 774, 737 cm^{-1}. **HRMS** (ESI+): Calculated for $C_{24}H_{31}O_2S$ $(M+H)^+$: 383.2039; Found 383.2036. **Chiral HPLC**: Agilent IB, 98:2 heptane:isopropanol, 0.8 mL/min, 220 nm, 18.92 min (major), 21.28 min (minor). **$[\alpha]_D^{25}$** = + 19.6° (*c* = 0.50, $CHCl_3$).

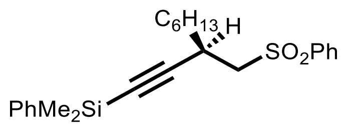

(R)-dimethyl(phenyl)(3-((phenylsulfonyl)methyl)non-1-yn-1-yl)silane (**9io**):

Prepared according to Representative Procedure G using $Cu(OAc)_2•H_2O$ (1.0 mg, 0.005 mmol, 5 mol %), WALPHOS ligand **17** (4.6 mg, 0.005 mmol, 5 mol %), *(E)*-dimethyl(phenyl)(3-((phenylsulfonyl)methylene)non-1-yn-1-yl)silane (41.1 mg, 0.10 mmol), diethoxymethylsilane (32 μL, 0.20 mmol, 2.0 equivalents), and water (9 μL, 0.50 mmol, 5 equivalents) in PhMe (0.25 mL for the catalyst solution and 0.25 mL total to transfer the substrate, including the rinse). The reaction duration was 6 h. Purification *via* column chromatography (6:1 to 4:1 hexanes:Et_2O) afforded the title compound (37.6 mg, 91%, 99% ee) as a viscous, clear, light yellow oil. **R$_f$** = 0.58 (2:1 hexanes:EtOAc). **^{1}H NMR** (400 MHz; $CDCl_3$): δ 7.93-7.91 (m, 2H), 7.62-7.57 (m, 1H), 7.53-7.47 (m, 4H), 7.40-7.33 (m, 3H), 3.40 (dd, *J* = 14.3, 7.1 Hz, 1H), 3.20 (dd, *J* = 14.3, 5.8 Hz, 1H), 3.07-3.00 (m, 1H), 1.69 (s, 1H), 1.59-1.45 (m, 2H), 1.41-1.23 (m, 7H), 0.88 (t, *J* = 6.9 Hz, 3H), 0.28 (s, 3H), 0.28 (s, 3H). **^{13}C NMR** (100 MHz; $CDCl_3$): 139.6, 137.1, 133.9, 133.8, 129.5, 129.3, 128.4, 127.9, 107.8, 85.7, 60.6, 34.9, 31.7, 28.8, 28.2, 26.7, 22.7, 14.2, -0.67, -0.70. **IR** (film): 2887, 2818, 2141, 1427, 1408, 1303, 1232, 1129, 1100, 1071, 824, 805, 770, 723, 691, 654 cm^{-1} **HRMS** (ESI+):

Calculated for $C_{24}H_{33}O_2SSi$ $(M+H)^+$: 413.1965; Found 413.1959. **Chiral HPLC**: Agilent IB, 99:1 heptane:isopropanol, 0.8 mL/min, 220 nm, 15.27 min (major), 16.25 min (minor). **$[\alpha]_D^{25}$** = + 25.0° (*c* = 0.50, $CHCl_3$).

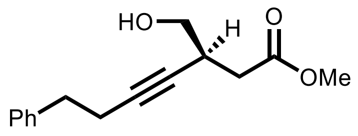

methyl *(S)*-3-(hydroxymethyl)-7-phenylhept-4-ynoate (**33**):

An oven-dried Biotage® microwave vial (0.5 – 2.0 mL size) was equipped with a stir bar, sealed with a septum, purged with nitrogen, and kept under a nitrogen atmosphere. A solution of methyl *(S)*-3-(((methoxycarbonyl)oxy)methyl)-7-phenylhept-4-ynoate (30.0 mg, 0.099 mmol, 1.0 equivalent) in freshly-distilled methanol (1.0 mL) was added *via* syringe. Anhydrous potassium carbonate (1.6 mg, 0.012 mmol, 0.12 equivalent) was added under nitrogen, and the reaction mixture was stirred for 3 h at room temperature. The reaction mixture was quenched with pH 7 phosphate buffer (1 mL), stirred for 10 min, and diluted with EtOAc (2 mL). The phases were separated, and the aqueous phase was extracted three times with EtOAc (3 x 2 mL). The organic phases were combined, washed with water, dried with sodium sulfate, and concentrated *in vacuo*. The crude product was purified *via* column chromatography (2:1 hexanes:EtOAc) to afford the title compound as a clear oil (15.7 mg, 65%). The corresponding lactone (*vide infra*) was also isolated (5.1 mg, 24%). **R_f** = 0.17 (4:1 hexanes:EtOAc). **^{1}H NMR** (400 MHz, $CDCl_3$) δ 7.32-7.28 (m, 2H), 7.24-7.20 (m, 3H), 3.69 (s, 3H), 3.61-3.50 (m, 2H), 3.04-2.98 (m, 1H), 2.79 (t, *J* = 7.3 Hz, 2H), 2.57-2.45 (m, 4H), 1.74 (br s, 1H). **^{13}C NMR** (125 MHz, $CDCl_3$) δ 172.3, 140.7, 128.6, 128.5, 126.5, 83.2, 79.8, 65.0, 52.0, 36.5, 35.2, 31.7, 21.0. **IR** (film): 3446, 3027, 2950, 1737, 1454, 1437, 1361, 1259, 1211, 1165, 1059, 700 cm^{-1}. **HRMS:** Calculated for $C_{15}H_{18}O_3Na$ $(M+Na)^+$: 269.1154; Found 269.1154. **$[\alpha]_D^{25}$** = + 21.54° (*c* = 0.52; CH_2Cl_2)

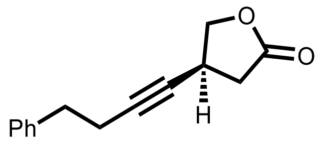

(S)-4-(4-phenylbut-1-yn-1-yl)dihydrofuran-2(3H)-one (**34**):

Methyl *(S)*-3-(((methoxycarbonyl)oxy)methyl)-7-phenylhept-4-ynoate (30.0 mg, 0.099 mmol, 1.0 equivalent) was hydrolyzed as described above. The crude material was transferred to a separate oven-dried Biotage® microwave vial (0.5 – 2.0 mL size) equipped with a stir bar, to which was added 1,1,3,3-tetrabutyl-1,3-diisothiocyanatodistannoxane (Otera's catalyst[19]) (11.8 mg, 0.0099 mmol, 0.10 equivalent). The vial was sealed with a septum and purged with nitrogen. PhMe (0.5 mL) was added *via* syringe, and the vial was sealed and heated to 85 °C for 2 h 30 min. The vial was cooled to room temperature, and the solvent was removed *in vacuo*. The crude product was purified *via* column chromatography (4:1 hexanes:EtOAc) to afford the title compound as a clear oil (16.5 mg, 0.077 mmol, 78% yield). **R_f** = 0.41 (2:1 hexanes:EtOAc). **^{1}H NMR** (400 MHz, $CDCl_3$) δ 7.31-7.27 (m, 2H), 7.23-7.17 (m, 3H), 4.43-4.39 (dd, *J* = 8.7 Hz, 7.6 Hz, 1H), 4.10-4.06 (dd, *J* = 8.7 Hz, 7.5 Hz, 1H), 3.34-3.25 (m, 1H), 2.79 (t, *J* = 7.5 Hz, 2H), 2.74-2.68 (dd, *J* = 17.3 Hz, 8.5 Hz, 1H), 2.52-2.43 (m, 3H). **^{13}C NMR** (125 MHz, $CDCl_3$): δ 175.6, 140.4, 128.6, 128.5, 126.5, 83.4, 78.3, 72.7, 35.4, 35.0, 27.5, 20.9. **IR** (film): 3062, 3027, 2917, 1786, 1170, 1026, 700 cm^{-1}. **HRMS:** Calculated for $C_{14}H_{14}O_2Na$ $(M+Na)^+$: 237.0891; Found 237.0887. **$[\alpha]_D^{25}$** = + 26.56° (*c* = 0.845; CH_2Cl_2)

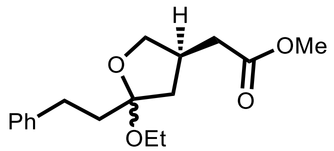

methyl 2-((3R)-5-ethoxy-5-phenethyltetrahydrofuran-3-yl)acetate (**35**):

This procedure was adapted from the literature.[20] A flame-dried Biotage® microwave vial (0.5 – 2.0 mL size) equipped with a stir bar was charged with AuCl (2.2 mg, 9.5 µmol,

0.10 equivalent) and pyridinium *p*-toluenesulfonate (2.3 mg, 9.2 µmol, 0.10 equivalent). The reaction vessel was sealed with a rubber septum and evacuated and backfilled with argon (3 x cycle). To these solids was then added EtOH (200 µL) to afford a homogeneous, light yellow reaction mixture which was then cooled to 0 °C. To this solution was then added methyl *(S)*-3-(hydroxymethyl)-7-phenylhept-4-ynoate (24.0 mg, 0.09 mmol, 1.0 equivalent, prepared as described above) as a solution in EtOH (500 µL, followed by a 200 µL rinse). The resulting mixture was then stirred at 0 °C for 30 min, diluted with EtOAc (5 mL), and then poured into a separatory funnel containing a saturated aqueous solution of $NaHCO_3$ (3 mL). The phases were separated, and the organic phase was washed with additional $NaHCO_3$ (3 x 5 mL) and brine (3 x 5 mL). The organic phase was dried over Na_2SO_4, filtered, and concentrated *in vacuo*. The resulting residue was purified by chromatography over basic alumina (10:1 hexanes:EtOAc) to afford the title compound as a 1:1 mixture of diastereomers and as a colorless oil (14.2 mg, 0.05 mmol, 50% yield). $\mathbf{R_f}$ = 0.60 (2:1 hexanes:EtOAc). **^{1}H NMR** (500 MHz, $CDCl_3$) δ 7.19-7.07 (m, 5H), 4.09 (dd, *J*= 8.0, 8.0 Hz, 1H), 4.08 (dd, *J*= 8.0, 8.0 Hz, 1H), 3.57-3.44 (m, 4 H), 3.35-3.27 (m, 8H), 2.92-2.82 (m, 1H), 2.74-2.47 (m, 5H), 2.41-2.15 (m, 5H), 2.09-2.00 (m, 2H), 1.90-1.81 (m, 3H), 1.66 (dd, *J*= 5.0, 15.0 Hz, 1H), 1.22 (dd, *J*= 10.5, 12.5 Hz, 1H), 1.13-1.07 (m, 6H). **^{13}C NMR** (125 MHz, C_6D_6): δ 172.5, 172.1, 142.33, 142.31, 128.6, 128.5, 126.0, 109.4, 109.2, 72.3, 55.8, 55.6, 50.9, 50.9, 42.7, 42.3, 38.6, 38.3, 37.4, 37.1, 34.8, 34.6, 31.5, 31.4, 15.9, 15.9. **IR** (film): 2952, 1739, 1438, 1261, 1175, 1068 cm^{-1}. **HRMS:** Calculated for $C_{17}H_{24}O_4Na$ $(M+Na)^+$: 315.1572; Found 315.1583. $[\alpha]_D^{24}$= –4.7° (*c* = 1.00, CH_2Cl_2).

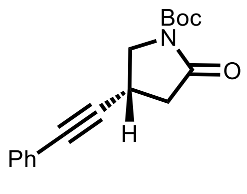

tert-butyl *(R)*-2-oxo-4-(phenylethynyl)pyrrolidine-1-carboxylate (**36**):

An oven-dried Biotage® microwave vial (0.5 – 2.0 mL size) equipped with a stir bar was charged with methyl *(R)*-3-(((*tert*-butoxycarbonyl)amino)methyl)-5-phenylpent-4-ynoate

(40 mg, 0.126 mmol), capped with a rubber septum, and purged with argon. PhMe (2.5 mL) was added *via* syringe, and the mixture was cooled to – 20 ˚C. A solution of $AlMe_3$ (0.095 mL, 0.190 mmol, 2.0 M in PhMe) was then added dropwise slowly over 10 min. The reaction was stirred at – 20 ˚C for 2 h, and then it was quenched with saturated aqueous Rochelle's salt (3.0 mL). The mixture was warmed to room temperature and extracted with EtOAc (3 x 5 mL). The combined organic phases were dried over Na_2SO_4, filtered, concentrated *in vacuo*, and purified by column chromatography (9:1 to 7:3 hexanes:EtOAc) to afford the title compound as a colorless oil (29 mg, 0.102 mmol, 81% yield, 99% *brsm*). $\mathbf{R_f}$ = 0.30 (3:1 hexanes:EtOAc). **^{1}H NMR** (500 MHz, $CDCl_3$) δ 7.42-7.38 (m, 2H), 7.33-7.29 (m, 3H), 4.08 (dd, J = 10.7, 8.1 Hz, 1H), 3.78 (dd, J = 10.7, 7.5 Hz, 1H), 3.37 (m, 1H), 2.86 (dd, J = 17.3, 8.5 Hz, 1H), 2.72 (dd, J = 17.1, 8.9 Hz, 1H), 1.55 (s, 9H). **^{13}C NMR** (125 MHz, $CDCl_3$) δ 172.1, 149.8, 131.8, 128.6, 128.5, 122.7, 88.0, 83.5, 82.9, 51.9, 39.9, 28.2, 23.5. **IR** (film) = 2978, 2927, 2358, 2337, 1788, 1752, 1714, 1367, 1342, 1314, 1255, 1150 cm^{-1}. **HRMS**: Calculated for $C_{17}H_{19}NO_3Na$ $(M+Na)^+$: 308.1263; Found 308.1267. $\mathbf{[\alpha]_D^{24}}$ = + 46.05° (c = 0.35; CH_2Cl_2).

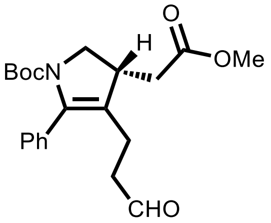

tert-butyl *(R)*-3-(2-methoxy-2-oxoethyl)-4-(3-oxopropyl)-5-phenyl-2,3-dihydro-1H-pyrrole-1-carboxylate (**37**):

This procedure was adapted from the literature.[21] A flame-dried Biotage® microwave vial (0.5 – 2.0 mL size) equipped with a stir bar was charged with LiBr (43.8 mg, 0.50 mmol, 2 equivalents). The reaction vessel was then capped with a septum, placed under high vacuum, and flame-dried. Upon cooling to room temperature, the septum was removed and $Pd(OAc)_2$ (2.8 mg, 13.0 μmol, 0.05 equivalent) was added. The reaction vessel was then sealed, evacuated, and backfilled with N_2. In a separate vial, methyl *(R)*-3-(((*tert*-

butoxycarbonyl)amino)methyl)-5-phenylpent-4-ynoate (80.0 mg, 0.25 mmol, 1.0 equivalent) was dissolved in THF (300 μL), and to the resulting homogeneous, colorless solution was added acrolein (168.0 μL, 141.0 mg, 10.0 equivalents). The resulting homogenous mixture was transferring to the $Pd(OAc)_2$/LiBr mixture *via* cannula, using THF (200 μL) to rinse the vial containing the substrate. The resulting homogenous, dark red mixture was stirred at 23 °C for 4 h, at which point additional $Pd(OAc)_2$ (1.4 mg, 6.5 μmol, 0.025 equivalent) and LiBr (21.9 mg, 0.25 mmol, 1 equivalent, *not dried*) were added. The resulting homogeneous, deep red mixture was stirred at 23 °C for an additional 10 h. The reaction mixture was diluted with EtOAc/CH_2Cl_2 (1:1, 1 mL) and filtered through a short pipette column of Florisil®, eluting with EtOAc/CH_2Cl_2 (1:1, 5 mL). The resulting yellow/green reaction mixture was then concentrated *in vacuo*, and the resulting residue was dissolved in hexanes:EtOAc (3:1, 2 mL) to afford a heterogeneous mixture that was once again filtered through Florisil®, eluting with hexanes:EtOAc (3:1, 5 mL). Concentration of the resulting solution *in vacuo* afforded a light yellow residue that was purified by column chromatography (3:1 hexanes:EtOAc) to afford the title compound as a colorless oil (60.0 mg, 0.16 mmol, 64% yield). $\mathbf{R_f}$ = 0.25 (2:1 hexanes:EtOAc). **^{1}H NMR** (400 MHz, $CDCl_3$) δ 9.61 (s, 1H), 7.34-7.28 (m, 3H), 7.17-7.15 (m, 2H), 4.06 (dd, *J*= 2.0, 9.6 Hz, 1H), 3.75-3.70 (m, 4H), 3.20 (tt, *J*= 4.4, 9.3 Hz, 1H), 2.61 (dd, *J*= 4.4, 16.0 Hz, 1H), 2.55-2.46 (m, 1H), 2.44-2.34 (m, 3H), 2.24-2.19 (m, 1H), 1.07 (s, 9H). **^{13}C NMR** (100 MHz, $CDCl_3$) δ 201.6, 172.6, 152.5, 138.8, 133.8, 128.8, 128.0, 127.8, 121.7, 80.0, 53.4, 52.0, 42.5, 38.3, 37.8, 27.9, 18.3. **IR** (film) = 2977, 1735, 1688, 1395, 1366, 1166 cm^{-1}. **HRMS**: Calculated for $C_{21}H_{28}O_5$ $(M+H)^+$: 374.1967, Found 374.1956. $\mathbf{[\alpha]_D^{25}}$ = + 42.7° (*c* = 0.50, CH_2Cl_2).

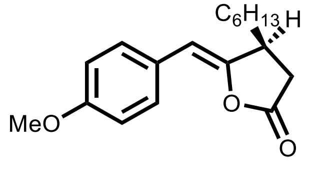

(*S*,*Z*)-4-hexyl-5-(4-methoxybenzylidene)dihydrofuran-2(3H)-one (**39**):

This procedure was adapted from the literature.[22] A 2-dram vial equipped with a stir bar was charged with methyl *(S)*-3-((4-methoxyphenyl)ethynyl)nonanoate (30.2 mg, 0.10 mmol, 1 equivalent), and to the vial were added MeOH (0.45 mL) and H_2O (50 µL). LiOH•H_2O (12.6 mg, 0.30 mmol, 3 equivalents) was added, and the homogeneous light yellow solution was stirred at 23 °C for 18 h. The reaction mixture was acidified with 1 N H_2SO_4 (1 mL) and extracted with Et_2O (2 x 5 mL). The pooled organic phases were dried over $MgSO_4$, filtered, and concentrated into an oven-dried Biotage® microwave vial (0.50 mL – 2.0 mL size). A stir bar was added, and the vial was flushed with N_2 (balloon). Degassed (N_2 sparged) MeCN (0.20 mL) was added, followed by Et_3N (*ca.* 0.7 µL, 0.005 mmol, 5 mol %) and $Pd(PhCN)_2Cl_2$ (1.0 mg, 0.0025 mmol, 0.025 equivalents). The vial was sealed with a septum-lined cap and heated to 80 °C, yielding a homogeneous light yellow solution. After 2 h, the vessel was cooled to room temperature, and additional $Pd(PhCN)_2Cl_2$ (1.0 mg, 0.0025 mmol, 0.025 equivalents) was added. The vessel was sealed again and heated to 80 °C for an additional 3 h. The vessel was cooled to room temperature, and the reaction mixture was diluted with Et_2O (3 mL). The mixture was filtered through a pipette plug of Florisil®, rinsing with additional Et_2O (10 mL). The solution was concentrated, and the residue was purified *via* preparative thin-layer chromatography (6:1 hexanes:EtOAc) to afford the title compound (17.0 mg, 59%) as a viscous, light yellow oil and as an inseparable mixture of olefin isomers (8:1 as judged by ^1H NMR spectroscopy based on the methyl ester and vinylic hydrogen signals). The major isomer was characterized, and the assignment of the olefin geometry was made by analogy to the above literature precedent cited. $\mathbf{R_f}$ = 0.69 (4:1 hexanes:EtOAc). $\mathbf{^1H}$ **NMR** (400 MHz, $CDCl_3$, major isomer) δ 7.52-7.48 (m, 2H), 6.88-6.85 (m, 2H), 5.47 (d, *J* = 1.1 Hz, 1H), 3.82 (s, 3H), 3.19-3.11 (m, 1H), 2.81 (dd, *J* = 17.9, 9.5 Hz, 1H), 2.37 (dd, *J* = 17.9, 5.6 Hz, 1H), 1.78-1.71 (m, 1H), 1.56-1.49 (m, 9H), 0.89 (t, *J* = 6.8 Hz, 3H). $\mathbf{^{13}C}$ **NMR** (100 MHz, $CDCl_3$, major isomer) δ 174.5, 158.5, 151.3, 129.8, 126.8, 114.0, 104.1, 55.4, 39.0, 34.5, 33.6, 31.8, 29.2, 26.8, 22.7, 14.2. **IR** (film) = 2888, 2816, 1781, 1661, 1586, 1490, 1443, 1399, 1279, 1233, 1163, 1109, 1085, 1019, 948, 837 cm^{-1}. **HRMS**: Calculated for $C_{18}H_{25}O_3$ $(M+H)^+$: 289.1798, Found 289.1806. $\mathbf{[\alpha]_D^{25}}$ = + 6.9° (*c* = 0.50, CH_2Cl_2).

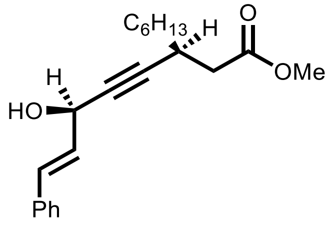

methyl *(S)*-3-((*R*,*E*)-3-hydroxy-5-phenylpent-4-en-1-yn-1-yl)nonanoate (**43**):

This procedure was adapted from the literature.[23] An oven-dried Biotage® microwave vial (0.50 – 2.0 mL size) equipped with a stir bar was charged with (*S*,*S*)-ProPhenol catalyst (6.4 mg, 0.010 mmol, 10 mol %) and triphenylphosphine oxide (5.6 mg, 0.020 mmol, 20 mol %). The vial was sealed with a septum and flushed with Ar, and then distilled, degassed (Ar sparge) PhMe (75 μL) was added. Methyl *(S)*-3-ethynylnonanoate (23.6 mg, 0.120 mmol, 1.2 equivalents) was added *via* tared μL syringe, and the vessel was cooled to 0 °C. $ZnMe_2$ (1.2 M in PhMe, 125 μL, 0.150 mmol, 1.5 equivalents) was added, and the cooling bath was removed. The reaction mixture was stirred for 45 min at room temperature, and then it was again cooled to 0 °C. Freshly distilled *trans*-cinnamaldehyde (12.6 μL, 0.10 mmol, 1.0 equivalent) was added, and the vessel was transferred to a cold room (4 °C) where it was stirred for 48 h under an Ar balloon. The vessel was then warmed to room temperature, and the reaction mixture was quenched by the addition of 1 N HCl (3 mL). The mixture was poured into Et_2O (10 mL), and the phases were separated. The aqueous phase was extracted with Et_2O (3 x 5 mL), and the pooled organic phases were dried over $MgSO_4$, filtered, and concentrated. Only a single diastereomer was observed by ^{1}H NMR analysis of the crude material. The crude material was purified by column chromatography (4:1 to 2:1 hexanes:EtOAc) to afford the title compound (27.8 mg, 85%, > 19:1 dr) as a viscous, light yellow oil. Assignment of (*R*)-stereochemistry at the carbon bearing the alcohol was made by analogy to the literature precedent cited. **R_f** = 0.41 (2:1 hexanes:EtOAc). **^{1}H NMR** (400 MHz; $CDCl_3$): δ 7.42-7.39 (m, 2H), 7.35-7.31 (m, 2H), 7.28-7.24 (m, 1H), 6.76 (dd, *J* = 15.8, 1.0 Hz, 1H), 6.28 (dd, *J* = 15.8, 5.8 Hz, 1H), 5.04 (tt, *J* = 6.0, 1.6 Hz, 1H), 3.69 (s, 3H), 2.97-2.90 (m, 1H), 2.56 (dd, *J* = 15.4, 7.8 Hz, 1H), 2.48 (dd, *J* = 15.4, 6.9 Hz, 1H), 2.08 (d, *J* = 6.0 Hz, 1H), 1.54-1.27 (m, 10H), 0.87 (t, *J* = 6.9 Hz, 3H). **13C NMR** (100 MHz; $CDCl_3$): 172.2, 136.3, 131.8, 128.7, 128.6, 128.1, 126.9, 88.9, 80.9, 63.1, 51.9, 40.0, 34.6, 31.9, 29.1, 28.5,

27.3, 22.8, 14.2. **IR** (film): 3375 (br), 2984, 2887, 2817, 1714, 1473, 1418, 1344, 1249, 1148, 1053, 1001, 953, 742, 684 cm-1. **HRMS** (ESI+): Calculated for $C_{21}H_{28}NaO_3$ (M+Na)+: 351.1931; Found 351.1934. $[\alpha]_D^{25}$ = + 1.6° (*c* = 0.5, $CHCl_3$)

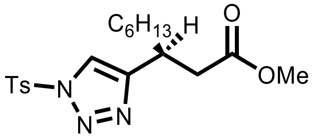

methyl *(S)*-3-(1-tosyl-1H-1,2,3-triazol-4-yl)nonanoate (**41**):

This procedure was adapted from the literature.[24] An oven-dried Biotage® microwave vial (0.5 – 2.0 mL size) equipped with a stir bar was charged with methyl *(S)*-3-ethynylnonanoate (47.1 mg, 0.24 mmol, 1.2 equivalents). The vial was sealed with a septum and evacuated and backfilled with N_2 three times. Distilled, degassed (N_2 sparged) PhMe (1.0 mL) was added, followed by copper(I)thiophene-2-carboxylate (3.8 mg, 0.02 mmol, 10 mol %). To the homogeneous, light yellow solution was added tosyl azide (39.5 mg, 0.20 mmol, 1 equivalent) dropwise, using a tared microliter syringe. Within 20 min, the appearance changed to bright green and homogeneous. After 1 h, the reaction mixture was applied directly to a silica gel column that was eluted with 4:1 hexanes:EtOAc to deliver the title compound (78.0 mg, 99%) as a viscous, clear oil. $\mathbf{R_f}$ = 0.40 (2:1 hexanes:EtOAc). ^{1}**H NMR** (300 MHz, $CDCl_3$) δ 7.98-7.94 (m, 2H), 7.89 (s, 1H), 7.38-7.35 (m, 2H), 3.57 (s, 3H), 3.35-3.25 (m, 1H), 2.71 (dd, *J* = 15.2, 7.0 Hz, 1H), 2.64 (dd, *J* = 15.2, 5.8 Hz, 1H), 2.43 (s, 3H), 1.74-1.59 (m, 2H), 1.25-1.11 (m, 8H), 0.83 (t, *J* = 6.8 Hz, 3H). ^{13}**C NMR** (75 MHz, $CDCl_3$) δ 172.4, 150.0, 147.2, 133.3, 130.5, 128.7, 120.8, 51.7, 39.1, 34.4, 33.1, 31.7, 29.1, 27.0, 22.6, 22.0, 14.2. **IR** (film) = 3103, 2887, 2817, 1713, 1573, 1529, 1417, 1375, 1277, 1178, 1162, 1077, 995, 959 cm^{-1}. **HRMS**: Calculated for $C_{19}H_{28}N_3O_4S$ $(M+H)^+$: 394.1795, Found 394.1799. $[\alpha]_D^{23}$ = – 0.33° (*c* = 0.67, $CHCl_3$).

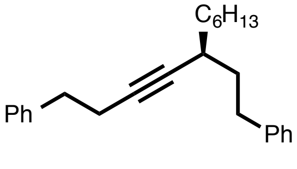

(S)-(5-hexylhept-3-yne-1,7-diyl)dibenzene (**44**):

This procedure was adapted from the literature.[25] To a homogeneous, light yellow solution of (R)-((2-(4-phenylbut-1-yn-1-yl)octyl)sulfonyl)benzene (45.9 mg, 0.12 mmol, 1 equivalent) in THF (0.48 mL) at – 78 °C was added n-butyllithium (2.3 M in hexanes, 55 μL, 0.126 mmol, 1.05 equivalents). The resulting homogeneous, red-brown mixture was stirred at – 78 °C for 15 min, and then a solution of benzyl bromide (17 μL, 0.144 mmol, 1.2 equivalents) in THF (0.48 mL) was added. The cooling bath was removed, and the resulting homogeneous, brown mixture was allowed to warm to room temperature. After 30 minutes, the reaction mixture was diluted with Et_2O (3 mL), and then it was quenched with 2 N HCl (3 mL). The phases were separated, and the aqueous phase was extracted with Et_2O (3 x 3 mL). The pooled organic phases were dried over $MgSO_4$ and filtered through a pipette plug of Florisil®. The filtrate was concentrated.

This crude material (R_f = 0.48, 4:1 hexanes:EtOAc) was dissolved in a mixture of freshly distilled MeOH (0.80 mL) and THF (0.40 mL).[26] To the resulting solution was added, first, Na_2HPO_4 (68.1 mg, 0.48 mmol, 4 equivalents) and then sodium-mercury amalgam (55 mg, spherical pellets, 20% sodium by weight, Strem Chemicals, Inc.; corresponds to *ca*. 11 mg of sodium, 0.48 mmol, 4 equivalents). The resulting heterogeneous, orange mixture was stirred at room temperature for 2 h, at which point it was diluted with Et_2O (5 mL) and water (5 mL). The phases were separated, and the aqueous phase was extracted with Et_2O (3 x 5 mL). The pooled organic phases were dried over $MgSO_4$ and filtered through a pipette plug of Florisil®. The filtrate was concentrated, and the crude material was purified by column chromatography (10:1 hexanes:EtOAc) to afford the title compound (33.2 mg, 83%) as a viscous, clear yellow oil. **R_f** = 0.92 (4:1 hexanes:EtOAc); 0.80 (10:1 hexanes:EtOAc). **^{1}H NMR** (500 MHz, $CDCl_3$) δ 7.32-7.27 (m, 5H), 7.25-7.17 (m, 5H), 2.85 (t, J = 7.5 Hz, 2H), 2.84-2.77 (m, 1H), 2.67-2.61 (m, 1H), 2.53 (td, J = 7.5, 2.2 Hz, 2H), 2.31-2.25 (m, 1H), 1.71-1.67 (m, 2H), 1.43-1.38 (m, 2H), 1.31-1.25 (m, 8H), 0.90 (t, J = 7.0 Hz, 3H). **^{13}C NMR** (125 MHz, $CDCl_3$) δ 142.6, 141.1, 128.7, 128.6, 128.41, 128.39, 126.7, 125.8, 84.3, 81.2, 37.4, 35.8, 35.6, 33.8, 32.0, 31.5, 29.4, 27.5, 22.8, 21.1, 14.3. **IR** (film) = 3042, 3020, 2886, 2816,

1581, 1475, 1434, 1358, 1324, 736, 688 cm^{-1}. **HRMS**: Calculated for $C_{25}H_{32}Na$ (M+Na) 355.2396; Found 355.2394. $[\alpha]_D^{24}$ = + 20.44° (*c* = 0.85, $CHCl_3$).

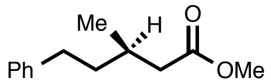

methyl (*R*)-3-methyl-5-phenylpentanoate (**45**):

To a 5 mL conical vial equipped with a stir bar was added a solution of **9bf** (31.0 mg, 0.153 mmol, 1 equivalent) in EtOAc (0.55 mL). Palladium on carbon (2.0 mg, 5 weight percent dry basis, wet, 6 weight percent) was added, and the vial was sealed with a septum and purged with hydrogen for 5 min (balloon). The reaction mixture was stirred at room temperature under a hydrogen atmosphere (balloon) for 24 h. The reaction mixture was then diluted with EtOAc (1 mL) and filtered through a small pipette plug of Florisil, eluting with EtOAc (10 mL). The resulting solution was concentrated to afford the title compound as a clear oil (30.2 mg, 0.146 mmol, 96% yield). R_f = 0.45 (10/1 Hexanes/EtOAc). **^{1}H NMR** (300 MHz, $CDCl_3$) δ 7.31-7.26 (m, 2H), δ 7.20-7.15 (m, 3H), 3.67 (s, 3H), 2.71-2.53 (tdt, *J* = 13.9 Hz, 10.2, 7.0 Hz, 2H), 2.40-2.33 (dd, *J* = 14.7 Hz, 6.1 Hz, 1H), 2.22-2.14 (dd, *J* = 14.7 Hz, 8.0 Hz, 1H), 2.10-1.94 (m, 1H), 1.73-1.45 (m, 2H), 1.02 (d, *J* = 6.6 Hz, 3H). **^{13}C NMR** (125 MHz, $CDCl_3$) δ 173.7, 142.4, 128.5, 128.4, 125.9, 51.4, 41.7, 38.7, 33.4, 30.2, 19.8. **IR** (film) 3027, 2953, 2931, 2857, 1738, 1496, 1455, 1436, 1367, 1304, 1260, 1201, 1157, 1090, 1010, 748, 699 cm^{-1}. **HRMS:** Calculated for $C_{13}H_{19}O_2$ $(M+H)^+$: 207.1380; Found 207.1372. $[\alpha]_D^{25}$ = +18.50° (c = 0.88; CH_2Cl_2).

---

[1] Pearson, D. L.; Tour, J. M. *J. Org. Chem.* **1997**, *62,* 1376–1387.
[2] Trost, B. M.; Frontier, A. J.; Thiel, O. R.; Yang, H.; Dong, G. *Chem. - Eur. J.* **2011**, *17*, 9762–9776.
[3] McConnell, O. J.; Fenical, W. *Phytochemistry*, **1980**, *19*, 233–247.
[4] Kwong, C. K.-W., Fu, M. Y., Law, H. C.-H., Toy, P. H. *Synlett*. **2010**, *17*, 2617.
[5] Trost, B. M.; Shen, H. C.; Horne, D. B.; Toste, F. D.; Steinmetz, B. G.; Koradin, C. *Chem. - Eur. J.* **2005**, *11*, 2577–2590.
[6] Dieter, R. K.; Lu, K. *J. Org. Chem.* **2002,** *67,* 847–855.

[7] Trost, B. M.; Sorum, M. T.; Chan, C.; Harms, A. E.; Rühter, G. *J. Am. Chem. Soc.* **1997**, *119*, 698–708.
[8] Marino, J. P.; McClure, M. S.; Holub, D. P.; Comasseto, J. V.; Tucci, F. C. *J. Am. Chem. Soc.* **2002**, *124*, 1664–1668.
[9] David, O.; Vanucci-Bacqué, C.; Fargeau-Bellassoued, M.-C.; Lhommet, G. *Heterocycles*, **2004**, *62*, 839.
[10] Trost, B. M.; Lumb, J.-P.; Azzarelli, J. M. *J. Am. Chem. Soc.* **2011**, *133*, 740–743.
[11] (a) Shimada, T.; Ueno, H.; Tsutsumi, K.; Aoyagi, K.; Manabe, T.; Sasaki, S.-Y.; Katoh, S. Spiro compounds and pharmaceutical use thereof. US 8299296, October 30, **2012**; (b) Lin, D. C.-H., *et al.*, *PLoS ONE* **2011**, *6*, e27270 and references therein; (c) Houze, J. B.; Zhu, L.; Sun, Y.; Akerman, M.; Qiu, W.; Zhang, A. J.; Sharma, R.; Schmitt, M.; Wang, Y.; Liu, J.; Liu, J.; Medina, J. C.; Reagan, J. D.; Luo, J.; Tonn, G.; Zhang, J.; Lu, J. Y.-L.; Chen, M.; Lopez, E.; Nguyen, K.; Yang, L.; Tang, L.; Tian, H.; Shuttleworth, S. J.; Lin, D. C.-H. *Bioorg. Med. Chem. Lett.* **2012**, *22*, 1267–1270; (d) Bharate, S. B.; Nemmani, K. V. S.; Vishwakarma, R. A. *Expert Opin. Ther. Pat.* **2009**, *19*, 237 and references therein.
[12] Walker, S. D.; Borths, C. J.; DiVirgilio, E.; Huang, L.; Liu, P.; Morrison, H.; Sugi, K.; Tanaka, M.; Woo, J. C. S.; Faul, M. M. *Org. Process Res. Dev.* **2011**, *15*, 570–580.
[13] Ishikawa, M.; Haketa, T.; Nakama, C.; Nishimura, T.; Shibata, J.; Shimamura, T.; Yamakawa, T. US201165739 A1. March 17, **2011**.
[14] Hamilton, J. Y.; Sarlah, D.; Carreira, E. M. *Angew. Chem. Int. Ed.* **2013**, *52*, 7532–7535.
[15] (a) Yazaki, R.; Kumagai, N.; Shibasaki, M. *Org. Lett.* **2011**, *13*, 952–955.
[16] Li, P.; Fong, W. M.; Chao, L. C. F.; Fung, S. H. C.; Williams, I. D. *J. Org. Chem.*, **2001**, *66*, 4087–4090.
[17] Van den Hoven, B. G.; Ali, B. E.; Alper, H. *J. Org. Chem.* **2000**, *65*, 4131–4137.
[18] Riddell, N.; Tam, W. *J. Org. Chem.*, **2006**, *71*, 1934–1937,
[19] Otera, J.; Danoh, N.; Nozaki, H. *J. Org. Chem.* **1991**, *56*, 5307–5311.
[20] Belting, V.; Krause, N. *Org. Lett.* **2006**, *8*, 4489–4492.
[21] Shen Z, Lu X. *Tetrahedron* **2006**, *62*, 10896–10899.
[22] Lambert, C.; Utimoto, K.; Nozaki, H. *Tetrahedron Lett.*, **1984**, *25*, 5323–5326.
[23] Trost, B. M.; Bartlett, M. J.; Weiss, A. H.; von Wangelin, A. J.; Chan, V. S. *Chem. - Eur. J.* **2012**, *18*, 16498–16509.
[24] Raushel, J.; Fokin, V. V. *Org. Lett.* **2010**, *12*, 4952–4955.
[25] Serra, S. *Tetrahedron: Asymmetry* **2014**, *25*, 1561−1572.
[26] Desrosiers, J.-N.; Charette, A. B. *Angew. Chem. Int. Ed.* **2007**, *46*, 5955–5957

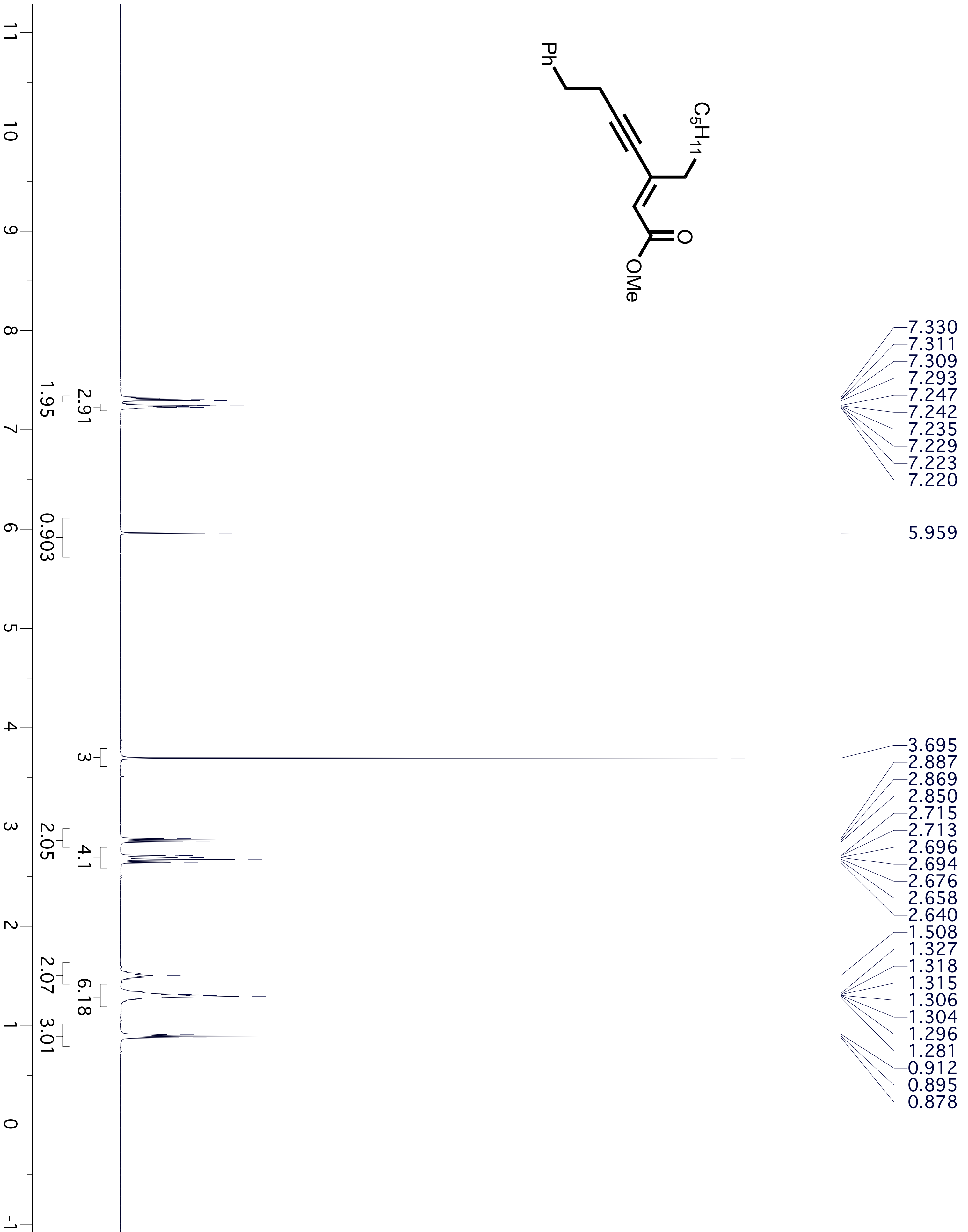
Ph
C5H11
O
OMe
7.330
7.311
7.309
7.293
7.247
7.242
7.235
7.229
7.223
7.220
5.959
3.695
2.887
2.869
2.850
2.715
2.713
2.696
2.694
2.676
2.658
2.640
1.508
1.327
1.318
1.315
1.306
1.304
1.296
1.281
0.912
0.895
0.878
1.95
2.91
0.903
3
2.05
4.1
2.07
6.18
3.01
11
10
9
8
7
6
5
4
3
2
1
0
-1

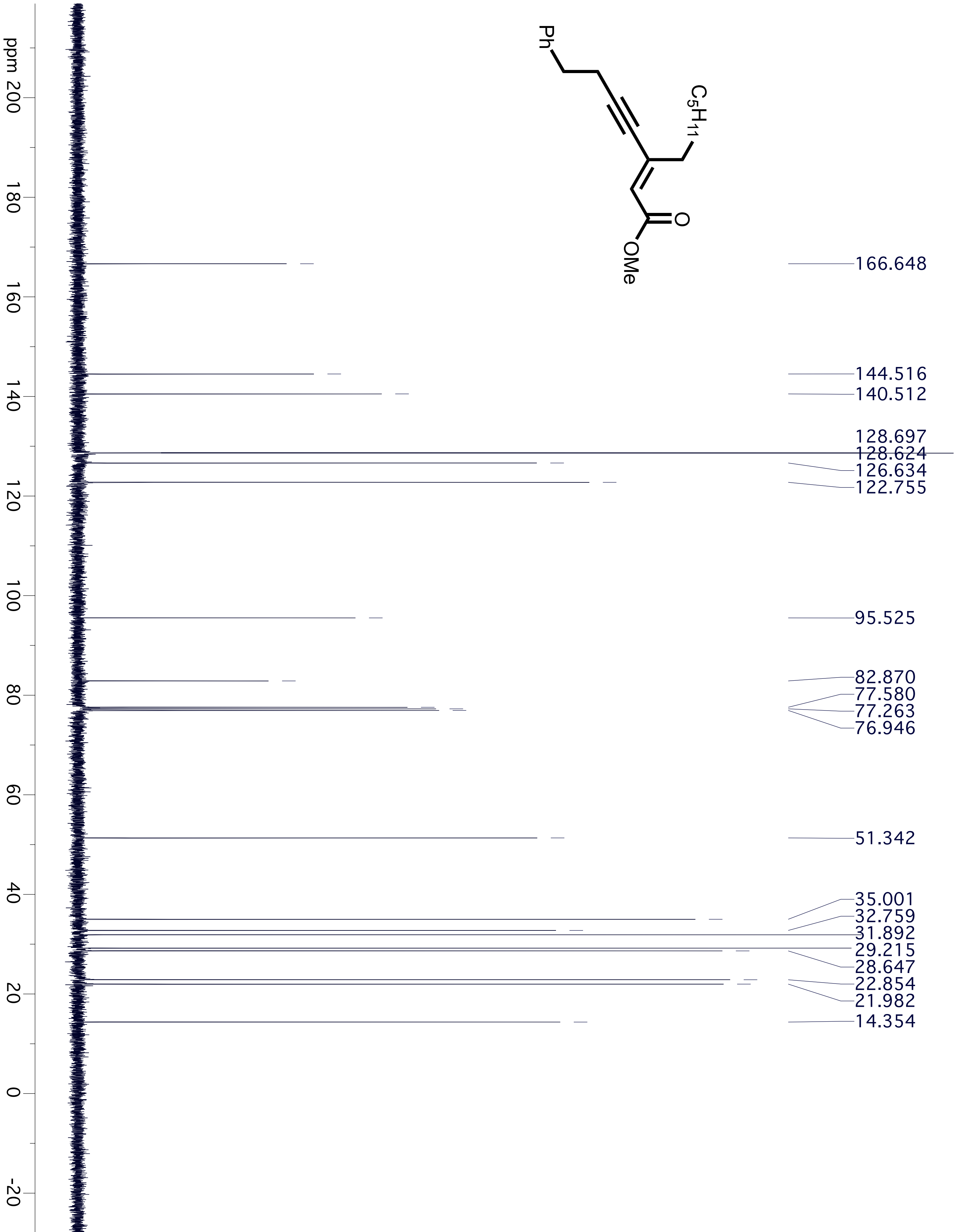
Ph
C5H11
O
OMe
166.648
144.516
140.512
128.697
128.624
126.634
122.755
95.525
82.870
77.580
77.263
76.946
51.342
35.001
32.759
31.892
29.215
28.647
22.854
21.982
14.354
ppm 200
180
160
140
120
100
80
60
40
20
0
-20

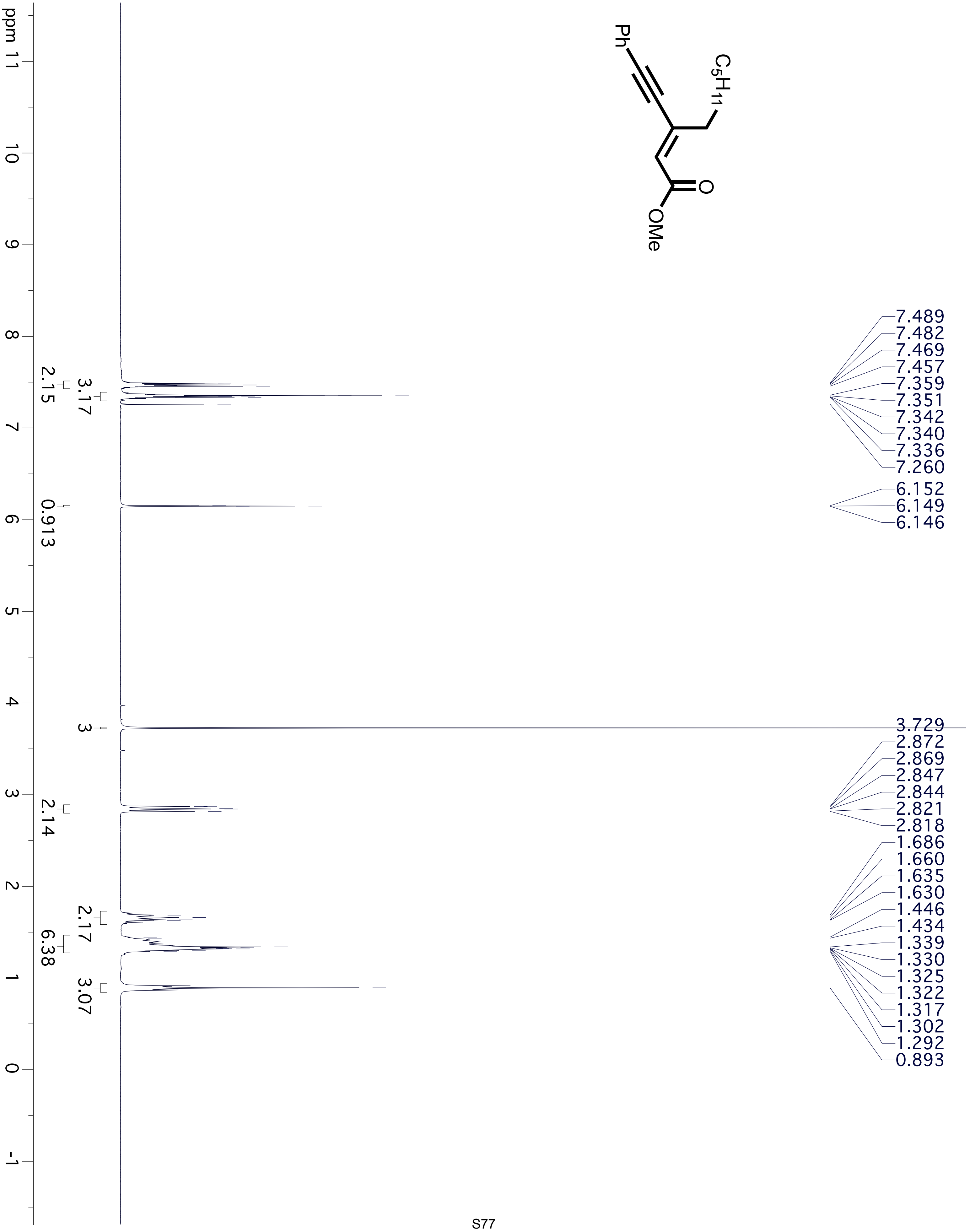
Ph
C5H11
O
OMe
ppm
11
10
9
8
7
6
5
4
3
2
1
0
-1
2.15
3.17
0.913
3
2.14
2.17
6.38
3.07
7.489
7.482
7.469
7.457
7.359
7.351
7.342
7.340
7.336
7.260
6.152
6.149
6.146
3.729
2.872
2.869
2.847
2.844
2.821
2.818
1.686
1.660
1.635
1.630
1.446
1.434
1.339
1.330
1.325
1.322
1.317
1.302
1.292
0.893

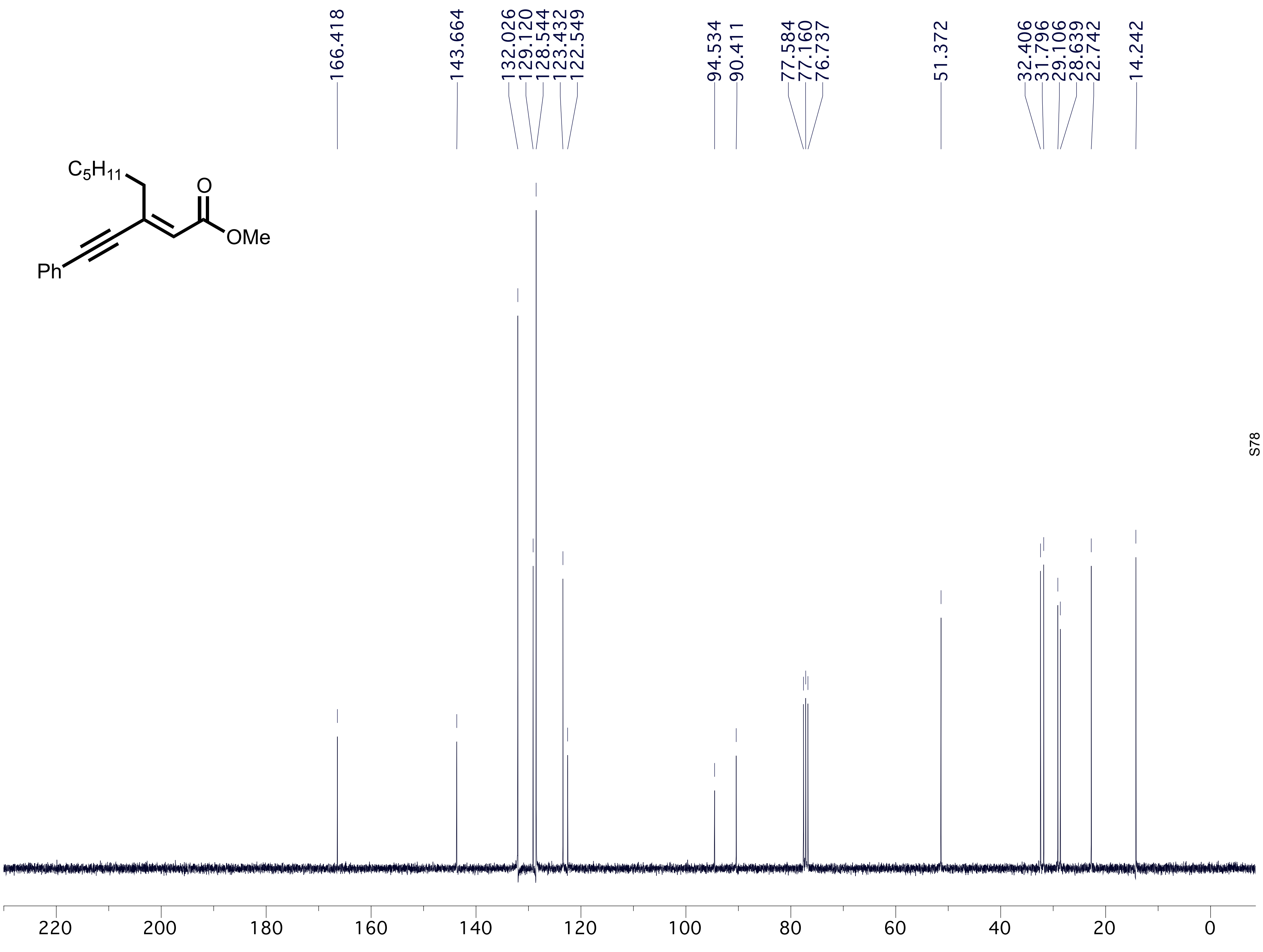
C5H11
O
OMe
Ph
166.418
143.664
132.026
129.120
128.544
123.432
122.549
94.534
90.411
77.584
77.160
76.737
51.372
32.406
31.796
29.106
28.639
22.742
14.242
220
200
180
160
140
120
100
80
60
40
20
0

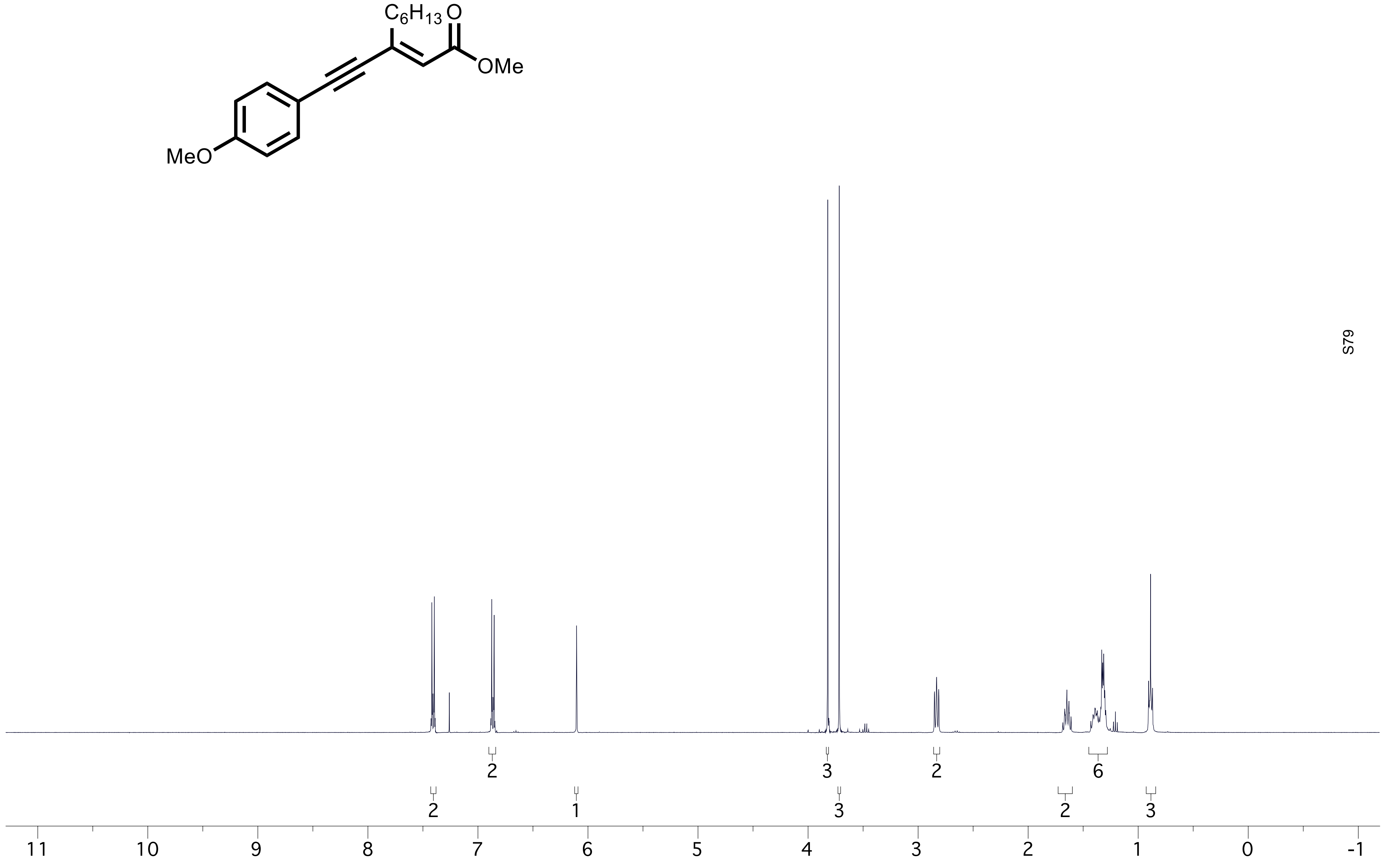

MeO
OMe
O
C6H13
2
2
1
3
3
2
6
2
3
11
10
9
8
7
6
5
4
3
2
1
0
-1

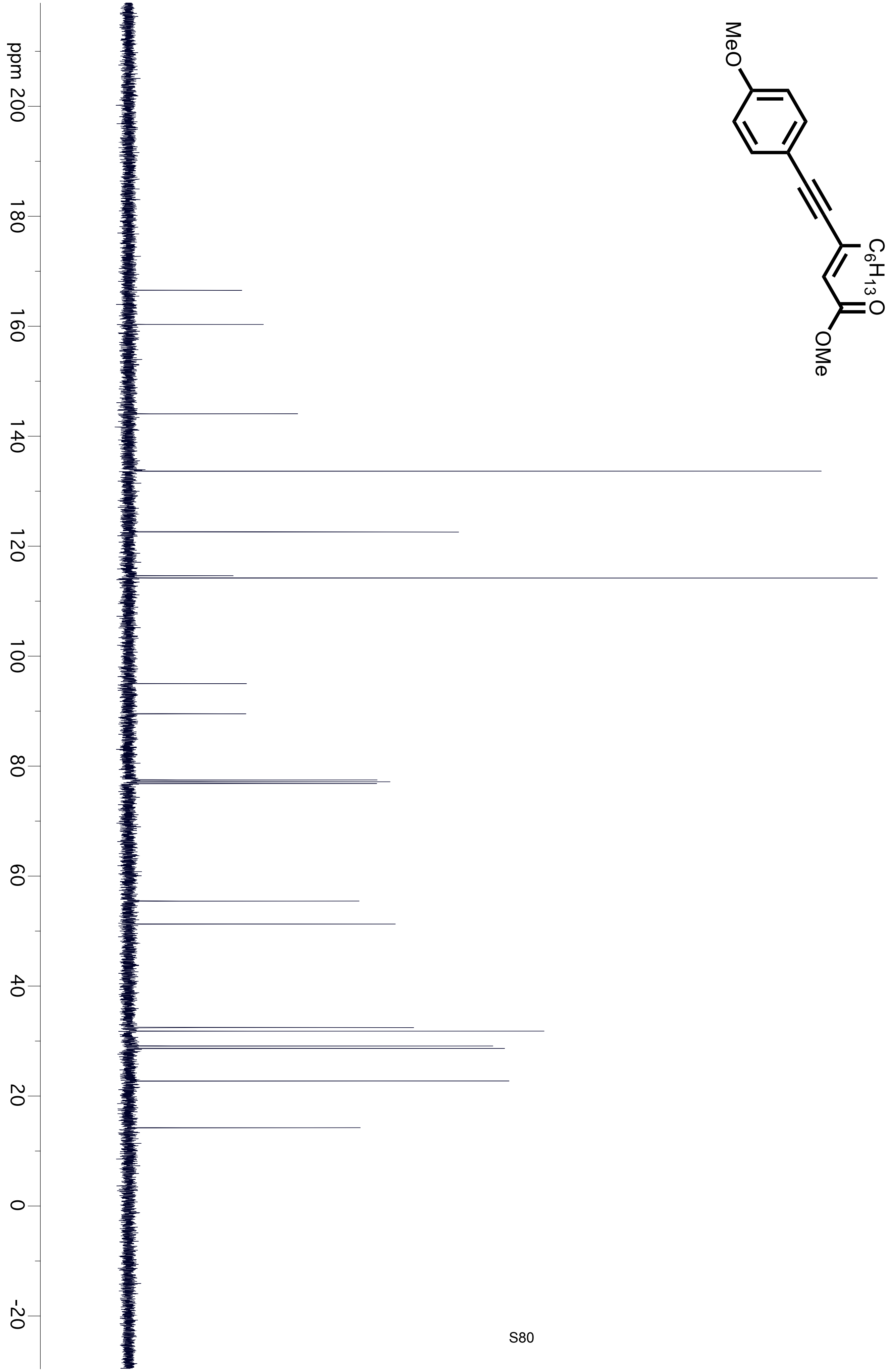
MeO
C6H13
O
OMe
ppm 200
180
160
140
120
100
80
60
40
20
0
-20

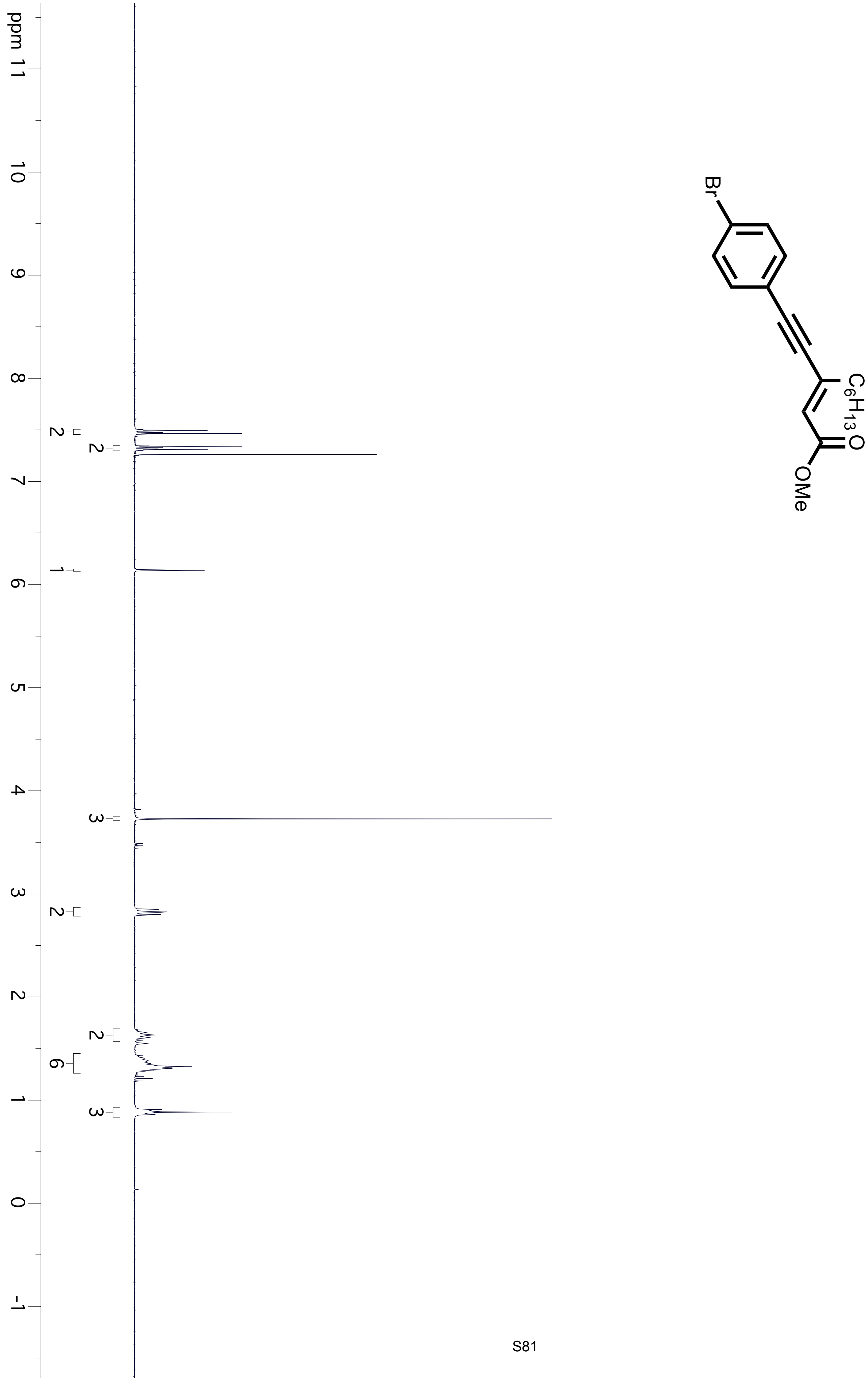

Br
C6H13
O
OMe
ppm
11
10
9
8
7
6
5
4
3
2
1
0
-1
2
2
1
3
2
2
6
3

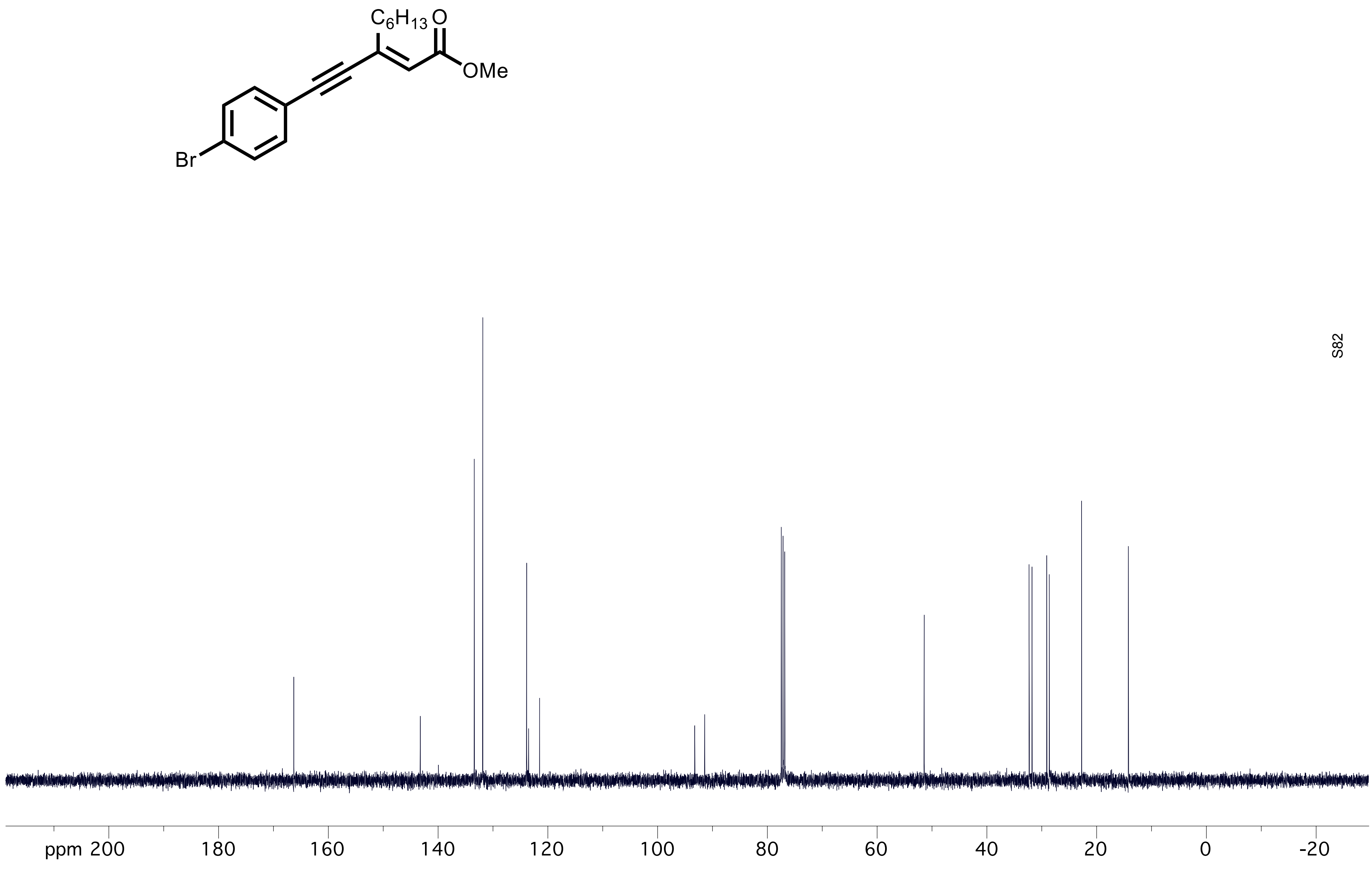

Br
C_6H_{13}
O
OMe
ppm 200
180
160
140
120
100
80
60
40
20
0
-20

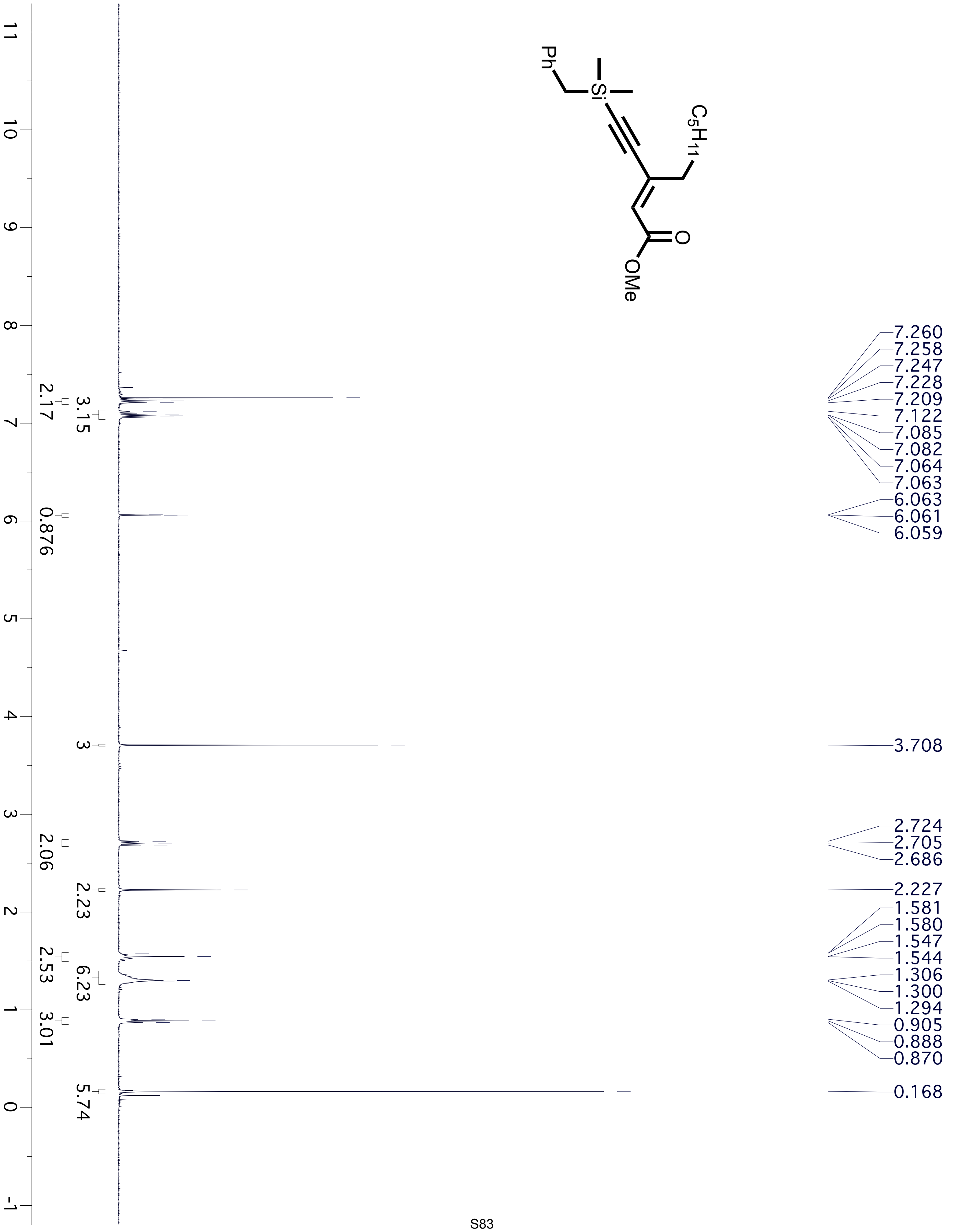
Ph
Si
C5H11
O
OMe
7.260
7.258
7.247
7.228
7.209
7.122
7.085
7.082
7.064
7.063
6.063
6.061
6.059
3.708
2.724
2.705
2.686
2.227
1.581
1.580
1.547
1.544
1.306
1.300
1.294
0.905
0.888
0.870
0.168
2.17
3.15
0.876
3
2.06
2.23
2.53
6.23
3.01
5.74
11
10
9
8
7
6
5
4
3
2
1
0
-1

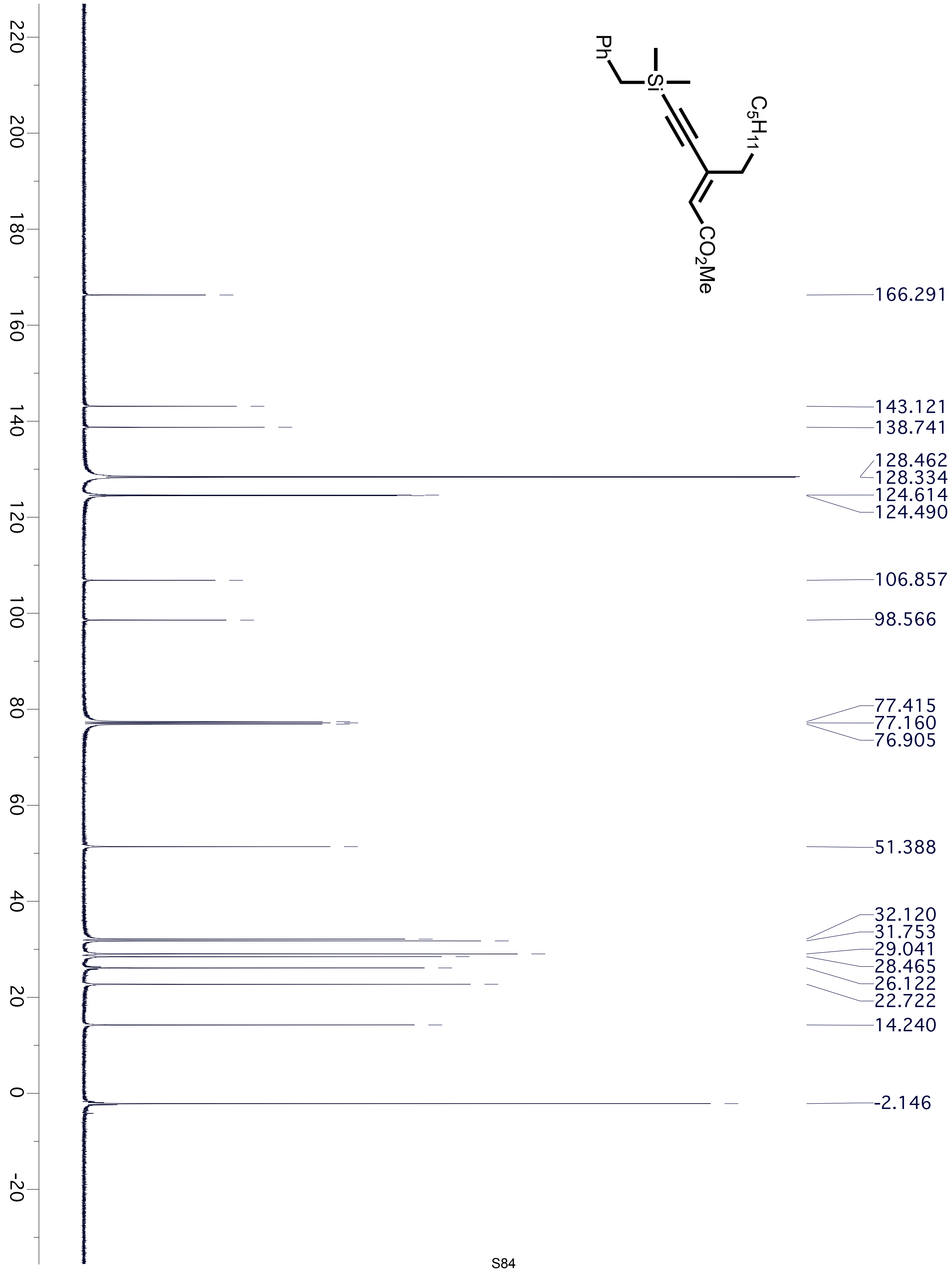
166.291
143.121
138.741
128.462
128.334
124.614
124.490
106.857
98.566
77.415
77.160
76.905
51.388
32.120
31.753
29.041
28.465
26.122
22.722
14.240
-2.146

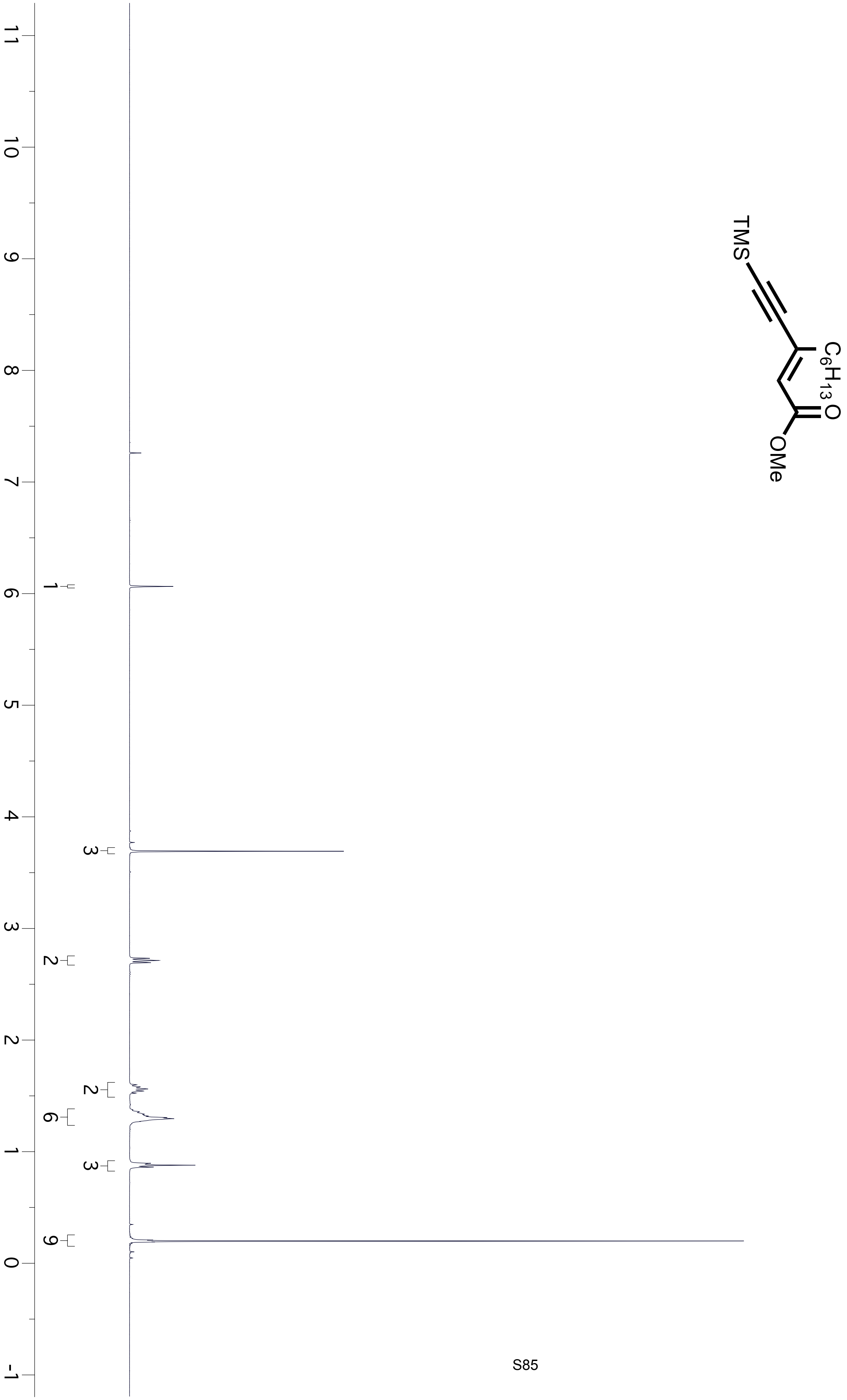
TMS
C_6H_{13}
O
OMe

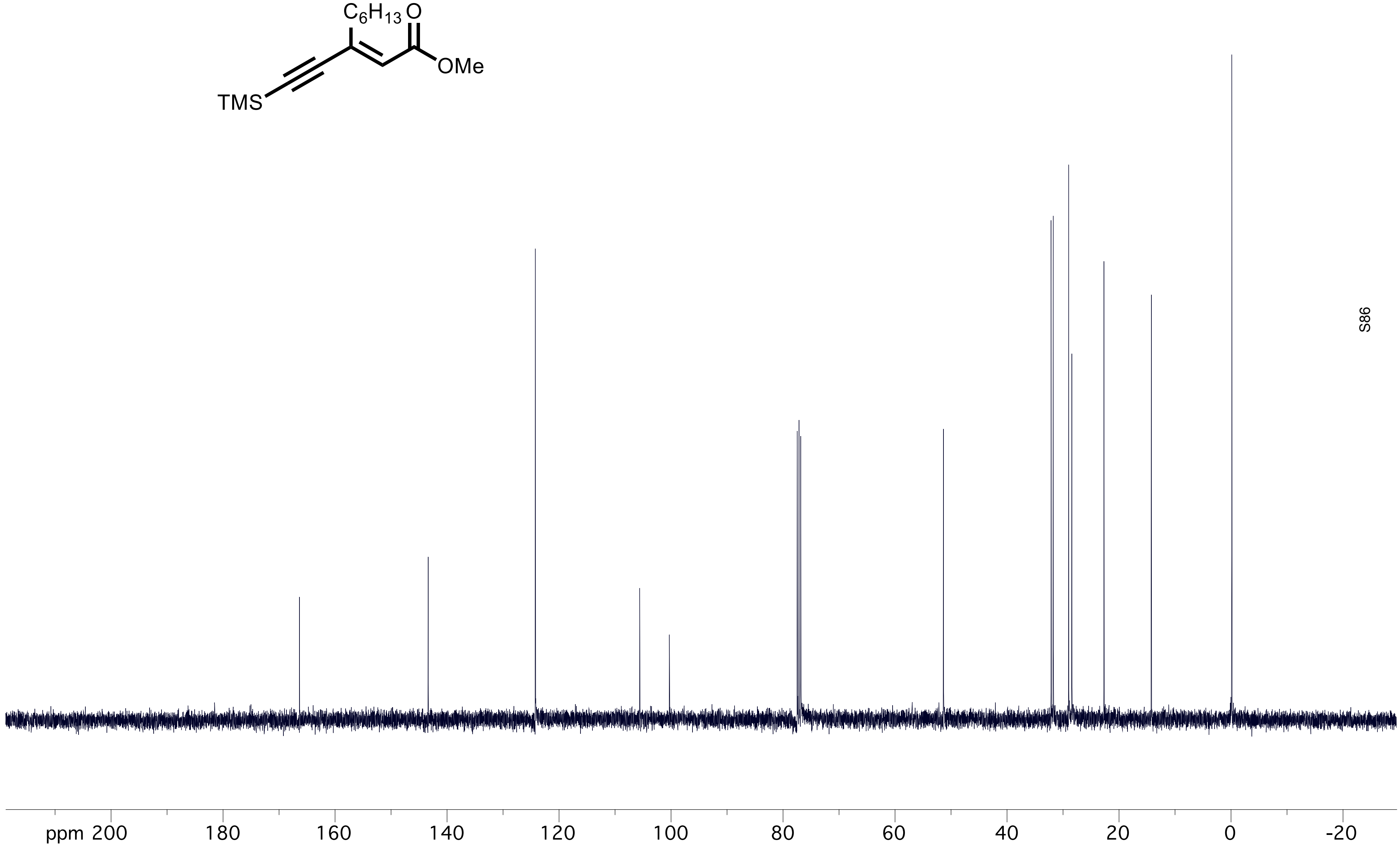

C_6H_{13}
O
OMe
TMS
ppm 200
180
160
140
120
100
80
60
40
20
0
-20

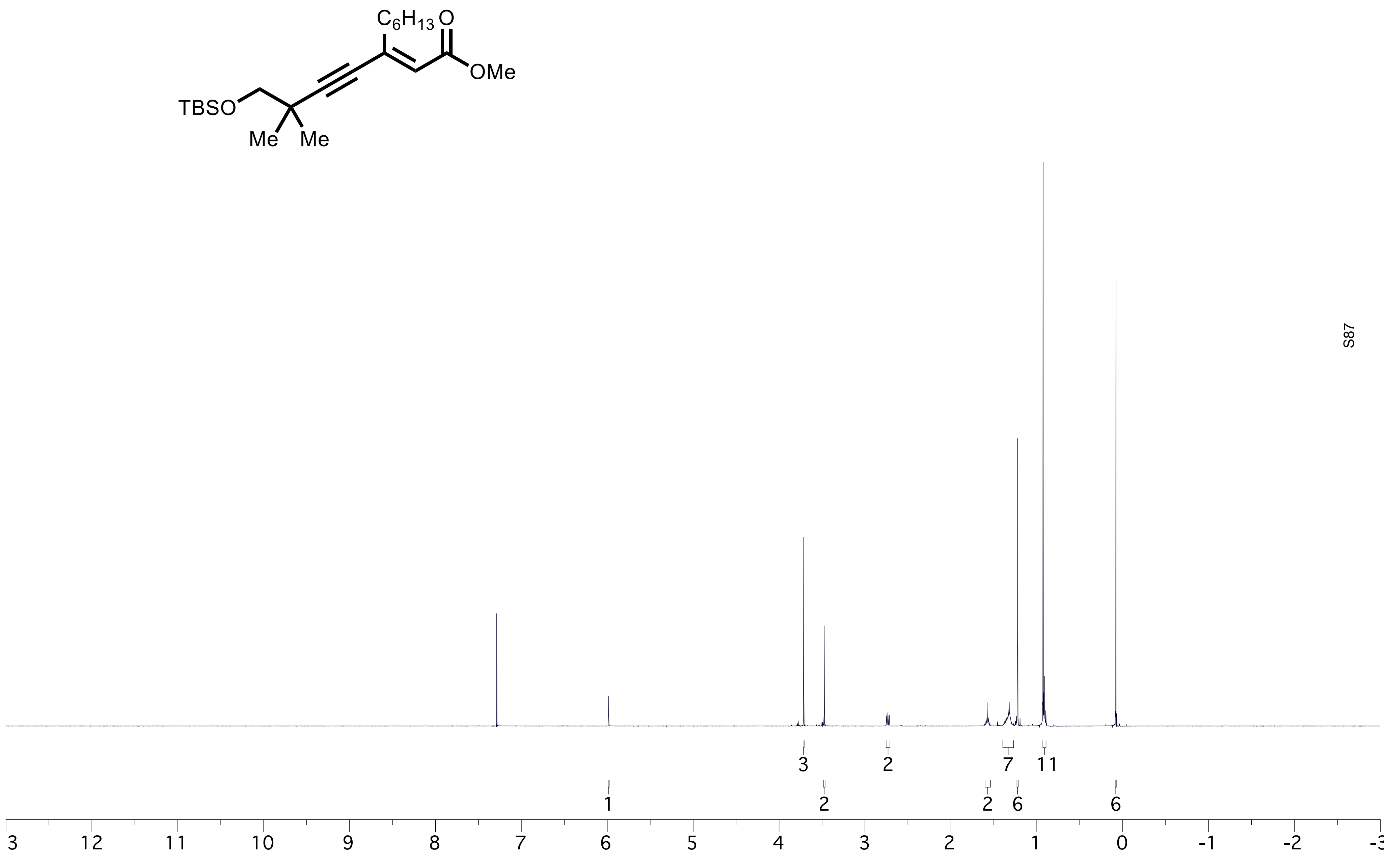

TBSO
Me
Me
C_6H_{13}
O
OMe
3
2
7
11
1
2
2
6
6
3
12
11
10
9
8
7
6
5
4
3
2
1
0
-1
-2

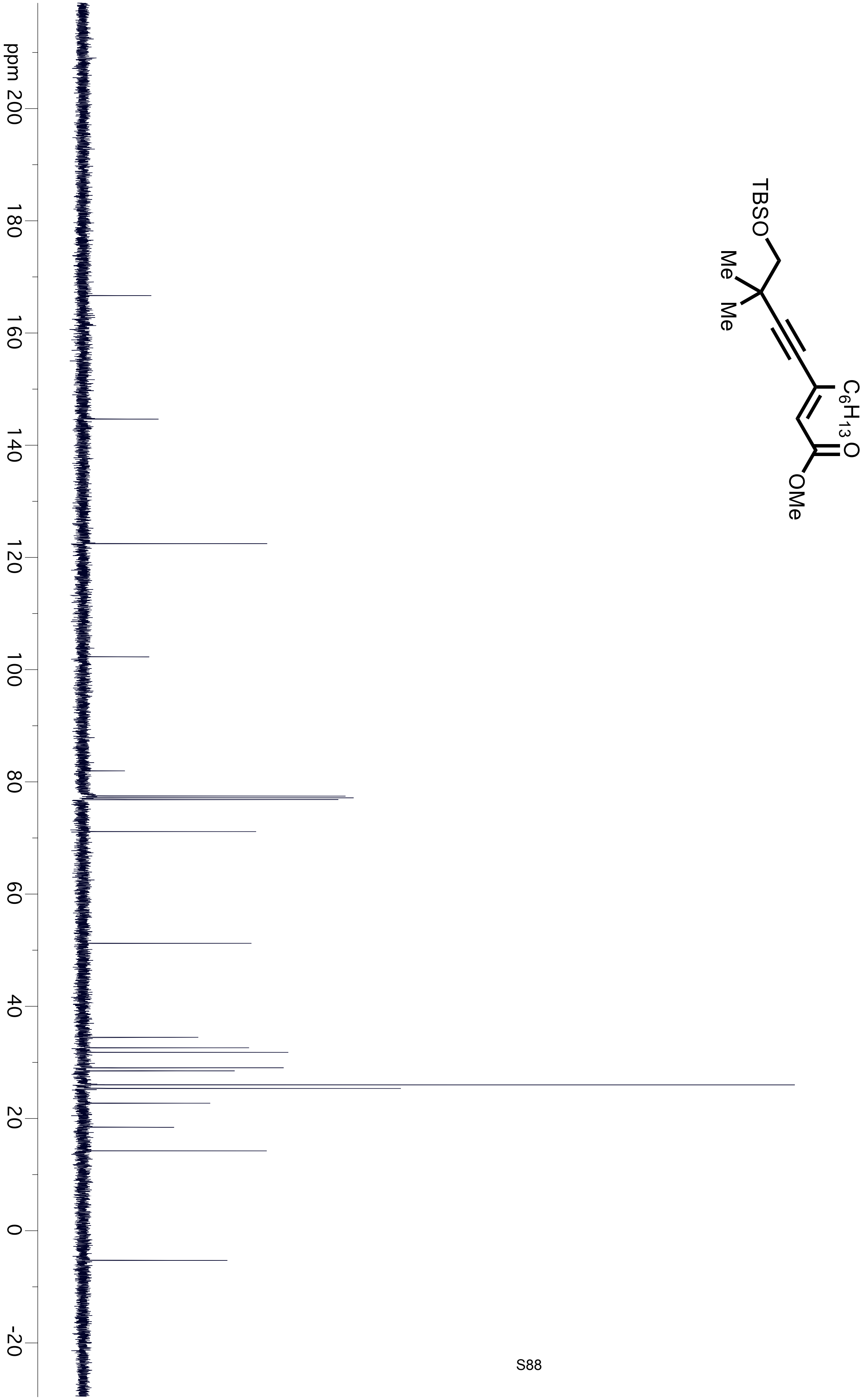
TBSO
Me
Me
C6H13
O
OMe
ppm 200
180
160
140
120
100
80
60
40
20
0
-20

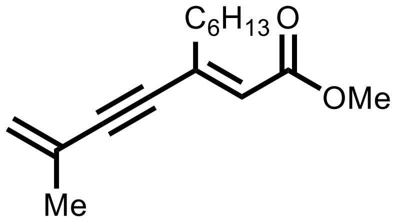
C6H13 O
OMe
Me

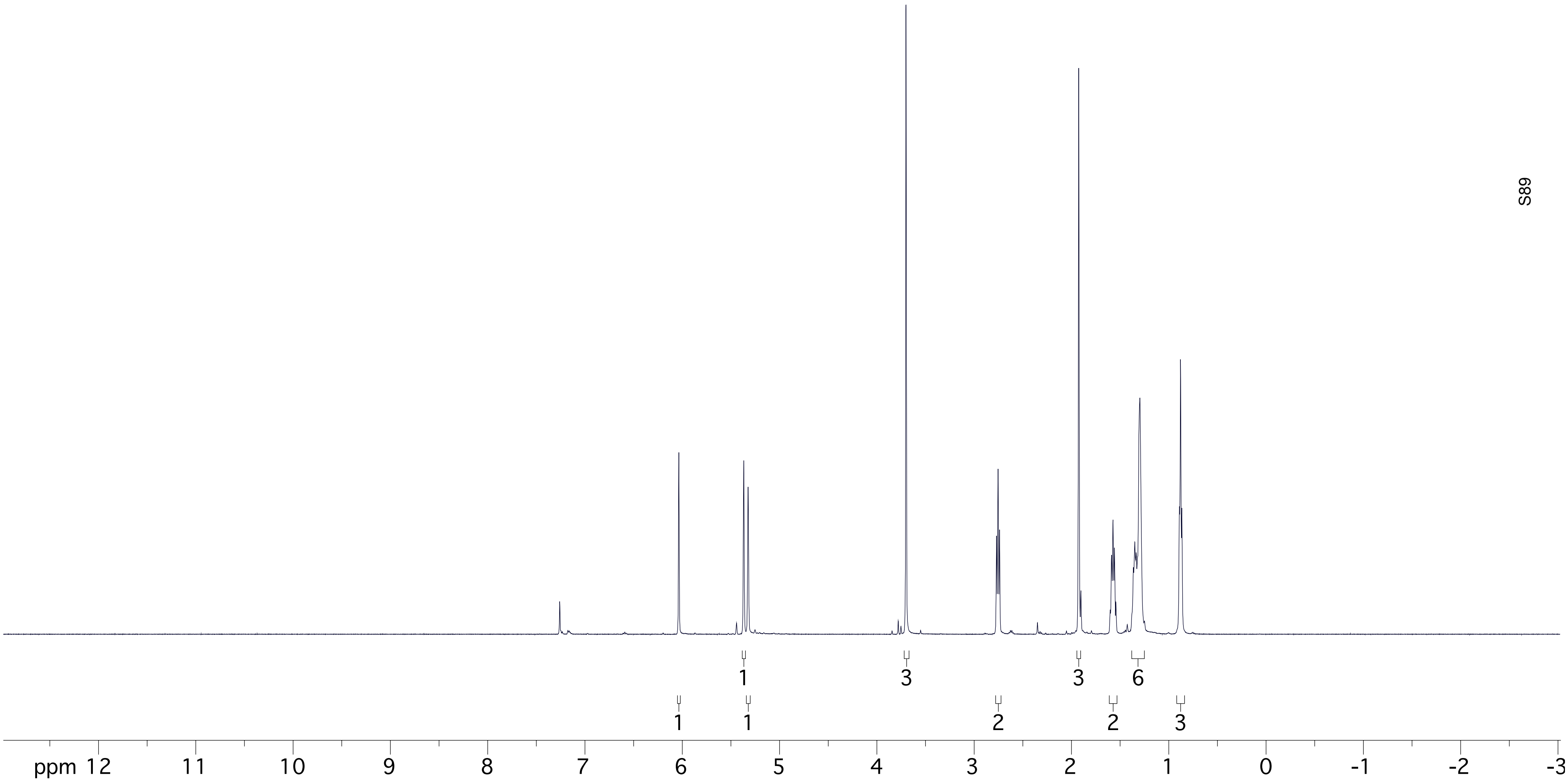
1 3 3 6
1 1 2 2 3
ppm 12 11 10 9 8 7 6 5 4 3 2 1 0 -1 -2 -3

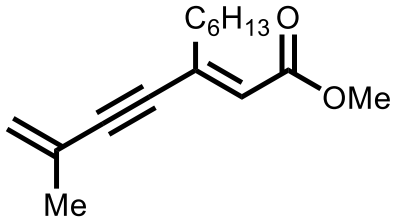

C6H13
O
OMe
Me

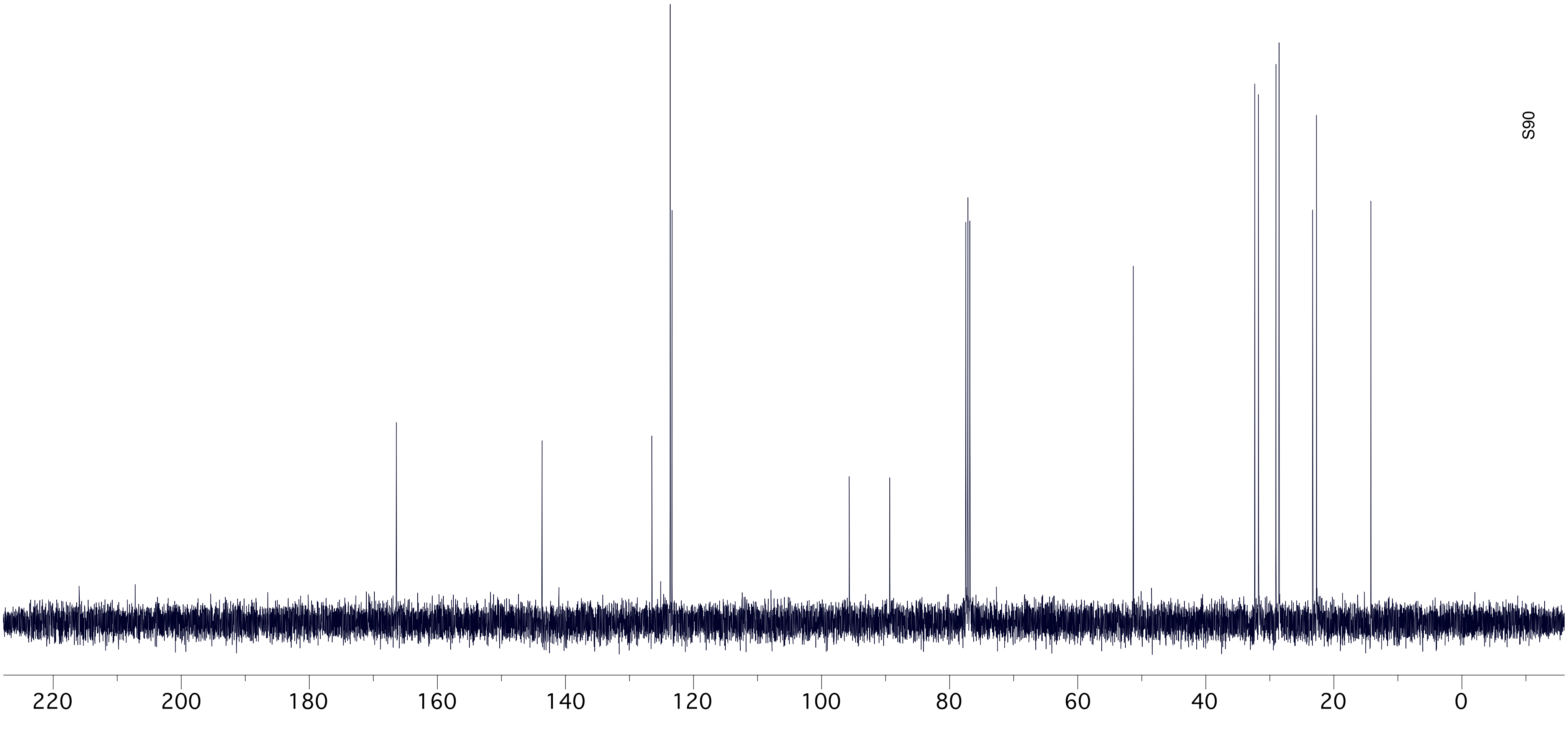

220
200
180
160
140
120
100
80
60
40
20
0

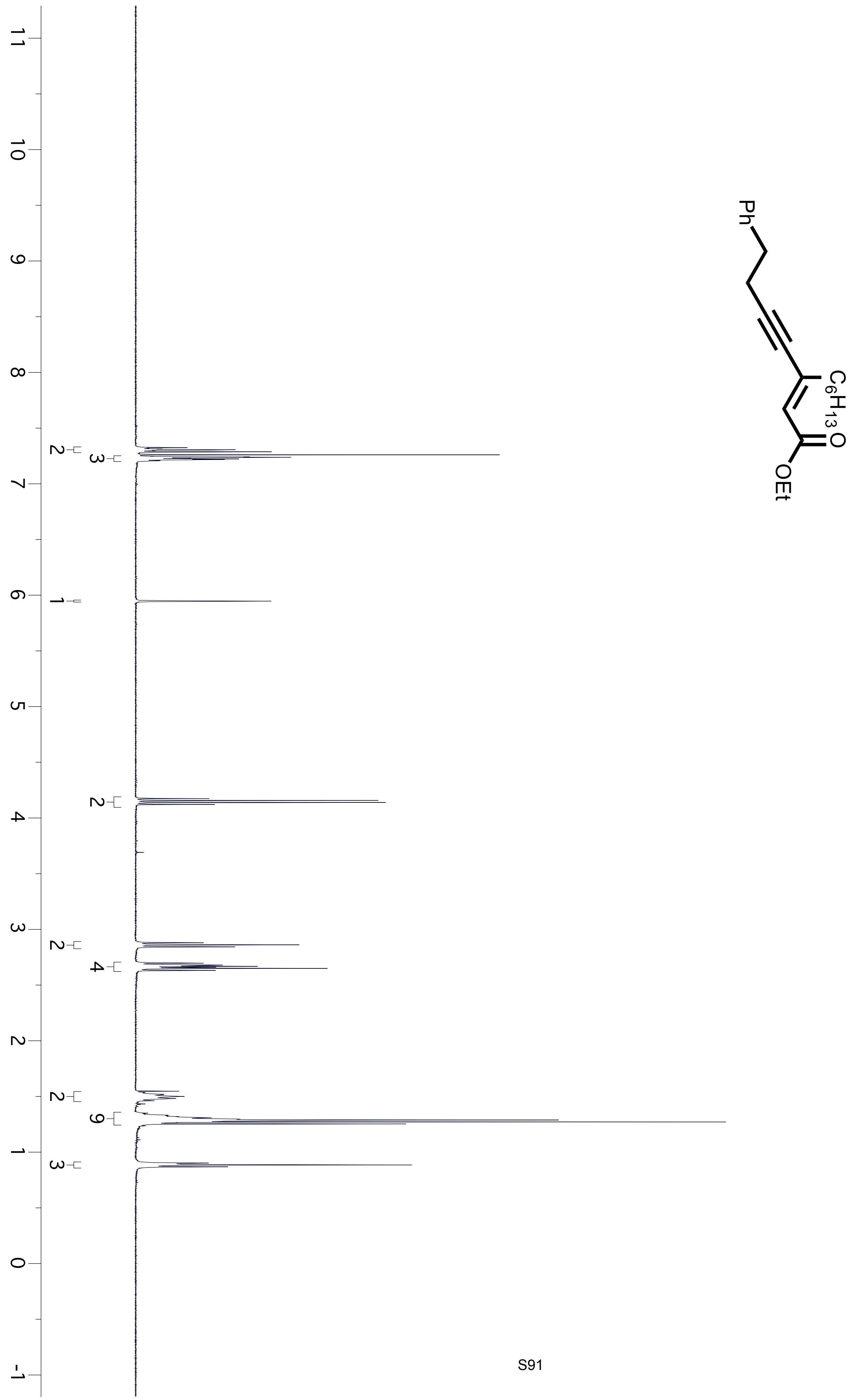
Ph
C6H13
O
OEt

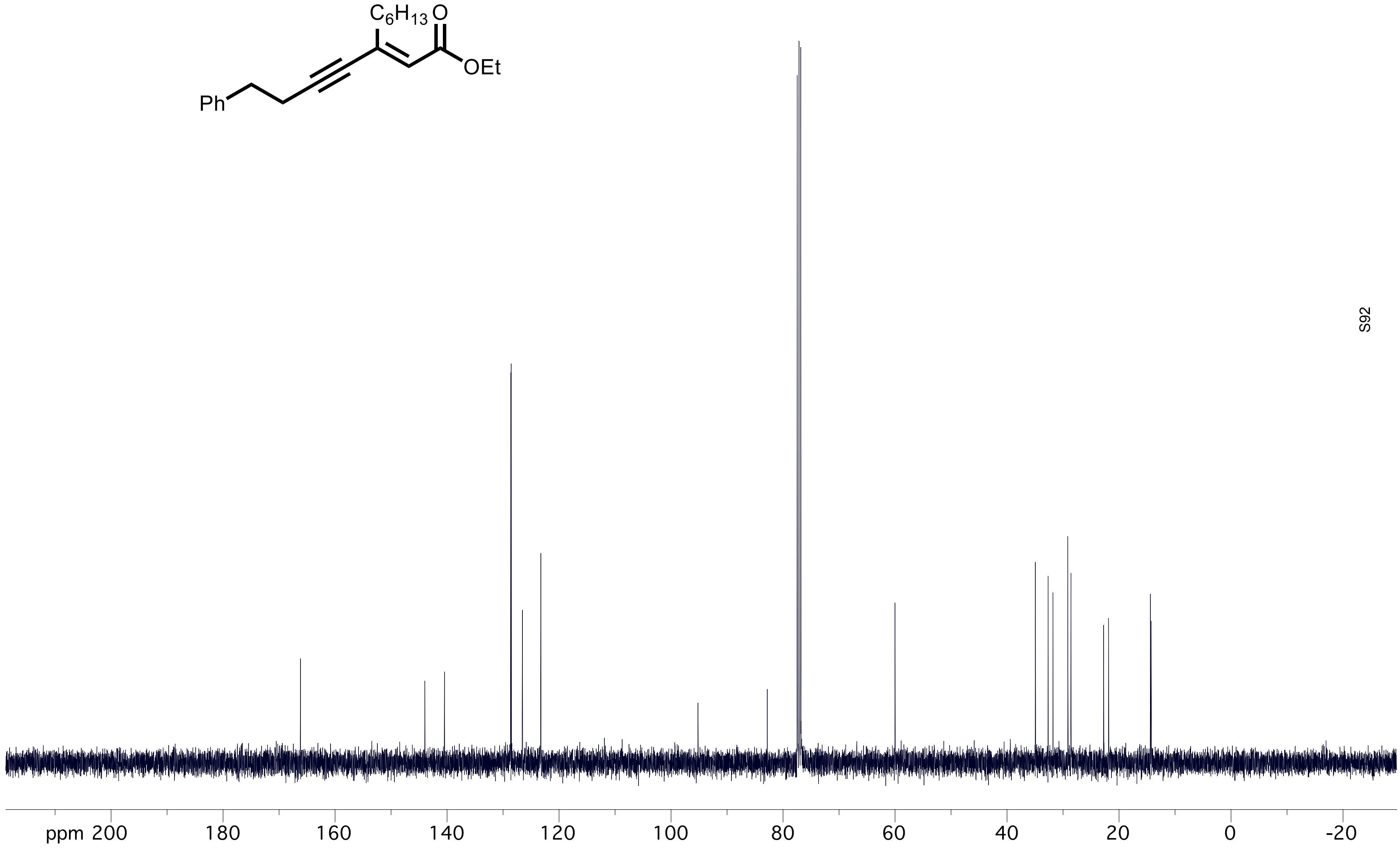
C6H13 O
OEt
Ph
ppm 200
180
160
140
120
100
80
60
40
20
0
-20

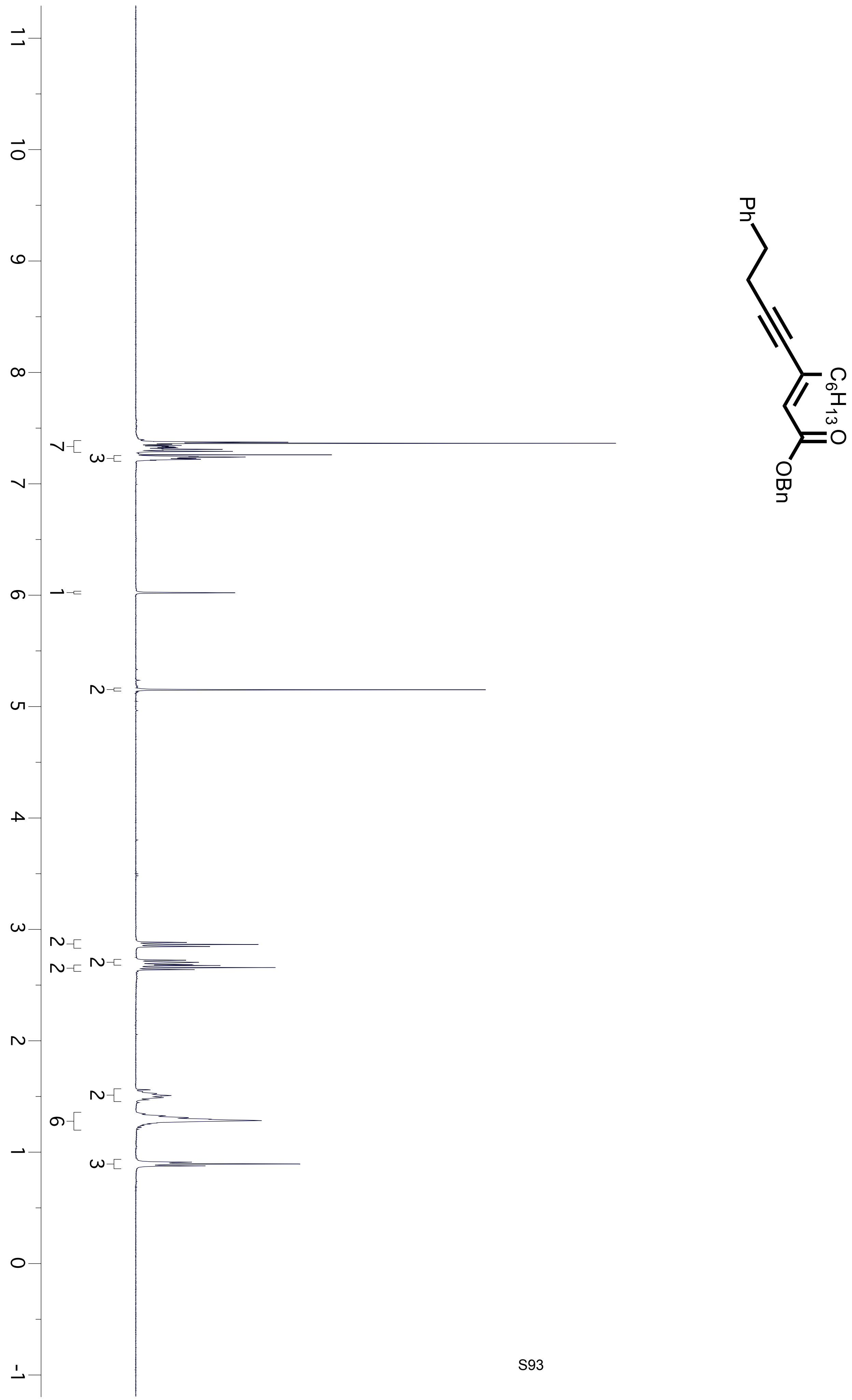
Ph
C6H13
O
OBn

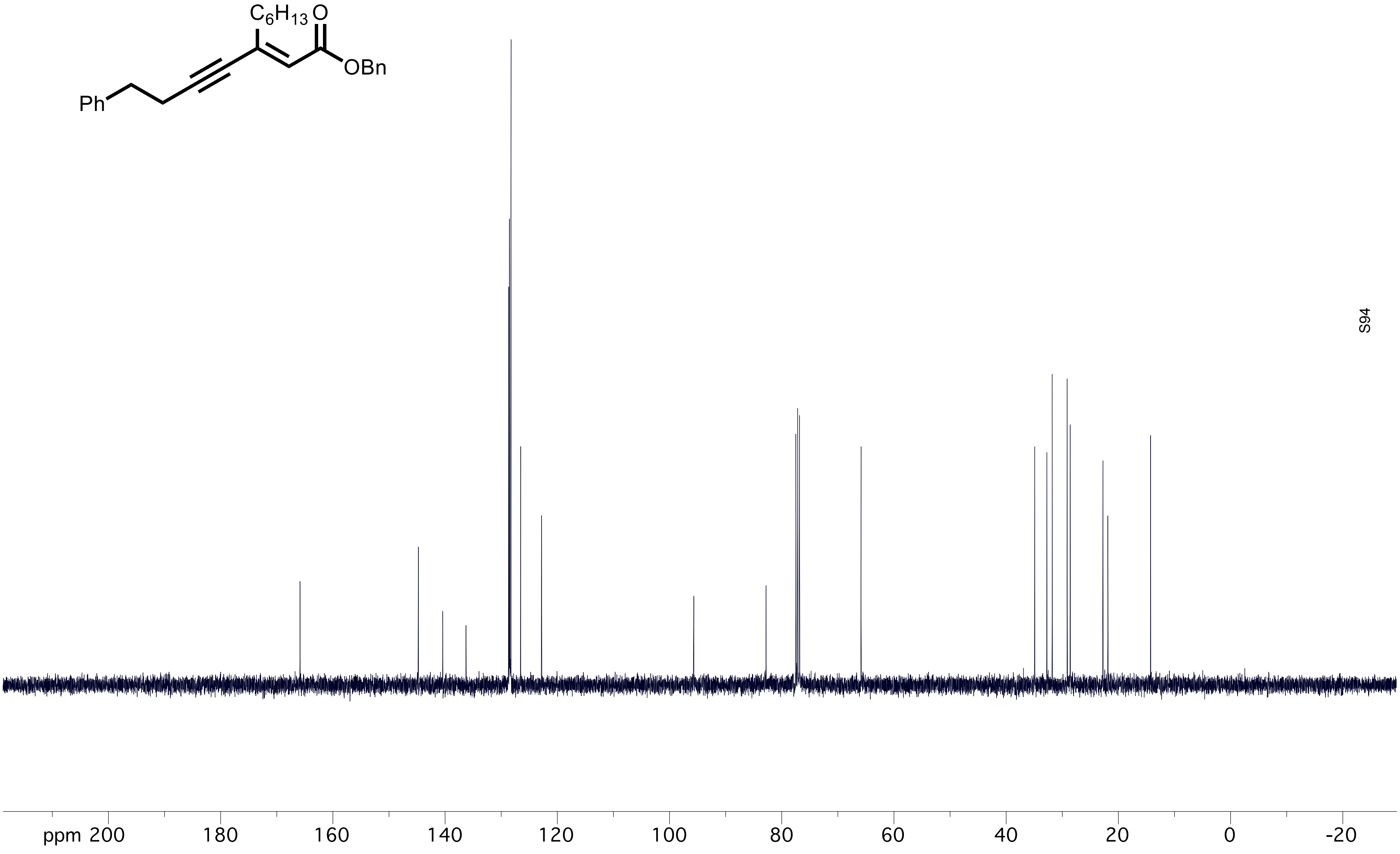
C6H13 O
OBn
Ph
ppm 200
180
160
140
120
100
80
60
40
20
0
-20

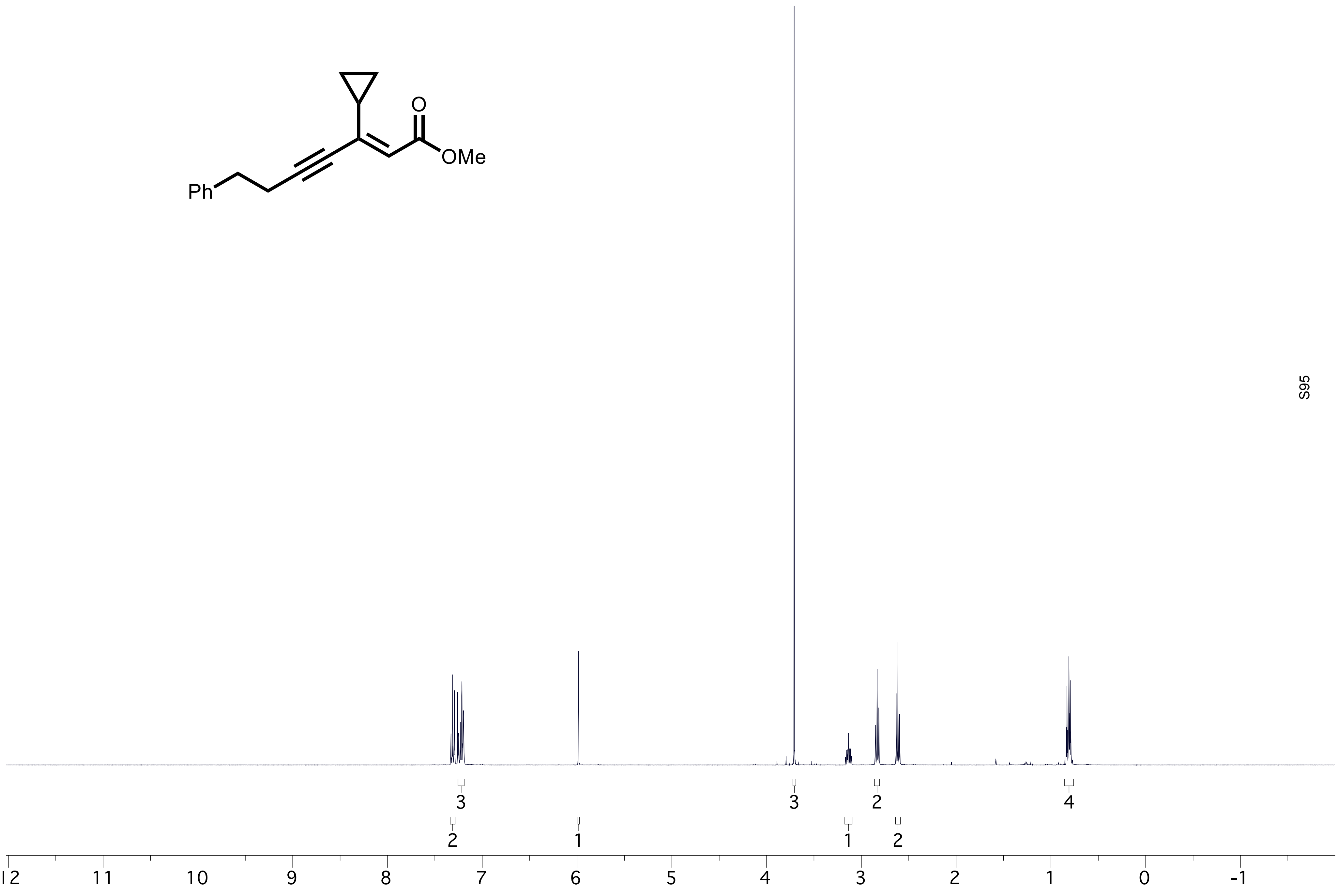

Ph
O
OMe
3
3
2
4
2
1
1
2
12
11
10
9
8
7
6
5
4
3
2
1
0
-1

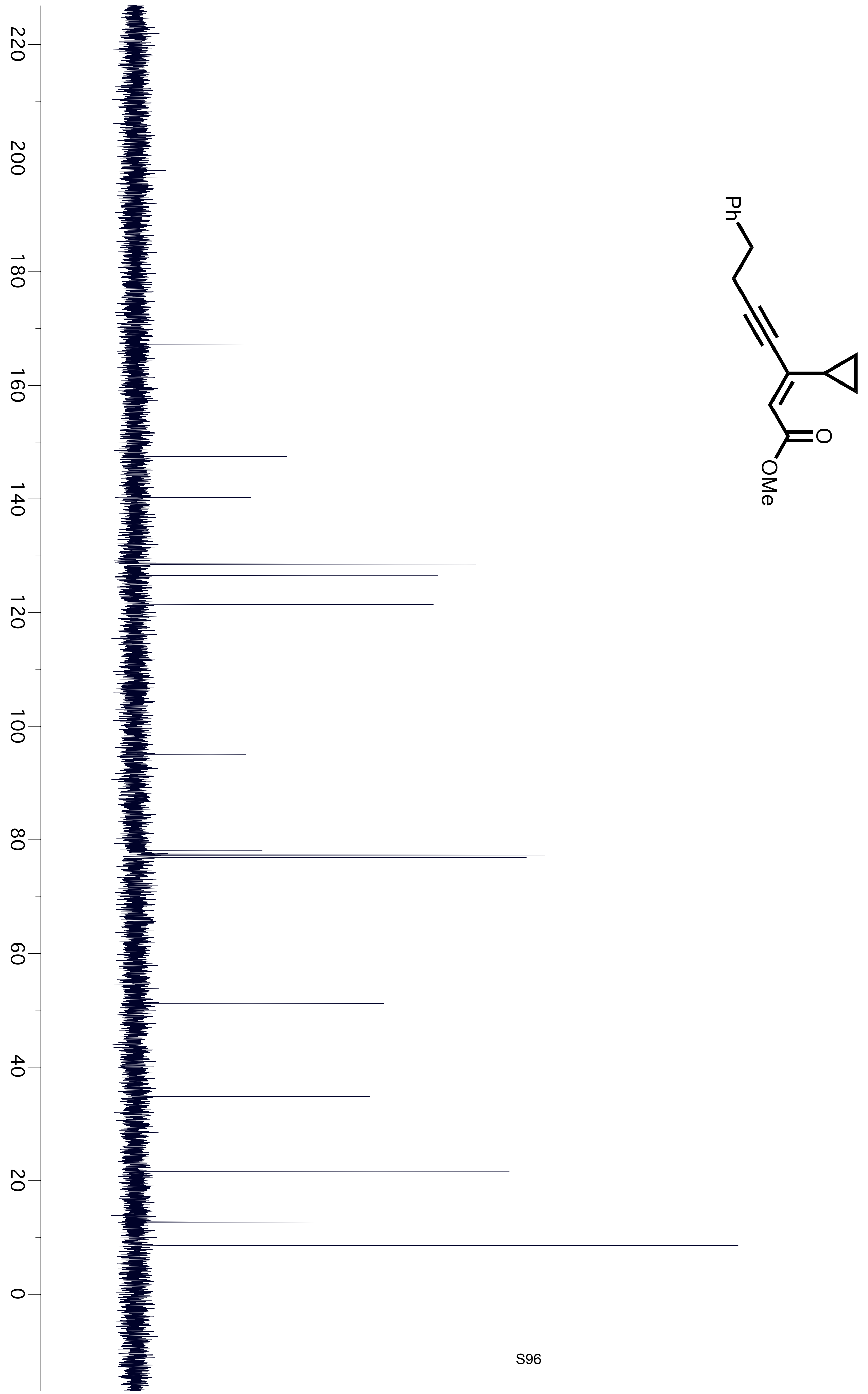

Ph
O
OMe
220
200
180
160
140
120
100
80
60
40
20
0

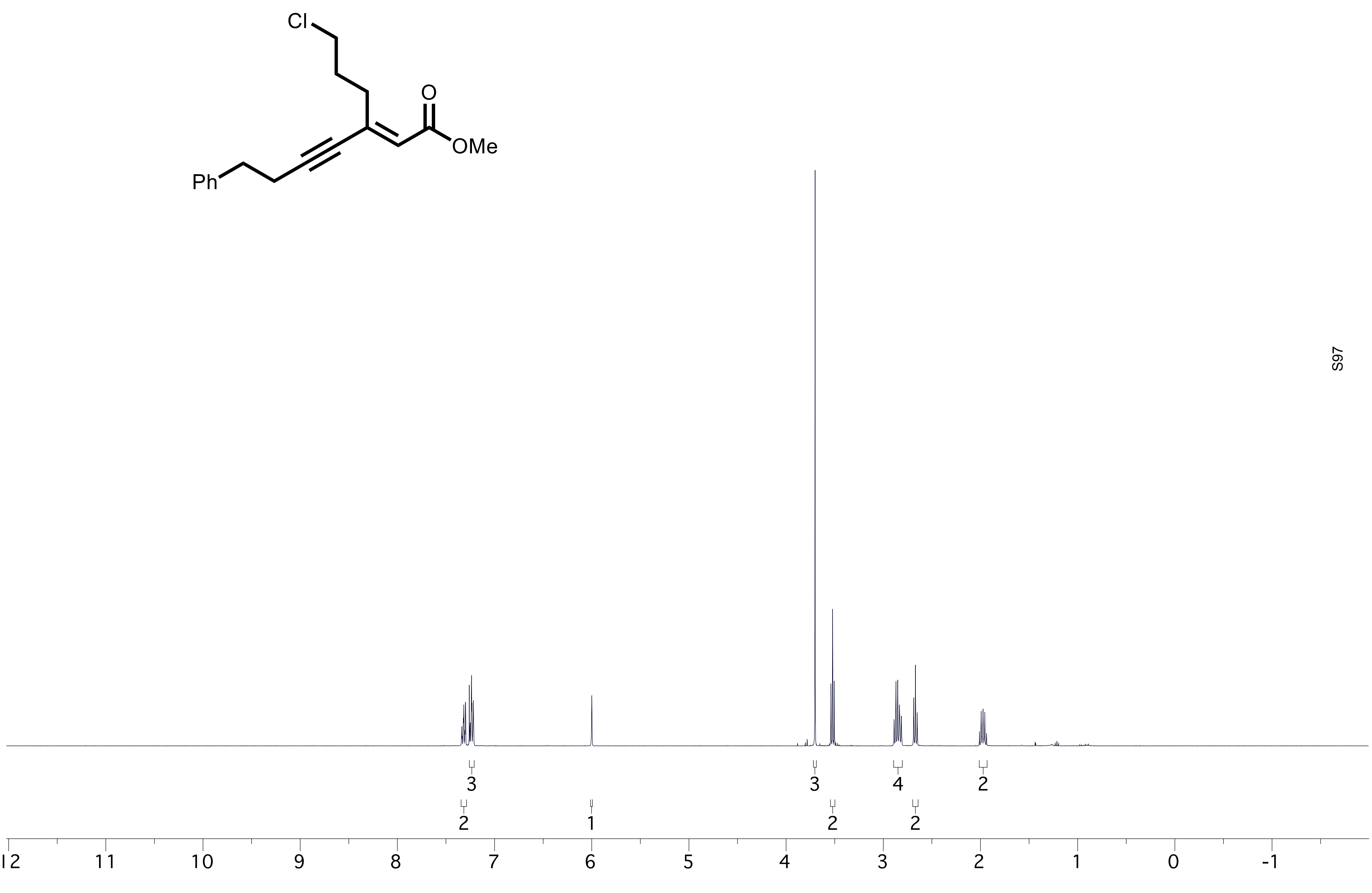
Cl
O
OMe
Ph
3
2
1
3
2
4
2
2
12
11
10
9
8
7
6
5
4
3
2
1
0
-1

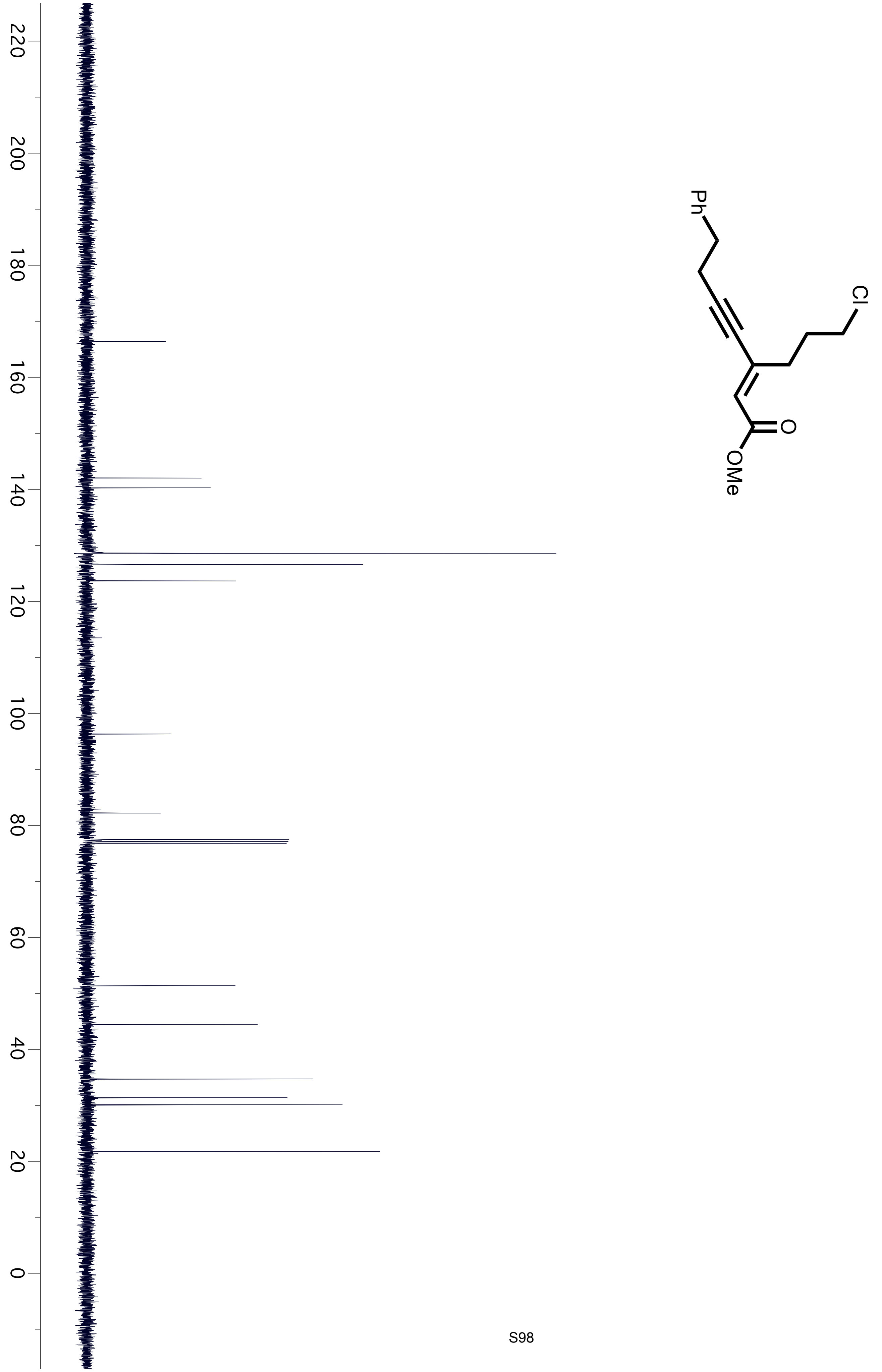
Ph
Cl
O
OMe
220
200
180
160
140
120
100
80
60
40
20
0

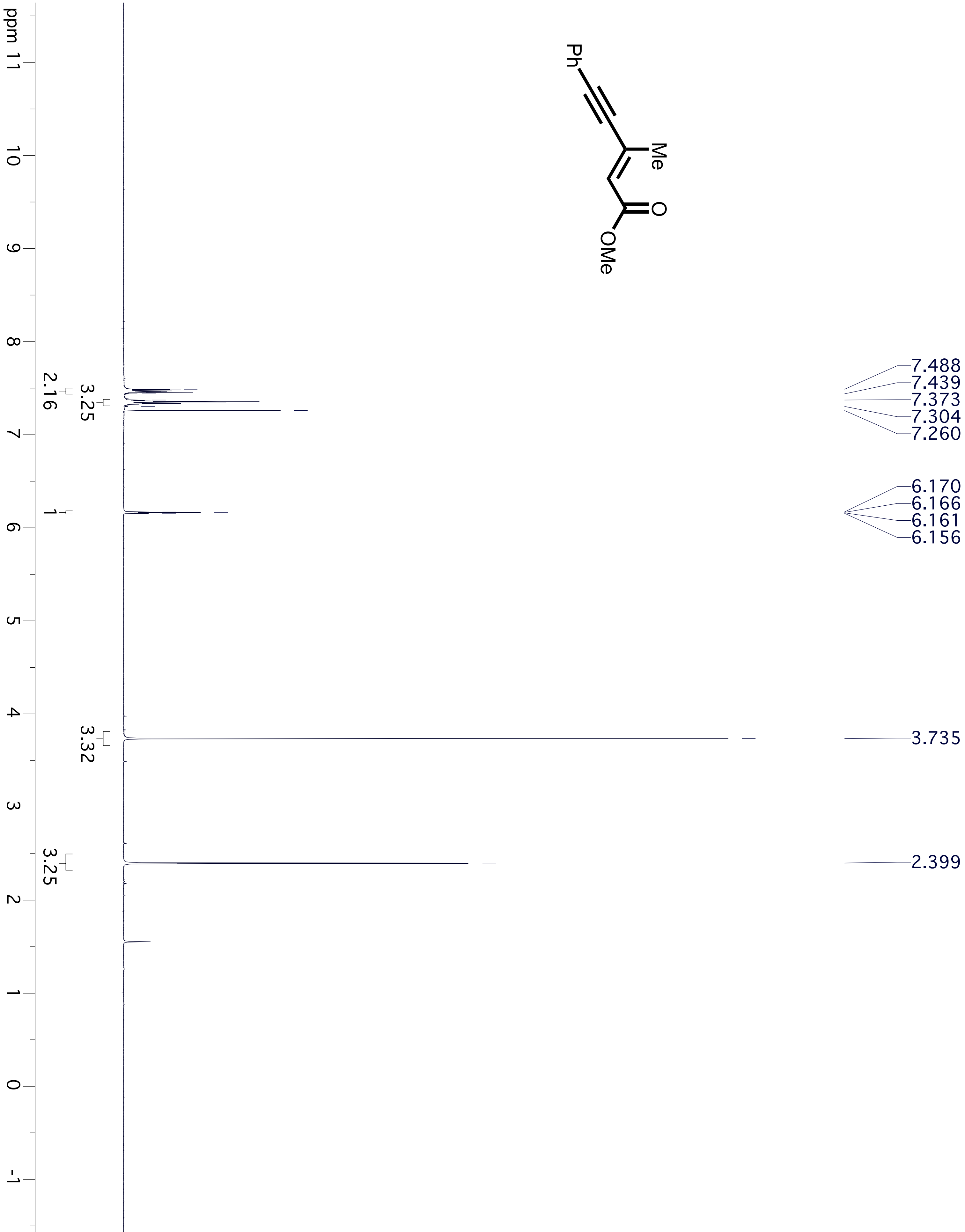
Ph
Me
O
OMe
7.488
7.439
7.373
7.304
7.260
6.170
6.166
6.161
6.156
3.735
2.399
2.16
3.25
1
3.32
3.25
ppm 11
10
9
8
7
6
5
4
3
2
1
0
-1

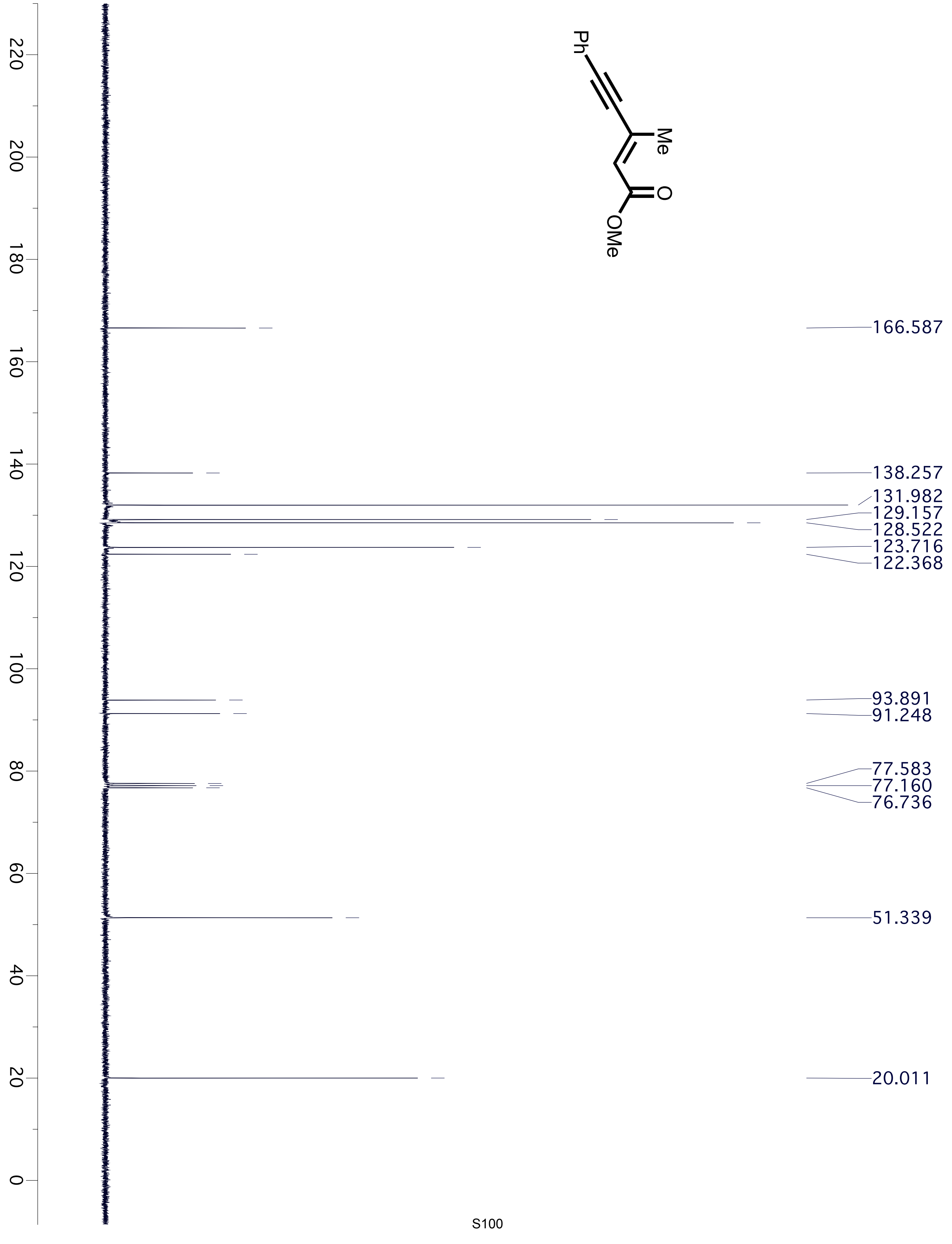
Ph
Me
O
OMe
166.587
138.257
131.982
129.157
128.522
123.716
122.368
93.891
91.248
77.583
77.160
76.736
51.339
20.011
220
200
180
160
140
120
100
80
60
40
20
0

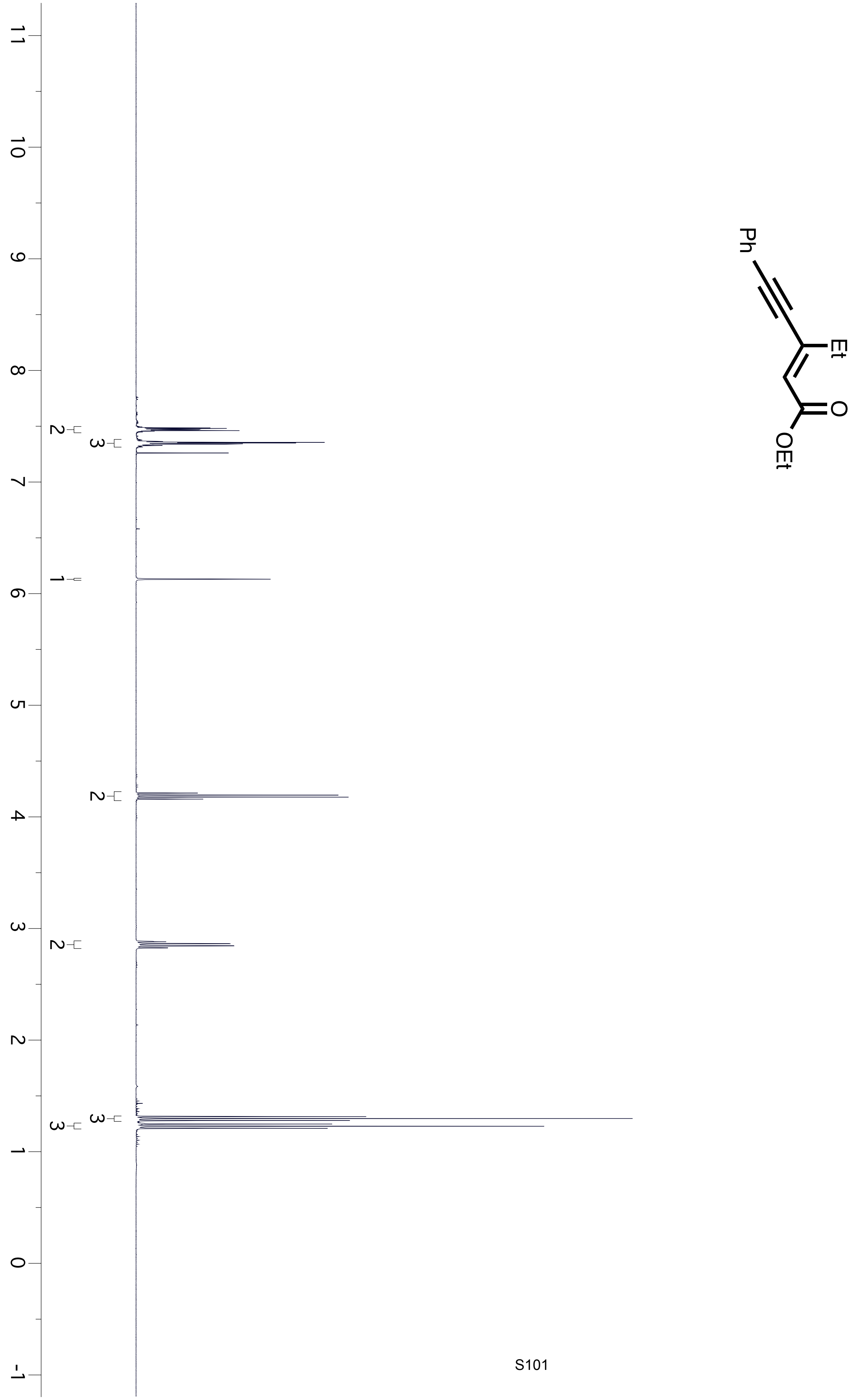
Ph
Et
O
OEt
11
10
9
8
7
6
5
4
3
2
1
0
-1
2
3
1
2
2
3
3

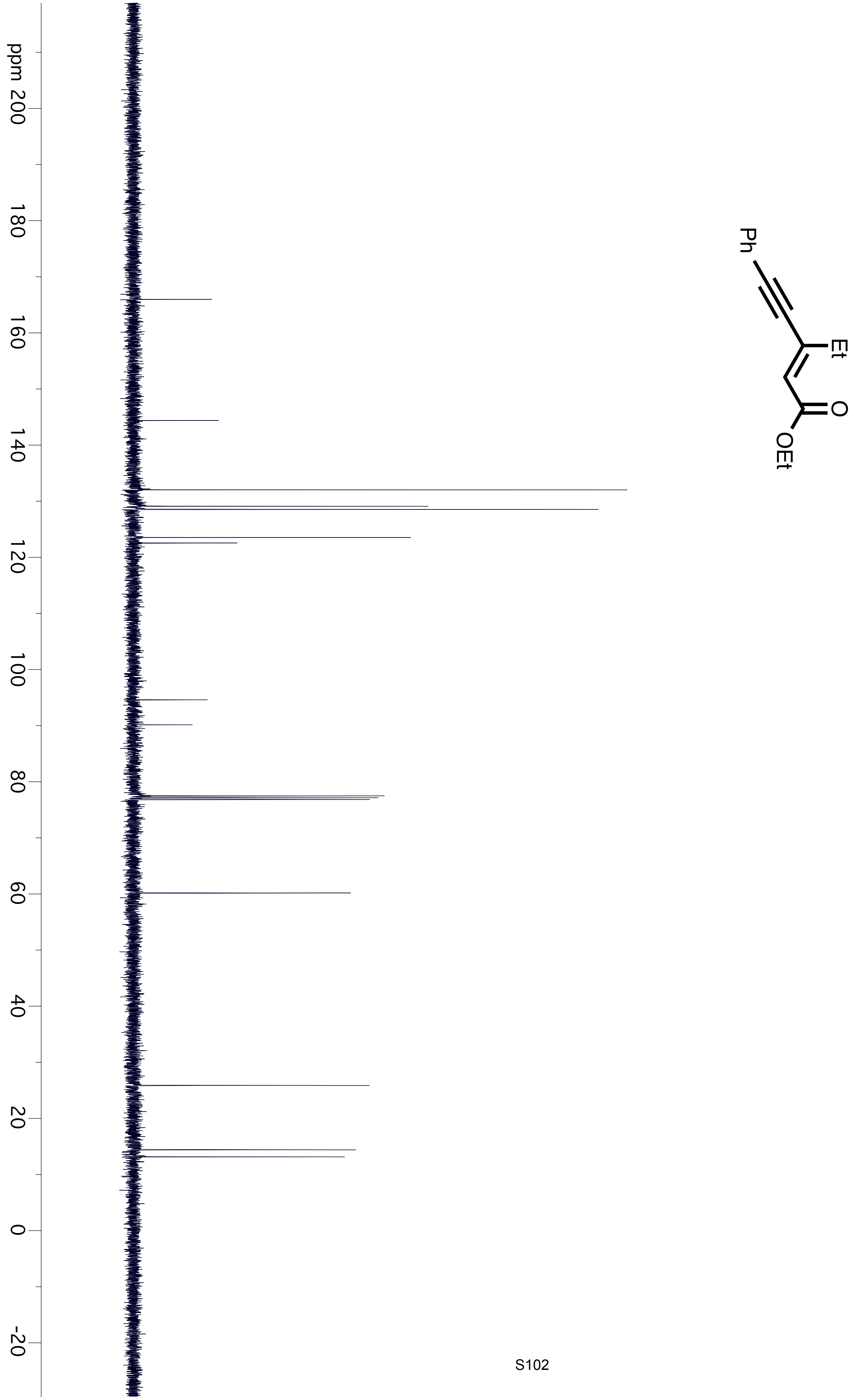
Ph
Et
O
OEt
ppm 200
180
160
140
120
100
80
60
40
20
0
-20

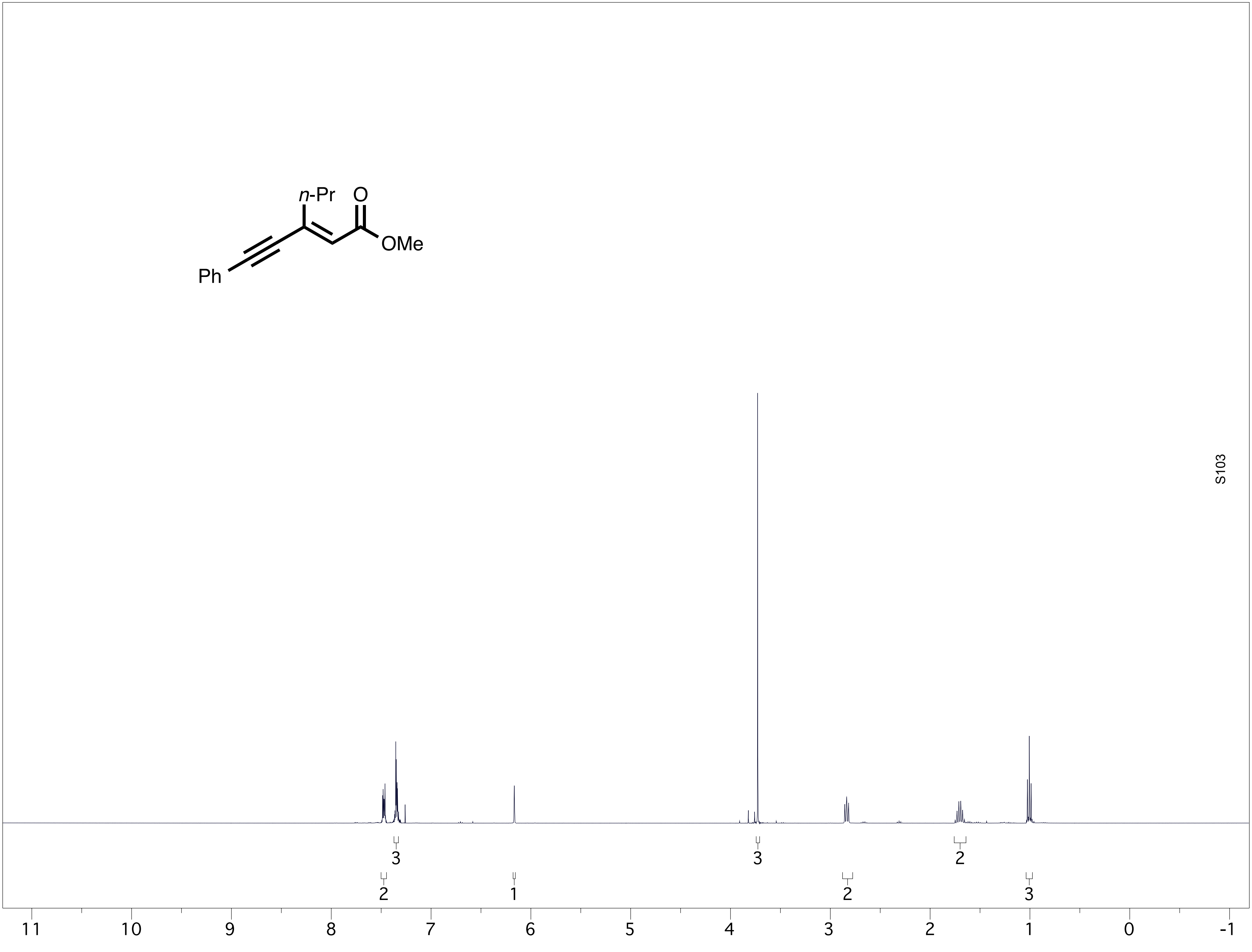

n-Pr
O
OMe
Ph
3
3
2
2
1
2
3
11
10
9
8
7
6
5
4
3
2
1
0
-1

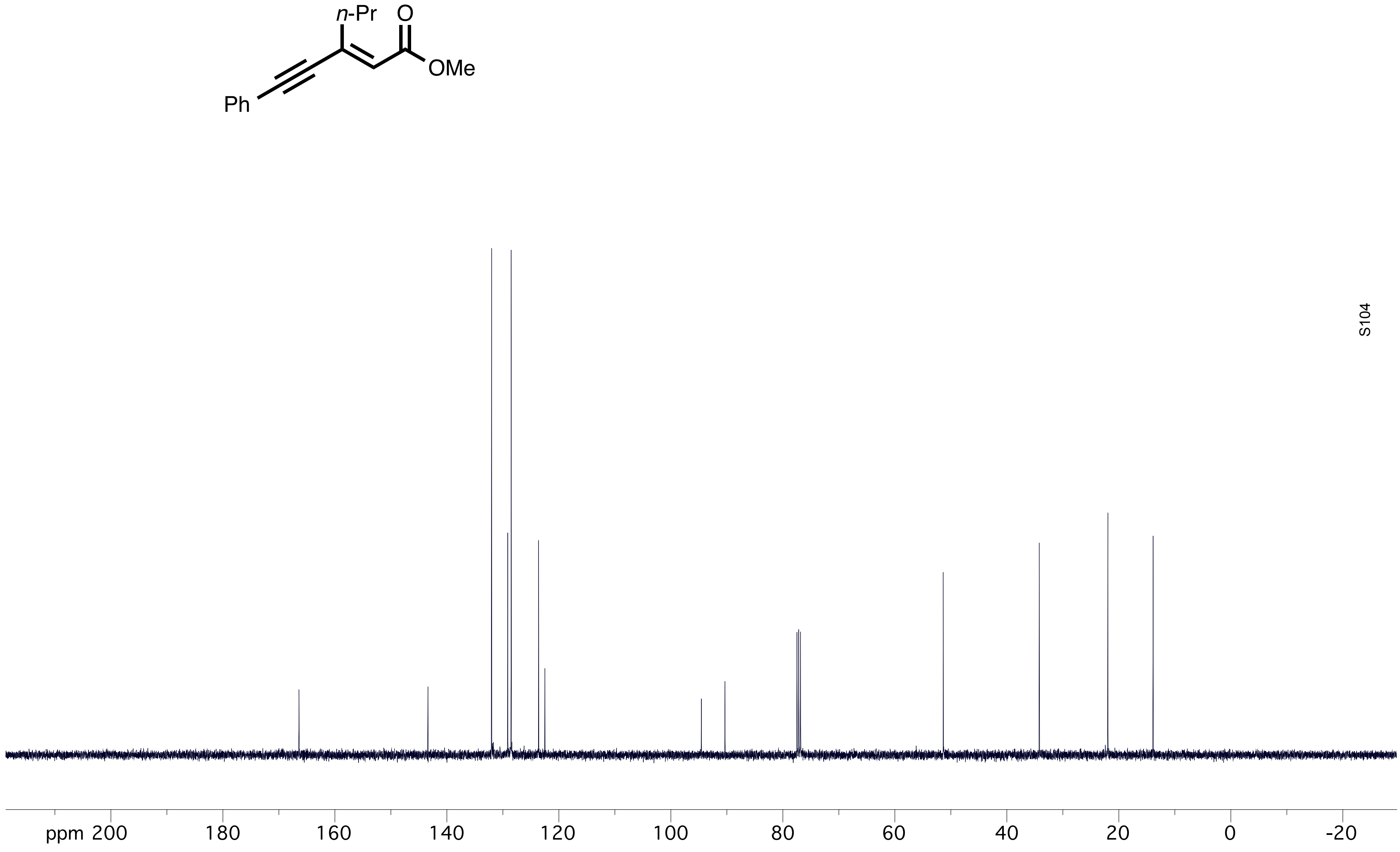
n-Pr
O
OMe
Ph
ppm 200
180
160
140
120
100
80
60
40
20
0
-20

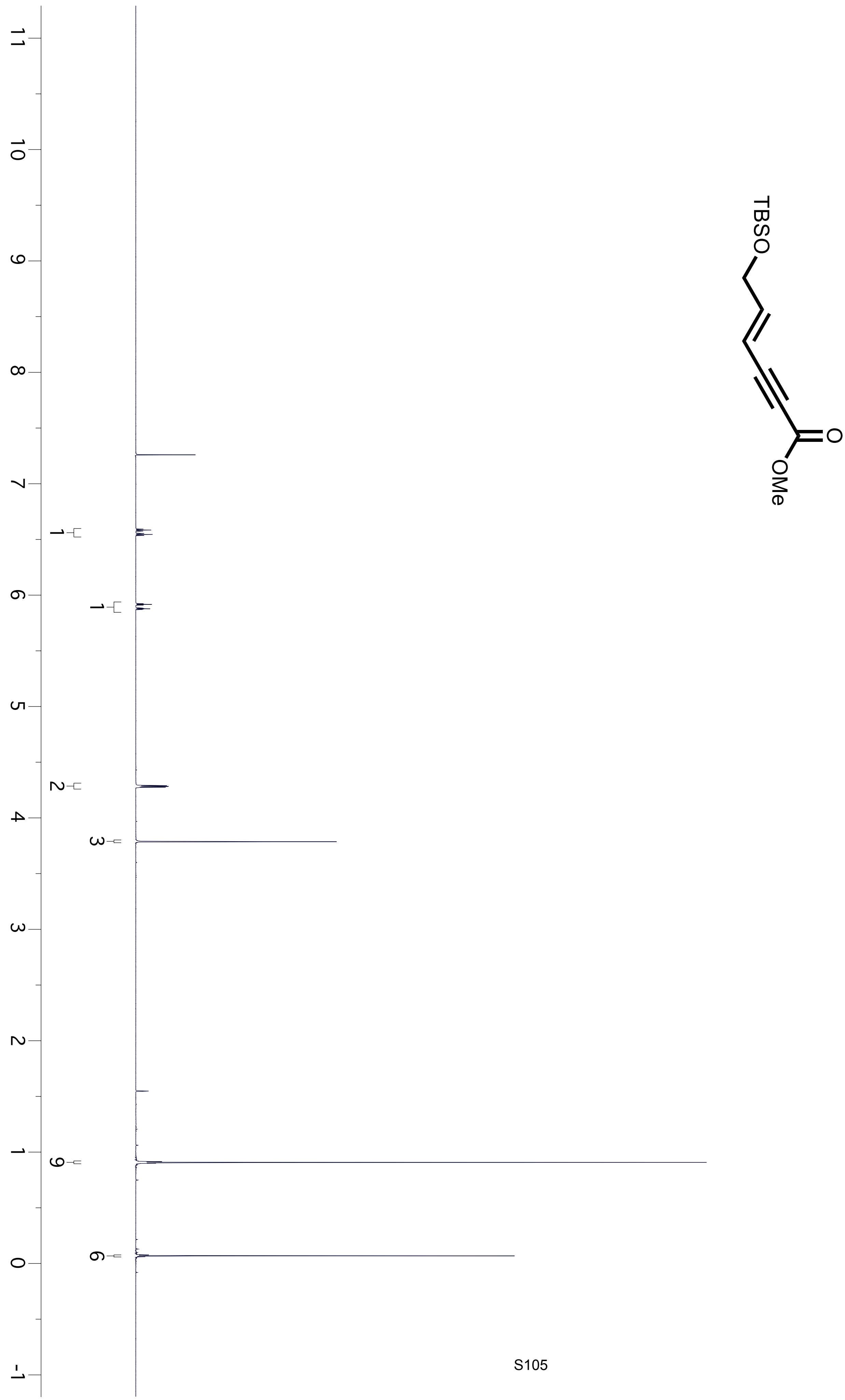

TBSO
O
OMe
11
10
9
8
7
6
5
4
3
2
1
0
-1
1
1
2
3
9
6

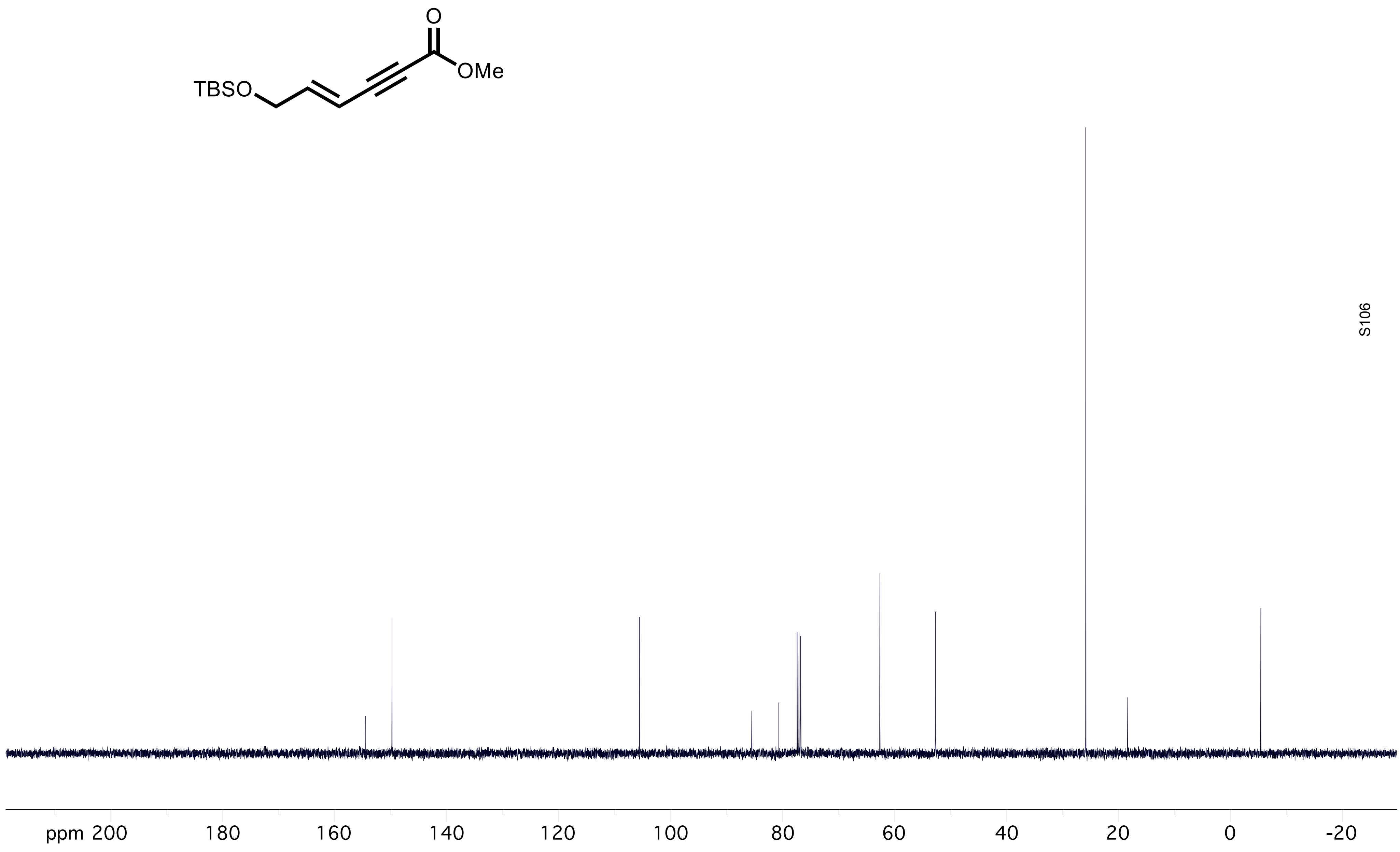

TBSO
O
OMe
ppm 200
180
160
140
120
100
80
60
40
20
0
-20

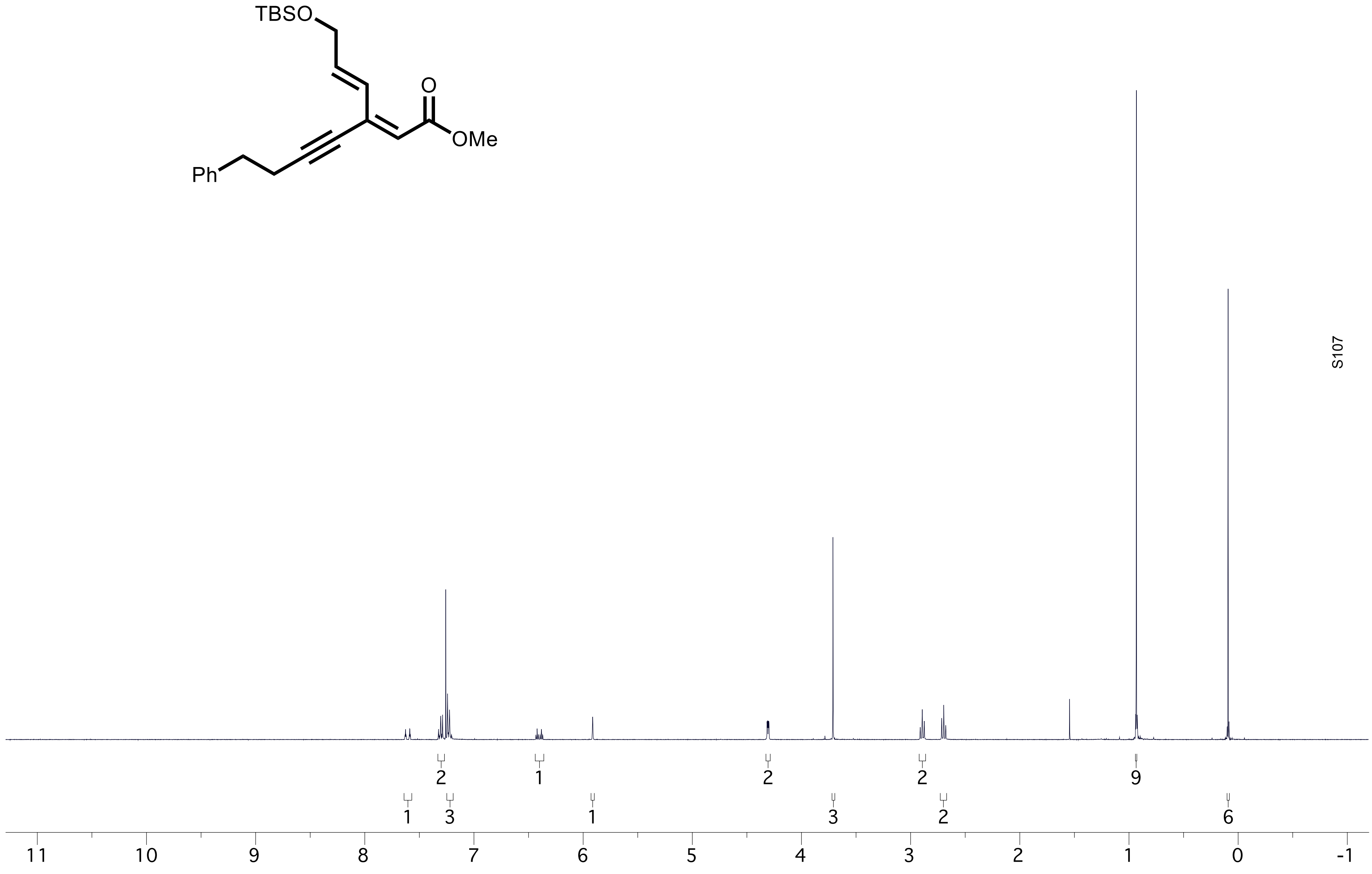
TBSO
O
OMe
Ph
2
1
2
2
9
1
3
1
3
2
6
11
10
9
8
7
6
5
4
3
2
1
0
-1

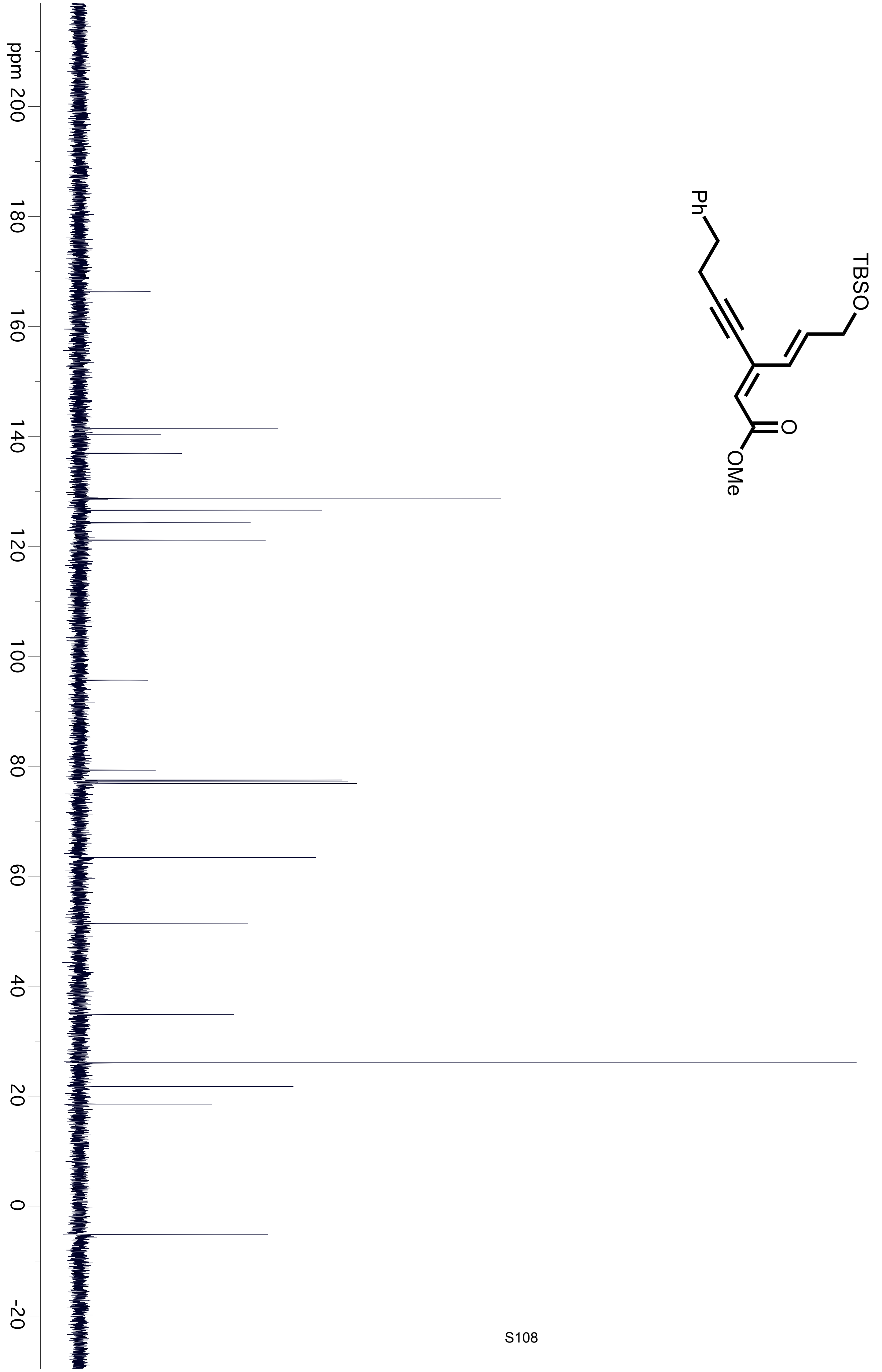
ppm 200
180
160
140
120
100
80
60
40
20
0
-20
TBSO
Ph
O
OMe

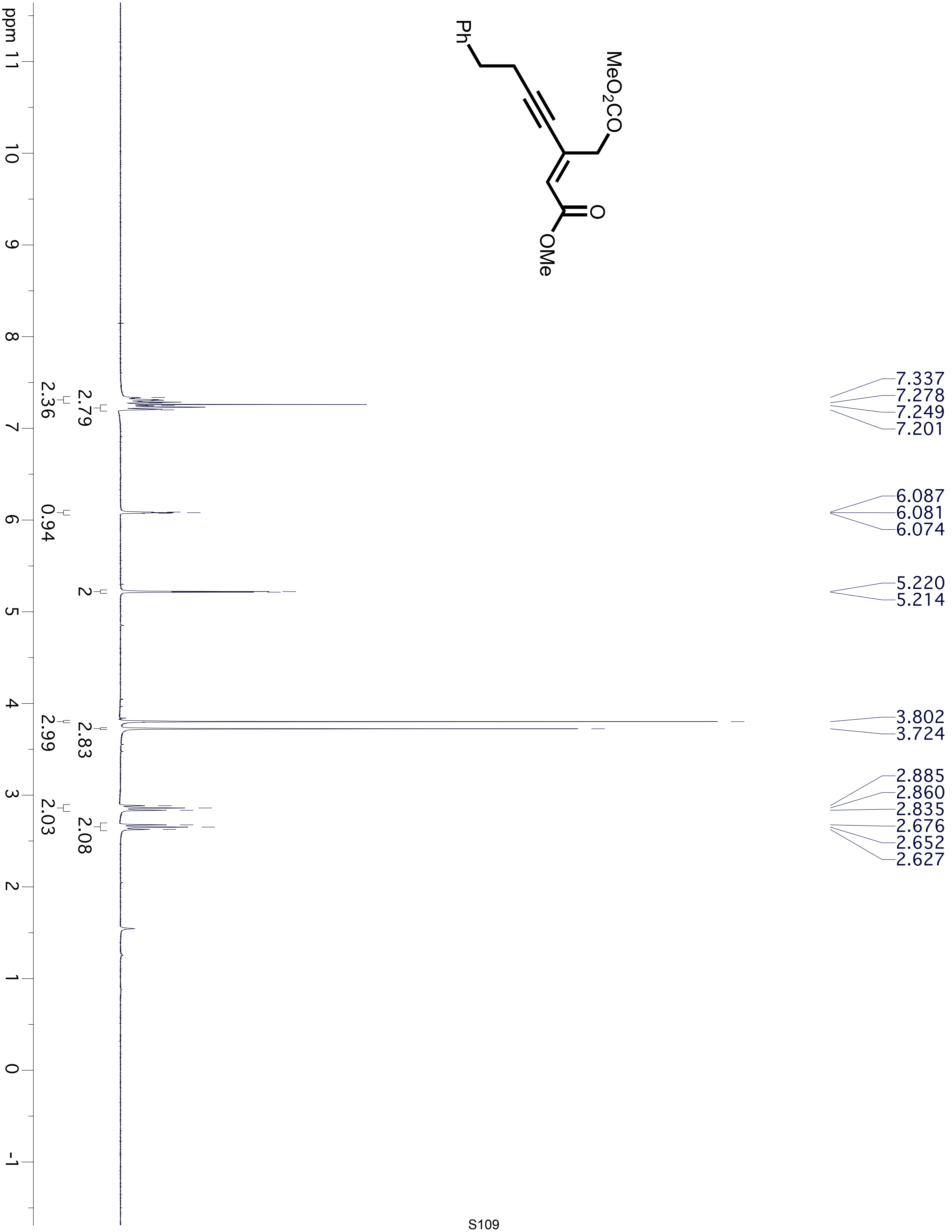

Ph
MeO2CO
O
OMe
7.337
7.278
7.249
7.201
6.087
6.081
6.074
5.220
5.214
3.802
3.724
2.885
2.860
2.835
2.676
2.652
2.627
2.36
2.79
0.94
2
2.99
2.83
2.03
2.08
ppm 11
10
9
8
7
6
5
4
3
2
1
0
-1

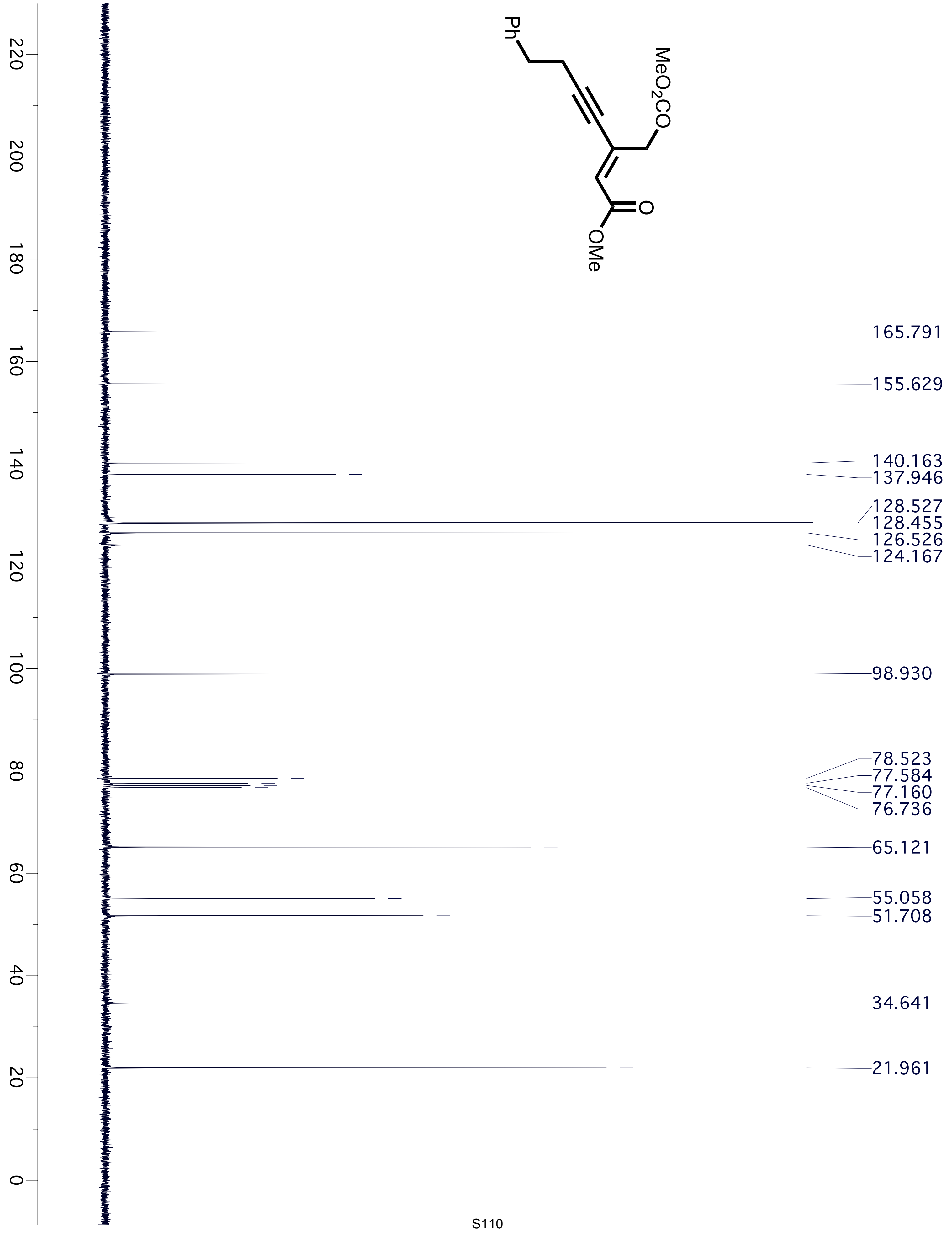
165.791
155.629
140.163
137.946
128.527
128.455
126.526
124.167
98.930
78.523
77.584
77.160
76.736
65.121
55.058
51.708
34.641
21.961
Ph
MeO_2CO
O
OMe

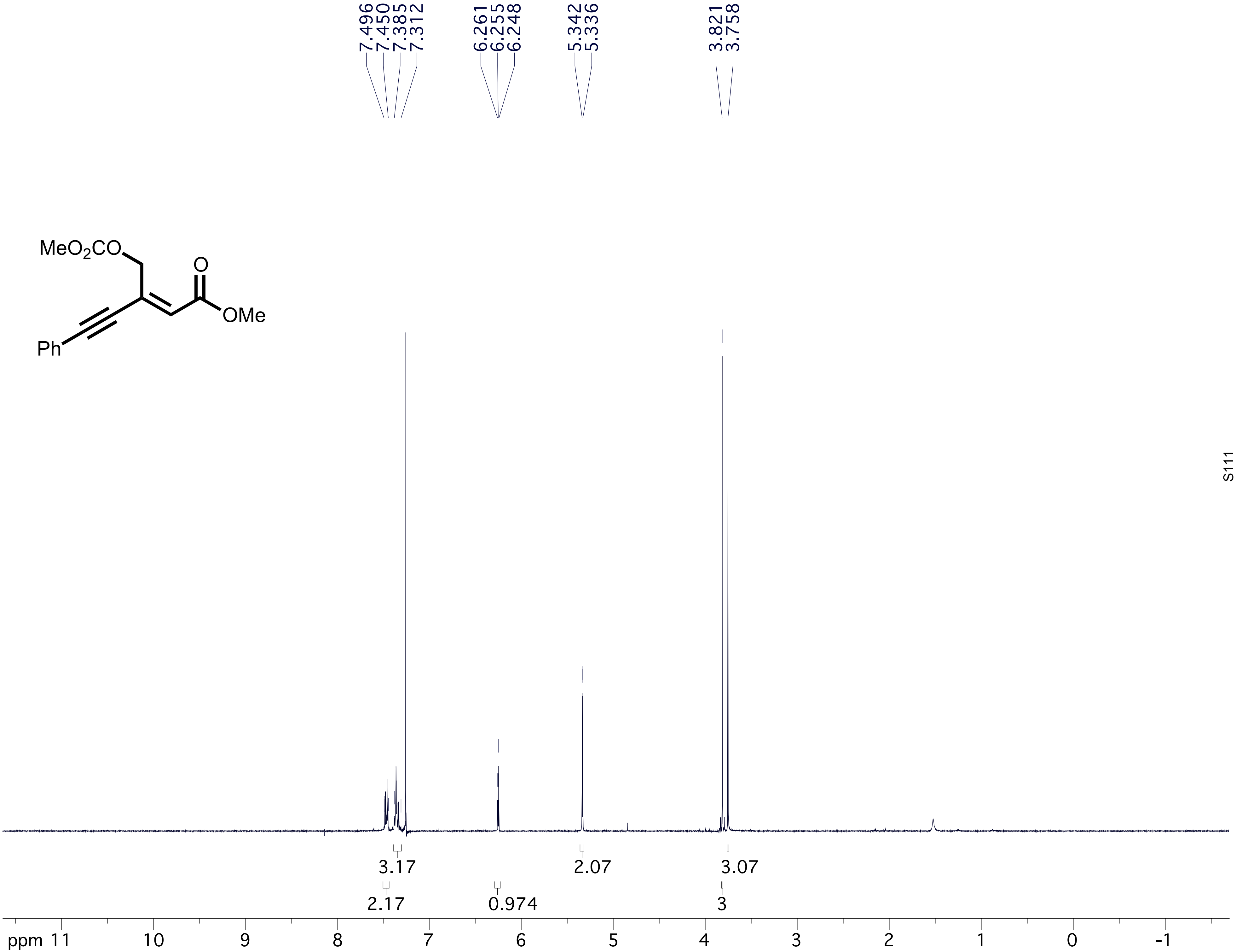
7.496
7.450
7.385
7.312
6.261
6.255
6.248
5.342
5.336
3.821
3.758
MeO2CO
O
OMe
Ph
3.17
2.17
0.974
2.07
3.07
3
ppm 11
10
9
8
7
6
5
4
3
2
1
0
-1

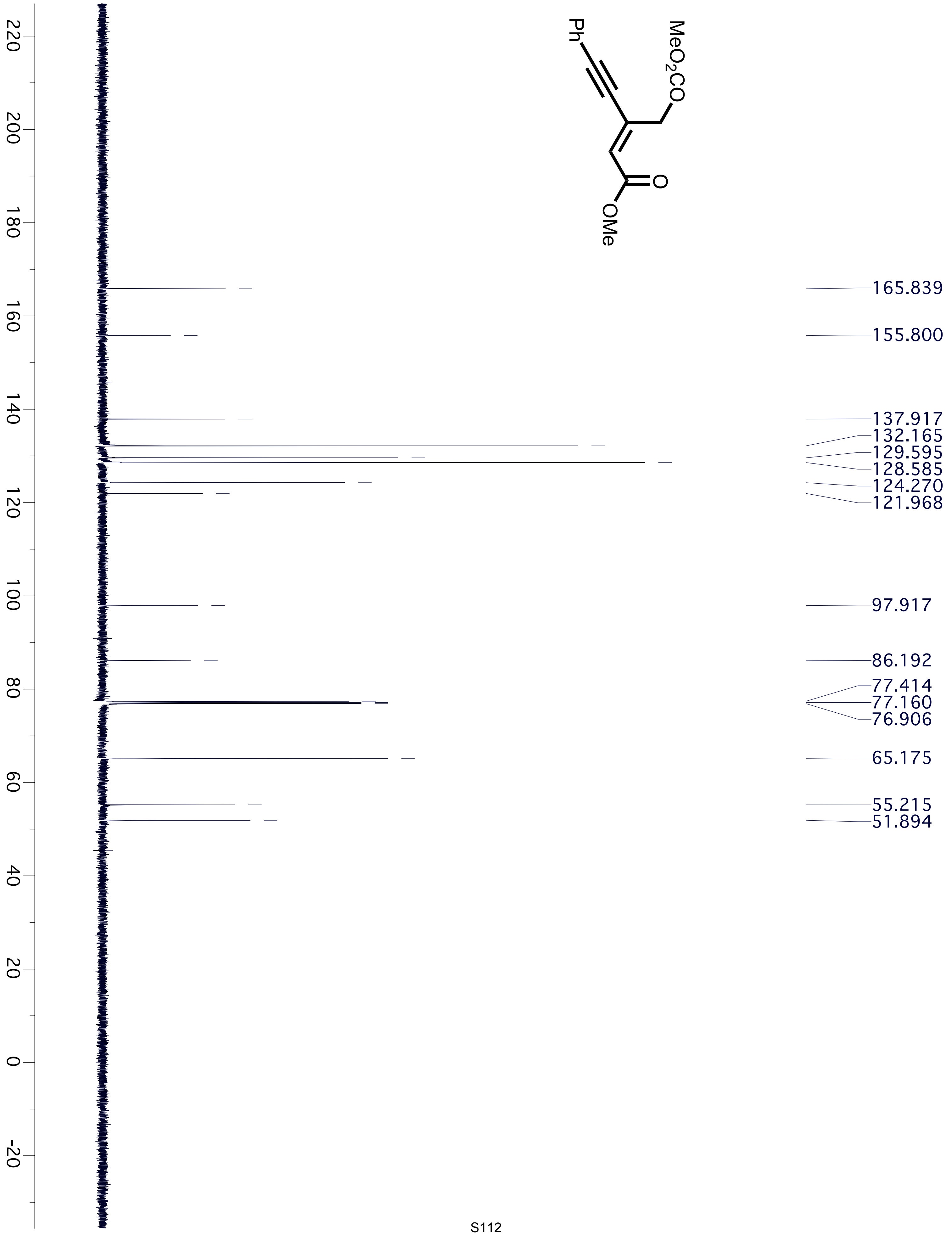
MeO2CO
Ph
O
OMe
165.839
155.800
137.917
132.165
129.595
128.585
124.270
121.968
97.917
86.192
77.414
77.160
76.906
65.175
55.215
51.894

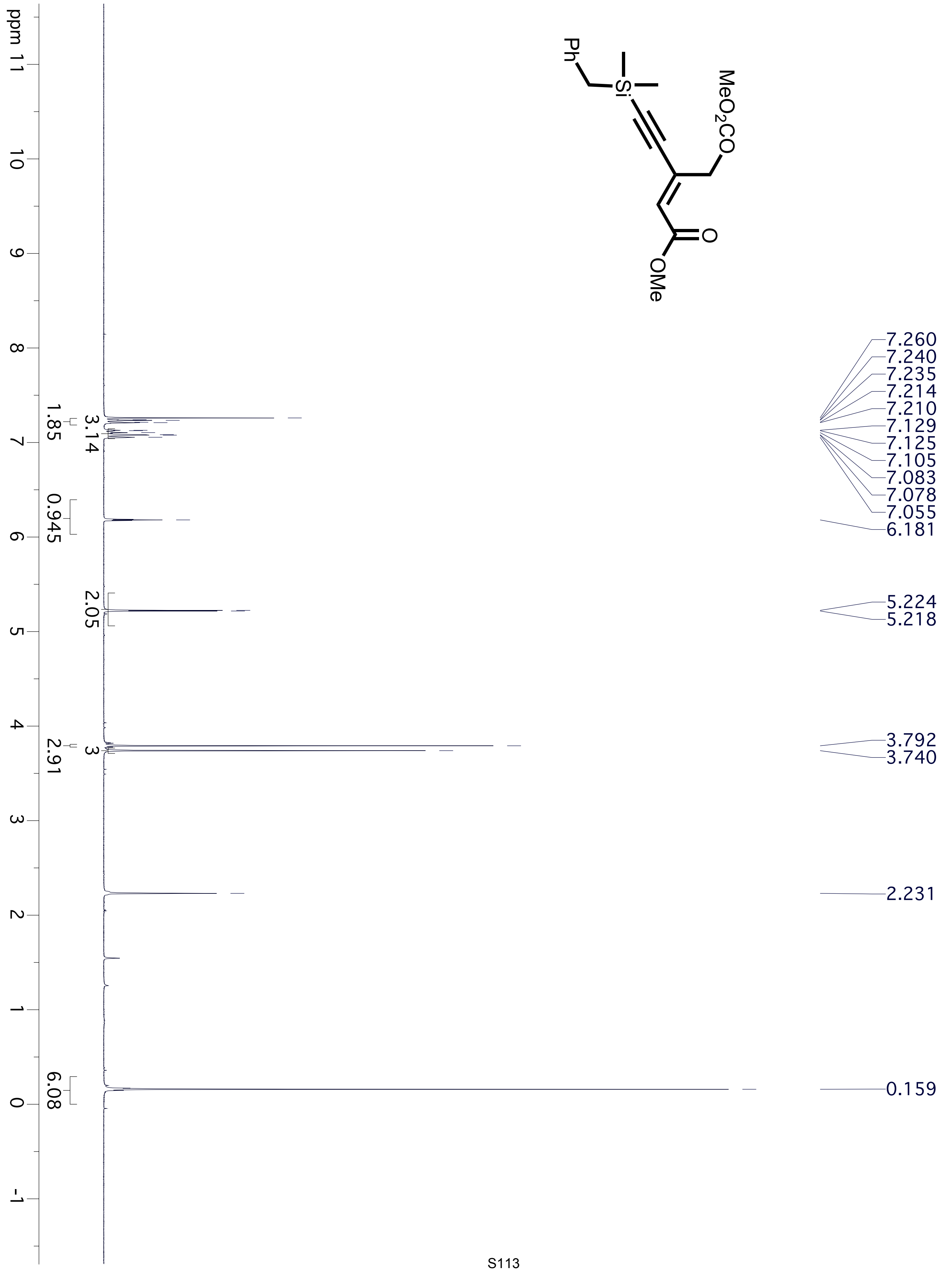

MeO2CO
Si
Ph
O
OMe
7.260
7.240
7.235
7.214
7.210
7.129
7.125
7.105
7.083
7.078
7.055
6.181
5.224
5.218
3.792
3.740
2.231
0.159
1.85
3.14
0.945
2.05
2.91
3
6.08
ppm

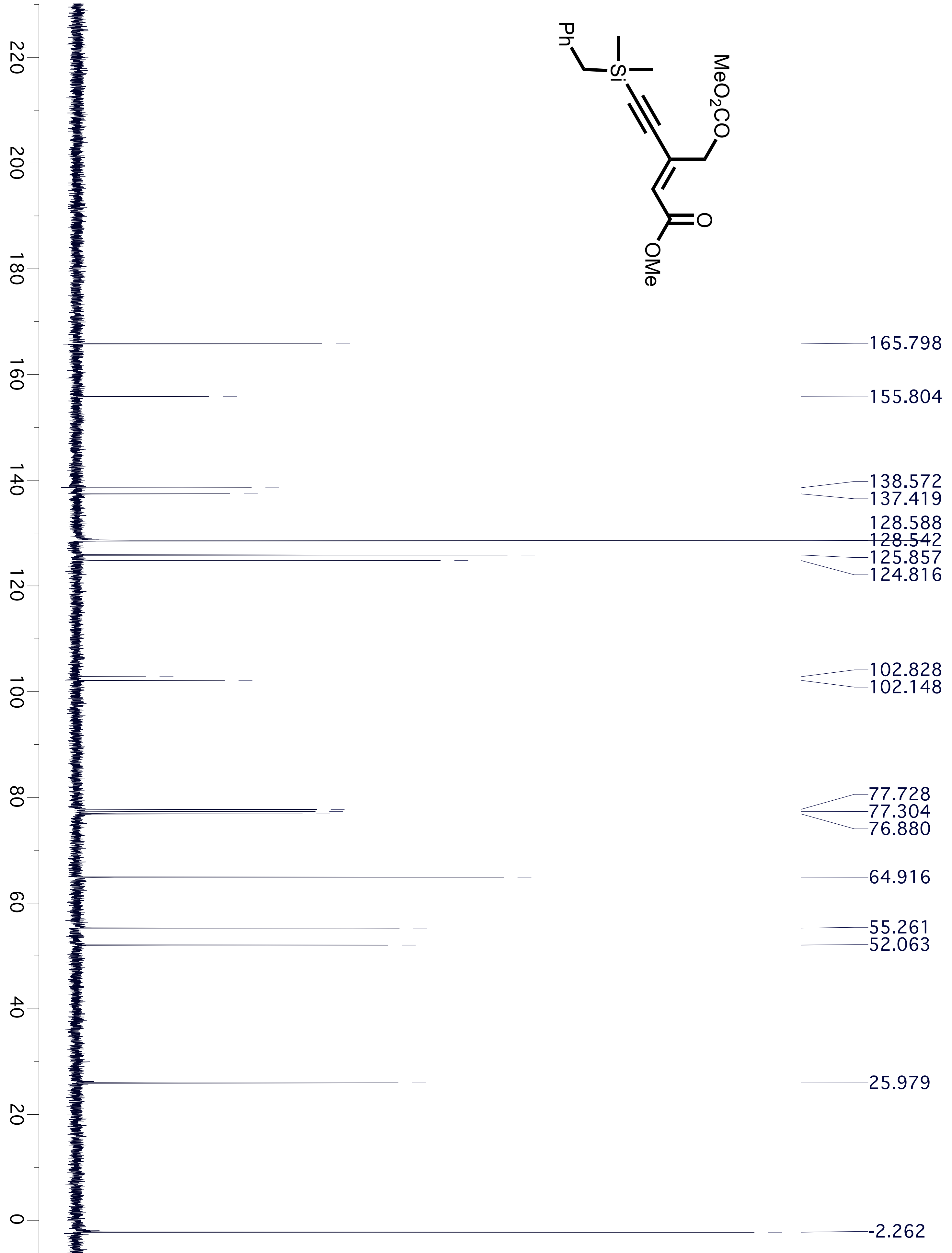
Ph
Si
MeO2CO
O
OMe
165.798
155.804
138.572
137.419
128.588
128.542
125.857
124.816
102.828
102.148
77.728
77.304
76.880
64.916
55.261
52.063
25.979
-2.262
220
200
180
160
140
120
100
80
60
40
20
0

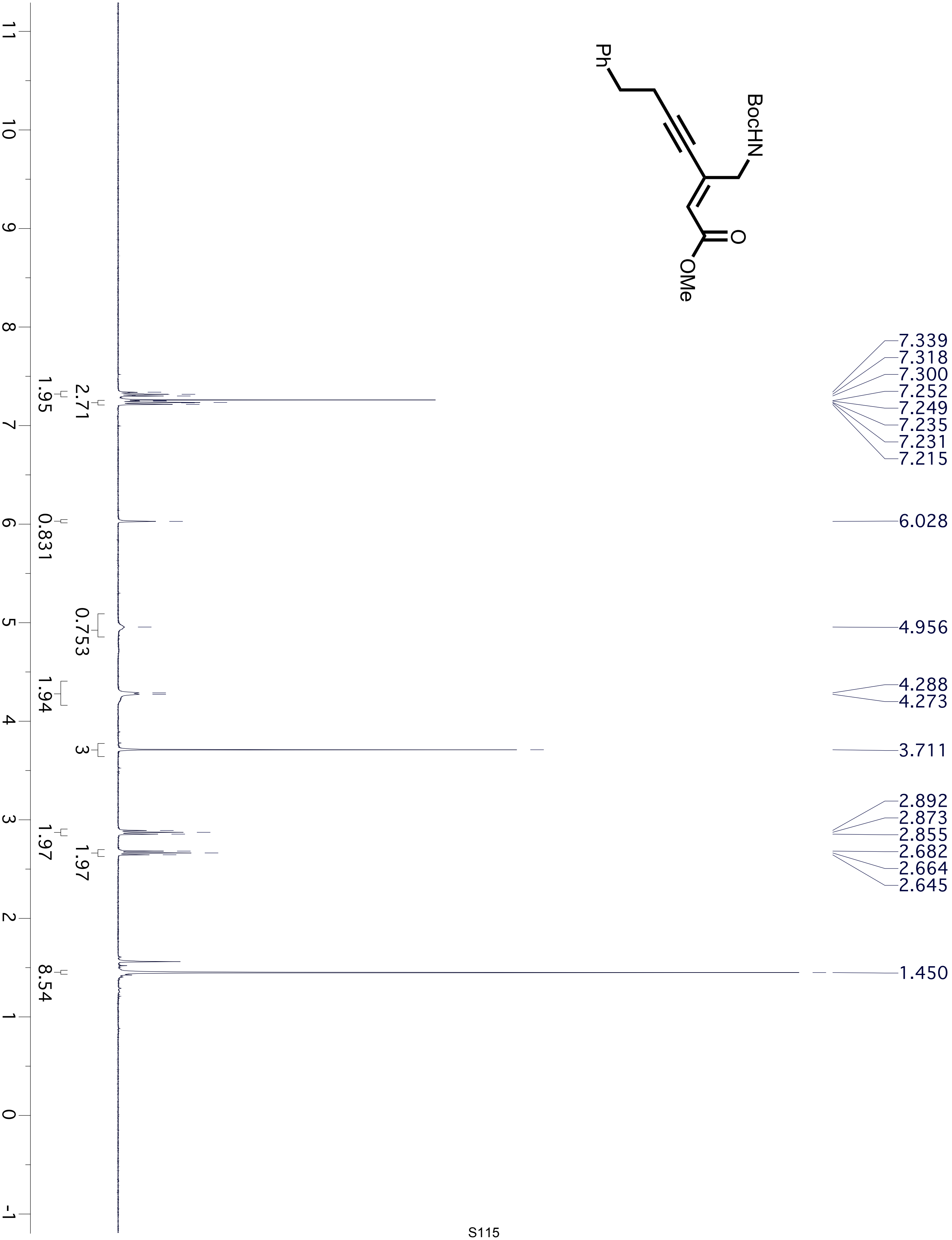

BocHN
Ph
O
OMe
7.339
7.318
7.300
7.252
7.249
7.235
7.231
7.215
6.028
4.956
4.288
4.273
3.711
2.892
2.873
2.855
2.682
2.664
2.645
1.450
1.95
2.71
0.831
0.753
1.94
3
1.97
1.97
8.54

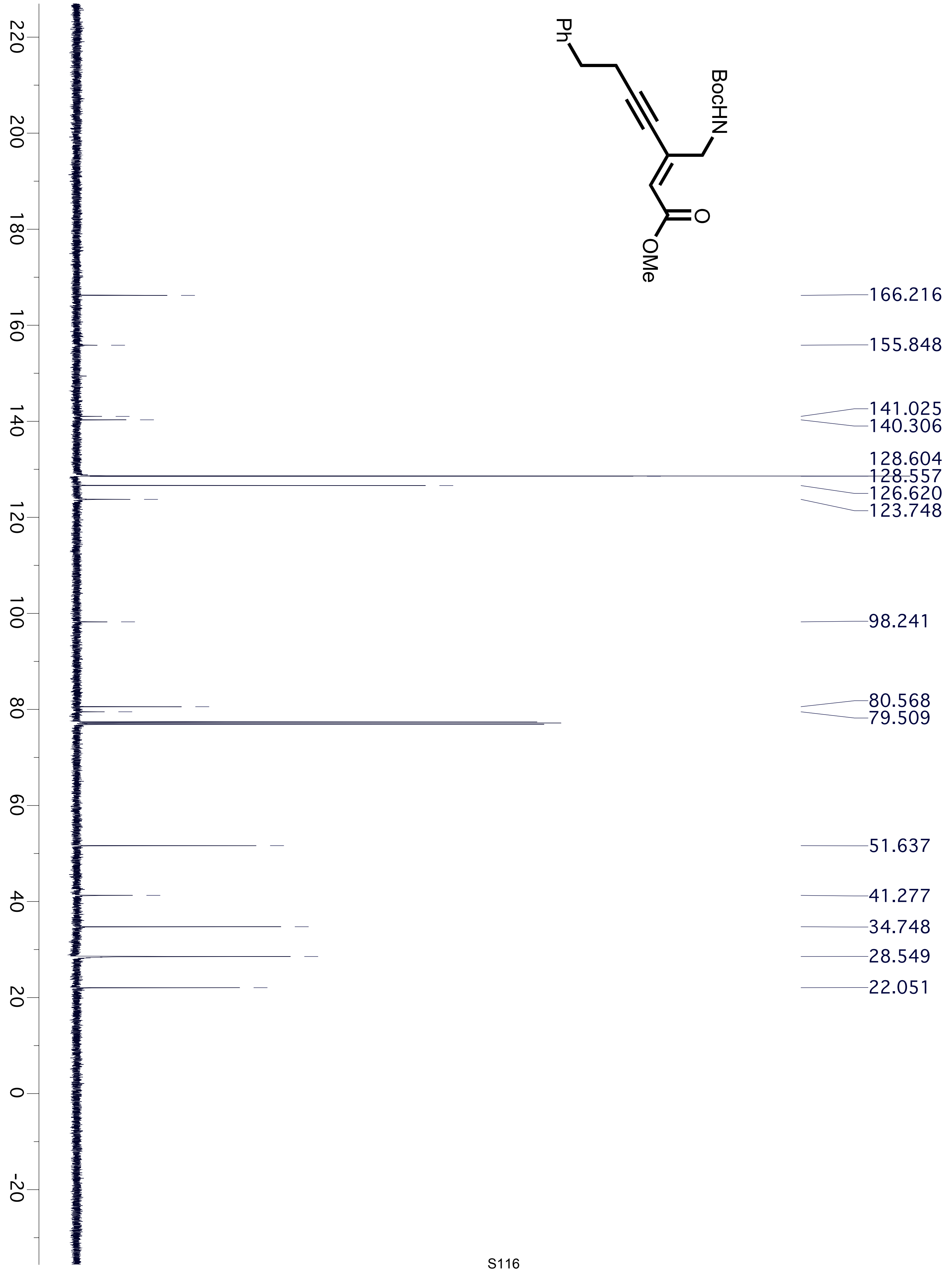

Ph
BocHN
O
OMe
166.216
155.848
141.025
140.306
128.604
128.557
126.620
123.748
98.241
80.568
79.509
51.637
41.277
34.748
28.549
22.051
220
200
180
160
140
120
100
80
60
40
20
0
-20

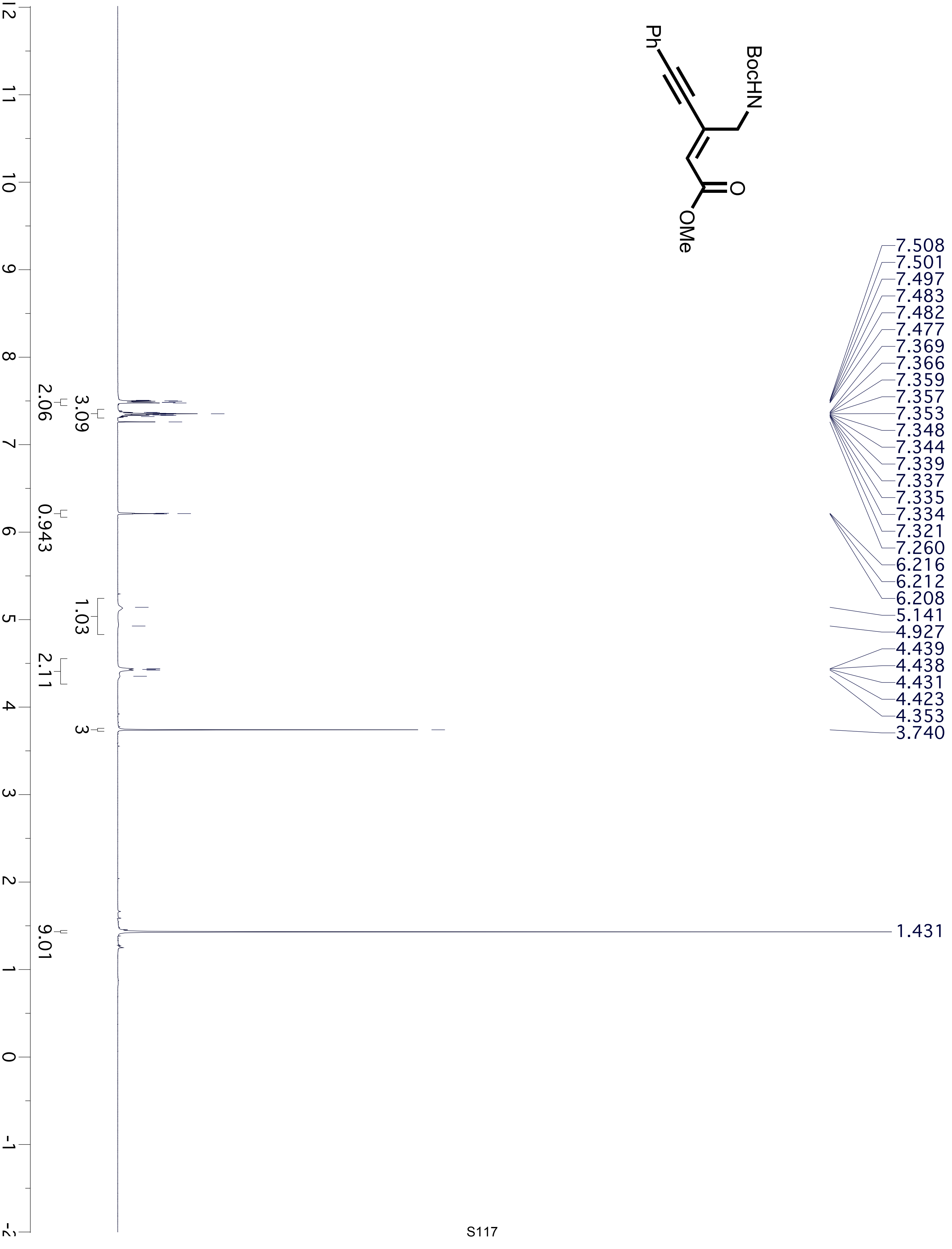
BocHN
Ph
O
OMe
7.508
7.501
7.497
7.483
7.482
7.477
7.369
7.366
7.359
7.357
7.353
7.348
7.344
7.339
7.337
7.335
7.334
7.321
7.260
6.216
6.212
6.208
5.141
4.927
4.439
4.438
4.431
4.423
4.353
3.740
1.431
2.06
3.09
0.943
1.03
2.11
3
9.01
12
11
10
9
8
7
6
5
4
3
2
1
0
-1
-2

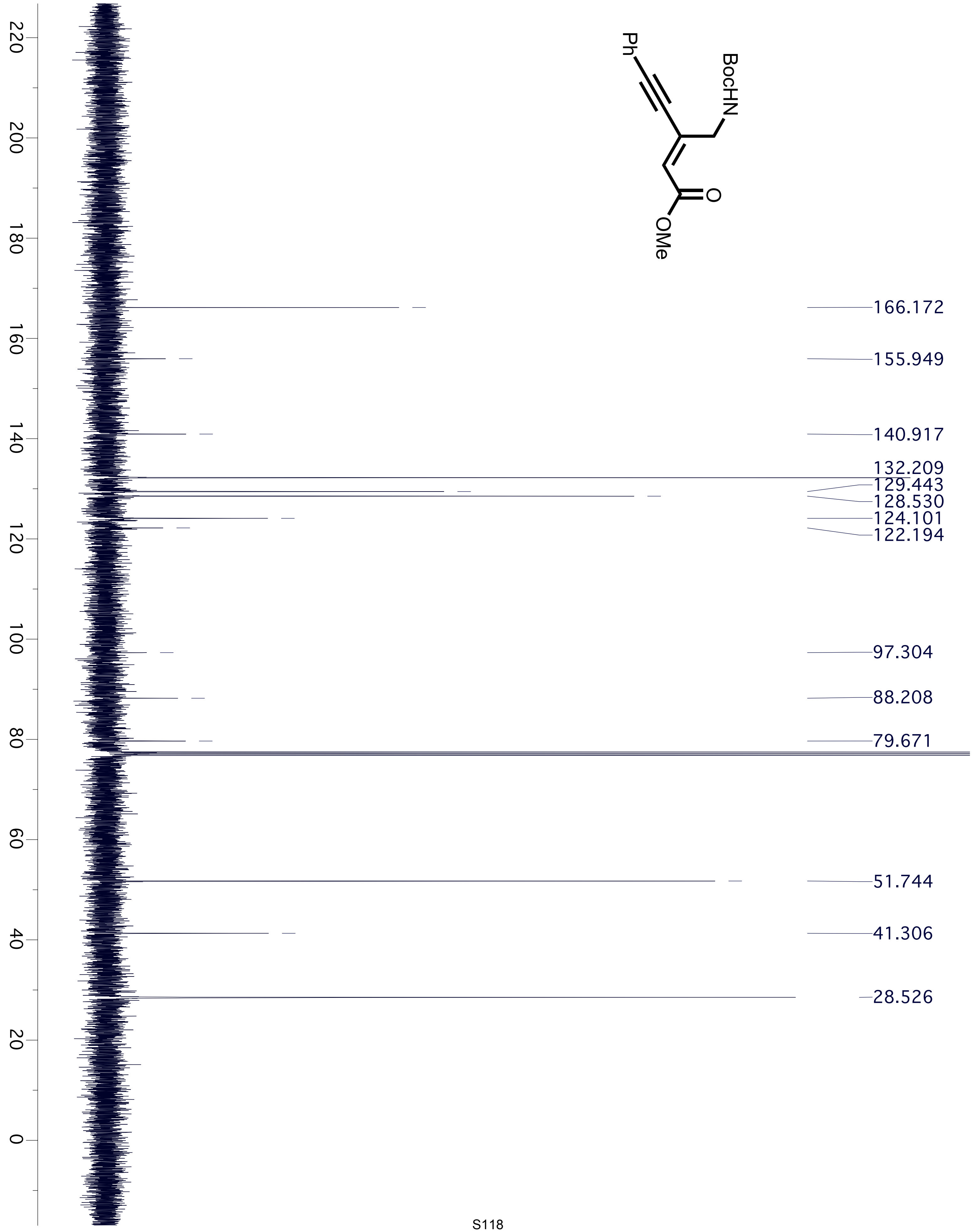
Ph
BocHN
O
OMe
166.172
155.949
140.917
132.209
129.443
128.530
124.101
122.194
97.304
88.208
79.671
51.744
41.306
28.526
220
200
180
160
140
120
100
80
60
40
20
0

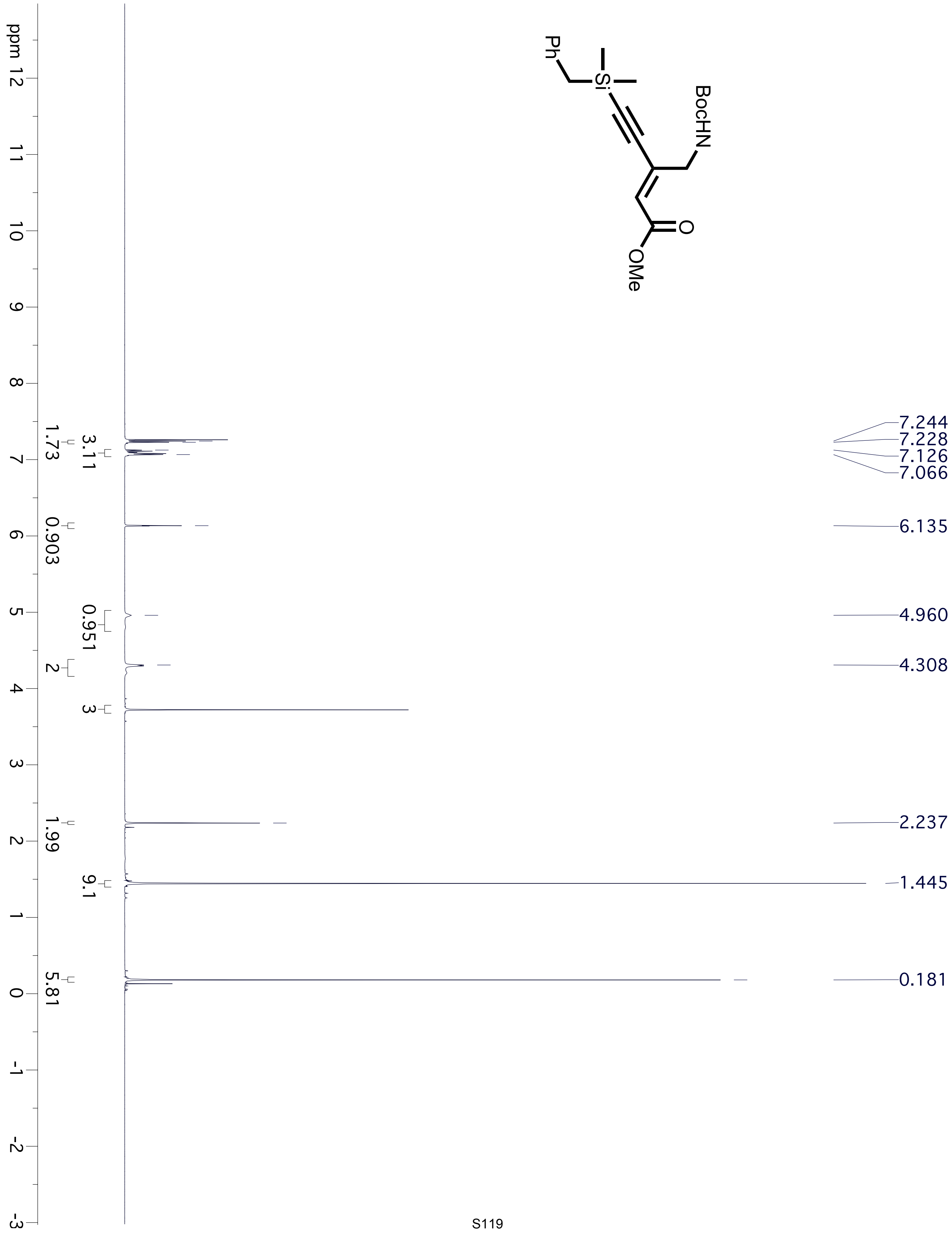
BocHN
Si
Ph
O
OMe
ppm
7.244
7.228
7.126
7.066
6.135
4.960
4.308
2.237
1.445
0.181
1.73
3.11
0.903
0.951
2
3
1.99
9.1
5.81

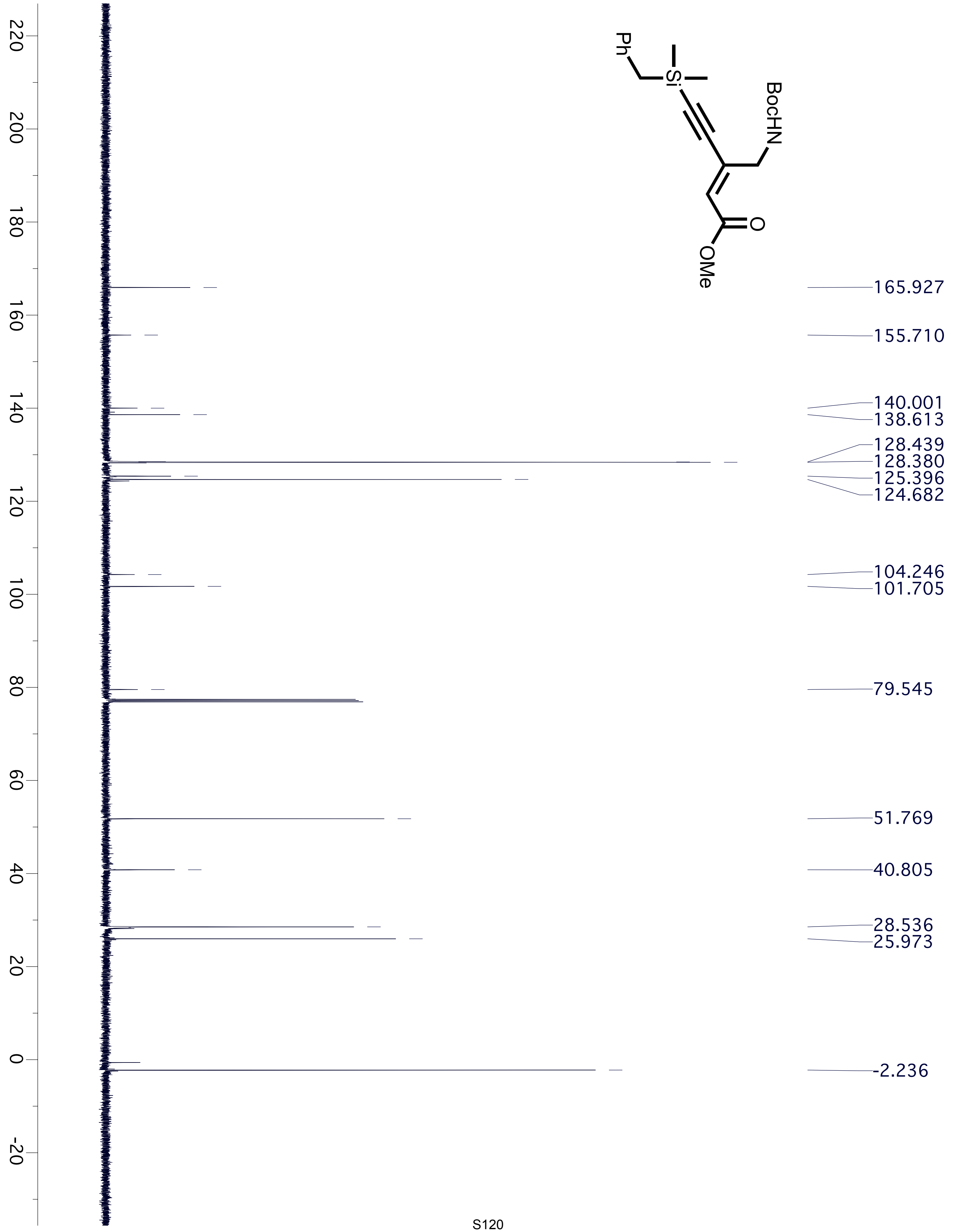

165.927
155.710
140.001
138.613
128.439
128.380
125.396
124.682
104.246
101.705
79.545
51.769
40.805
28.536
25.973
-2.236
Ph
Si
BocHN
O
OMe

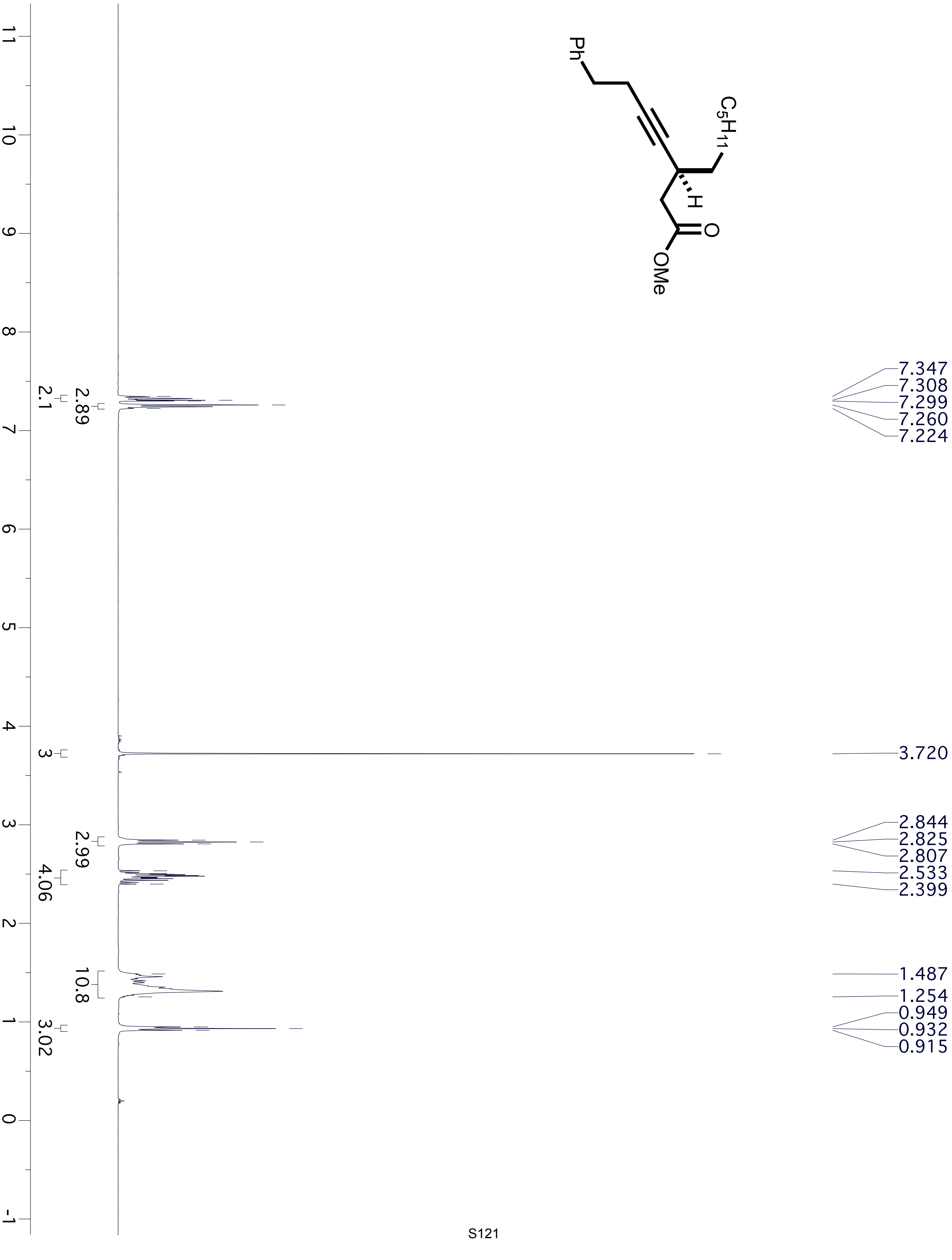
Ph
C5H11
H
O
OMe
7.347
7.308
7.299
7.260
7.224
3.720
2.844
2.825
2.807
2.533
2.399
1.487
1.254
0.949
0.932
0.915
2.1
2.89
3
2.99
4.06
10.8
3.02

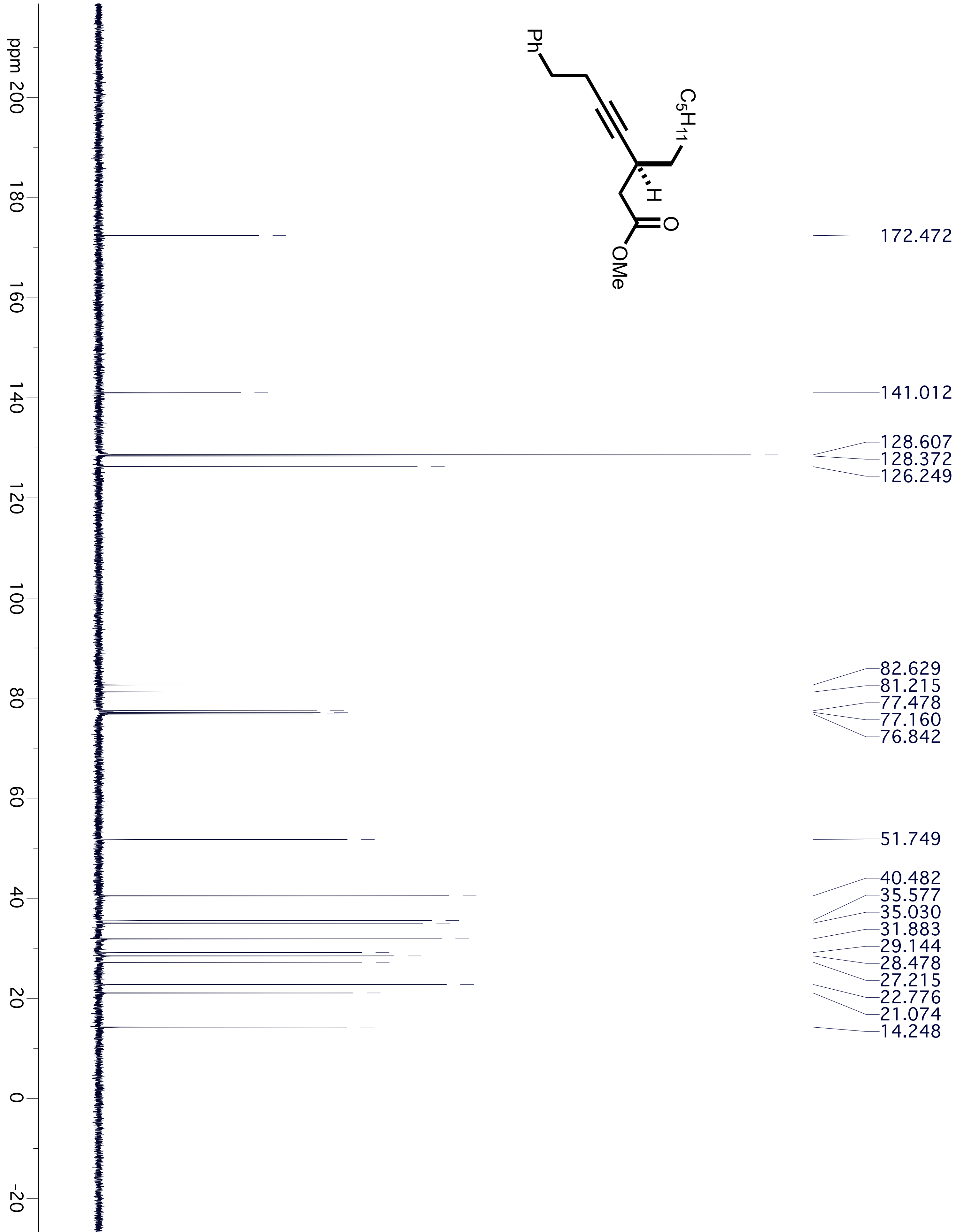
Ph
C5H11
H
O
OMe
172.472
141.012
128.607
128.372
126.249
82.629
81.215
77.478
77.160
76.842
51.749
40.482
35.577
35.030
31.883
29.144
28.478
27.215
22.776
21.074
14.248
ppm 200
180
160
140
120
100
80
60
40
20
0
-20

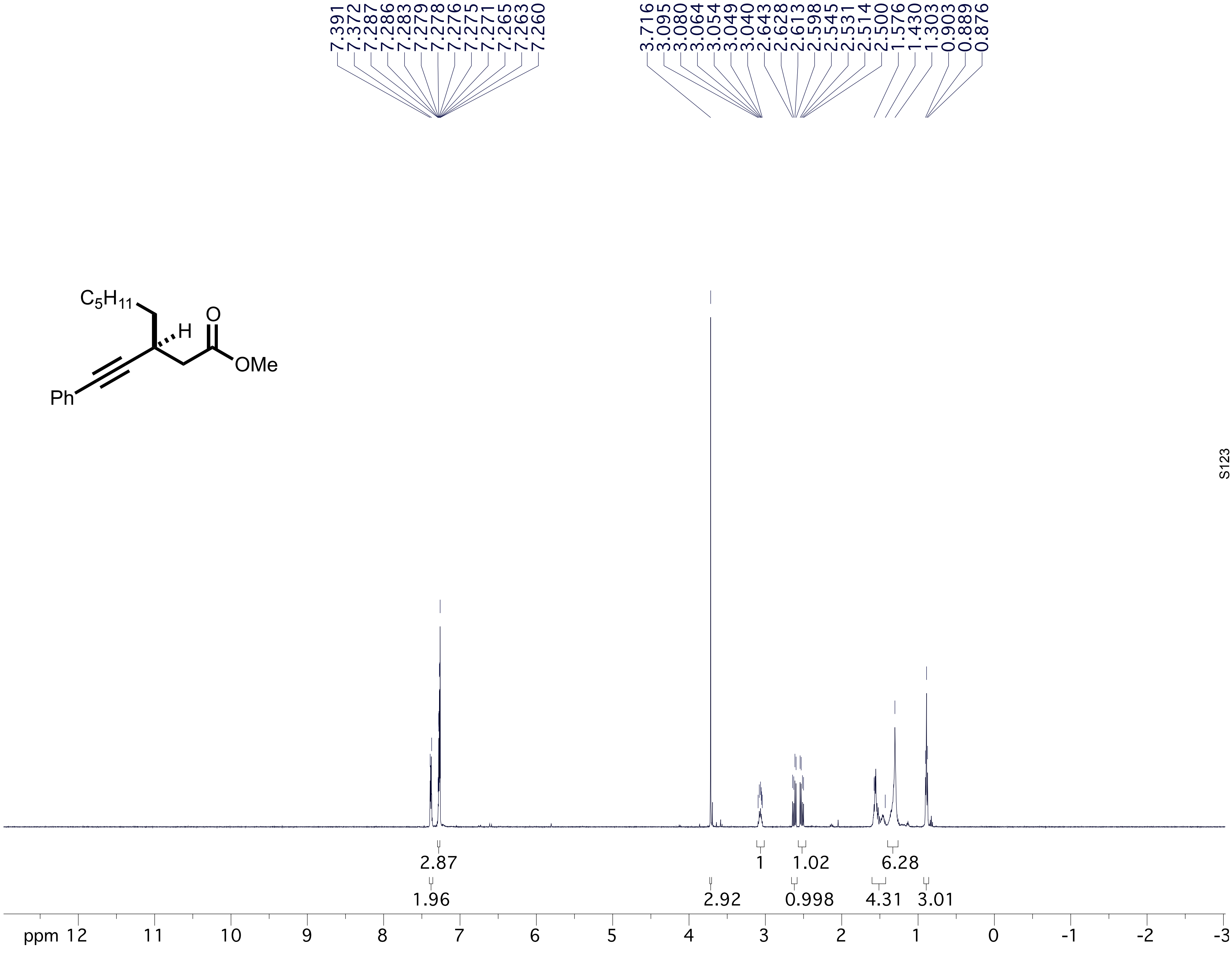
C5H11
H
O
OMe
Ph
7.391
7.372
7.287
7.286
7.283
7.279
7.278
7.276
7.275
7.271
7.265
7.263
7.260
3.716
3.095
3.080
3.064
3.054
3.049
3.040
2.643
2.628
2.613
2.598
2.545
2.531
2.514
2.500
1.576
1.430
1.303
0.903
0.889
0.876
2.87
1.96
2.92
1
1.02
0.998
6.28
4.31
3.01
ppm 12
11
10
9
8
7
6
5
4
3
2
1
0
-1
-2
-3

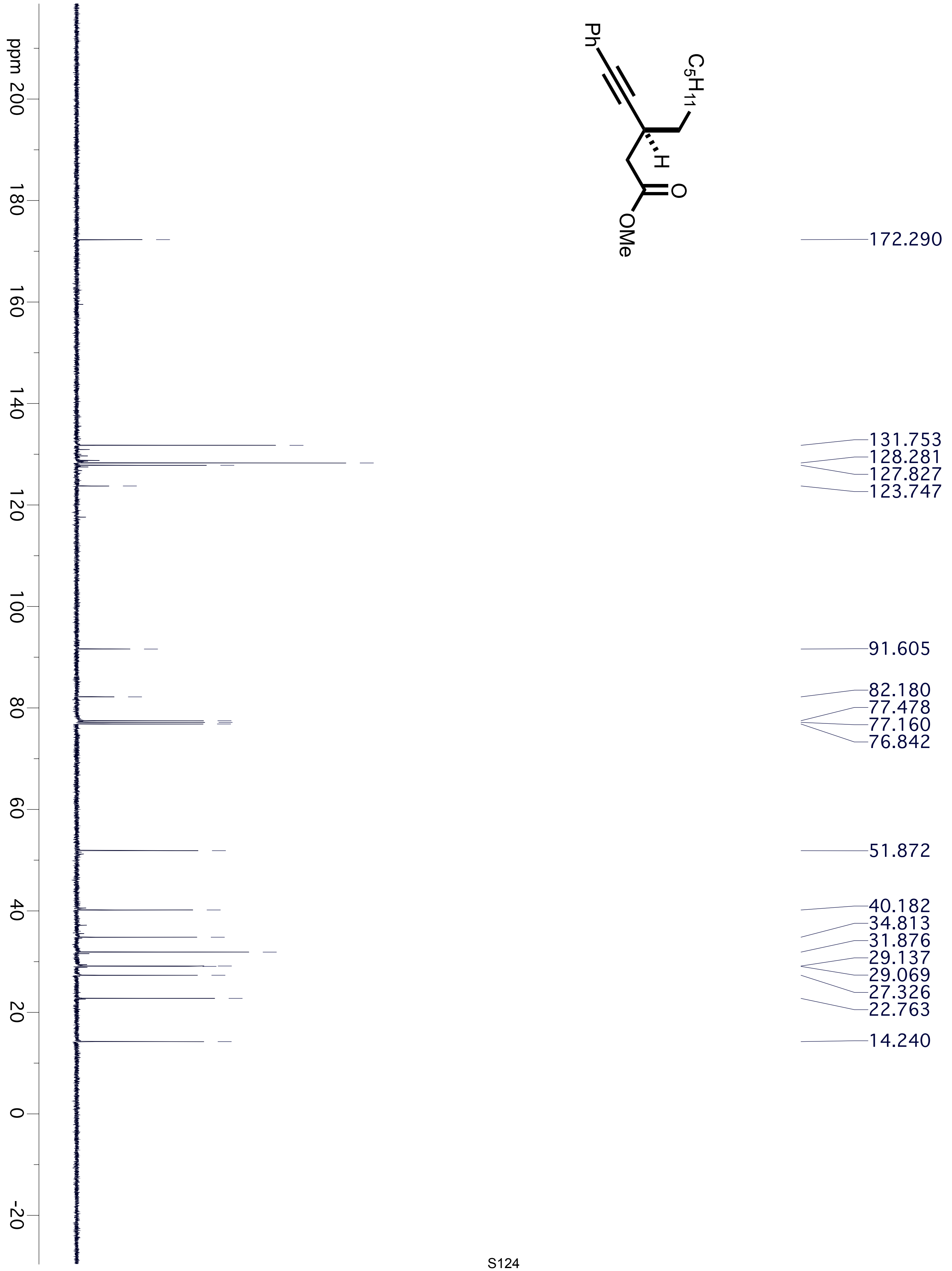
ppm
200
180
160
140
120
100
80
60
40
20
0
-20
Ph
C5H11
H
O
OMe
172.290
131.753
128.281
127.827
123.747
91.605
82.180
77.478
77.160
76.842
51.872
40.182
34.813
31.876
29.137
29.069
27.326
22.763
14.240

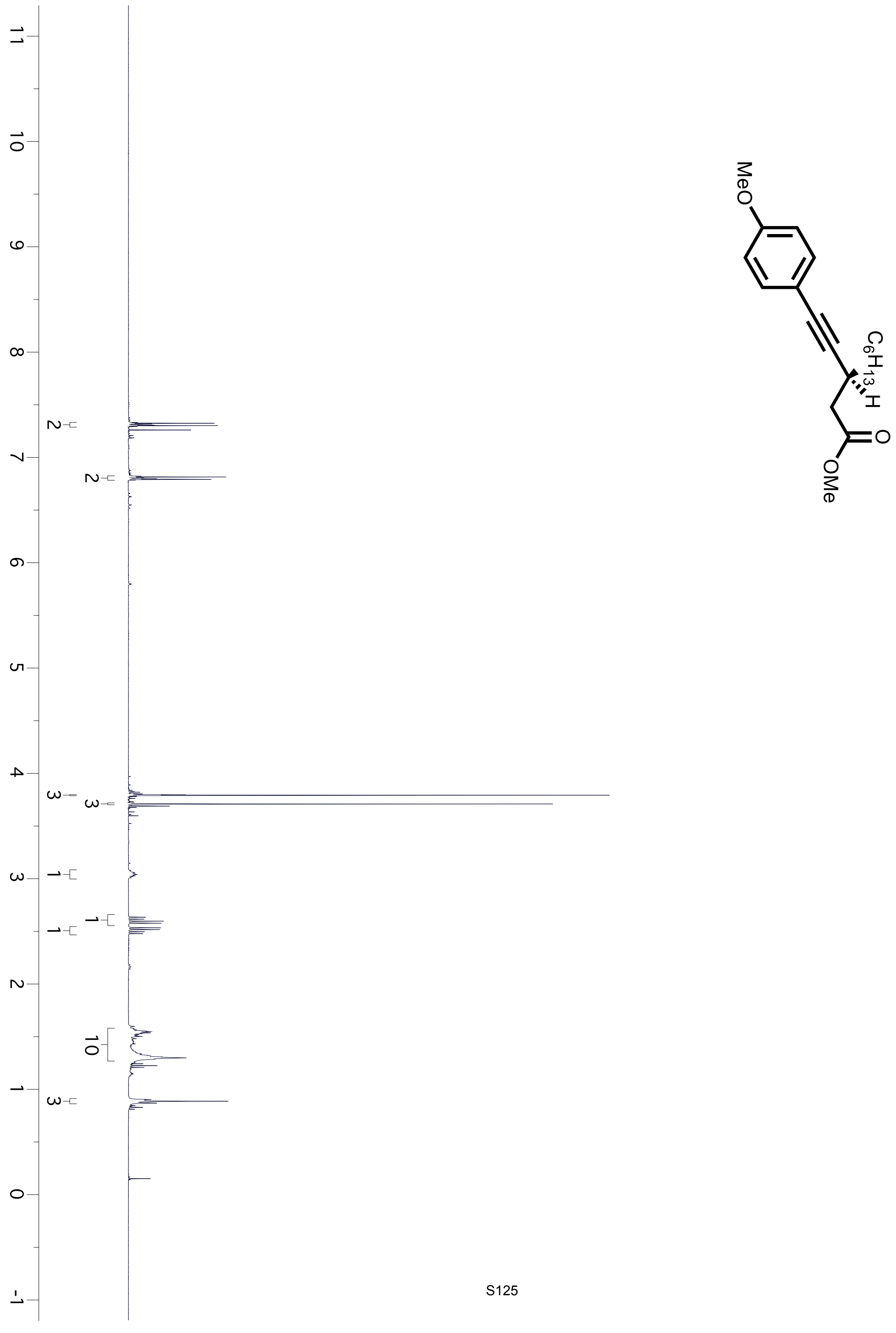
MeO
C6H13
H
O
OMe
11
10
9
8
7
6
5
4
3
2
1
0
-1
2
2
3
3
1
1
1
10
3

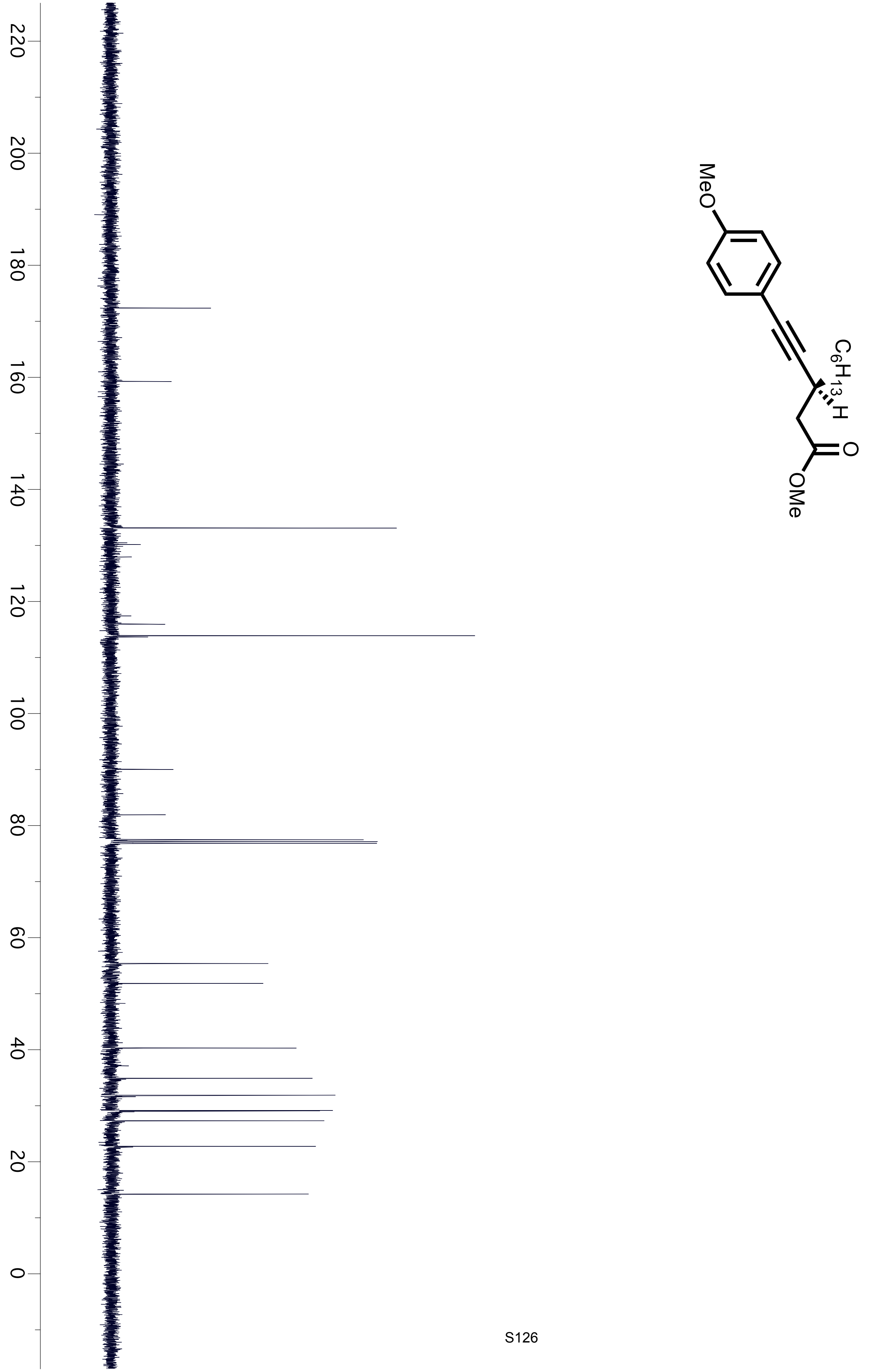
220
200
180
160
140
120
100
80
60
40
20
0
MeO
C6H13
H
O
OMe

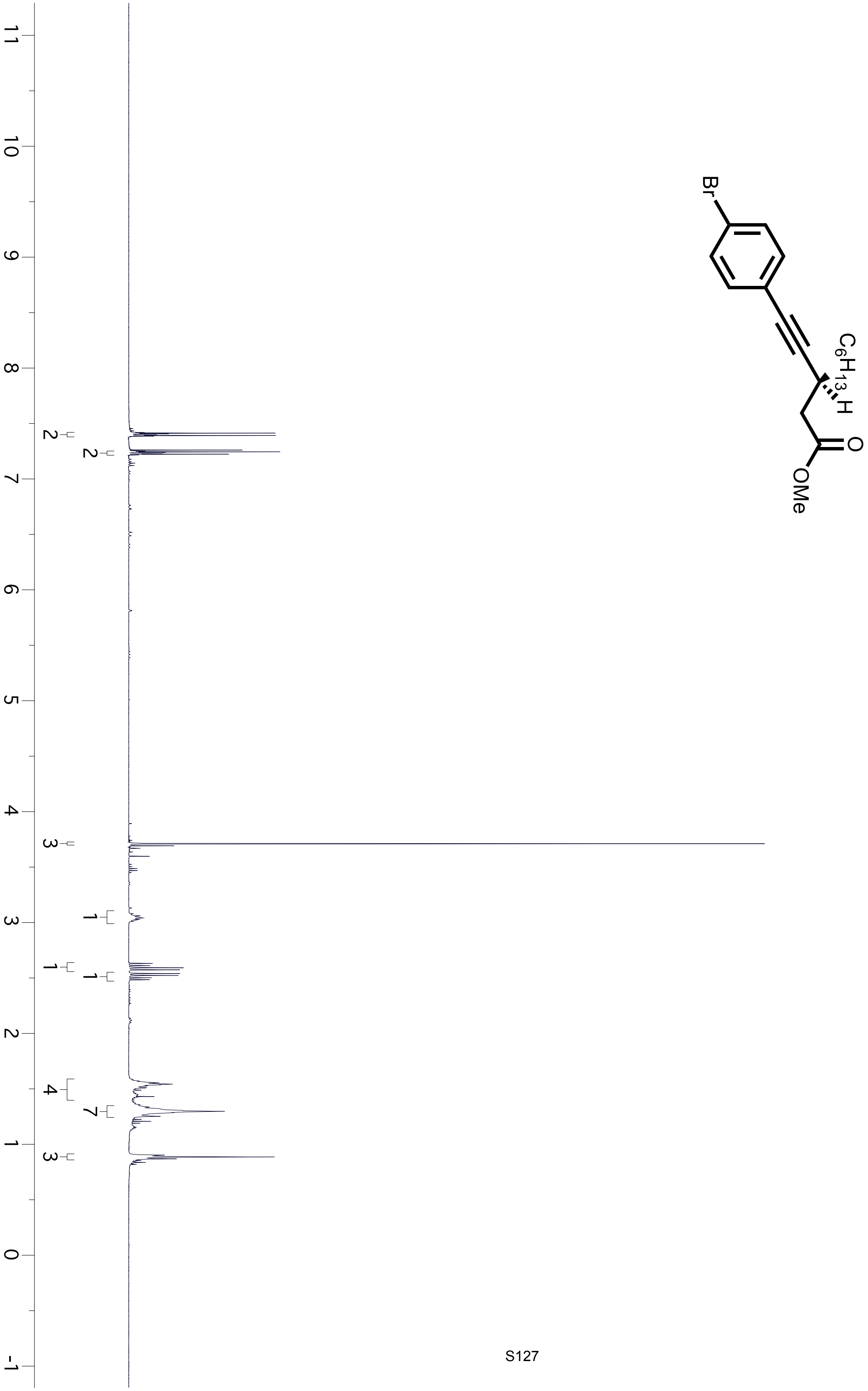
Br
C6H13
H
O
OMe
11
10
9
8
7
6
5
4
3
2
1
0
-1
2
2
3
1
1
1
4
7
3

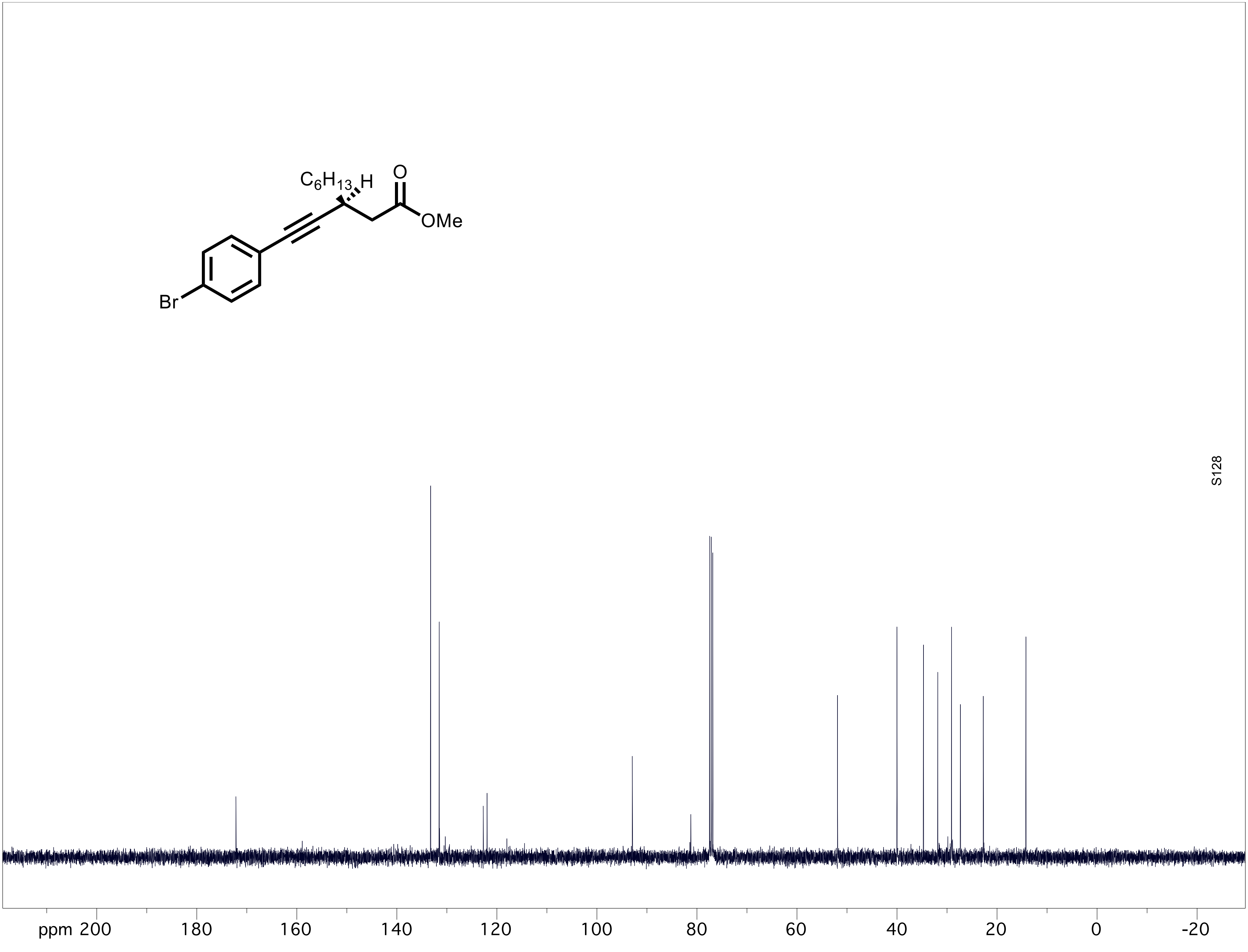

C_6H_{13} H
O
OMe
Br
ppm 200
180
160
140
120
100
80
60
40
20
0
-20

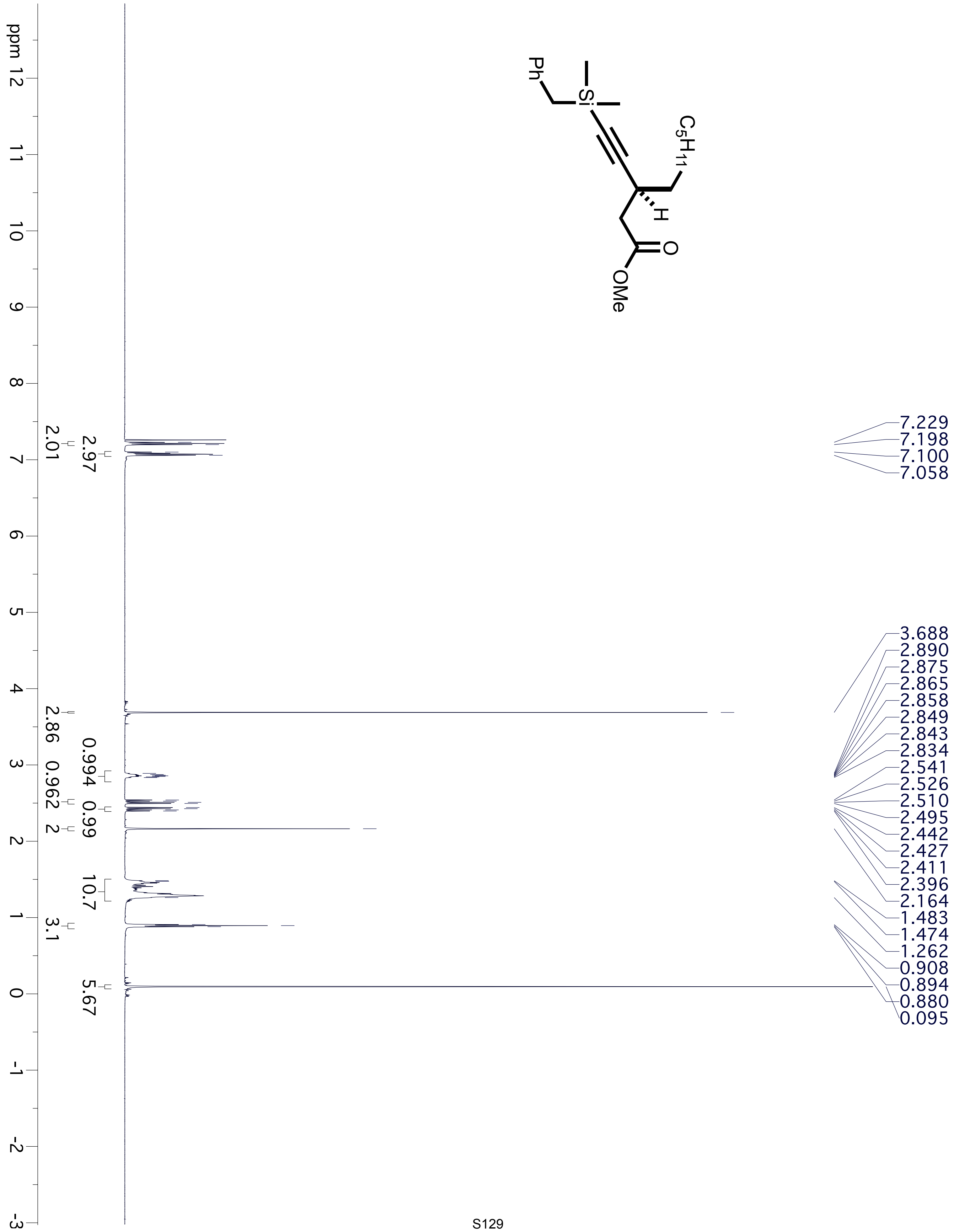
C5H11
H
O
OMe
Si
Ph
7.229
7.198
7.100
7.058
3.688
2.890
2.875
2.865
2.858
2.849
2.843
2.834
2.541
2.526
2.510
2.495
2.442
2.427
2.411
2.396
2.164
1.483
1.474
1.262
0.908
0.894
0.880
0.095
2.01
2.97
2.86
0.994
0.962
0.99
2
10.7
3.1
5.67
ppm 12
11
10
9
8
7
6
5
4
3
2
1
0
-1
-2
-3

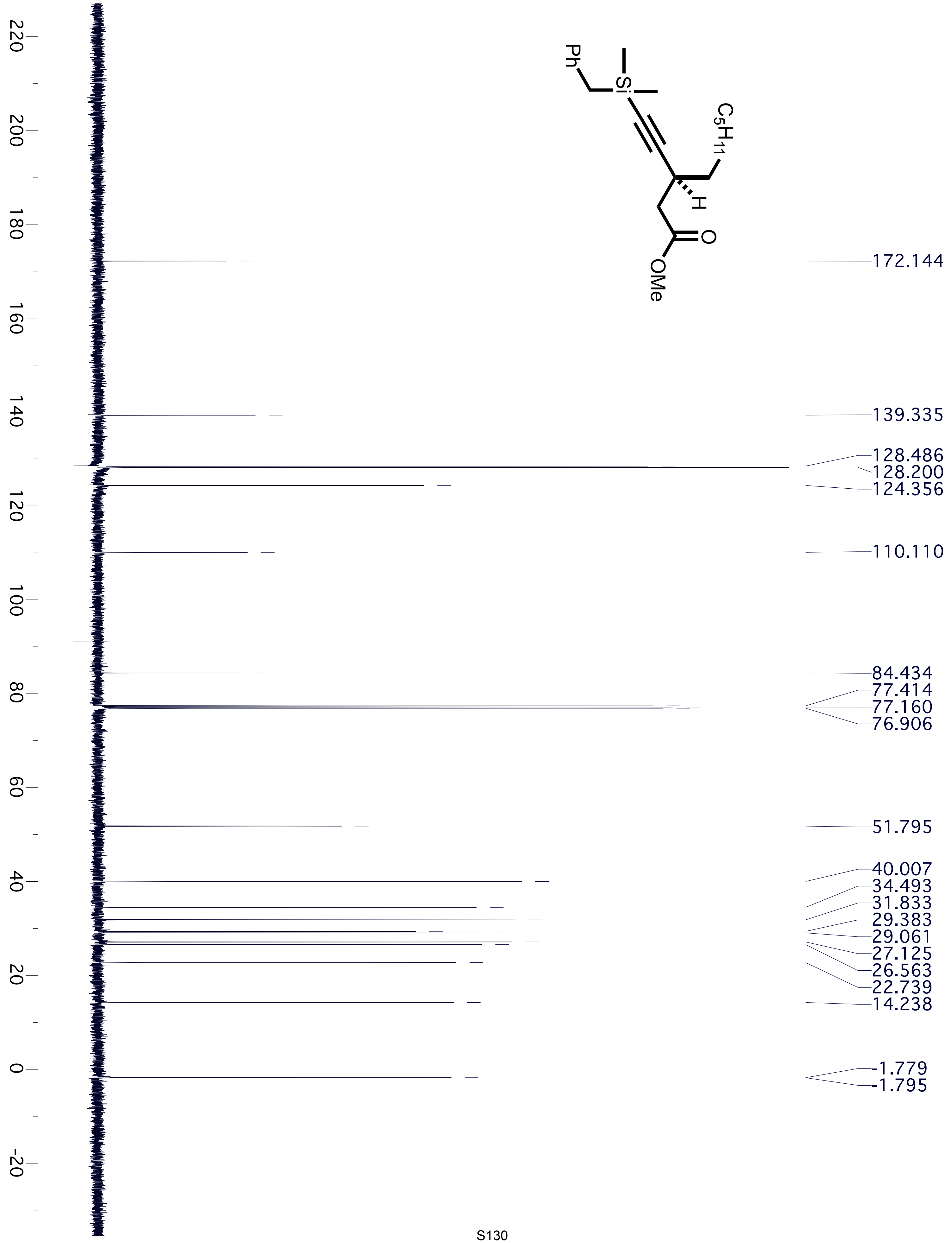

Ph
Si
C_5H_{11}
H
O
OMe
172.144
139.335
128.486
128.200
124.356
110.110
84.434
77.414
77.160
76.906
51.795
40.007
34.493
31.833
29.383
29.061
27.125
26.563
22.739
14.238
-1.779
-1.795
220
200
180
160
140
120
100
80
60
40
20
0
-20

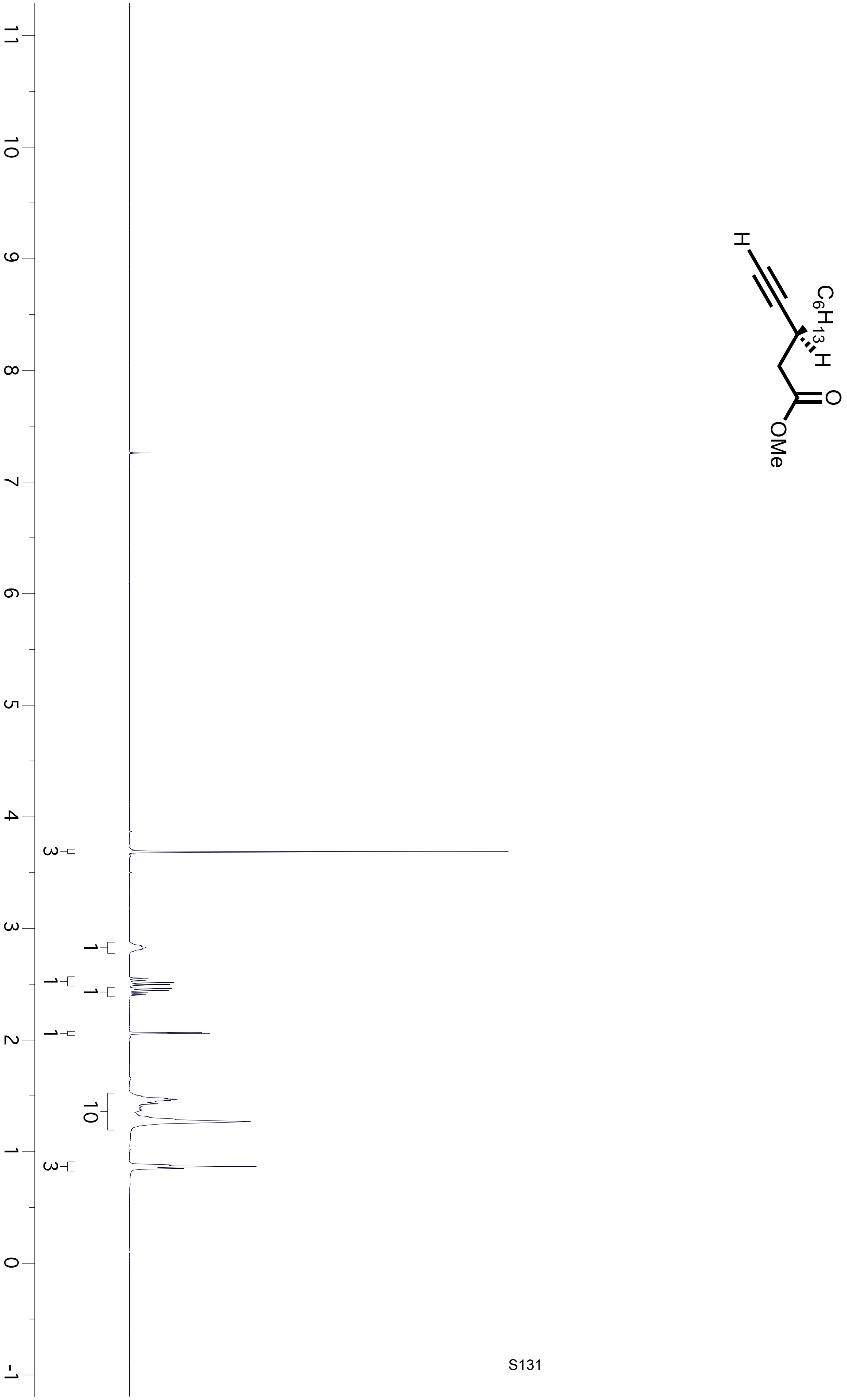

H
C_6H_{13}
H
O
OMe
11
10
9
8
7
6
5
4
3
2
1
0
-1
3
1
1
1
1
10
3

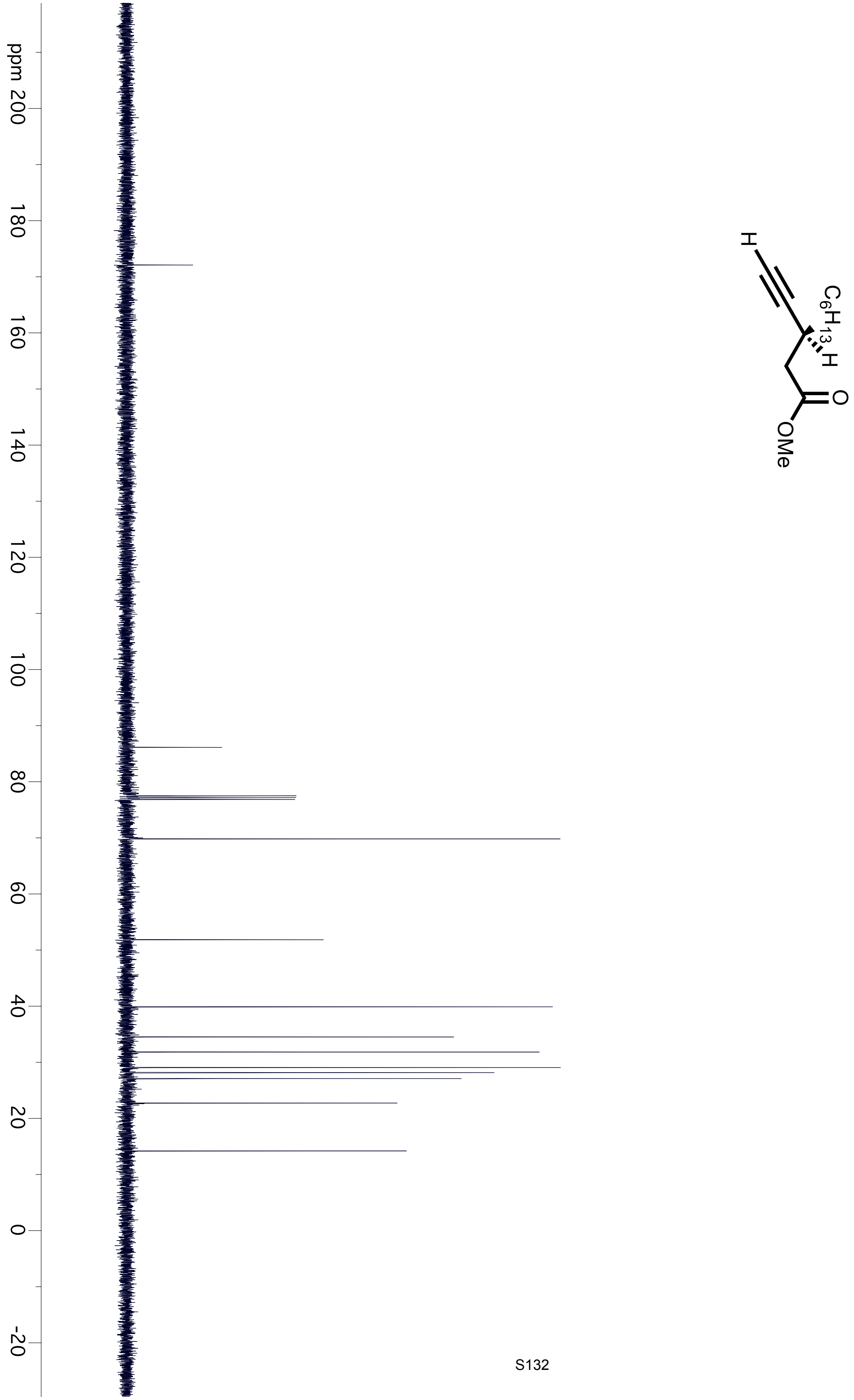

ppm 200
180
160
140
120
100
80
60
40
20
0
-20
H
C_6H_{13}
H
O
OMe

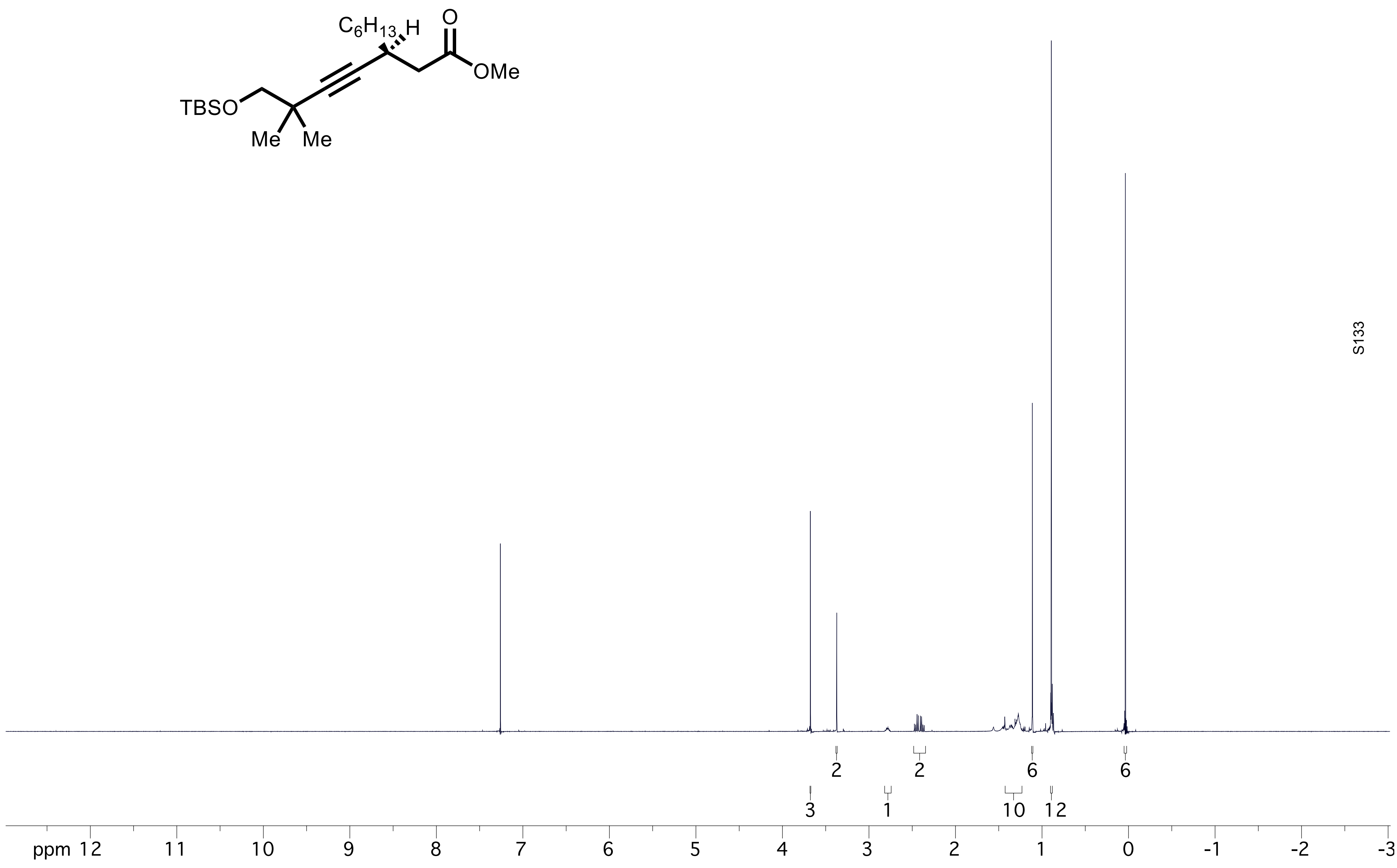
TBSO
Me
Me
C_6H_{13}
H
O
OMe
2
2
6
6
3
1
10
12
ppm 12
11
10
9
8
7
6
5
4
3
2
1
0
-1
-2
-3

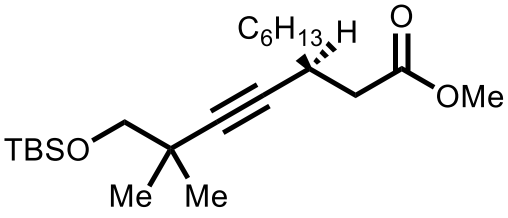

TBSO
Me
Me
C_6H_{13}
H
O
OMe

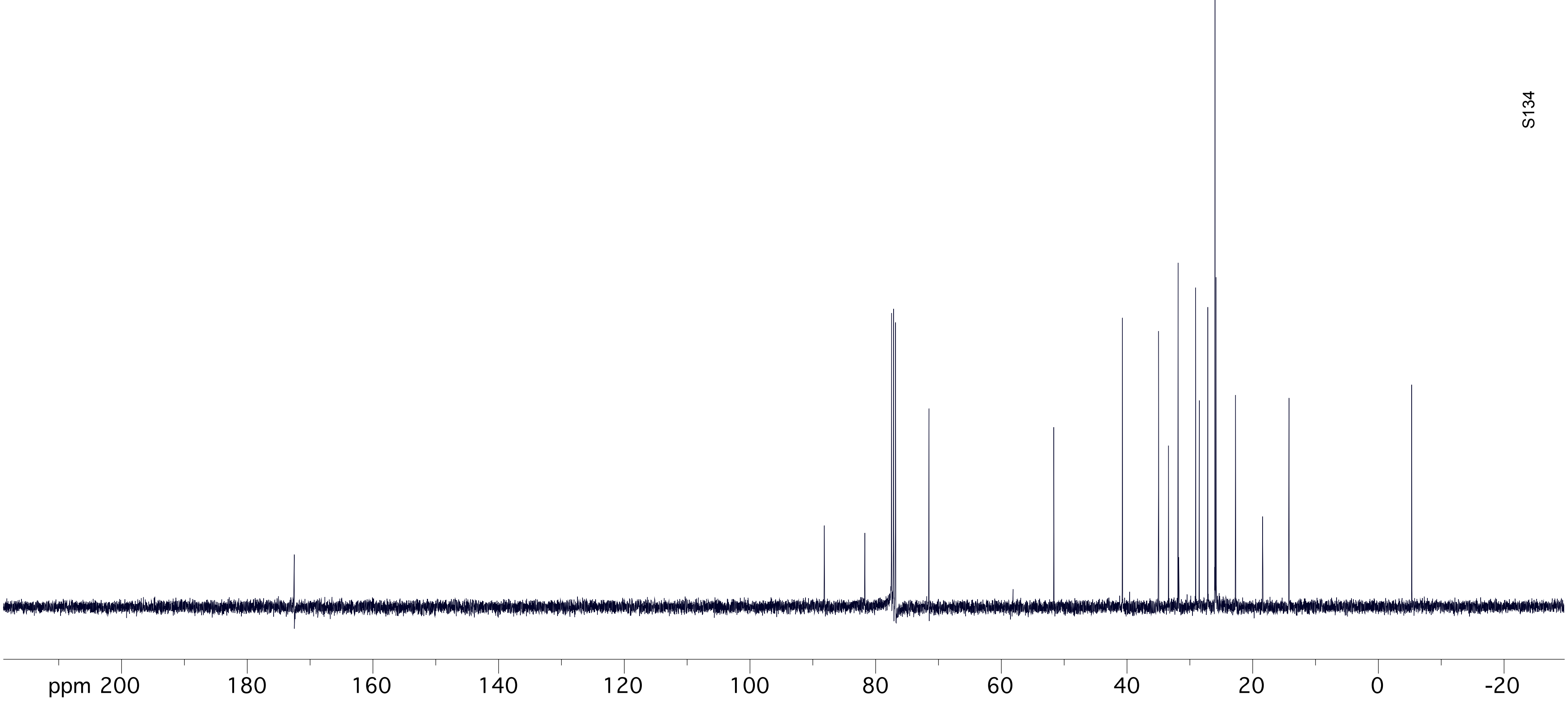

ppm 200
180
160
140
120
100
80
60
40
20
0
-20

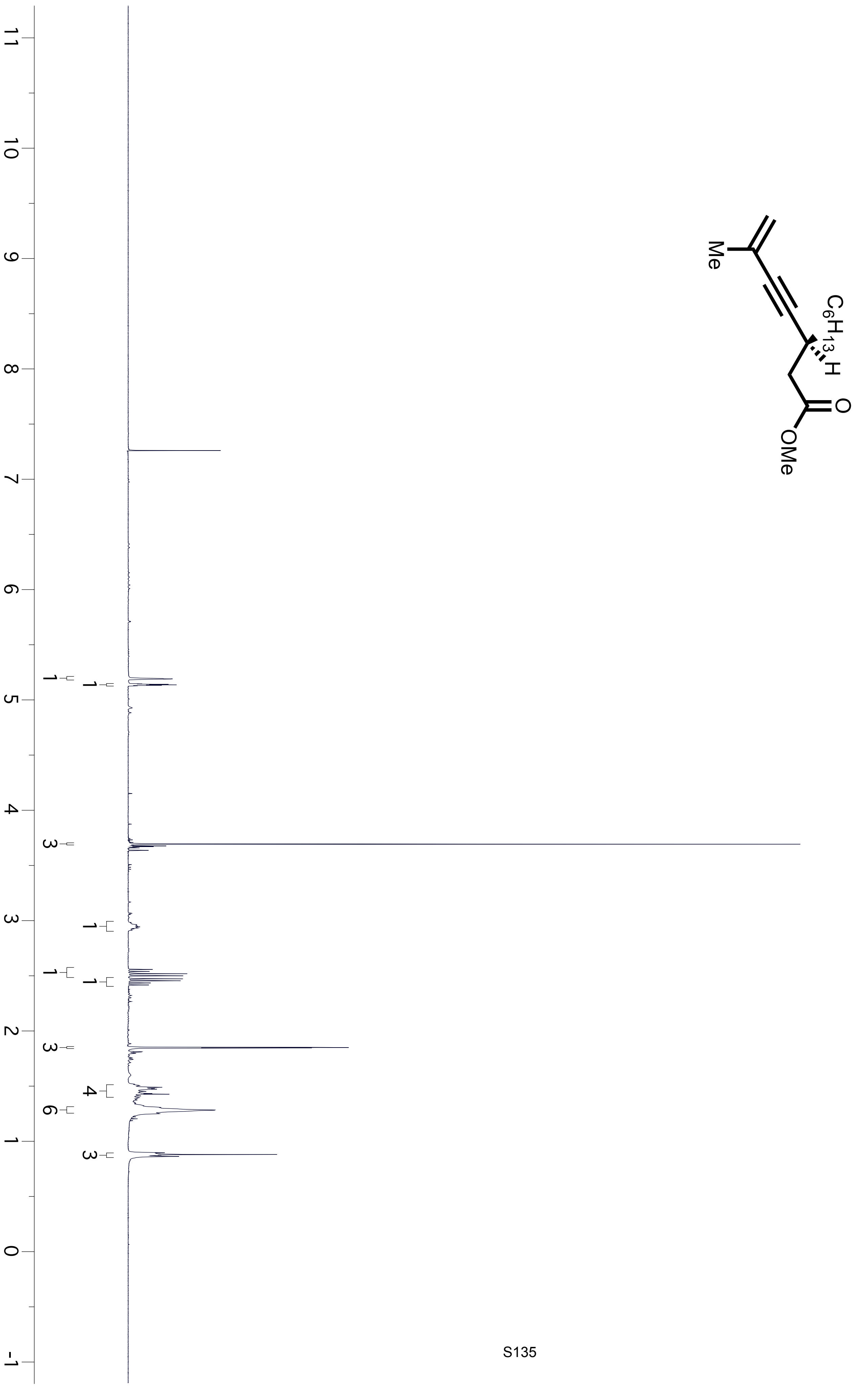

Me
C_6H_{13}
H
O
OMe
11
10
9
8
7
6
5
4
3
2
1
0
-1

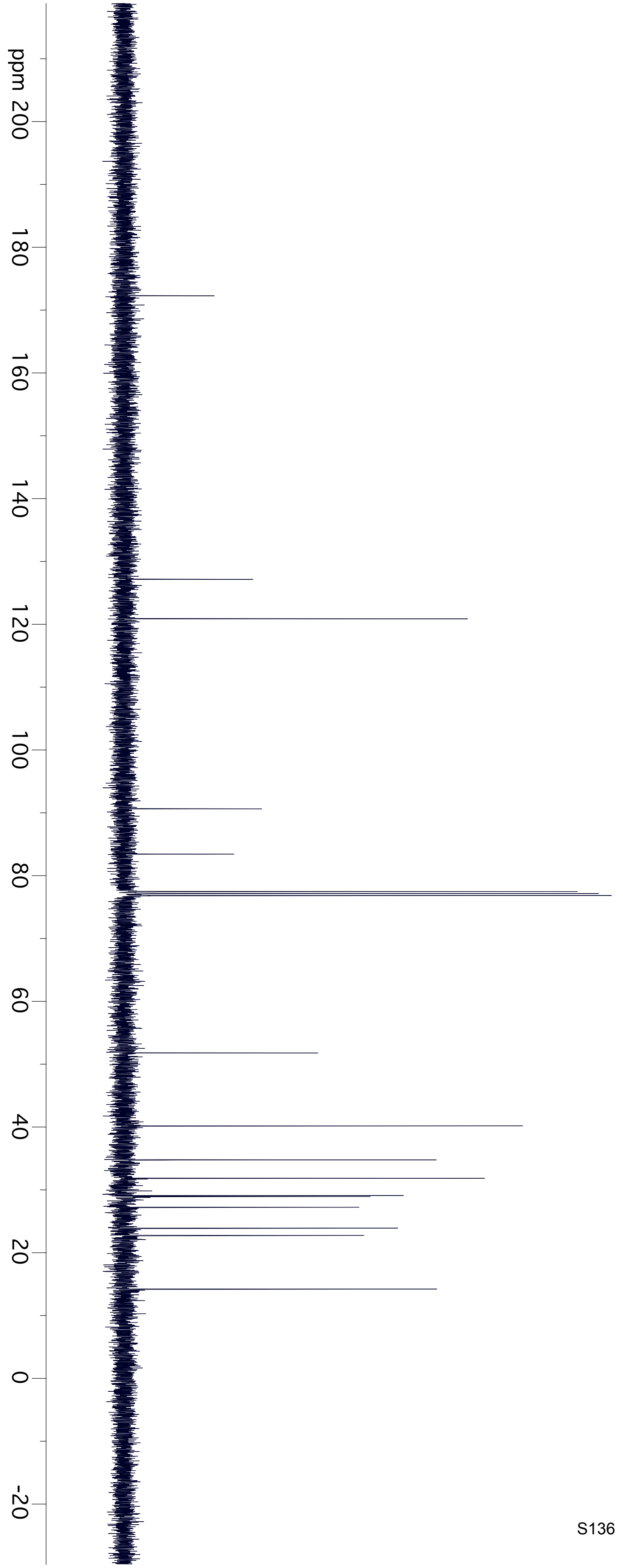
ppm 200
180
160
140
120
100
80
60
40
20
0
-20

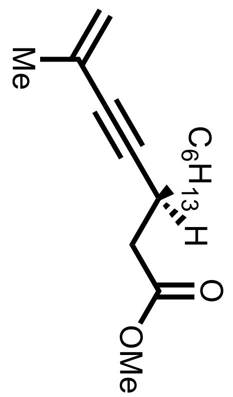
Me
C6H13
H
O
OMe

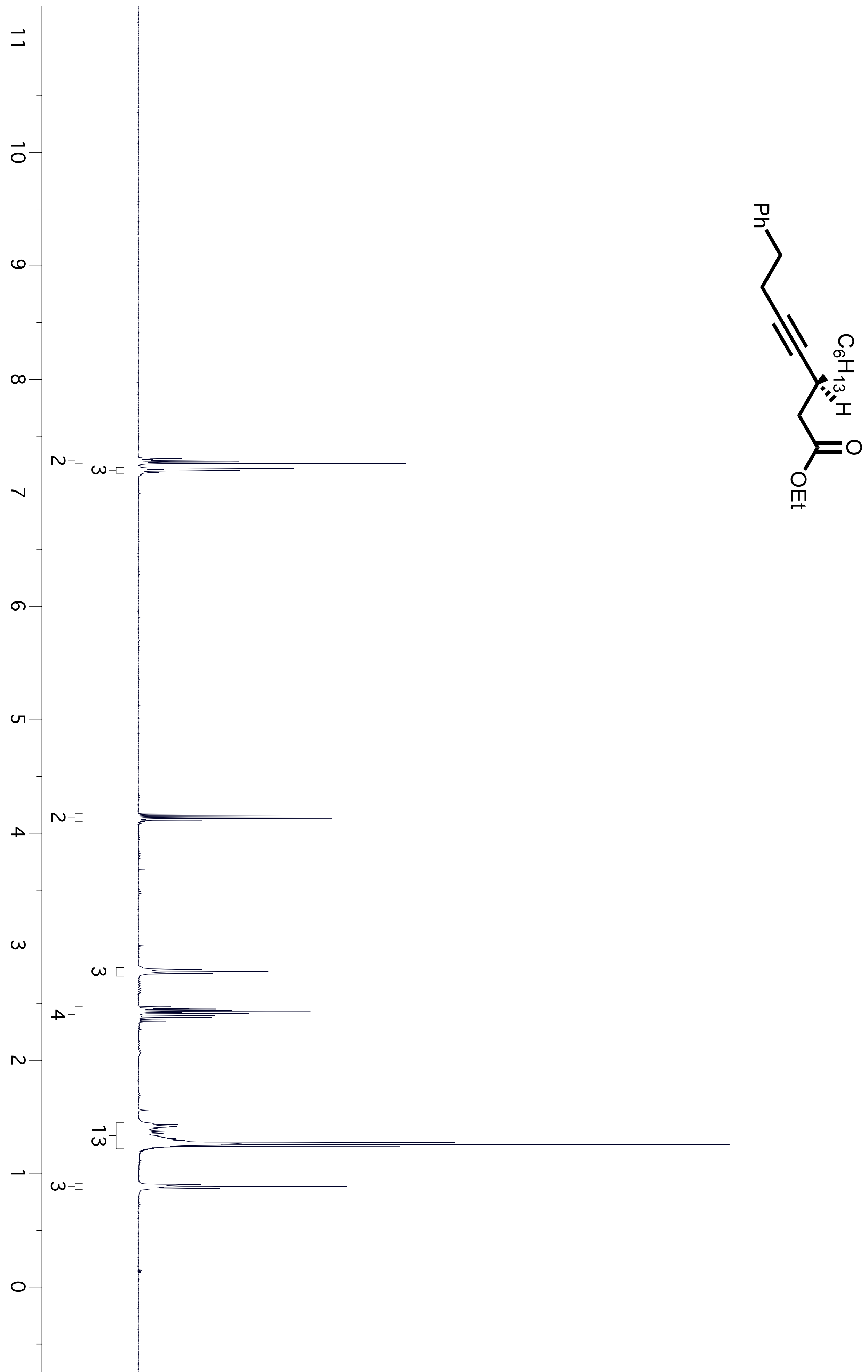
Ph
C6H13
H
O
OEt
11
10
9
8
7
6
5
4
3
2
1
0
-1
2
3
2
3
4
13
3

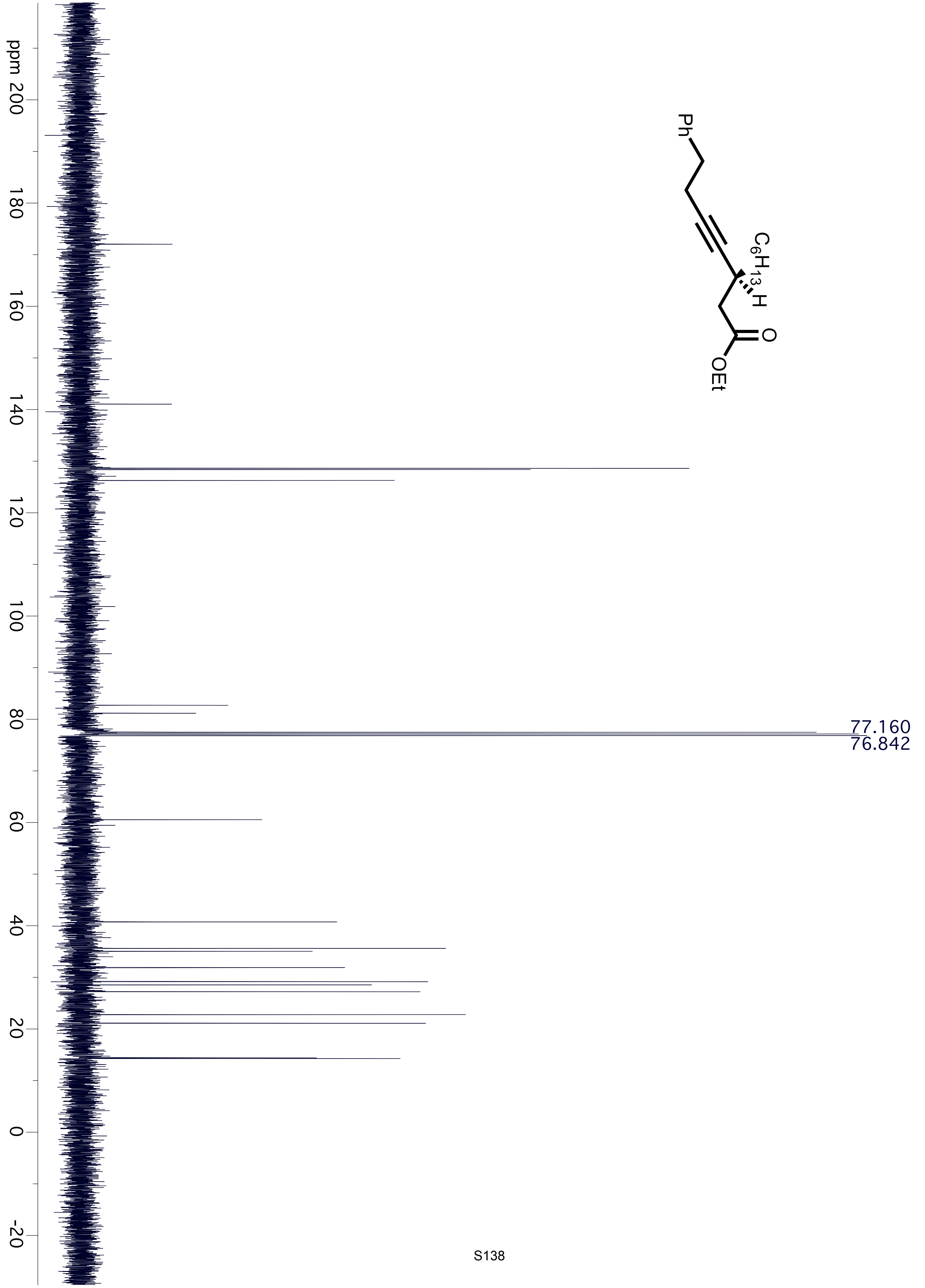

ppm 200
180
160
140
120
100
80
60
40
20
0
-20
77.160
76.842
Ph
C_6H_{13}
H
O
OEt

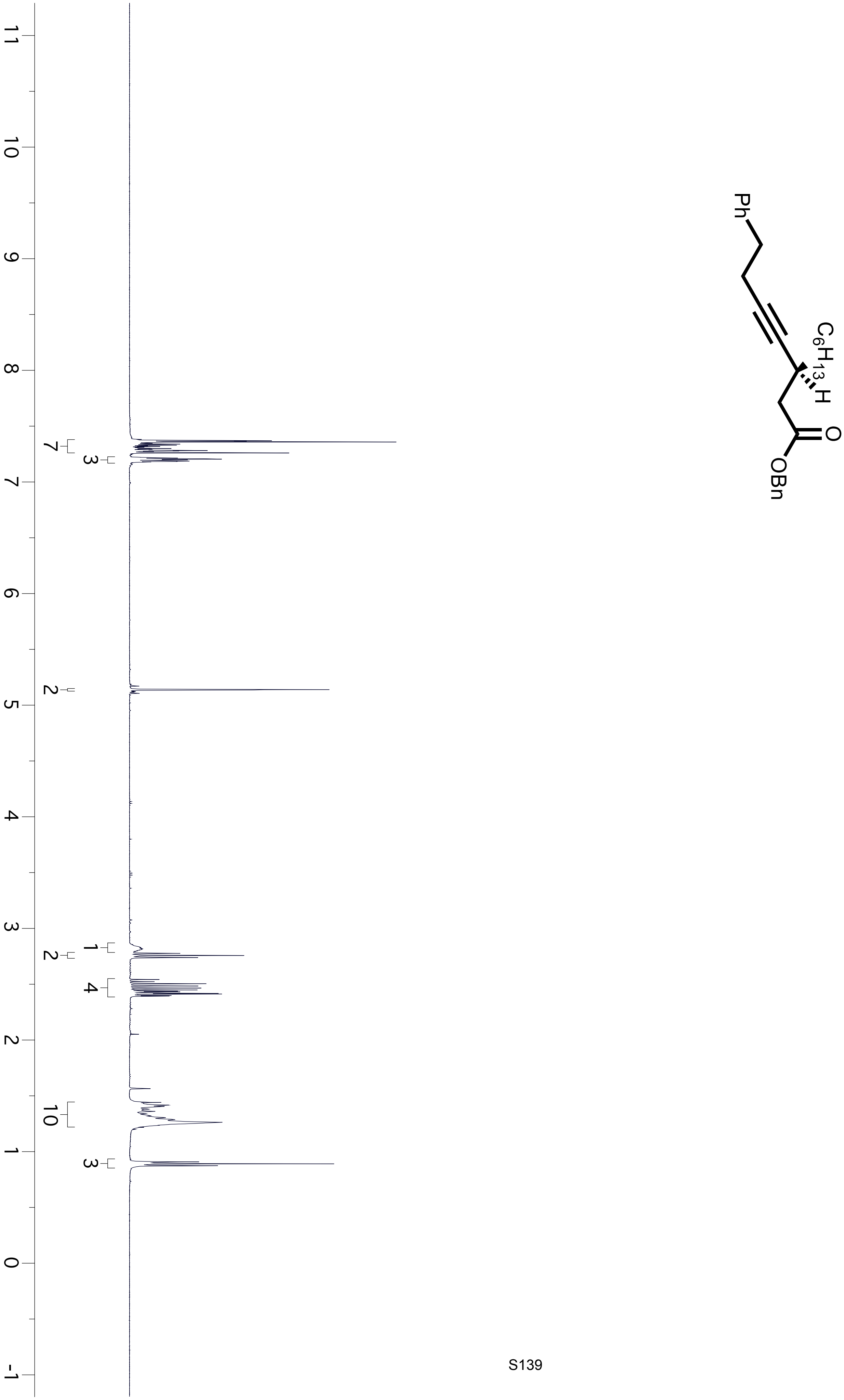
Ph
C6H13
H
O
OBn
11
10
9
8
7
6
5
4
3
2
1
0
-1
7
3
2
1
2
4
10
3

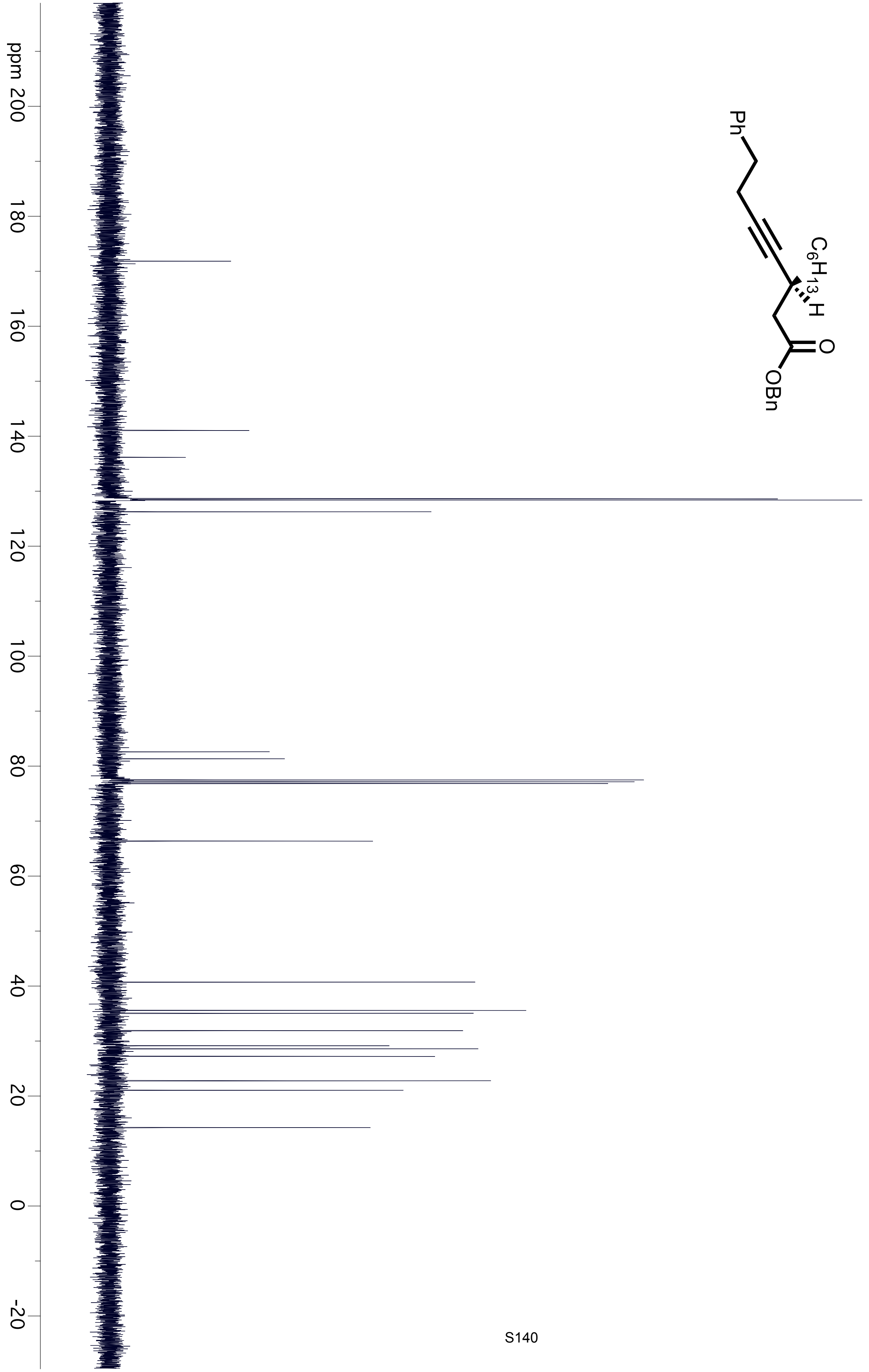
ppm 200
180
160
140
120
100
80
60
40
20
0
-20
Ph
C6H13
H
O
OBn

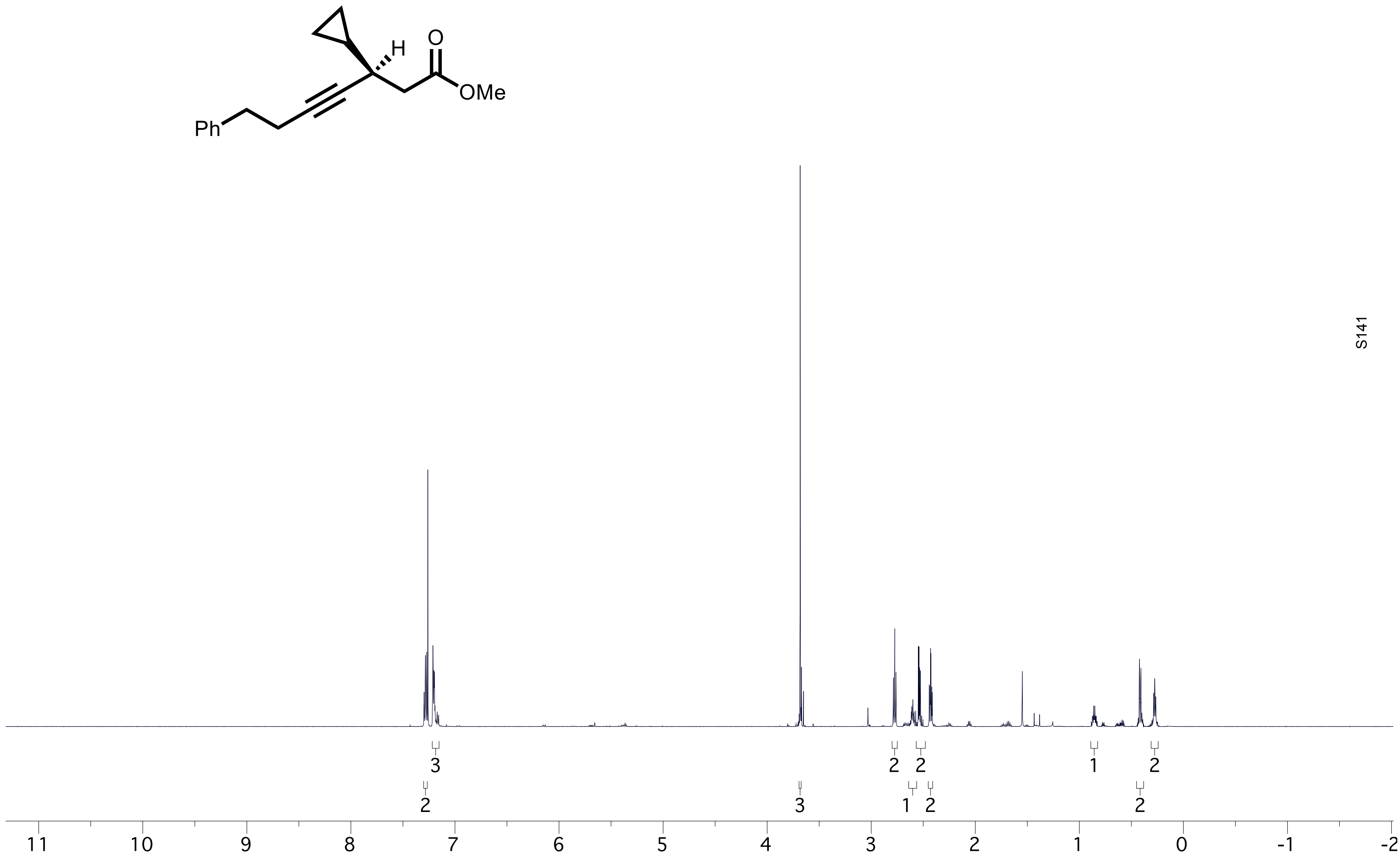
Ph
H
O
OMe
3
2
2
1
2
2
2
3
1
2
2
11 10 9 8 7 6 5 4 3 2 1 0 -1 -2

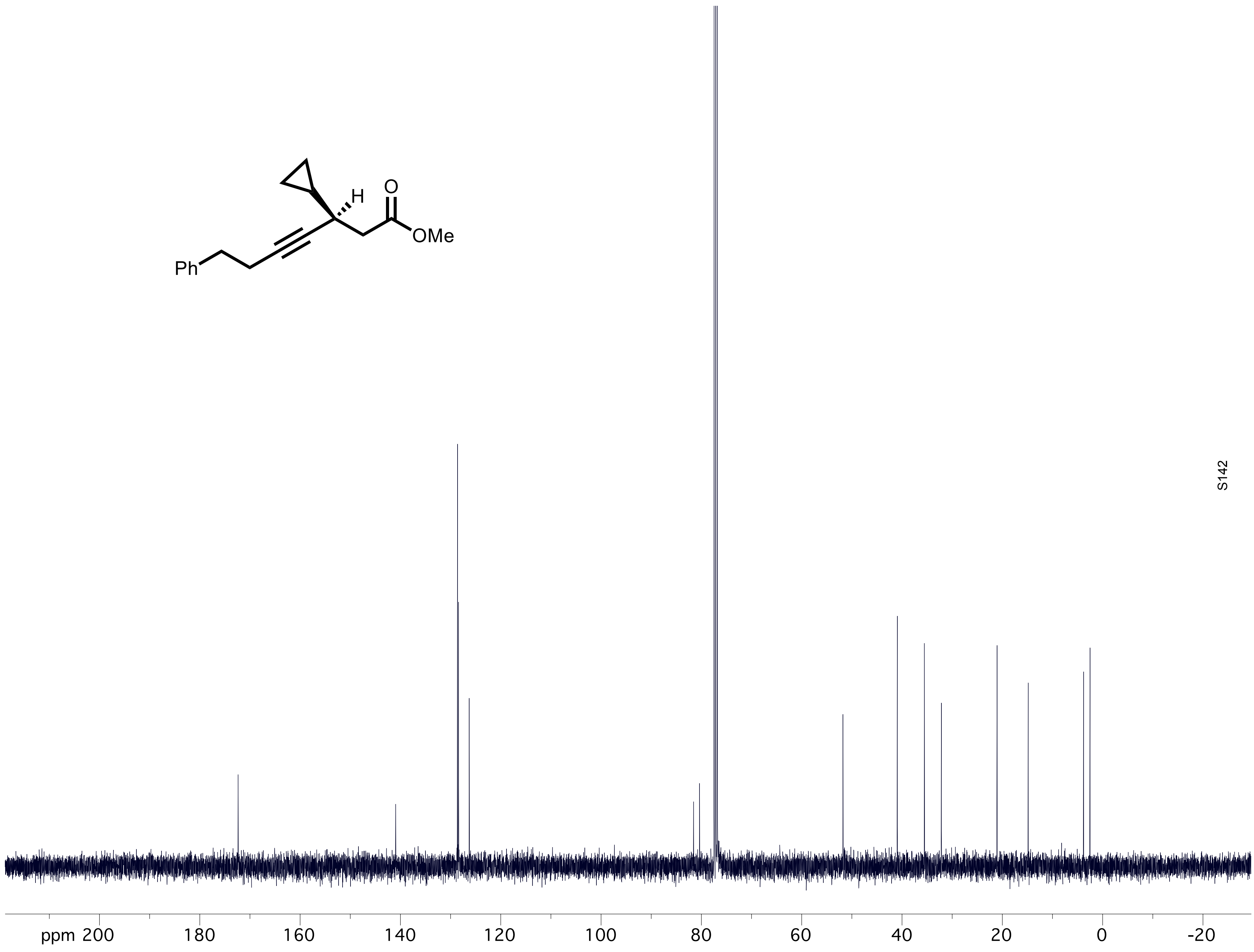
Ph
H
O
OMe
ppm 200
180
160
140
120
100
80
60
40
20
0
-20

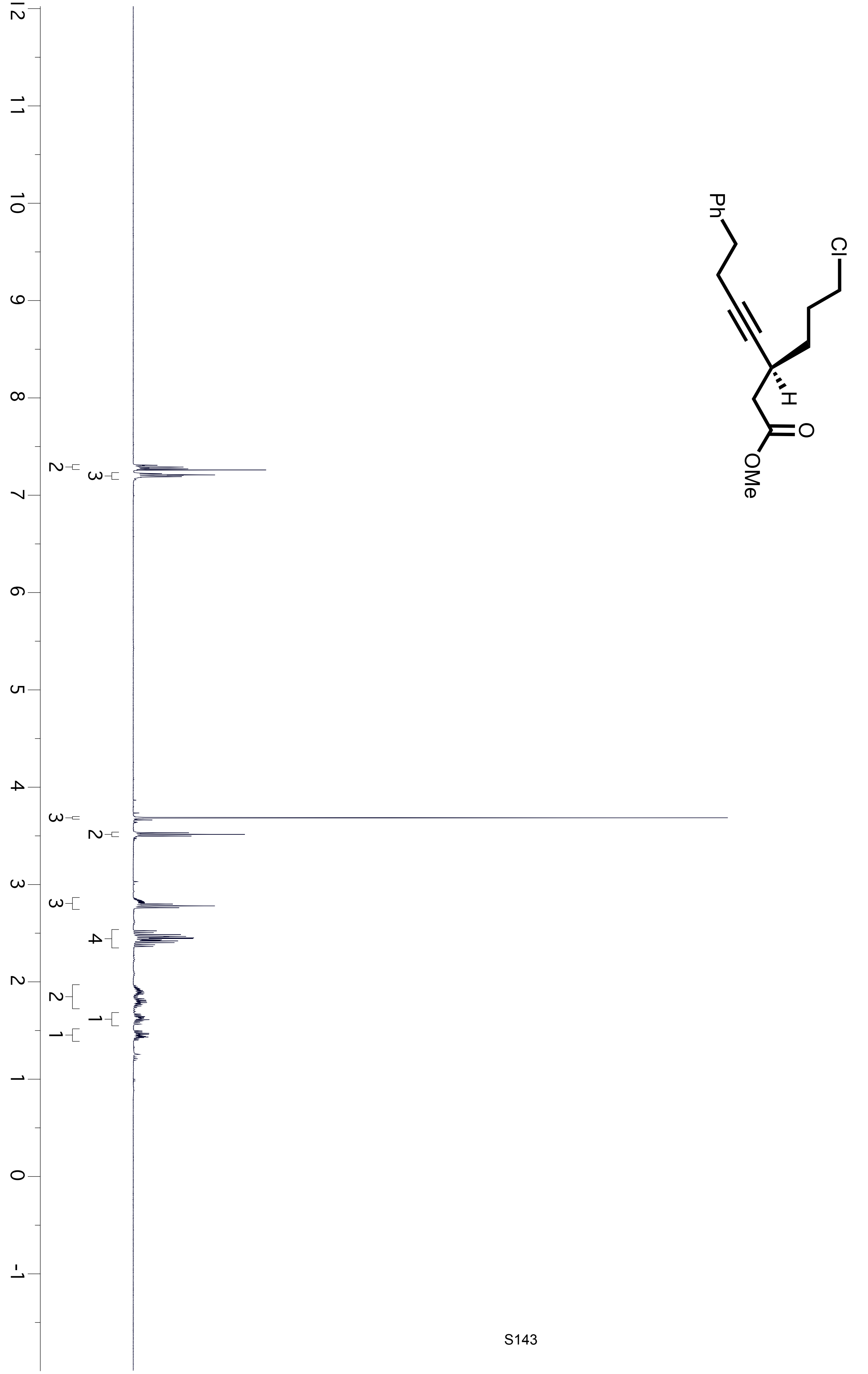

Ph
Cl
H
O
OMe

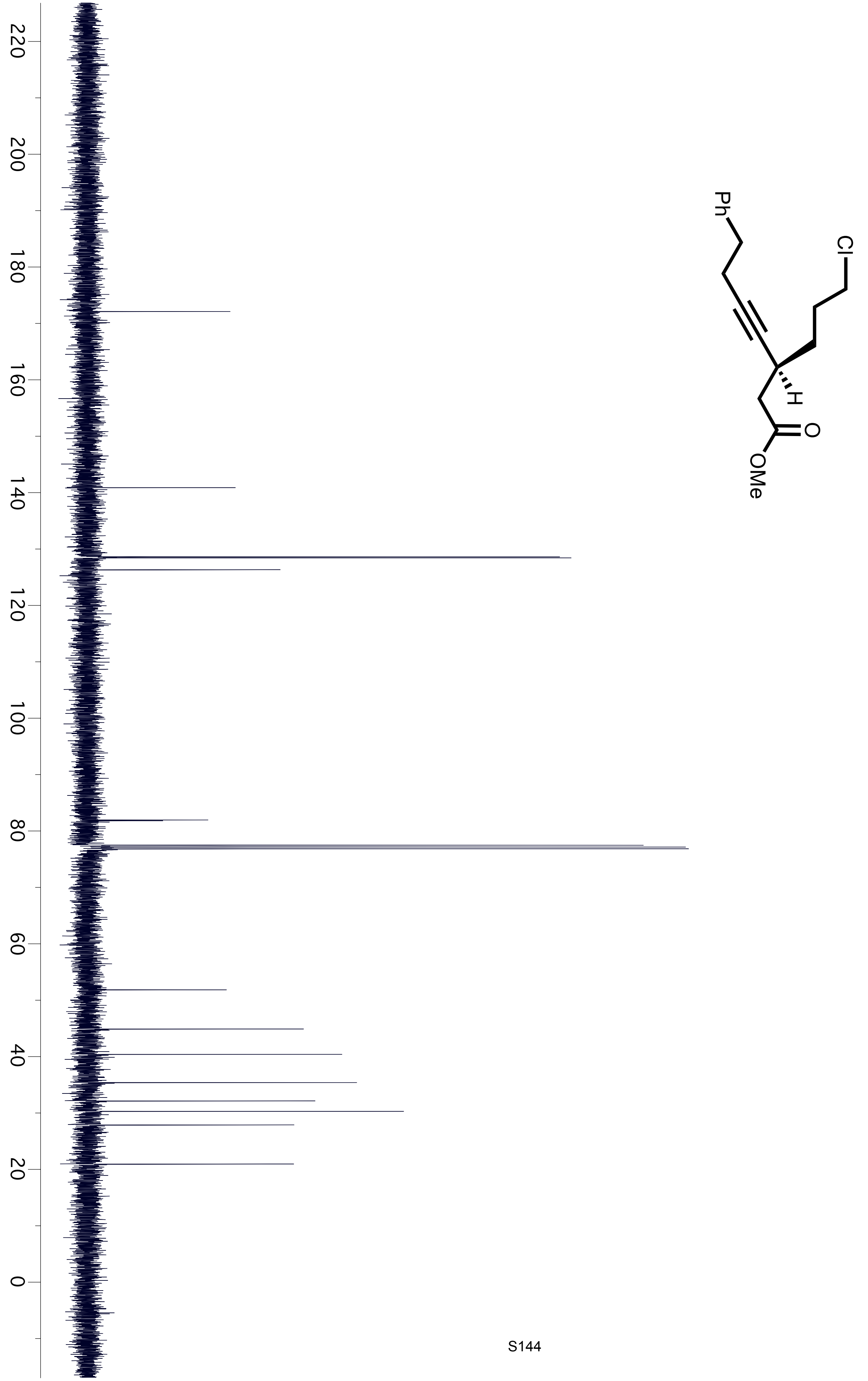

220
200
180
160
140
120
100
80
60
40
20
0
Ph
Cl
H
O
OMe

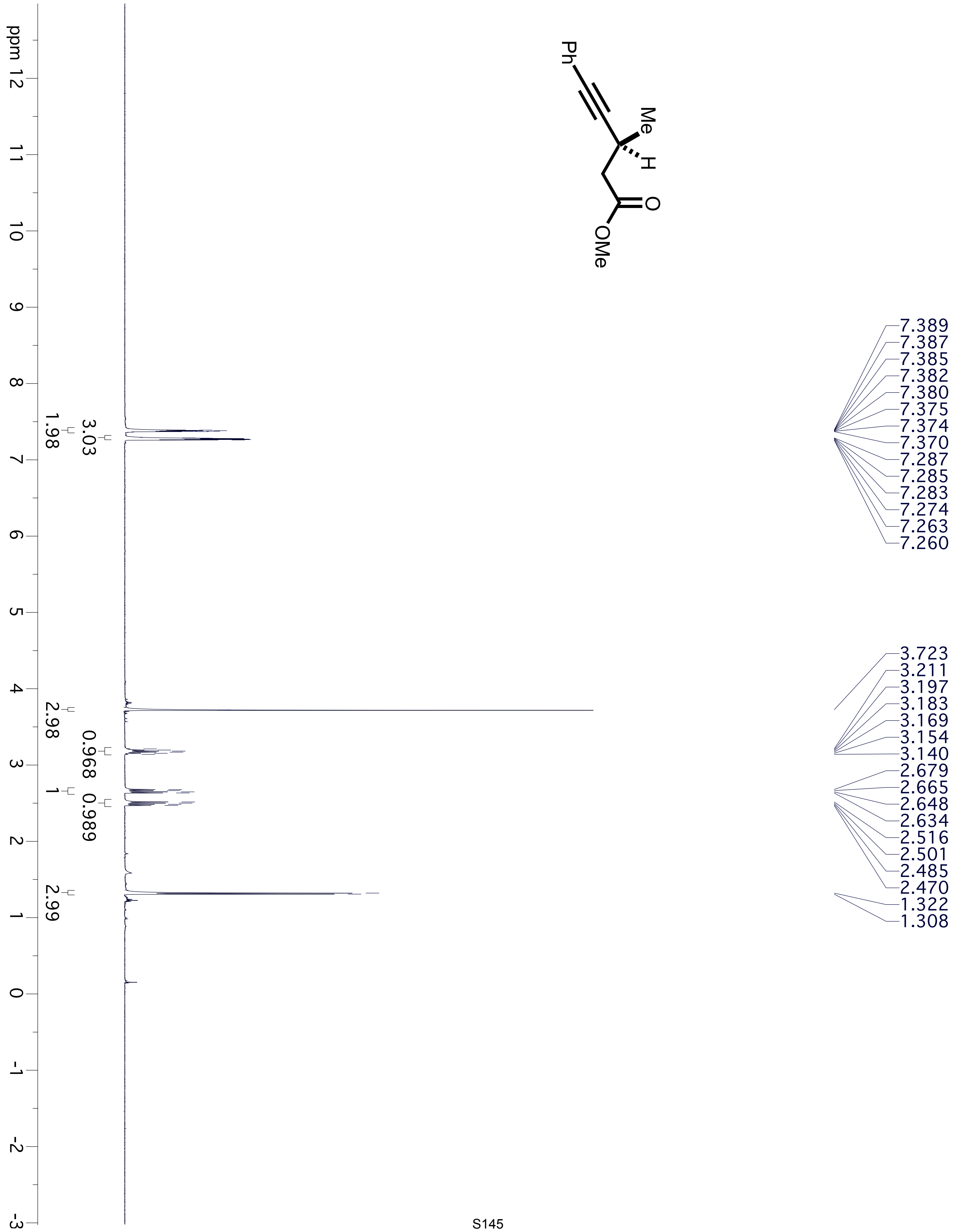
Ph
Me
H
O
OMe
7.389
7.387
7.385
7.382
7.380
7.375
7.374
7.370
7.287
7.285
7.283
7.274
7.263
7.260
3.723
3.211
3.197
3.183
3.169
3.154
3.140
2.679
2.665
2.648
2.634
2.516
2.501
2.485
2.470
1.322
1.308
1.98
3.03
2.98
0.968
1
0.989
2.99
ppm 12
11
10
9
8
7
6
5
4
3
2
1
0
-1
-2
-3

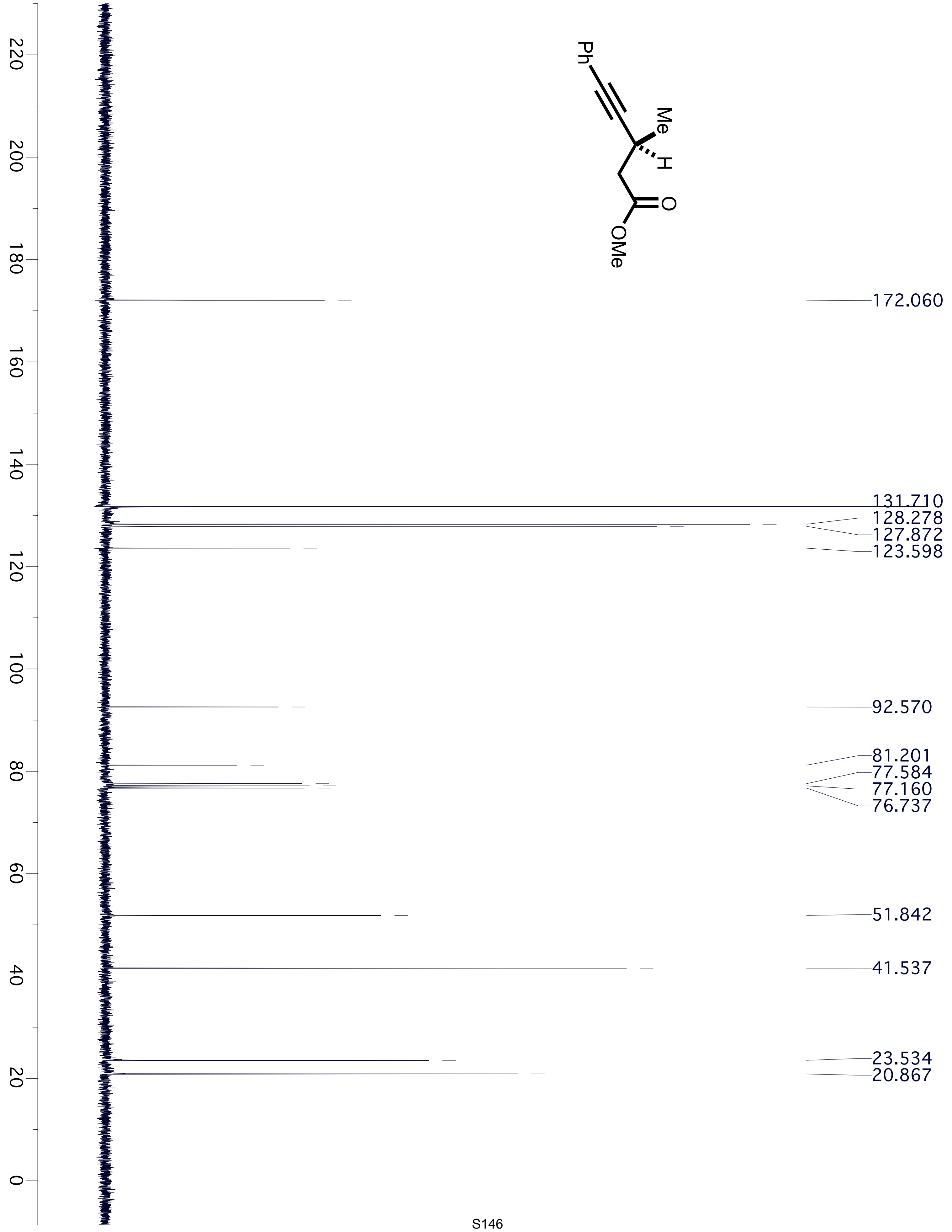
Ph
Me
H
O
OMe
172.060
131.710
128.278
127.872
123.598
92.570
81.201
77.584
77.160
76.737
51.842
41.537
23.534
20.867
220
200
180
160
140
120
100
80
60
40
20
0

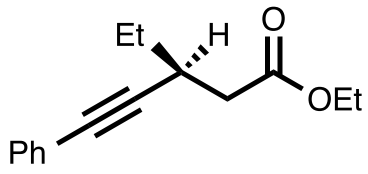
Et
H
O
OEt
Ph

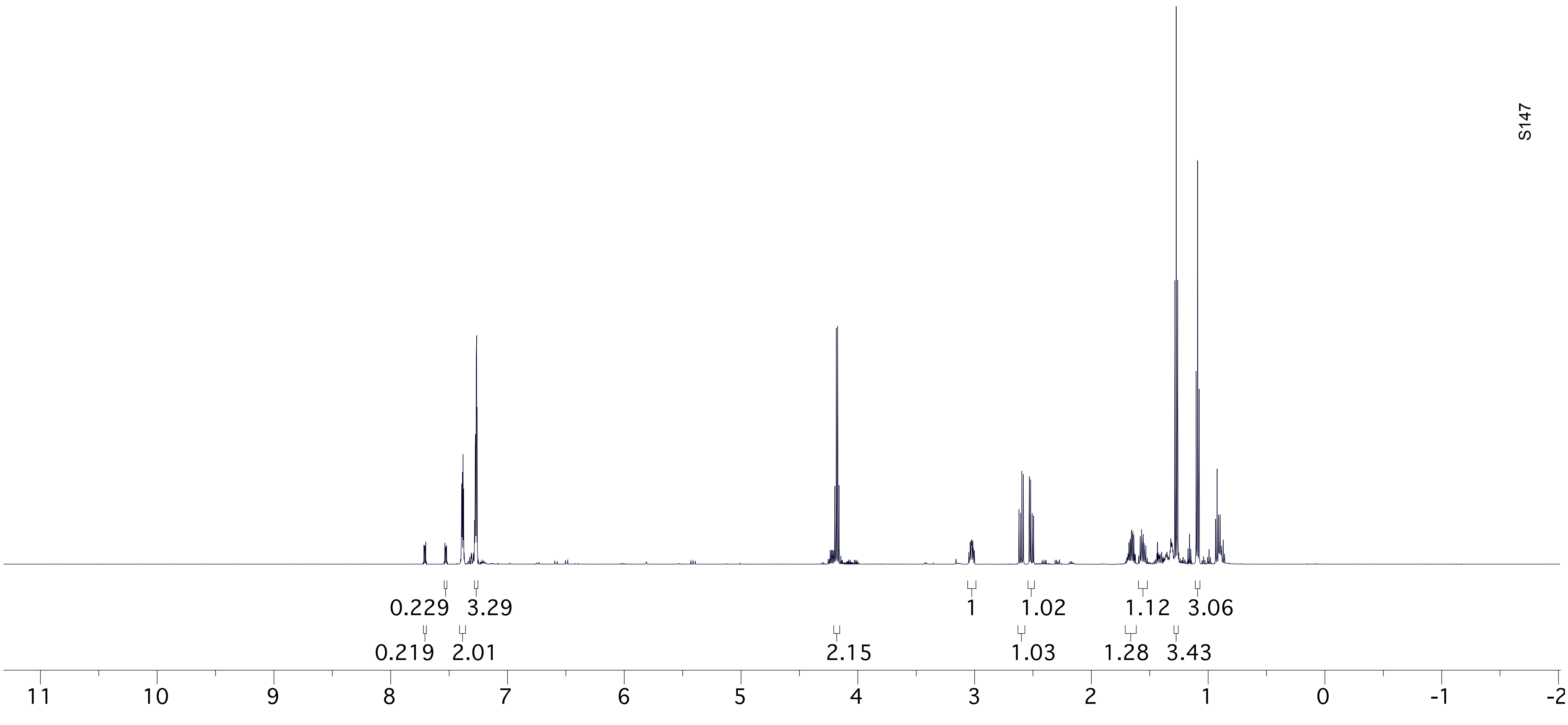
0.229
3.29
1
1.02
1.12
3.06
0.219
2.01
2.15
1.03
1.28
3.43
11
10
9
8
7
6
5
4
3
2
1
0
-1
-2

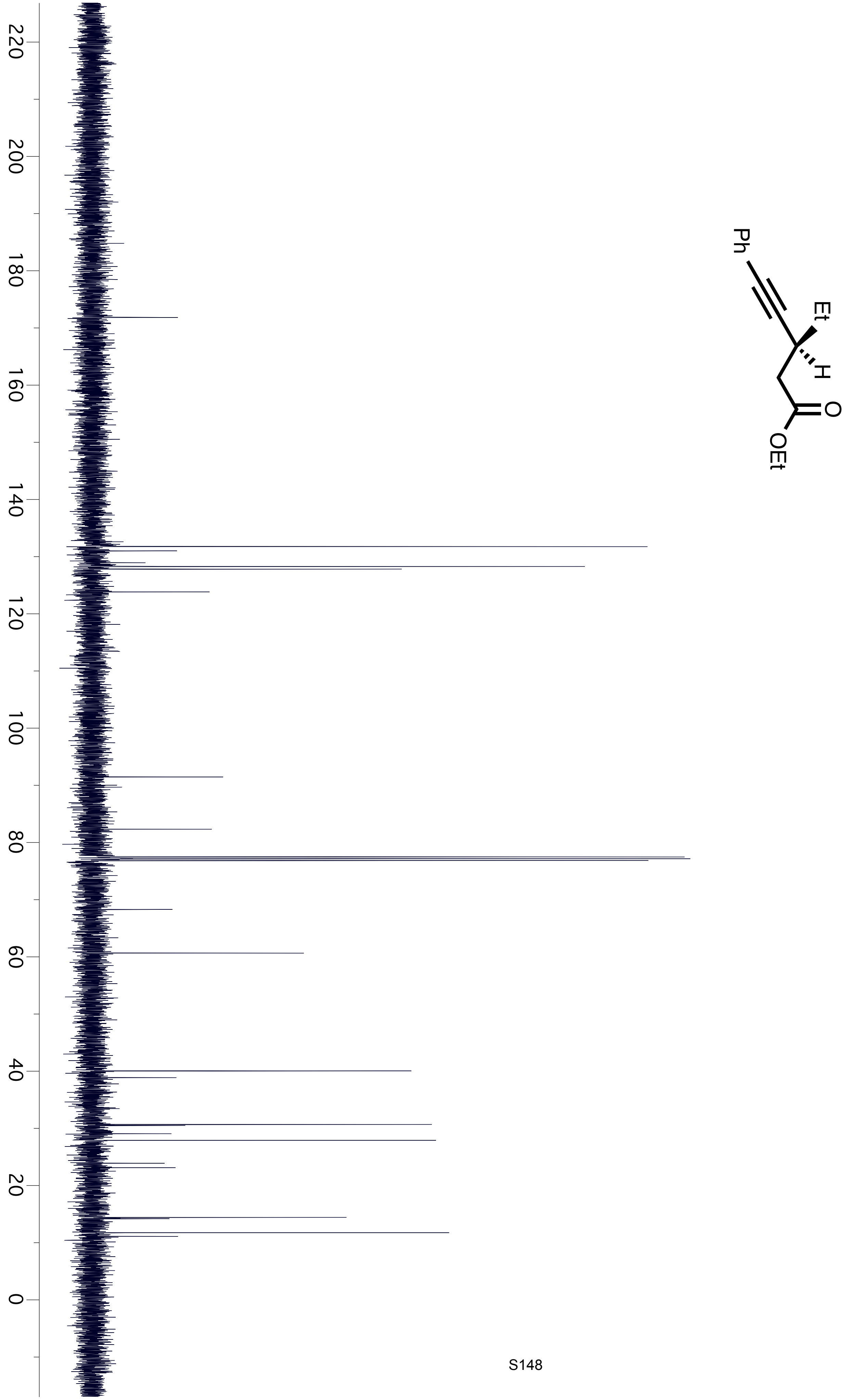
Ph
Et
H
O
OEt
220
200
180
160
140
120
100
80
60
40
20
0

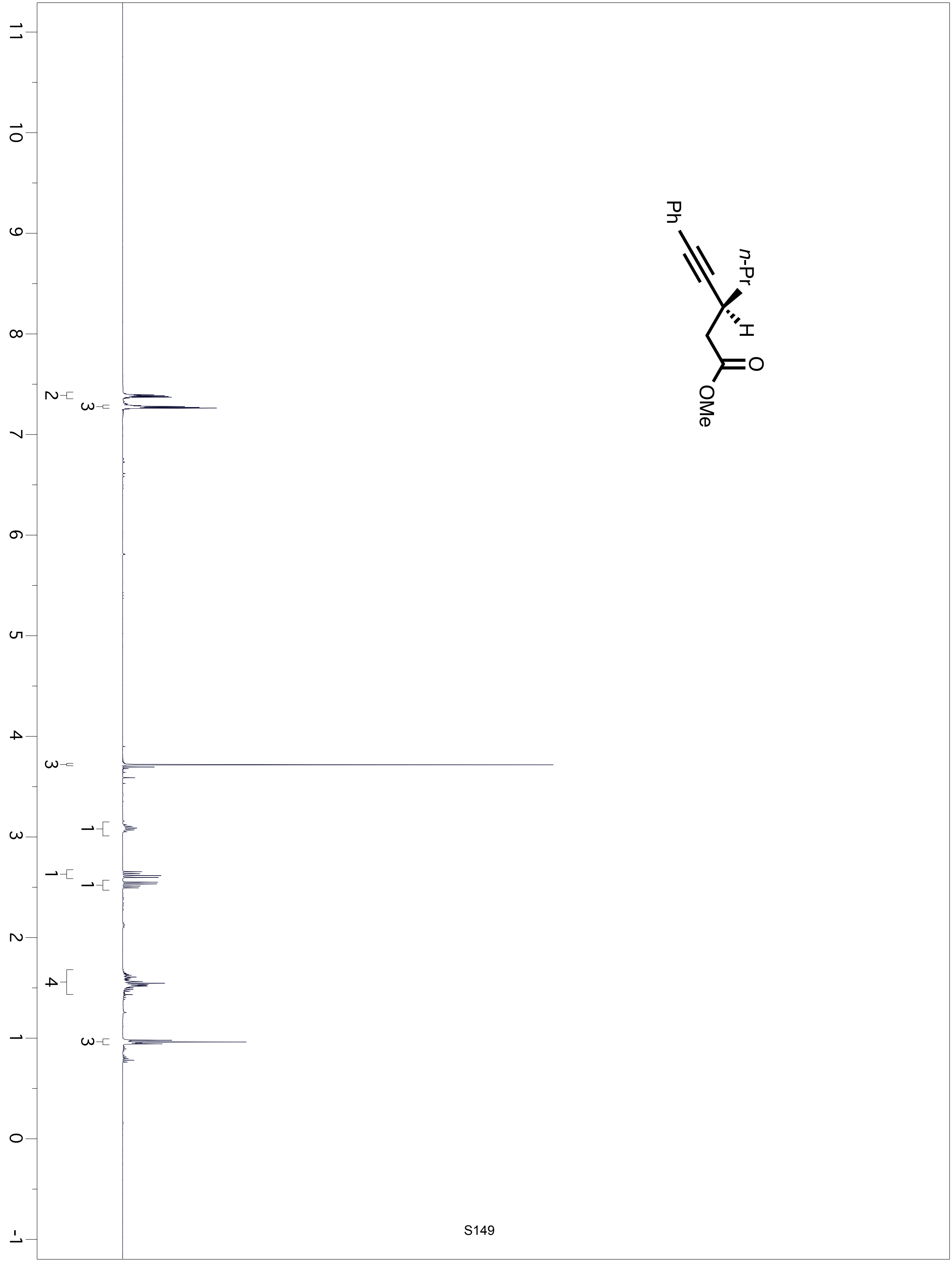

Ph
n-Pr
H
O
OMe
11
10
9
8
7
6
5
4
3
2
1
0
-1

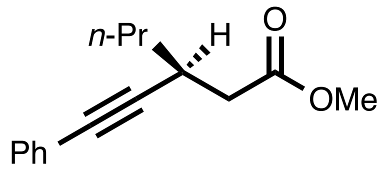

n-Pr
H
O
OMe
Ph

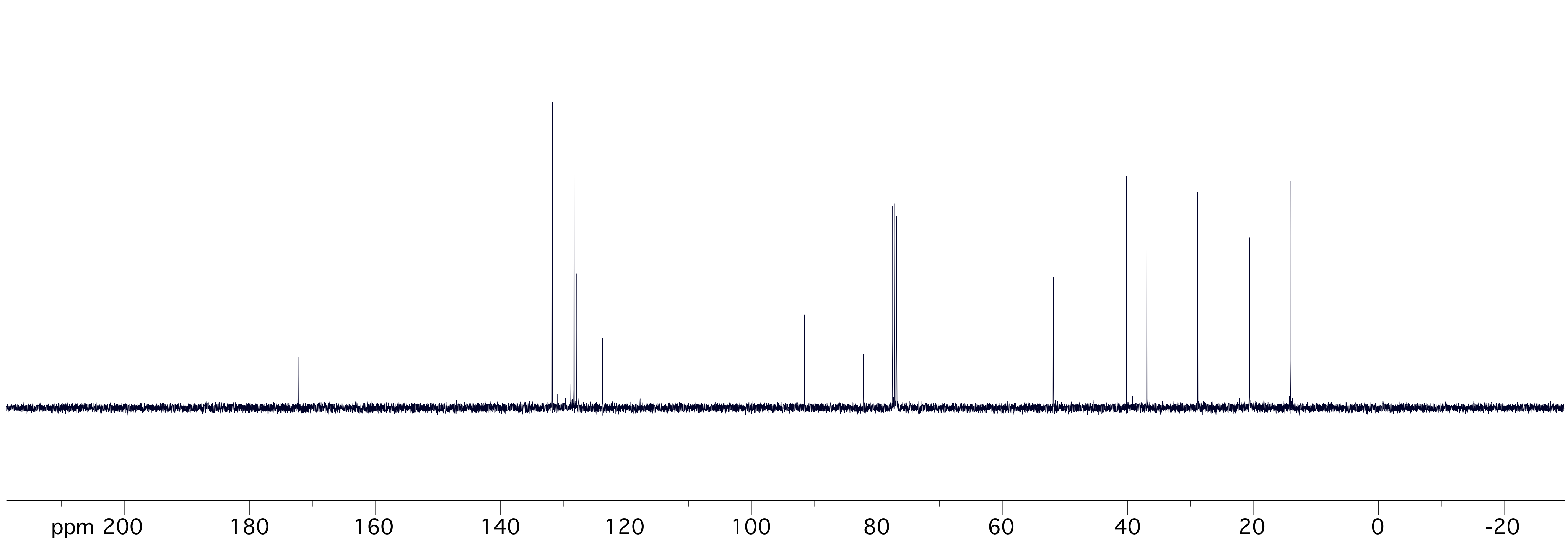

ppm 200
180
160
140
120
100
80
60
40
20
0
-20

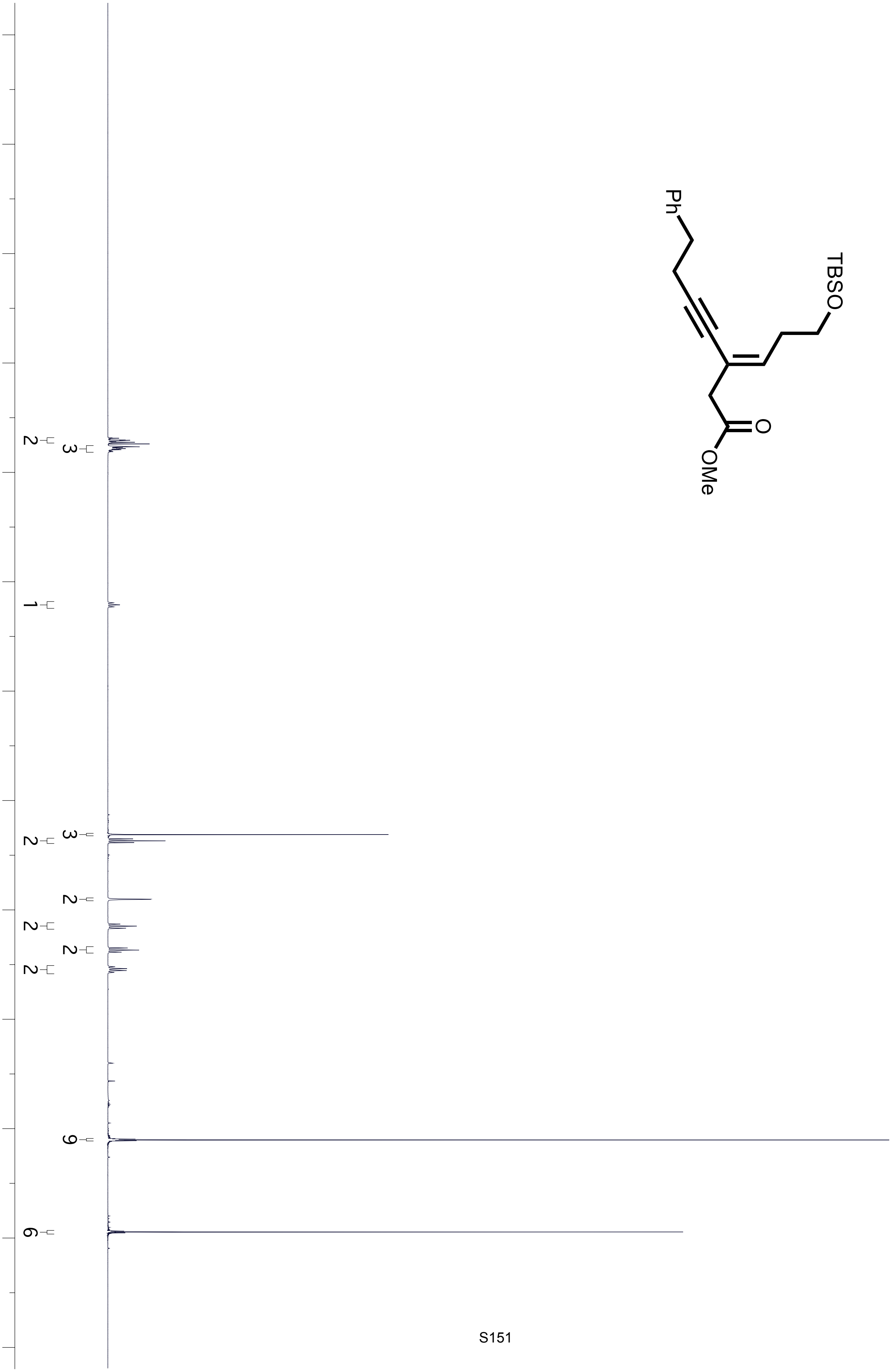
Ph
TBSO
O
OMe

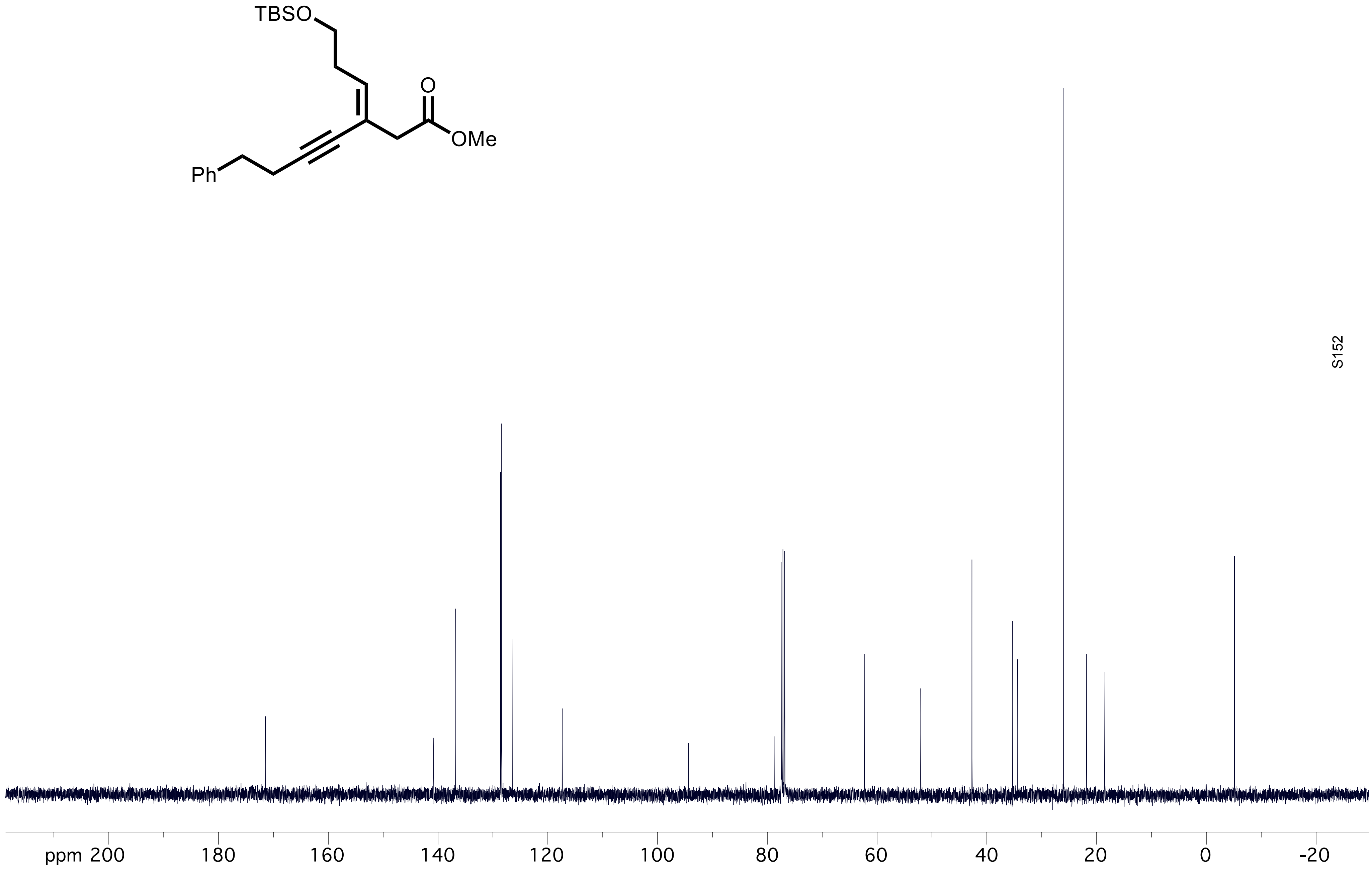

TBSO
O
OMe
Ph
ppm 200
180
160
140
120
100
80
60
40
20
0
-20

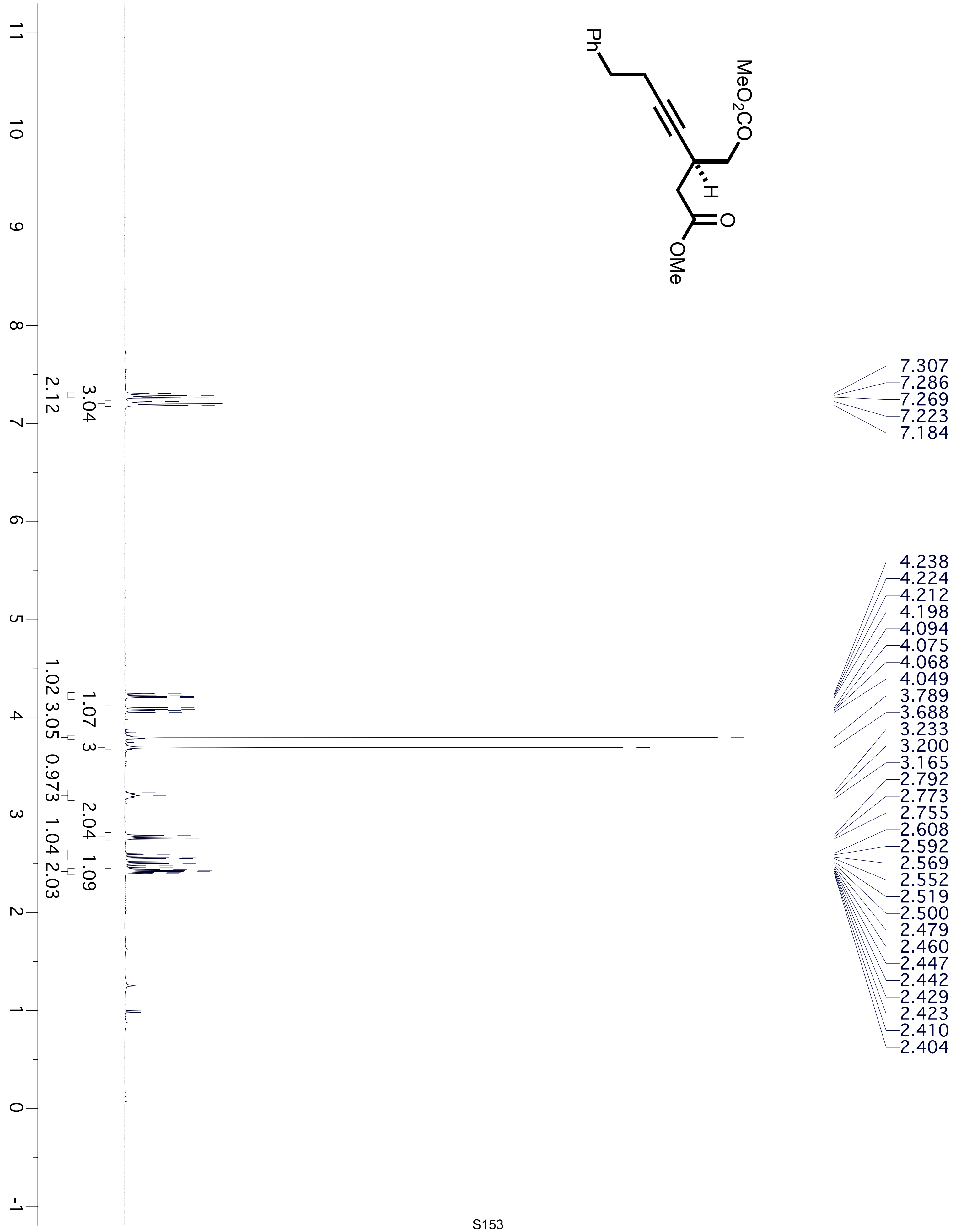

Ph
MeO2CO
H
O
OMe
7.307
7.286
7.269
7.223
7.184
4.238
4.224
4.212
4.198
4.094
4.075
4.068
4.049
3.789
3.688
3.233
3.200
3.165
2.792
2.773
2.755
2.608
2.592
2.569
2.552
2.519
2.500
2.479
2.460
2.447
2.442
2.429
2.423
2.410
2.404
2.12
3.04
1.02
1.07
3.05
3
0.973
2.04
1.04
1.09
2.03
11
10
9
8
7
6
5
4
3
2
1
0
-1

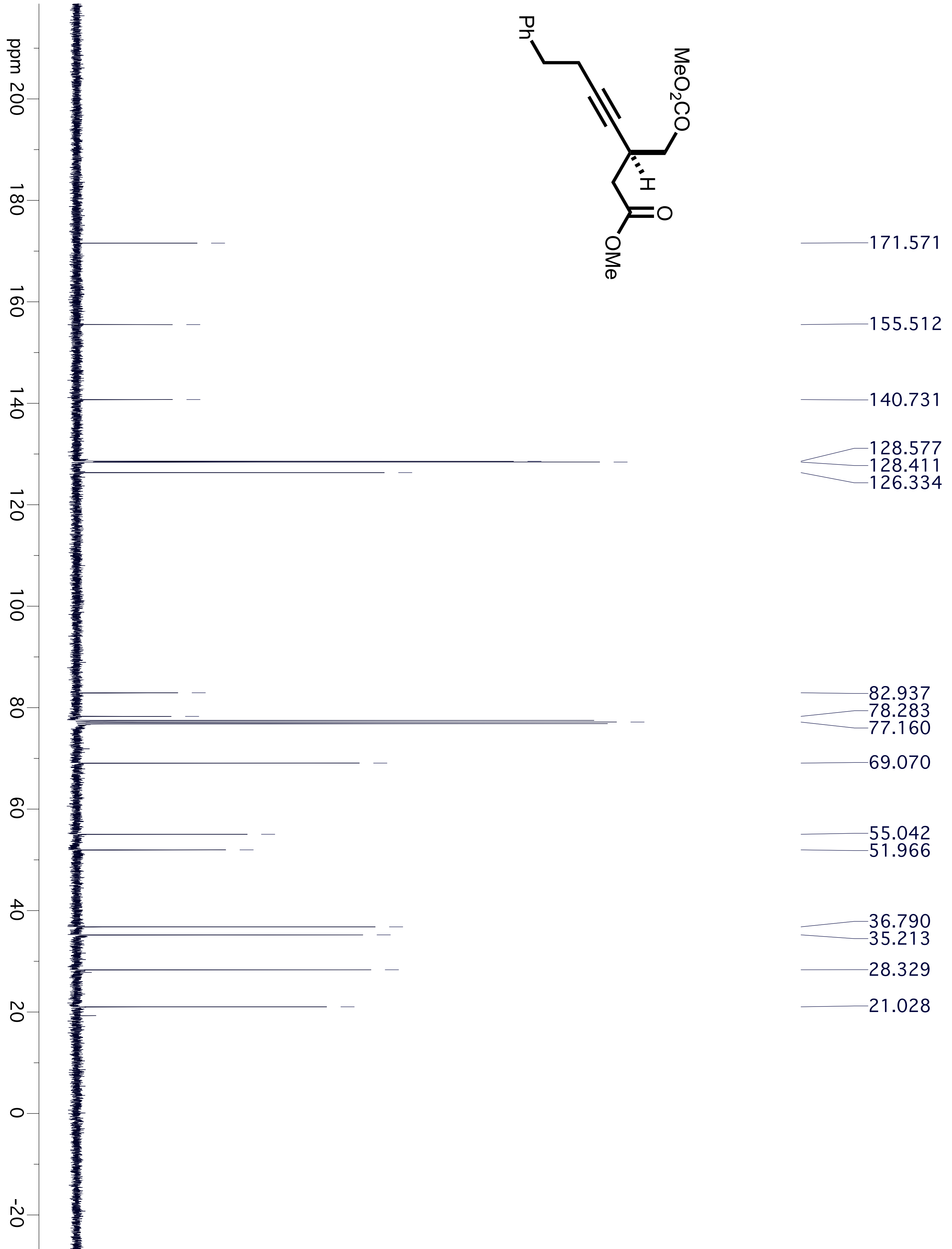
ppm 200
180
160
140
120
100
80
60
40
20
0
-20
Ph
MeO2CO
H
O
OMe
171.571
155.512
140.731
128.577
128.411
126.334
82.937
78.283
77.160
69.070
55.042
51.966
36.790
35.213
28.329
21.028

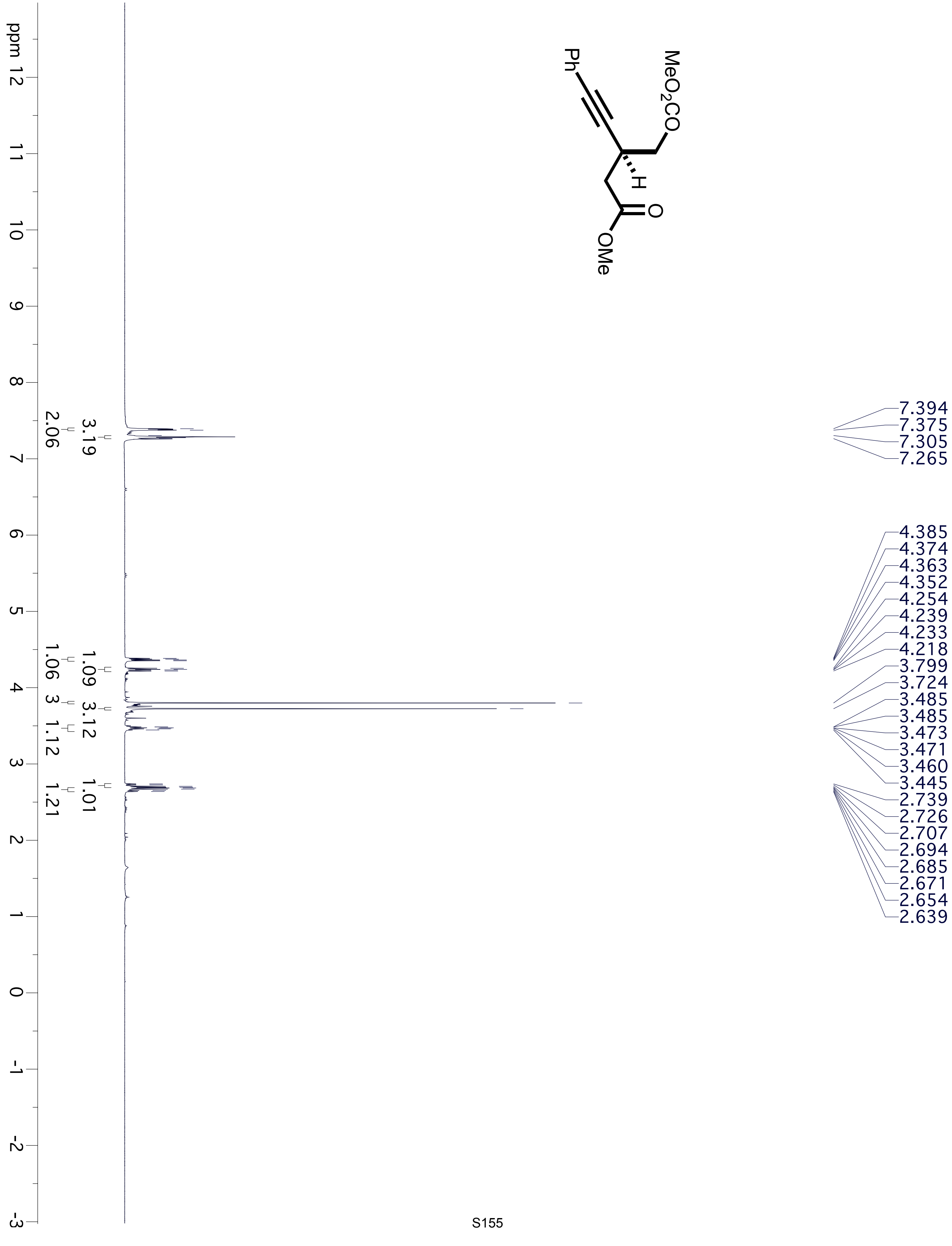

MeO2CO
Ph
H
O
OMe
7.394
7.375
7.305
7.265
4.385
4.374
4.363
4.352
4.254
4.239
4.233
4.218
3.799
3.724
3.485
3.485
3.473
3.471
3.460
3.445
2.739
2.726
2.707
2.694
2.685
2.671
2.654
2.639
2.06
3.19
1.06
1.09
3
3.12
1.12
1.01
1.21
ppm 12
11
10
9
8
7
6
5
4
3
2
1
0
-1
-2
-3

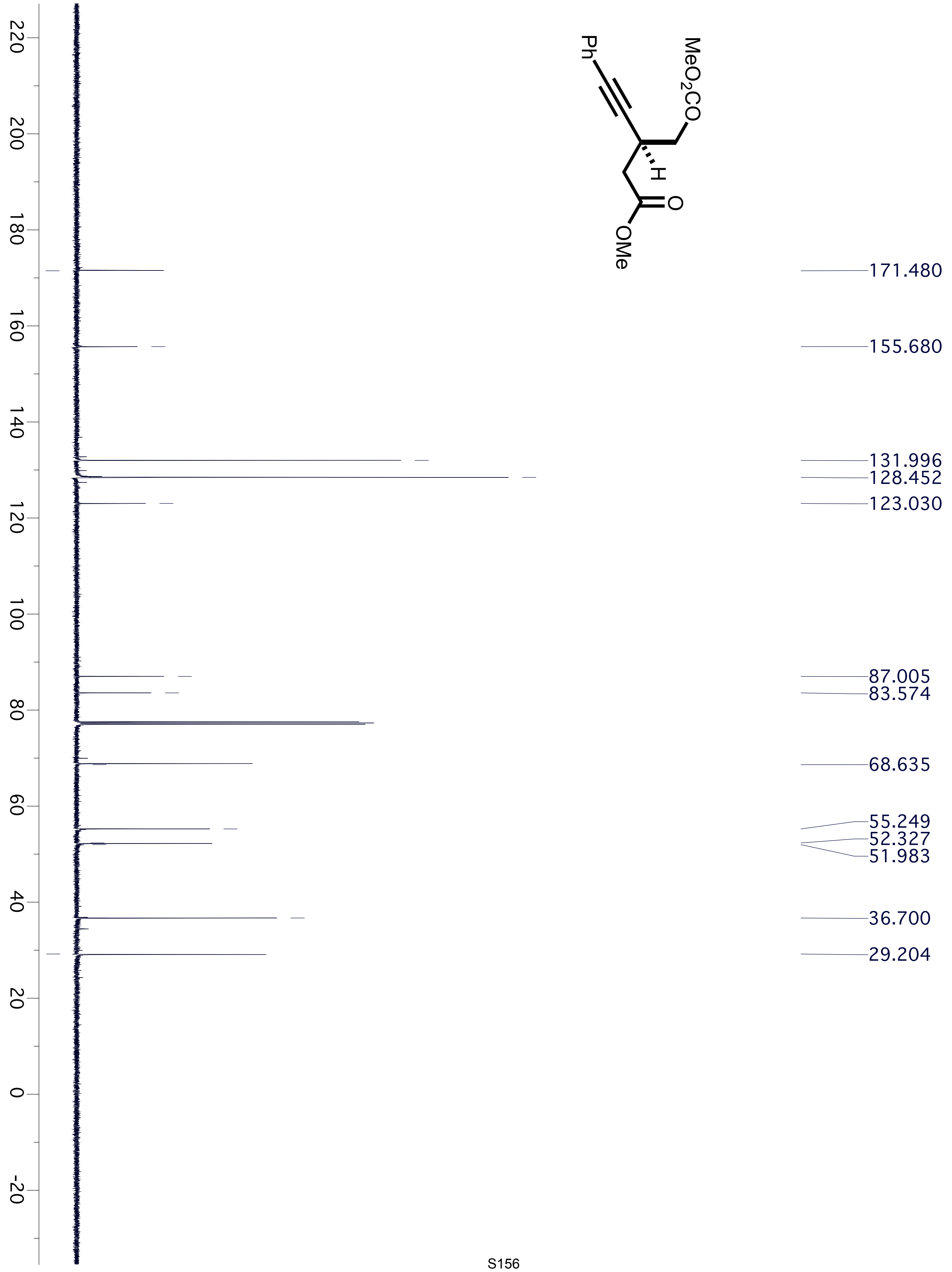
171.480
155.680
131.996
128.452
123.030
87.005
83.574
68.635
55.249
52.327
51.983
36.700
29.204
Ph
MeO2CO
H
O
OMe

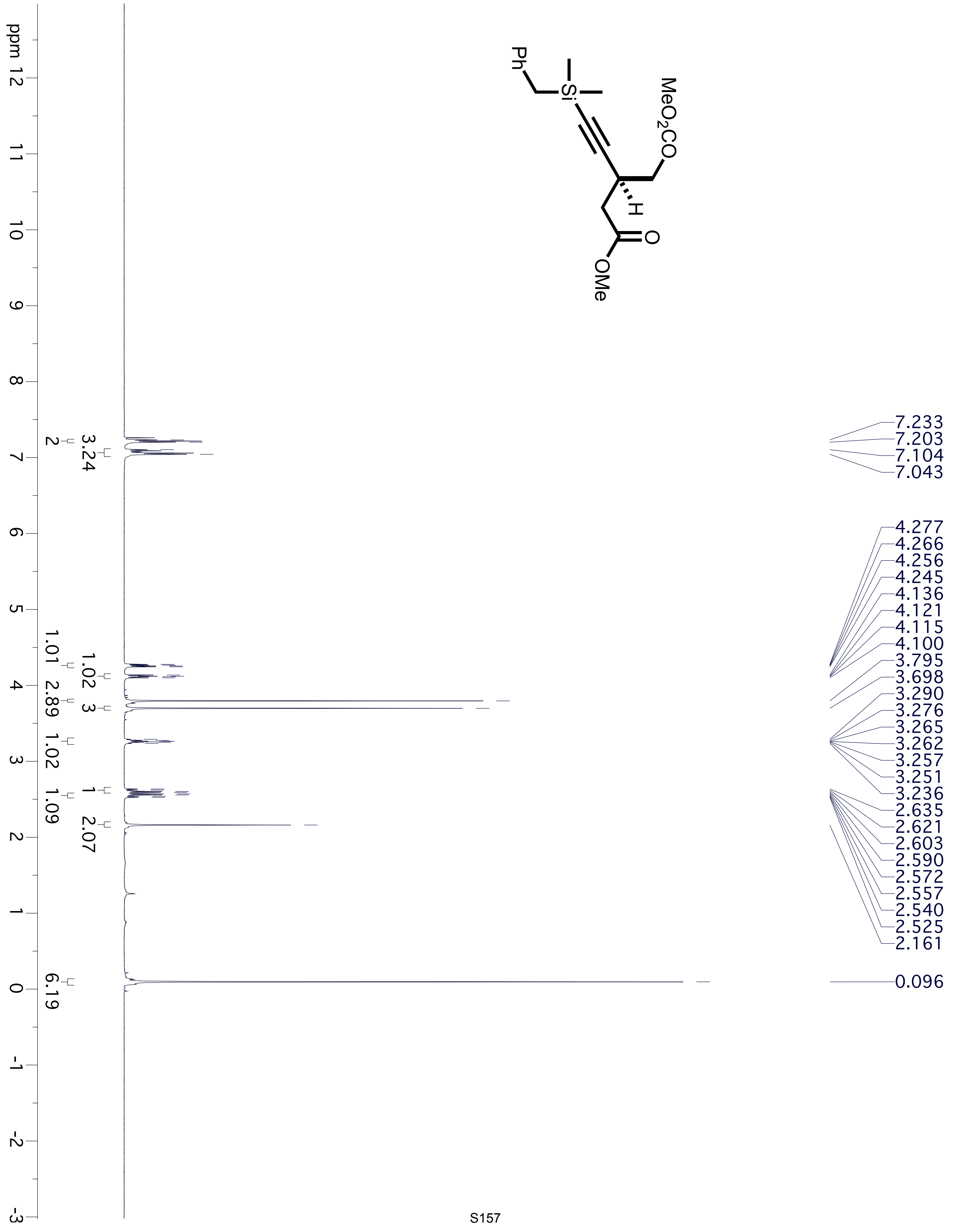

MeO2CO
Si
Ph
H
O
OMe
ppm
7.233
7.203
7.104
7.043
4.277
4.266
4.256
4.245
4.136
4.121
4.115
4.100
3.795
3.698
3.290
3.276
3.265
3.262
3.257
3.251
3.236
2.635
2.621
2.603
2.590
2.572
2.557
2.540
2.525
2.161
0.096

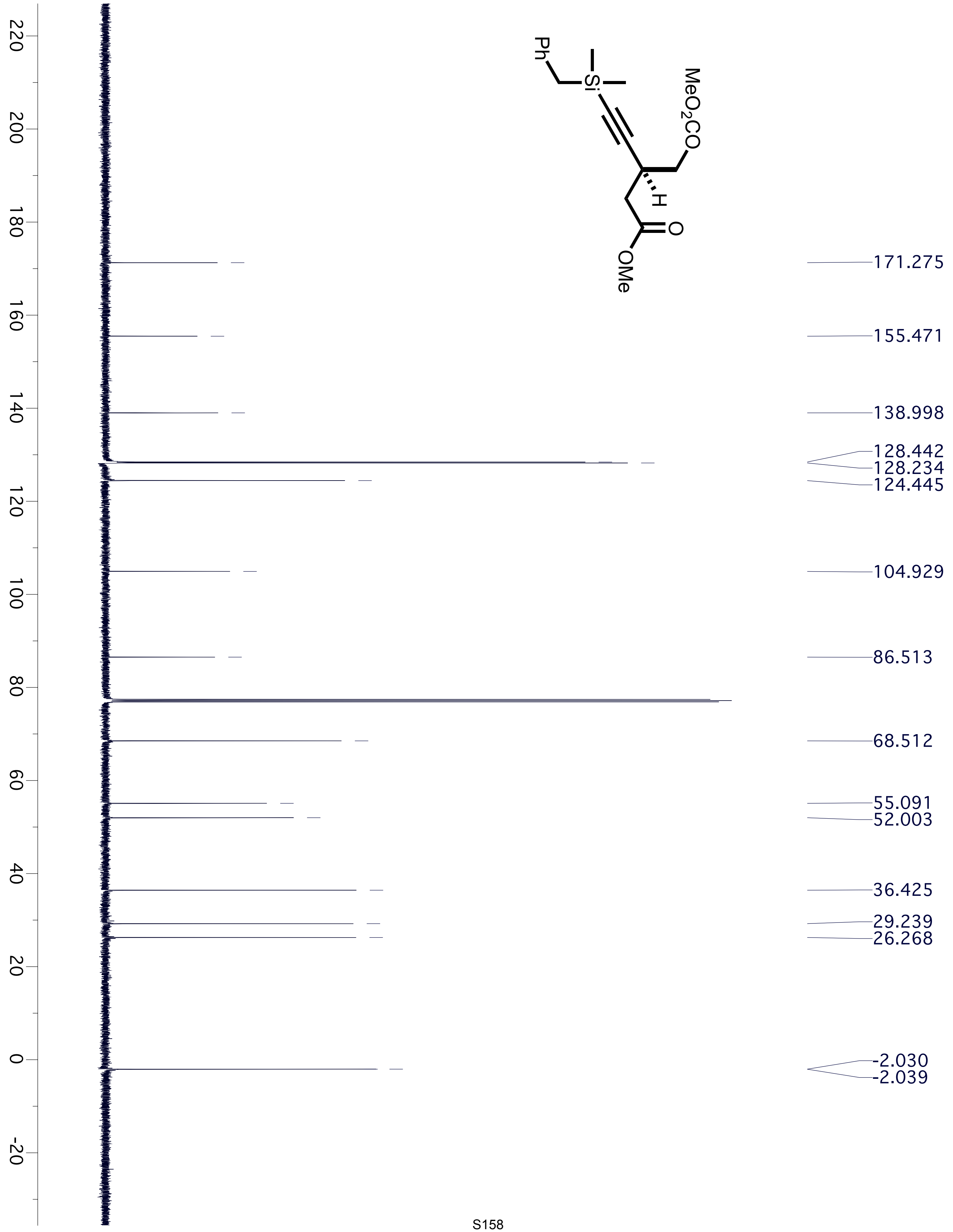
171.275
155.471
138.998
128.442
128.234
124.445
104.929
86.513
68.512
55.091
52.003
36.425
29.239
26.268
-2.030
-2.039
Ph
Si
MeO2CO
H
O
OMe
220
200
180
160
140
120
100
80
60
40
20
0
-20

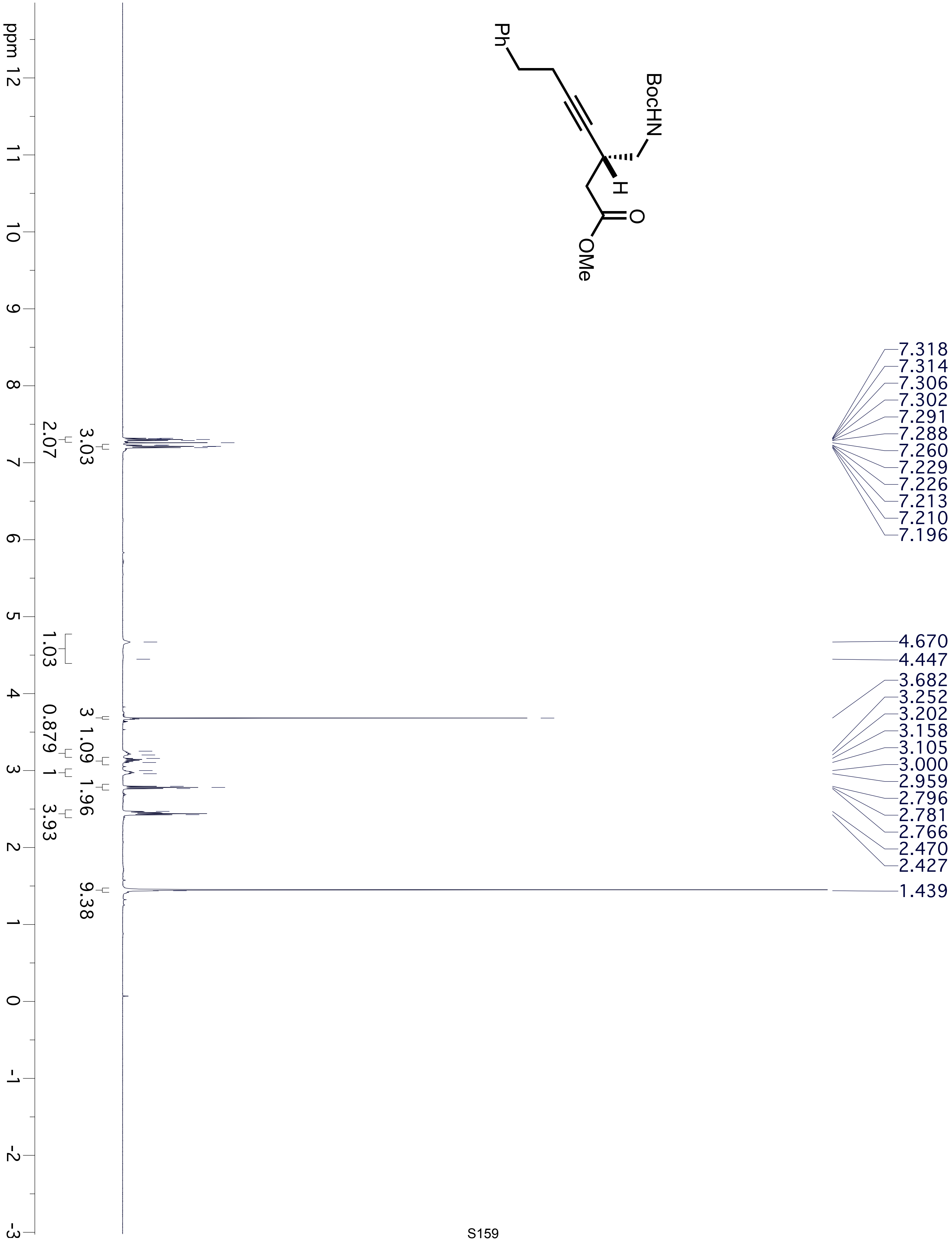
BocHN
H
O
OMe
Ph
7.318
7.314
7.306
7.302
7.291
7.288
7.260
7.229
7.226
7.213
7.210
7.196
4.670
4.447
3.682
3.252
3.202
3.158
3.105
3.000
2.959
2.796
2.781
2.766
2.470
2.427
1.439
2.07
3.03
1.03
3
0.879
1.09
1
1.96
3.93
9.38
ppm 12
11
10
9
8
7
6
5
4
3
2
1
0
-1
-2
-3

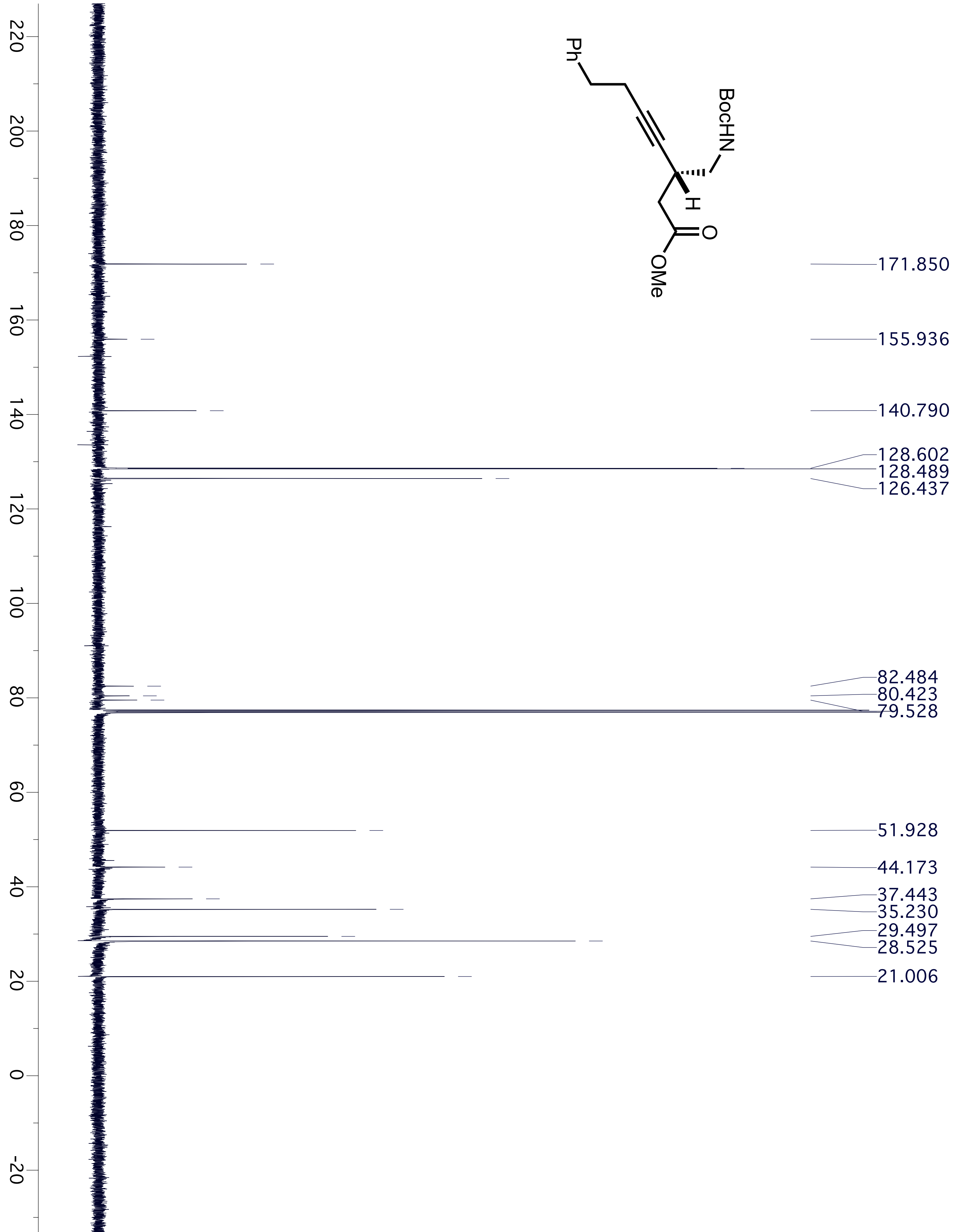

BocHN
Ph
H
O
OMe
171.850
155.936
140.790
128.602
128.489
126.437
82.484
80.423
79.528
51.928
44.173
37.443
35.230
29.497
28.525
21.006
220
200
180
160
140
120
100
80
60
40
20
0
-20

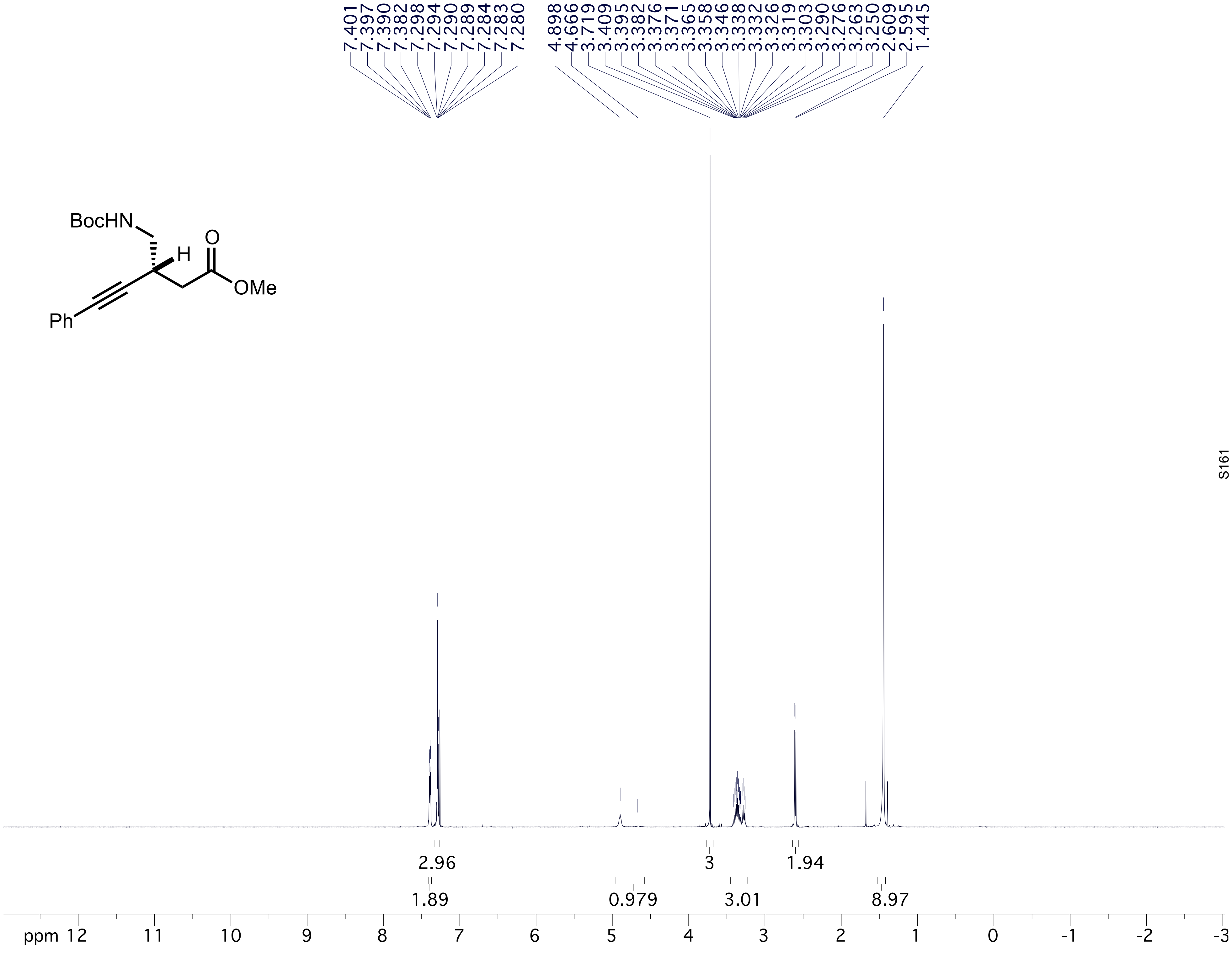
BocHN
H
O
OMe
Ph
7.401
7.397
7.390
7.382
7.298
7.294
7.290
7.289
7.284
7.283
7.280
4.898
4.666
3.719
3.409
3.395
3.382
3.376
3.371
3.365
3.358
3.346
3.338
3.332
3.326
3.319
3.303
3.290
3.276
3.263
3.250
2.609
2.595
1.445
2.96
1.89
0.979
3
3.01
1.94
8.97
ppm 12
11
10
9
8
7
6
5
4
3
2
1
0
-1
-2
-3

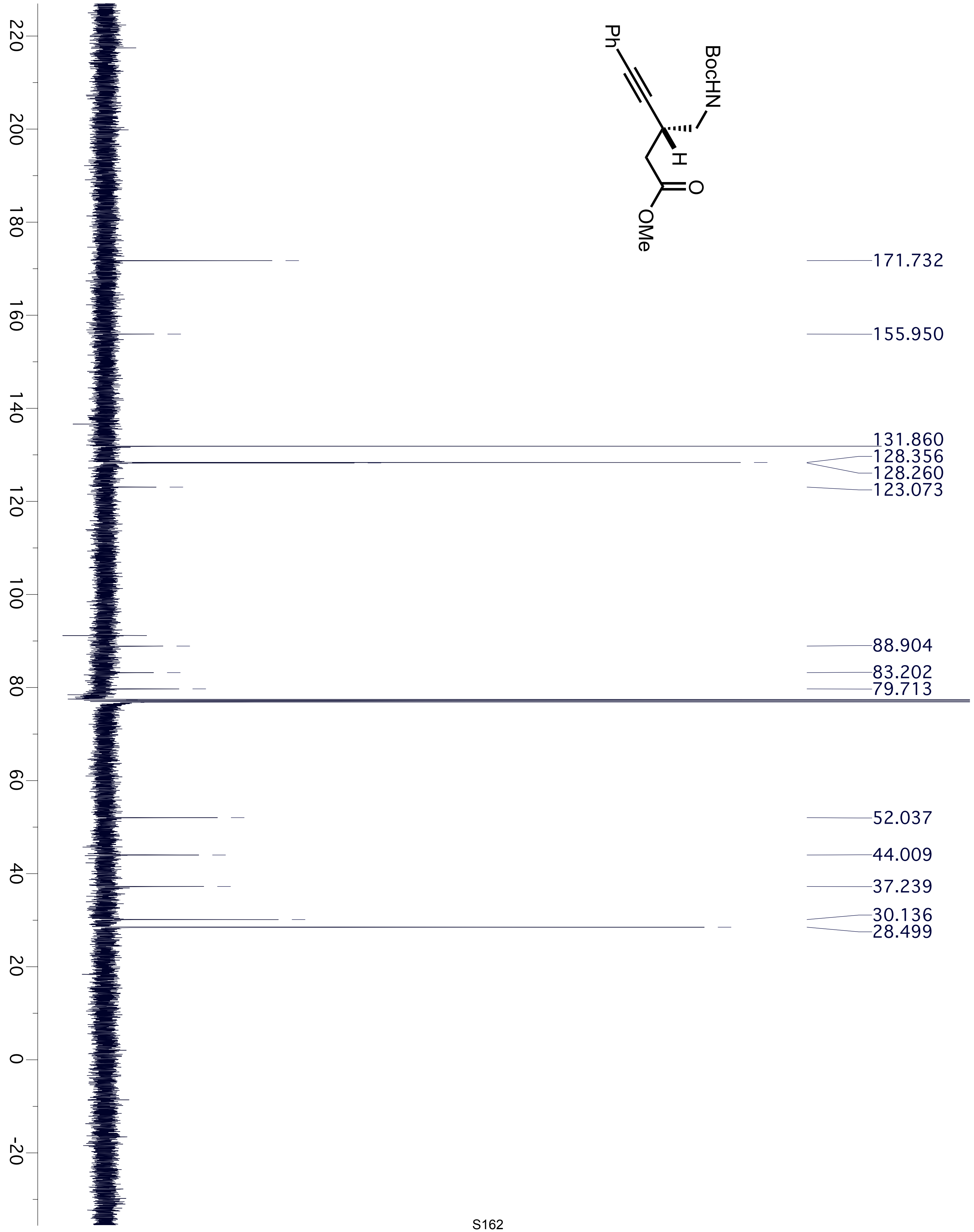
BocHN
Ph
H
O
OMe
171.732
155.950
131.860
128.356
128.260
123.073
88.904
83.202
79.713
52.037
44.009
37.239
30.136
28.499
220
200
180
160
140
120
100
80
60
40
20
0
-20

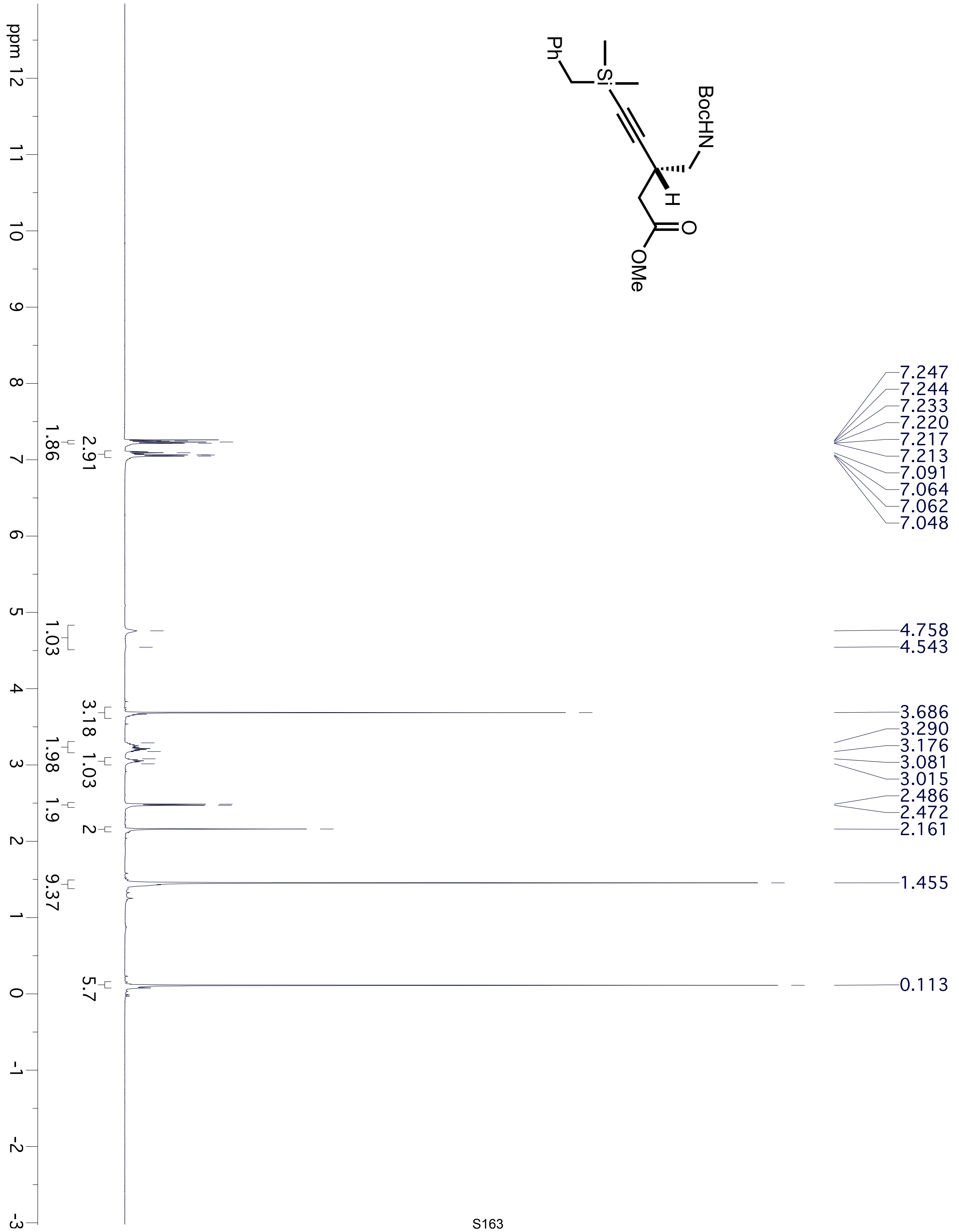
BocHN
H
O
OMe
Si
Ph
ppm 12
11
10
9
8
7
6
5
4
3
2
1
0
-1
-2
-3
1.86
2.91
1.03
3.18
1.98
1.03
1.9
2
9.37
5.7
7.247
7.244
7.233
7.220
7.217
7.213
7.091
7.064
7.062
7.048
4.758
4.543
3.686
3.290
3.176
3.081
3.015
2.486
2.472
2.161
1.455
0.113

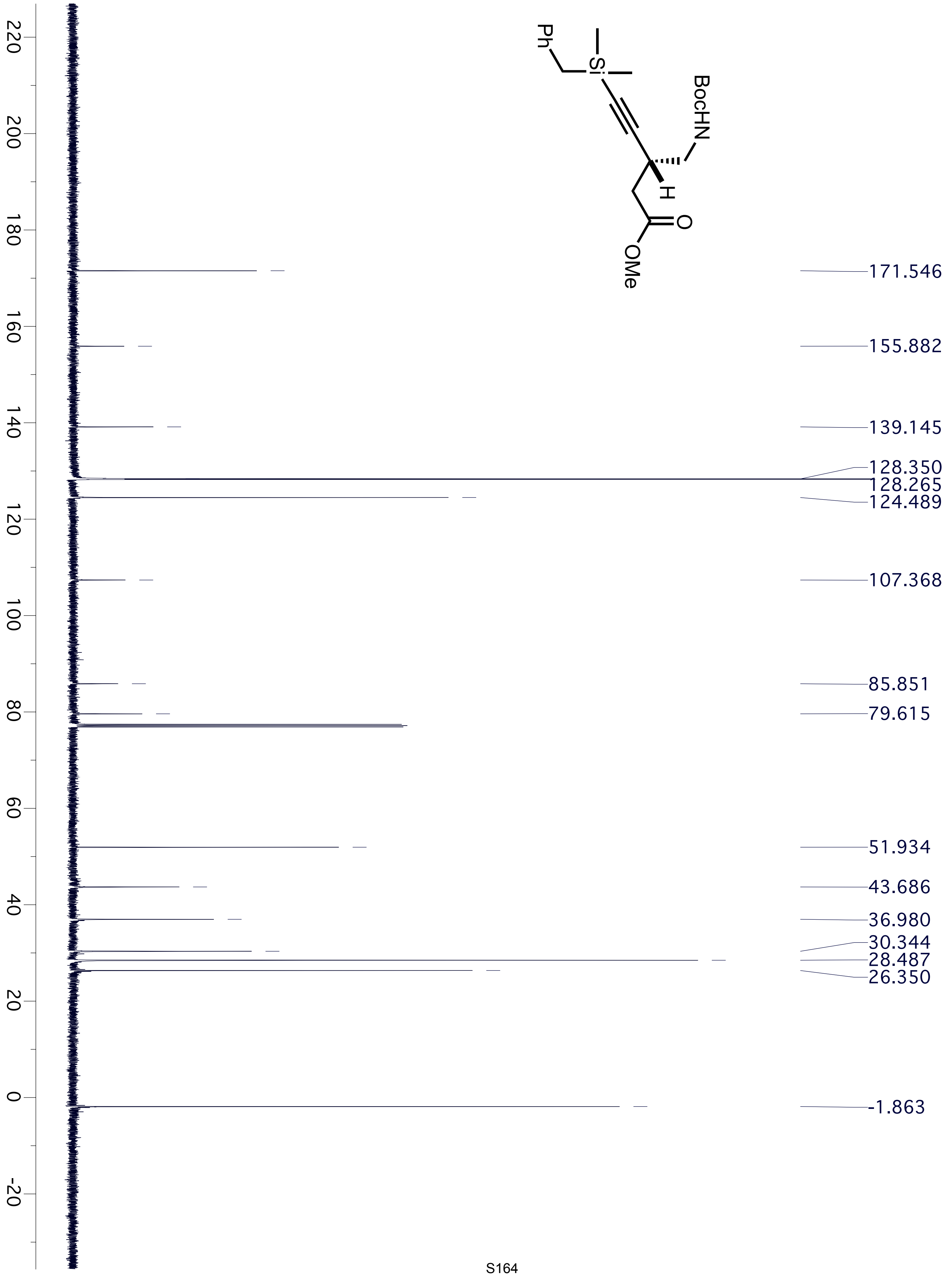
BocHN
Ph
Si
H
O
OMe
171.546
155.882
139.145
128.350
128.265
124.489
107.368
85.851
79.615
51.934
43.686
36.980
30.344
28.487
26.350
-1.863
220
200
180
160
140
120
100
80
60
40
20
0
-20

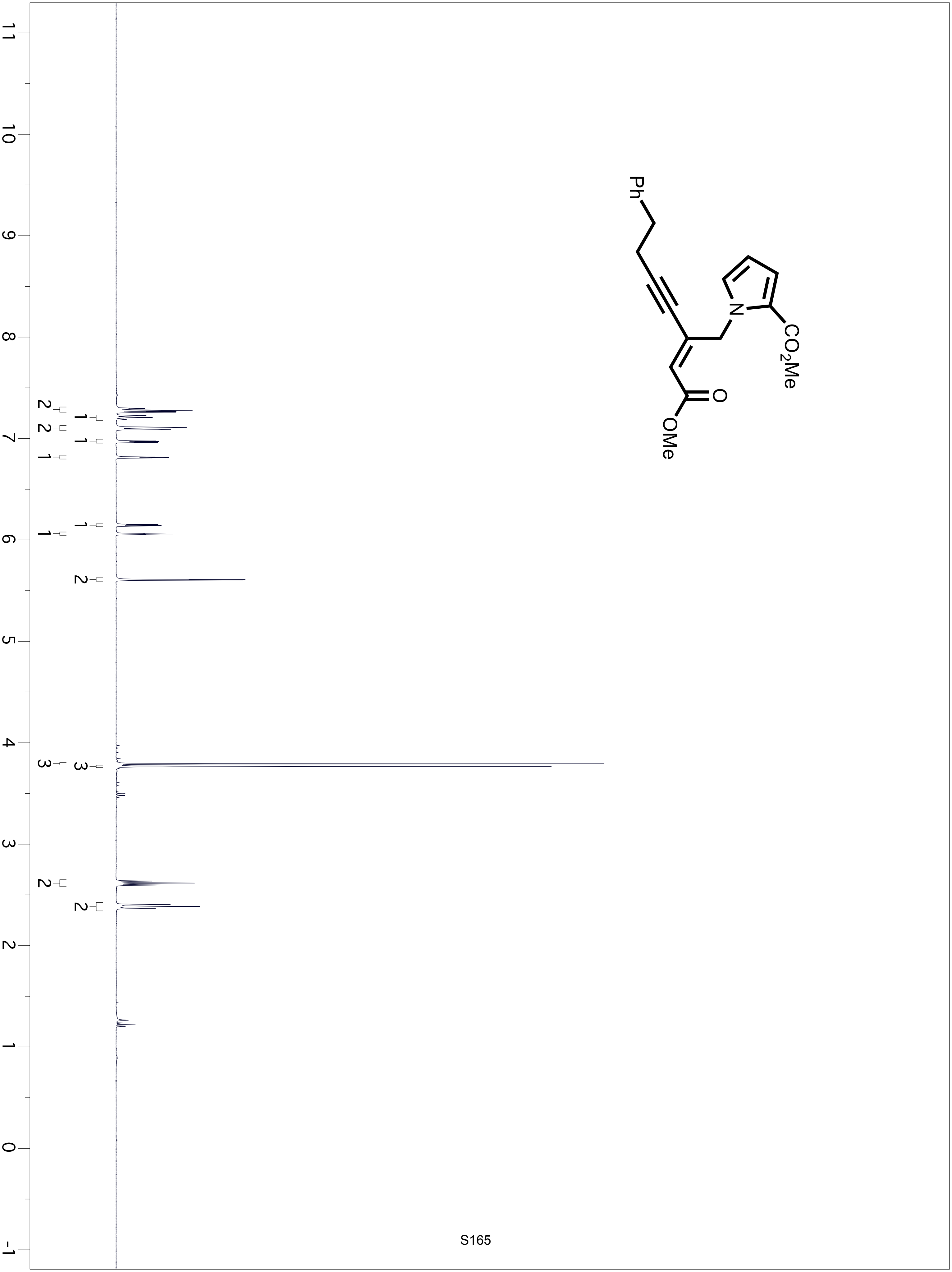

Ph
N
CO2Me
O
OMe

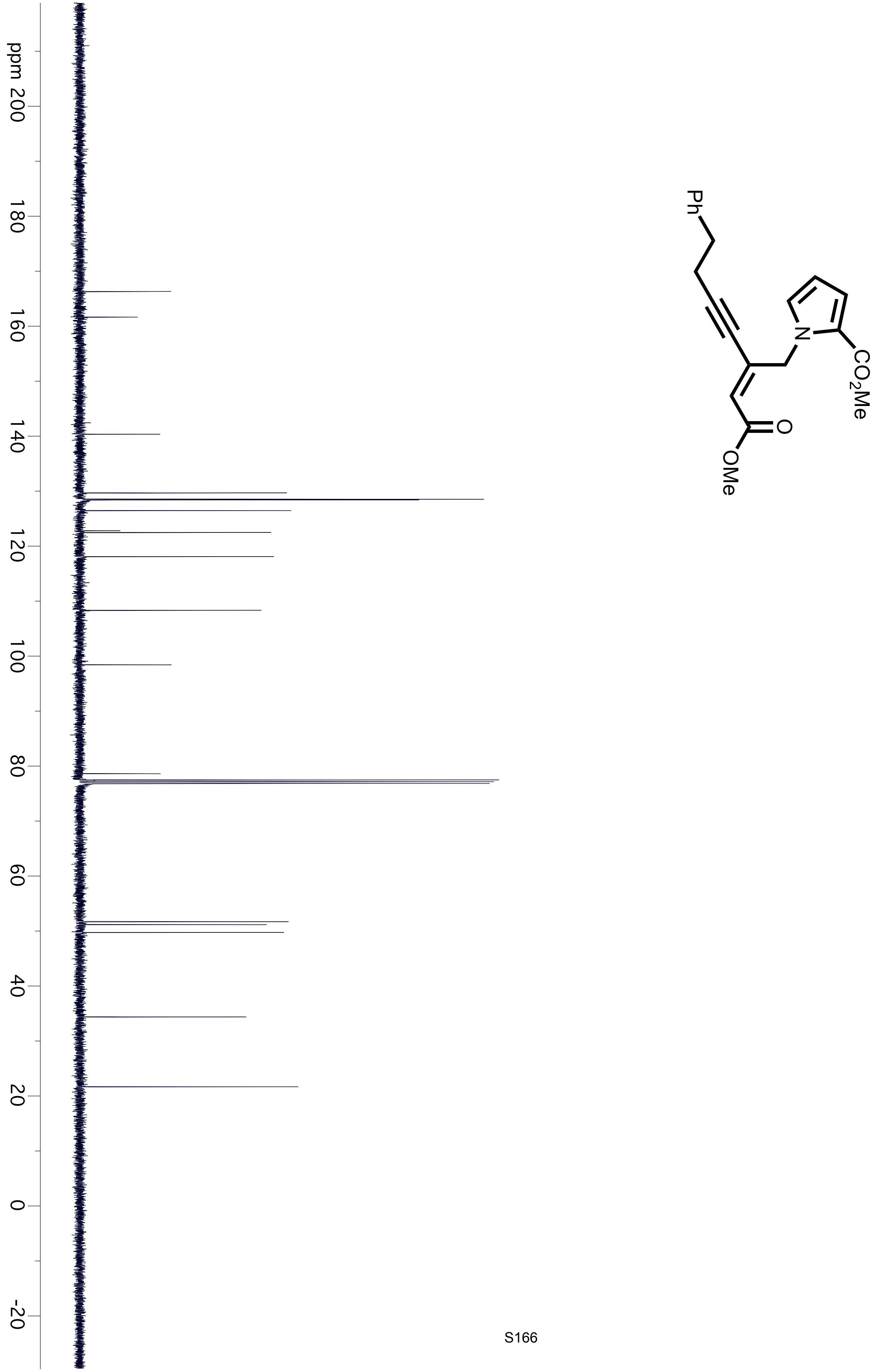
ppm
200
180
160
140
120
100
80
60
40
20
0
-20
Ph
N
CO_2Me
O
OMe

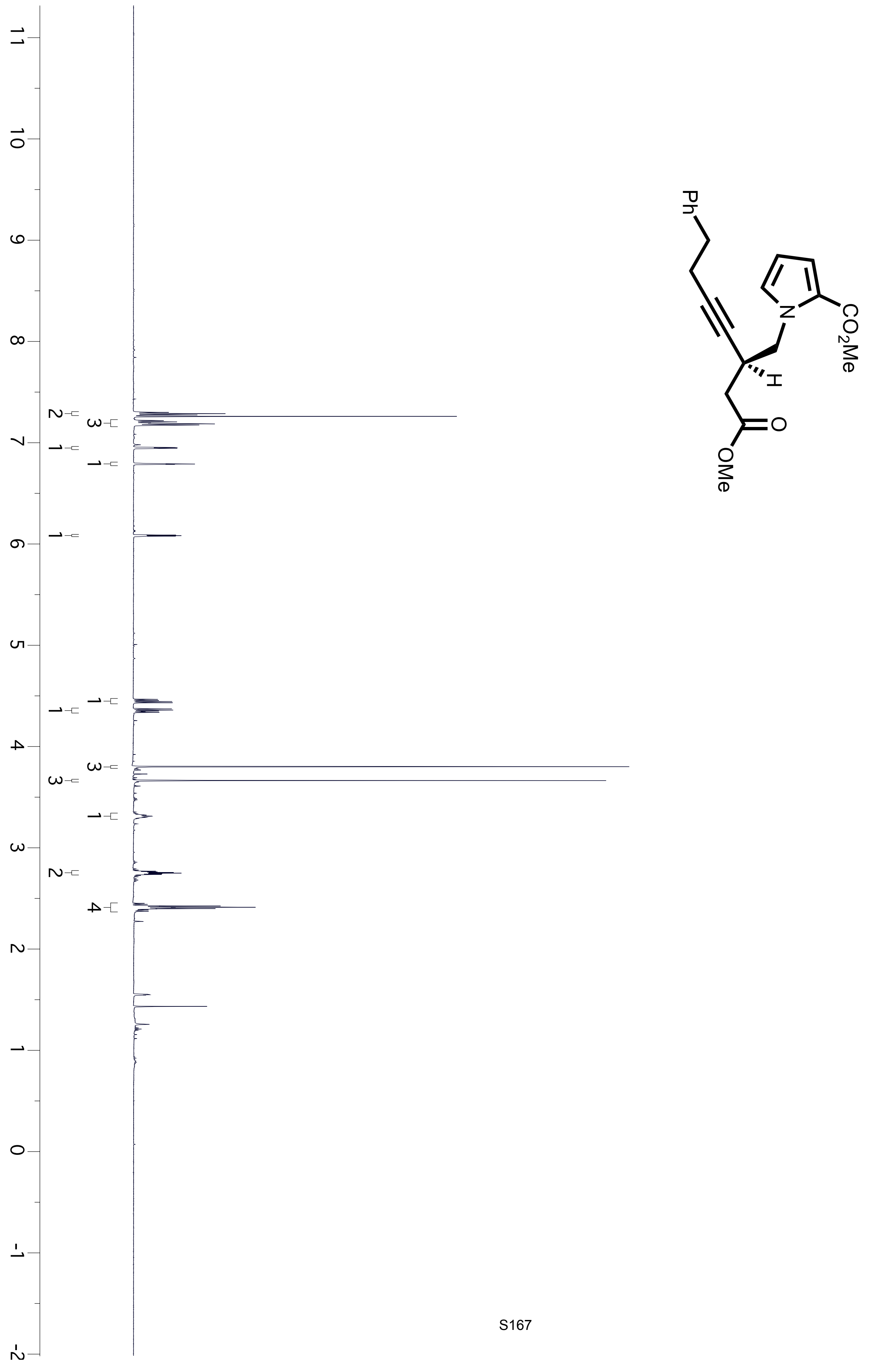
Ph
N
CO_2Me
H
O
OMe
11
10
9
8
7
6
5
4
3
2
1
0
-1
-2

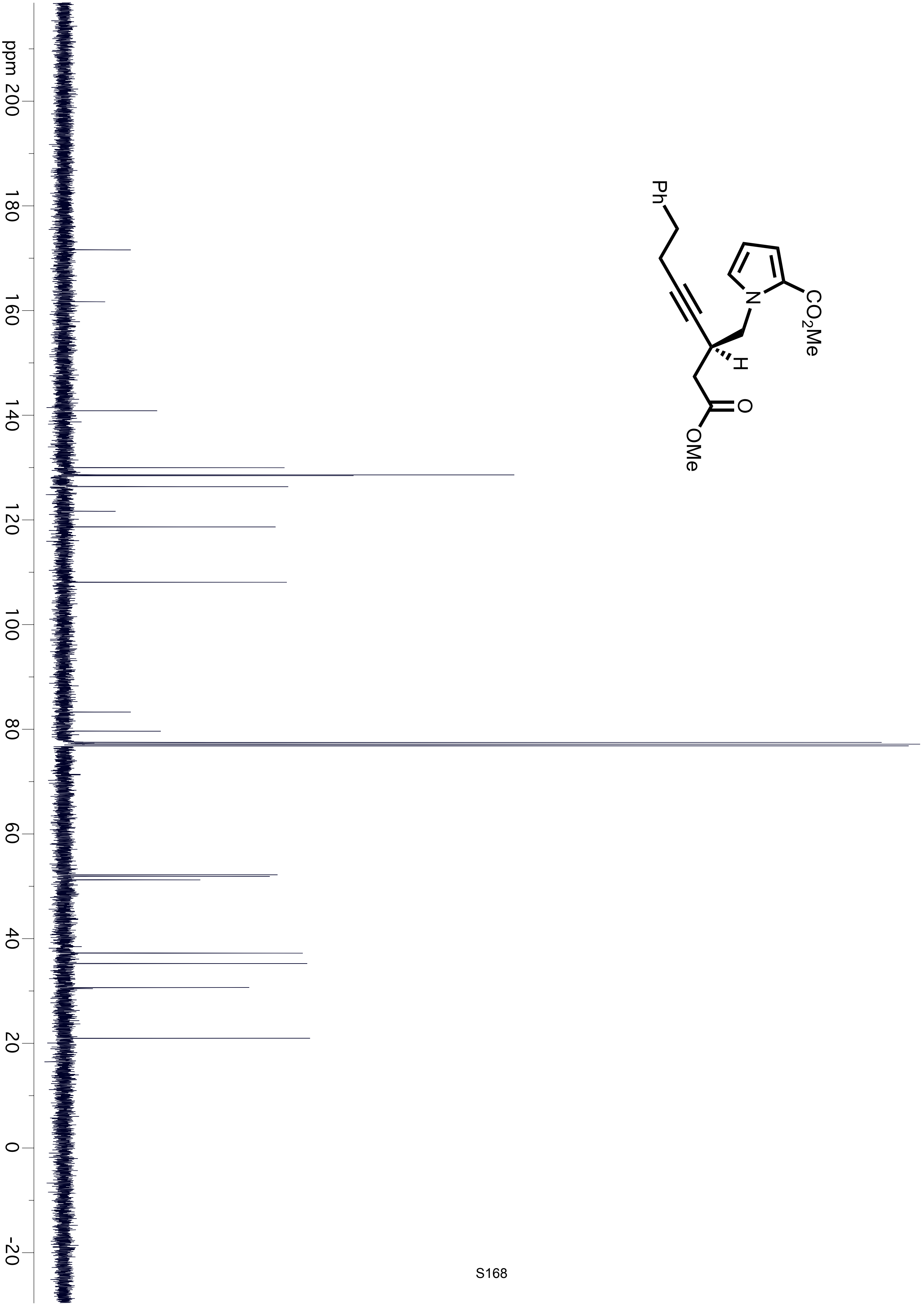

ppm 200
180
160
140
120
100
80
60
40
20
0
-20
Ph
N
CO2Me
H
O
OMe

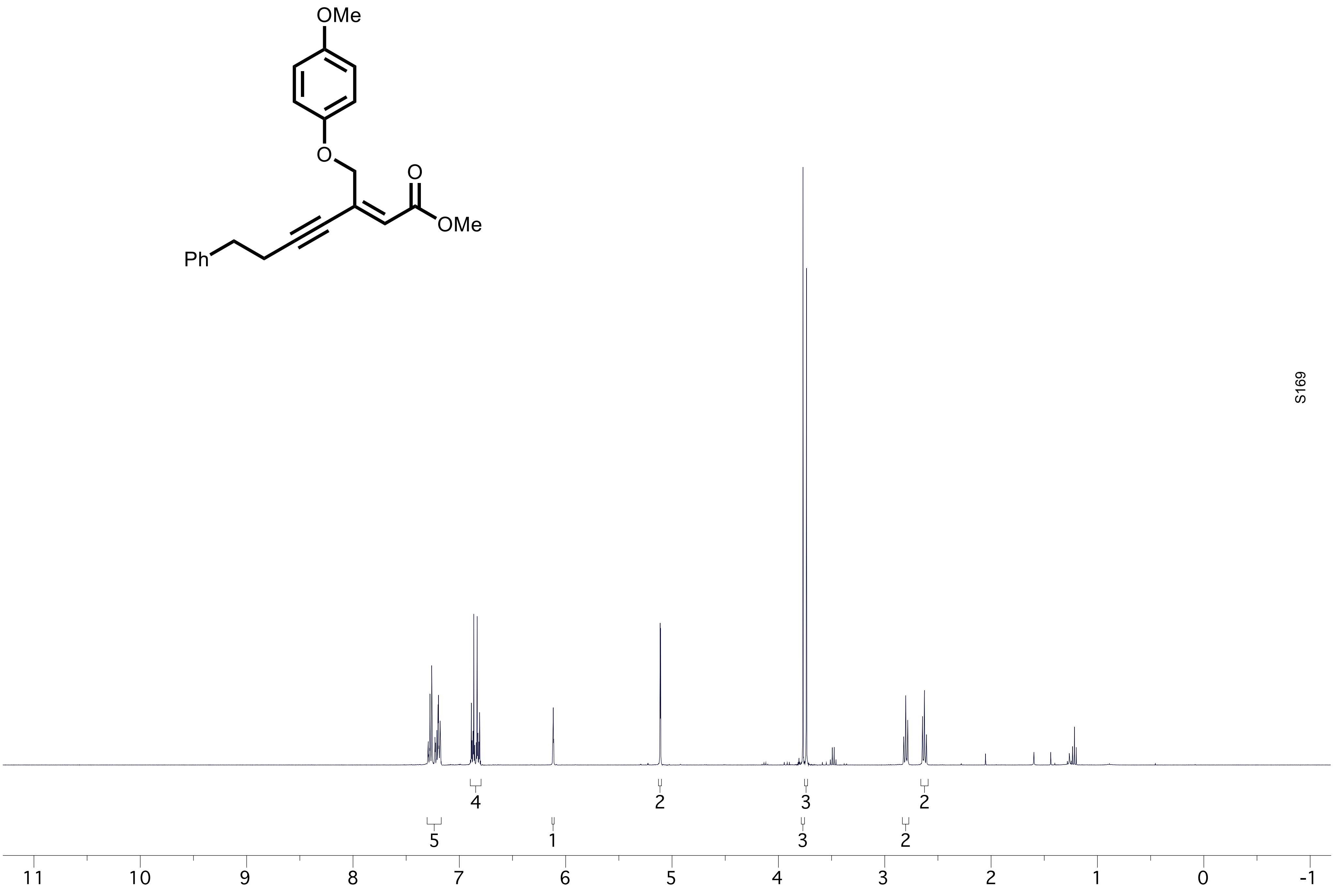

OMe
O
O
OMe
Ph
4
2
3
2
5
1
3
2
11
10
9
8
7
6
5
4
3
2
1
0
-1

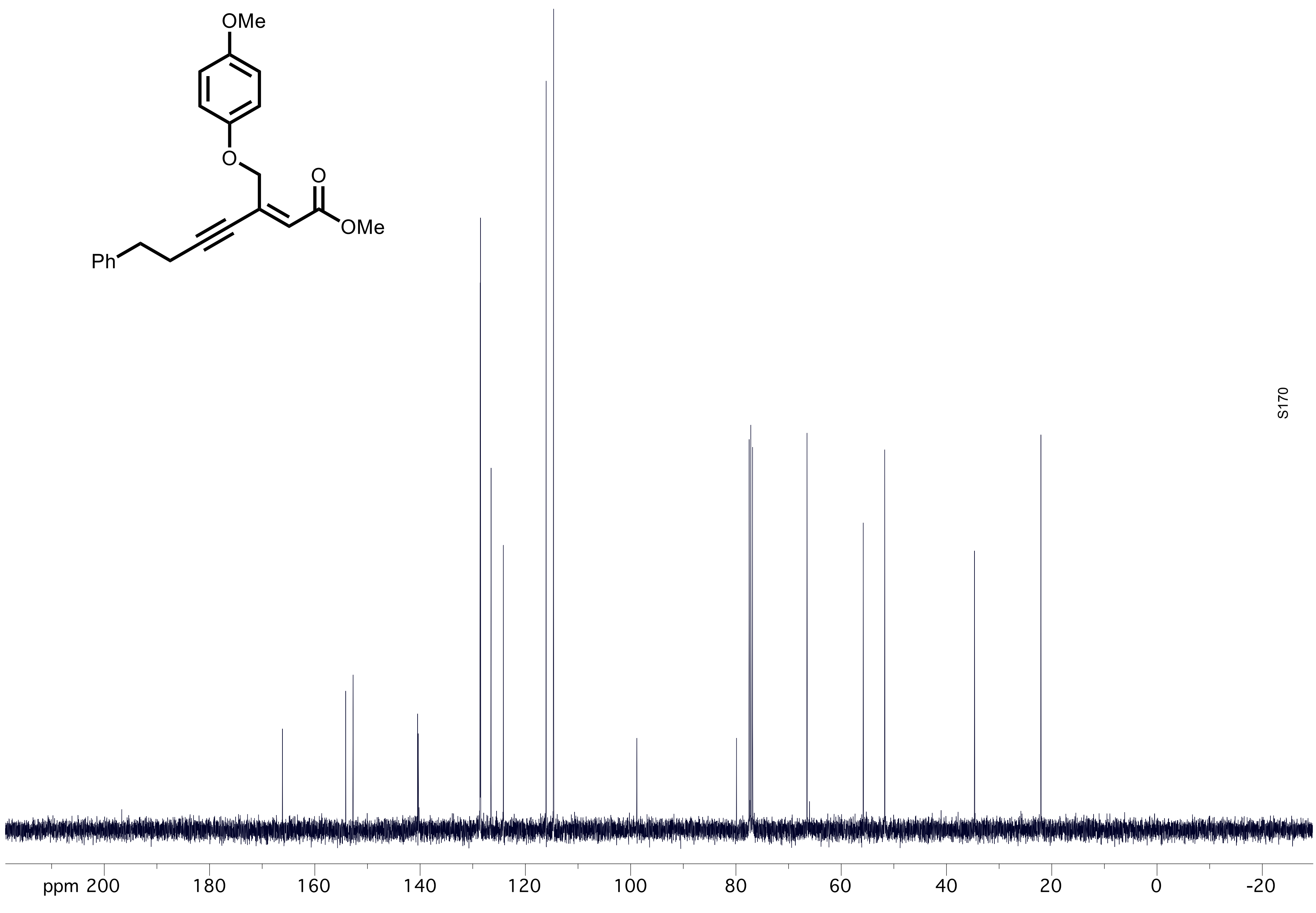
OMe
O
O
OMe
Ph
ppm 200
180
160
140
120
100
80
60
40
20
0
-20

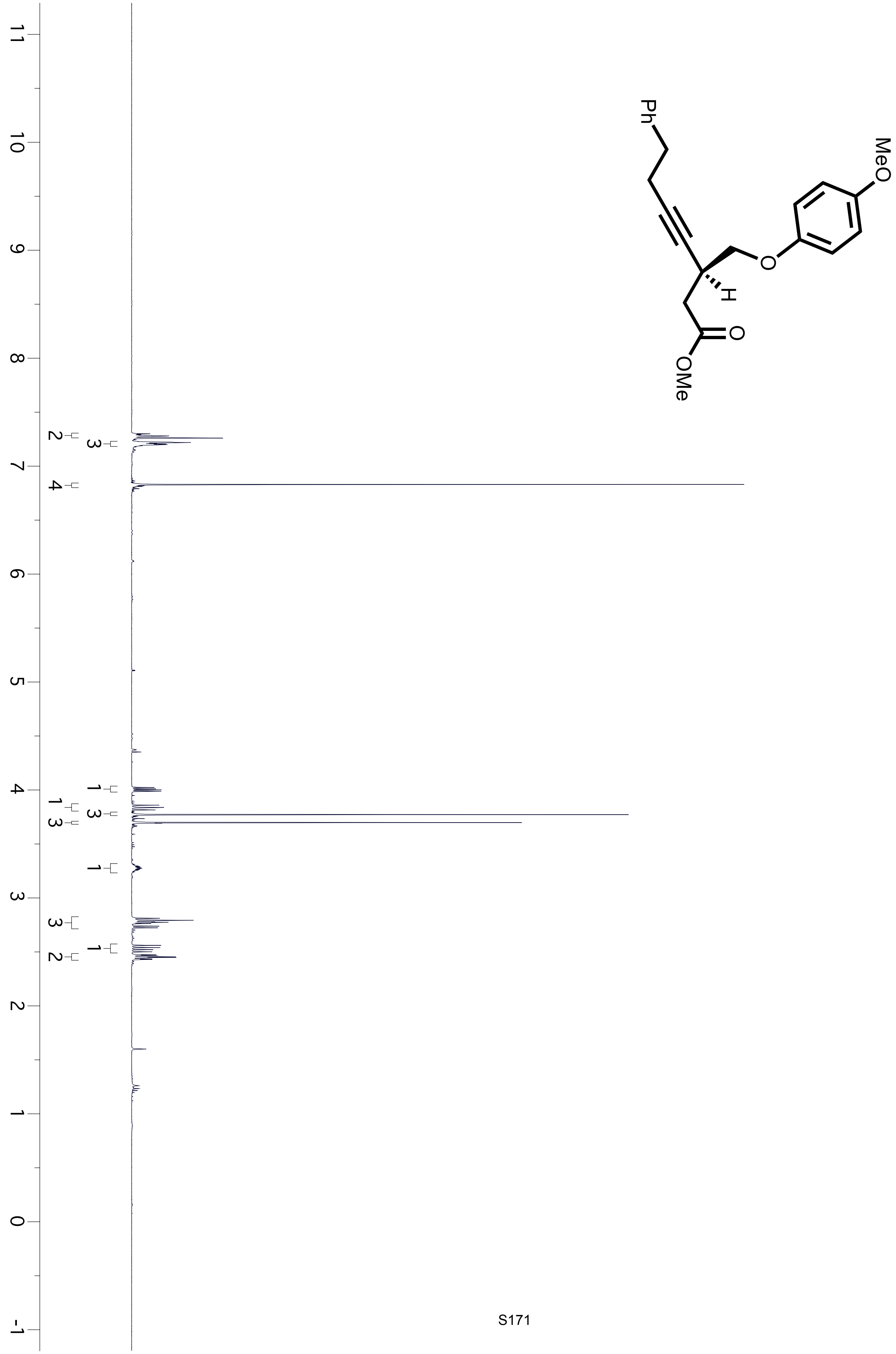

Ph
MeO
O
H
O
OMe
11
10
9
8
7
6
5
4
3
2
1
0
-1

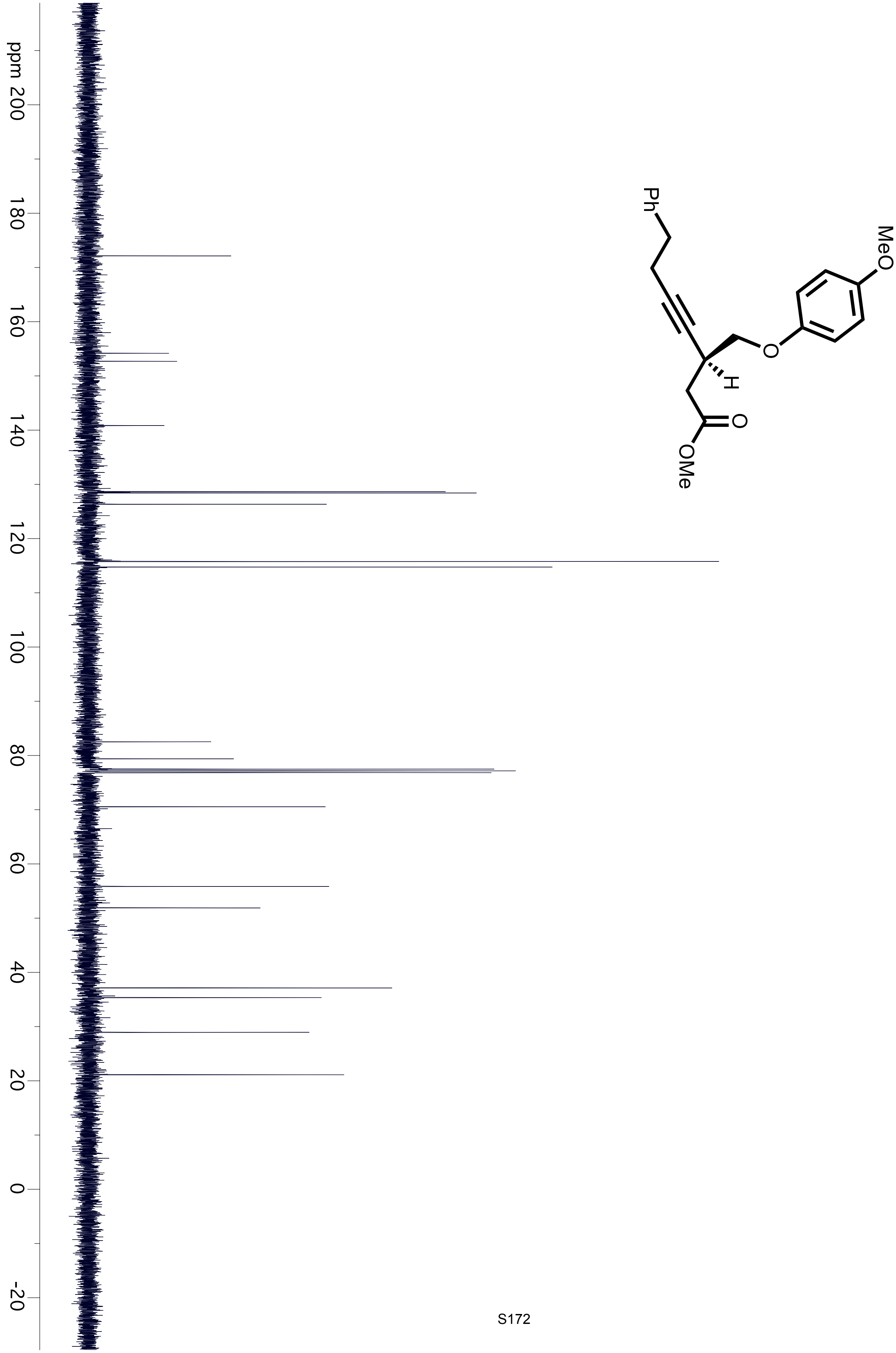
ppm 200
180
160
140
120
100
80
60
40
20
0
-20
Ph
MeO
O
H
O
OMe

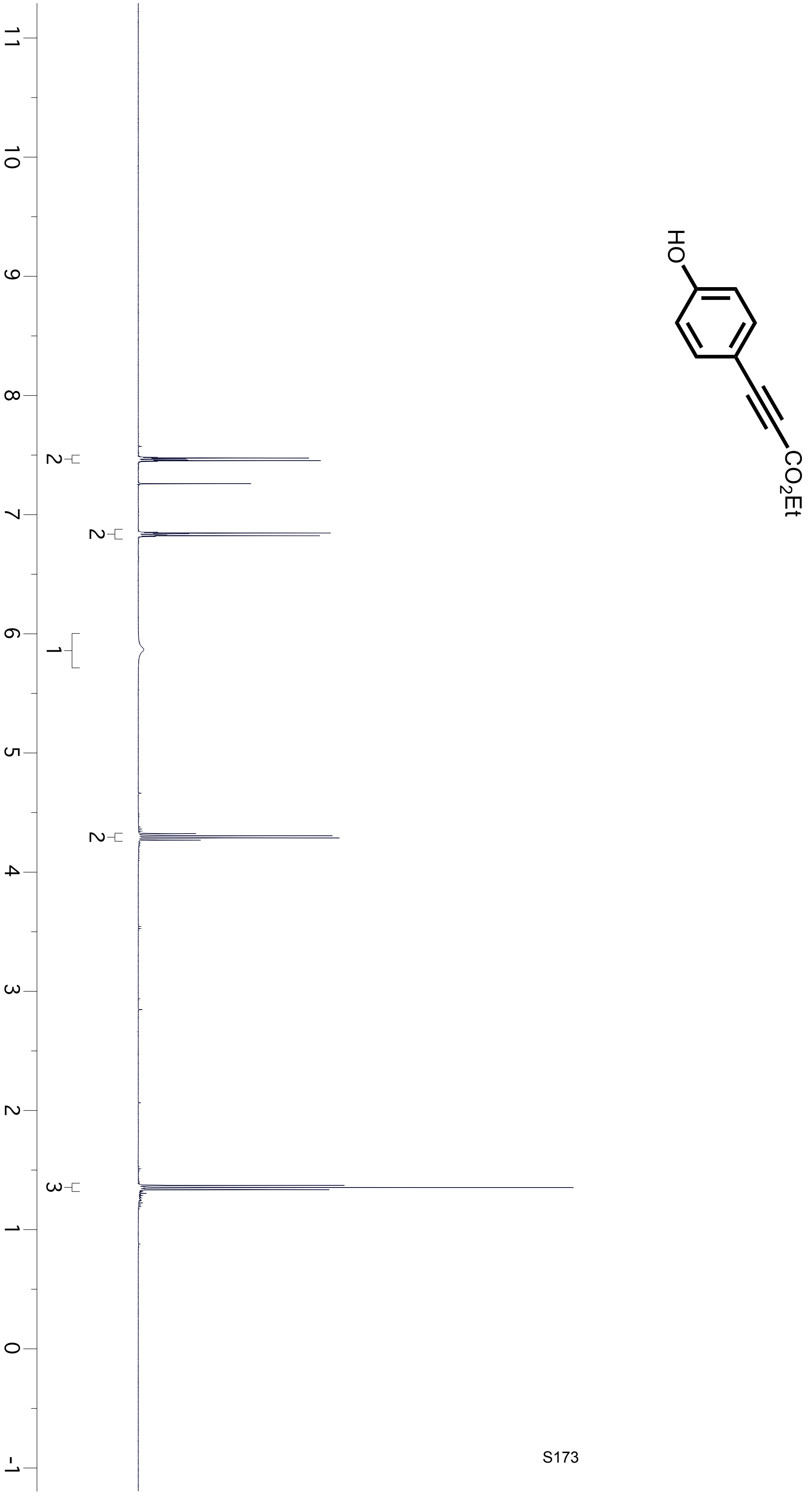
HO
CO2Et
11
10
9
8
7
6
5
4
3
2
1
0
-1

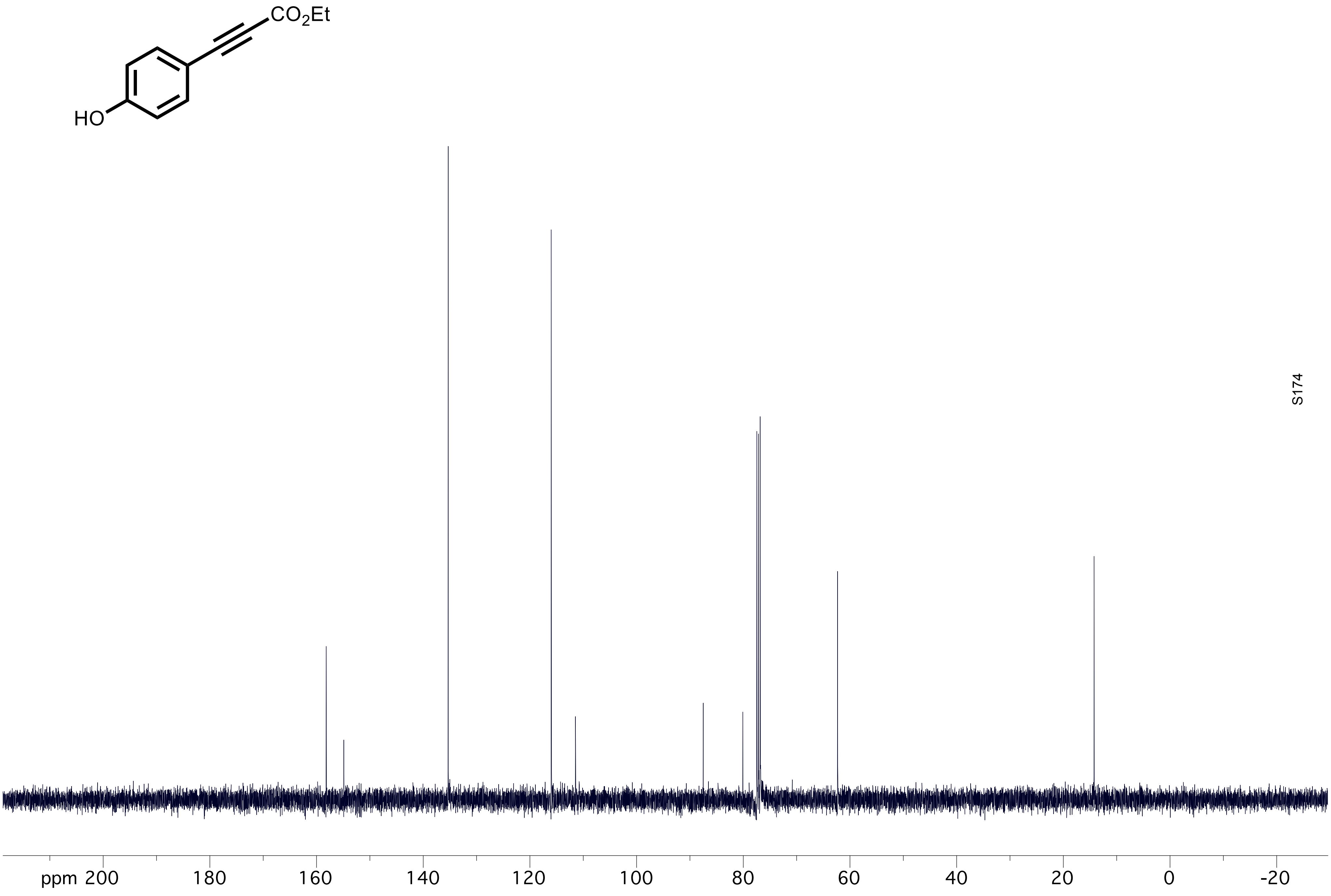
CO2Et
HO
ppm 200
180
160
140
120
100
80
60
40
20
0
-20

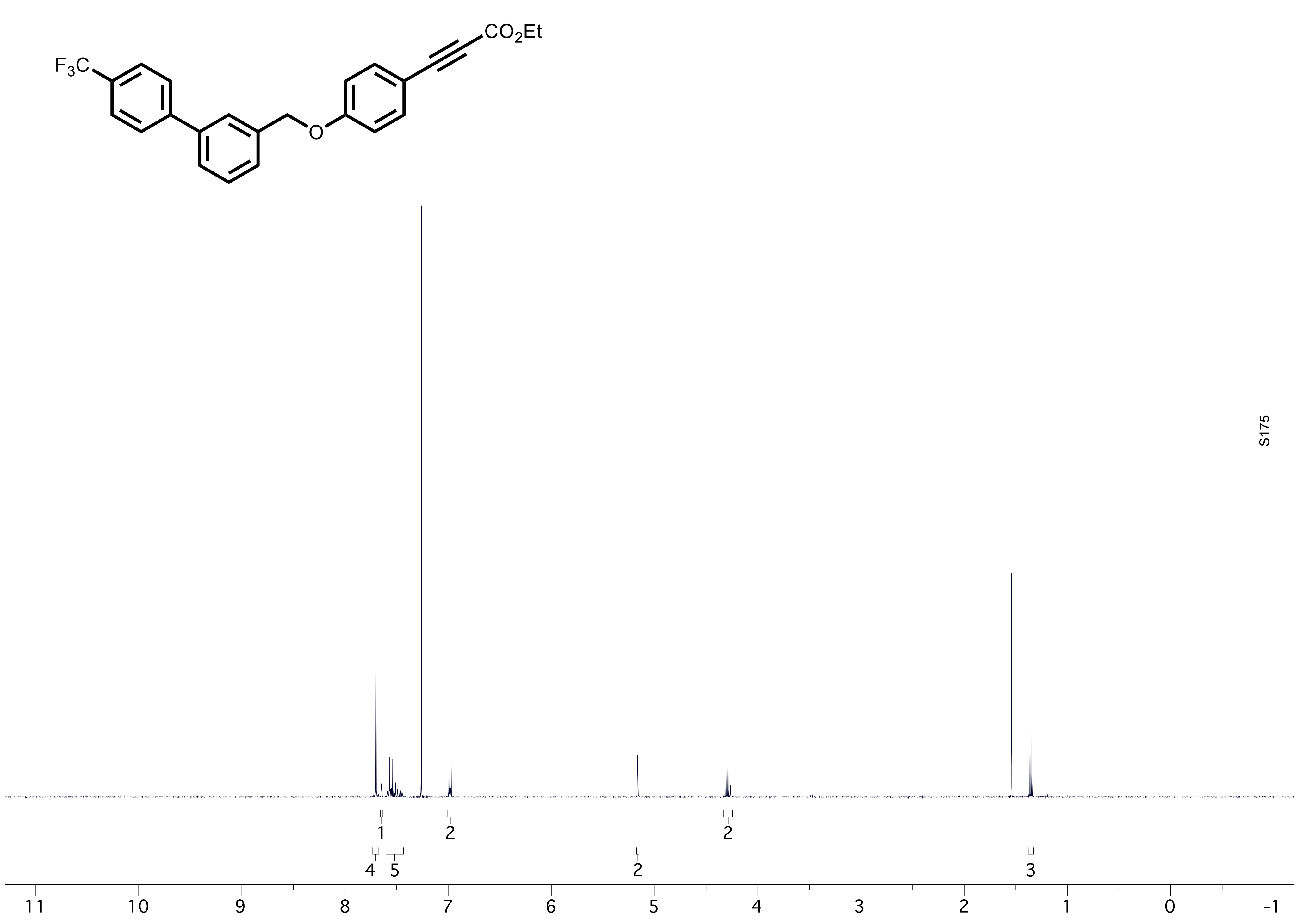
F3C
O
CO2Et
1
2
2
4
5
2
3
11
10
9
8
7
6
5
4
3
2
1
0
-1

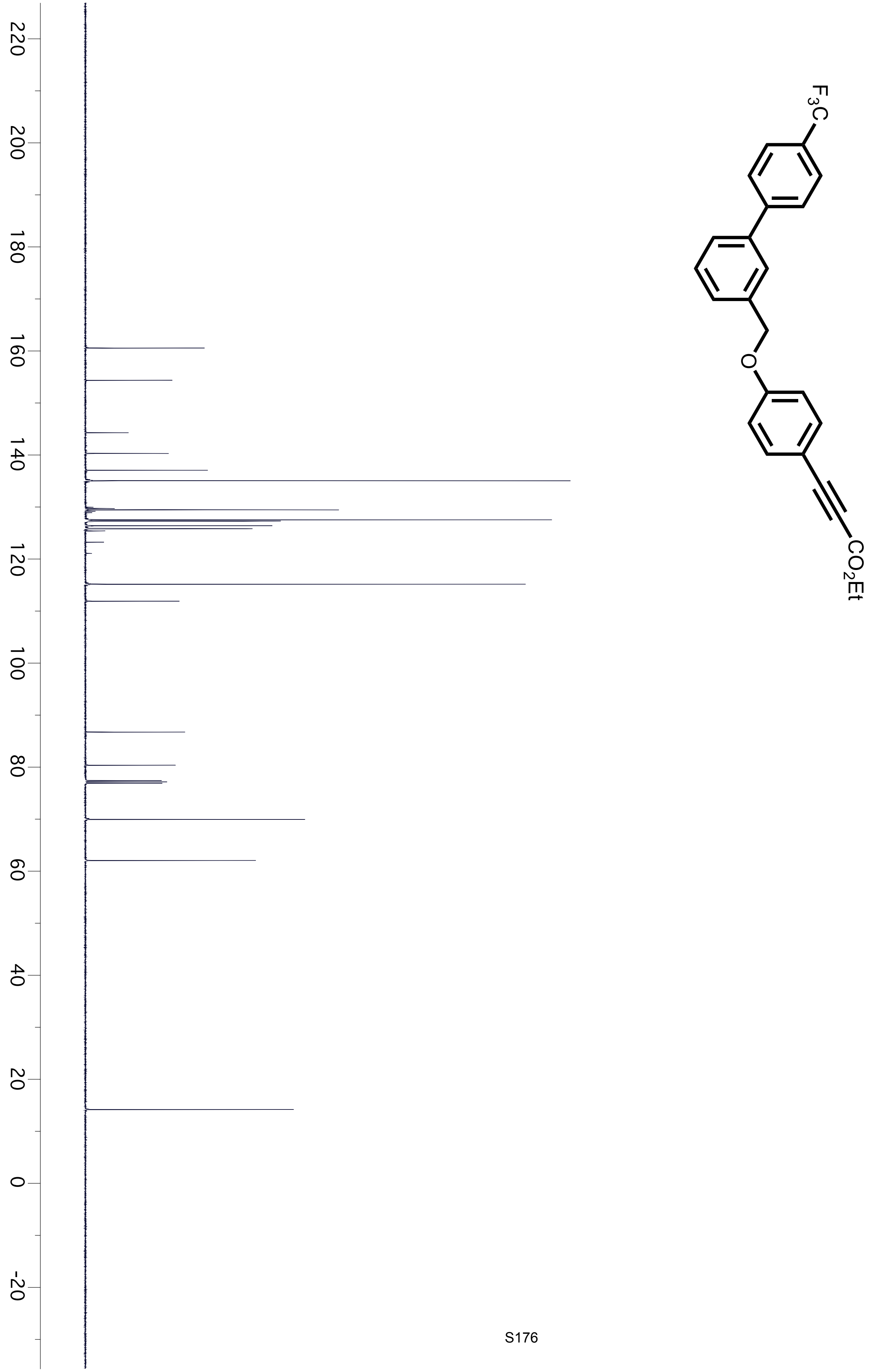
F3C
O
CO2Et
220
200
180
160
140
120
100
80
60
40
20
0
-20

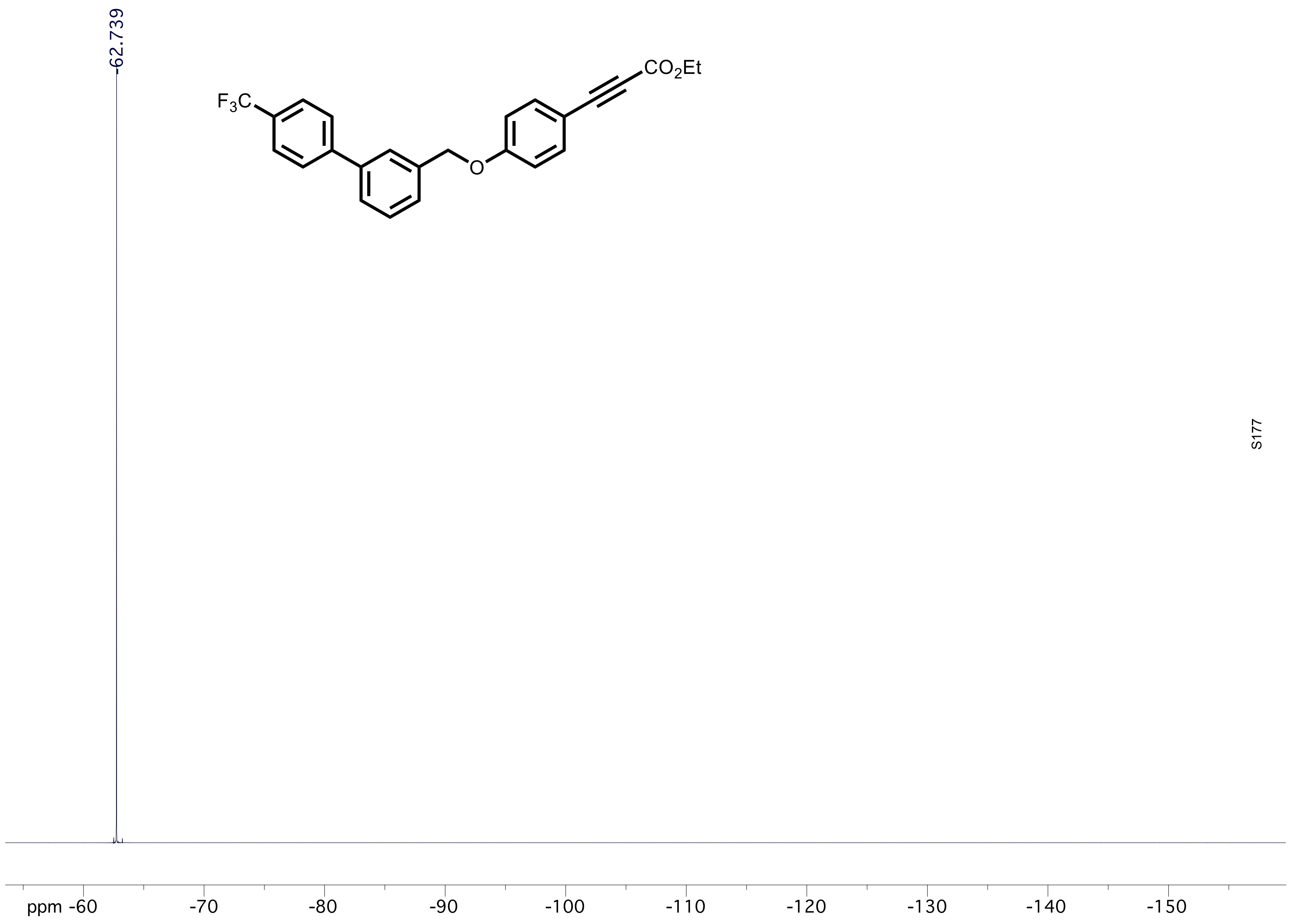
-62.739
F3C
O
CO2Et
ppm -60
-70
-80
-90
-100
-110
-120
-130
-140
-150

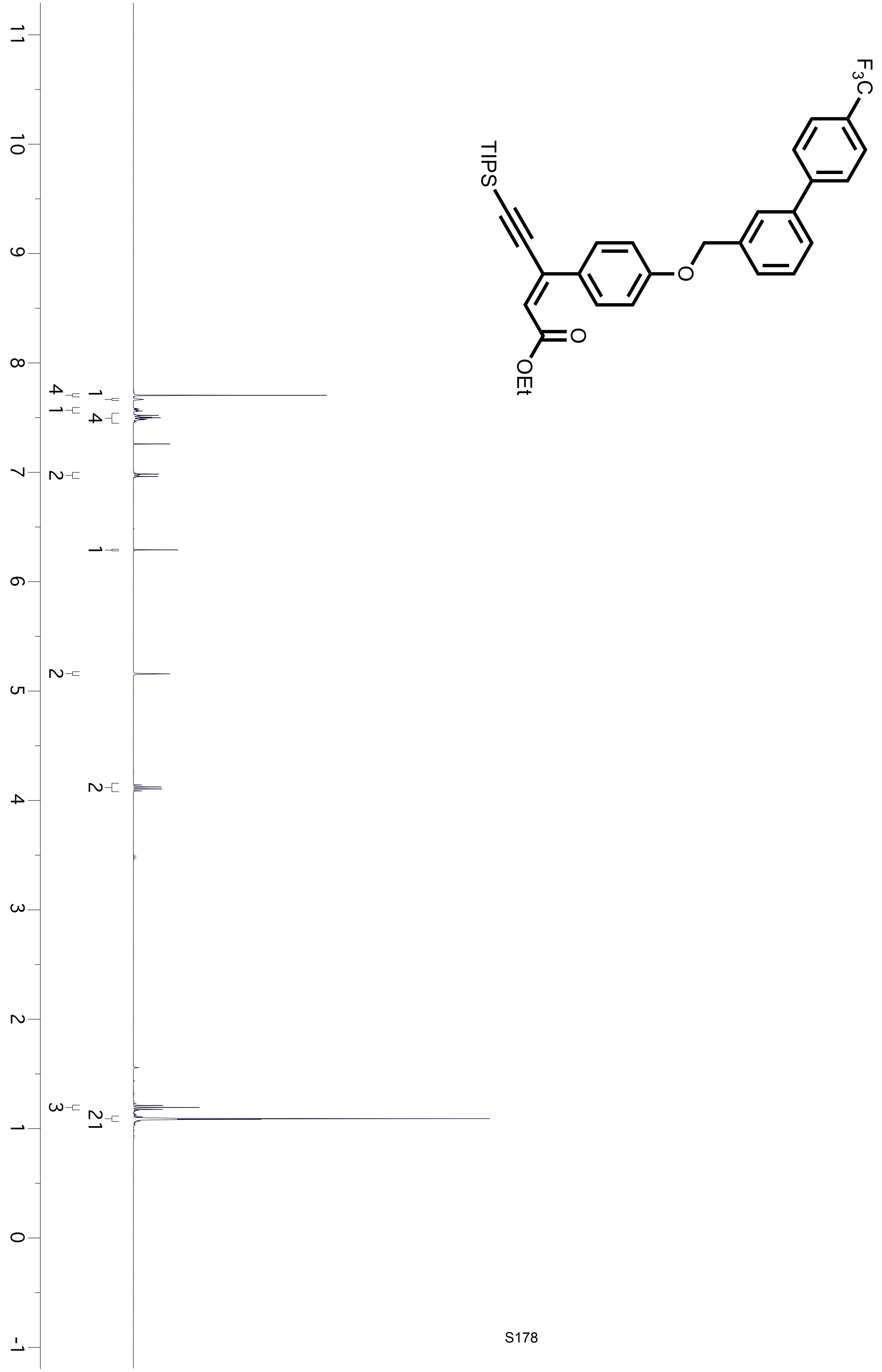
TIPS
O
OEt
O
F3C

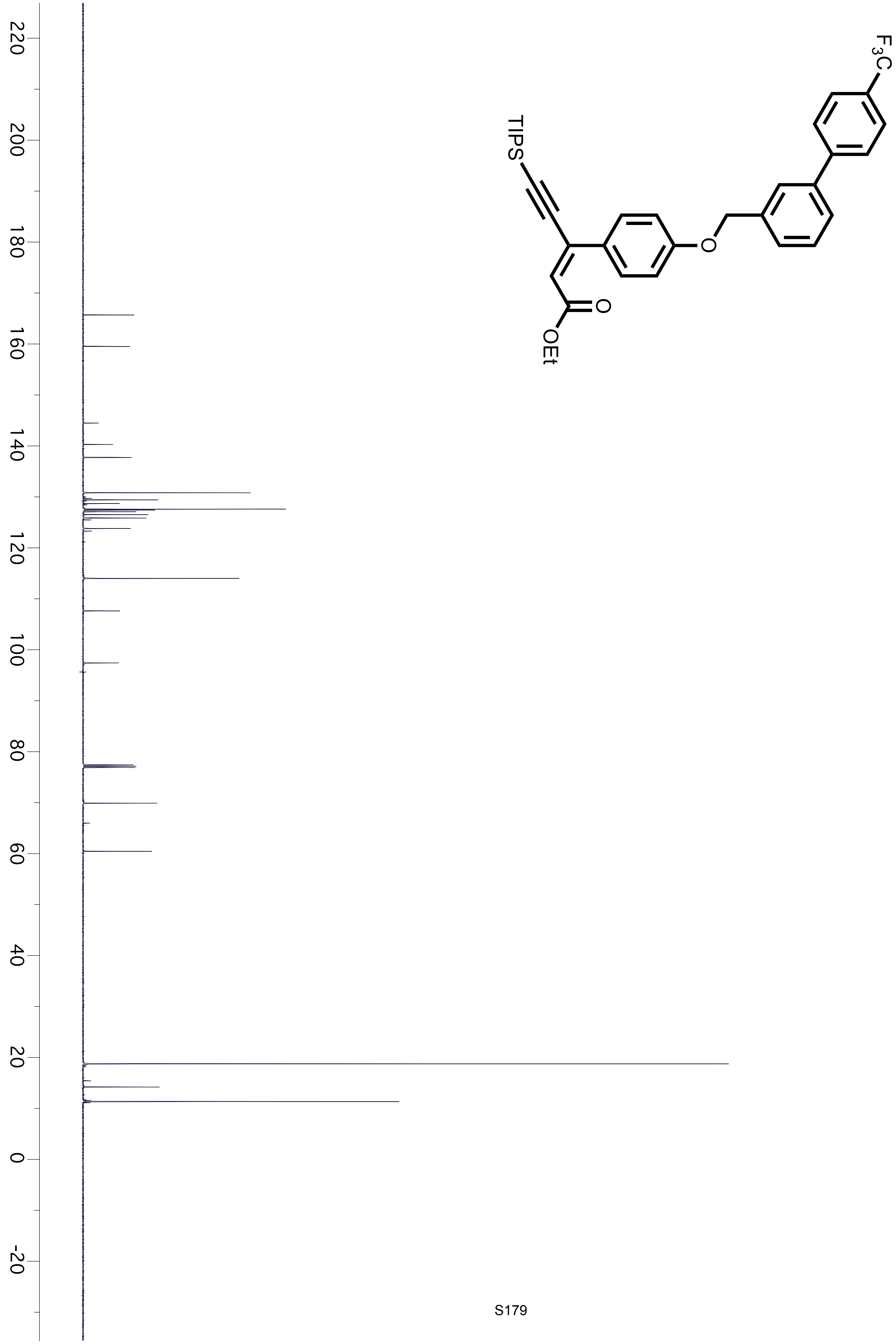
TIPS
O
O
OEt
F3C
220
200
180
160
140
120
100
80
60
40
20
0
-20

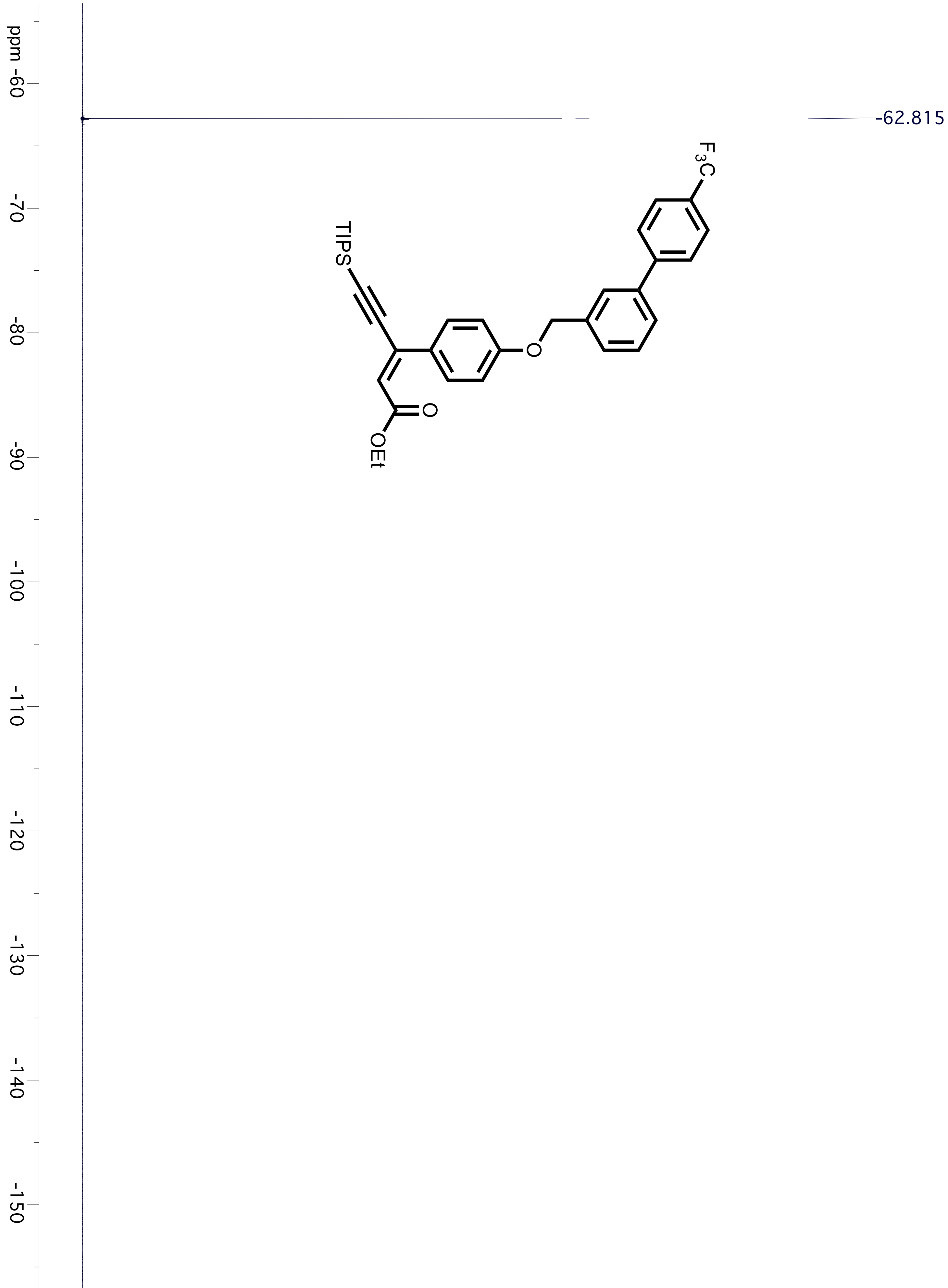
ppm
-60
-70
-80
-90
-100
-110
-120
-130
-140
-150
-62.815
TIPS
O
OEt
F3C

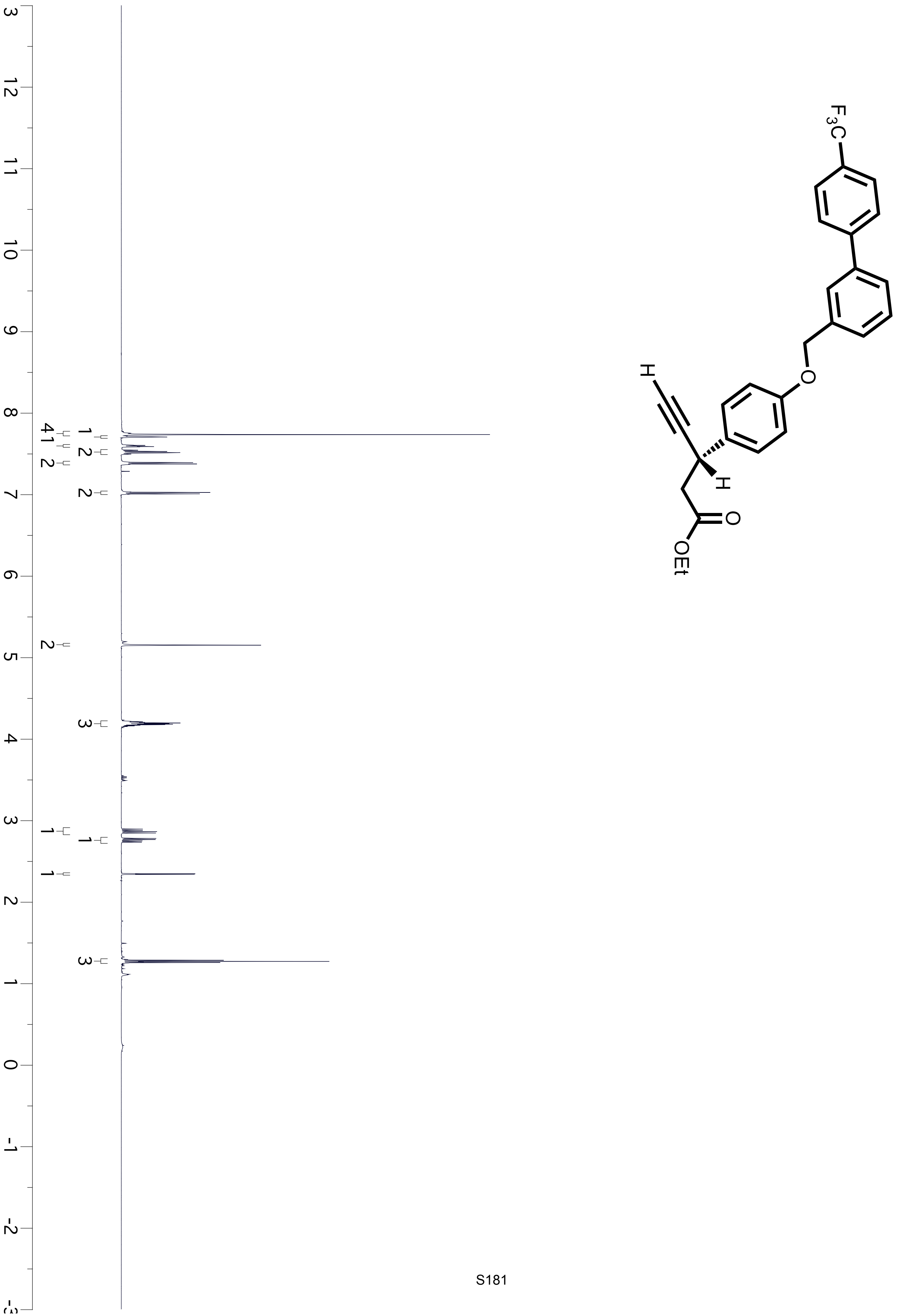

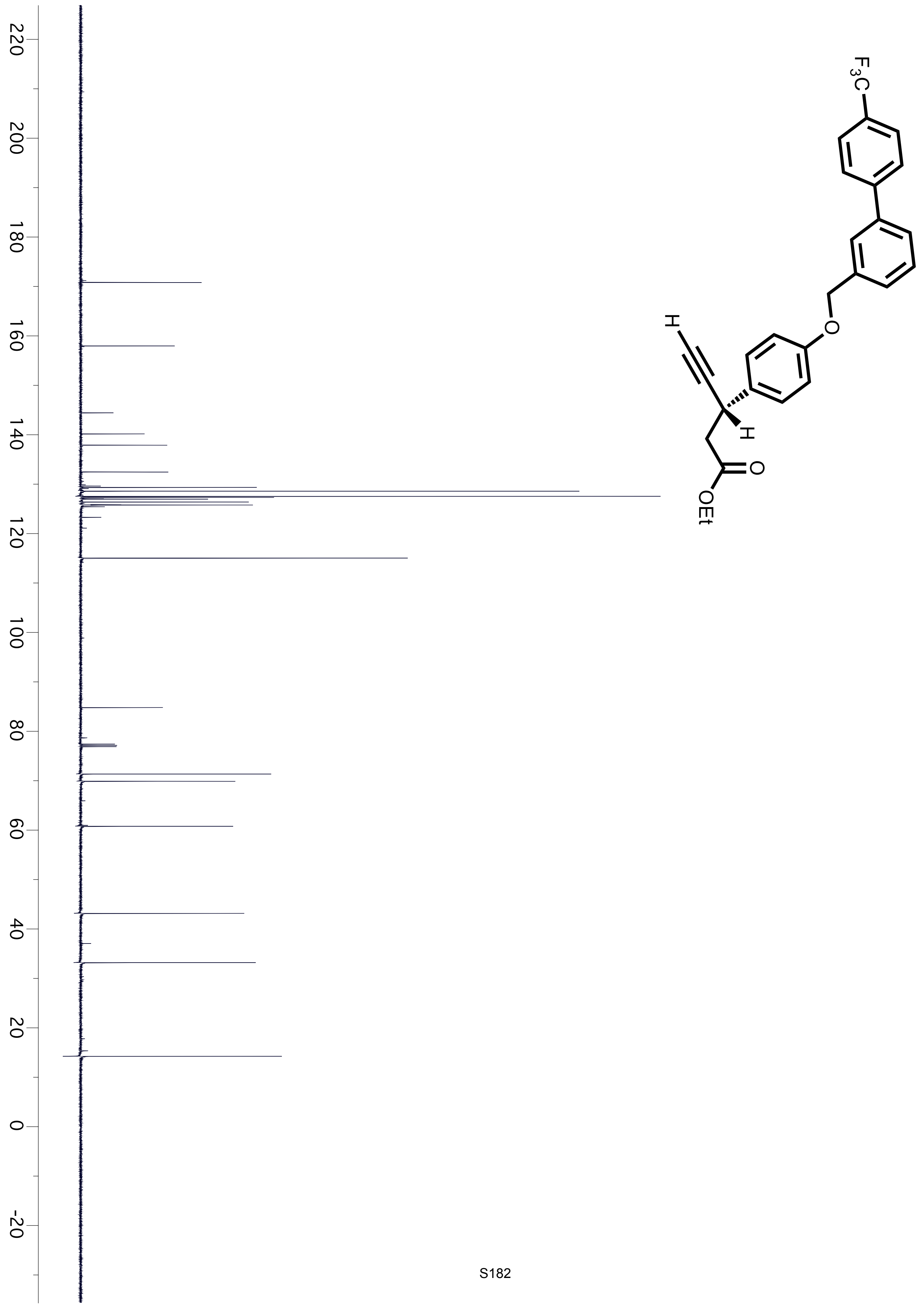

220
200
180
160
140
120
100
80
60
40
20
0
-20
F_3C
O
H
O
OEt
H

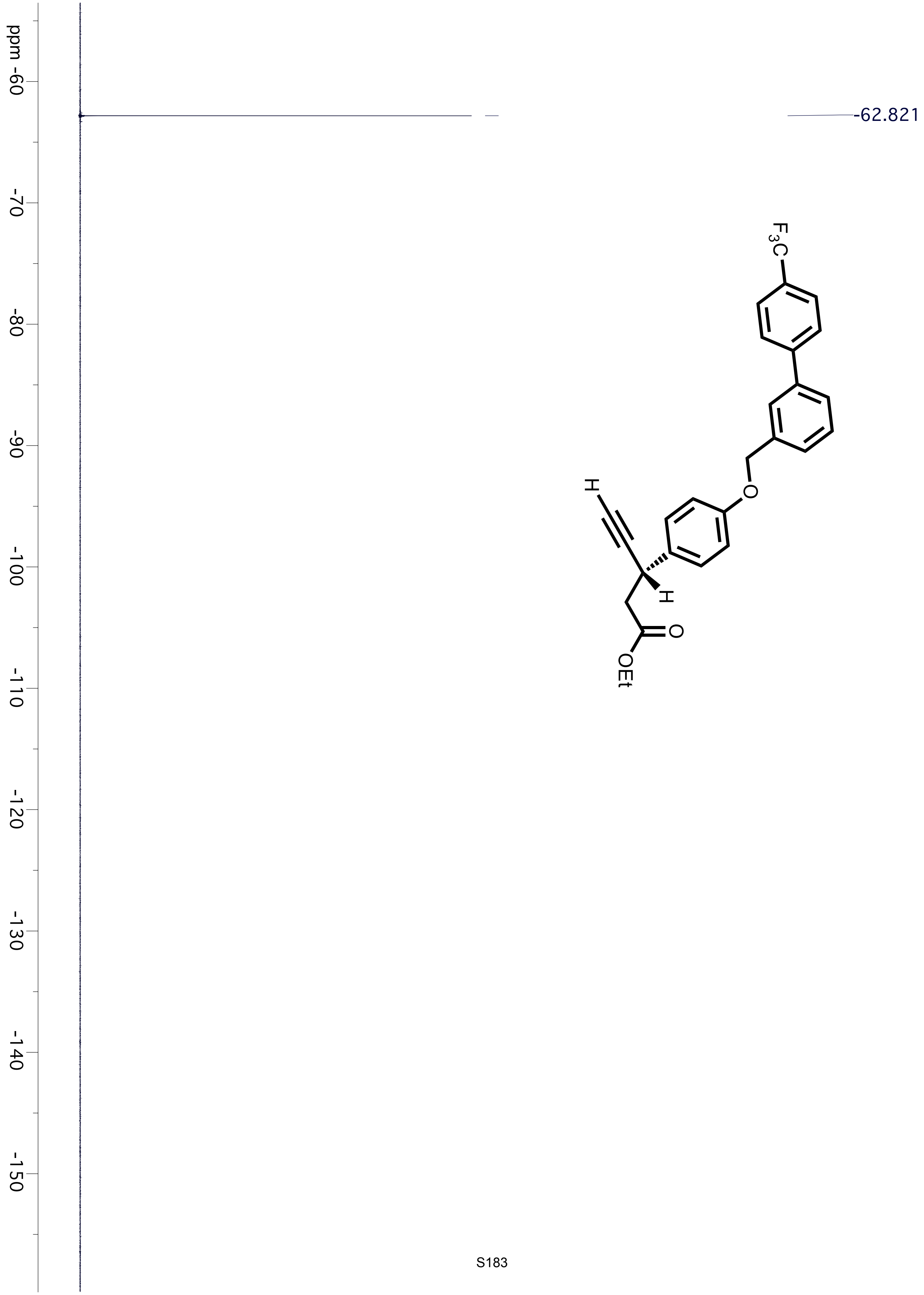
-62.821
F3C
O
H
H
O
OEt
ppm -60
-70
-80
-90
-100
-110
-120
-130
-140
-150

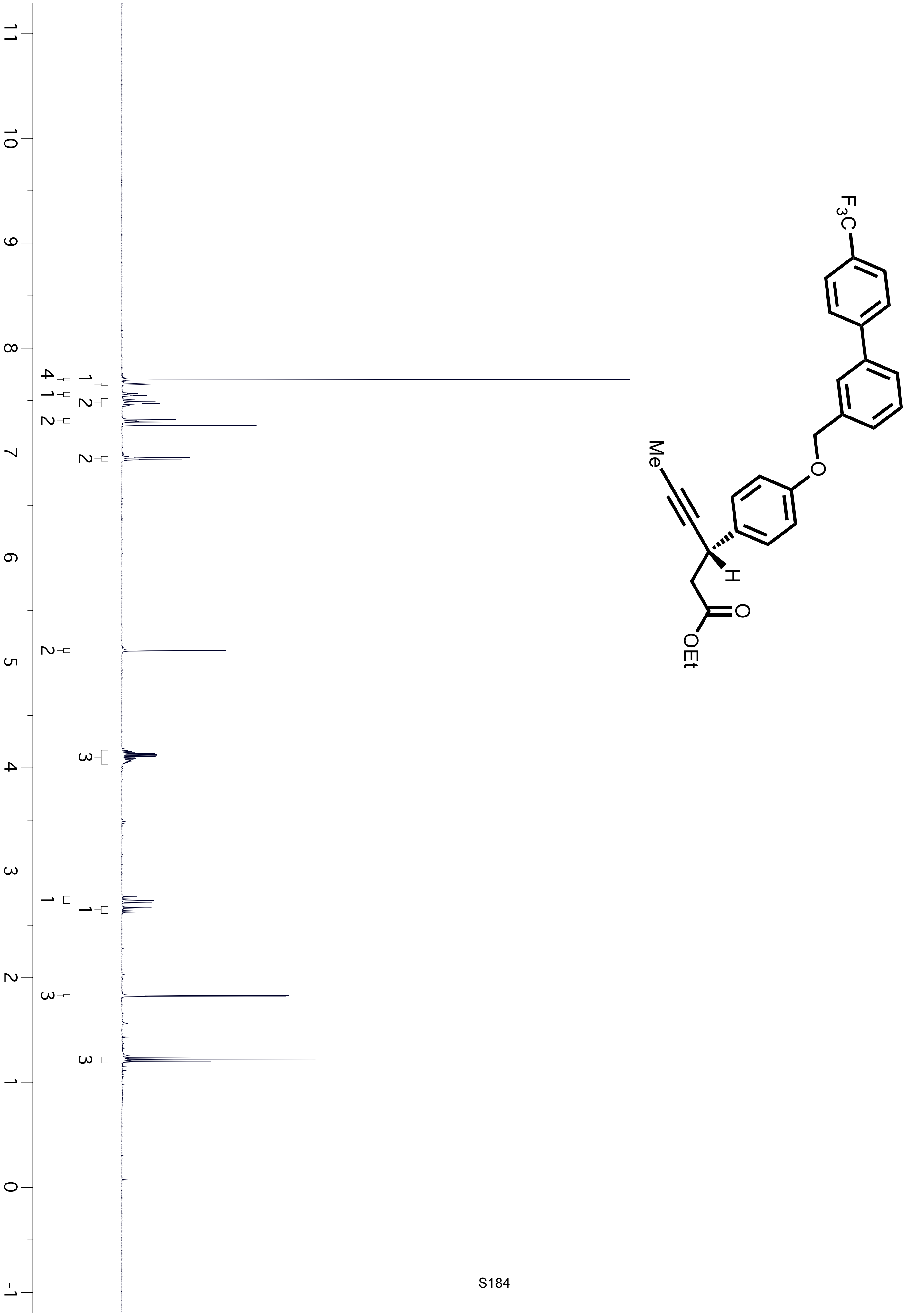
F3C
Me
O
H
O
OEt

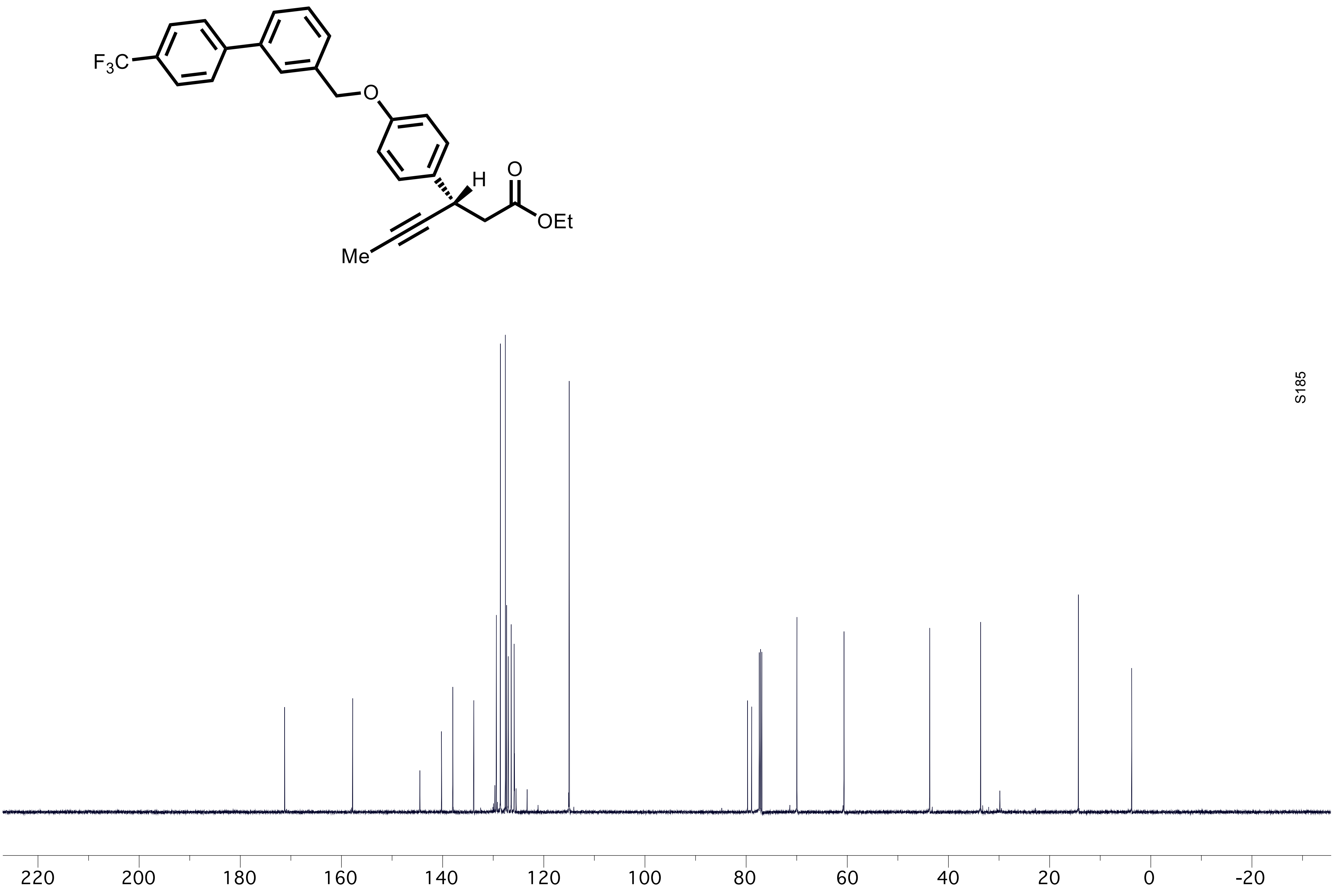

F3C
O
H
O
OEt
Me
220
200
180
160
140
120
100
80
60
40
20
0
-20

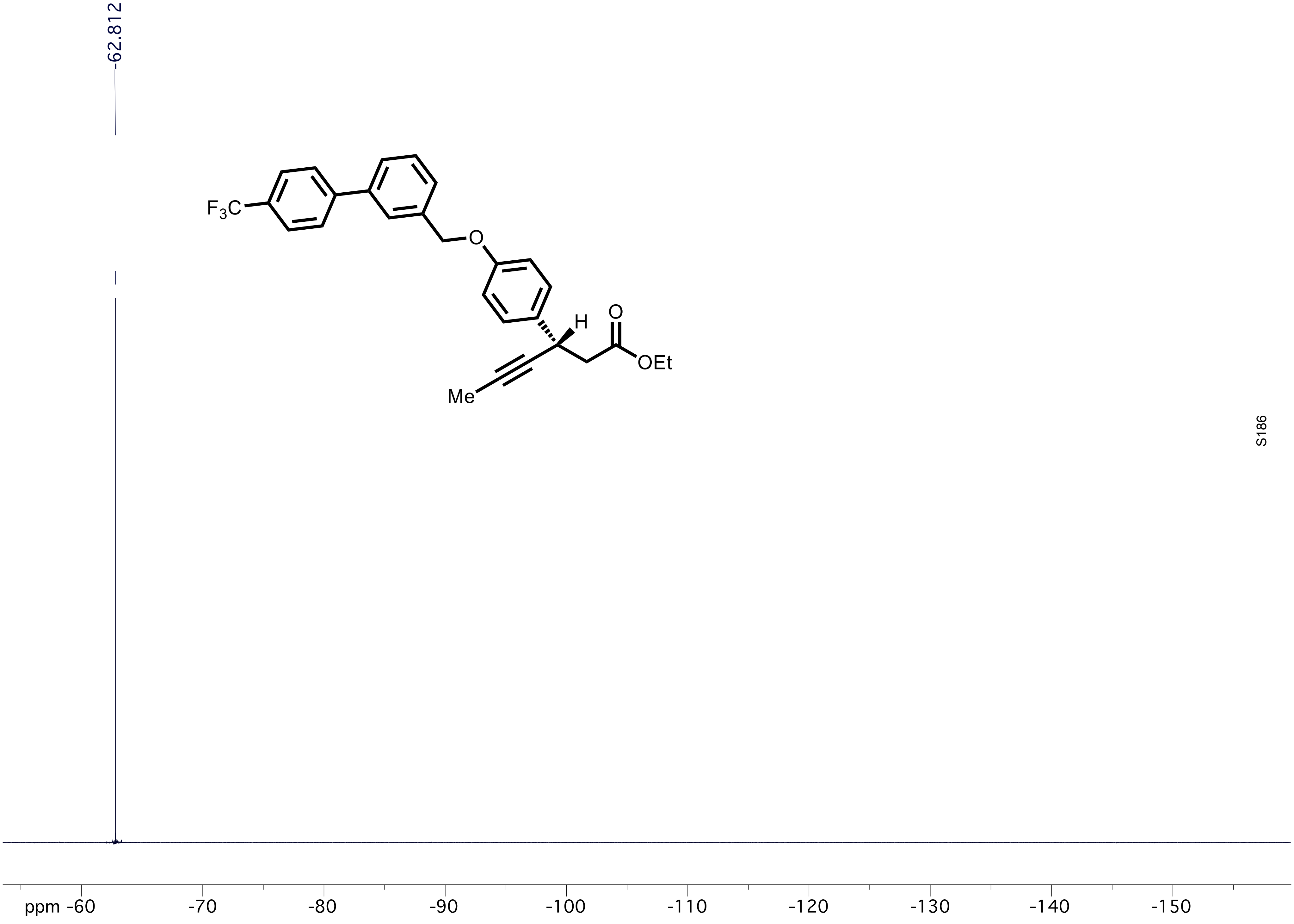

-62.812
F3C
O
H
O
OEt
Me
ppm -60
-70
-80
-90
-100
-110
-120
-130
-140
-150

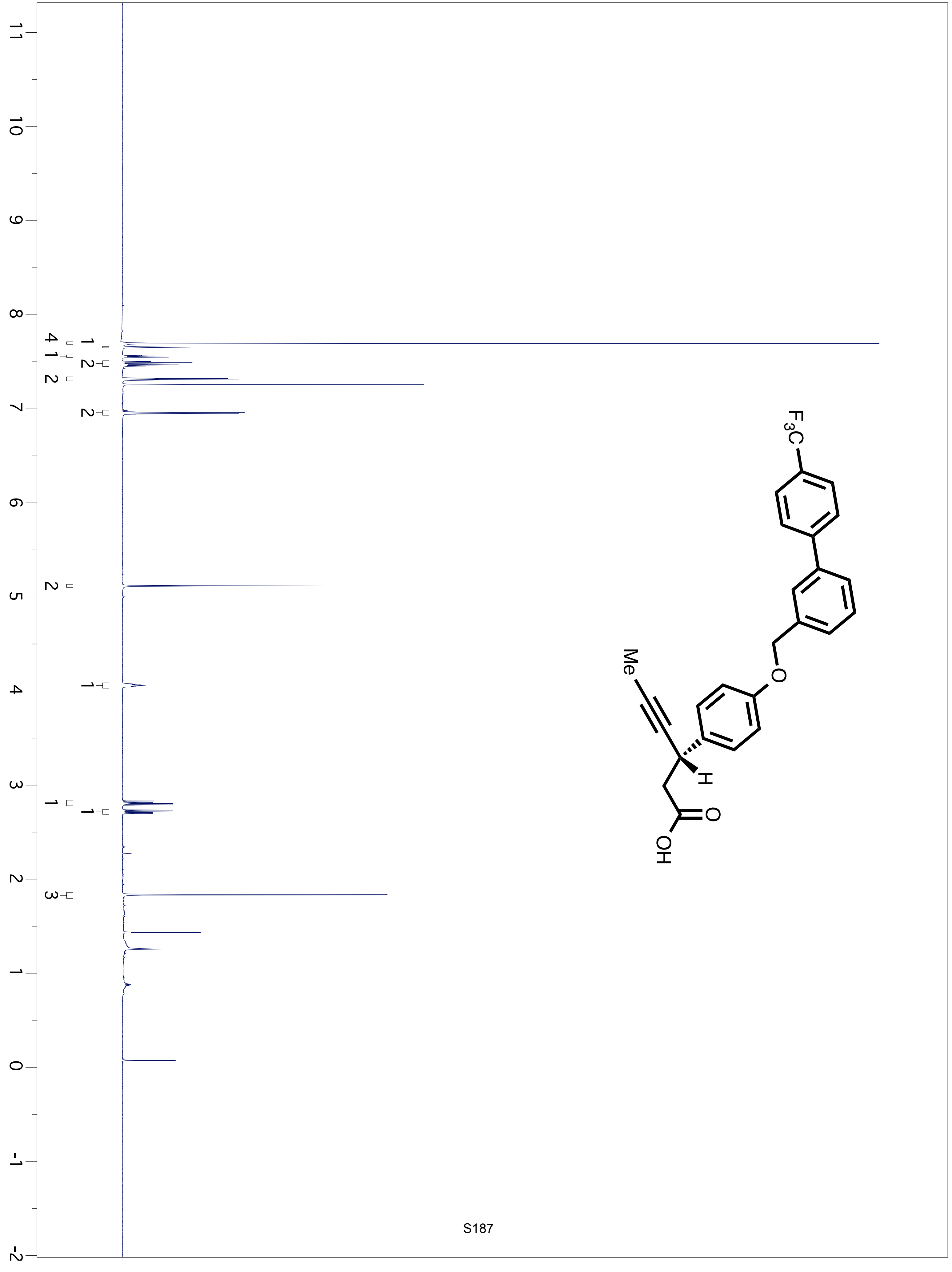

F3C
O
Me
H
O
OH

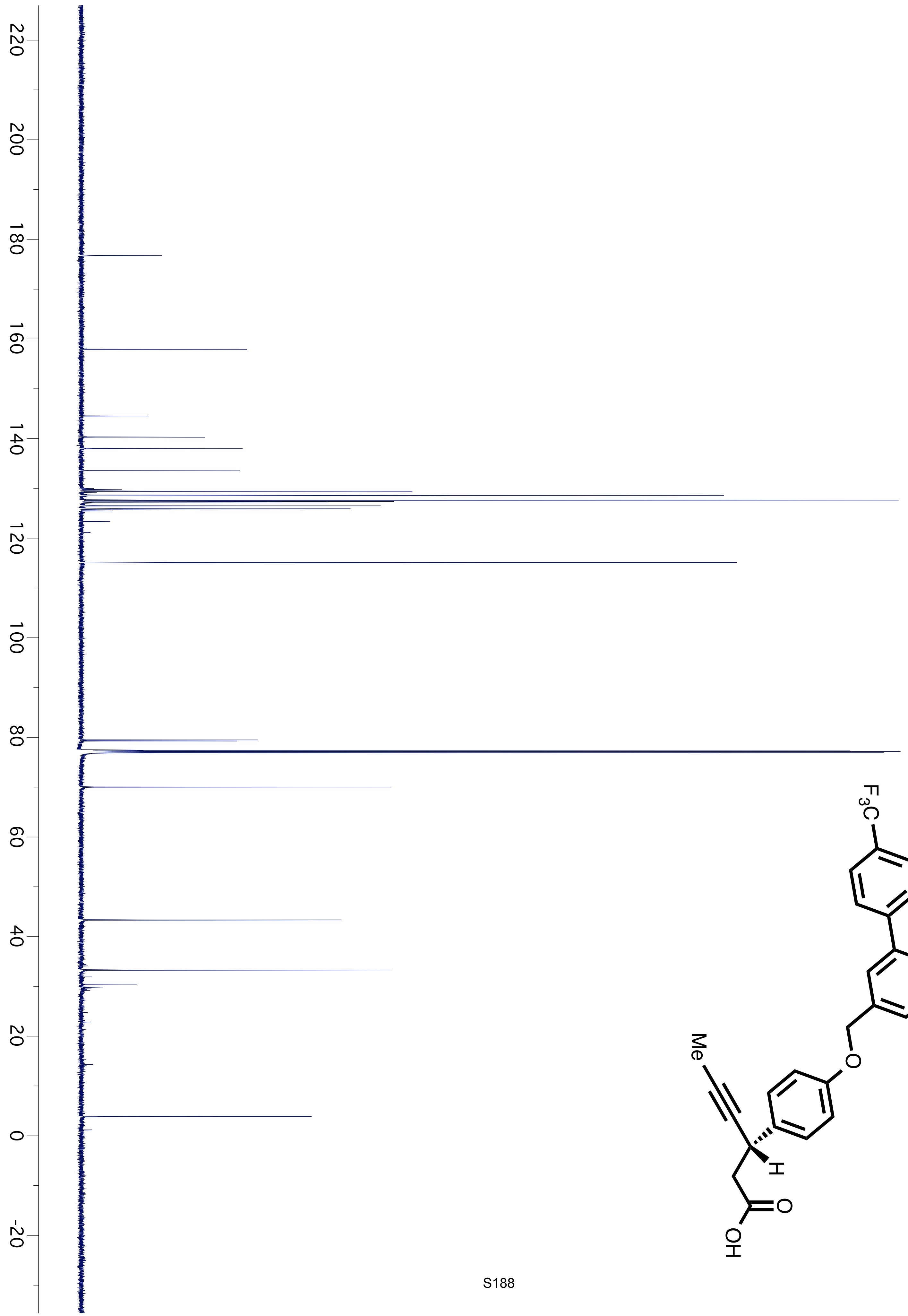
220
200
180
160
140
120
100
80
60
40
20
0
-20
F3C
O
Me
H
O
OH

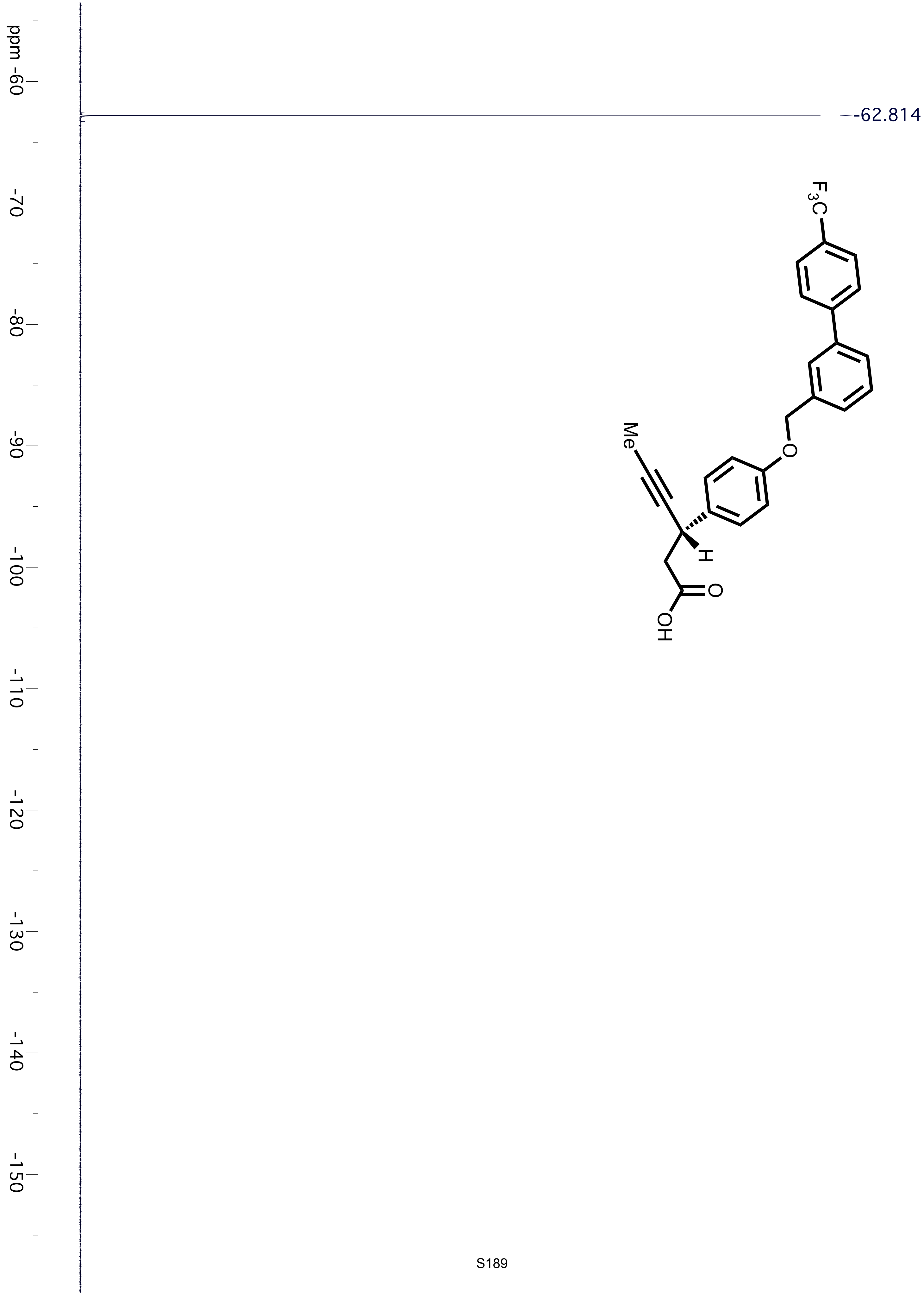
ppm -60
-70
-80
-90
-100
-110
-120
-130
-140
-150
-62.814
F3C
O
Me
H
O
OH

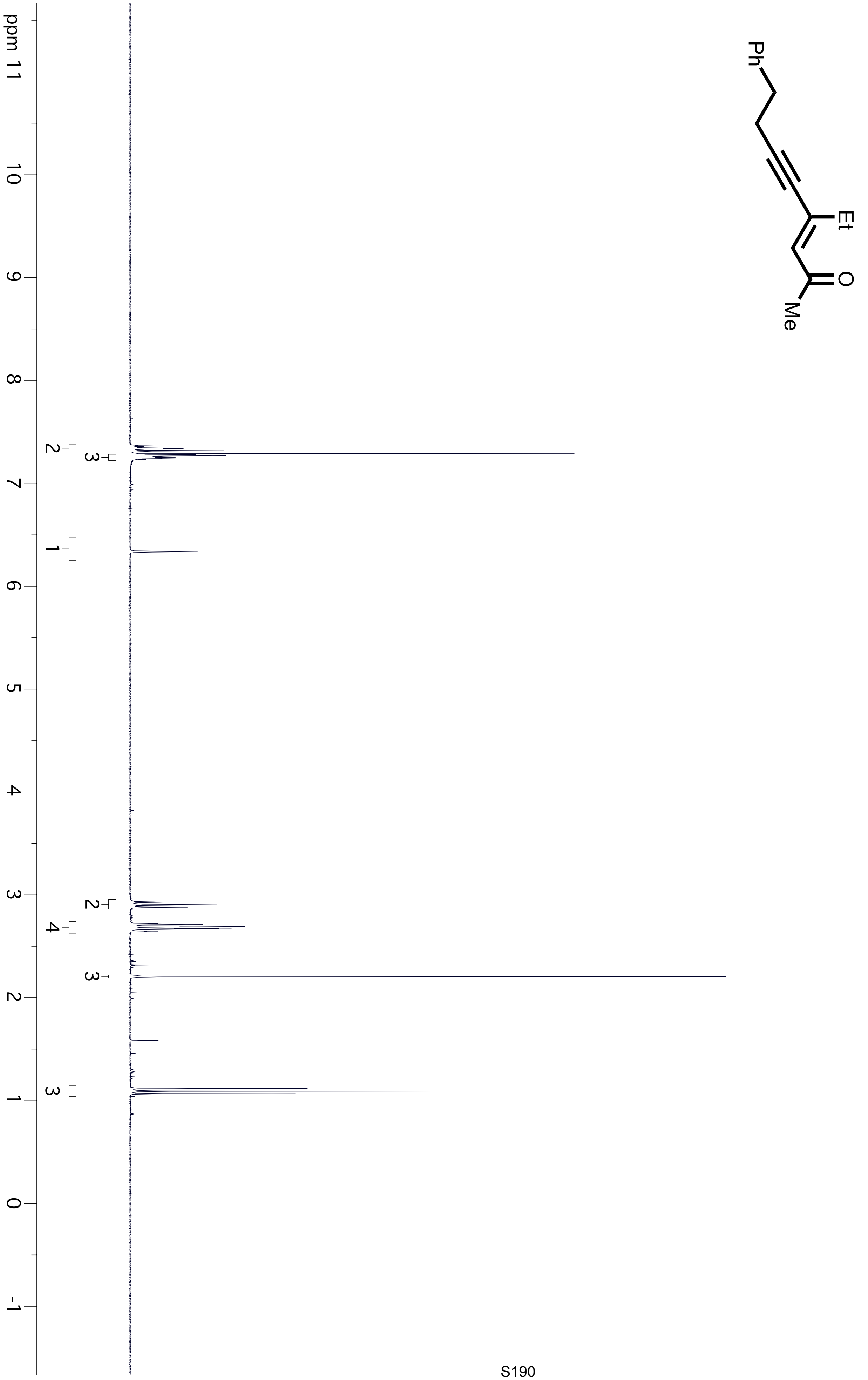
Ph
Et
O
Me
ppm

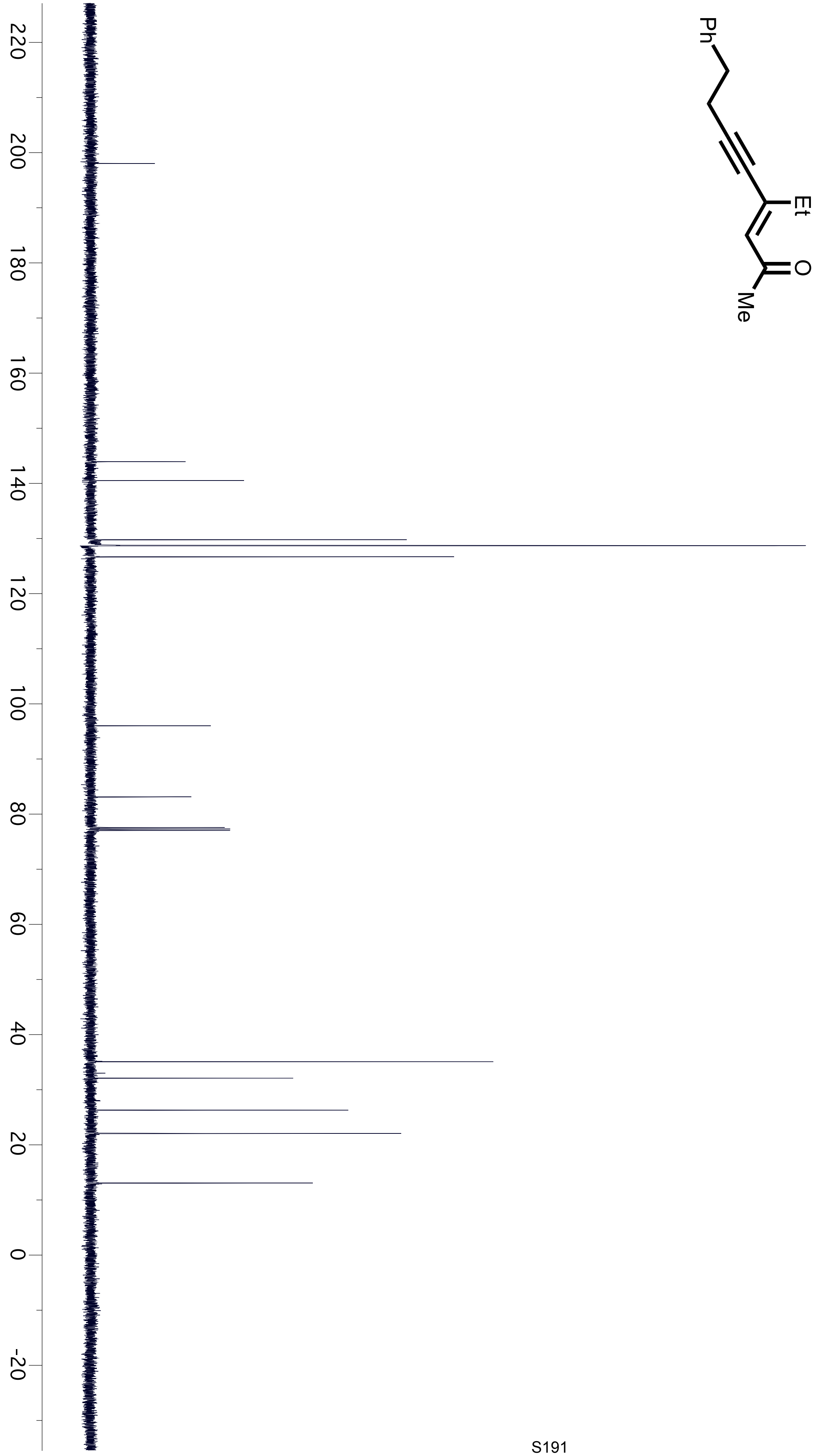
Ph
Et
O
Me
220
200
180
160
140
120
100
80
60
40
20
0
-20

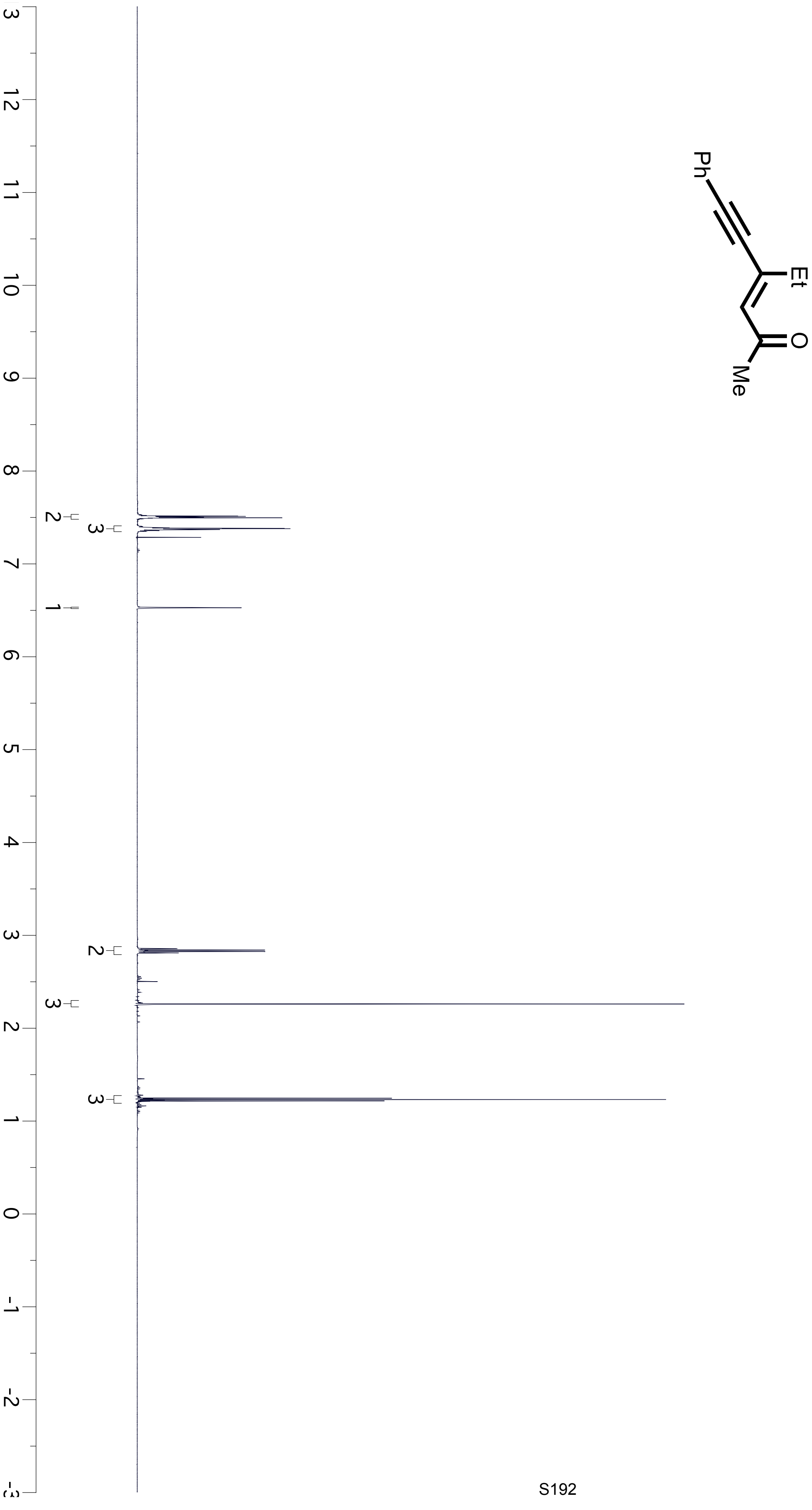
Ph
Et
O
Me

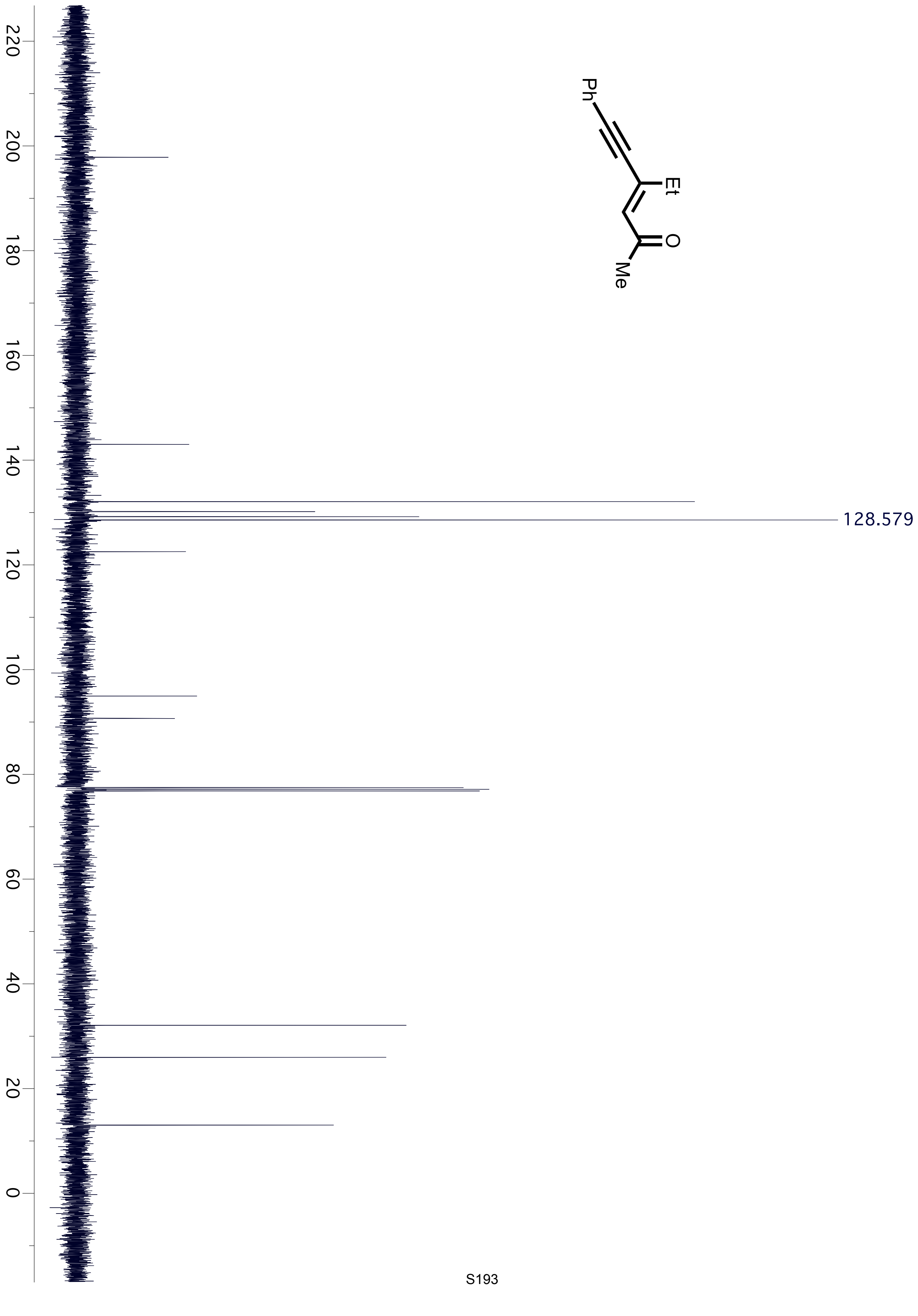
Ph
Et
O
Me
128.579
220
200
180
160
140
120
100
80
60
40
20
0

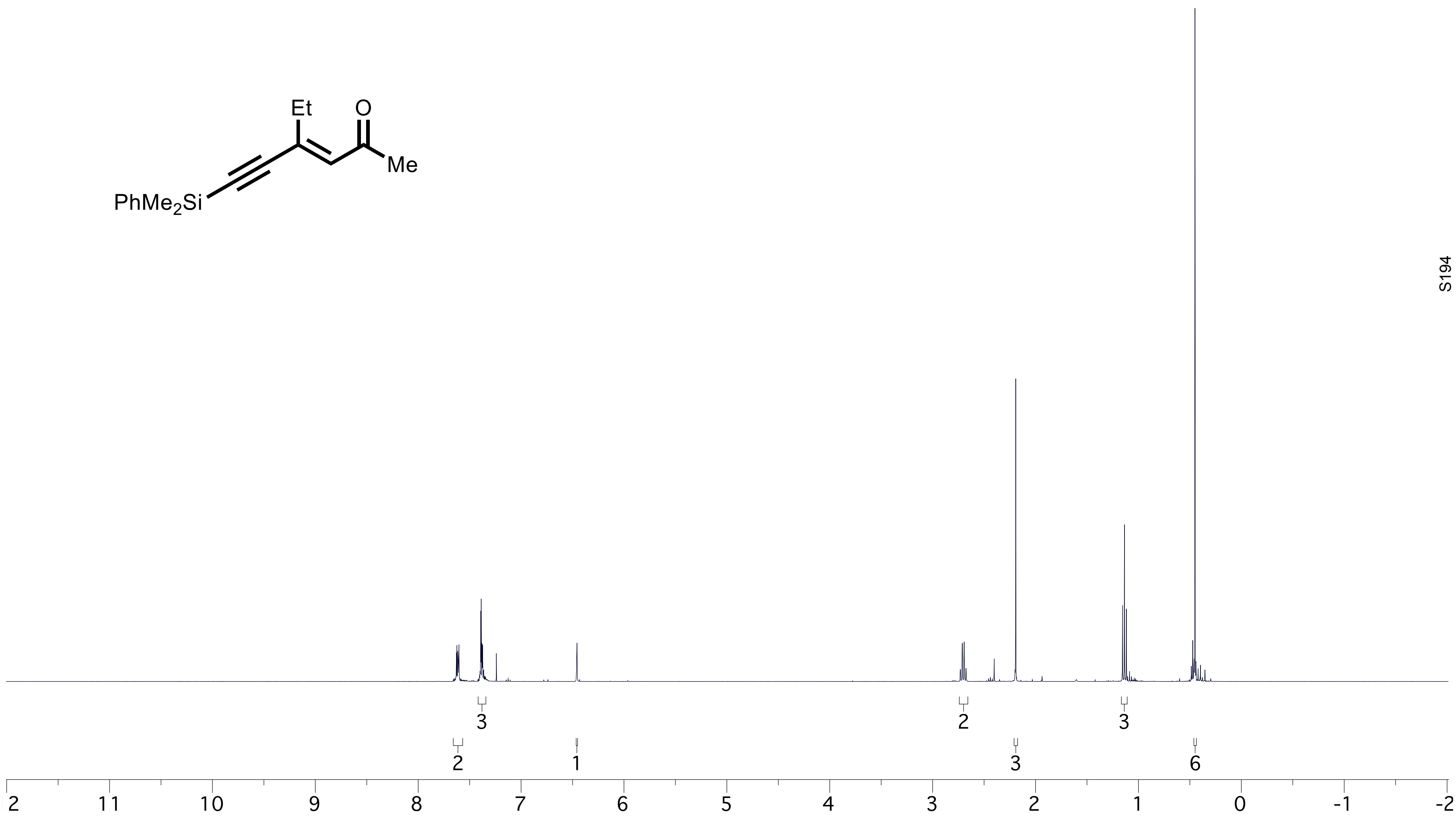

Et
O
Me
PhMe2Si
3
2
3
2
1
3
6
2
11
10
9
8
7
6
5
4
3
2
1
0
-1
-2

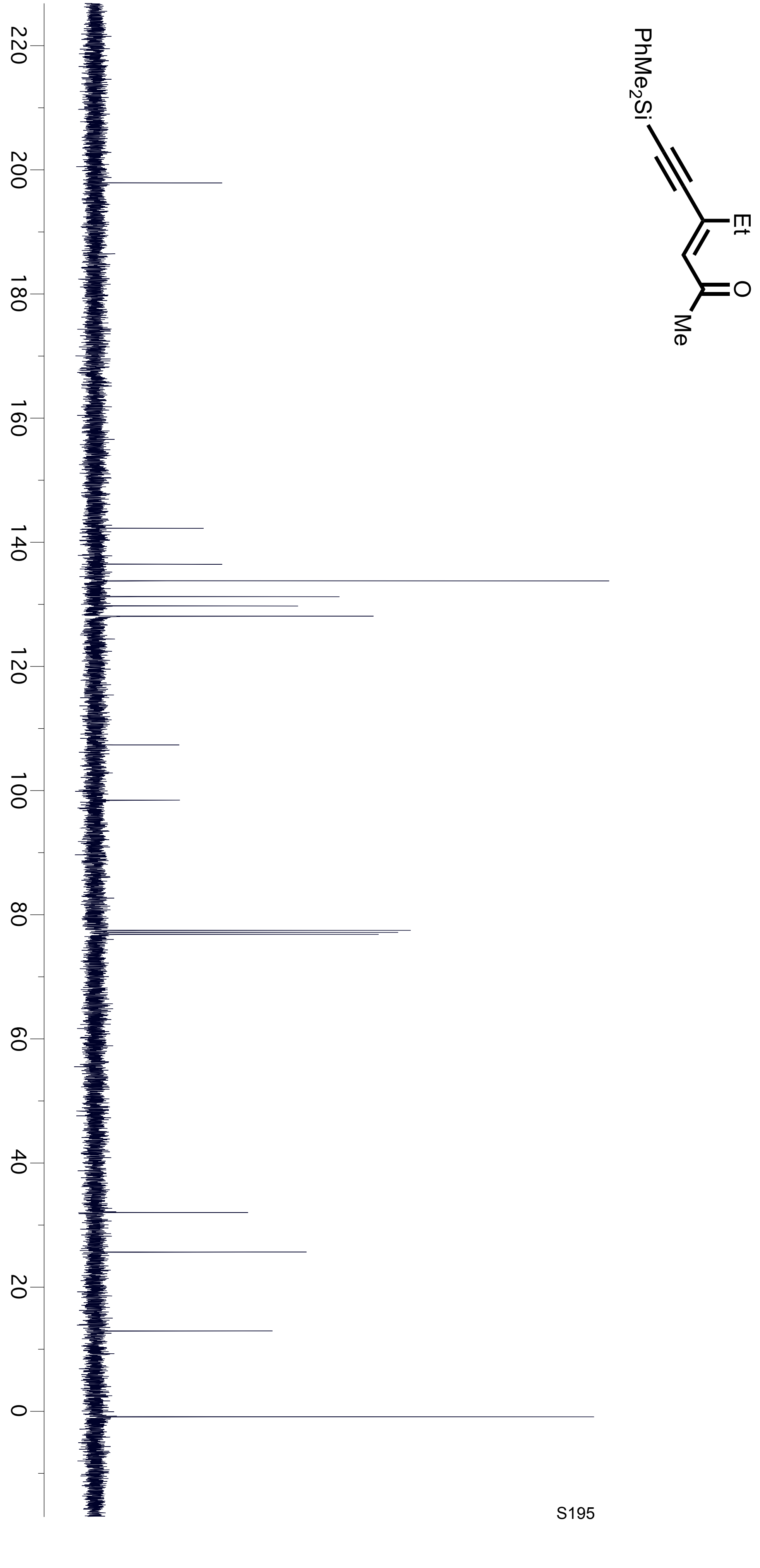
PhMe$_2$Si
Et
O
Me
220
200
180
160
140
120
100
80
60
40
20
0

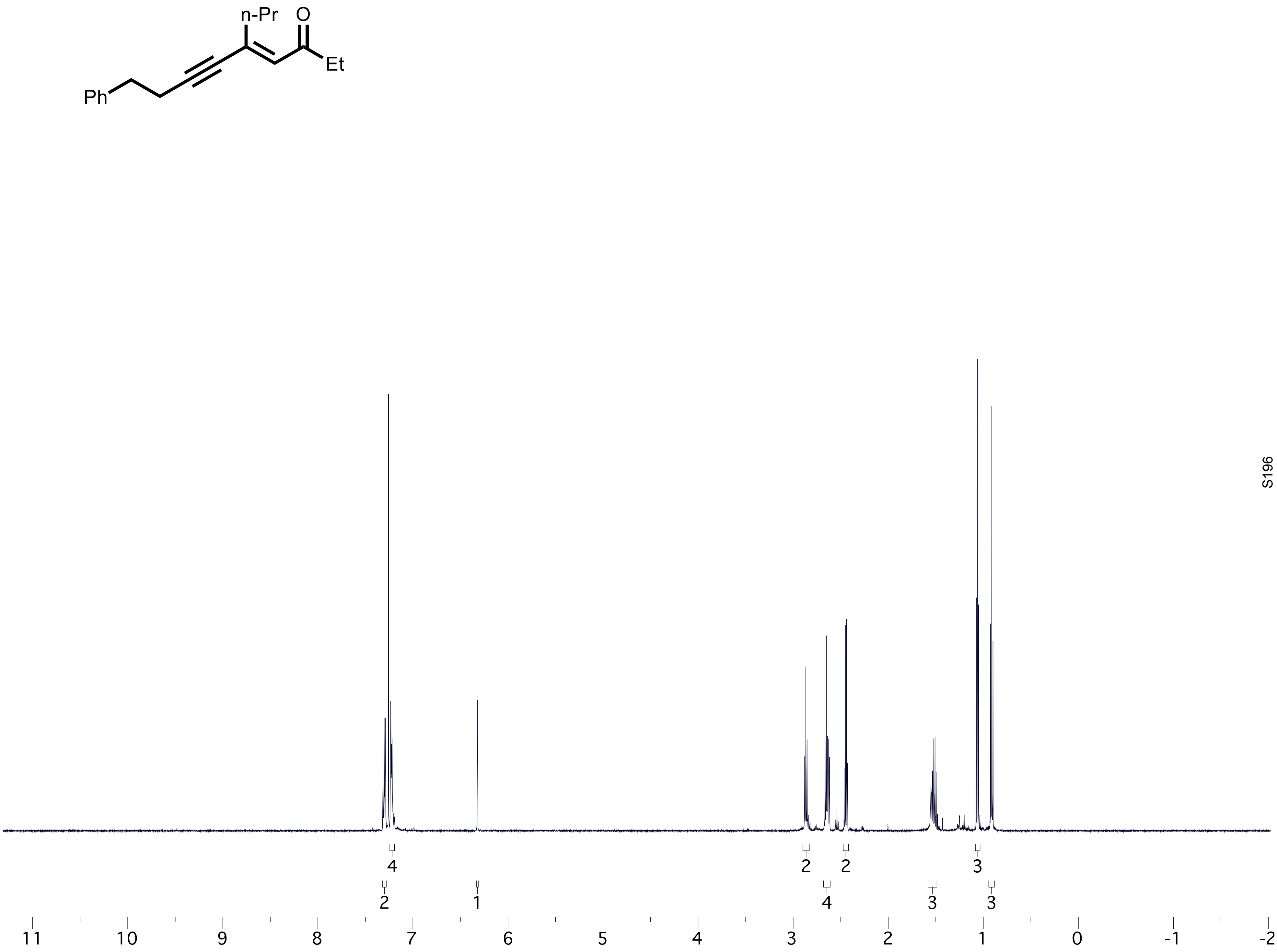
n-Pr
O
Et
Ph
4
2
2
3
2
1
4
3
3
11
10
9
8
7
6
5
4
3
2
1
0
-1
-2

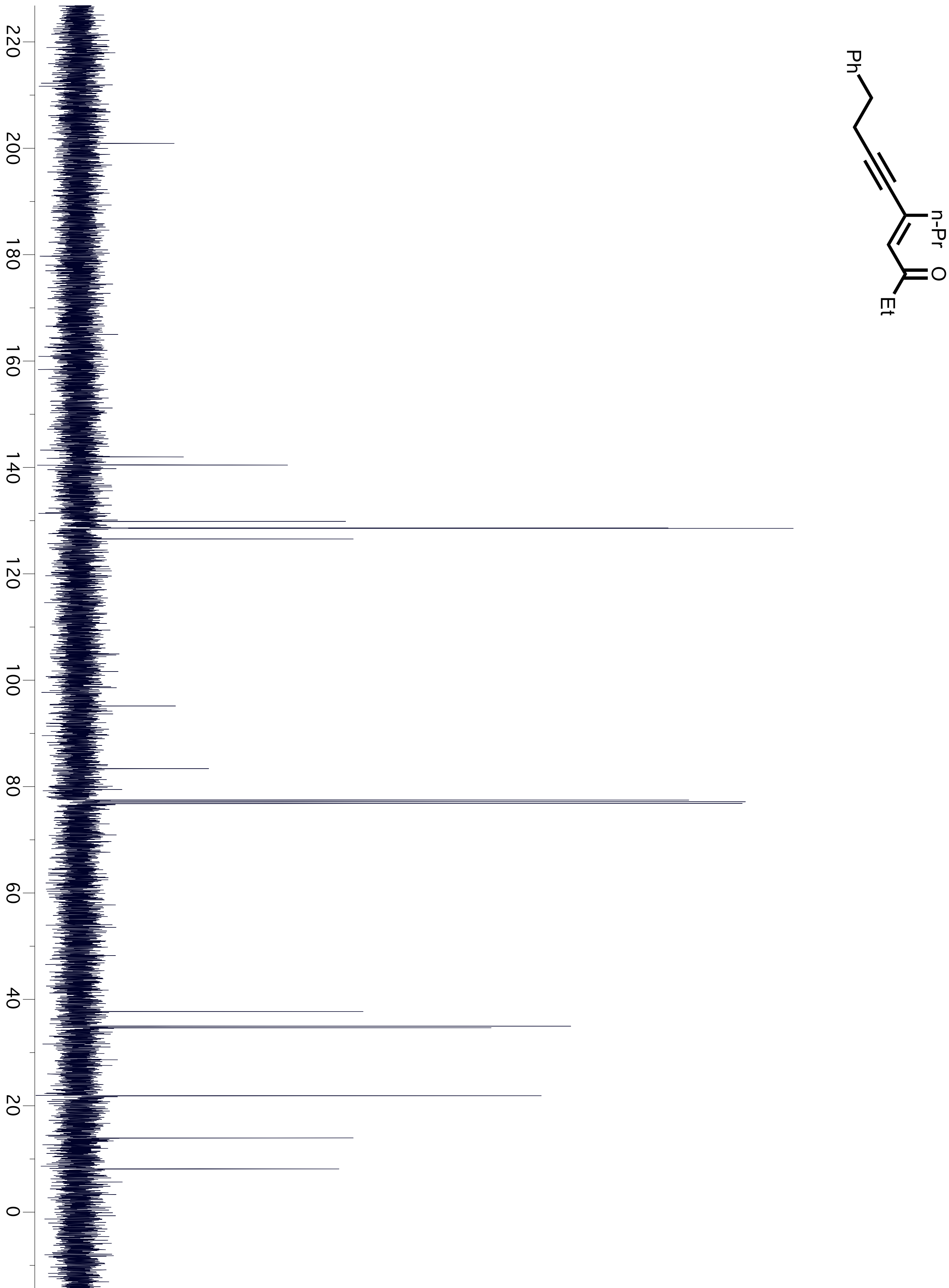
Ph
n-Pr
O
Et
220
200
180
160
140
120
100
80
60
40
20
0

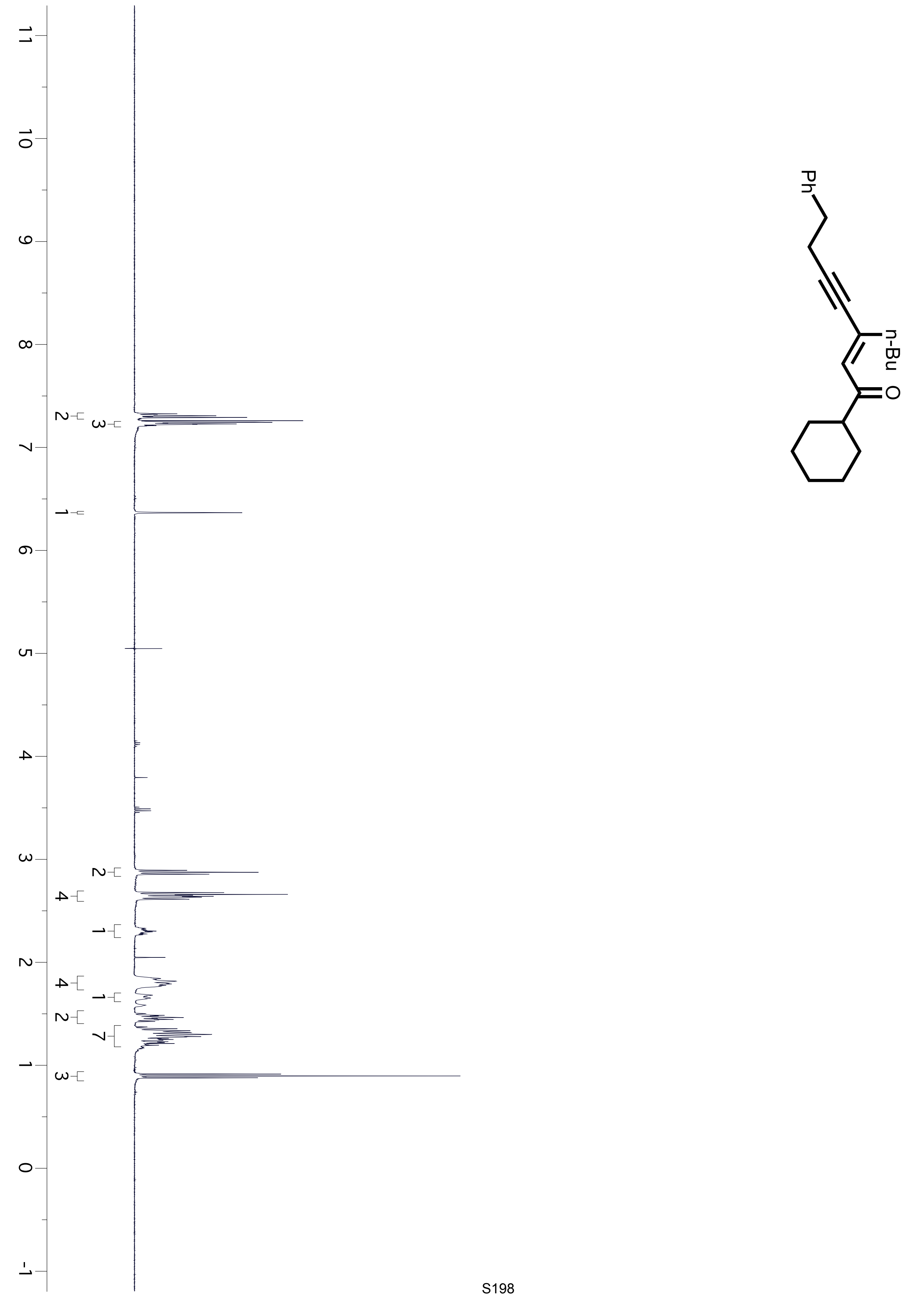

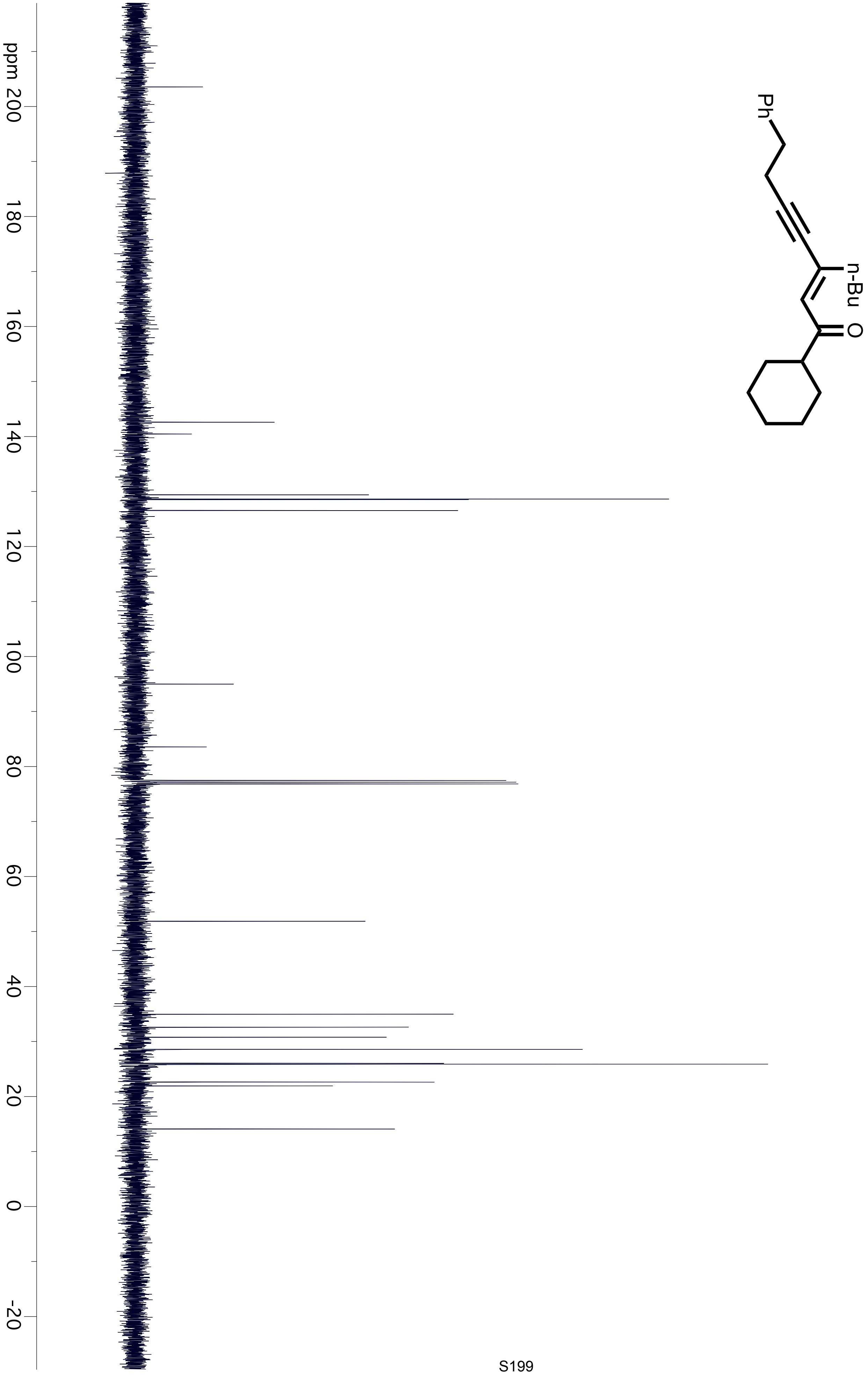
ppm 200
180
160
140
120
100
80
60
40
20
0
-20
Ph
n-Bu
O

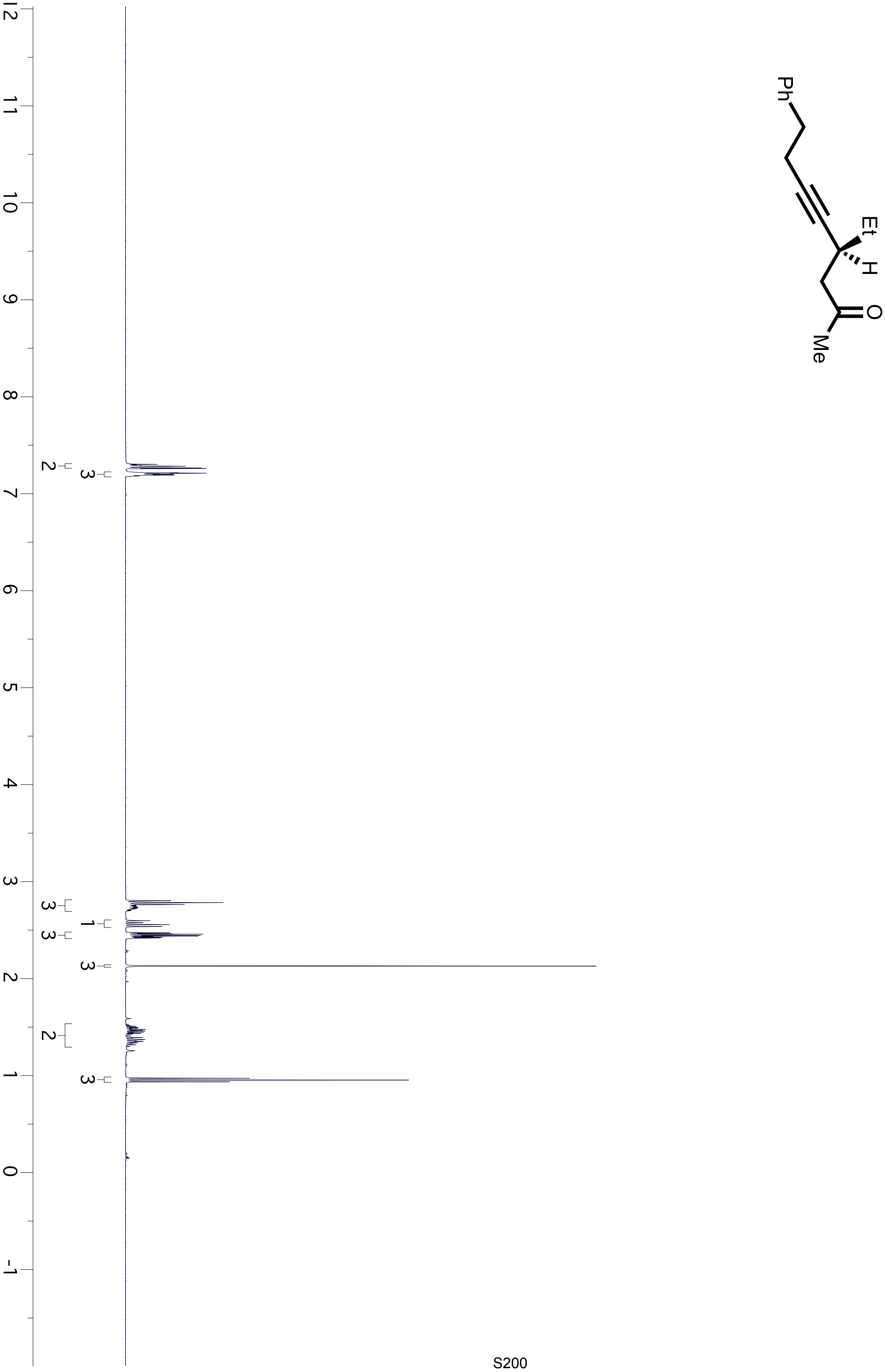

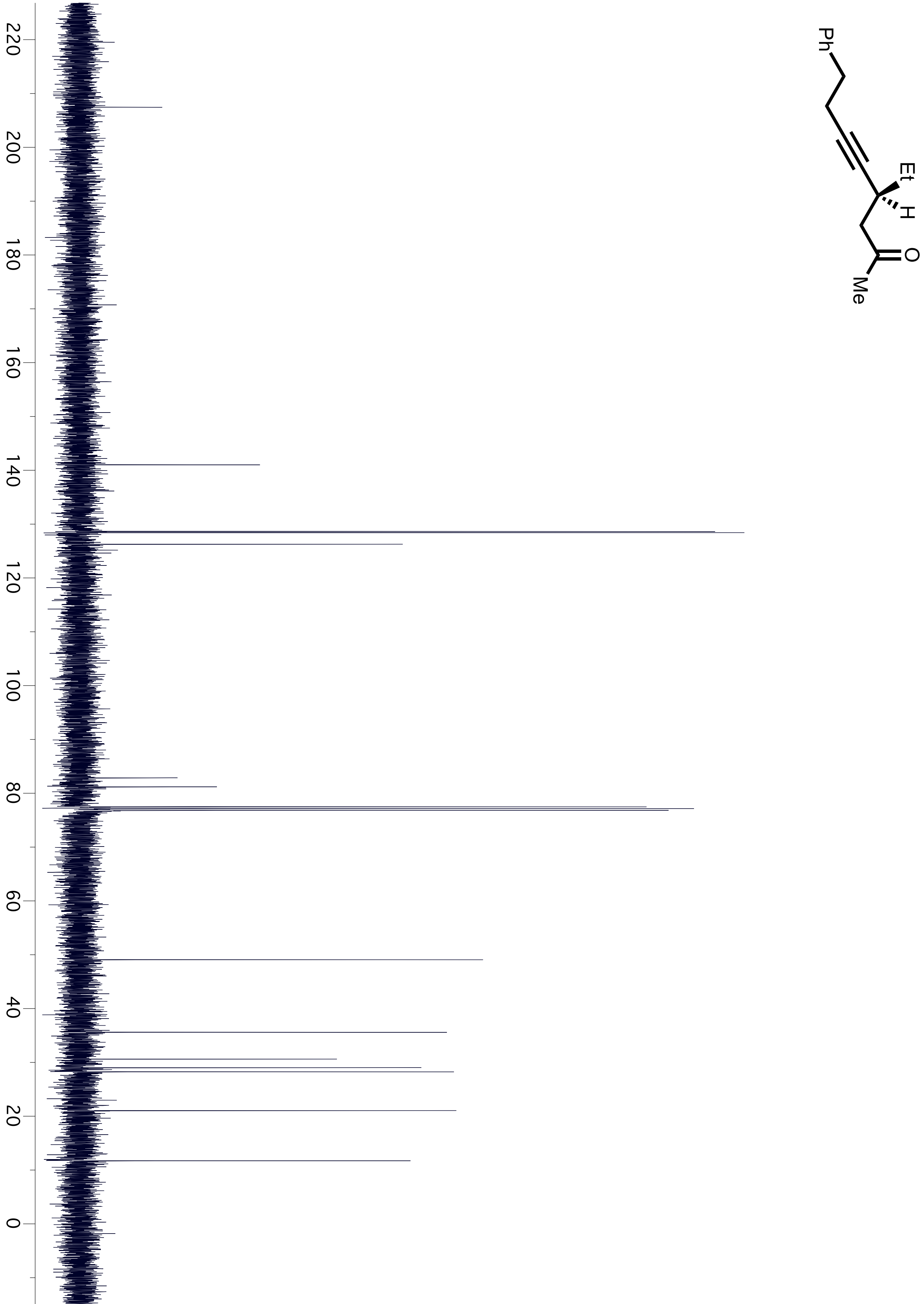
Ph
Et
H
O
Me
220
200
180
160
140
120
100
80
60
40
20
0

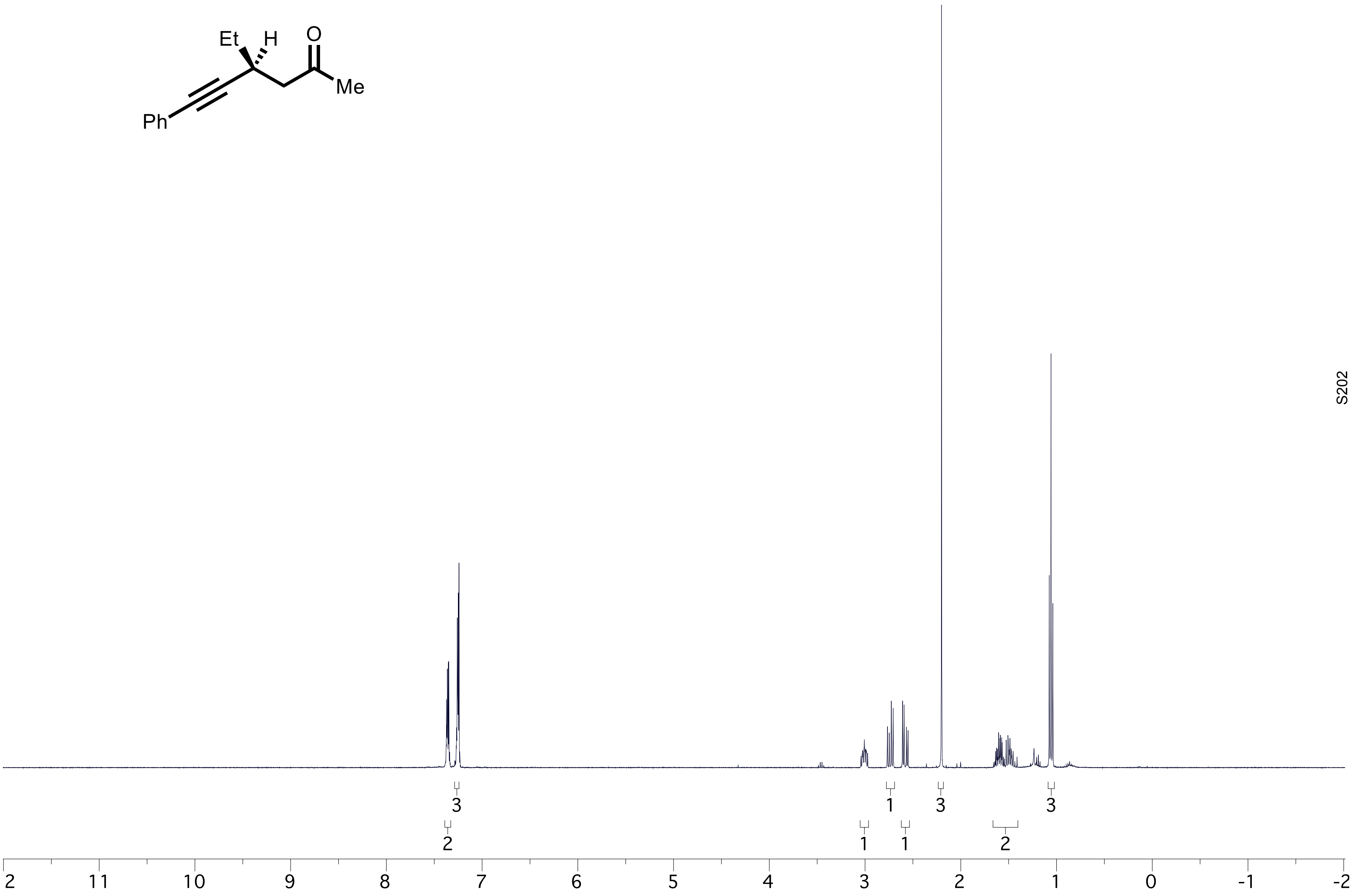
Et
H
O
Me
Ph
3
1
3
3
2
1
1
2
2
11
10
9
8
7
6
5
4
3
2
1
0
-1
-2

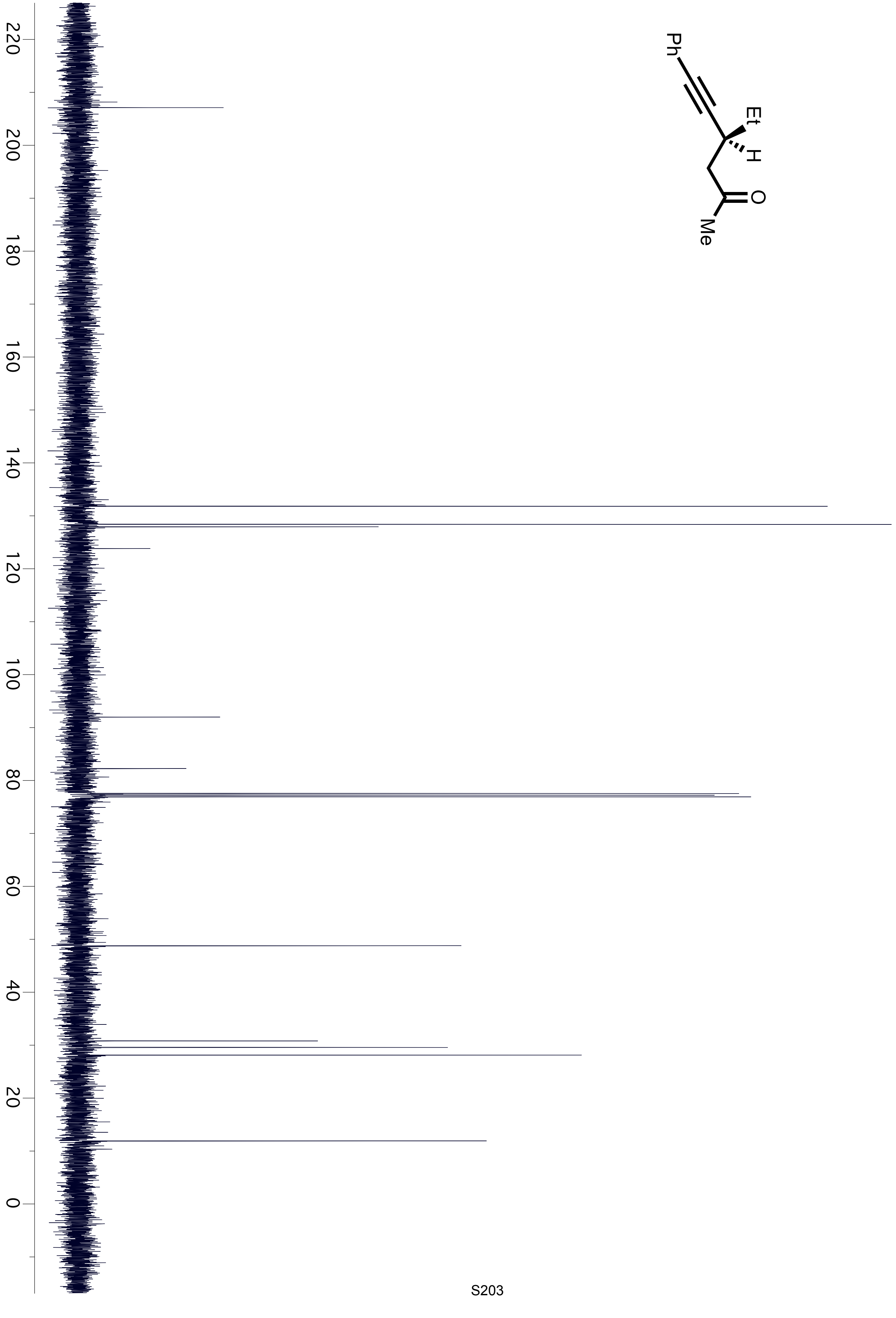
Ph
Et
H
O
Me
220
200
180
160
140
120
100
80
60
40
20
0

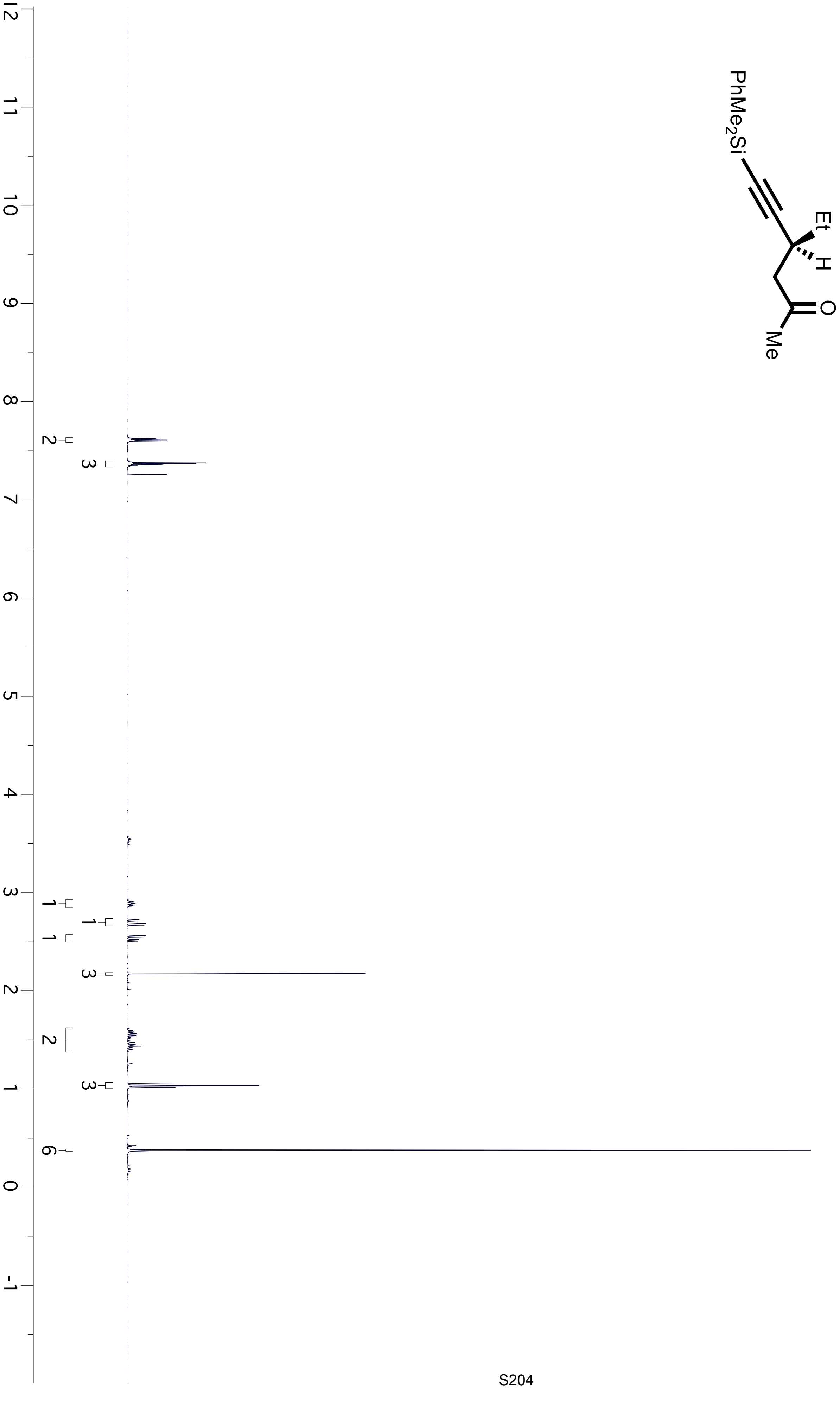
PhMe$_2$Si
Et
H
O
Me

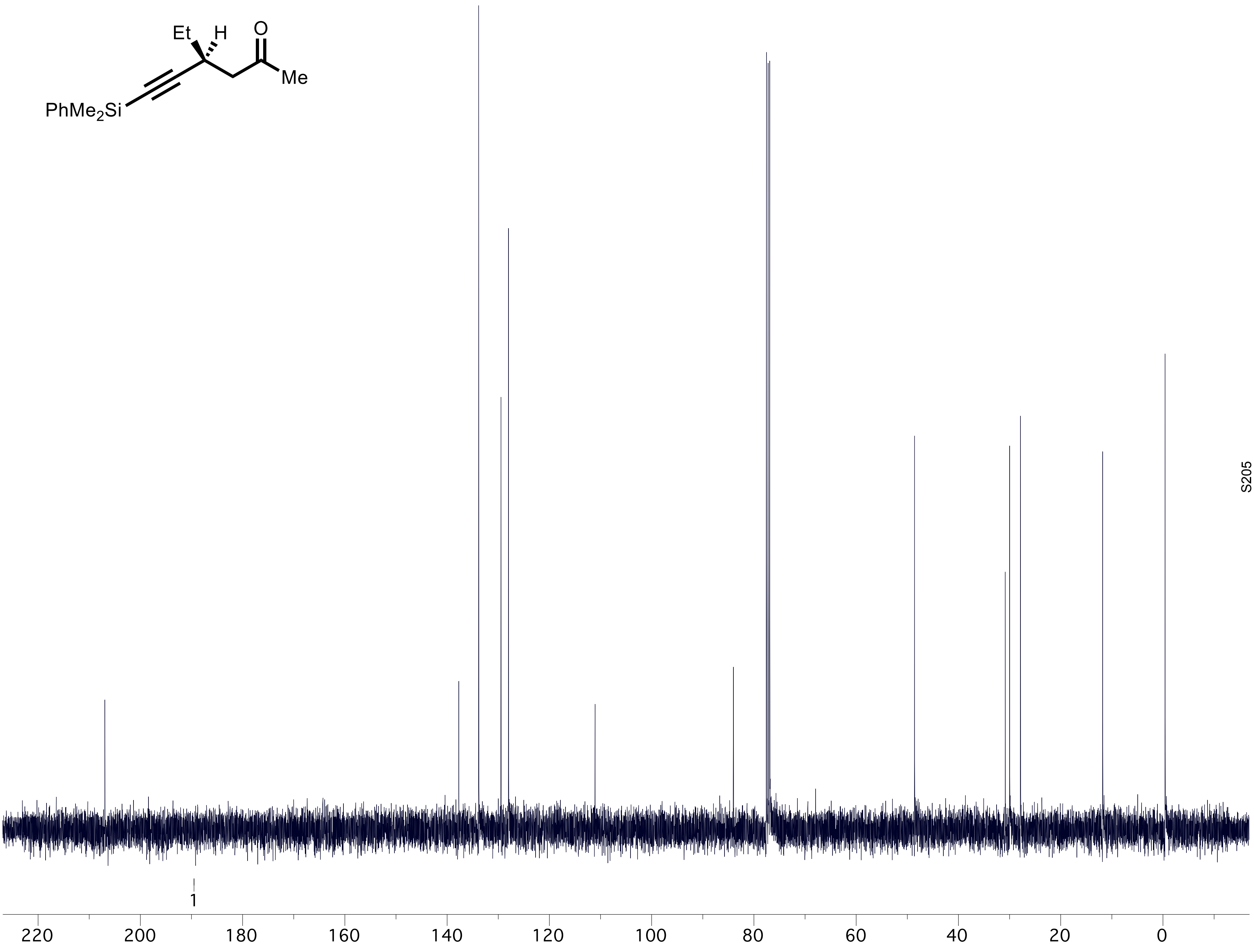
Et
H
O
Me
PhMe2Si
1
220
200
180
160
140
120
100
80
60
40
20
0

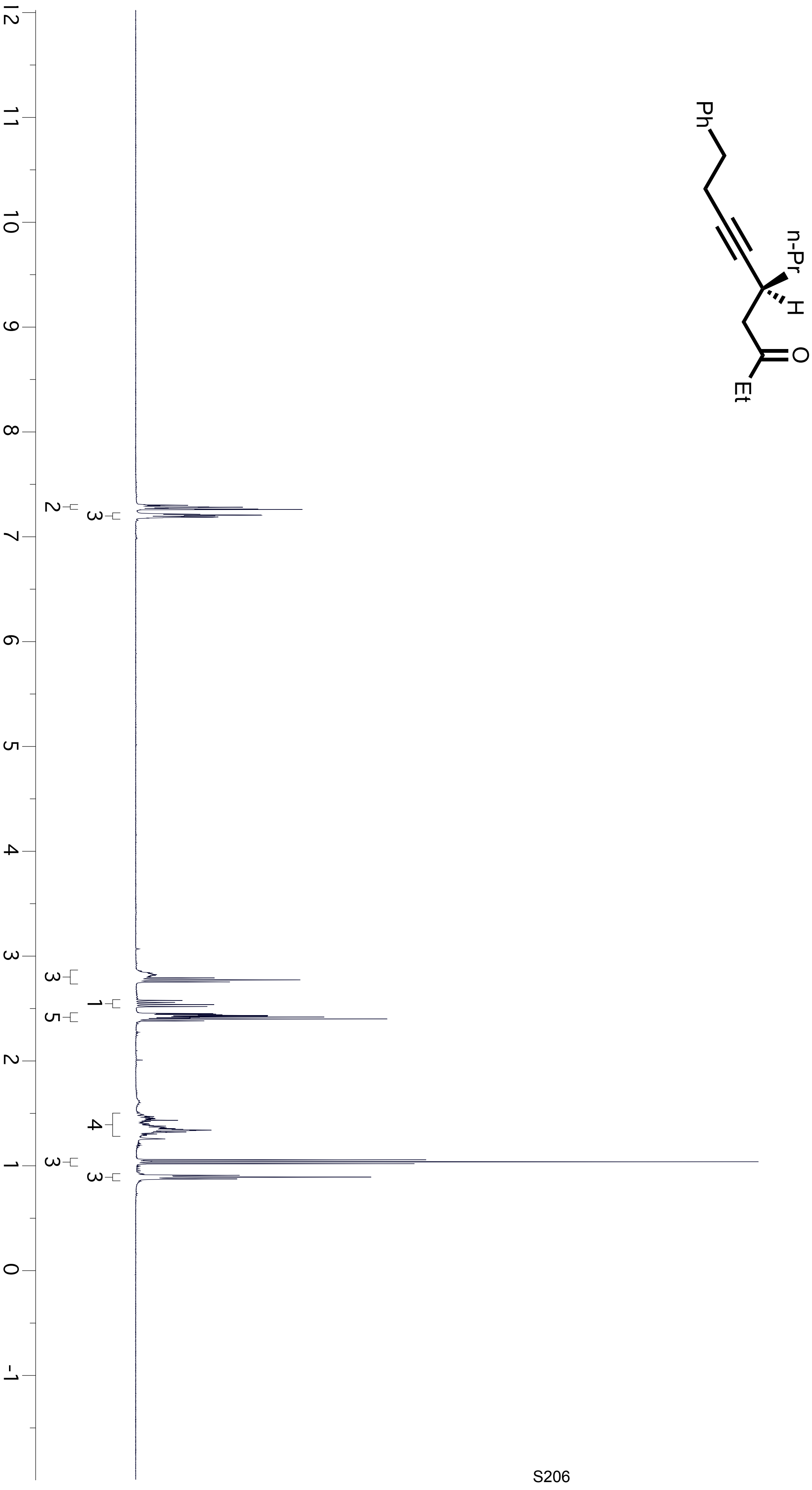

Ph
n-Pr
H
O
Et
12
11
10
9
8
7
6
5
4
3
2
1
0
-1
2
3
3
1
5
4
3
3

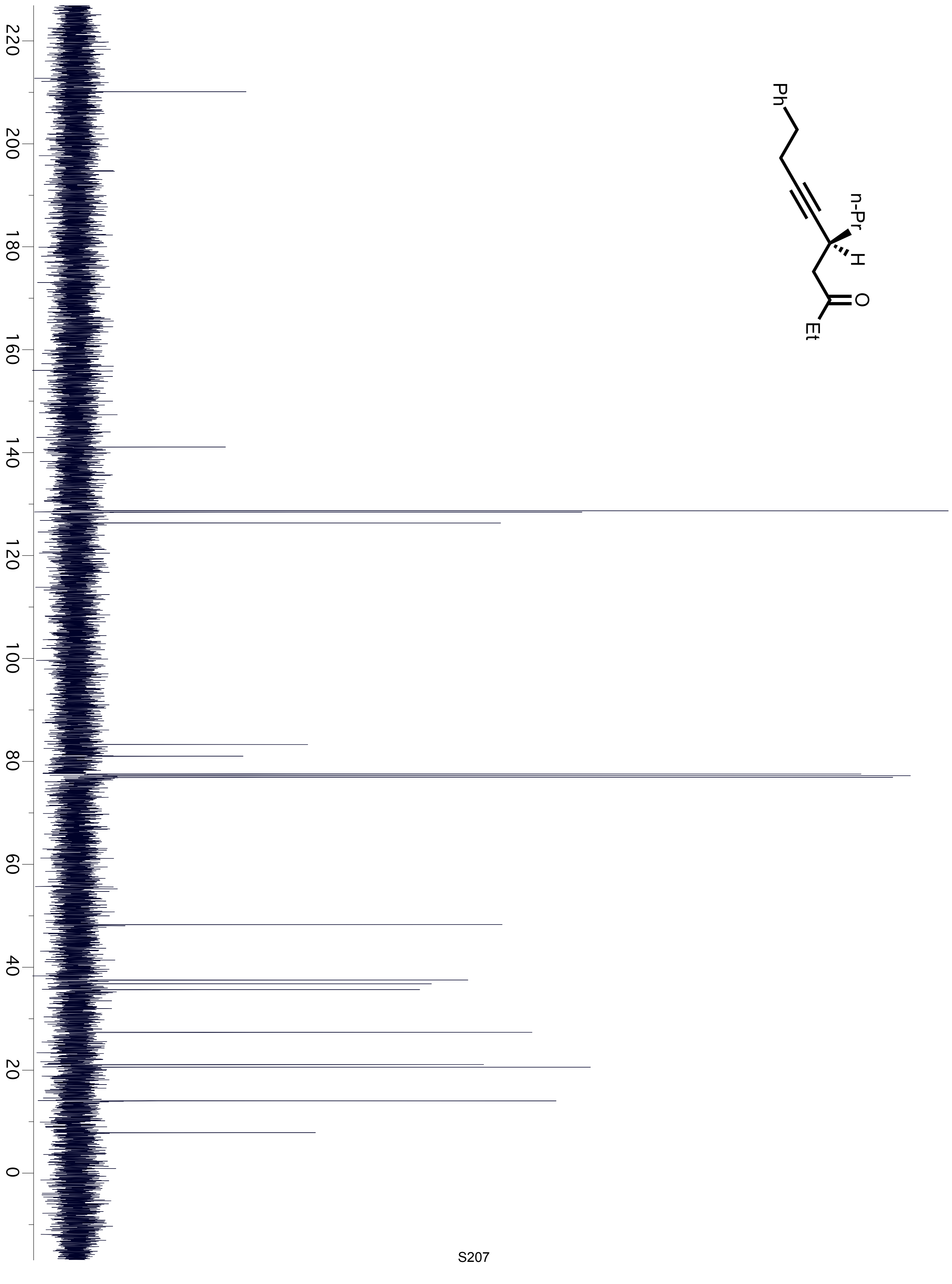

Ph
n-Pr
H
O
Et
220
200
180
160
140
120
100
80
60
40
20
0

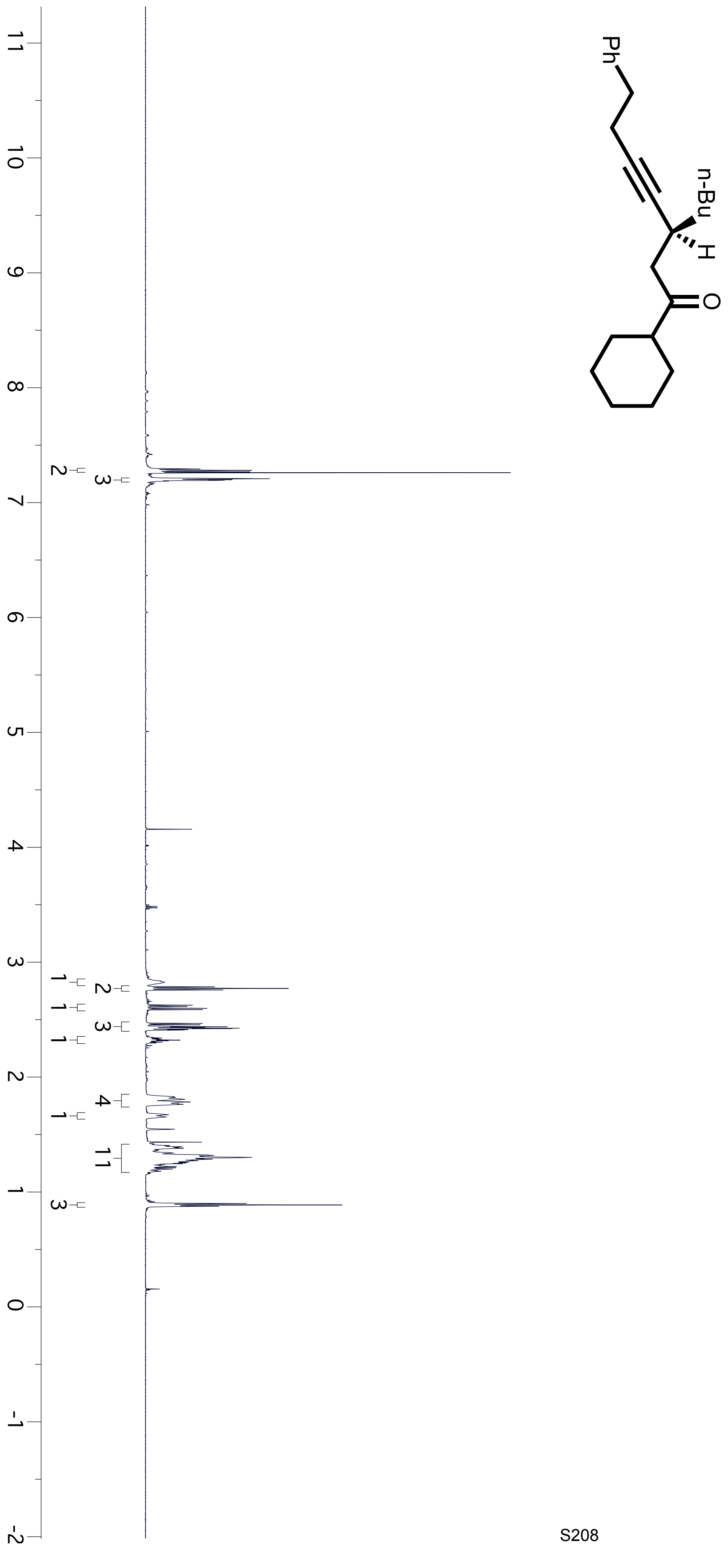

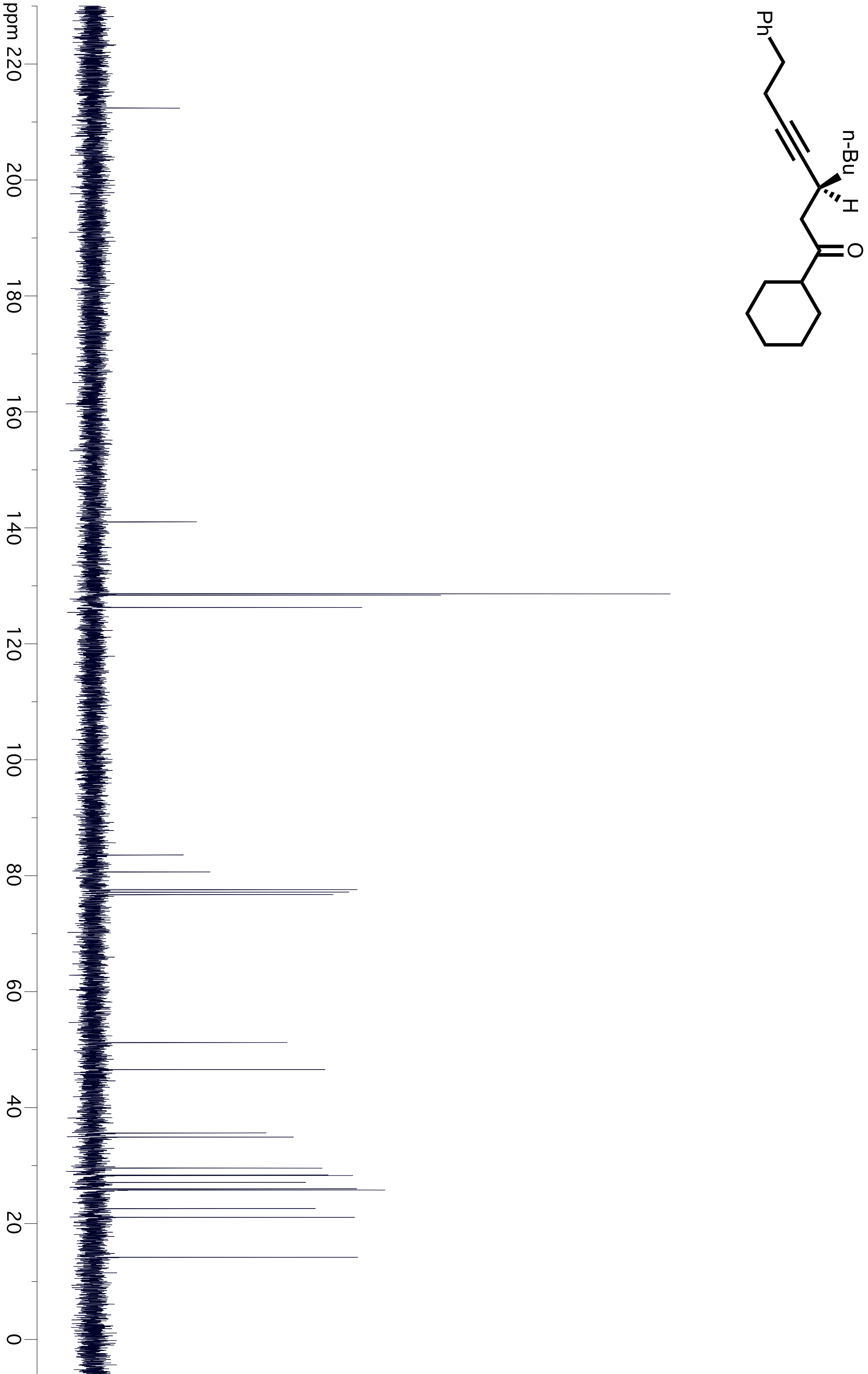
ppm 220
200
180
160
140
120
100
80
60
40
20
0
Ph
n-Bu
H
O

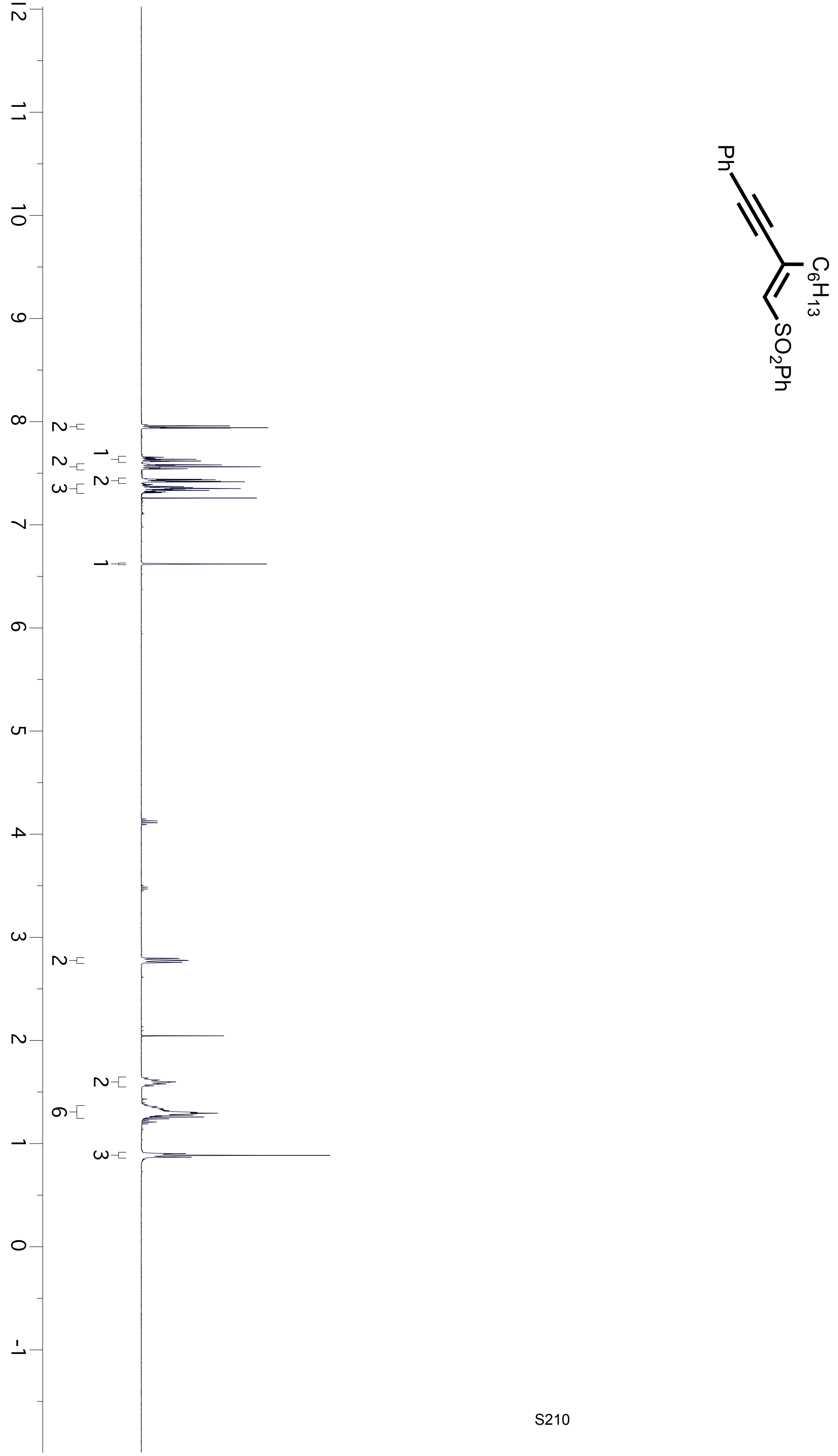
Ph
C6H13
SO2Ph

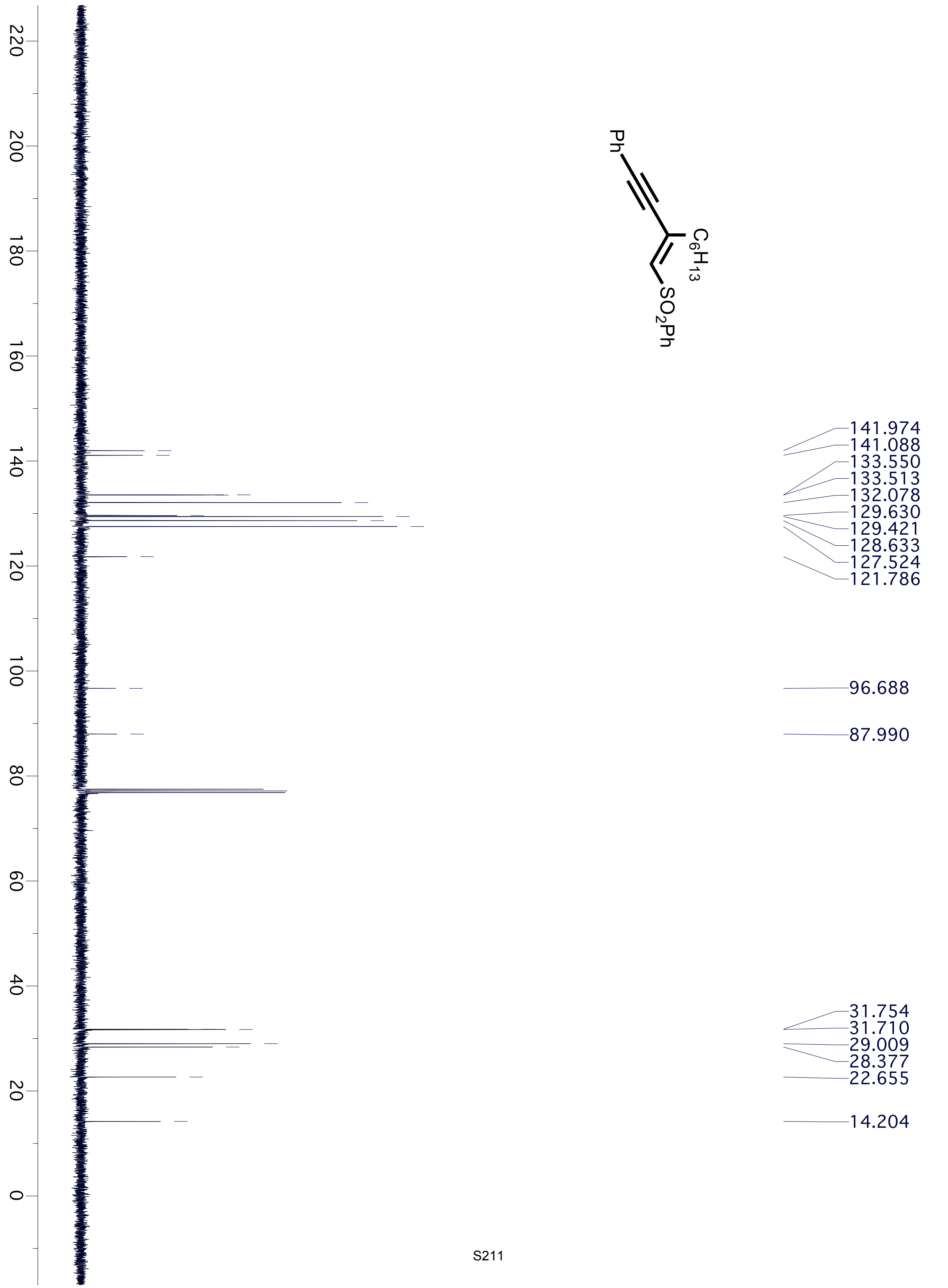
Ph
C_6H_{13}
SO_2Ph
141.974
141.088
133.550
133.513
132.078
129.630
129.421
128.633
127.524
121.786
96.688
87.990
31.754
31.710
29.009
28.377
22.655
14.204
220
200
180
160
140
120
100
80
60
40
20
0

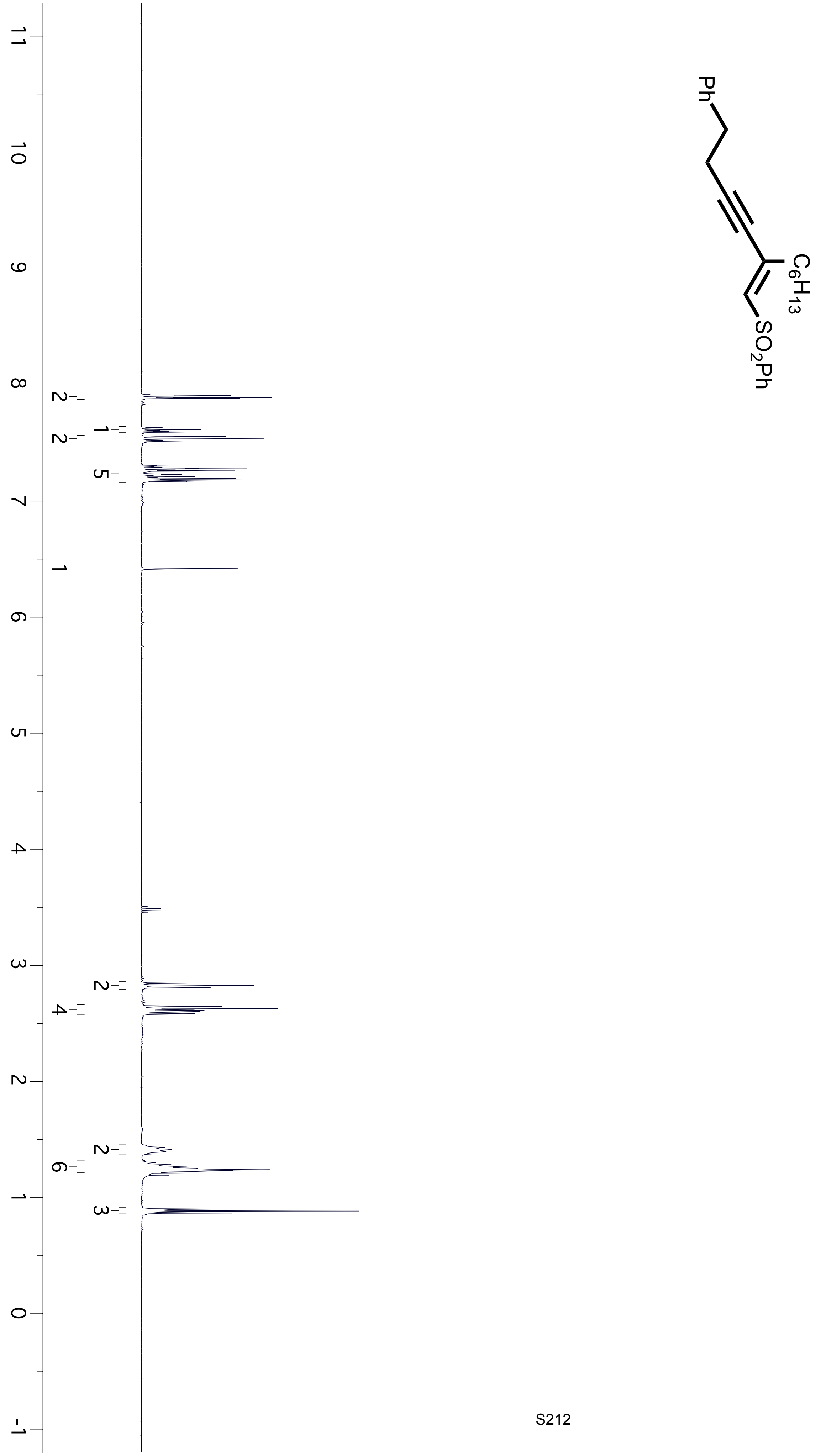
Ph
C6H13
SO2Ph
11
10
9
8
7
6
5
4
3
2
1
0
-1

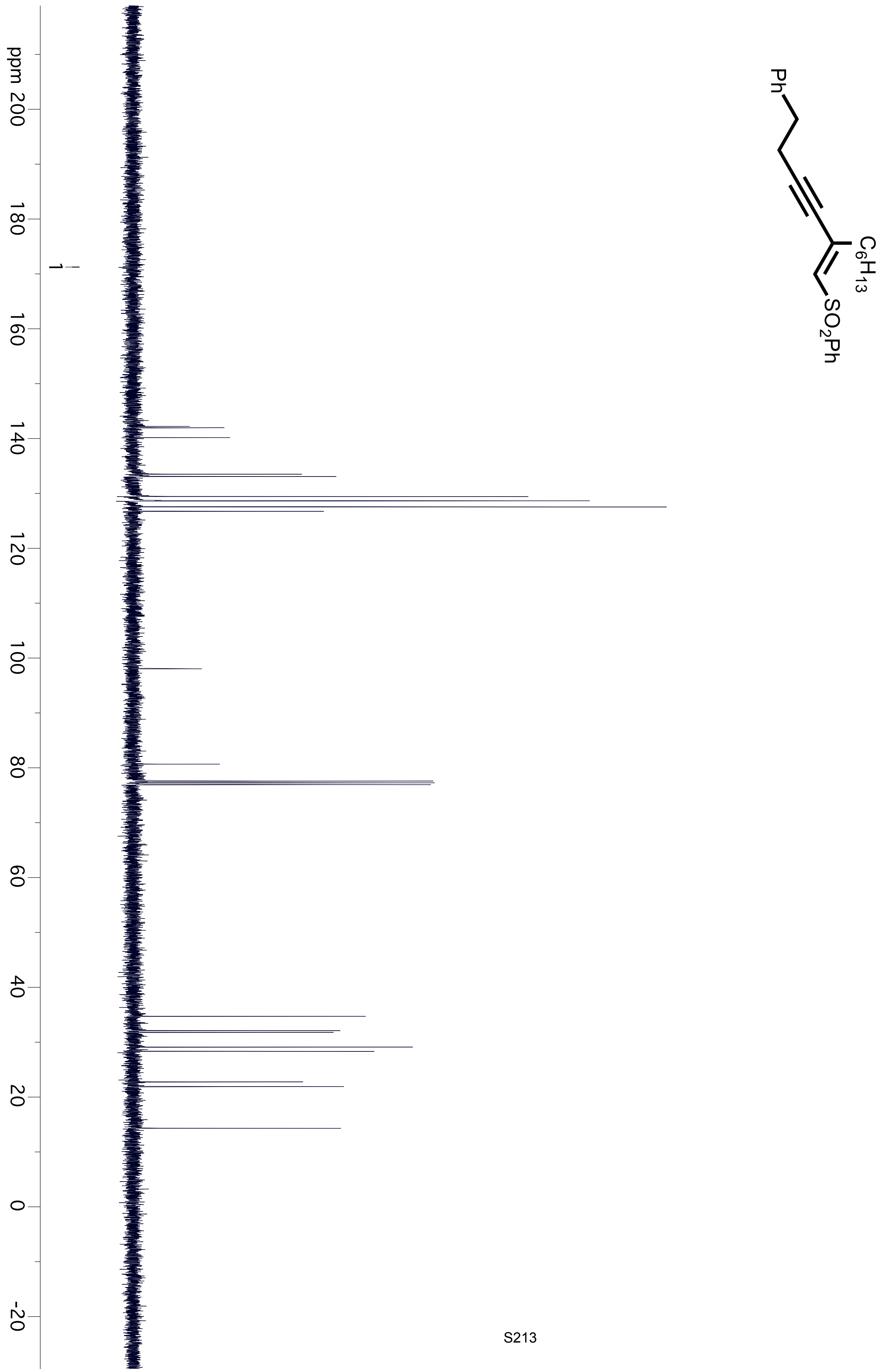
Ph
C6H13
SO2Ph
ppm 200
180
160
140
120
100
80
60
40
20
0
-20

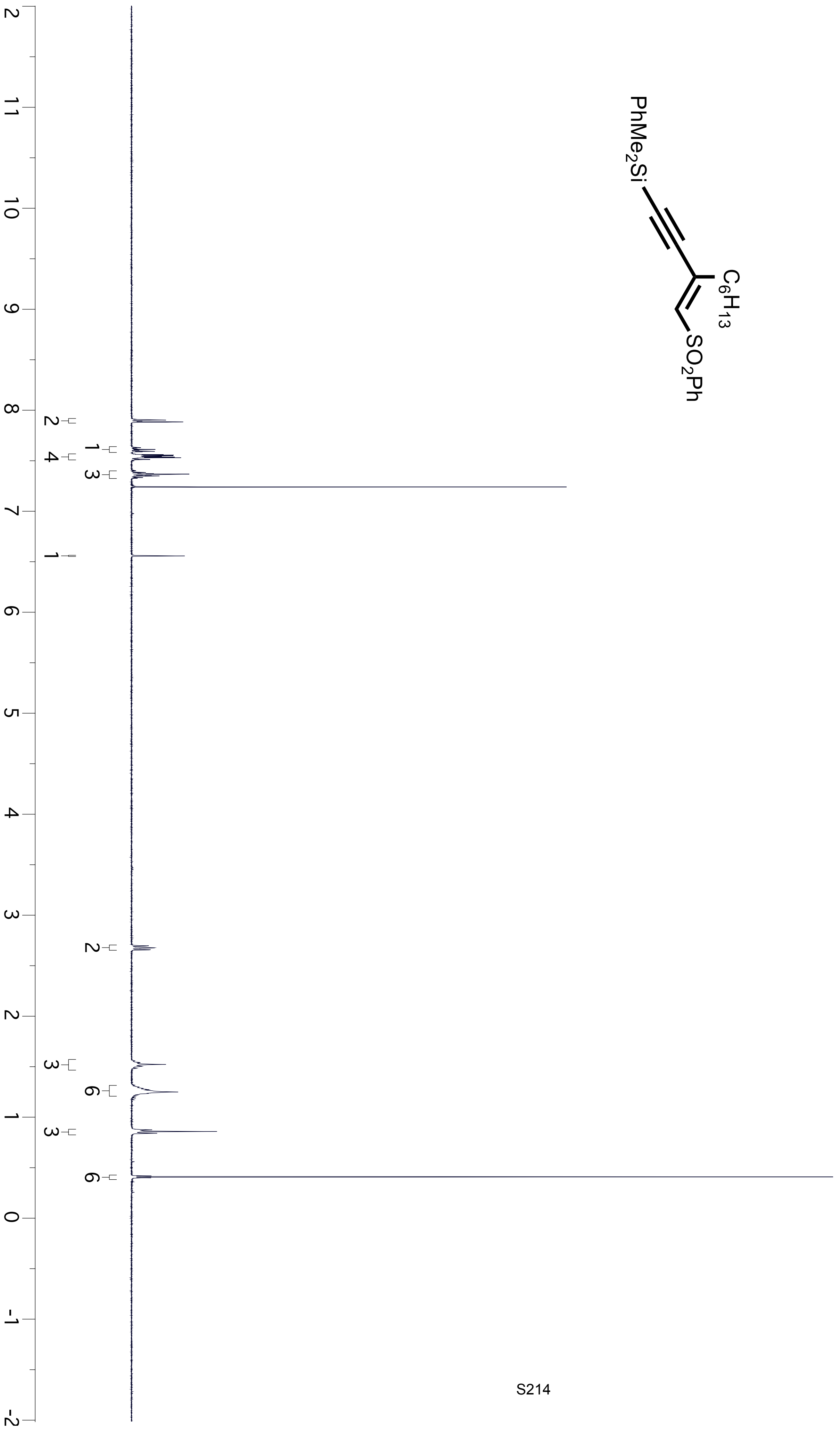
PhMe2Si
C6H13
SO2Ph
12
11
10
9
8
7
6
5
4
3
2
1
0
-1
-2
2
1
4
3
1
2
3
6
3
6

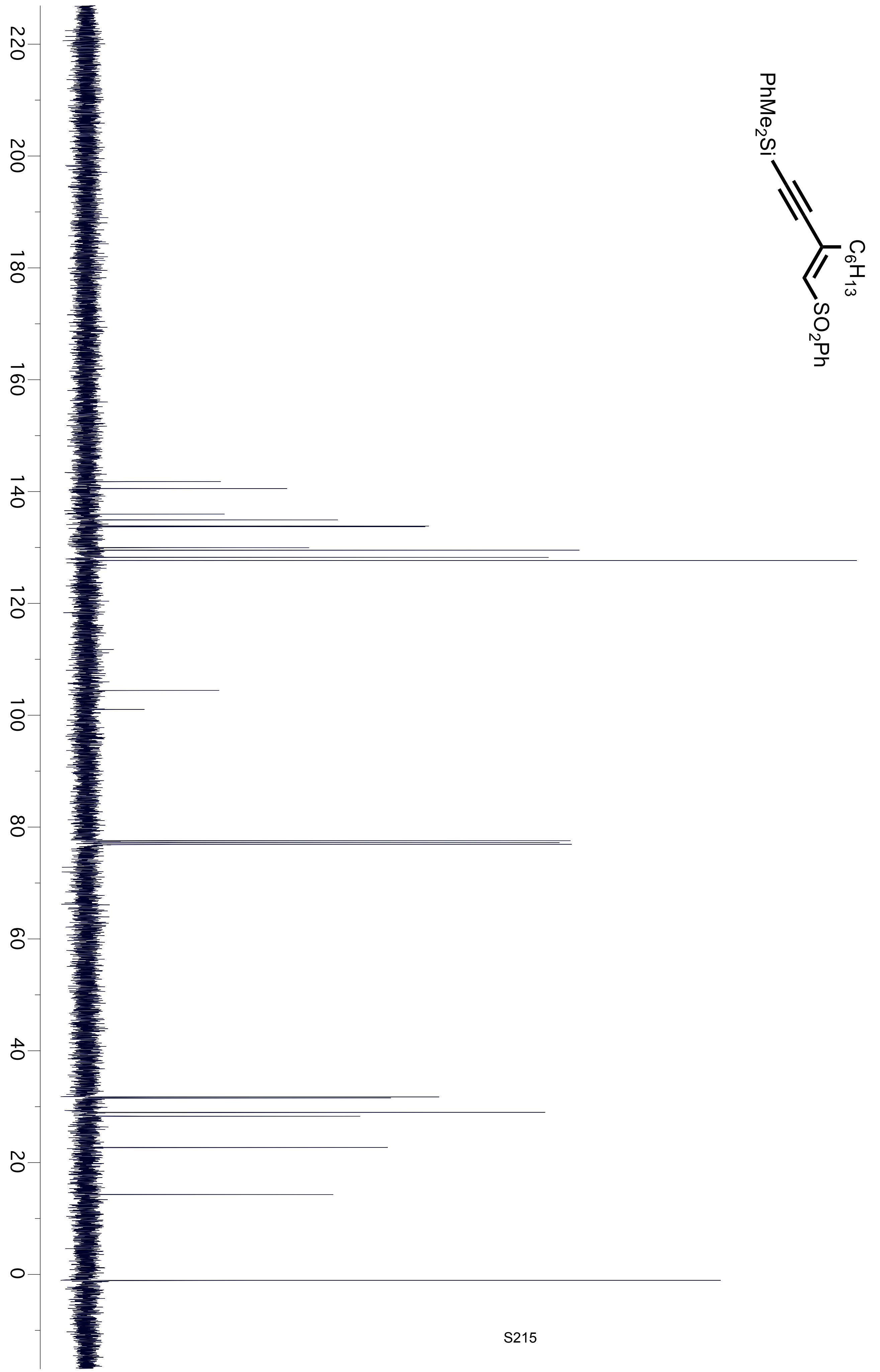
PhMe$_2$Si
C$_6$H$_{13}$
SO$_2$Ph
220
200
180
160
140
120
100
80
60
40
20
0

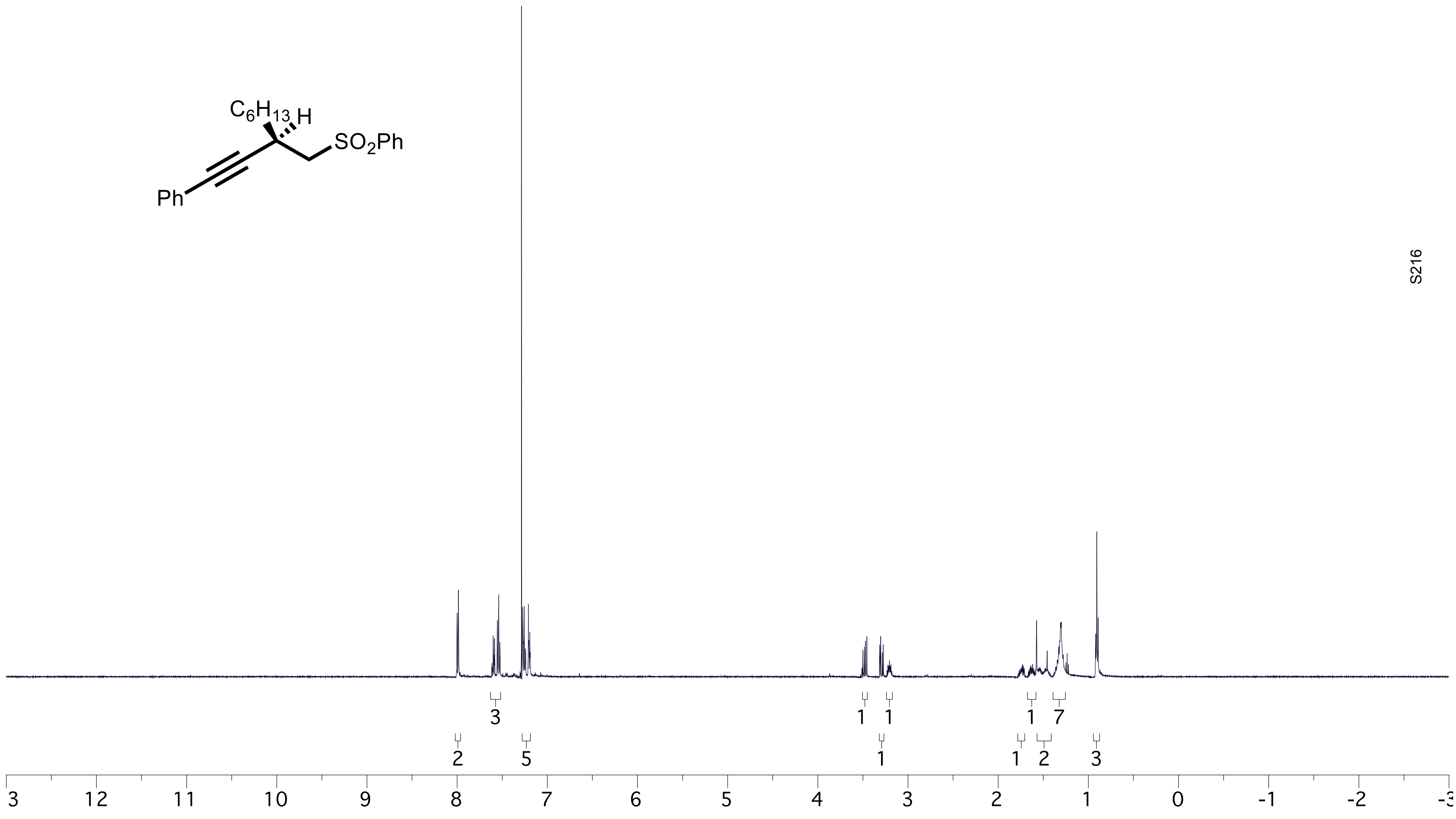

C6H13
H
SO2Ph
Ph
3 1 1 1 7
2 5 1 1 2 3
3 12 11 10 9 8 7 6 5 4 3 2 1 0 -1 -2 -3

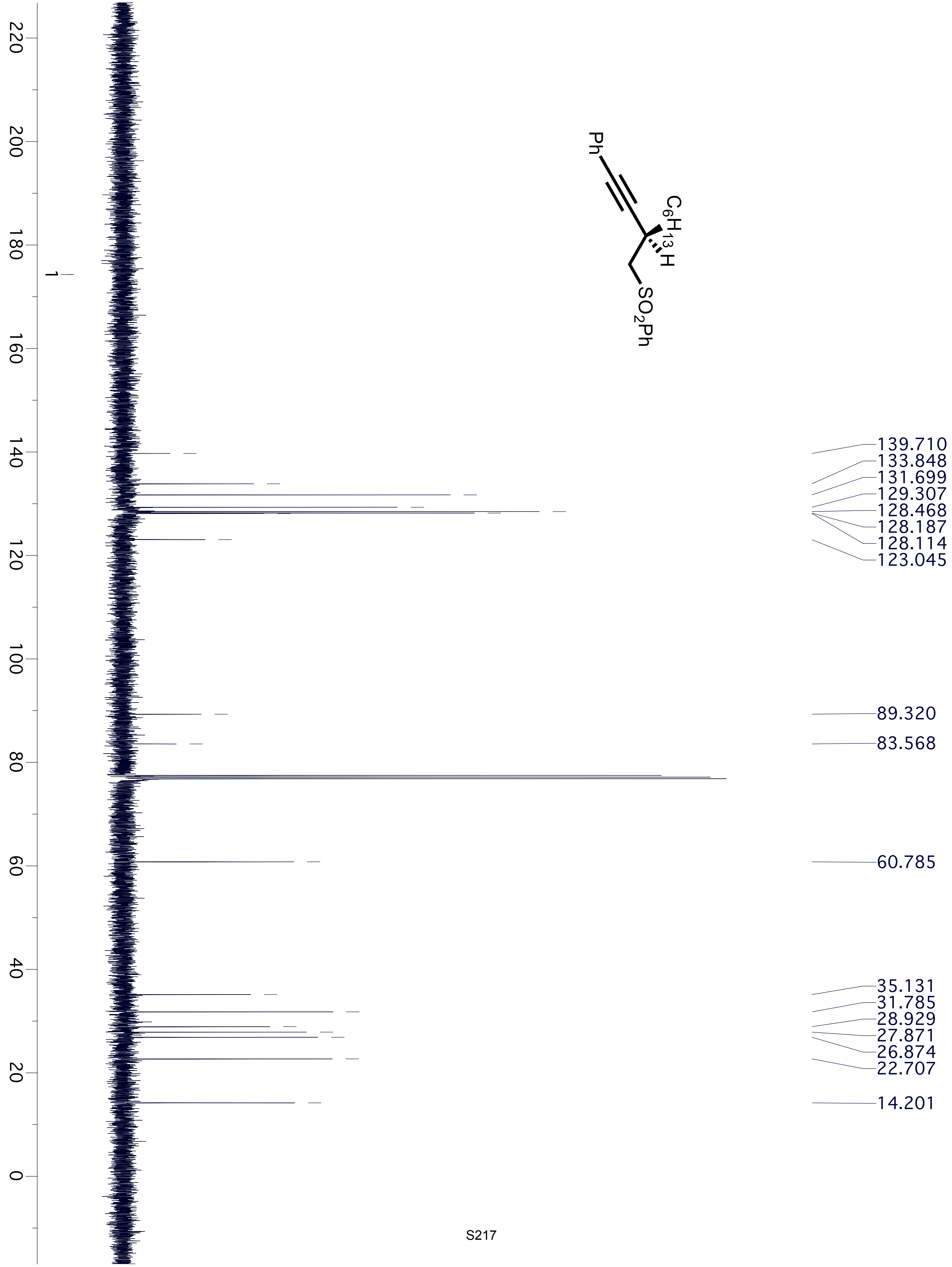

139.710
133.848
131.699
129.307
128.468
128.187
128.114
123.045
89.320
83.568
60.785
35.131
31.785
28.929
27.871
26.874
22.707
14.201

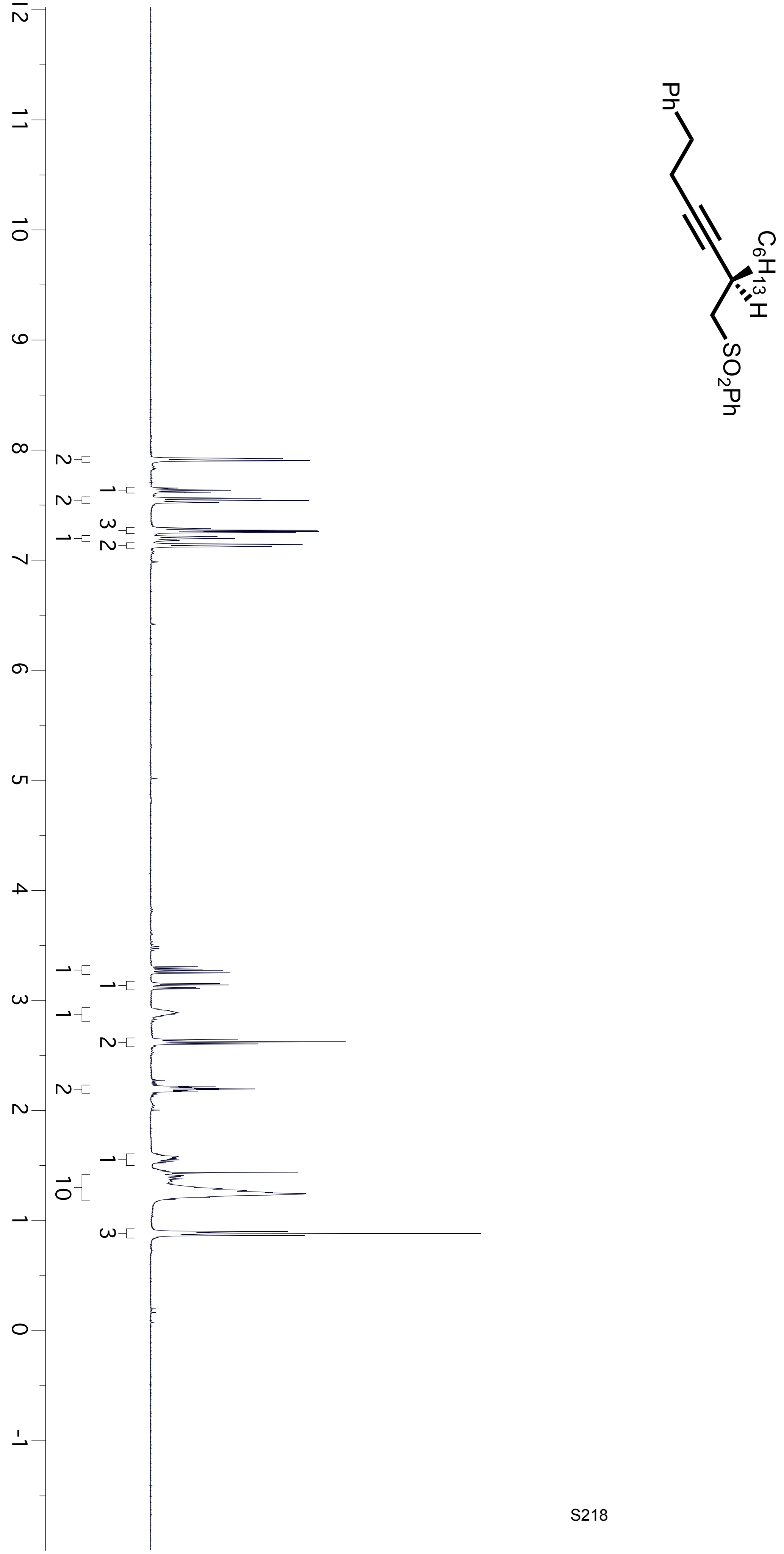
Ph
C6H13
H
SO2Ph

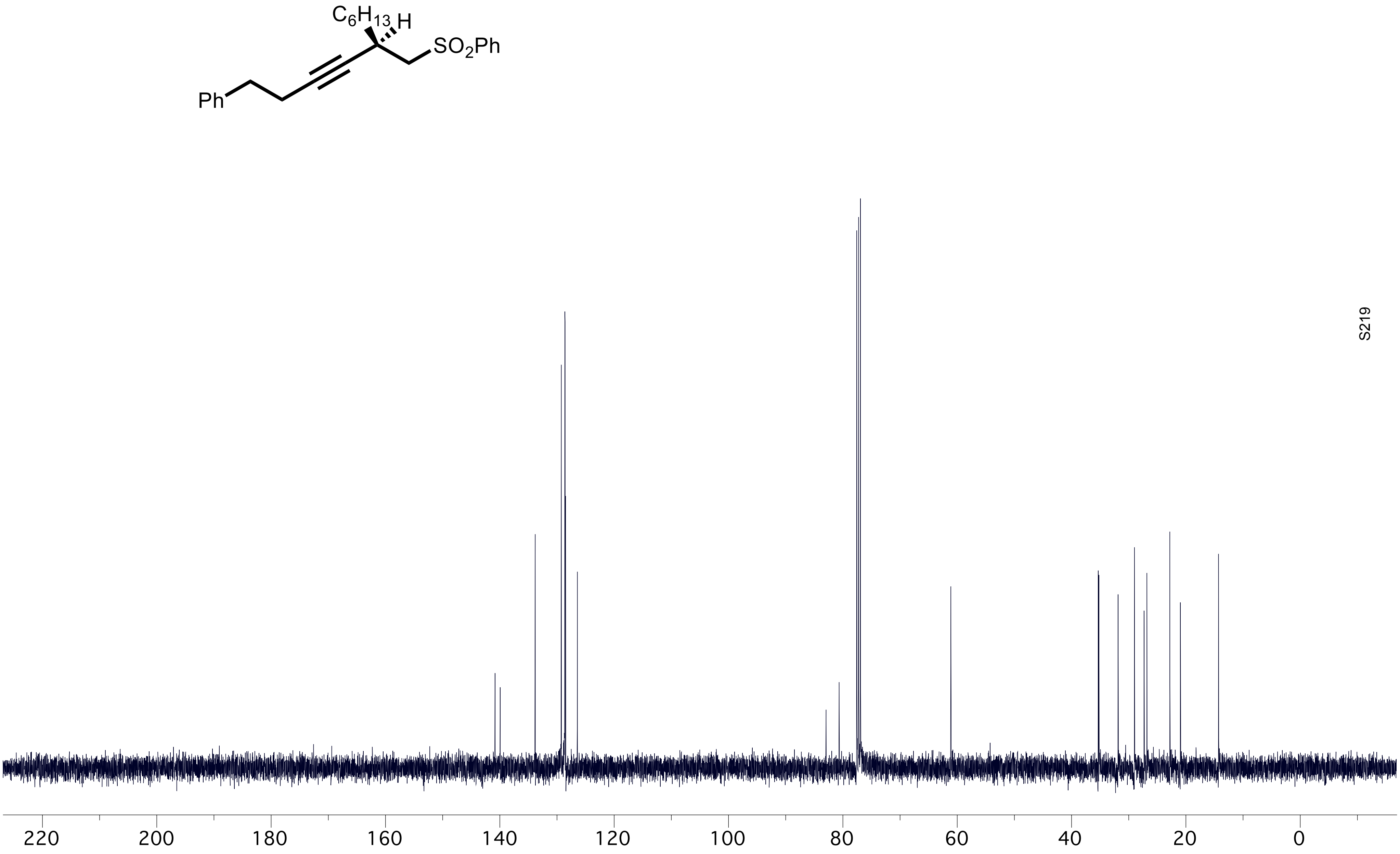
C6H13 H
SO2Ph
Ph
220 200 180 160 140 120 100 80 60 40 20 0

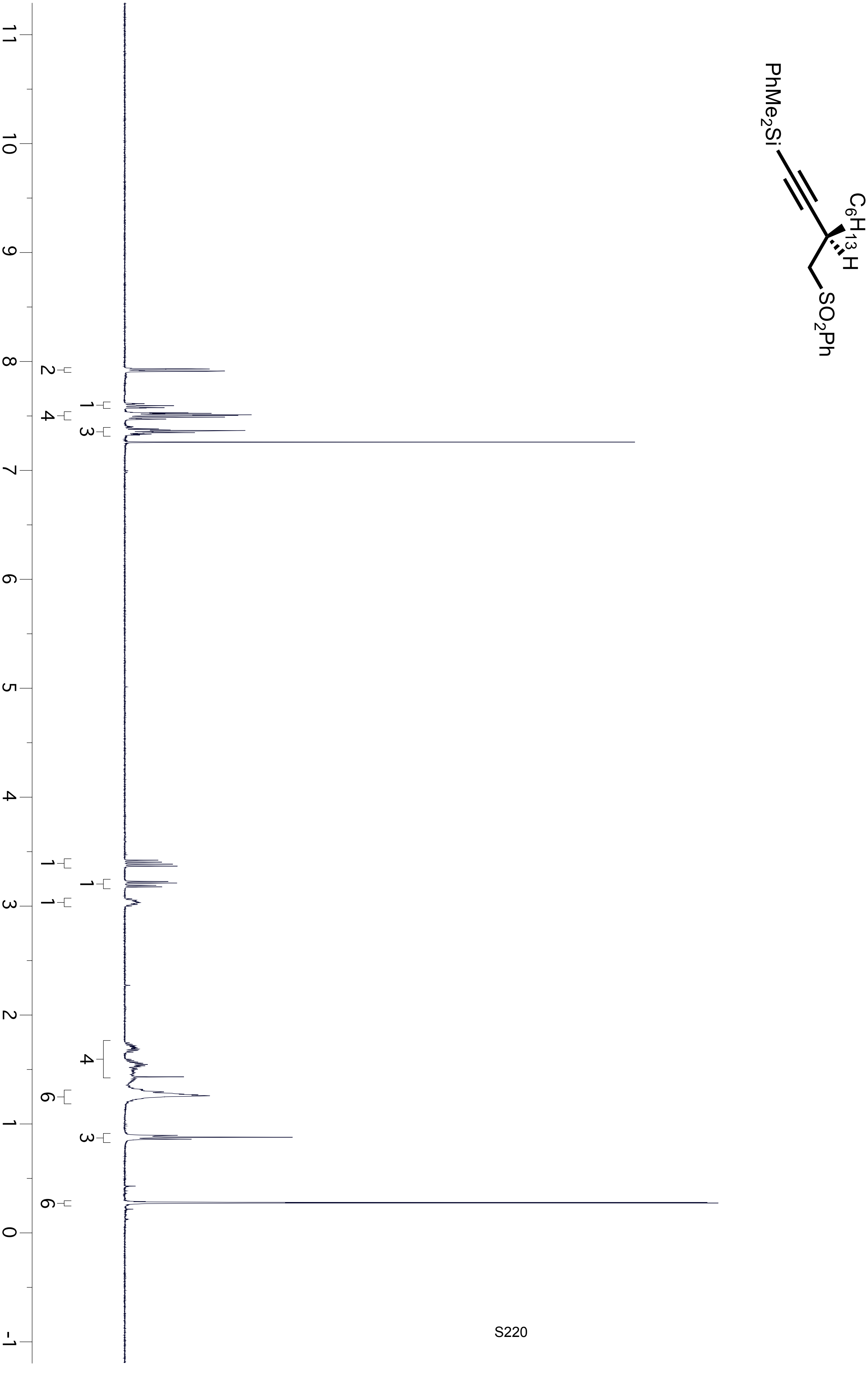
PhMe$_2$Si
C_6H_{13}
H
SO_2Ph
11
10
9
8
7
6
5
4
3
2
1
0
-1
2
1
4
3
1
1
1
4
6
3
6

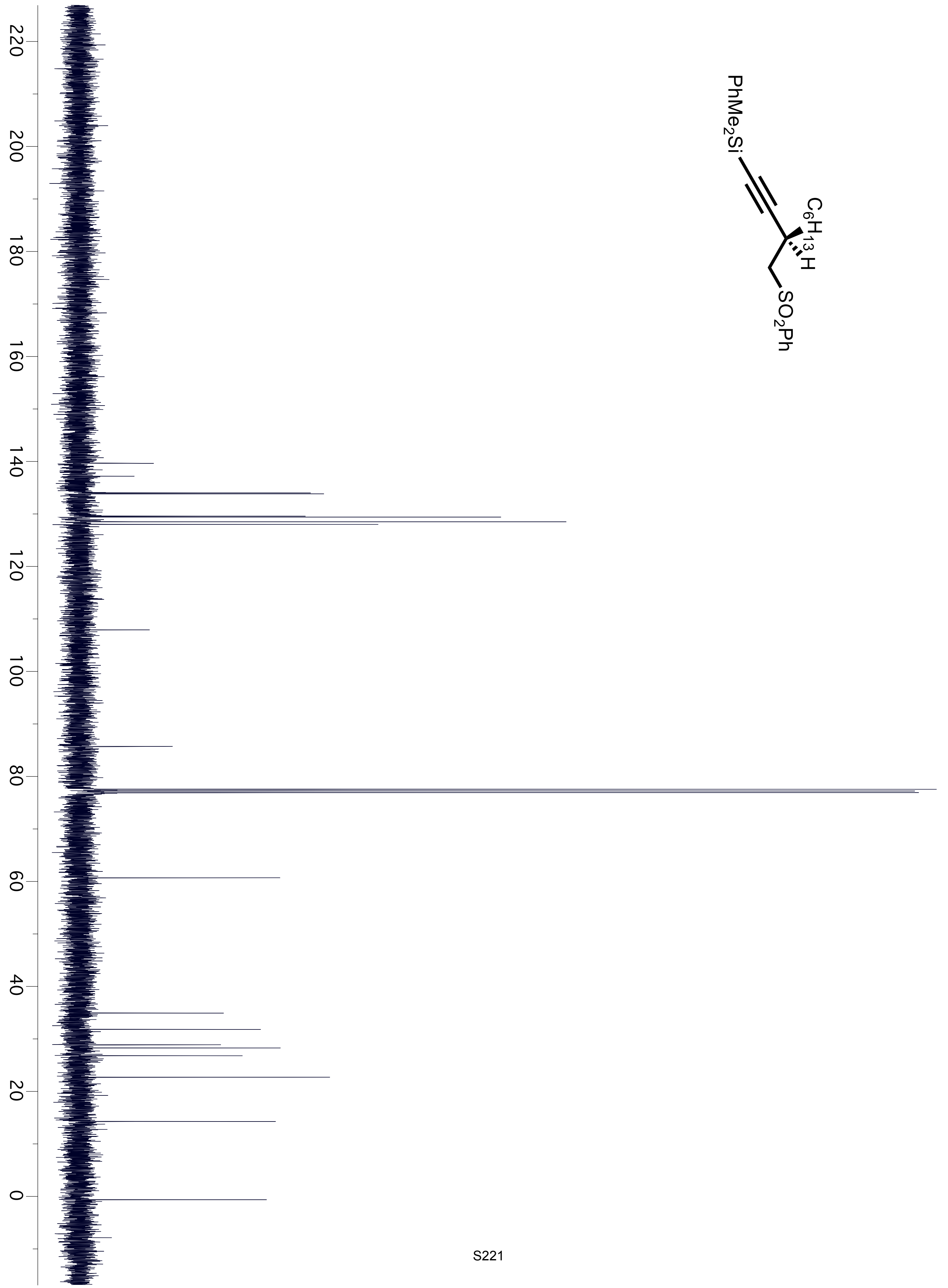
PhMe2Si
C6H13
H
SO2Ph
220
200
180
160
140
120
100
80
60
40
20
0

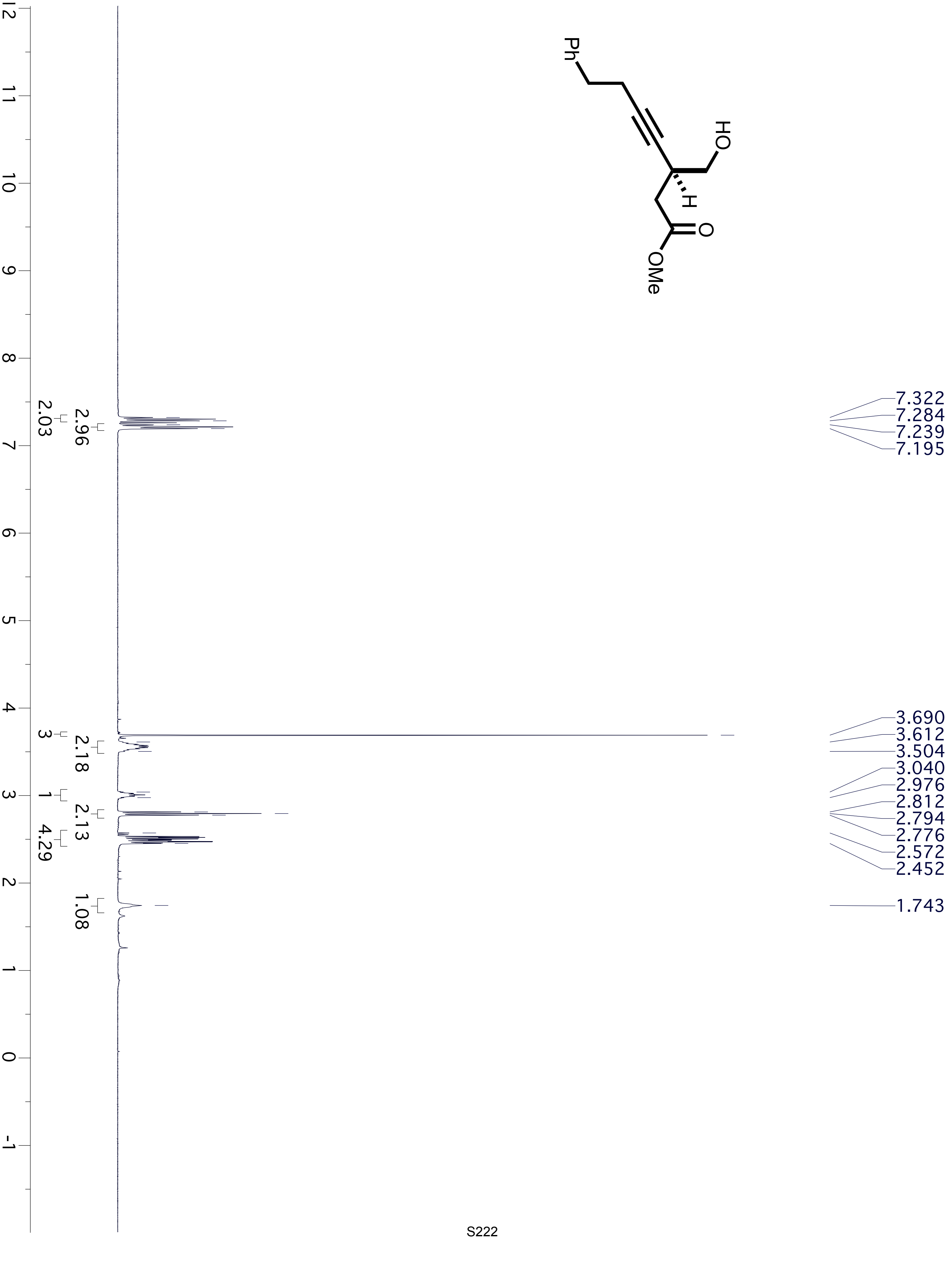
Ph
HO
H
O
OMe
7.322
7.284
7.239
7.195
3.690
3.612
3.504
3.040
2.976
2.812
2.794
2.776
2.572
2.452
1.743
2.03
2.96
3
2.18
1
2.13
4.29
1.08

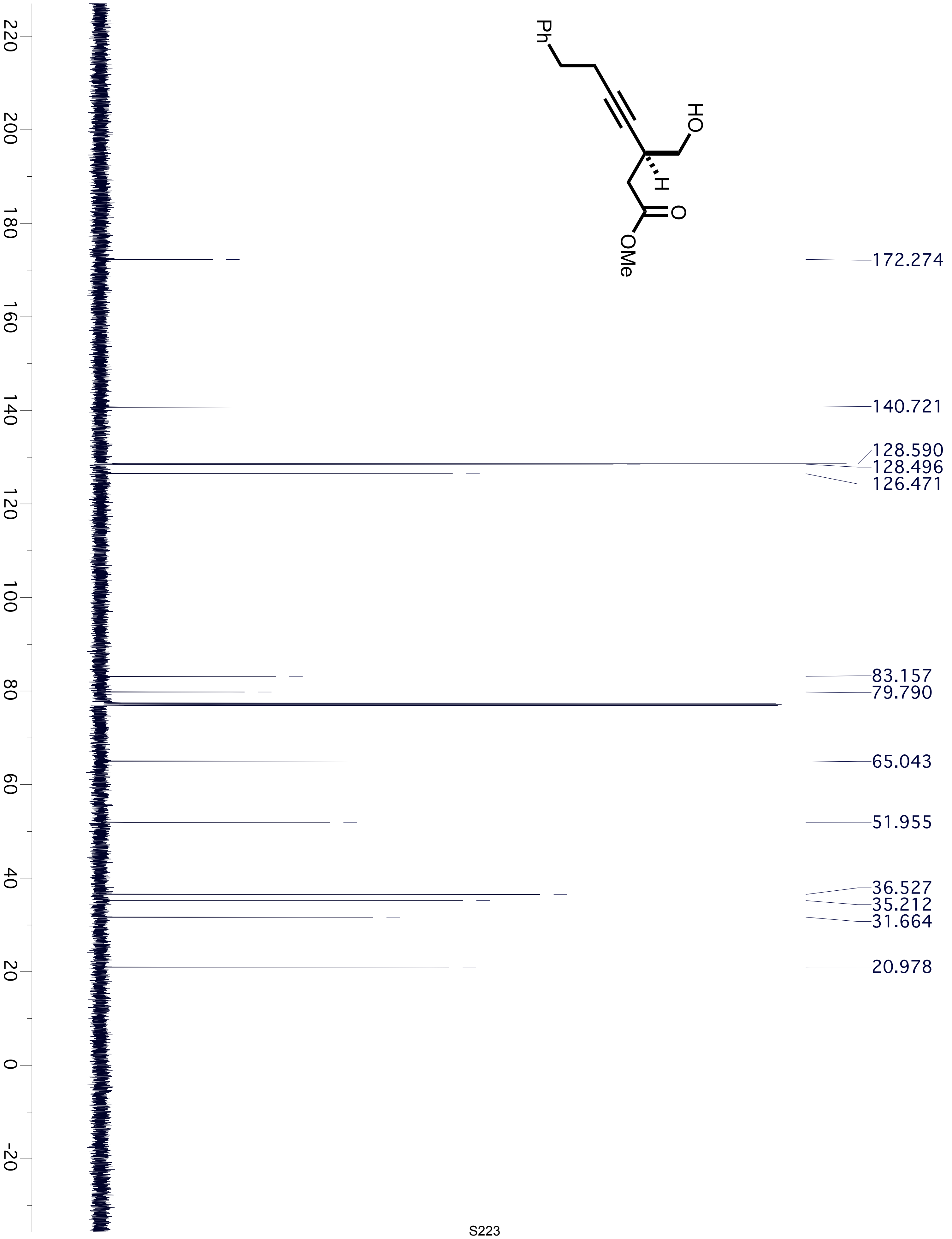
Ph
HO
H
O
OMe
172.274
140.721
128.590
128.496
126.471
83.157
79.790
65.043
51.955
36.527
35.212
31.664
20.978
220
200
180
160
140
120
100
80
60
40
20
0
-20

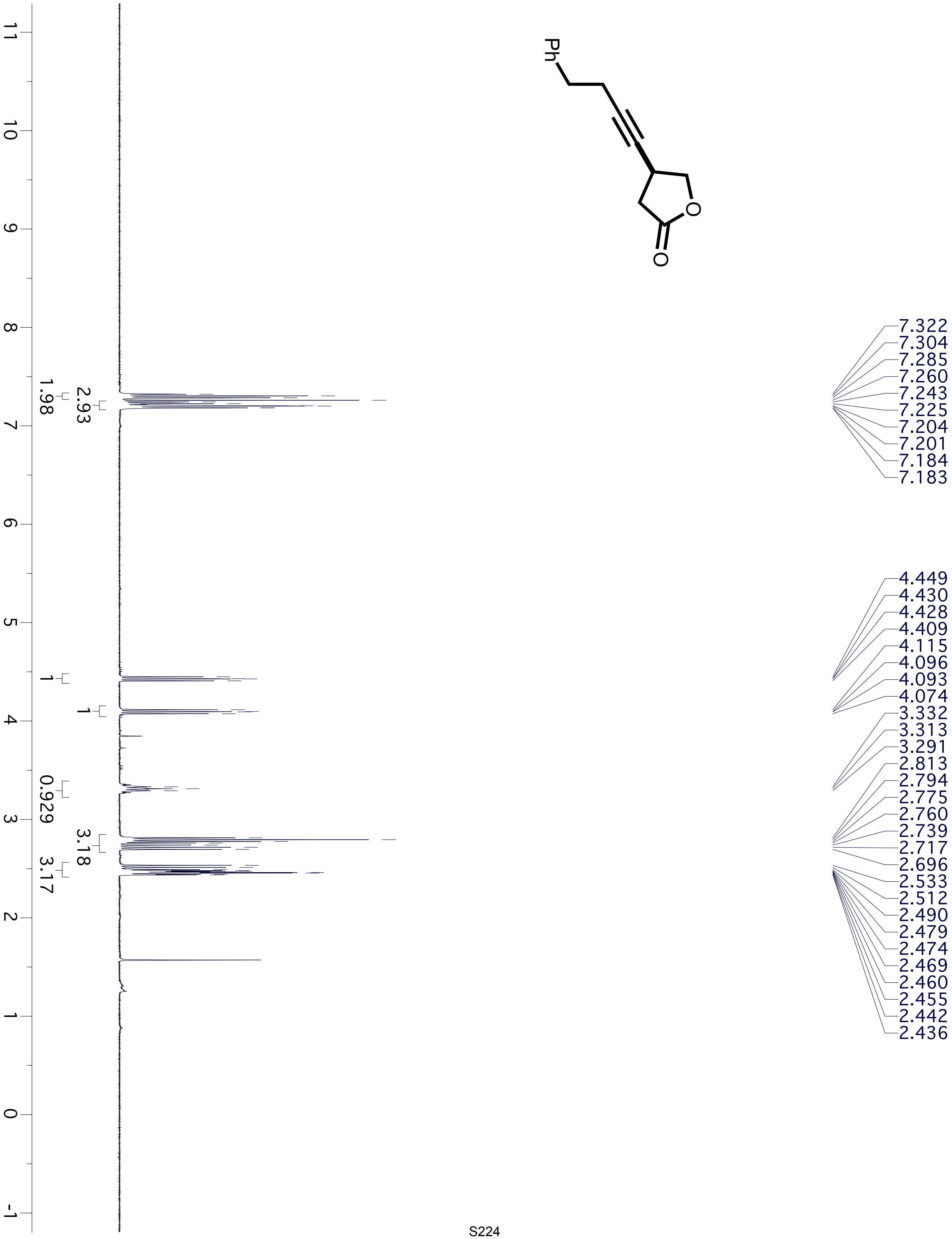
Ph
O
O
7.322
7.304
7.285
7.260
7.243
7.225
7.204
7.201
7.184
7.183
4.449
4.430
4.428
4.409
4.115
4.096
4.093
4.074
3.332
3.313
3.291
2.813
2.794
2.775
2.760
2.739
2.717
2.696
2.533
2.512
2.490
2.479
2.474
2.469
2.460
2.455
2.442
2.436
1.98
2.93
1
1
0.929
3.18
3.17
11
10
9
8
7
6
5
4
3
2
1
0
-1

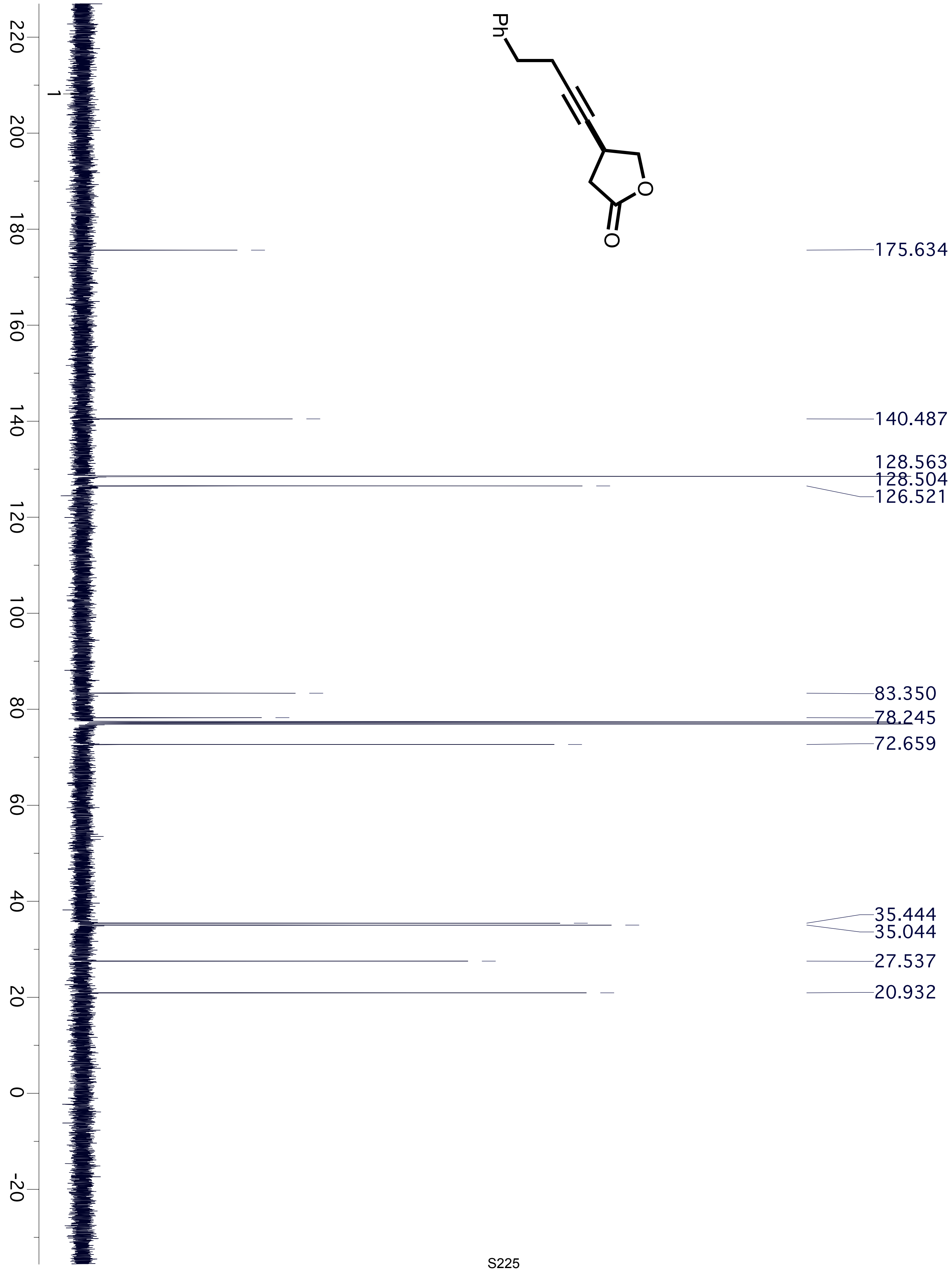
175.634
140.487
128.563
128.504
126.521
83.350
78.245
72.659
35.444
35.044
27.537
20.932
Ph
O
O
1

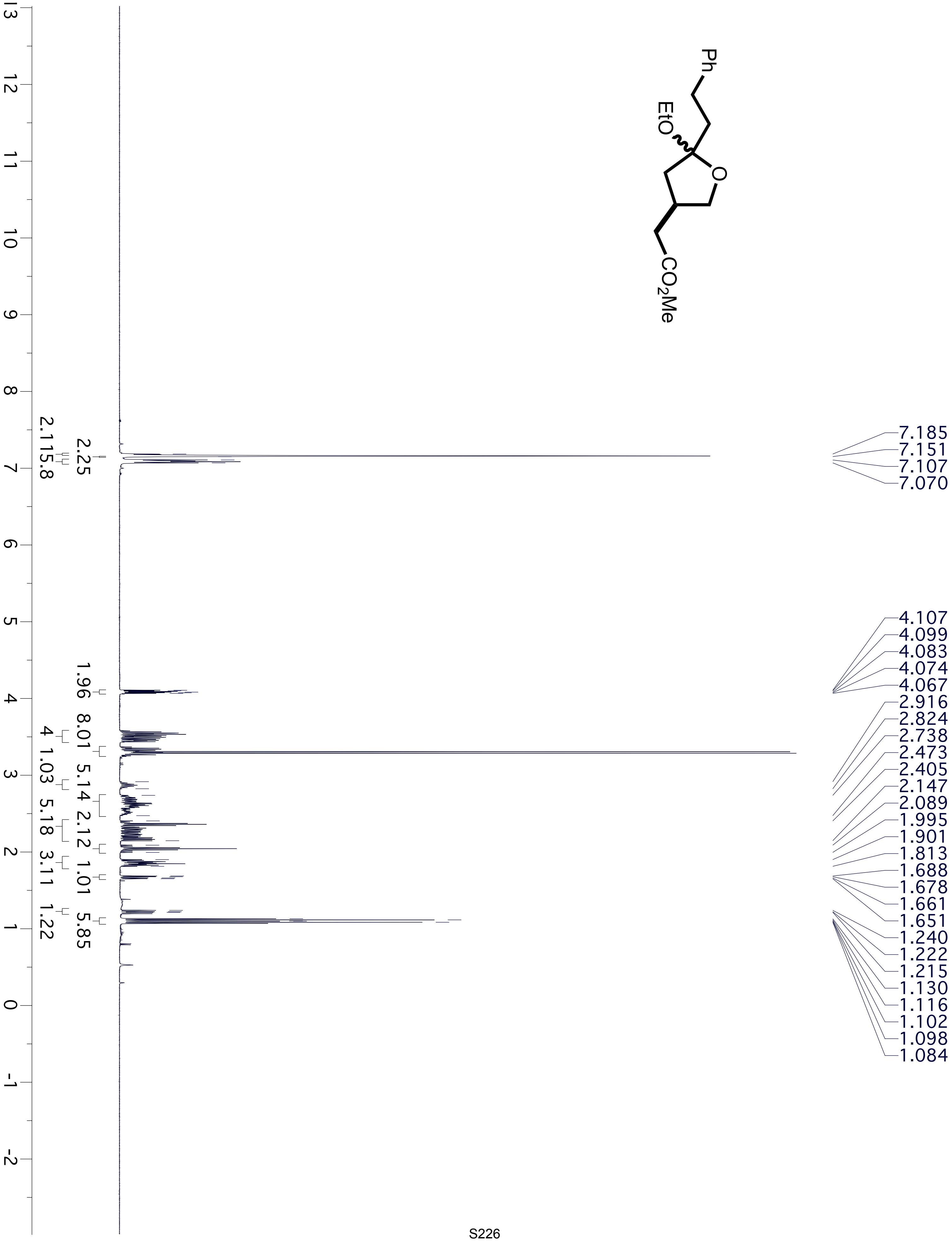
Ph
EtO
O
CO2Me
7.185
7.151
7.107
7.070
4.107
4.099
4.083
4.074
4.067
2.916
2.824
2.738
2.473
2.405
2.147
2.089
1.995
1.901
1.813
1.688
1.678
1.661
1.651
1.240
1.222
1.215
1.130
1.116
1.102
1.098
1.084
2.25
2.11
5.8
1.96
8.01
4
5.14
1.03
2.12
5.18
1.01
3.11
5.85
1.22

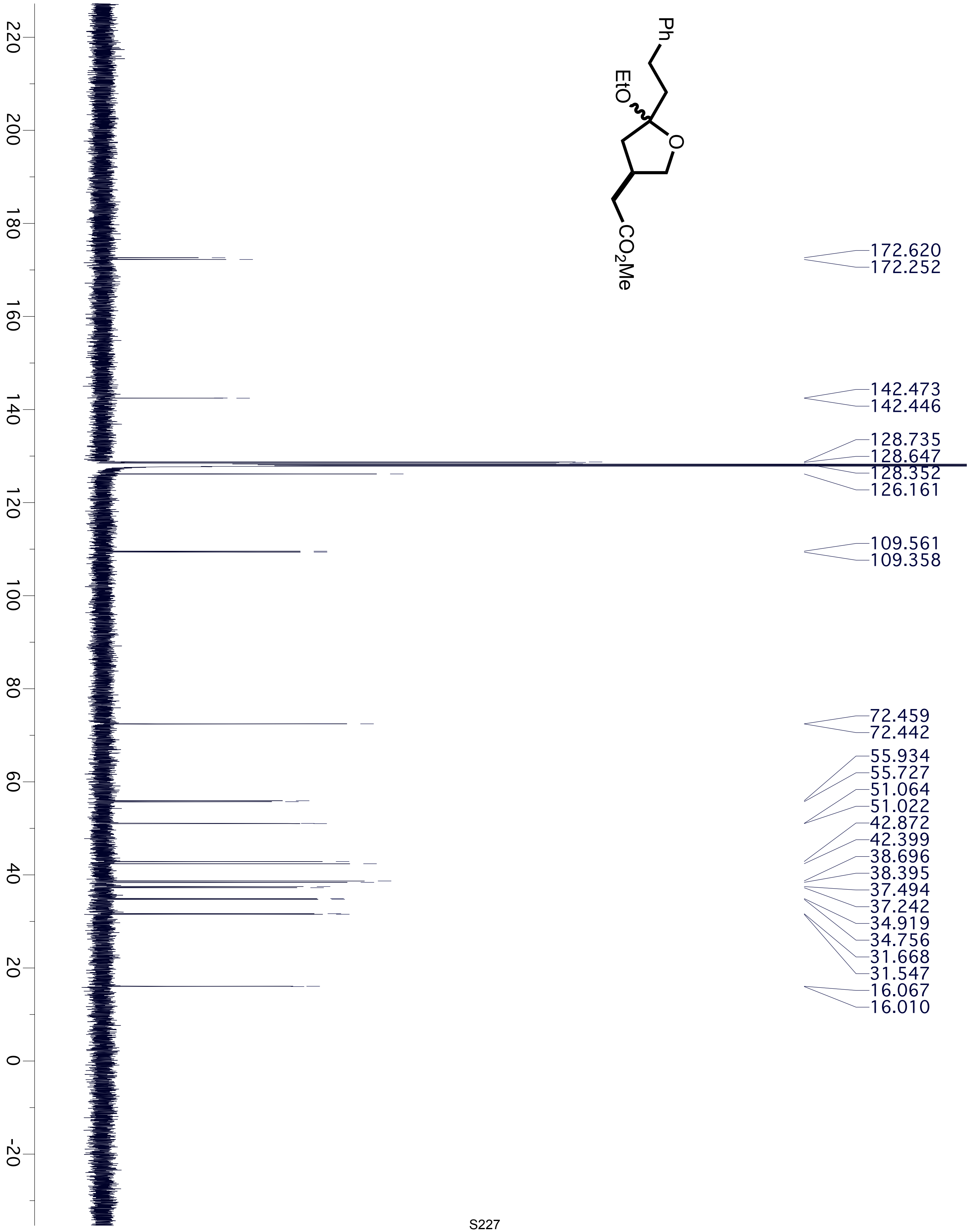
Ph
EtO
O
CO2Me
172.620
172.252
142.473
142.446
128.735
128.647
128.352
126.161
109.561
109.358
72.459
72.442
55.934
55.727
51.064
51.022
42.872
42.399
38.696
38.395
37.494
37.242
34.919
34.756
31.668
31.547
16.067
16.010
220
200
180
160
140
120
100
80
60
40
20
0
-20

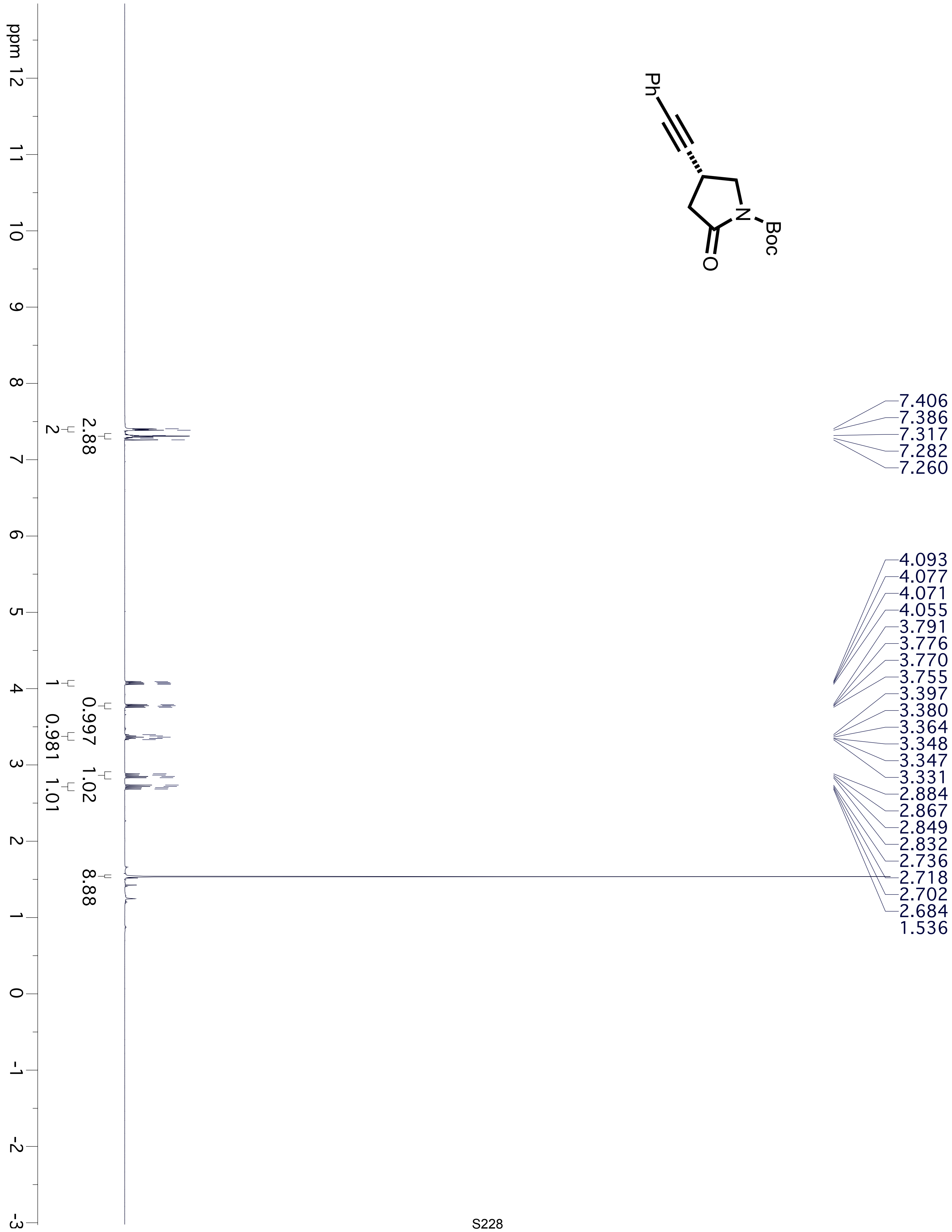
Ph
N
Boc
O
7.406
7.386
7.317
7.282
7.260
4.093
4.077
4.071
4.055
3.791
3.776
3.770
3.755
3.397
3.380
3.364
3.348
3.347
3.331
2.884
2.867
2.849
2.832
2.736
2.718
2.702
2.684
1.536
2.88
2
1
0.997
0.981
1.02
1.01
8.88
ppm
12
11
10
9
8
7
6
5
4
3
2
1
0
-1
-2
-3

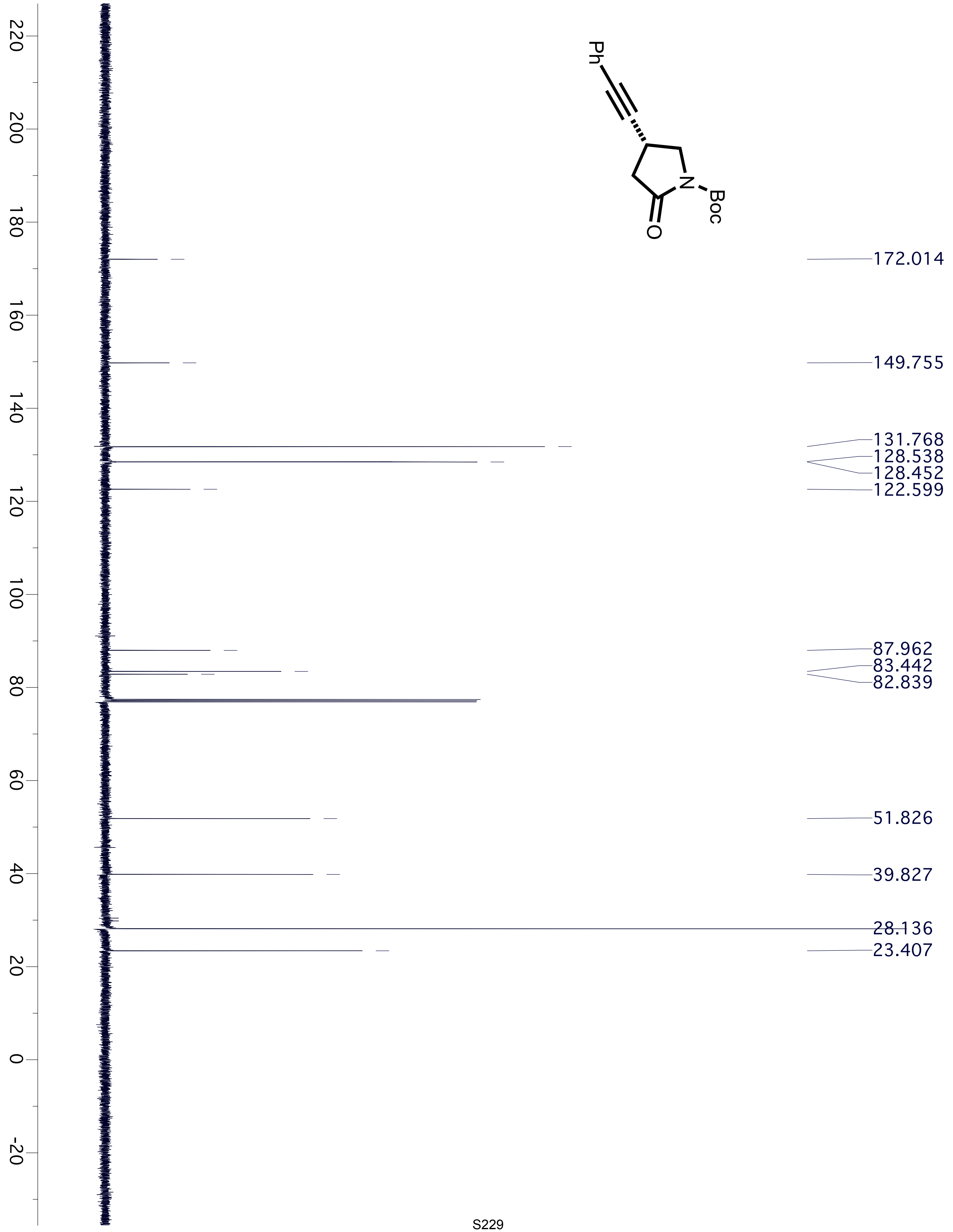
Ph
N
Boc
O
172.014
149.755
131.768
128.538
128.452
122.599
87.962
83.442
82.839
51.826
39.827
28.136
23.407
220
200
180
160
140
120
100
80
60
40
20
0
-20

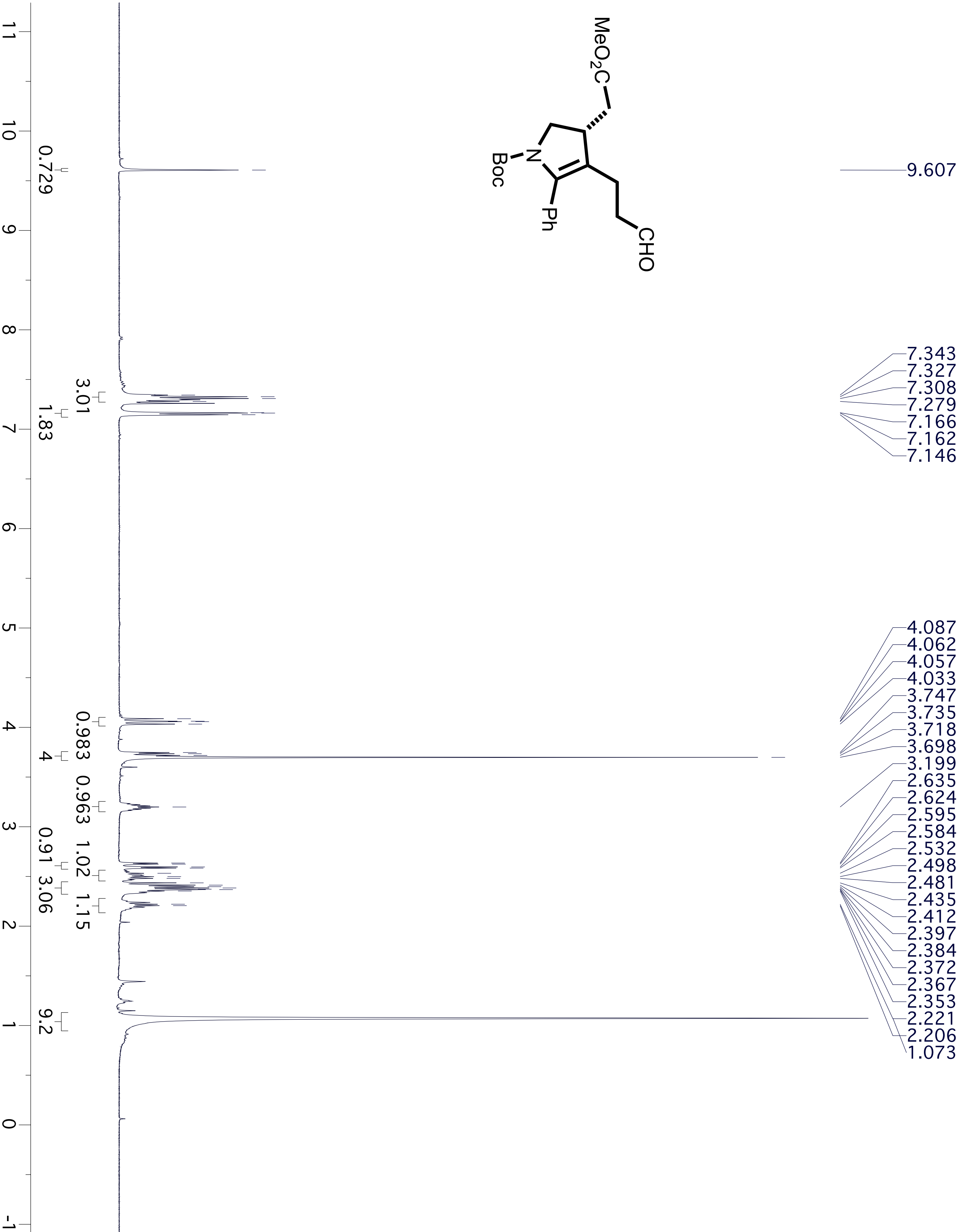
MeO2C
N
Boc
Ph
CHO
9.607
7.343
7.327
7.308
7.279
7.166
7.162
7.146
4.087
4.062
4.057
4.033
3.747
3.735
3.718
3.698
3.199
2.635
2.624
2.595
2.584
2.532
2.498
2.481
2.435
2.412
2.397
2.384
2.372
2.367
2.353
2.221
2.206
1.073
0.729
3.01
1.83
0.983
4
0.963
1.02
0.91
3.06
1.15
9.2

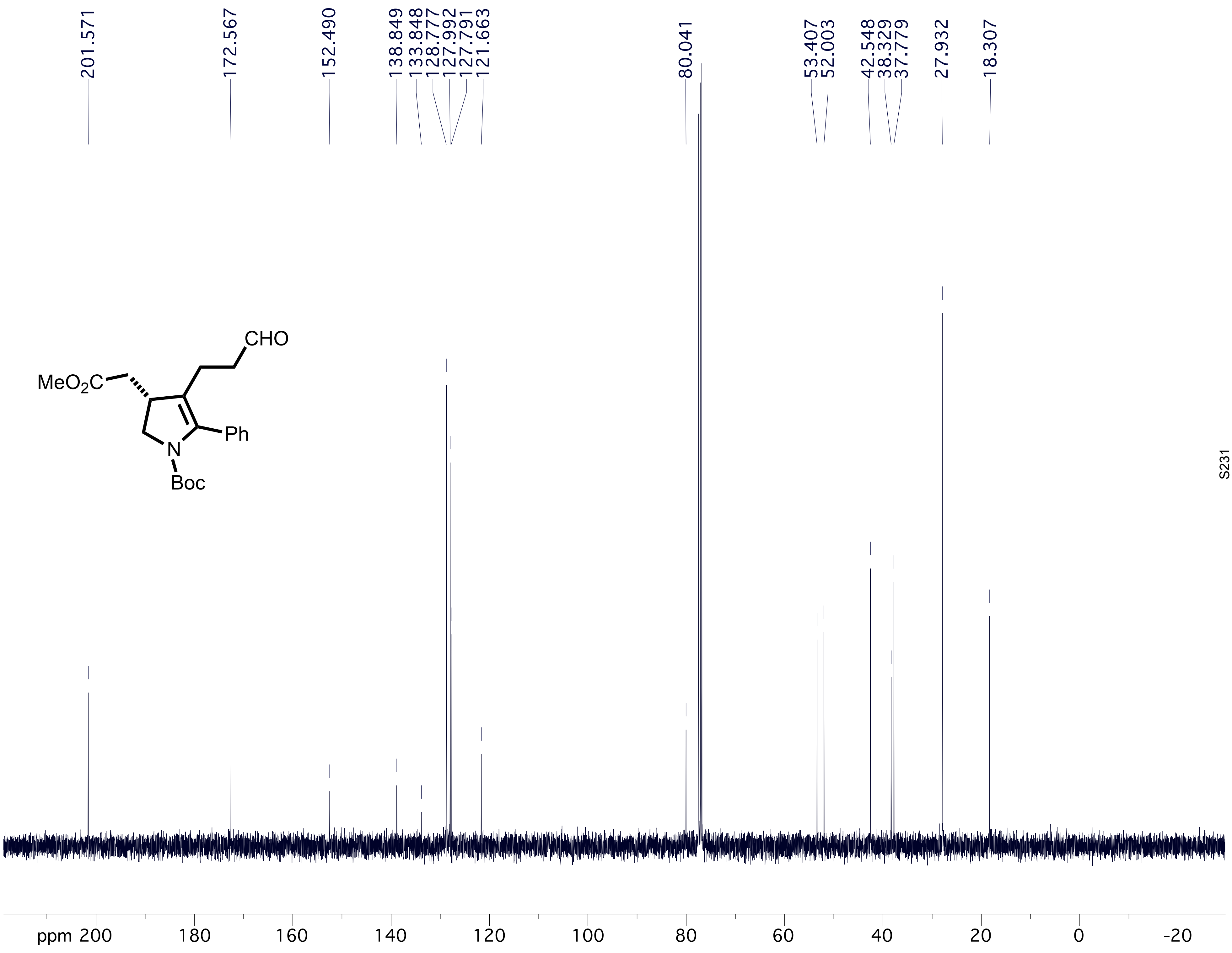
201.571
172.567
152.490
138.849
133.848
128.777
127.992
127.791
121.663
80.041
53.407
52.003
42.548
38.329
37.779
27.932
18.307
MeO2C
CHO
Ph
N
Boc
ppm 200
180
160
140
120
100
80
60
40
20
0
-20

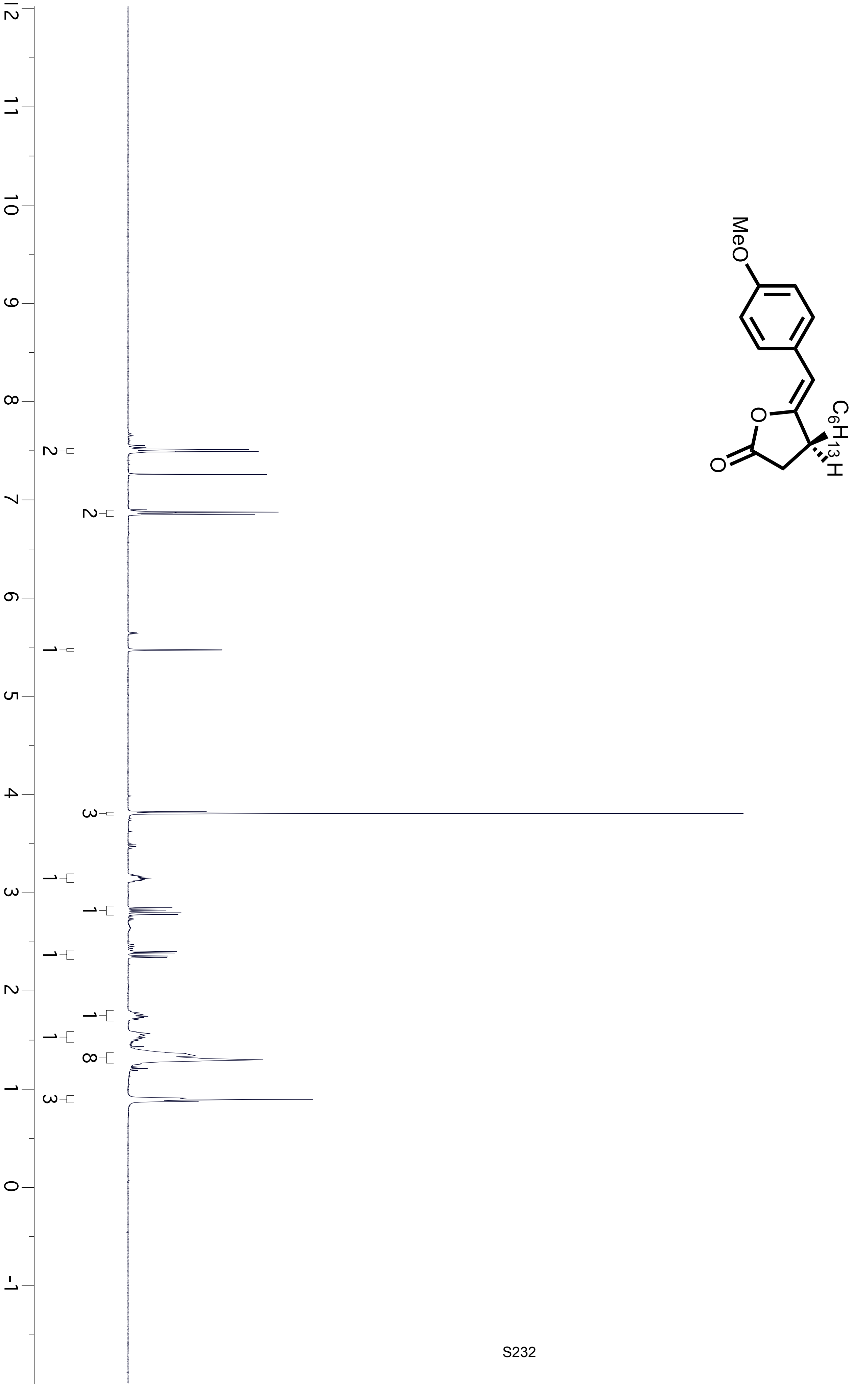
MeO
O
O
C6H13
H

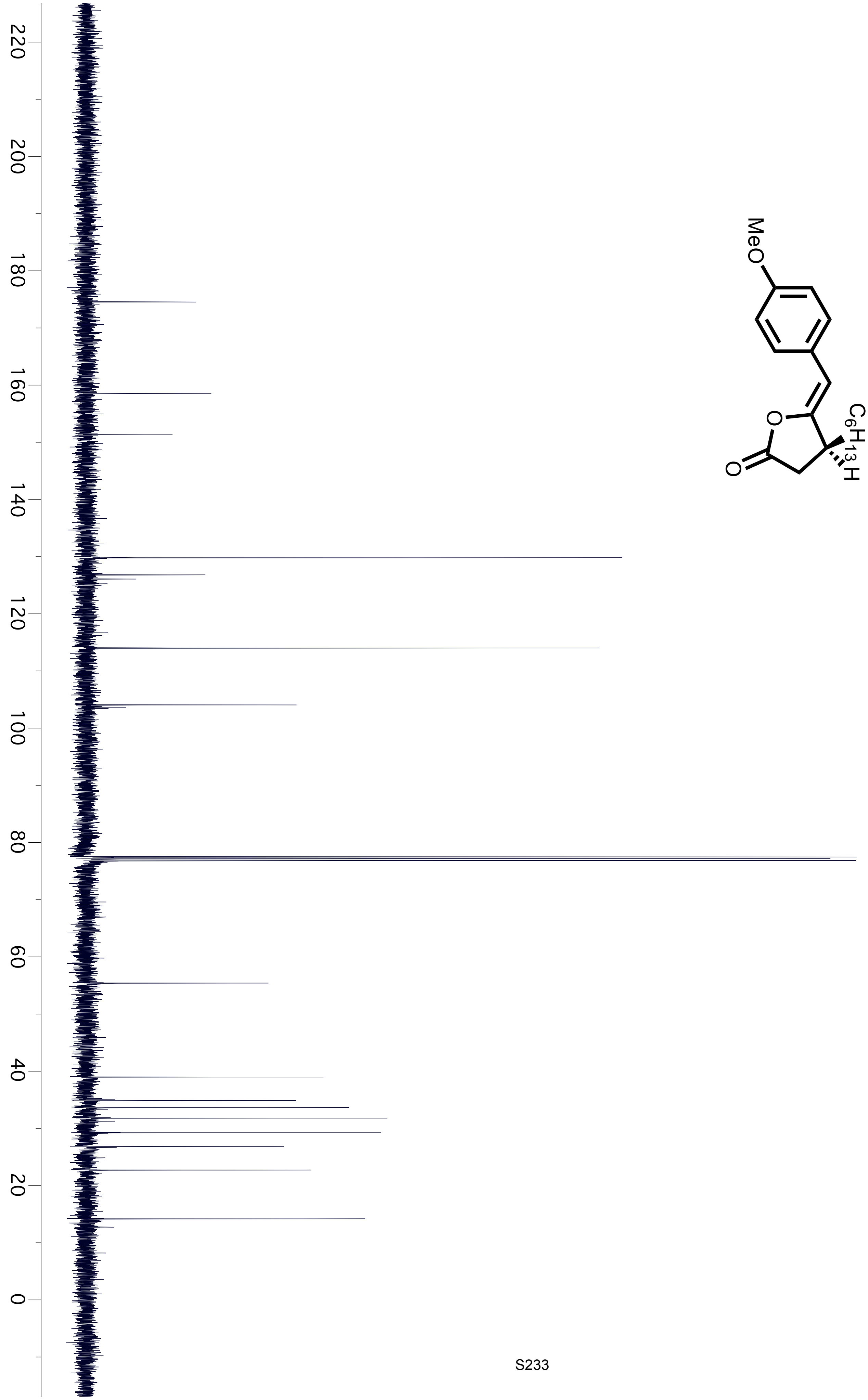

MeO
O
O
C6H13
H
220
200
180
160
140
120
100
80
60
40
20
0

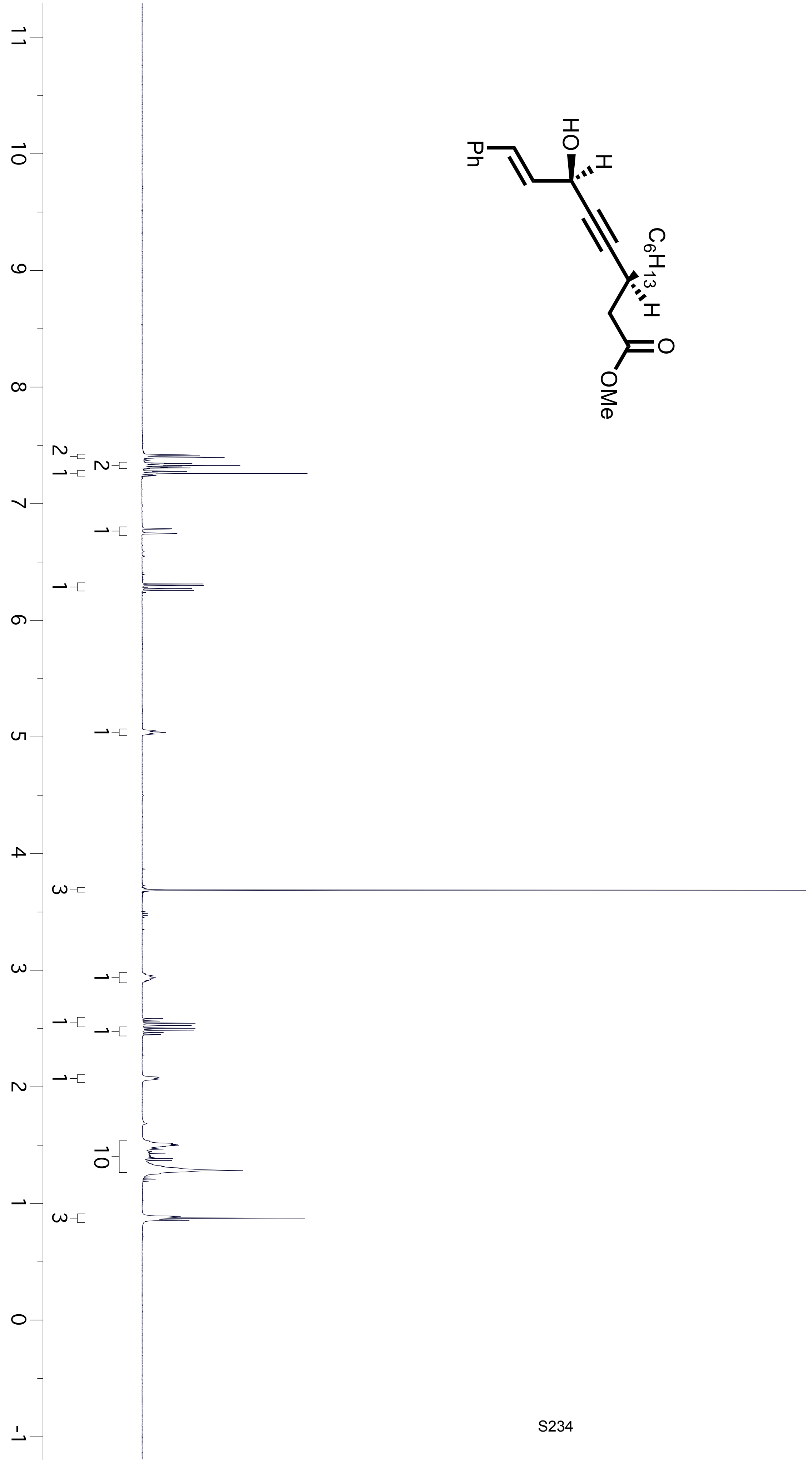
Ph
HO
H
C6H13
H
O
OMe
11
10
9
8
7
6
5
4
3
2
1
0
-1

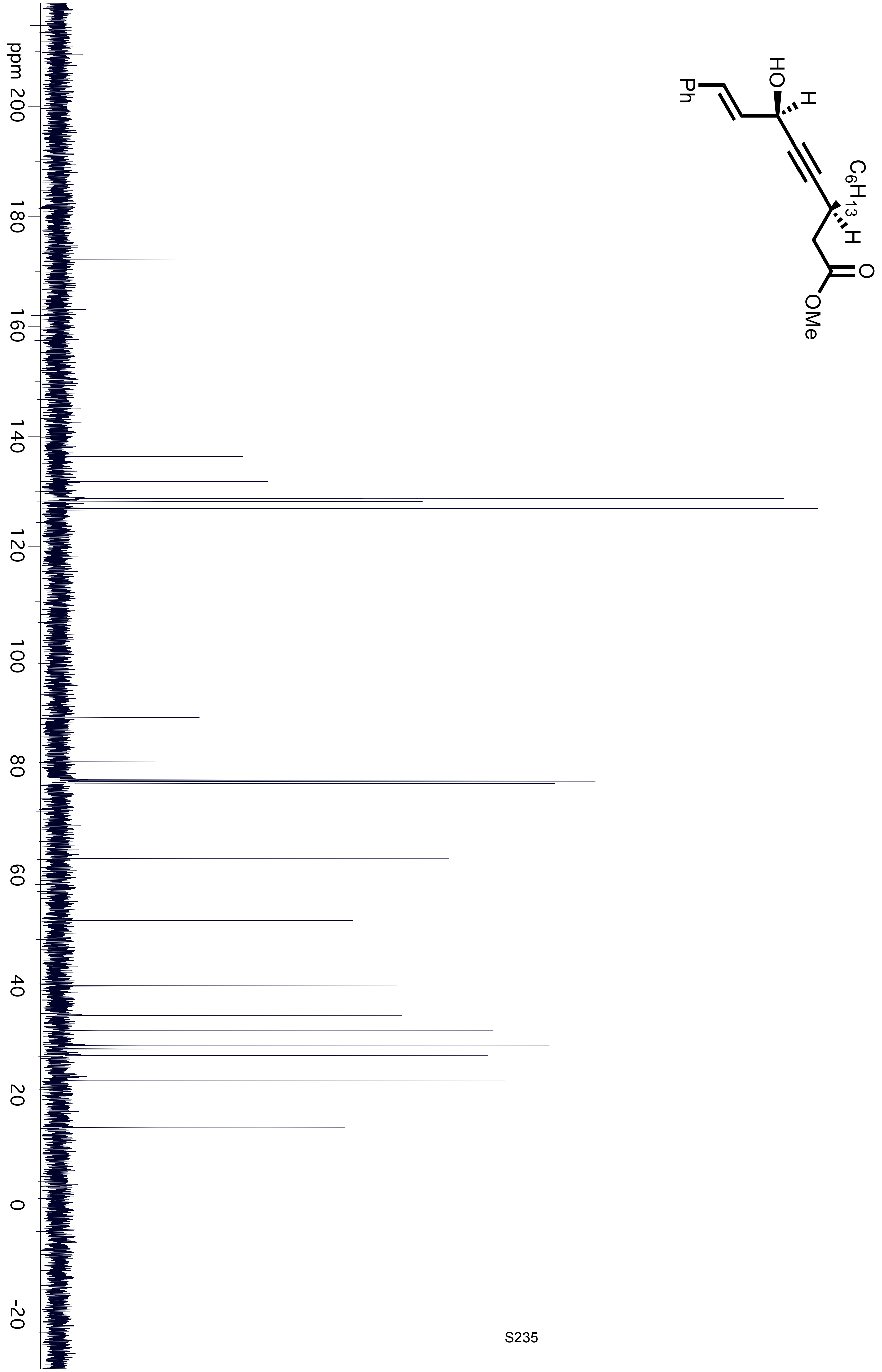
ppm 200
180
160
140
120
100
80
60
40
20
0
-20
HO
H
Ph
C_6H_{13}
H
O
OMe

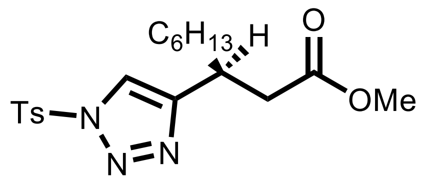
Ts
N
N
N
C6H13
H
O
OMe

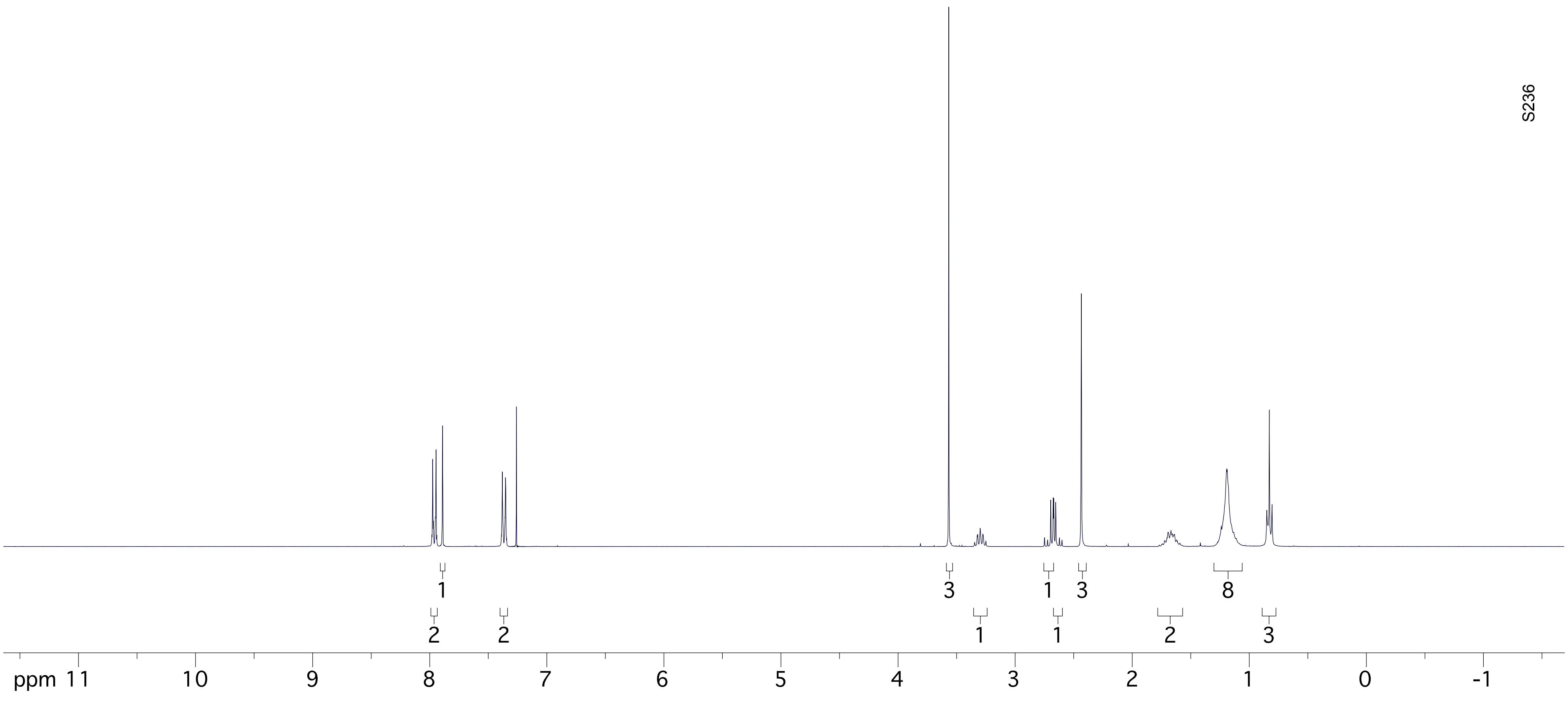
1
3
1
3
8
2
2
1
1
2
3
ppm 11
10
9
8
7
6
5
4
3
2
1
0
-1

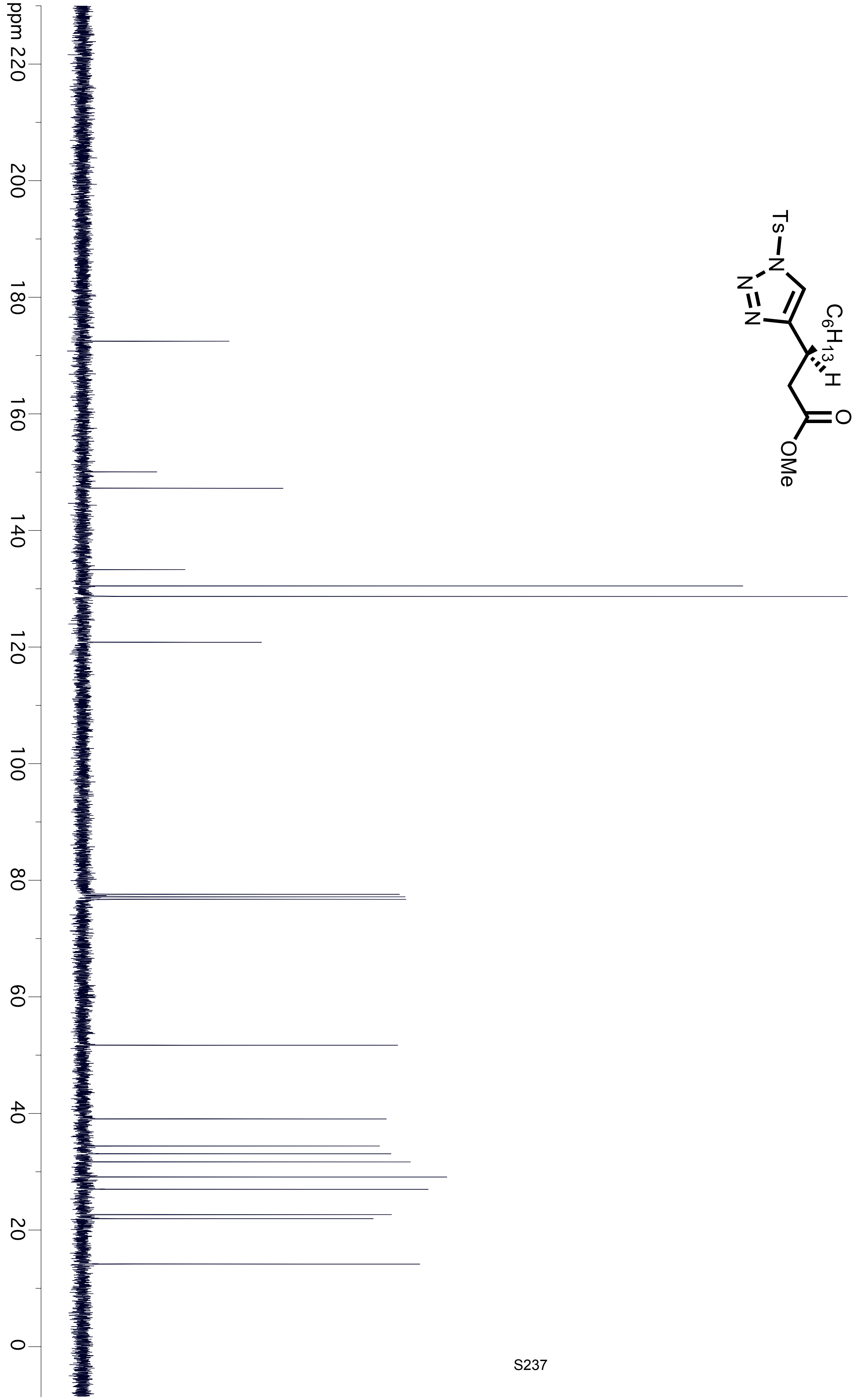
ppm 220
200
180
160
140
120
100
80
60
40
20
0
Ts
N
N
N
C6H13
H
O
OMe

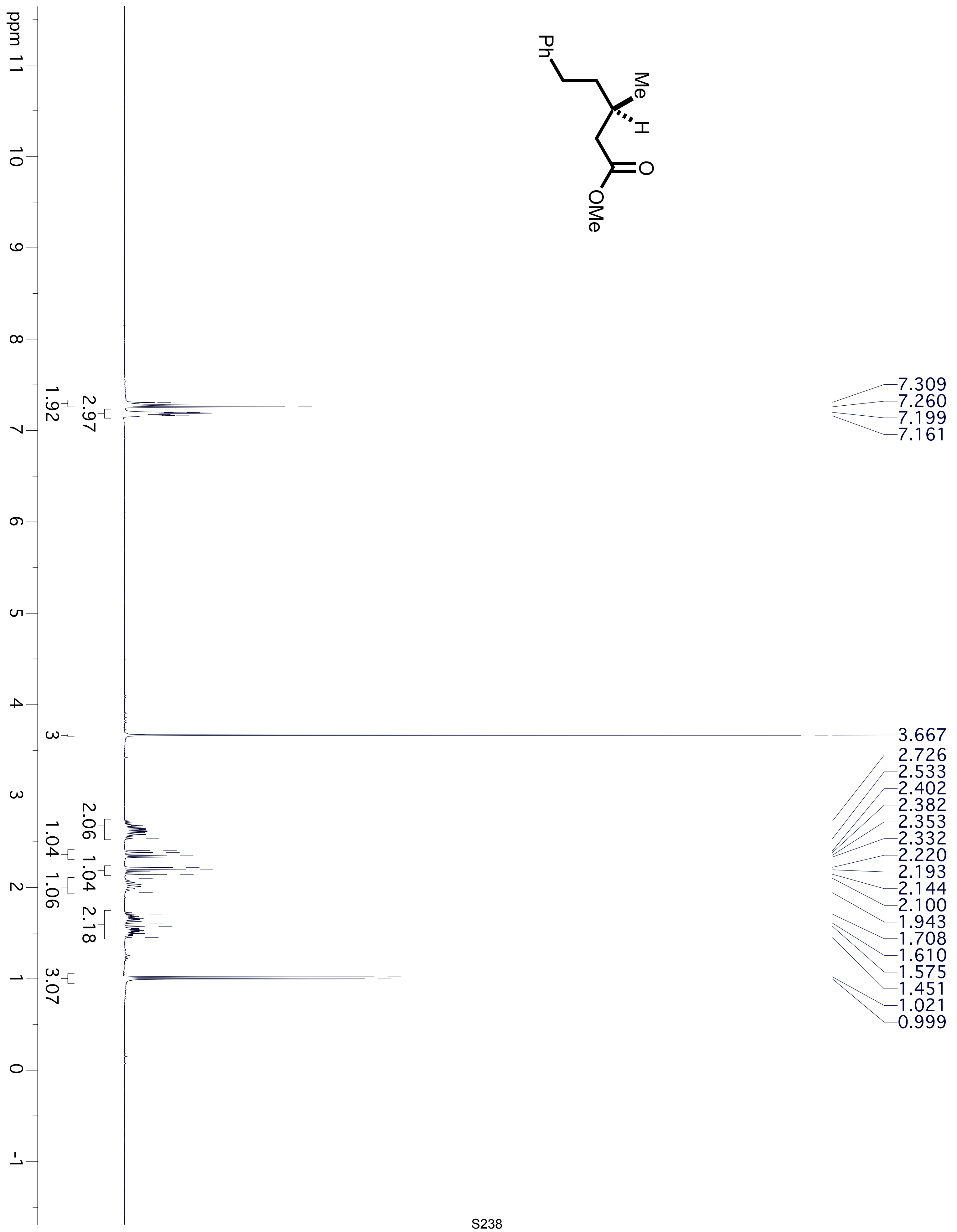

Ph
Me
H
O
OMe
7.309
7.260
7.199
7.161
3.667
2.726
2.533
2.402
2.382
2.353
2.332
2.220
2.193
2.144
2.100
1.943
1.708
1.610
1.575
1.451
1.021
0.999
1.92
2.97
3
2.06
1.04
1.04
1.06
2.18
3.07
ppm 11
10
9
8
7
6
5
4
3
2
1
0
-1

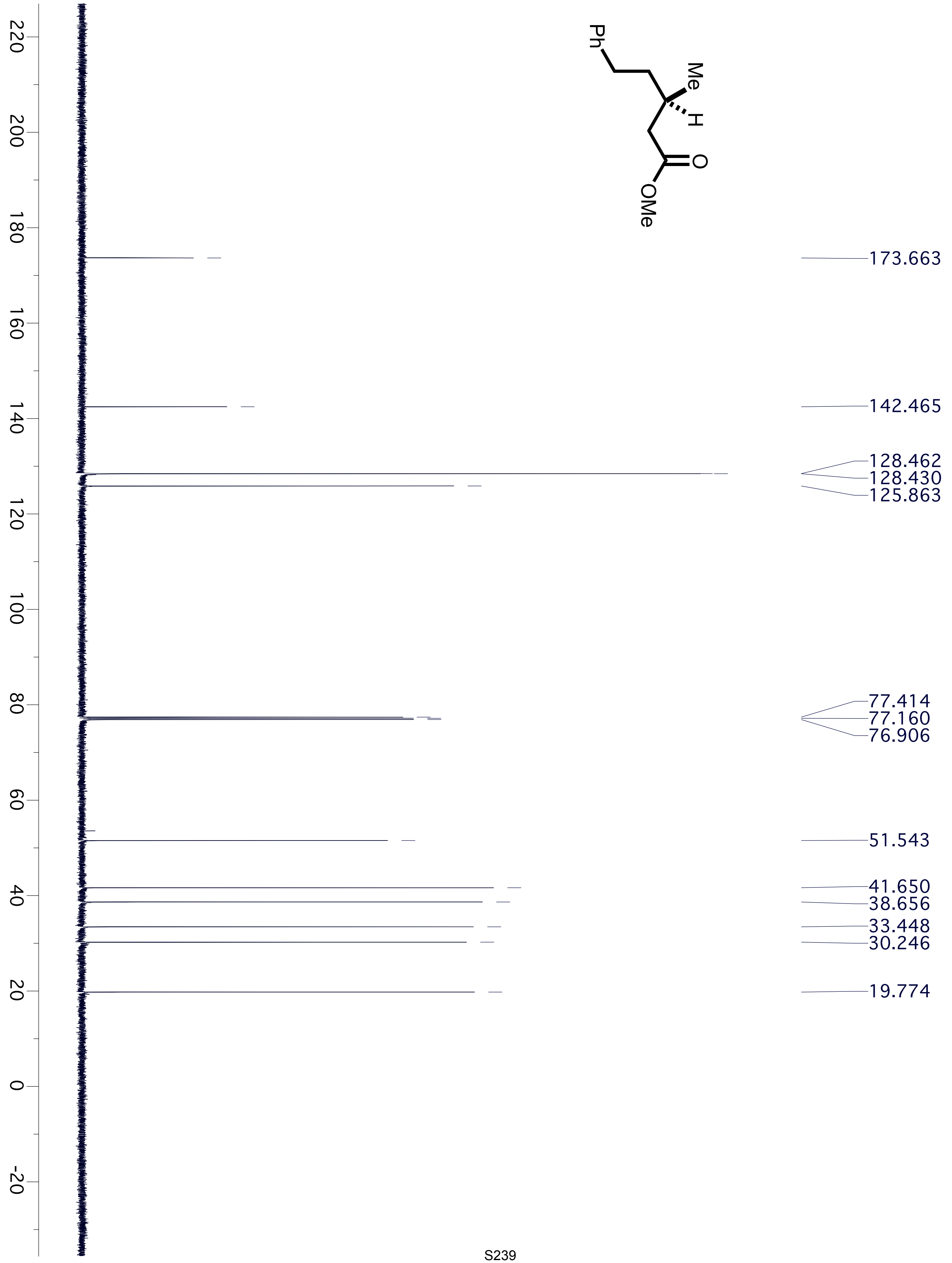
Ph
Me
H
O
OMe
173.663
142.465
128.462
128.430
125.863
77.414
77.160
76.906
51.543
41.650
38.656
33.448
30.246
19.774
220
200
180
160
140
120
100
80
60
40
20
0
-20

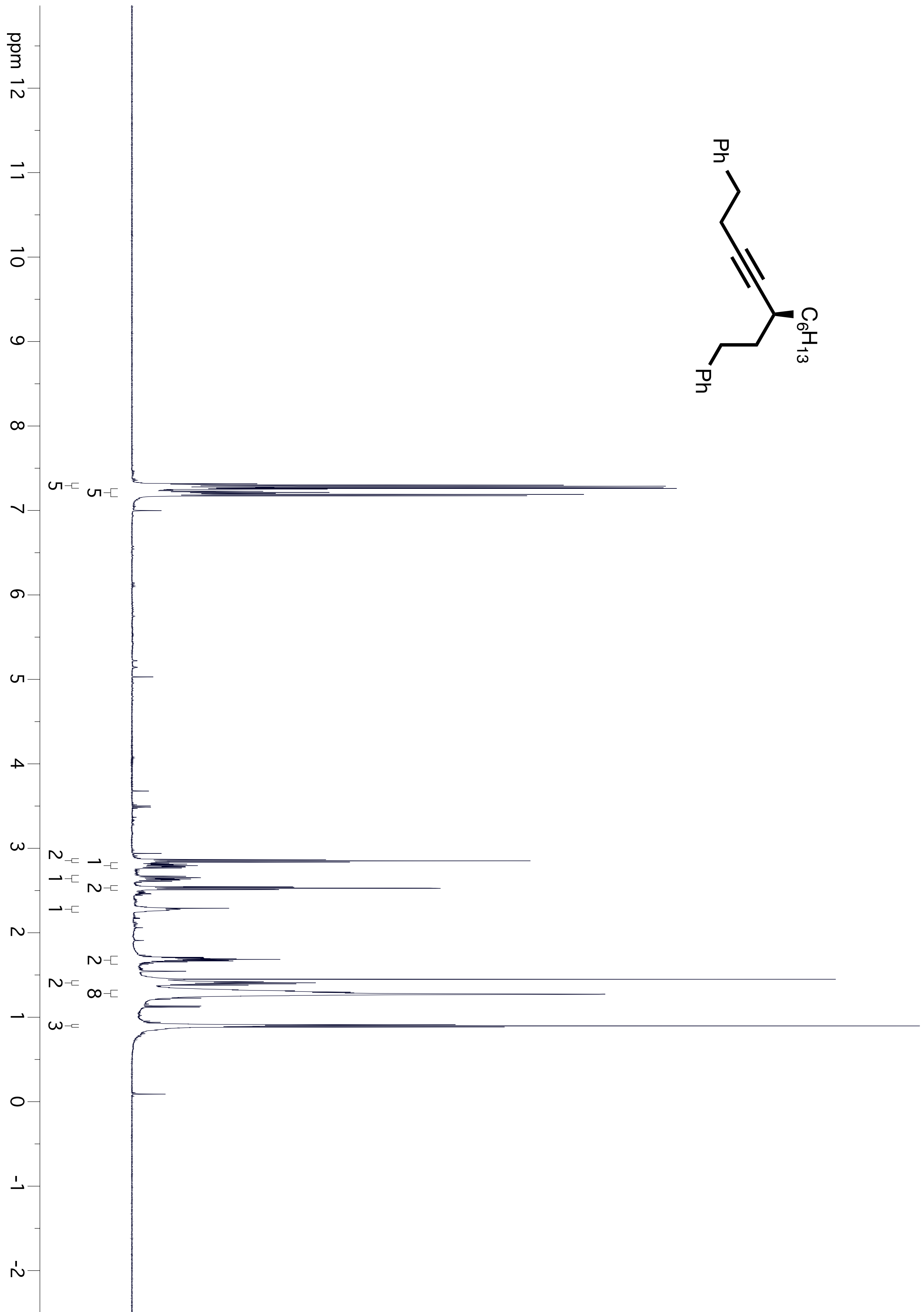
Ph
C6H13
Ph
ppm 12
11
10
9
8
7
6
5
4
3
2
1
0
-1
-2
-3

C13par

Pulse Sequence: s2pul

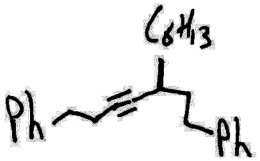

Solvent: CDCl3
Ambient temperature
User: 1-15-87
File: jtm-29-16-13C
INOVA-500 "ui500"

Relax. delay 1.000 sec
Pulse 48.5 degrees
Acq. time 1.500 sec
Width 33003.3 Hz
278 repetitions
OBSERVE C13, 125.6618455 MHz
DECOUPLE H1, 499.7505605 MHz
Power 44 dB
continuously on
WALTZ-16 modulated
DATA PROCESSING
Line broadening 2.0 Hz
FT size 131072
Total time 1 hr, 25 min, 38 sec

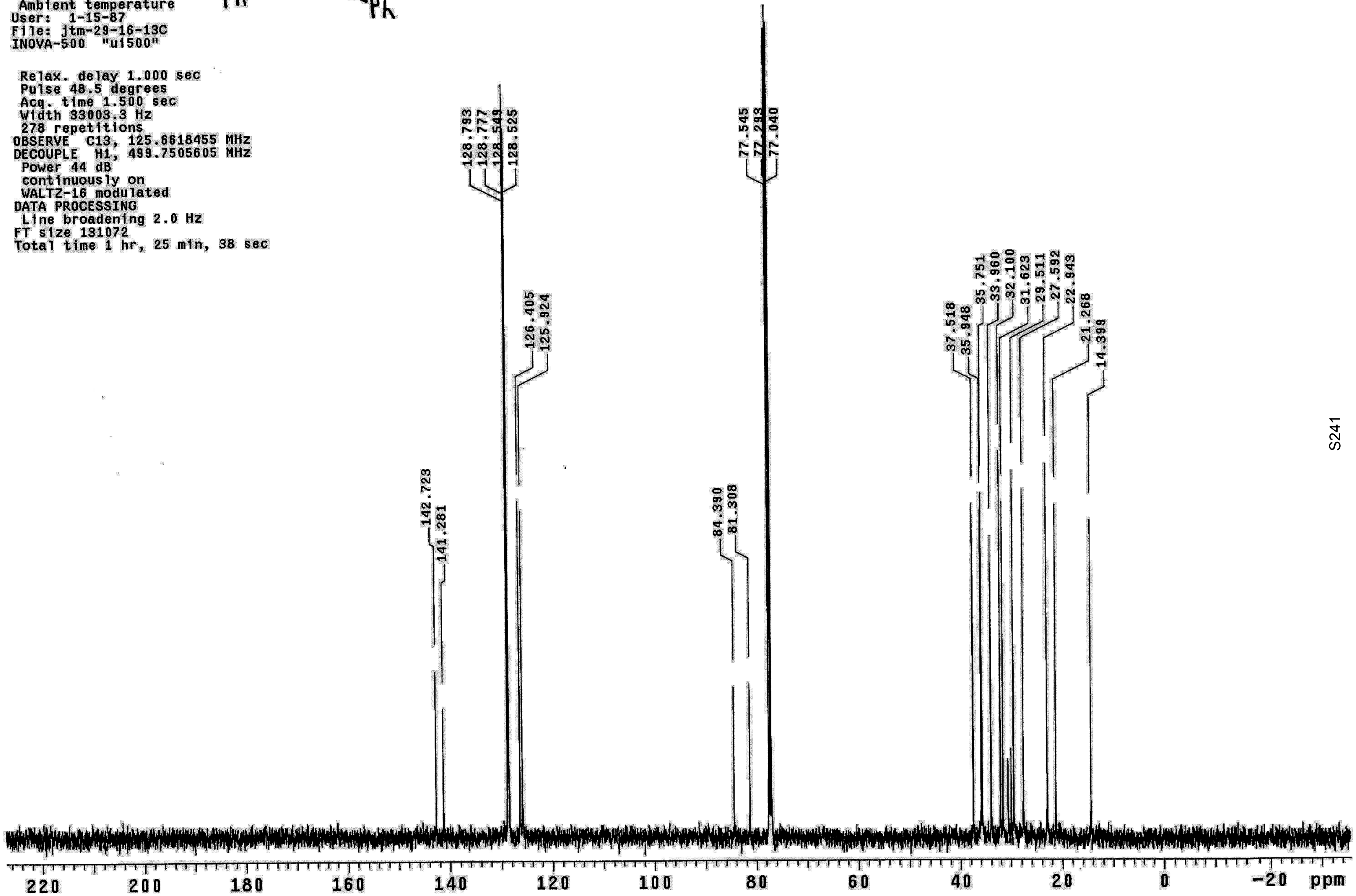

Supplement: Supplementary file 1 [file SC-007-C6SC01724J-s001.pdf]
